# Mechanistic Characterisation of Bacterial Terpene Synthases from Chitinophagaceae Producing Marine‐Type Diterpenes

**DOI:** 10.1002/anie.202517373

**Published:** 2025-11-13

**Authors:** Heng Li, Kexin Yang, Zhiyong Yin, Bernd Goldfuss, Jeroen S. Dickschat

**Affiliations:** ^1^ Kekulé Institute of Organic Chemistry and Biochemistry University of Bonn Gerhard‐Domagk‐Straße 1 Bonn 53121 Germany; ^2^ Department of Chemistry University of Cologne Greinstraße 4 Cologne 50939 Germany

**Keywords:** Biosynthesis, Enzymes, Substrate analogs, Terpenoids, Thermal reactions

## Abstract

Two neodolabellane‐type diterpene synthases from *Chitinophaga japonensis* (CjCsS and CjNdtS) in addition to one enzyme from *Dinghuibacter silviterrae* (DsJS) were investigated. CjCsS produces the instable diterpene alcohol chitinosphaerol, which degrades upon chromatographic purification on silica gel to known presphaerol and isosphaerodienes I and II. Despite seemingly solid evidence in the literature, for all three compounds a structural revision is presented. Treatment of chitinosphaerol with *N*‐bromosuccinimide resulted in the formation of the stable compounds bromosphaerols A and B. Phylogenetically unrelated CjNdtS catalyses the formation of neodolabella‐1(14),2,7‐triene, a compound that undergoes a remarkable thermal three‐step sequence by Diels–Alder reaction to thermocyclene A, followed by a water‐mediated rearrangement to thermocyclene B and double‐bond isomerisation to thermocyclene C. Japonene A was identified as a side product, but is the main product of DsJS, where it is accompanied by japonene B and anaerol B. The good production of anaerol B by DsJS allowed reinvestigation of its recently addressed biosynthesis as a trace product of peyssonnosol B synthase AbPS2. All structure elucidations in this study were secured through stereoselective deuteration experiments, and special isotopic labelling experiments in conjunction with density functional theory (DFT) calculations revealed the cyclisation mechanisms of the investigated enzymes.

## Graphical abstract

Six bacterial terpene synthases are reported, one of which produces instable chitinosphaerol, which is spontaneously transformed into several known natural products requiring structural revision. A second enzyme yields thermally labile neodolabella‐1(14),2,7‐triene, which upon heating shows sequential conversion into thermocyclenes A–C. The mechanisms of the enzymatic and thermal reactions were investigated experimentally and computationally.

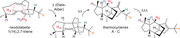

## Introduction

Terpenes are a fascinating class of compounds whose biosynthesis is in terms of the enzymology strikingly simple, but with respect to the reaction mechanisms one of the most complex processes in nature. All terpenes are made up from dimethylallyl diphosphate (DMAPP) and isopentenyl diphosphate (IPP) that can – because of their different reactivities as electrophile and nucleophile, respectively – be fused to geranyl diphosphate (GPP).^[^ ^1^, ^2^, ^3^ ^]^ Successive additions of IPP units then lead to farnesyl (FPP), geranylgeranyl (GGPP), geranylfarnesyl (GFPP) and higher oligoprenyl diphosphates. These acyclic and reactive compounds are converted by terpene synthases (TSs) through a tightly controlled multistep reaction with high stereoselectivity into polycyclic compounds with multiple stereogenic centers.^[^ ^4^, ^5^ ^]^ Type I TSs orchestrate this process through the abstraction of diphosphate, while type II enzymes make use of a different initiation mechanism involving protonation of the substrate.^[^ ^6^ ^]^ In both cases a cationic cascade reaction is promoted that makes largely use of the intrinsic substrate reactivity.

**Figure 1:**
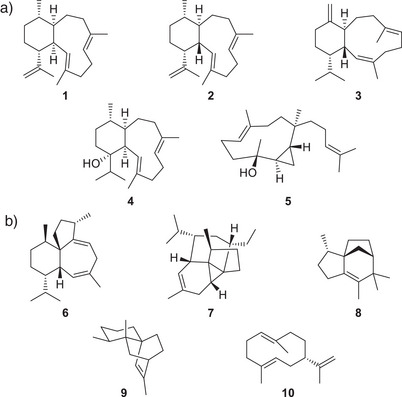
a) Diterpenes from bacterial DTSs also observed in corals and red algae. b) Terpenes produced by TSs from *C. japonensis* characterised in this study.

The first TSs have been characterised from higher plants,^[^ ^7^, ^8^ ^]^ fungi^[^ ^9^ ^]^ and bacteria,^[^ ^10^ ^]^ while only recent research has tapped protists,^[^ ^11^ ^]^ marine animals,^[^ ^12^, ^13^, ^14^ ^]^ red algae^[^ ^15^, ^16^ ^]^ and even viruses^[^ ^17^ ^]^ as sources of TSs. Interestingly, several recent studies have demonstrated that bacterial TSs can make products that are typically known from marine organisms. For instance, eunicellane diterpenes are not only widespread in corals,^[^ ^18^ ^]^ but also several bacterial diterpene synthases (DTSs) including Bnd4 from *Streptomyces* sp. CL12‐4 that produces benditerpe‐2,6,15‐triene (1),^[^ ^19^ ^]^ AlbS from *Streptomyces albireticuli* NRRL B‐1670 that catalyses the formation of albireticulene (2),^[^ ^20^ ^]^ MicA from *Micromonospora* sp. HM134 that generates microeunicellene (3),^[^ ^21^ ^]^ and CpDTS2 from *Chitinophaga pinensis* for chitinol (4)^[^ ^22^ ^]^ have been reported (Figure 1). In addition, CpDTS2 from *C. pinensis* produces palmatol (5),^[^ ^22^ ^]^ a diterpene alcohol that was previously isolated from the octocoral *Alcyonium palmatum*.^[^ ^23^ ^]^ Here we present three bacterial DTSs, two from *Chitinophaga japonensis* and one from *Dinghuibacter silviterrae* (Chitinophagaceae), that catalyse the formation of compounds known from corals and red algae, respectively. Our study includes a structural revision of several reported compounds, a deep mechanistic investigation of the producing enzymes through isotopic labelling experiments and DFT calculations, and exploration of the unusual reactivity of the isolated diterpenes.

## Results and Discussion

**Figure 2:**
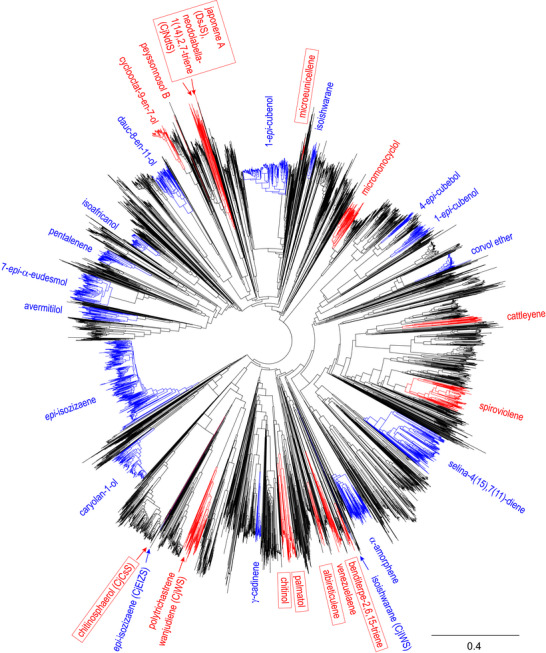
Phylogenetic tree constructed from 5000 bacterial TS homologs. Clades of homologous sesquiterpene synthases with at least one characterised member are shown in blue, clades of DTSs are shown in red. Red boxes highlight the clades containing the enzymes producing coral or algal type diterpenes, and arrows indicate all enzymes characterised in this study. The previously characterised enzymes ScEIZS and SlIWS fall into the branches labelled “*epi*‐isozizaene” and “isoishwarane”, respectively. The scale bar represents substitutions per site.

The genome of *Chitinophaga japonensis* DSM 13 484 encodes at least eleven type I TS homologs (Table S1) that all contain the highly conserved motifs required for enzyme functionality, sometimes with slight modifications (Figure S1). The function of one of these enzymes has been reported before as chitino‐2,5(6),9(10)‐triene synthase,^[^ ^24^ ^]^ while one enzyme showed high sequence identity to chitinol synthase from *C. pinensis* ^[^ ^22^ ^]^ and was not further investigated in this study. Two enzymes both with γ‐cadinene synthase from *C. pinensis* ^[^ ^25^ ^]^ being the closest characterised relative were not expressed. Two enzymes turned out to be inactive under standard incubation conditions with the substrates GPP, FPP, GGPP and GFPP, and three enzymes produced the known compounds wanju‐2,5‐diene (**6**)^[^ ^26^ ^]^ and polytrichastrene A (**7**, CjWS),^[^ ^27^ ^]^ (+)‐*epi*‐isozizaene (**8**, CjEIZS),^[^ ^28^ ^]^ and (+)‐isoishwarane (**9**)^[^ ^29^ ^]^ along with its biosynthetically related compounds germacrene A (**10**), detected as its Cope rearrangement product β‐elemene, aristolochene and valencene (CjIWS) (Figures S2–S4). The production of **8** and **9** is rather surprising, because the enzymes CjEIZS and CjIWS only show an amino acid sequence identity of 21% and 20% to the previously identified (+)‐*epi*‐isozizaene synthase from *Streptomyces coelicolor* (ScEIZS)^[^ ^28^ ^]^ and (+)‐isoishwarane synthase from *Streptomyces lincolnensis* (SlIWS),^[^ ^30^ ^]^ respectively, and occur in different branches of a phylogenetic tree constructed from 5000 bacterial TS homologs (Figure 2).

**Scheme 1:**
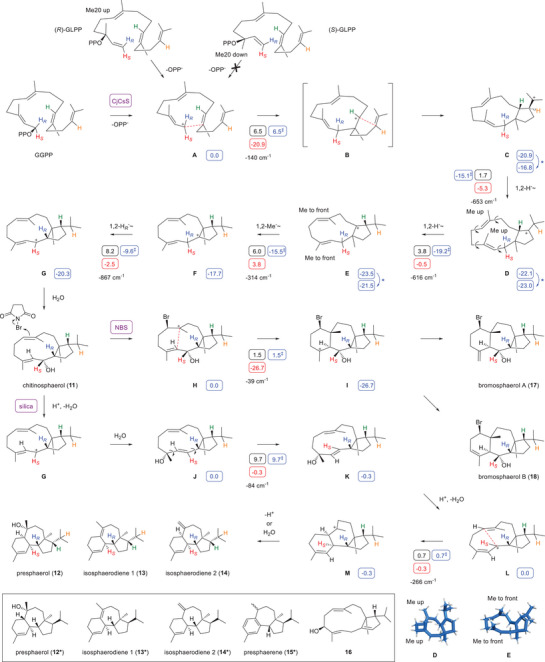
Cyclisation mechanism from GGPP to **11** and its chemcial transformation with NBS to **17** and **18**, or through treatment with silica gel to **12** – **14**. The previously assigned structures of 12* – 14* that are revised in this study and of related known compounds are shown in the box. The results from DFT calculations (wB97M‐V/Def2‐TZVPPD//B97D3/6–31G(d,p), 298 K) are given in small blue boxes (free (Gibbs) energies relative to **A** set to 0.0 kcal mol^−1^; ‡ denotes free energies of transition states relative to **A**), black boxes (free energies of activation, Gibbs reaction barriers), and red boxes (free reaction energies, all figures in kcal mol^−1^). Blue asterisks indicate required minor conformational changes between computed structures. Imaginary frequencies of transition states are given in cm^−1^. Selected computed structures are shown in blue at the bottom, the cartesian coordinates of other computed structures are given in the Supporting Information.

The nearest neighbour of CjEIZS is another enzyme from *C. japonensis* (CjCsS), with a sequence identity of only 29%, pointing to a different function (Figure S5). Purified CjCsS (Figure S6) did not accept GPP, FPP or GFPP, but converted GGPP into a diterpene alcohol (Figure S7). Attempts to isolate this compound by column chromatography on silica gel failed due to compound decomposition. Instead, the three decomposition products **12**, **13** and **14** were isolated (Scheme 1, Figures S8–S30, and Tables S3–S5). The alcohol 12* (presphaerol) was first reported from the red alga *Sphaerococcus coronopifolius*,^[^ ^31^ ^]^ and based on an X‐ray analysis of its dehydration product 13* its structure was later reassigned.^[^ ^32^ ^]^ This reassignment is in line with the structure of presphaerene (15*) from *S. coronopifolius*,^[^ ^33^ ^]^ that was secured by total synthesis.^[^ ^34^ ^]^ Compound 12* was later reisolated from the same species showing identical NMR data to our enzymatically prepared **12**,^[^ ^35^ ^]^ which unequivocally established the identity of the two materials, but our NMR‐based structure elucidation pointed to a different structure with a *cis* orientation of the *i*Pr and the Me substituents at the 5‐membered ring and a *cis* fusion between the 6‐ and the 7‐membered ring. These structural assignments were based on key NOESY correlations between H14 and H9β, H11 and H13β, and between H9β and Me19, that placed these hydrogens and Me19 in one hemisphere, while correlations between Me18 and H1, H9α, H12α and H13α, and between H1 and H6 located these hydrogens and Me18 in the opposite hemisphere (Figure S31). Analogous NOESY correlations indicated that the originally assigned structures of 13* and 14*.^[^ ^36^ ^]^ should also be corrected to those of **13** and **14** (Figures S32 and S33). These structural revisions argue against seemingly strong evidence, and therefore a few more explanations are required: 1.) Despite the generally high reliability of crystallographic methods, the crystal structure of 13* published in 1981^[^ ^32^ ^]^ may still be erroneous. 2.) The paper reporting the synthesis of 15* claims the identity of the synthetic and the natural compound, based on a comparison of NMR data.^[^ ^34^ ^]^ However, the NMR solvent used for the natural product has not been reported, the comparison is only based on a few ^1^H‐NMR signals, and the match is not very good. Conclusively, the previously assigned structures were not built on rigorous evidence and we suggest the above mentioned structural revisions for **12** – **14**. This view is further supported by biosynthetic arguments discussed below.

To determine the structure of the instable precursor to **12** – **14**, the compound was extracted from an incubation of GGPP with CjCsS and the NMR spectra of the crude product were recorded, revealing a purity of > 95%. The NMR spectra showed the presence of two conformers and strong peak broadening even at elevated temperature (348 K), but low temperature measurements (238 K) gave substantial improvements and allowed for a structure elucidation to the level of the planar structure (Tables S6 and S7 and Figures S34–S41). Still, assignment of the relative configuration by NOESY proved to be difficult, but a strong NOESY correlation between H2 and H4 was observed for both conformers, establishing their 2*E* configuration that was also supported by the ^13^C‐NMR shifts of Me20 well below 20 ppm (16.06 ppm and 15.96 ppm, respectively). For determination of the relative configuration the bicyclic ring system of the alcohol named chitinosphaerol (**11**) was rigidified through a reaction with N‐bromosuccinimide (NBS), resulting in two products that were isolated and structurally characterised as bromosphaerols A (**17**) and B (**18**) (Scheme 1, Tables S8 and S9, Figures S42–S57), allowing to conclude on the full structure of **11**. The structurally similar diterpene alcohol **16** has previously been isolated from an unidentified Australian soft coral.^[^ ^37^ ^]^ All these compounds showed the same *cis* substitution of the Me and the *i*Pr group at the 5‐membered ring, confirming the structural reassignments for **12** – **14**. The characterised DTS was named *Chitinophaga japonensis* Chitinosphaerol Synthase (CjCsS).

**Scheme 2:**
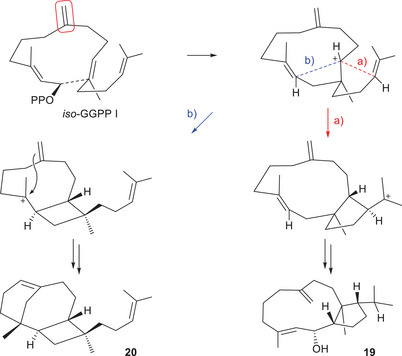
Conversion of *iso*‐GGPP I into **19** and **20** with CjCsS.

To gain further insights into CjCsS catalysis and the nature of its products, the GGPP analog *iso*‐GGPP I^[^ ^38^ ^]^ with the C6 = C7 double bond shifted to C7 = C19 was converted with CjCsS yielding isochitinosphaerol (**19**) and chitinoxenene (**20**) (Scheme 2, Tables S10 and S11, Figures S58–S74). Notably, **19** is a double bond isomer of **11**, but did not show peak broadening in the NMR spectra, allowing for full elucidation of its structure. The isolation of **20** impressively demonstrates how the small structural change in *iso*‐GGPP I can result in dramatic consequences for the product structure (the name “chitinoxenene” indicates a product that is foreign to CjCsS; greek ξενοσ = foreign). Its stereostructure is a direct consequence of the *iso*‐GGPP I fold required for **19**.

The absolute configurations of the isolated compounds were investigated through stereoselective labelling experiments (isotopic labelling experiments are summarised in Table S12). Our strategy makes use of enantioselectively deuterated precursors whose enzymatic conversion result in chiral products in which specific hydrogens in CH_2_ groups are replaced by deuterium. Because the configurations at the deuterated carbons are known, the absolute configurations of the TS products can be resolved through determination of the relative configuration between the naturally present stereogenic centres and the introduced deuterated anchors. After a full NOESY‐based assignment of the diastereotopic hydrogens in CH_2_ groups the approach only requires to determine which hydrogen is replaced by deuterium, and for this purpose the enantioselectively deuterated precursors carry an additional ^13^C‐label at the deuterated carbon that allows for a sensitive detection by HSQC analysis even in small scale reactions (typically 1 mg of the labelled precursor is used), without the need of compound purification. In the present case, the strong peak broadening in the NMR spectra of **11** prevented its direct analysis, but instead all samples were converted with NBS to obtain labelled **17** and **18**, or by treatment with silica gel to yield labelled **12** – **14**. The small scale enzymatic conversions of DMAPP and (*E*)‐ or (*Z*)‐(4–^13^C,4–^2^H)IPP^[^ ^39^ ^]^ with *Streptomyces cyaneofuscatus* GGPP synthase^[^ ^40^ ^]^ (GGPPS) and CjCsS, followed by treatment with NBS were unsuccessful, but the strategy using silica gel treatment showed an efficient conversion, revealing the absolute configurations of **12** – **14**, respectively, as depicted in Scheme 1 (Figures S75–S77). Additional experiments with (*R*)‐ and (*S*)‐(1–^13^C,1–^2^H)IPP^[^ ^41^ ^]^ that were transformed with *Escherichia coli* isopentenyl diphosphate isomerase (IDI),^[^ ^42^ ^]^ GGPPS and CjCsS and subsequent silica mediated conversion confirmed the assigned absolute configurations (Figures S78–S80).

The proposed cyclisation mechanism to **11** (Scheme 1) starts with the ionisation of GGPP to **A**, followed by a 1,11‐cyclisation to **B** and 10,14‐cyclisation to **C**. Two sequential 1,2‐hydride shifts and a Me group migration may proceed through **D** and **E** to **F** that upon a third 1,2‐hydride transfer to **G** and capture with water results in **11**. This mechanistic proposal was investigated by isotopic labelling experiments and computationally. Experimental evidence for hydride migrations can efficiently be obtained using labelled substrates in which the migrating hydrogen is substituted by deuterium and its target position by ^13^C, which will result in slightly upfield shifted triplet signals as a result of ^2^H‐^13^C spin coupling. In the present case enzymatically formed **11** was again transformed into the mixture of **12** – **14** by treatment with silica gel before product analysis. Using this approach, the conversion of (3–^13^C,2–^2^H)DMAPP^[^ ^43^ ^]^ and IPP with GGPPS and CjCsS confirmed the 1,2‐hydride shift from **C** to **D** (Figure S81, observed for **13**: ^1^ *J*_C,D_ = 18.9 Hz, Δ*δ* = –0.45 ppm), establishing a sequence of two 1,2‐hydride shifts instead of one 1,3‐hydride transfer for the transformation from **C** to **E**. The enzymatic conversion of (7–^13^C)FPP^[^ ^44^ ^]^ and (1,1–^2^H_2_)IPP^[^ ^45^ ^]^ gave evidence for the 1,2‐hydride shift from **F** to **G** (Figure S82, for **12**: ^1^ *J*_C,D_ = 19.3 Hz, Δ*δ* = –0.58 ppm). The experiments with (*R*)‐ and (*S*)‐(1–^13^C,1–^2^H)IPP for the absolute configuration determination also confirmed the selective migration of the 1‐*pro*‐*R* hydrogen in this step (Figure S83).

The starting conformation of GGPP was verified through the incubation of (*R*)‐ and (*S*)‐geranyllinalyl diphosphate (GLPP).^[^ ^45^ ^]^ GLPP is an intermediate in diterpene biosynthesis that allows the installation of 2*Z*‐configured double bonds in diterpenes through rotation of its vinyl group prior to cyclisation.^[^ ^46^ ^]^ While cation **A** can also be formed from GLPP by diphosphate abstraction, only (*R*)‐GLPP can adopt the same conformation with Me20 pointing up, while with (*S*)‐GLPP this Me group should point down. The experiment demonstrated a much more efficient conversion of (*R*)‐GLPP in comparison to (*S*)‐GLPP into **11** (Figure S84), supporting a starting conformation of GGPP with Me20 up.

The cyclisation mechanism to **11** was further refined through DFT calculations (Scheme 1, Table S13 and Figure S85). The results revealed a direct 1,11–10,14‐cyclisation from **A** to **C** with skipping of intermediate **B**. All proposed steps were associated with low to moderate reaction barriers with the highest barrier found for the 1,2‐hydride shift from **F** to **G** (8.2 kcal/mol), and the overall process is with Δ*G* = −20.3 kcal mol^−1^ strongly exergonic. Moreover, an interesting conformational change was observed in the transformation from **D** to **E**. While the Me groups Me19 and Me20 point up in **D**, the 1,2‐hydride shift to **E** results in a conformational change in the macrocycle (computed structures are shown at the bottom of Scheme 1). Ultimately, in **G** both Me groups point to the front and stand almost parallel to the plane defined by the cyclopentane ring. Chitinosphaerol (**11**) in the analogous conformation with coplanar π‐systems nicely explains the formation of **17** and **18** through the bromonium ion **H** and cyclisation to **I** for which the computations demonstrated a nearly barrierless and strongly exergonic reaction. Also the acid (silica gel) catalysed formation of **12** – **14** can be explained from the same conformer of **11**. The protonation induced elimination of water leads to **G** that cannot cyclise directly to a tricyclic intermediate, because of its 2*E*‐configured bond with partial double bond character. After attack of a nucleophile such as water at C3 a conformational change in **J** can lead to **K** that upon reionisation to **L** can now cyclise to **M** as the direct precursor of **12** – **14**. According to computational data, the conformational change (**J**‐**K**) can proceed through a moderate barrier, while the cyclisation reaction (**L**‐**M**) is nearly barrierless.

**Scheme 3:**
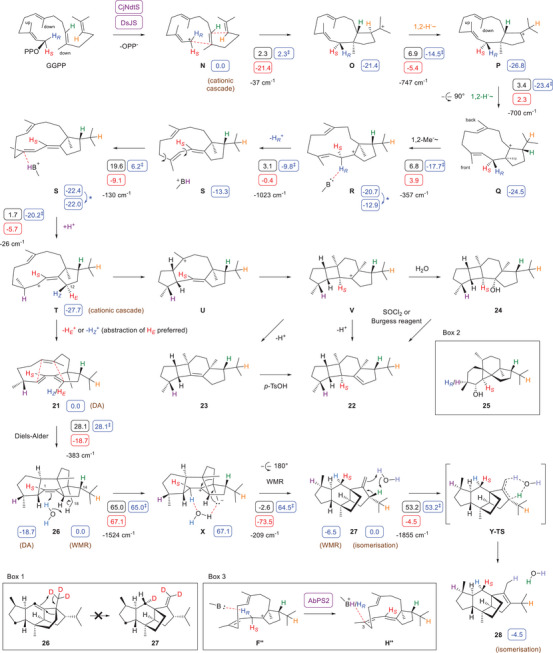
Cyclisation mechanism of CjNdtS and DsJS. Cyclisation from GGPP to **21** – **24**, and thermal rearrangement of **21** to **28**. Box 1 shows a hypothetical concerted process with intramolecular proton transfer that was excluded experimentally. Box 2: structure of peyssonnosol B (**25**). Box 3: deprotonation‐reprotonation sequence in the biosynthesis of **25**. DFT calculations (wB97M‐V/Def2‐TZVPPD//B97D3/6–31G(d,p), 298 K) were performed for four aspects, including the cationic cyclisation cascade from **N** to **T**, the Diels–Alder (DA) reaction from **21** to **26**, the Wagner–Meerwein rearrangement (WMR) from **26** to **27**, and the double bond isomerisation from **27** to **28**. Their start and end points are labelled accordingly, with starting structures set to 0.0 kcal mol^−1^ (energy data are only comparable within the four sections, not in between them). For further explanations regarding computational data cf. the legend of Scheme 1.

A second DTS of unknown function from *C. japonensis* efficiently converted GGPP, but did not accept GPP, FPP or GFPP. GC/MS analysis of the products showed significant broadening of the chromatographic peaks, suggesting a thermal reaction caused by the heat impact during GC separation (Figure S86).^[^ ^47^ ^]^ Compound isolation and structure elucidation revealed the structures of neodolabella‐1(14),2,7‐triene (**21**) for the main and japonene A (**22**) for a minor product (Scheme 3, Tables S14 and S15, Figures S87–S102), which characterised the DTS as *Chitinophaga japonensis* Neodolabella‐1(14),2,7‐triene Synthase (CjNdtS). To investigate the nature of the thermal reaction product, a sample of **21** dissolved in Ph_2_O was heated in a sealed tube for 5 h to 230 °C, resulting in **27** (Table S16, Figures S103–S110). The surprising structure of this compound can be explained through a Diels‐Alder (DA) reaction of **21** to a hypothetical intermediate **26**, followed by a skeletal rearrangement (Scheme 3). For this rearrangement different mechanistic hypotheses were experimentally tested. A first attempt addressed the possibility of a reaction from **26** to **27** proceeding through a concerted intramolecular proton transfer and skeletal rearrangement (Box 1 in Scheme 3). This initial hypothesis was falsified experimentally by an incubation of (10,10,10–^2^H_3_)GPP, synthesised as shown in Scheme S1, and (1–^13^C)IPP^[^ ^40^ ^]^ with GGPPS and CjNdtS, and thermal conversion of the enzyme product. The product **27** only showed a doublet for C1 in the ^13^C‐NMR originating from a ^3^ *J*_C,C_ coupling, but no triplet from a ^1^ *J*_C,D_ coupling, and a loss of one deuterium in the step from **26** to **27** by GC/MS, disfavouring an intramolecular deuterium shift (Figure S111). The second hypothesis included protonation of **26** by an external acid to promote a skeletal rearrangement followed by deprotonation to **27**. To investigate the protonation of **26**, a heating experiment with **21**, enzymatically generated from (1–^13^C)GGPP^[^ ^48^ ^]^ and dissolved in Ph_2_O saturated with D_2_O was performed. This experiment did not only yield the expected labelled **27**, but also a newly observed compound **28** not seen in heating experiments without water addition. The NMR analysis of **27** confirmed incorporation of a proton from the medium at C1 (^1^ *J*_C,D_ = 19.5 Hz, Δ*δ* = –0.40 ppm), while HSQC analysis of this product revealed the protonation of **26** from the sterically less hindered convex side (*Si* face attack) (Figure S112). This finding was confirmed in another labelling experiment using (*S*)‐(1–^13^C,1–^2^H)GGPP^[^ ^45^ ^]^ in H_2_O (Figure S113), which additionally demonstrated retainment of the 1‐*pro*‐*S* hydrogen in **27** (this question is addressed in more detail below). Shorter reaction times and lower temperatures (heating for 2 h at 180 °C) allowed for isolation of the intermediate DA adduct **26** for its structural characterisation (Table S17, Figures S114–S120). Heating to 230 °C over prolonged reaction times (9 h), again with addition of water to promote its formation, resulted in an almost complete conversion into **28** (Figure S121). The accumulated material was isolated and identified as the double bond isomer of **27** (Table S18 and Figures S122–S129). Additional heating experiments demonstrated that **26** can be converted into **27**, while heating of **27** resulted in the formation of **28**, suggesting a reaction sequence from **21** through **26** and then **27** to finally yield heat stable (up to 230 °C) **28**. Furthermore, a proton uptake into **28** was shown in a heating experiment of **27** in D_2_O (Figure S130). The three thermal reaction products were named thermocyclenes A (**26**), B (**27**) and C (**28**).

The thermal reaction from **21** to intermediate **26** and further to **27** and **28** was also investigated computationally (Table S19, Figure S131). For the DA reaction an activation barrier of 28.1 kcal mol^−1^ and a free reaction energy of −18.7 kcal mol^−1^ was found. In comparison, for the DA reaction between malonic anhydride and cyclopentadiene a calculated reaction barrier of 16.8 kcal mol^−1^ (B3LYP/6–311 + G**) has been reported,^[^ ^49^ ^]^ explaining why the intramolecular reaction of **21** requires an elevated temperature, while the DA reaction between malonic anhydride and cyclopentadiene proceeds at room temperature. For the second step the computations revealed a remarkable mechanism: Using water as an acid, **26** was protonated at C1 and simultaneously deprotonated at Me18, resulting in the water stabilised highly unusual zwitterionic intermediate **X** with a cationic center at C11 and a carbanion at Me18, that is reminiscent of a C,C‐ylide described in a recent computational study.^[^ ^50^ ^]^ This high energy species (+67.1 kcal mol^−1^) rearranges in a barrierless reaction to **27** with a free reaction energy of −73.5 kcal mol^−1^. For the conversion of **27** to **28** another unusual water‐mediated concerted process through **Y‐TS** was realised computationally. Also this step exhibits a high activation barrier (+53.2 kcal mol^−1^) and is because of the formation of a higher substituted alkene exergonic (−4.5 kcal mol^−1^). The computed figures for the steps from **26** to **28** may represent an upper limit, as traces of acid e. g. from Si‐OH groups of the glass vessel may significantly lower the activation barriers. The computations also explain why **26** for whose formation the lowest reaction barrier was determined can be accumulated using a lower reaction temperature. Furthermore, the computations reveal why **26**, **27** and **28** are sequentially formed, and why not the more stable **28** is directly obtained from **26**: In the reaction of **26** with one water molecule water has a dual function and acts not only as an acid, but also as a base. The deprotonation can only happen at Me18, which is near the water molecule, but not with abstraction of H14 that is on the other side. In consequence, **26** is first formed and then isomerised to the more stable **27**.

Notably, although CjNdtS is with an amino acid sequence identity of 20% only distantly related to CjCsS, the biosynthetic mechanisms of both enzymes share similar steps, resulting in rearranged skeletons with a shifted Me18. The biosynthesis of **21** starts with GGPP ionisation by diphosphate abstraction to **N**, followed by a 1,11–10,14‐cyclisation to **O**. A sequence of two 1,2‐hydride shifts via **P** to **Q** and a 1,2‐methyl group migration leads to **R** whose deprotonation yields the neutral intermediate **S**. Its reprotonation at C3 results in **T** that may undergo subsequent deprotonation to **21**. Alternatively, **T** can react in two cyclisations to **U** and **V** as the precursor of **22** by deprotonation. With this, the CjNdtS cyclisation cascade to **21** and **22** is very similar to the CjCsS cascade towards **11**: From the structure of **22** a GGPP conformation with Me18 and Me19 oriented down can be inferred, because both Me groups also point down in this enzyme product. The orientation of Me20 was investigated through incubation of (*R*)‐ and (*S*)‐GLPP with CjNdtS, showing more efficient conversion of the *R* enantiomer and suggesting a GGPP fold with Me20 pointing up (Figure S132).

The cyclisation mechanism of CjNdtS is also similar to the cascade towards anaerol B (**24**), a product of the recently described L56V‐F75A variant of the peyssonnosol B (**25**) synthase AbPS2 (Box 2 of Scheme 3).^[^ ^51^ ^]^ CjNdtS and AbPS2 occur in the same branch of the phylogenetic tree (Figure 1), but are with 22% sequence identity only distantly related. For AbPS2 a sequence of two 1,2‐hydride shifts (**O**‐**P**‐**Q**) was experimentally and computationally confirmed.^[^ ^51^ ^]^ The biosynthesis of **21** by CjNdtS also proceeds with a 1,2‐hydride shift from **O** to **P**, as established by the conversion of (3–^13^C,2–^2^H)DMAPP and IPP with GGPPS and CjNdtS, resulting in a broad triplet signal in the ^13^C‐NMR for C3 (^1^ *J*_C,D_ = 18.9 Hz, Δ*δ* = –0.47 ppm, Figure S133). Much sharper signals were observed after thermal conversion into labelled **27** (Figure S134). The deprotonation‐reprotonation sequence **R**‐**S**‐**T** was investigated using (*R*)‐ and (*S*)‐(1–^13^C,1–^2^H)IPP, showing retainment of the 1‐*pro*‐*S* hydrogen of GGPP, but a complete loss of the 1‐*pro*‐*R* hydrogen in the deprotonation to **S** (Figure S135). The reprotonation to **T** was investigated by incubation of (3–^13^C)GGPP^[^ ^48^ ^]^ with CjNdtS in D_2_O, revealing deuterium uptake at C3 (^1^ *J*_C,D_ = 20.0 Hz, Δ*δ* = −0.46 ppm, Figure S136). These findings uncover a difference to the AbPS2 mechanism for which a partial retainment of the 1‐*pro*‐*R* hydrogen was observed, which was explained by a deprotonation‐reprotonation sequence with abstraction and reintroduction of the same proton.^[^ ^51^ ^]^ Conversion of DMAPP and (*E*)‐ or (*Z*)‐(4–^13^C,4–^2^H)IPP with GGPPS and CjNdtS, resulting in stereoselective labellings at C12, demonstrated a non‐selective deprotonation from **T** to **21** with a slight preference for removal of H12*_E_* at the bottom, suggesting the same base may act in the sequence **R**‐**S**‐**T** and the deprotonation to **21** (Figure S137).

During our studies another DTS from *Dinghuibacter silviterrae* DSM 100 059 was investigated (Table S1, Figure S1), showing 45% amino acid sequence identity to CjNdtS. This enzyme converted GGPP efficiently into japonene A (**22**) as the main product (Scheme 3, Figure S138), besides traces of japonene B (**23**) (Table S20, Figures S139–S145) and known anaerol B (**24**), identifying the newly characterised enzyme as *Dinghuibacter silviterrae* Japonene A Synthase (DsJS). The low yield of **23** (0.2 mg from a conversion of 100 mg GGPP with DsJS) did not allow for its full spectroscopic characterisation by NOESY, and therefore the relative configuration was tentatively assigned based on biosynthetic considerations. In small scale incubations as used in isotopic labelling experiments **24** was the main product, and its absolute configuration was determined through the stereoselective deuteration method (Figures S146 and S147). The same sign of optical rotation indicated that **24** from DsJS and from AbPS2, and also **22** from DsJS and from CjNdtS are identical, revealing that all three enzymes produce compounds from the same enantiomeric series. The structural relationship between **24** and **22**/**23** was further strengthened through treatment of **24** with SOCl_2_, causing *anti*‐elimination to **22** as the sole product (Figure S148). Also with the Burgess reagent known to promote *syn* eliminations^[^ ^52^ ^]^ only **22**, but not **23** was obtained, likely because of a high strain associated with the tetrasubstituted double bond semicyclic to a cyclobutane ring in **23**. This observation led to the idea to perform an acid (*p*‐TsOH) catalysed isomerisation of **23** to **22**, ultimatly correlating these two structures with each other and confirming the relative configuration of **23** (Figure S149).

Analogous labelling experiments as described above were performed to confirm the 1,2‐hydride shift from **O** to **P**, and the deprotonation‐reprotonation sequence **R**‐**S**‐**T** with complete loss of the 1‐*pro*‐*R* hydrogen and reprotonation at C3 in the biosynthesis of **22** and **24** by DsJS (Figures S150–S152). Furthermore, incubation experiments with both enantiomers revealed a preference of DsJS for (*R*)‐GLPP (Figure S153), suggesting a similar GGPP fold for this enzyme with Me20 pointing up.

**Scheme 4:**
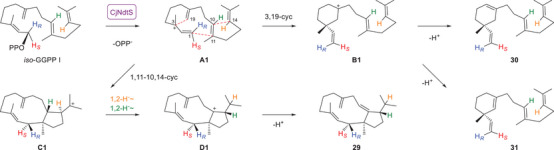
Enzymatic conversion of *iso*‐GGPP I with CjNdtS with cyclisation mechanism to **29** – **31**.

This finding contrasts our recently suggested cyclisation mechanism for **24** by AbPS2‐L56V‐F75A for which we assumed a GGPP fold with Me20 oriented down, because this Me group also points down in **24**.^[^ ^51^ ^]^ The alcohol **24** was only a minor product of AbPS2‐L56V‐F75A, rendering test incubations with (*R*)‐ and (*S*)‐GLPP inconclusive, but based on the results obtained here we must conclude that GGPP adopts likely the same conformation regarding Me20 orientation for the biosynthesis of all compounds **21** – **24**. This problem was further investigated using *iso*‐GGPP I,^[^ ^38^ ^]^ whose conversion with CjNdtS yielded three main products (Scheme 4, Figure S154), including not only the 1,11–10,14‐cyclisation product dolabella‐1,4(16),8‐triene (**29**) (Table S21, Figures S155–S162), but also two compounds arising through 3,19‐cyclisation. These were identified as taxasimplene (**30**), a compound previously obtained from *iso*‐GGPP I using taxa‐4,11‐diene synthase (TxS) from *Taxus brevifolia*,^[^ ^53^ ^]^ and japosimplene (**31**) (Table S22, Figures S163–170). The absolute configuration of **29** was determined through stereoselective labelling experiments (Figure S171). Notably, the absolute configurations of **30** from CjNdtS and from TxS are different based on different signs of optical rotation, which supports the shown stereochemical course for the 3,19‐cyclisation after ionisation of *iso*‐GGPP I to **A1**, with Me20 pointing up leading to (*R*)‐**30** (Scheme 4). According to the model, this reaction is expected to proceed with the 1‐*pro*‐*R* hydrogen of *iso*‐GGPP I ending up in the *Z* position of the vinyl group, and the 1‐*pro*‐*S* hydrogen in the *E* position. This was confirmed for **30** and **31** by converting (*R*)‐ and (*S*)‐(1–^13^C,1–^2^H)‐*iso*‐GGPP^[^ ^54^ ^]^ I with CjNdtS (Figure S172).

Taken together, the above described experimental results are in favour of a GGPP fold with Me18 and Me19 pointing down and Me20 oriented upward, which is the same fold as proposed for the biosynthesis of **25** in our previous study.^[^ ^51^ ^]^ Nevertheless, significant differences in the cyclisation cascades towards **22** – **24** versus **25** must be assumed, that were addressed by DFT calculations (Scheme 3, Table S23, Figure S173). Starting from **N**, the computations revealed a smooth conversion until **R** through similar steps as calculated before for **25**, only the conformations of the intermediates were slightly different. As a result of these differences **R** is obtained that can be deprotonated with abstraction of the 1‐*pro*‐*R* hydrogen to yield the *cisoid* diene **S** (ammonia was used for this purpose as a surrogate base in the DFT calculations). At this stage a conformational change to *transoid* **S** is required, that proceeds with an acceptable reaction barrier of 19.6 kcal mol^−1^ and brings Me20 from the front to the back. This allows for: 1. the reprotonation of **S** with setting of the correct stereochemistry at C3, 2. direct cyclisations to **U** and **V** with installation of *cis* ring junctions, and 3. deprotonation to **21** with a *cisoid* diene structure as required for the intramolecular DA reaction to **26**. In contrast, **25** shows a loss of the 1‐*pro*‐*R* hydrogen from C1 and its partial reintroduction with installation of a different configuration at C3, which was explained by the sequence **F“** to **H”** (Box 3 in Scheme 3).

## Conclusion

In this study, two sesquiterpene synthases (STSs) and three diterpene synthases (DTSs) with new function from bacteria of the Chitinophagaceae family were investigated. The identified STSs for *epi*‐isozizaene and isoishwarane are phylogenetically distant to previously characterised bacterial enzymes with same function.^[^ ^28^, ^30^ ^]^ Similarly, although phylogenetically distant, all three DTSs produced neodolabellane‐type diterpenes characterised by an initially formed 5,11‐bicyclic system with a shifted methyl group, with eventual further cyclisations to polycyclic skeletons. These results illustrate convergent evolution of TS function toward privileged structures in terpene biosynthesis, for which active site architectures may be main determinants, while overall amino acid sequences of TSs yielding the same product can be very different. Several of the compounds obtained in this study are typically known from marine organisms, including the red algal diterpenes presphaerol (**12**) and isosphaerodienes 1 (**13**) and 2 (**14**).^[^ ^31^, ^32^, ^35^ ^]^ Potentially, this could mean that the algal compounds may be formed by bacterial symbionts, however, recent research demonstrated that red algae encode functional type I TSs in their genomes.^[^ ^15^, ^16^ ^]^ As we disclosed here, all three compounds **12** – **14** require a structural revision and are actually not the direct enzyme products of the DTS CjCsS, but rather the highly reactive chitinosphaerol (**11**) is formed that turns into the three derivatives upon chromatographic purification. Future research on algal DTSs may reveal, whether **11** is also the true enzyme product in this case.

Two more enzymes, CjNdtS and DsJS, phylogenetically distant to CjCsS, but more closely related to each other and the recently characterised DTSs for peyssonnosol and peyssonnosol B,^[^ ^51^ ^]^ catalysed the formation of the main products neodolabella‐1(14),2,7‐triene (**21**) and japonene A (**22**), respectively, with partial overlap in their side products, which allowed to secure structural assignments through chemical correlations. Compound **21** is closely related to hydroxylated diterpenes from soft corals, reported as the first representatives of the neodolabellane skeleton,^[^ ^37^ ^]^ and shows a remarkable thermal reactivity through intramolecular DA reaction to **26**, followed by protonation induced skeletal rearrangement to **27** and isomerisation to **28**.

We have also investigated the cyclisation mechanisms of all three neodolabellane diterpene synthases in detail. All three enzymes start with a 1,11–10,14‐cyclisation, followed by two sequential 1,2‐hydride shifts and a 1,2‐methyl migration. The *E* configured 10,11‐double bond in the substrate GGPP causes a *trans* ring fusion in the initially formed bicyclic intermediate. As a consequence, and independent of the set C14 configuration, the subsequent 1,2‐hydride and methyl migrations necessarily lead to a *cis* orientation of the *i*Pr and the migrated Me group. These considerations further support our structural reassignments for **12** – **14** for which a *trans* substitution was assumed so far.

Another intriguing problem in the structure elucidation of the bicyclic neodolabellane diterpenes **11** and **21** was their special molecular mechanics that caused peak broadening in the NMR spectra. This is a well known phenomenon especially of terpenes with medium to large rings^[^ ^22^, ^41^, ^55^, ^56^ ^]^ or otherwise strained architectures.^[^ ^57^, ^58^ ^]^ As we demonstrate here, rigidification of the skeleton as realised by the NBS‐mediated conversion of **11** into bromosphaerols A and B can be a solution to overcome problems in structure elucidation and biosynthesis research.

The *epi*‐isozizaene synthase from *Streptomyces coelicolor* and isoishwarane synthase from *S. lincolnensis* are genetically clustered with a cytochrome P450 monooxygenase (CYP450) for downstream conversion into albaflavenone^[^ ^59^ ^]^ and an unidentified oxidation product,^[^ ^30^ ^]^ respectively. This is not the case for CjEIZS and CjIWS, and also for CjCsS and CjNdtS there are no hints for downstream oxidation pathways. Also none of the products from these four enzymes could be detected in heaspace extracts from *C. japonensis* obtained with a closed‐loop stripping apparatus (CLSA),^[^ ^60^ ^]^ suggesting that the coding genes may not be expressed under laboratory culture conditions. Only wanju‐2,5‐diene, the main product of CjWS, was observed in this analysis (Figure S174). Finally, none of the DsJS products were detected in the bouquet of *D. silviterrae*, demonstrating that phylogeny‐driven genome mining as performed in this study continues to be a promising method to uncover new terpene synthase functions.

## Conflict of Interests

The authors declare no conflict of interest.

## Supporting information



Supporting Information

## Data Availability

The data that support the findings of this study are available in the Supporting Information of this article.
