# Supplementary material for: Mechanistic Characterisation of Bacterial Terpene Synthases from Chitinophagaceae Producing Marine‐Type Diterpenes
Source: Angew Chem Int Ed Engl. 2025 Nov 13;64(52):e17373. doi: 10.1002/anie.202517373 (PMC12723471; doi:10.1002/anie.202517373)
Supplement: Supplementary file 1 — Supporting Information [file ANIE-64-e17373-s001.pdf]

Table of Contents

Type I TS homologs in *C. japonensis* DSM 13484 and D. silviterrae DSM 100059	3
GC/MS analysis of the products obtained from CjWS, CjEIZS and CjIWS	6
Phylogenetic analysis of CjEIZS and CjCsS	9
General Microbiological and Biochemical Methods	10
General Chemical and Spectroscopic Methods	12
Conversion of FPP with CjEISZ and CjIWS and compound isolation	13
Conversion of GGPP with CjCsS and product isolation (**12** – **14**)	14
GC/MS analysis of the products obtained from GGPP with CjCsS	15
Structure elucidation and NMR data of **12**	16
Structure elucidation and NMR data of **13**	24
Structure elucidation and NMR data of **14**	32
Detailed analysis of NOESY spectra for **12** – **14** (structural revision)	39
Incubation experiments with CjCsS and compound isolation (**11**)	42
Structure elucidation and NMR data of **11**	43
Conversion of **11** with N-bromosuccinimide	53
Structure elucidation and NMR data of **17**	54
Structure elucidation and NMR data of **18**	62
Conversion of *iso*-GGPP I with CjNdtS and product isolation (**19** and **20**)	70
Structure elucidation and NMR data of **19**	72
Structure elucidation and NMR data of **20**	80
Isotopic labelling experiments	88
The absolute configurations of **12**, **13** and **14**	89
The 1,2-hydride shift from **C** to **D** in the biosynthesis of **11**	95
The 1,2-hydride shift from **F** to **G** in the biosynthesis of **11**	96
The stereoselectivity of 1,2-hydride shift from **F** to **G** in the biosynthesis of **11**	97
The starting conformation of GGPP for CjCsS	98
Computational methods	99
Results of DFT calculations for CjCsS related reactions	100
GC/MS analysis of the products obtained from GGPP with CjNdtS	102
Conversion of GGPP with CjNdtS and product isolation (**21** and **22**)	103
Structure elucidation and NMR data of **21**	104
Structure elucidation and NMR data of **22**	112
Thermal reactions with **21**	120
Structure elucidation and NMR data of **27**	121
Chemical synthesis of (10,10,10-$^2H_6$)GPP	129
Experiments on the mechanism of the rearrangement from **26** to **27**	131
Structure elucidation and NMR data of **26**	134
Thermal reaction products of **21** obtained after prolonged heating	141
Structure elucidation and NMR data of **27**	142
Proton uptake in the isomerization of **27** to **28**	150

Results of DFT calculations for thermal reactions of **21** 151
The starting conformation of GGPP for CjNdtS 153
The 1,2-hydride shift from **O** to **P** in the biosynthesis of **21** 154
The deprotonation-reprotonation sequence **R**-**S**-**T** in the biosynthesis of **21** 156
The stereoselectivity of the deprotonation from **T** to **21** 158
GC/MS analysis of the products obtained from GGPP with DsJS 159
Incubation experiments with DsJS and compound isolation (**22** – **24**) 160
Structure elucidation and NMR data of **23** 161
The absolute configuration of **24** 168
Chemical correlation of **24** to **22** and **23** 170
The 1,2-hydride shift from **O** to **P** in the biosynthesis of **24** 171
The deprotonation-reprotonation sequence **R**-**S**-**T** in the biosynthesis of **24** 174
The starting conformation of GGPP for DsJS 176
GC/MS analysis of the products obtained from *iso*-GGPP I with CjNdtS 177
Conversion of *iso*-GGPP I with CjNdtS and product isolation (**29** – **31**) 178
Structure elucidation and NMR data of **29** 179
Structure elucidation and NMR data of **31** 187
The absolute configuration of **29** 195
The stereochemical course of the formation of **30** and **31** 196
Results of DFT calculations for the cyclisation from GGPP to **21** by CjNdtS 197
The volatiles released by *Chitinophaga japonensis* 199
Cartesian coordinates of computed structures for the CjCsS cyclisation cascade 200
Cartesian coordinates of computed structures for the thermal products of **21** 227
Cartesian coordinates of computed structures for the CjNdtS cyclisation cascade 241
References 265

Table S1. Type I terpene synthase homologs encoded in the genomes of *Chitinophaga japonensis* DSM 13484 and *Dinguibacter silviterrae* DSM 100059.

**accession no.**	**enzyme[a]**	**closest characterised homolog[b]**	**function**
WP_145709842	–	chryseodiene synthase (WP_073290622, *Chryseobacterium polytrichastri* DSM 26899, 41%)[1]	inactive[c]
WP_145709843	–	cembrene A synthase (WP_158688833, *Streptomyces mobaraensis* NBRC 13819, 33%)[2]	inactive[c]
WP_145711301	CjEIZS	wanju-2,5-diene synthase (WP_089795910, *Chryseobacterium wanjuense* DSM 17724, 25%)[1]	*epi*-isozizaene synthase
WP_145712228	CjCsS	wanju-2,5-diene synthase (WP_089795910, *Chryseobacterium wanjuense* DSM 17724, 26%)[1]	chitinosphaerol synthase
WP_145712912	–	γ-cadinene synthase (WP_012792334, *Chitinophaga pinensis* DSM 2588, 36%)[3]	no expression
WP_145715058	CjNdtS	peyssonnosol synthase (MCA9894015, *Anaerolineae bacterium* HKST-UBA68, 21%)[4]	neodolabella-1(14),2,7-triene synthase
WP_145715552	–	–	chitino-2,5(6),9(10)-triene synthase[5]
WP_145716435	CjIWS	(*Z*)-γ-bisabolene synthase (WP_035857999, *Cryptosporangium arvum* DSM 44712, 26%)[6]	isoishwarane synthase
WP_145716547	–	chitinol synthase (WP_012791584, *Chitinophaga pinensis* DSM 2588, 62%)[7]	n.d.[d]
WP_145716966	CjWS	wanju-2,5-diene synthase (WP_089795910, *Chryseobacterium wanjuense* DSM 17724, 63%)[1]	wanju-2,5-diene synthase
WP_145717101	–	γ-cadinene synthase (WP_012792334, *Chitinophaga pinensis* DSM 2588, 59%)[3]	no expression
WP_133998790	DsJS	peyssonnosol synthase (MCA9894015, *Anaerolineae bacterium* HKST-UBA68, 20%)[4]	japonene synthase

[a] Abbreviation used in this study. Enzymes named “Cj” are from *C. japonensis* and “Ds” are from *D. silviterrae*. [b] Closest characterised bacterial homolog published in the literature. The accession no., source organism, and amino acid sequence identities are given in brackets. If the enzyme itself has been published, this field is empty. [c] Inactive with substrates GPP, FPP, GGPP and GFPP. [d] n.d. = not determined.

WP_145709842 (*C. japonensis* DSM 13484, inactive)
MNAQMLSRFRYPFPTLKNPFAEALQELTEHEWIDKEYRWLYQDDEATRLKYKRTQTAHIASQWFPTAS
YERLQPVCRLMLWTLYN**DDIYE**EALPEEIRQVHTRTIAILKGEMTAGESRLPLGQLLAALRMELSAFL
TPGSLQRFVAALSSYFTGLEQEMSYKARKTFPGVEECLAI**R**ENSLCLRPFLELADMETRQPLPDEIHD
HPVMQRLMSLAIRMMVCF**NEVQSVIKD**EATDGIYYNVVKAIQHNRNLSLEEACLEDLHIHNEYLREFI
HLQSFLPDFGKWQDVVINRVHYISMTLSGWKSISFNLP**RY**NSTDGFPAVNVLKQQKYR

WP_145709843 (*C. japonensis* DSM 13484, inactive)
MNTPLNVPKPVYPWPVFQSPFVGAFNEEECNWYDEDYQFLSPEARARYKENRLADVGAFMVPTVTDRD
KVRAVARFVIYMTVT**DDYVE**LLPLKEIAAFRDRVFEVMMGDEPRPEEKGILRQMQTNRKEWIAFGMPD
SWIKRIAMNYKYRFLNDGTMEESPYKISKDVPPLPLFHLI**R**ANSIGMIPFIDQICTLTGFALPDYIYK
HTVIQRIVMLQAIIVALQ**NDFATIRKE**LSIESETFNIITLLQHHNKISFDEACTAGMRMHDEFVEEFV
TLADHLPDFSPYQKETEEFIFYIKQMISGLNSWYYNSGSK**RY**QVGGFPVPPNGKGETTHEIAVKHF

WP_145711301 (*C. japonensis* DSM 13484, *epi*-isozizaene synthase, CjEIZS)
MTIMTPAALPALQYPFDVQEGAHRNPYEQEIEAAISGWIDHEYLFLSEAQRAYYKKCRFGMLSSCFYP
EASYEALEVGARLVLLLFIH**DDYTD**KFSYDELQSYLLKSGSIMAGNHVPHDKGDMLHQFVLFRKALQP
LVPATWMQRWNSDLSYFYEGMLMERLFTAQQYPSIQQYIFL**R**EHLIGMHIYQDITELYMPSFMPHELL
FHPHVRELRQSAVRIIAWC**HDYYSVEKE**LDAGQIMNLVLVIQQERRCSLEDAFVEMAHVHDEEVNTFL
RLKEHAPDFGPYNELFREYAHHLQLMFKGNYLWHLESG**RY**NKF

WP_145712228 (*C. japonensis* DSM 13484, chitinosphaerol synthase, CjCsS)
MNQTLLTKFSPYYPRENRINPHVKILDTSTNAWIKGYHCLSEQVKQKVKNANYGILTAQIFPDTACNI
LLPIARWMLWAFVY**DDLYG**PFPKDELDLVCKKCLSILQGSRPLKGDNVFFRELADIRDELTPISTPEW
MERFTASHRHYFEGLMLDEFSYKQDISYPSSEQYLFI**R**ERLIGGNMVCDLLELVDGILPLELLLHPYI
QRLRQLAGRLMIYD**NDLFSYKKE**LQEKEAMNLVLILASERKISMDQAIQEVFEIRRSDFDELLSYEEA
IPEFGSYNNLVRNYLRNVLILLQGQLEWYLHQSL**RY**

WP_145712912 (*C. japonensis* DSM 13484, no expression)
MEQLRLPQIYCPFPASISRFVDEVNAHNIYWVQQFDLAPSPPLFAAYRKARFPWFVSRIYHTASYLEL
CIACDFCTWLFLV**DDILE**KTADDQAGHAQAVTEMLHVLQHNKVPLFSEYVNLAAALKDIWERLQAICP
PAWQRRFIGNMKTLFDATAWEARNRSLGQLPSIAEYISM**R**PFTSAMYPCIDLIEMVEQTWLPDEVLQQ
DLVQKLVLICIEAVCWV**NDLVSFEKE**QRENEPHNMVLLLREEHHLSPFDAIREVTEVCNESIRHFMFL
EKKLLSSAKGGQAYLQHYIMGLRSWIRGNLDWCIFDTE**RY**GLKLARQEEGGLVFSKLFLEE

WP_145715058 (*C. japonensis* DSM 13484, neodolabella-1(14),2,7-triene synthase, CjNdtS)
MSKELQHQTSAILEKVQQAYSTLTGAAPVFSLQELFNFEGFRLDEYCKVFSPHPQIHQLLADARAFGE
RFGIWLPNAEHYISCAIYLFPNAGVDRMKAIVRNLAIDYFL**NDTMG**RDIFPRLAPAEQEAASRVKERM
FNMAADLFIEEADATPVELANVKVLAFMRDTSPFSWFLEFLRLYSYHIYVAHKDCNINAAGRIASIDE
YIDS**R**CHLSGMHHVISLVEYSEGLFLDWEWLKETGIDKQLQRLHYTTAAIGGLM**NDLFSFEKE**VIDNQ
ADSNLVMIILHNKPRLSLEGAIRNAAAIVRSQLIEFVGLLDYIKGEVQQYTAVYPEKAAVMEAHFRGL
ERCVQASWIWQVYTK**RY**KSEYSIFRETRLDSMLVAG

Figure S1. Amino acid sequences and highly conserved motifs (highlighted in bold) of terpene synthase homologs from *C. japonensis*.

WP_145715552 (*C. japonensis* DSM 13484, chitino-2,5(6),9(10)-triene synthase)
MKTLPFPRISYPFTYGMNPHTAAVDVYTINWAVKYDLIENEAMLARYRQMQLNALVGRLYYSAGFQAL
RLLADYNFLFFIM**DDRCD**AVPRGQKKVYWEGNTARLTAVMDQHKRVFRGEGHPFEVAFSDAWERTQHA
ADAEWRSWFAATIREYFHACIWEAGNLDAGTPPTLEKYLAM**R**PYIAAAPFNVCAITYAENIRLPYEVY
RHPVVRQLMHLPARIVCWV**NDLFSLRKE**QQAGDMHNLVLVLQQERGLSMEAAMEAAACIHAEDMAQYL
RLEQQLPVLDKATGKELARYLTYLRAMISGNLEWSTVDTN**RY**YC

WP_145716435 (*C. japonensis* DSM 13484, isoishwarane synthase, CjIWS)
MIPFNVPNLPFDDLISPHIGELETKMNTWIQSLRDYPDSHKTKLRNMKLAHFSARLYPYADLPSLETI
SILALWIILN**DDVHD**GKTGRQLETMCRQSLQLLDAPPPADGHYAYSQLLSLFGQEVRKKQMSPRWMER
FKQHLFWYLKAMQWAADFDAAAKYPGEINFTQM**R**LKAGGVLMFTDLIELAIATELPLAVIRHPVIEHL
TEITNYILCVD**NDIFSLEKE**QLQRETMNLVLVLQNEHGCTIGEALNMAVERHNRYLAAYLELRETLPG
FGIWAAAVDKYVYGLETWMQGHLTWMQKDTE**RY**LVN

WP_145716547 (*C. japonensis* DSM 13484, putative chitinol synthase)
MKHTINIPKLYCPFPSRISPLVEKVDQHTFRWLHKFNLLQSDEMYEKFRRYKFAWMTARTYPYADYEF
LCIANNLYTWLFVL**DDQLD**NIKPETDSVRERPFLQKIIADFMNILRYDKVITPAEGGDILAALSDFWS
HILKMSSTSWQSQFMLSFKATFEAAVWEAVNAKRKQHPDVTQYMNL**R**TFFSGANLGTDMIEVASKVCL
PVYILQNEQFQKLVDLGRRVVCWA**NDLFSLSKE**LEHGDQHNLVVVLMNQHNMSLEEAIAEAAKIHNKE
VRQFIELKYKLPSFGSWKVDDDVRRYIDALEIMVRGFFDWSVYDTD**RY**HFTYAEDRKKKSLVRAA

WP_145716966 (*C. japonensis* DSM 13484, wanju-2,5-diene synthase, CjWS)
MSKNITDEQFYAGLQHLPKPHYPFPDFMHPDLQRQREEYYAWIDKEYTFHSKAAREKHKSHHLTDIAA
RGCPFLNGIEELRPLANFAANGAMM**DDYWD**RCSRDEMNEVTERITALLTGEDSREPSDNGIFHQFWIL
RQDAIKCGMPERLYKKYIAAIHDVLRGYAEERVYYKANVPPPLPVYLII**R**LDTSGGLPFCKYVAMQKN
YRDLPDSVLEHPFILRMHNLCAWMIGMH**NDCISLPKE**LHRDGDTMNIVKVMQHEHKLPLKEAYMAALE
LHDSYLQEFLLLKENLPPFGDLQDTASQYATDLGVMVAGVYAWHTNDKT**RY**VKGGYVEGEYVSQG

WP_145717101 (*C. japonensis* DSM 13484, putative γ-cadinene synthase, no expression)
MQTIQFPRMQYPFPSLMNRFANLANEQNLLWAQTFGLLKTEEALARFRKAKFALLVSRTFPNAGYEEL
CILCNFNSWLFLH**DDQCD**EAQMGRQATNLQQVTDYFTEILTREIAEPEQGGAFALALADIWQRLLPLS
RPAWRQRFIRSMQEYFKACLWEAGNRAEQRAPSVADYVIM**R**PYTGALFVDLEFVEISEKVHLPDQVLQ
HAMVQRLALACNNIVCWT**NDIMSCRKE**AGQGDVHNLVLALMRERSLSMQDAINETVRMHDEEVRLFAA
LEKLLPSFDAETDQELTRYVAVLRSWITGNYDWSVLDTG**RY**RVDPAPETHVQA

WP_133998790 (*D. silviterrae* DSM 100059, japonene synthase, DsJS)
MKVEASARLSLEQELDRARAQYHHVDRPAYSLQRLFDRPGITLSDFCRDFHPHPQSEVLRKWGRDFGE
QYGIWLPNAQHHVTCALYLYPEATTDRMVTMMKNLIVDFYL**NDLYG**RDQFGQCSPDEQYRGKQLIDHI
ATMGEALAPQLPTNPVEVANAATLEEFKKGSPAHWFAAFLRLYCHHIGVTYKDNNTATTGRLLSIDNY
IER**R**CHFAGMHHIVMWVEYCDGQFLDWHRLSQLGLSDKMRDLHWTIAAFGALS**NDLFSFEKE**VIDNGS
DSNLVMITLLNHPGLPLDEALLAAARIVSDLLARLFSLLQSIQAEIVQSSFPDPTMRDLLVRHLRGLE
RCMQASWLWQVFTQ**RY**KRPNSIWEETNLERV

Figure S1 (continued). Amino acid sequences and highly conserved motifs (highlighted in bold) of terpene synthase homologs from *C. japonensis* and *D. silviterrae*.

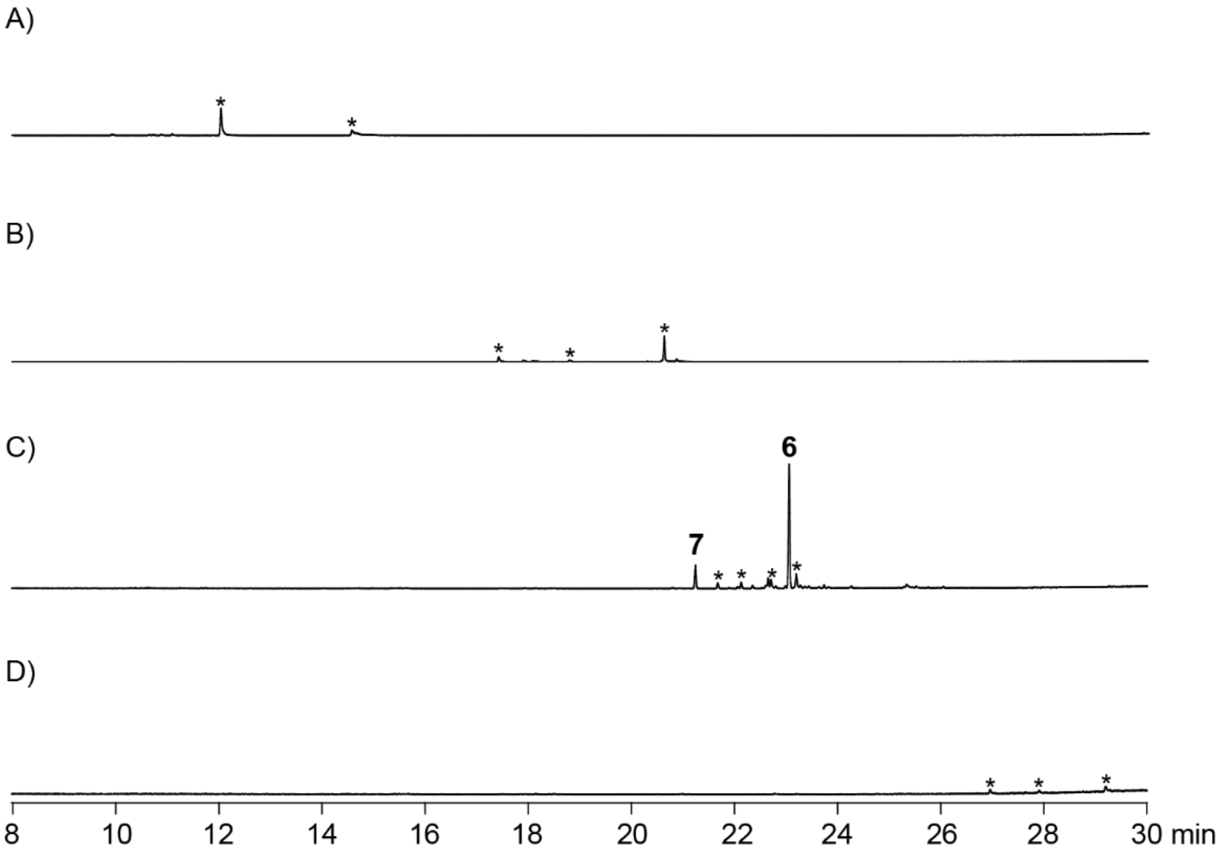

Figure S2. Total ion chromatograms of extracts obtained from incubations of A) GPP, B) FPP, C) GGPP, and D) GFPP with CjWS. Products identified from GGPP are wanju-2,5-diene (**6**) and polytrichastrene A (**7**). Asterisks indicate spontaneous hydrolysis products and contaminants such as plasticisers.

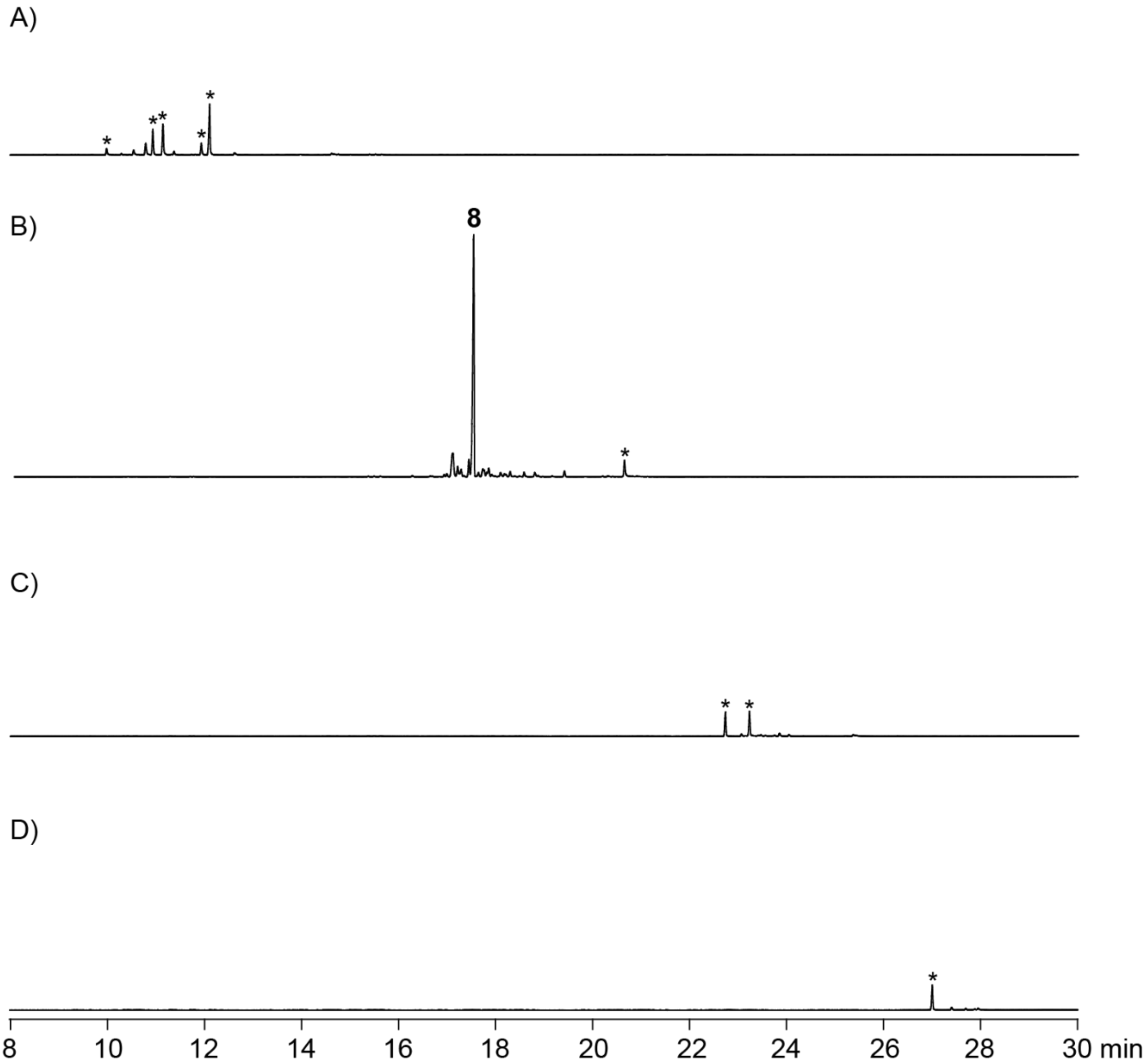

Figure S3. Total ion chromatograms of extracts obtained from incubations of A) GPP, B) FPP, C) GGPP, and D) GFPP with CjEIZS. The product identified from FPP is *epi*-isozizaene (**8**). Asterisks indicate spontaneous hydrolysis products and contaminants such as plasticisers.

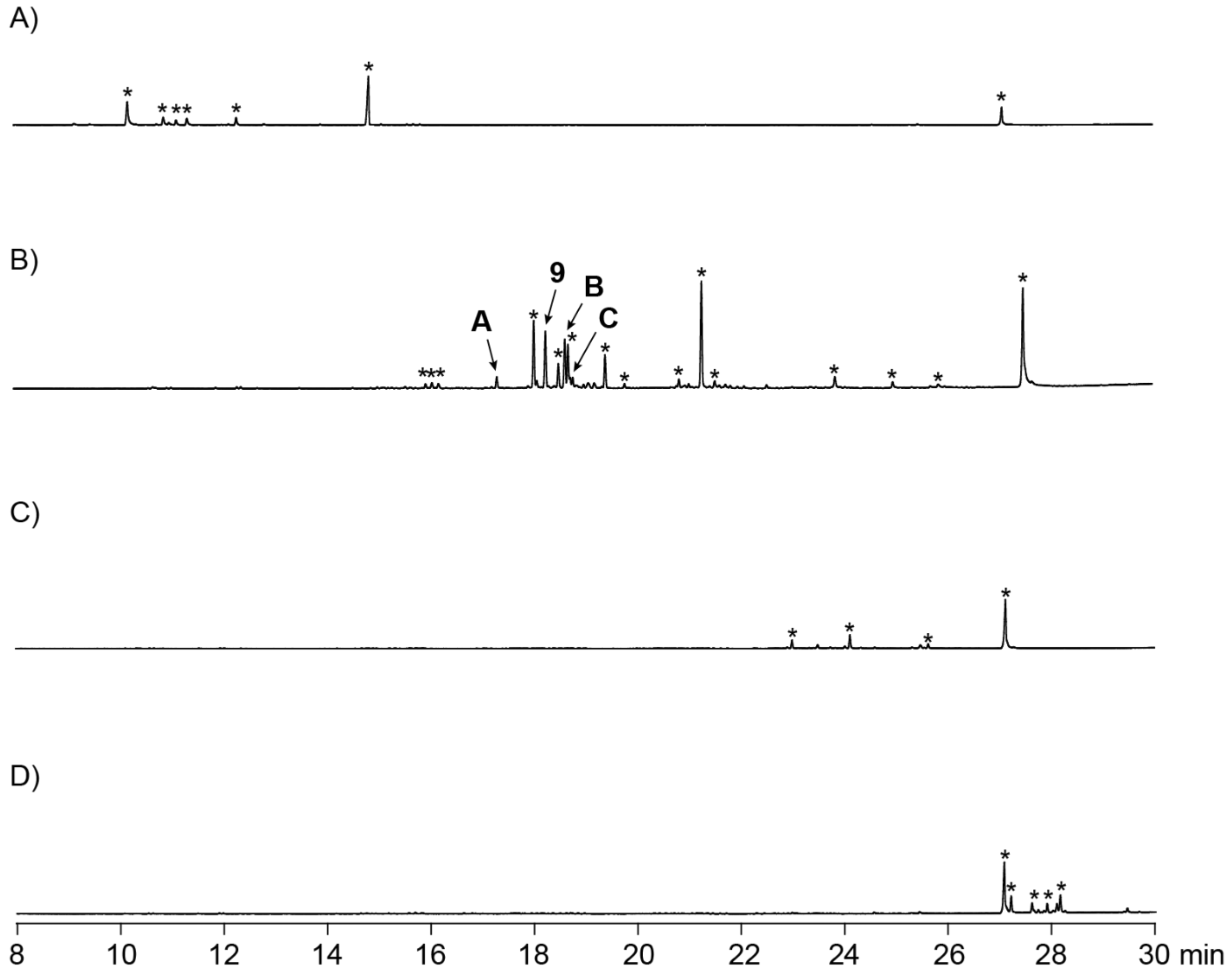

Figure S4. Total ion chromatograms of extracts obtained from incubations of A) GPP, B) FPP, C) GGPP, and D) GFPP with CjIWS. The products identified from FPP are isoishwarane (**9**), β-elemene (**A**, Cope rearrangement product of germacrene A), aristolochene (**B**) and valencene (**C**). Asterisks indicate spontaneous hydrolysis products and contaminants such as plasticisers.

Construction of phylogenetic tree

In our ongoing work, continuous BLAST searches using the amino acid sequences of various characterised terpene synthases as probes resulted in the discovery of the amino acid sequences of 5000 bacterial terpene synthase homologs. All these sequences are included in the phylogenetic tree of Figure 2 and the partial representation in Figure S5. Every included sequence was verified by individual inspection for the presence of the highly conserved motifs in type I terpene synthases. The tree was constructed using the tree builder function of Geneious (alignment type: global alignment with free end gaps, cost matrix: Blosum45, genetic distance model: Jukes-Cantor, tree build method: neighbor-joining, gap open panelty: 8, gap extension penalty: 2).

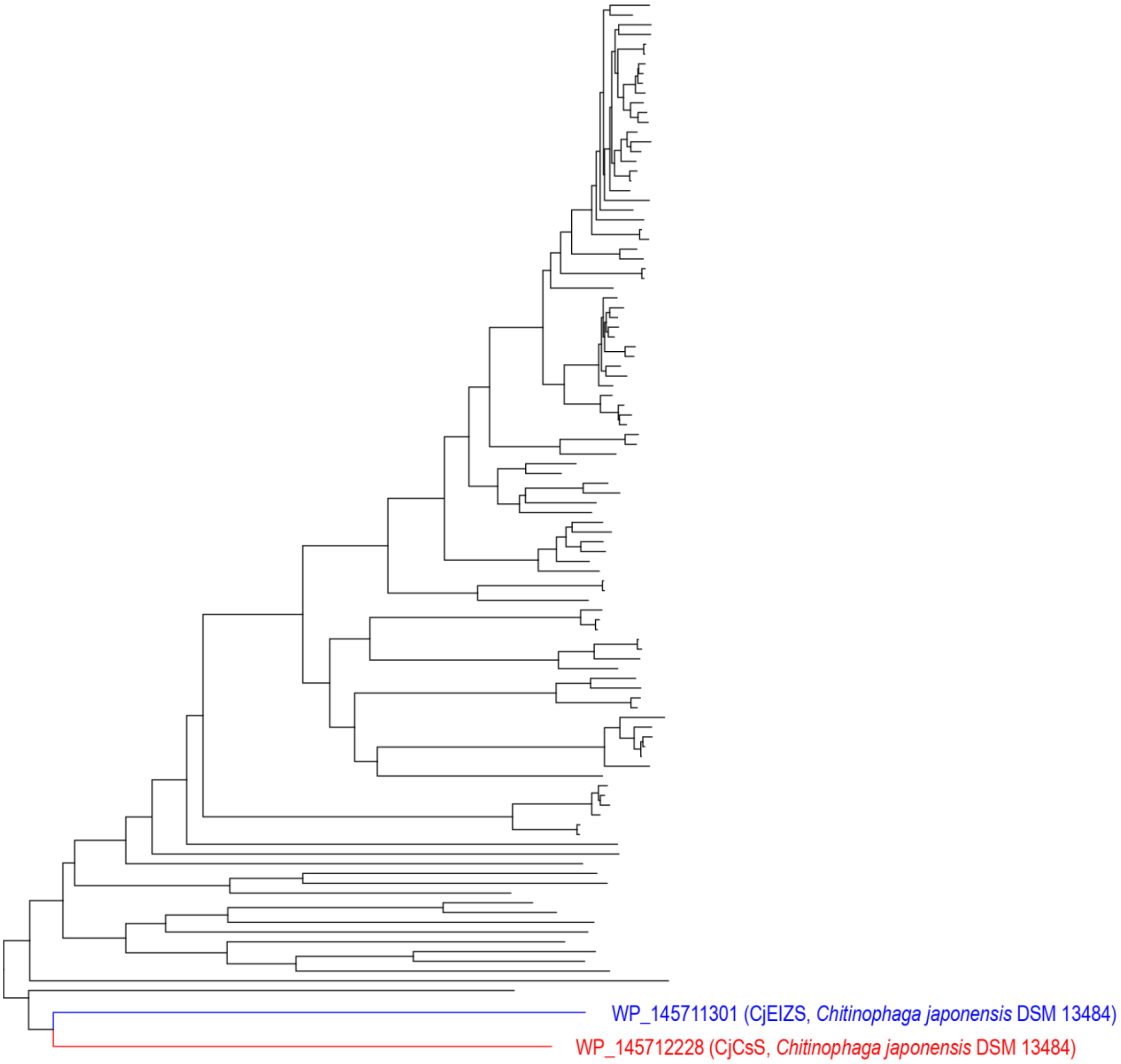

Figure S5. Detailed view on a branch of the phylogenetic tree constructed from 5000 bacterial TS homologs (Figure 2 of main text), showing CjEIZS and the closest related enzyme CjCsS from the same strain. These two enzymes have an amino acid sequence identity of 29%.

General Microbiological and Biochemical Methods

Strains and culture conditions

Chitinophaga japonensis DSM 13484 and *Dinghuibacter silviterrae* DSM 100059 were obtained from the Leibniz Institute DSMZ – German Collection of Microorganisms and Cell Cultures GmbH. *C. japonensis* DSM 13484 was cultivated in medium 1 (5.0 g peptone and 3.0 g meat extract dissolved in 1 L of deionised water, pH 7.2) with 0.5% NaCl at 28 °C. *D. silviterrae* DSM 100059 was grown in 25% strength of medium 830 (original recipe: 0.5 g yeast extract, 0.50 g proteose peptone, 0.5 g casamino acids, 0.50 g glucose, 0.50 g starch, 0.30 g sodium pyruvate, 0.30 g K_2HPO_4 and 0.05 g $MgSO_4$ x 7 H_2O dissolved in 1 L of deionised water, pH 7.2). For agar plate cultures agar (20.0 g L^{-1}) was added to the medium. *Saccharomyces cerevisiae* FY834 was grown on SM-URA agar (425 mg yeast nitrogen base, 1.25 g ammonium sulfate, 5 g glucose, 192.5 mg nutritional supplement minus uracil (Carl Roth GmbH, Karlsruhe, Germany), 5 g agar, 250 mL water).

Gene cloning

The target genes (Table S1) from C. *japonensis* DSM 13484 and *D. silviterrae* DSM 100059 were amplified from gDNA by PCR using Q5 High-fidelity DNA polymerase (New England Biolabs, Ipswich, MA, USA) and the primer pairs listed in Table S2, carrying arms homologous to the end sequences of the linearised (BamHI and HindIII digestion) pYE-Express shuttle vector.[8] Yeast homologous recombination of the PCR products with this linearised vector was carried out through the standard protocol using LiOAc, polyethylene glycol and salmon sperm DNA.[9] After transformation of *Saccharomyces cerevisiae* FY834 cultures were grown on SM-URA agar at 28 °C for 3 days. The recombinant plasmids were isolated from grown yeast colonies using the Zymoprep Yeast Plasmid Miniprep II kit (Zymo Research, Irvine, CA, USA) and subsequently used for transformation of *Escherichia coli* BL21 (DE3) electrocompetent cells. Cells were plated on LB agar plates amended with kanamycin sulfate (50 μg mL^{-1}) followed by incubation at 37 °C overnight. Single colonies were selected and used to inoculate LB medium (6 mL) liquid cultures with kanamycin sulfate (6 μL; 50 mg mL^{-1}). After 24 h growth plasmid DNA was isolated and checked for correct insertion of the desired gene by PCR amplification of the inserted DNA sequence using the T7 primer pair and by sequencing.

Table S2. Primers used in this study.

Primer	Nucleotide sequence (5'→3')[a]
WP_145709842-Fw	**GGCAGCCATATGGCTAGCATGACTGGTGGA**ATGAACGCACAAATGCTTTC
WP_145709842-Rv	**TCTCAGTGGTGGTGGTGGTGGTGCTCGAGTG**CTACCTATACTTTTGCTGCT
WP_145709843-Fw	**GGCAGCCATATGGCTAGCATGACTGGTGGA**ATGAACACGCCGTTAAATGT
WP_145709843-Rv	**TCTCAGTGGTGGTGGTGGTGGTGCTCGAGTG**CTAAAAATGTTTAACTGCTA
WP_145712912-Fw	**GGCAGCCATATGGCTAGCATGACTGGTGGA**ATGGAACAGTTACGCCTGCC
WP_145712912-Rv	**TCTCAGTGGTGGTGGTGGTGGTGCTCGAGTG**TCATTCCTCCAGGAACAGCT
WP_145715058-Fw	**GGCAGCCATATGGCTAGCATGACTGGTGGA**ATGTCCAAAGAACTGCAACA
WP_145715058-Rv	**TCTCAGTGGTGGTGGTGGTGGTGCTCGAGTG**CTATCCGGCAACCAGCATG
WP_145715552-Fw	**GGCAGCCATATGGCTAGCATGACTGGTGGA**ATGAAAACCCTTCCGTTTCC
WP_145715552-Rv	**TCTCAGTGGTGGTGGTGGTGGTGCTCGAGTG**TCAACAATAATAACGATTTG
WP_145716435-Fw	**GGCAGCCATATGGCTAGCATGACTGGTGGA**ATGATTCCTTTTAATGTGCC
WP_145716435-Rv	**TCTCAGTGGTGGTGGTGGTGGTGCTCGAGTG**CTAGTTCACCAGGTAGCGTT
WP_145717101-Fw	**GGCAGCCATATGGCTAGCATGACTGGTGGA**ATGCAAACGATCCAATTCCC
WP_145717101-Rv	**TCTCAGTGGTGGTGGTGGTGGTGCTCGAGTG**CTAAGCCTGGACATGTGTTT
WP_145716966-Fw	**GGCAGCCATATGGCTAGCATGACTGGTGGA**ATGTCAAAAAACATTAC
WP_145716966-Rv	**TCTCAGTGGTGGTGGTGGTGGTGCTCGAGTG**TTATCCCTGGCTAACAT
WP_145711301-Fw	**GGCAGCCATATGGCTAGCATGACTGGTGGA**ATGACCATTATGACACC
WP_145711301-Rv	**TCTCAGTGGTGGTGGTGGTGGTGCTCGAGTG**CTAAAATTTGTTATACCG
WP_145712228-Fw	**GGCAGCCATATGGCTAGCATGACTGGTGGA**ATGAACCAAACGCTGCTG
WP_145712228-Rv	**TCTCAGTGGTGGTGGTGGTGGTGCTCGAGTG**TTAGTACCTCAGGGATTG
WP_133998790-Fw	**GGCAGCCATATGGCTAGCATGACTGGTGGA**ATGAAAGTAGAAGCCTCC
WP_133998790-Rv	**TCTCAGTGGTGGTGGTGGTGGTGCTCGAGTG**TCACACCCTCTCCAGATT

[a] Sequences of homology arms for homologous recombination in yeast are indicated in bold, priming sequences for the target genes are indicated by underline.

Buffers

The buffers used for protein purification and incubation experiments were: binding buffer (20 mM Na_2HPO_4, 500 mM NaCl, 20 mM imidazole, 1 mM $MgCl_2$, pH = 7.4), washing buffer (20 mM Na_2HPO_4, 500 mM NaCl, 100 mM imidazole, 1 mM $MgCl_2$, pH = 7.4), elution buffer (20 mM Na_2HPO_4, 500 mM NaCl, 500 mM imidazole, 1 mM $MgCl_2$, pH = 7.4), incubation buffer (50 mM Tris/HCl, 10 mM $MgCl_2$, 10% glycerol, 20 mM β-cyclodextrin, pH = 8.2) and substrate buffer (25 mM aq. NH_4HCO_3).

Gene expression and protein purification

Small scale cultures of *Escherichia coli* BL21 (DE3) transformed with the corresponding recombinant pYE-Express vectors containing the cloned terpene synthase gene sequences were grown in LB medium (10 mL) amended with kanamycin sulfate overnight with shaking at 37 °C. Large scale expression cultures in LB medium (1 L) containing kanamycin sulfate were inoculated with the grown overnight culture (2 %). Culturing was continued with shaking at 37 °C until an OD_{600} = 0.4 – 0.6 was reached. After cooling the culture to 18 °C, enzyme expressions were induced by the addition of IPTG solution (100 mM, 1 ‰). The cultures were shaken at 18 °C for 18 h. Cells were harvested via centrifugation (1,500 g, 40 min, 4 °C), resuspended in binding buffer (20 mL, 4 °C) and lysed by ultrasonication (10x 1 min) on ice. The cell debris was removed by centrifugation (14,600 g, 10 min, 4 °C) and the supernatant was loaded on a Ni^{2+}-NTA superflow affinity chromatography column (Qiagen, Venlo, Netherlands) equilibrated with binding buffer. The column was washed with binding buffer (2 column volumes, CV, 4 °C) and washing buffer (2 CV, 4 °C). The desired proteins were eluted with elution buffer (1 CV, 4 °C). Protein purity was checked by SDS-PAGE analysis (Figure S6) and protein concentrations were determined through Bradford assay.[10]

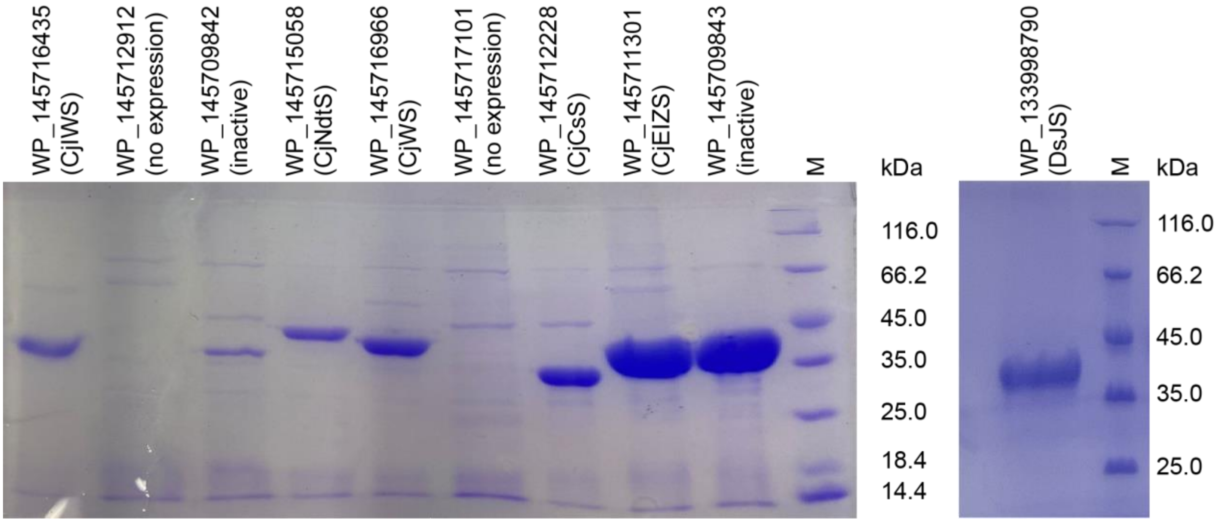

Figure S6. SDS-PAGE analysis of purified recombinant enzymes (listed in Table S1). M = marker.

Incubation experiments with recombinant proteins

Test enzymatic conversions of GPP, FPP, GGPP and GFPP were performed in incubation buffer (1 mL) and enzymes were added at a concentration of ca. 0.2 mg mL^{-1}, followed by incubation for 16 h at 30 °C. The reaction mixtures were extracted with n-hexane, the extracts were dried with $MgSO_4$ and analysed by GC/MS.

General Chemical and Spectroscopic Methods
Chemicals were purchased from Sigma Aldrich Chemie GmbH (Steinheim, Germany), Carbolution Chemicals GmbH (St. Ingbert, Germany), or Carl Roth (Karlsruhe, Germany) and used without purification. Solvents for column chromatography were purchased in p.a. grade and purified by distillation. Thin-layer chromatography (TLC) was performed with 0.2 mm precoated plastic sheets Polygram Sil G/UV254 purchased from Machery-Nagel (Düren, Germany). Column chromatography was performed using silica gel 60 purchased from Merck (Darmstadt, Germany).

GC/MS
GC/MS analyses were performed on a 5977A GC/MSD system (Agilent, Santa Clara, CA, USA) composed of a 7890B GC and a 5977A mass selective detector. The GC was equipped with a HP5-MS fused silica capillary column (30 m, 0.25 mm i. d., 0.50 μm film). Specific GC settings were 1) inlet pressure: 77.1 kPa, He at 23.3 mL min^{-1}, 2) injection volume: 1 μL, 3) temperature program: 5 min at 50 °C increasing at 10 °C min^{-1} to 320 °C, 4) 60 s valve time, and 5) carrier gas: He at 1.2 mL min^{-1}. MS settings were 1) source: 230 °C, 2) transfer line: 250 °C, 3) quadrupole: 150 °C and 4) electron energy: 70 eV. Retention indices (*I*) were determined from retention times in comparison to the retention times of *n*-alkanes (C_7-C_{40}).

HRMS
High resolution mass spectra were recorded on an Orbitrap XL instrument (APCI; Thermo Fisher Scientific, Waltham, MA, USA) or using a 7890B/7200 series gas chromatography/accurate mass Q-ToF detector system (Agilent). The GC was equipped with a HP5-MS fused silica capillary column (30 m, 0.25 mm i. d., 0.50 μm film). GC settings were 1) injection volume: 1 μL, 2) temperature program: 5 min at 50 °C, increasing 10 °C min^{-1} to 320 °C, 3) split ratio: 5:1, 60 s valve time and 4) carrier gas flow: He at 1 mL min^{-1}. MS settings were 1) inlet pressure: 83.2 kPa, He flow at 24.6 mL min^{-1}, 2) transfer line temperature: 250 °C, 3) ionization energy: 70 eV.

HPLC
Analytical scale HPLC separation was carried out using a EUROPA HPLC system (Knauer, Berlin, Germany), equipped with UV/Vis-DAD detector (190–600 nm) and a MACHEREY-NAGEL Nucleodur 100-3 C18 column (3.0 μm, 2.0 mm × 100 mm). The UV-Vis absorption was monitored at 190 – 600 nm. Preparative scale HPLC purification was performed on a TITAN HPLC system with a multi wavelength detector MWL 2.1L (190 – 700 nm) using a KNAUER Eurospher II 100-5 C18 column (5 μm, 8 x 250 mm).

NMR spectroscopy
NMR spectra were recorded on a Bruker (Billerica, MA, USA) Avance I (300 MHz), Avance I (400 MHz), Avance I (500 MHz), Avance III HD Prodigy (500 MHz) or an Avance III HD Cryo (700 MHz) NMR spectrometer. Spectra were measured in C_6D_6 and referenced against solvent signals (^{1}H-NMR, residual proton signal: δ = 7.16; ^{13}C-NMR: δ = 128.06).[11]

IR spectroscopy
IR spectra were recorded on a Bruker α infrared spectrometer with a diamond ATR probehead. Peak intensities are given as s (strong), m (medium), w (weak) and br (broad).

Optical rotations
Optical rotations were recorded on a Modular Compact Polarimeter MCP 100 (Anton Paar, Graz, Austria). The temperature setting was 25 °C; the wavelength of the light used was 589 nm (sodium D line); the path-length was 10 cm; the compound concentrations *c* are given in g 100 mL^{-1}.

Conversion of FPP with CjEIZS and CjIWS and product isolation
FPP (80 mg, 185 μmol) was dissolved in incubation buffer (135 mL), followed by the addition of an enzyme preparation of CjEIZS (15 mL, 5.4 mg mL^{-1}). The reaction mixture was incubated for 16 h at 30 °C, followed by the extraction with n-pentane (3x 150 mL). The combined extracts were dried with $MgSO_4$ and the solvent was evaporated. The crude product was purified via column chromatography on silica gel to yield pure **8** (9.9 mg, 49 μmol, 26%) as a colourless oil.
Analogously, a preparative scale enzymatic conversion of FPP (40 mg, 93 μmol) was conducted in incubation buffer (35 mL) with the addition of an enzyme preparation of CjIWS (15 mL, 3.7 mg mL^{-1}), followed by incubation for 16 h at 30 °C. The reaction mixture was extracted with n-pentane (3x 50 mL), the combined extracts were dried with $MgSO_4$ and the solvent was evaporated. The crude product was purified via column chromatography on silica gel to yield pure **9** (1.2 mg, 5.9 μmol, 6.3%) as a colourless oil.

(+)-*epi*-Isozizaene (8). Optical rotation: $[\alpha]_D^{25}$ = +10.0, *c* 0.2, CH_2Cl_2). NMR data were identical to literature data.[12]

(+)-Isoishwarane (9). Optical rotation: $[\alpha]_D^{25}$ = +31.7, *c* 0.1, CH_2Cl_2). NMR data were identical to literature data.[13]

Conversion of GGPP with CjCsS and product isolation
A preparative scale enzymatic conversion of GGPP (80 mg, 160 μmol) was conducted in incubation buffer (140 mL) with the addition of an enzyme preparation of CjCsS (20 mL, 3.6 mg mL^{-1}), followed by incubation for 16 h at 30 °C. The reaction mixture was extracted with n-pentane (3 x 100 mL), the combined extracts were dried with $MgSO_4$ and the solvent was evaporated. The crude product was purified via column chromatography on silica gel to yield pure **12** (0.5 mg, 1.7 μmol, 1.1%), **13** (0.8 mg, 2.9 μmol, 1.8%) and **14** (0.45 mg, 1.7 μmol, 1.1%).

Presphaerol (12). TLC (pentane/Et_2O = 2:1): R_f = 0.5. GC (HP-5MS): *I* = 2252. MS (EI, 70 eV): *m*/*z* (%) = 41 (13), 43 (21), 55 (8), 79 (13), 95 (11), 105 (6), 119 (6), 123 (3), 134 (1), 147 (2), 159 (2), 163 (2), 175 (2), 187 (2), 219 (4), 229 (2), 247 (1), 257 (1), 272 (2), 290 (1). IR (diamond ATR): ṽ = 3375 (br), 2951 (s), 2927 (s), 2869 (m), 1723 (w), 1674 (w), 1449 (w), 1377 (w), 1261 (w), 1167 (w), 1095 (w), 1019 (w), 878 (w), 800 (w) cm^{-1}. HR-MS (Q-TOF, 70 eV): calc. $[C_{20}H_{34}O]^+$ *m*/*z* = 290.2604; found: *m*/*z* = 290.2601. Optical rotation: $[\alpha]_D^{25}$ = +44.0 (*c* 0.05, acetone). NMR data are given in Table S3.

Isosphaerodiene 1 (13). TLC (pentane): R_f = 0.80. GC (HP-5MS): *I* = 2092. MS (EI, 70 eV): *m*/*z* (%) = 41 (10), 43 (8), 55 (5), 67 (4), 81 (5), 91 (9), 105 (8), 119 (13), 134 (8), 145 (2), 159 (2), 175 (7), 187 (4), 201 (1), 215 (1), 229 (4), 244 (1), 257 (2), 272 (6). IR (diamond ATR): ṽ = 2955 (s), 2925 (s), 2873 (m), 2188 (w), 1999 (w), 1735 (w), 1671 (w), 1440 (w), 1381 (w), 1258 (w), 1082 (w), 1020 (w), 845 (w) cm^{-1}. HR-MS (Q-TOF, 70 eV): calc. $[C_{20}H_{32}]^+$ *m*/*z* = 272.2499; found: *m*/*z* = 272.2509. Optical rotation: $[\alpha]_D^{25}$ = +95.0 (*c* 0.08, acetone). NMR data are given in Table S4.

Isosphaerodiene 2 (14). TLC (pentane): R_f = 0.75. GC (HP-5MS): *I* = 2062. MS (EI, 70 eV): *m*/*z* (%) = 41 (11), 43 (10), 55 (7), 67 (5), 79 (10), 91 (9), 95 (4), 105 (7), 119 (7), 134 (4), 147 (3), 159 (2), 163 (1), 175 (3), 187 (2), 201 (2), 229 (7), 244 (2), 257 (2), 272 (3). IR (diamond ATR): ṽ = 2955 (m), 2922 (s), 2868 (m), 2853 (m), 1731 (w), 1659 (w), 1632 (w), 1456 (w), 1376 (w), 1259 (w), 1091 (m), 1018 (m), 858 (w), 800 (w) cm^{-1}. HR-MS (Q-TOF, 70 eV): calc. $[C_{20}H_{32}]^+$ *m*/*z* = 272.2499; found: *m*/*z* = 272.2493. Optical rotation: $[\alpha]_D^{25}$ = +72.5 (*c* 0.04, CH_2Cl_2). NMR data are given in Table S5.

A)

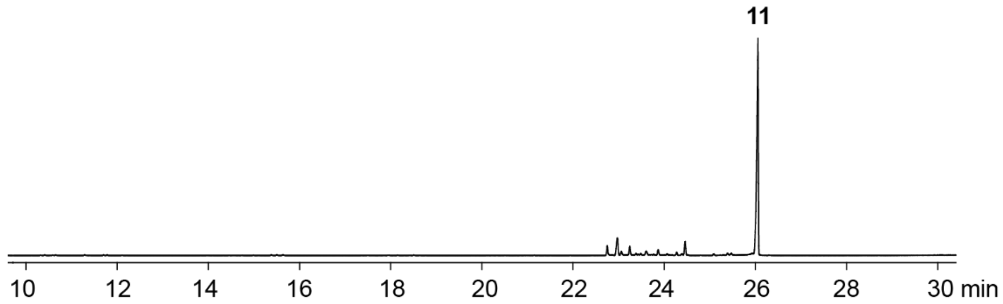

B)

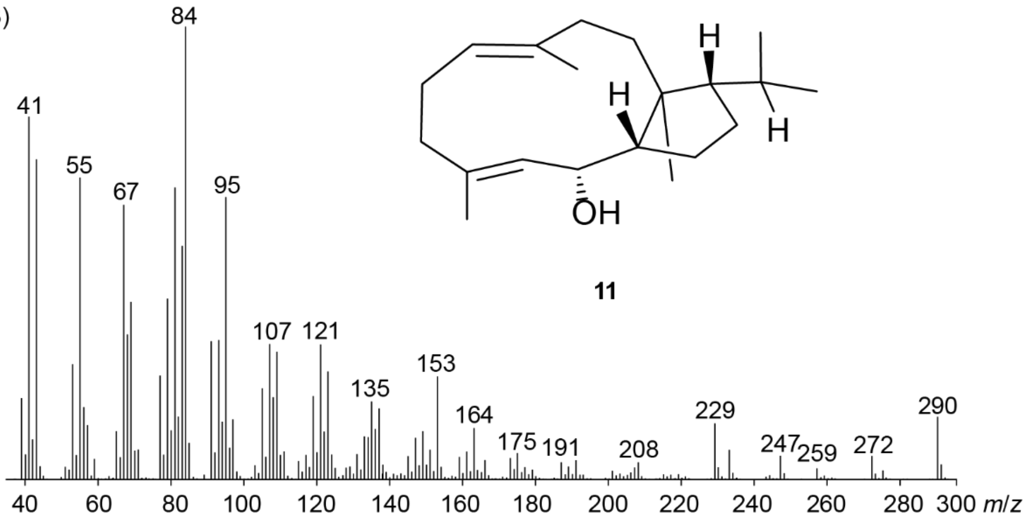

Figure S7. Enzymatic conversion of GGPP with CjCsS (GPP, FPP and GFPP were not accepted). A) Total ion chromatogram of an extract from the incubation of GGPP with CjCsS. B) EI mass spectrum of **11**. Asterisks indicate contaminants and spontaneous lysis and hydrolysis products of GGPP.

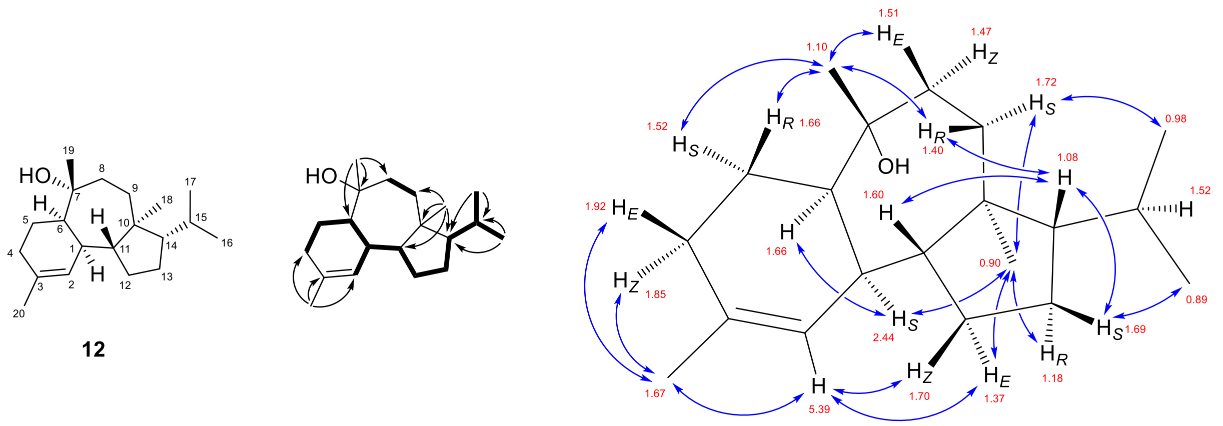

Figure S8. Structure elucidation of presphaerol (**12**). Bold: $^{1}H,^{1}H$-COSY, single headed arrows: key HMBC, and double headed arrows: NOESY correlations. Carbon numbering follows GGPP numbering to indicate the origin of each carbon. H_R, H_S, H_E and H_Z indicate the results of stereoselective labelling experiments (Figures S58 and S61).

Table S3. NMR data of presphaerol (**12**) in C_6D_6 recorded at 298 K.

C[a]	type	^{13}C[b]	^{1}H[b]
1	CH	36.13	2.44 (br s)
2	CH	126.84	5.39 (m)
3	C_q	132.47	–
4	CH_2	30.83	1.92 (m)
			1.85 (m)
5	CH_2	22.56	1.66 (m)
			1.52 (m)
6	CH	48.25	1.66 (m)
7	C_q	74.48	–
8	CH_2	37.81	1.51 (m)
			1.47 (m)
9	CH_2	37.23	1.72 (m)
			1.40 (m)
10	C_q	45.36	–
11	CH	50.50	1.60 (m)
12	CH_2	28.22	1.70 (m)
			1.37 (m)
13	CH_2	27.24	1.69 (m)
			1.18 (m)
14	CH	59.50	1.08 (m)
15	CH	30.99	1.52 (m)
16	CH_3	23.00	0.89 (d, J = 6.7)
17	CH_3	23.70	0.98 (d, J = 6.7)
18	CH_3	15.16	0.90 (s)
19	CH_3	23.82	1.67 (br s)
20	CH_3	29.72	1.10 (s)

[a] Carbon numbering as shown in Figure S8 indicates the origin of each carbon from GGPP by same number. [b] Chemical shifts δ in ppm, multiplicity: s = singlet, d = doublet, m = multiplet, br = broad, coupling constants J are given in Hertz.

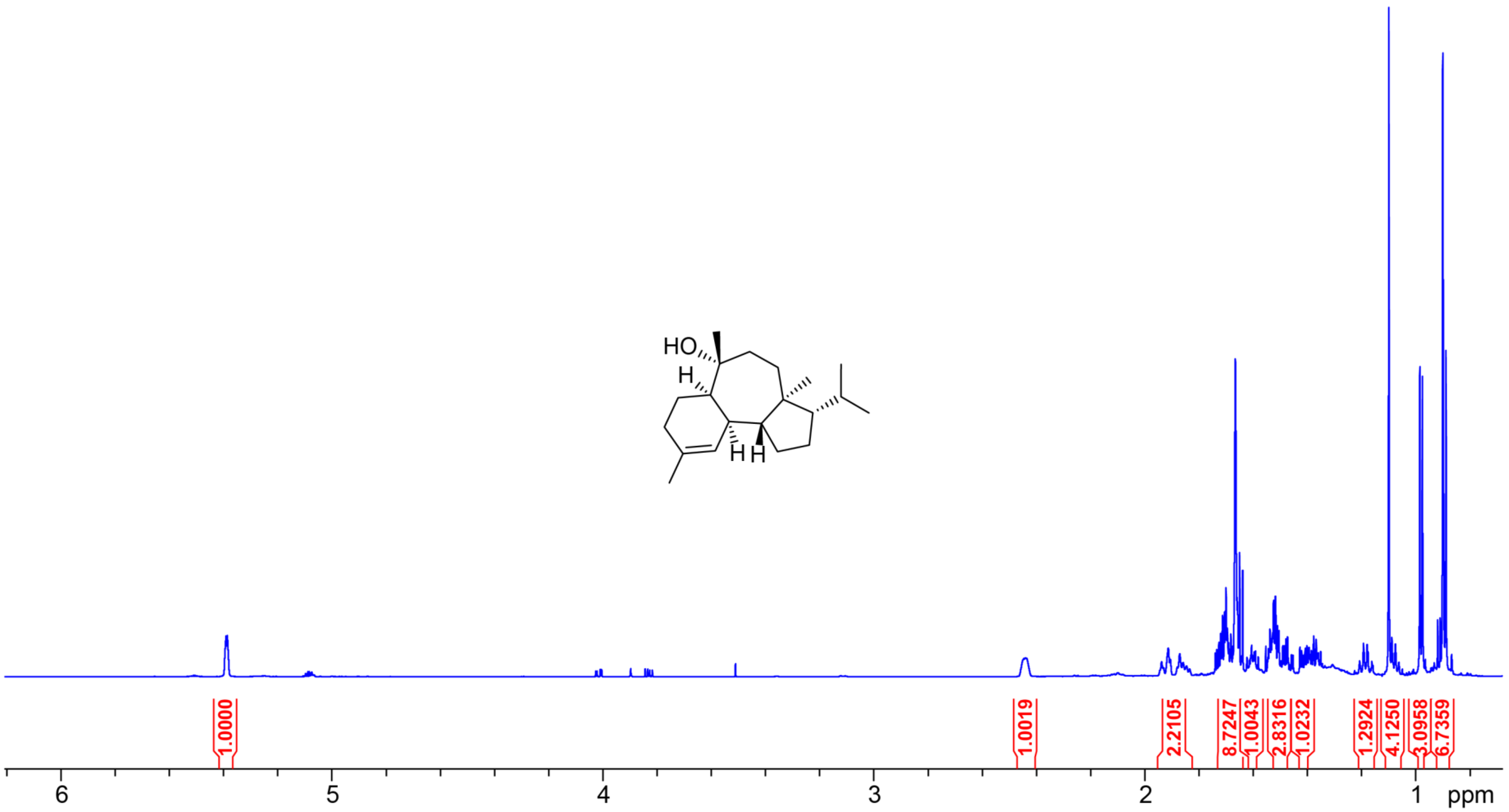

Figure S9. ^{1}H-NMR spectrum of **12** (700 MHz, C_6D_6).

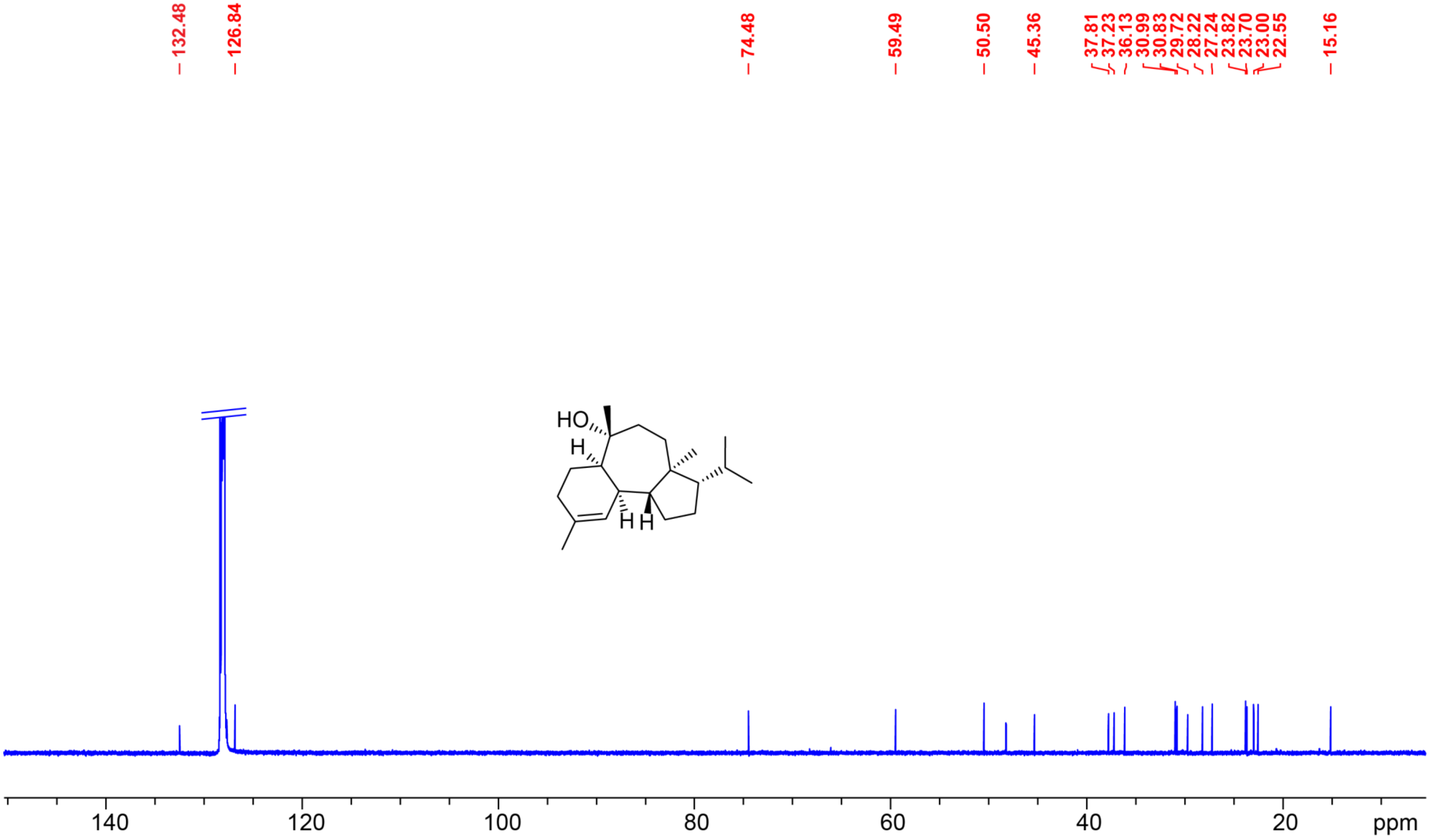

Figure S10. ^{13}C-NMR spectrum of **12** (176 MHz, C_6D_6).

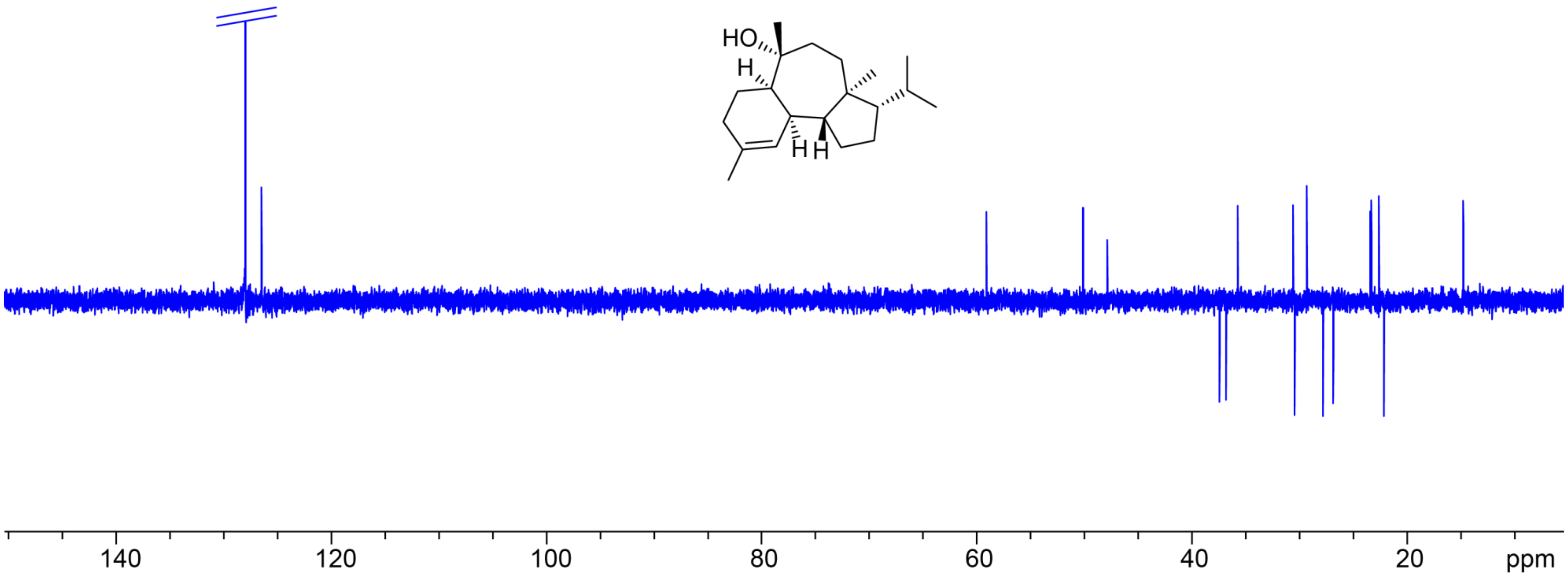

Figure S11. ^{13}C-DEPT135 spectrum of **12** (176 MHz, C_6D_6).

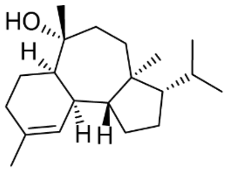

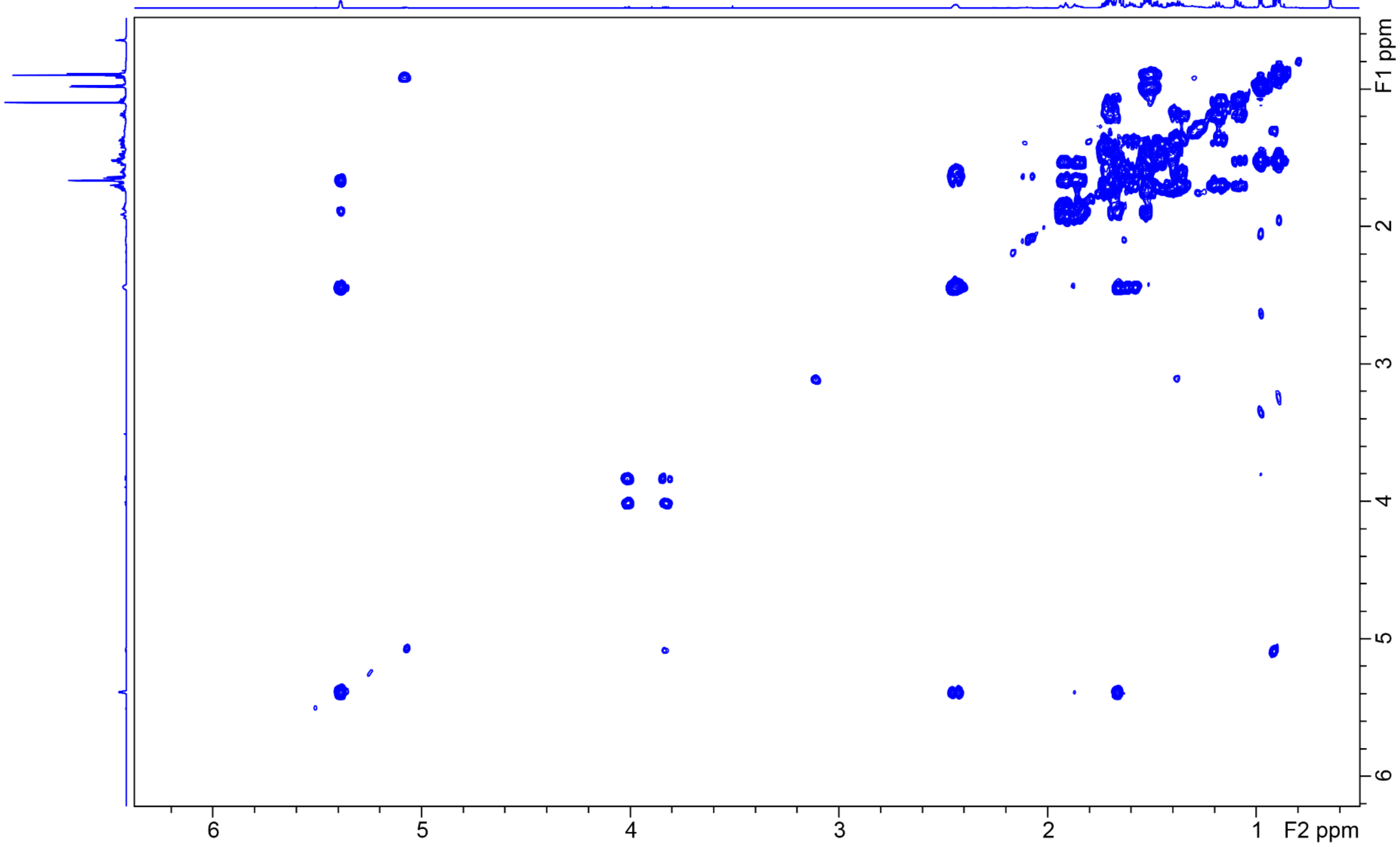

Figure S12. ^{1}H-^{1}H-COSY spectrum (C_6D_6) of **12**.

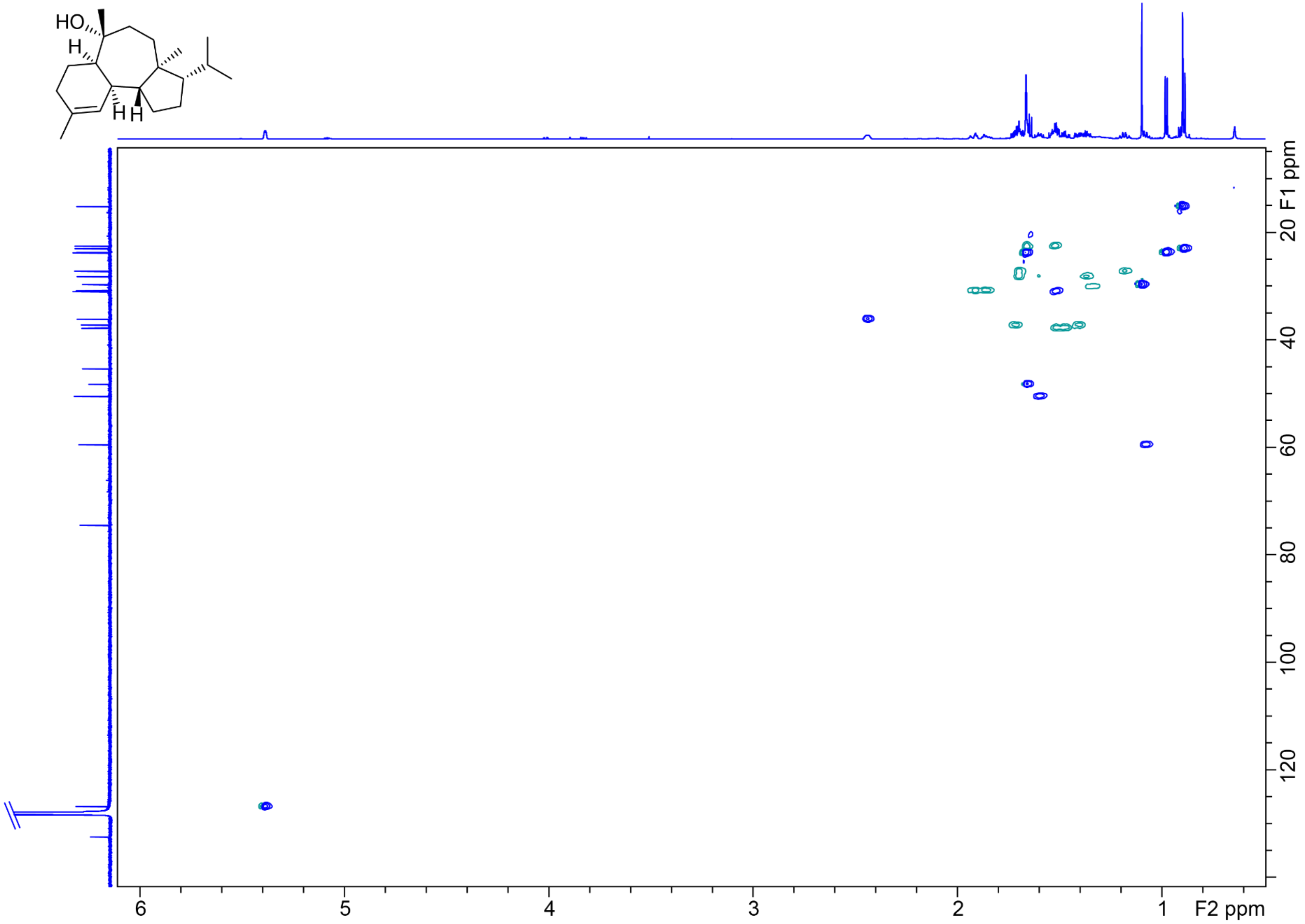

Figure S13. HSQC spectrum (C_6D_6) of **12**.

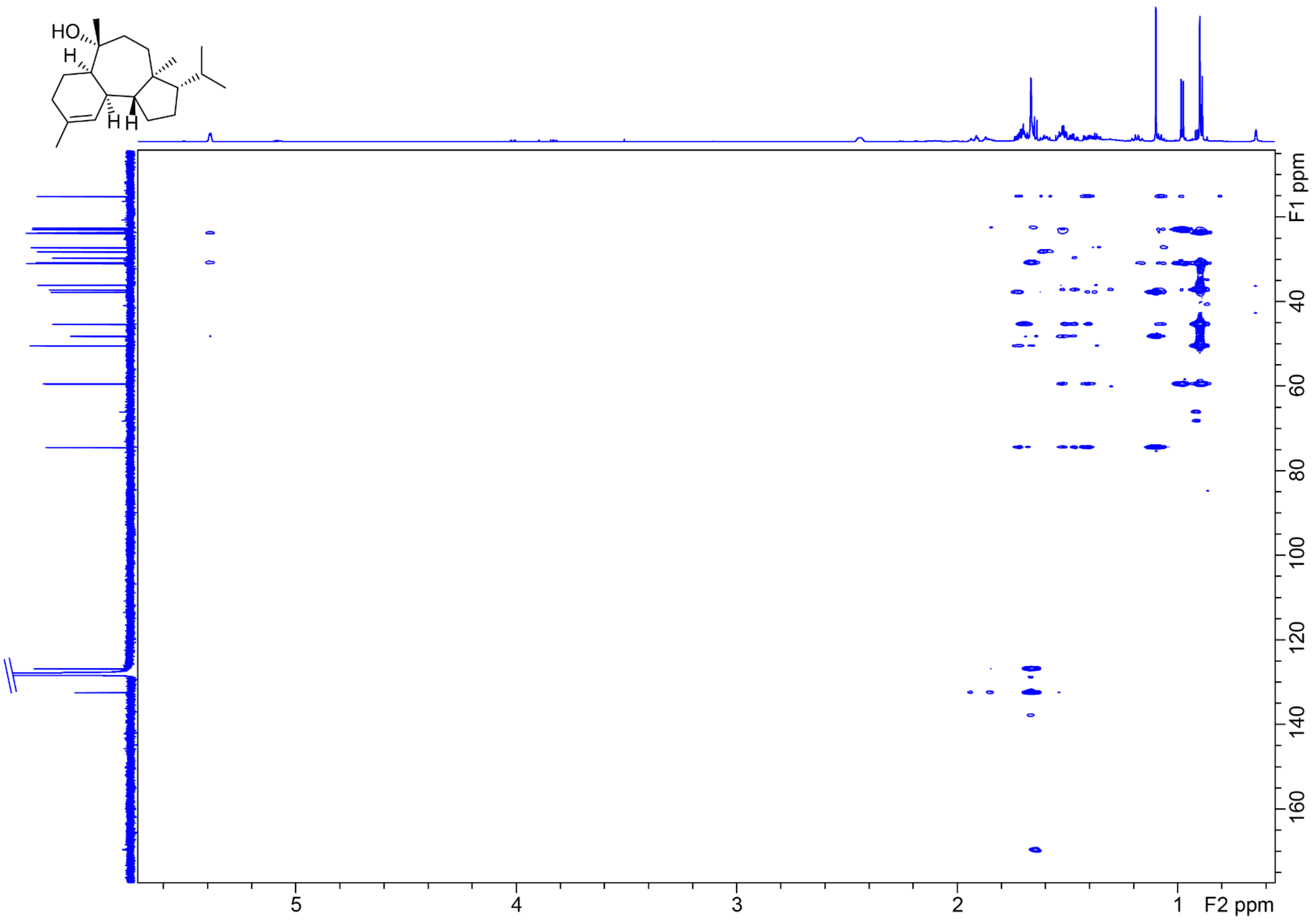

Figure S14. HMBC spectrum (C_6D_6) of **12**.

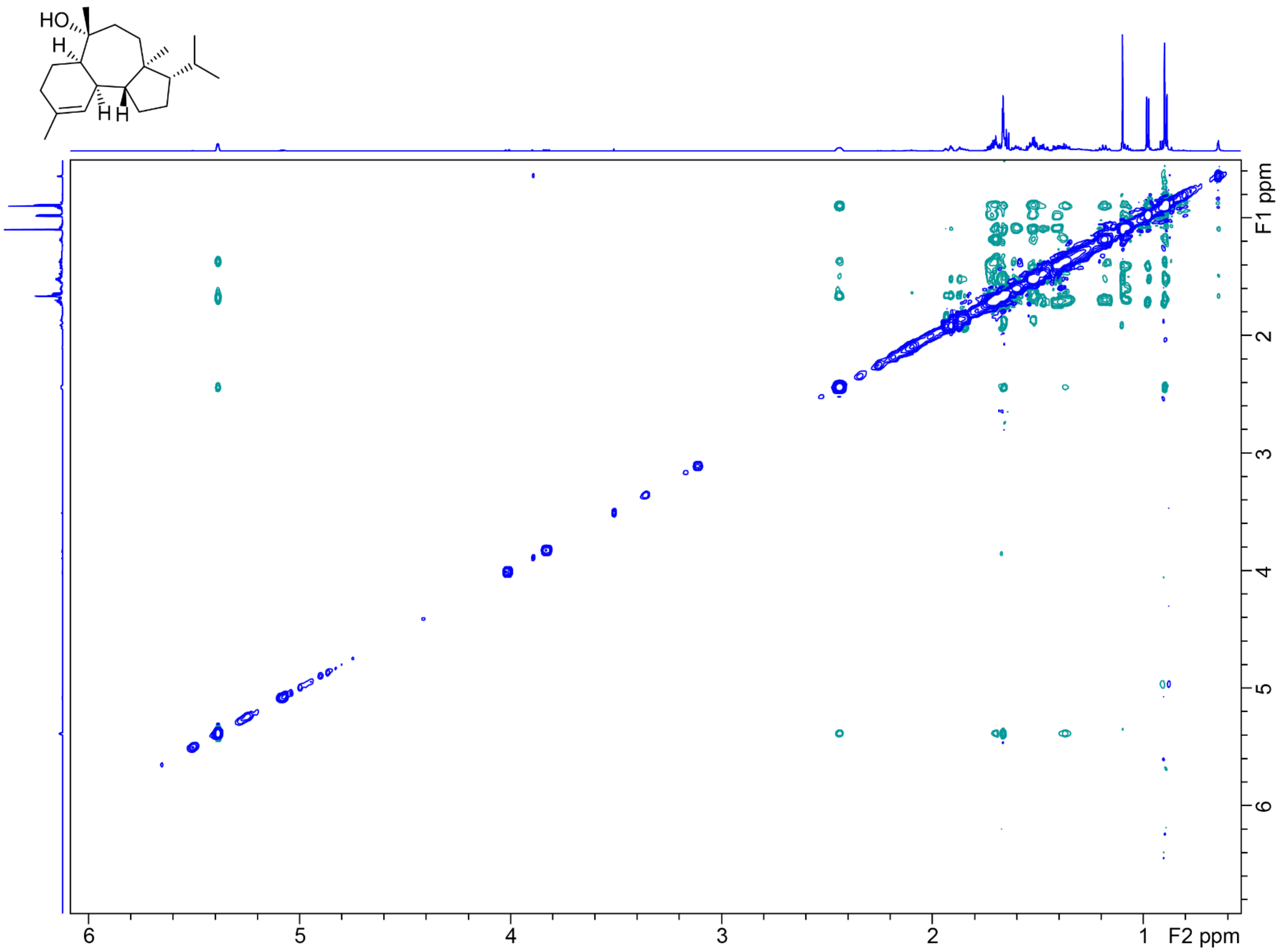

Figure S15. NOESY spectrum (C_6D_6) of **12**.

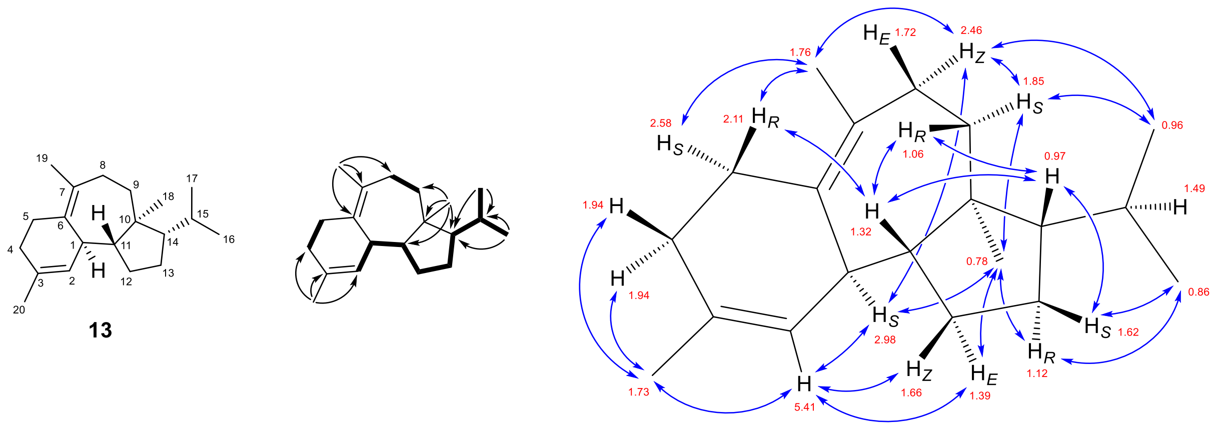

Figure S16. Structure elucidation of isosphaerodiene 1 (**13**). Bold: ^{1}H,^{1}H-COSY, single headed arrows: key HMBC, and double headed arrows: NOESY correlations. Carbon numbering follows GGPP numbering to indicate the origin of each carbon. H_R, H_S, H_E and H_Z indicate the results of stereoselective labelling experiments (Figures S59 and S62).

Table S4. NMR data of isosphaerodiene 1 (**13**) in C_6D_6 recorded at 298 K.

C[a]	type	^{13}C[b]	^{1}H[b]
1	CH	37.36	2.98 (br s)
2	CH	124.71	5.41 (br s)
3	C_q	134.00	–
4	CH_2	30.23	1.94 (m, 2H)
5	CH_2	26.00	2.58 (ddd, J = 14.1, 4.8, 3.8)
			2.11 (m)
6	C_q	135.87	–
7	C_q	129.08	–
8	CH_2	30.29	2.46 (dddd, J = 14.3, 12.8, 1.1, 1.1)
			1.72 (m)
9	CH_2	40.04	1.85 (ddd, J = 13.2, 7.4, 1.1)
			1.06 (br dd, J = 12.8, 12.8)
10	C_q	46.42	–
11	CH	53.10	1.32 (ddd, J = 13.3, 8.5, 6.8)
12	CH_2	26.04	1.66 (m)
			1.39 (m)
13	CH_2	26.51	1.62 (m)
			1.12 (m)
14	CH	58.79	0.97 (dd, J = 9.2, 9.2)
15	CH	30.94	1.49 (dhept, J = 9.0, 6.6)
16	CH_3	23.30	0.86 (d, J = 6.6)
17	CH_3	23.59	0.96 (d, J = 6.6)
18	CH_3	13.07	0.78 (s)
19	CH_3	24.14	1.73 (br s)
20	CH_3	20.77	1.76 (dd, J = 1.3, 1.3)

[a] Carbon numbering as shown in Figure S16 indicates the origin of each carbon from GGPP by same number. [b] Chemical shifts δ in ppm, multiplicity: s = singlet, d = doublet, hept = heptet, m = multiplet, br = broad, coupling constants J are given in Hertz.

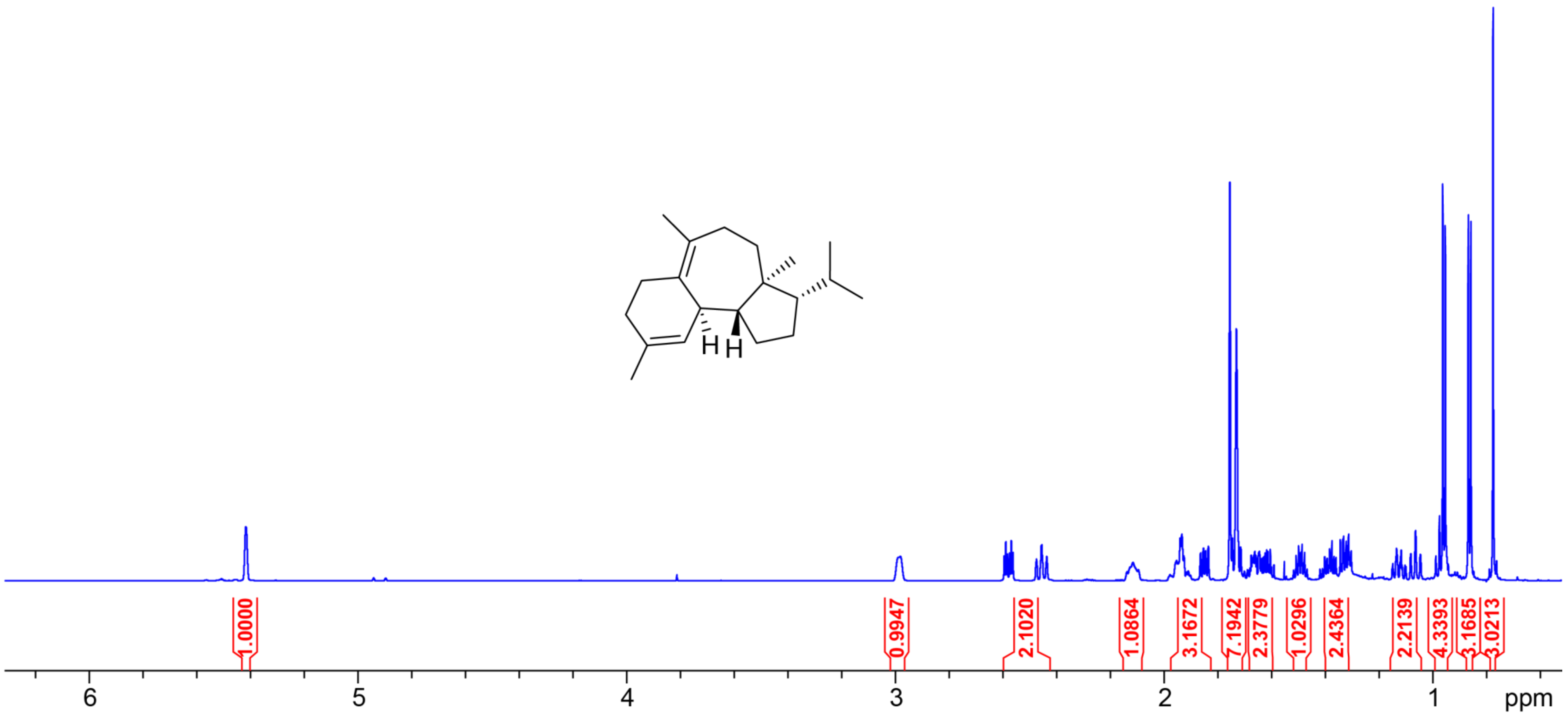

Figure S17. ^{1}H-NMR spectrum of **13** (700 MHz, C_6D_6).

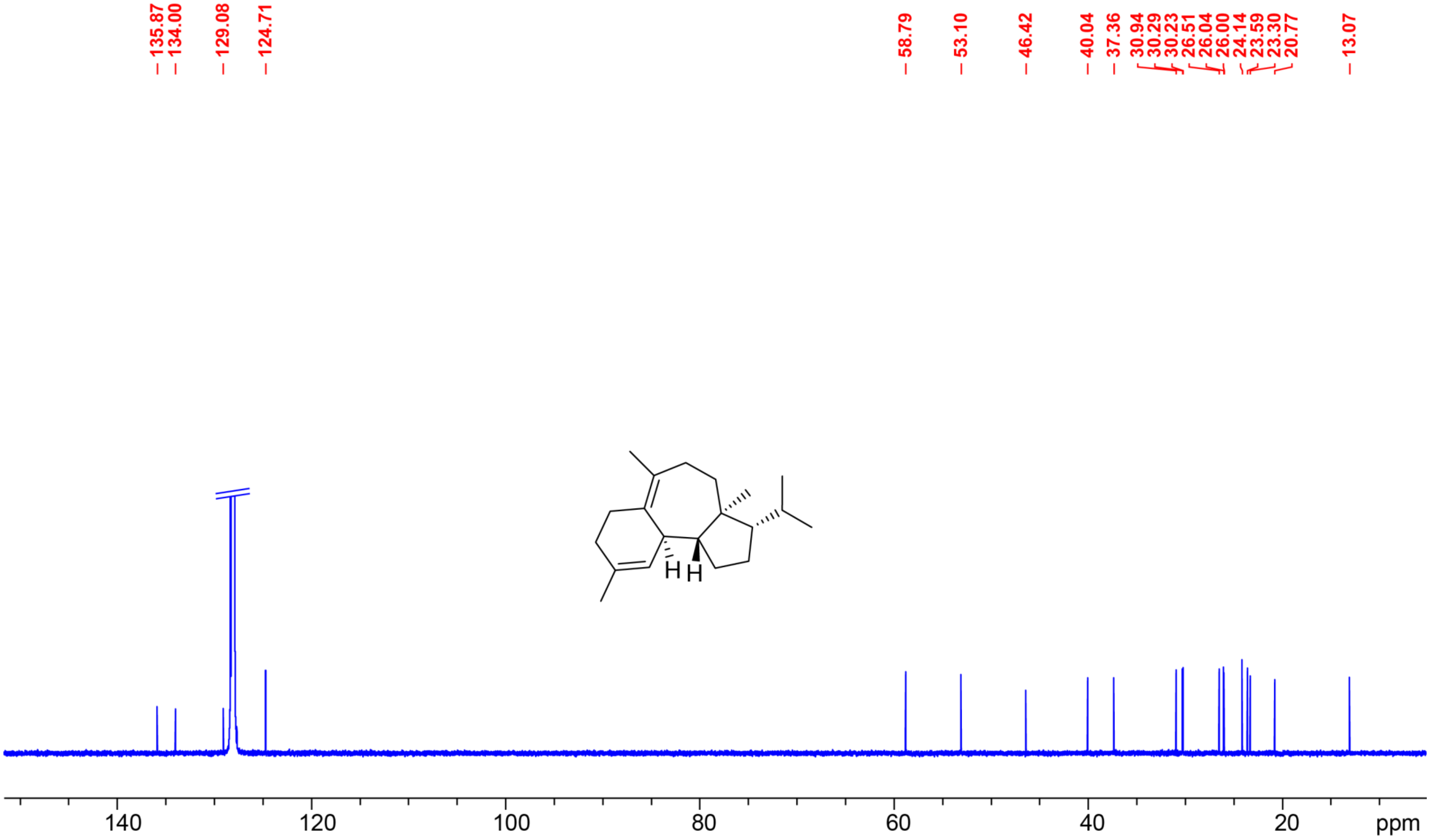

Figure S18. ^{13}C-NMR spectrum of **13** (176 MHz, C_6D_6).

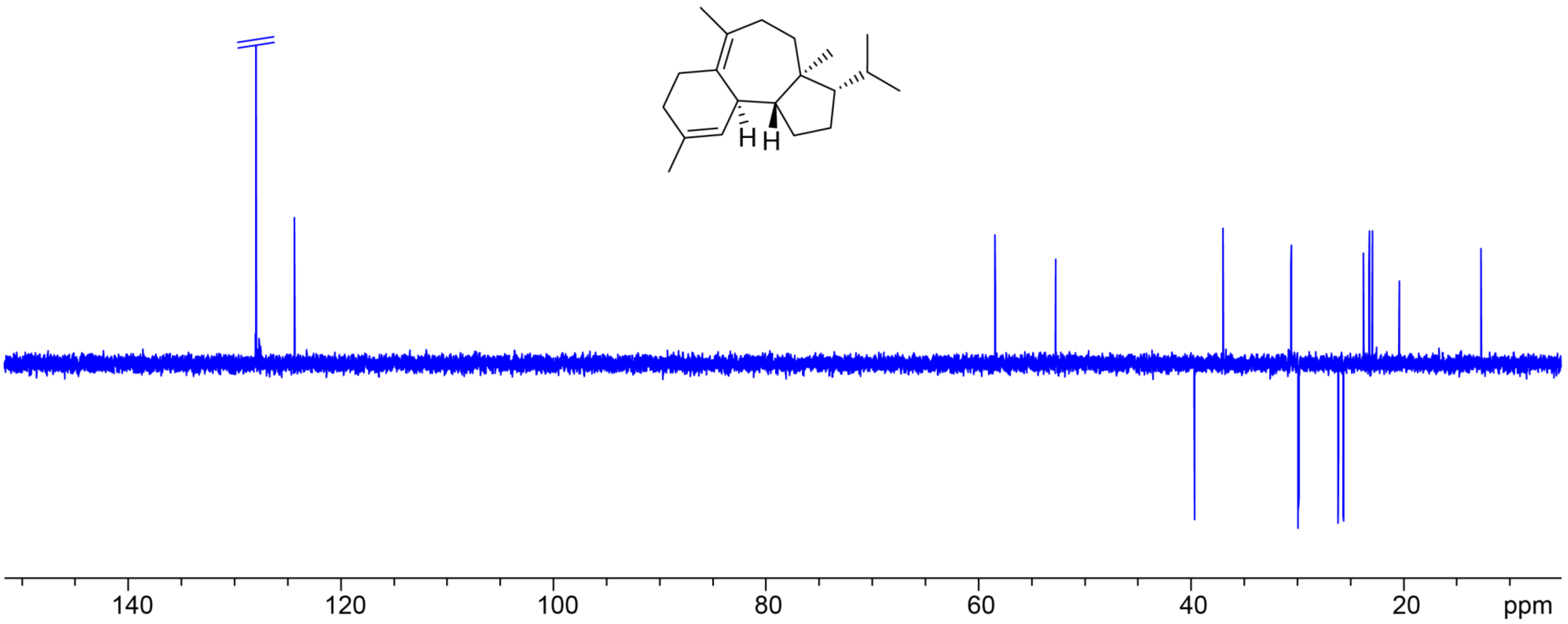

Figure S19. ^{13}C-DEPT135 spectrum of **13** (176 MHz, C_6D_6).

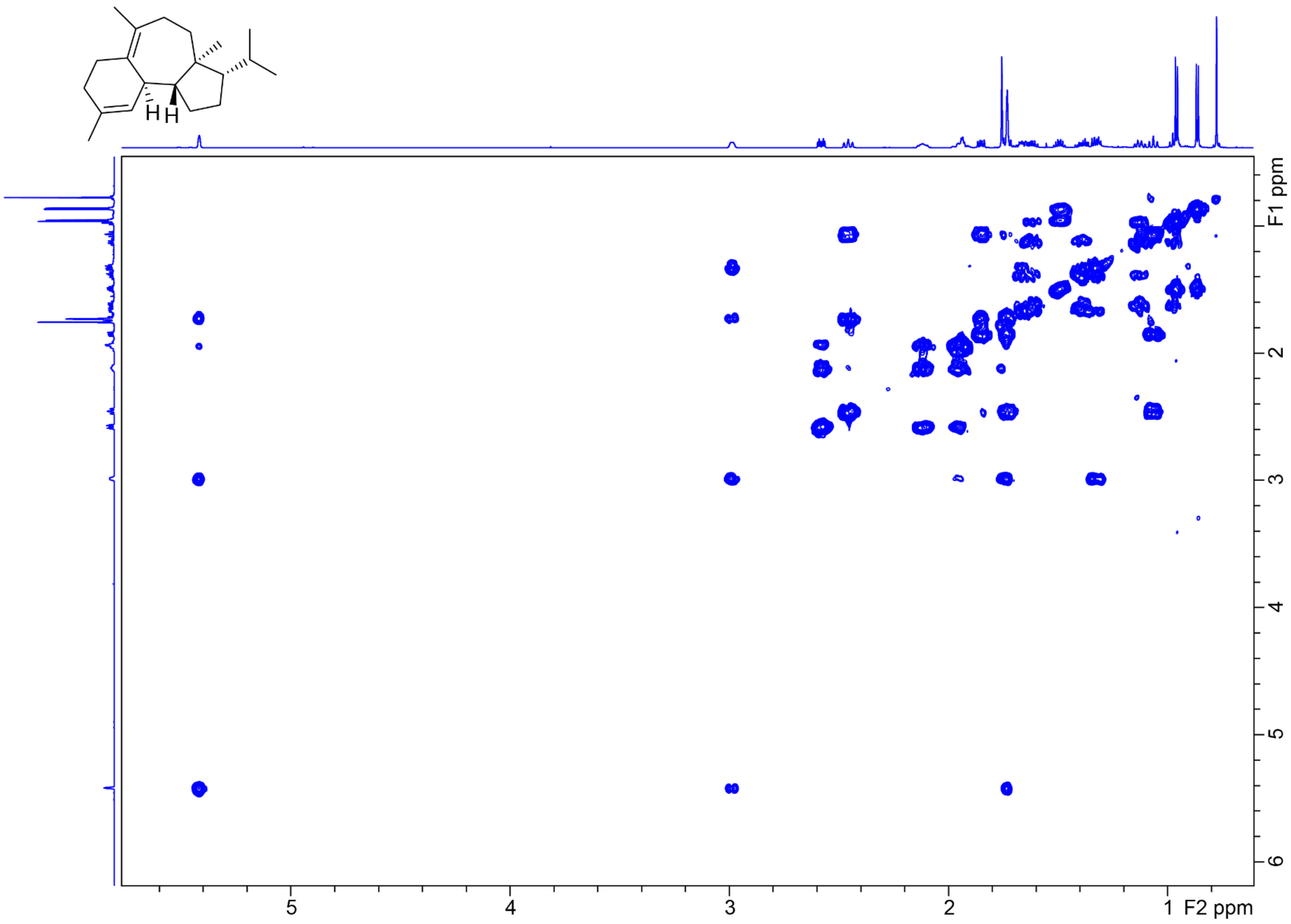

Figure S20. ^{1}H-^{1}H-COSY spectrum (C_6D_6) of **13**.

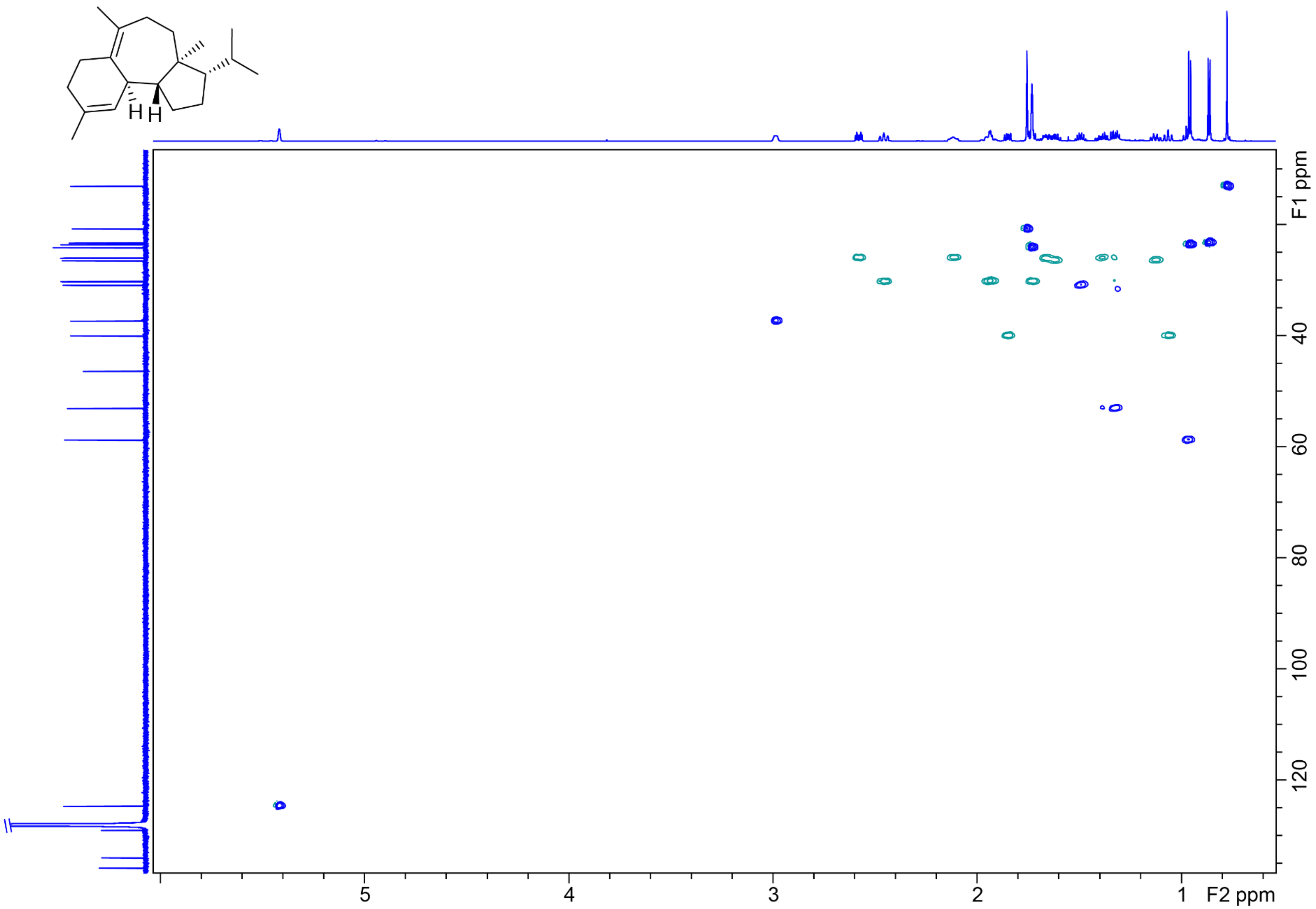

Figure S21. HSQC spectrum (C_6D_6) of **13**.

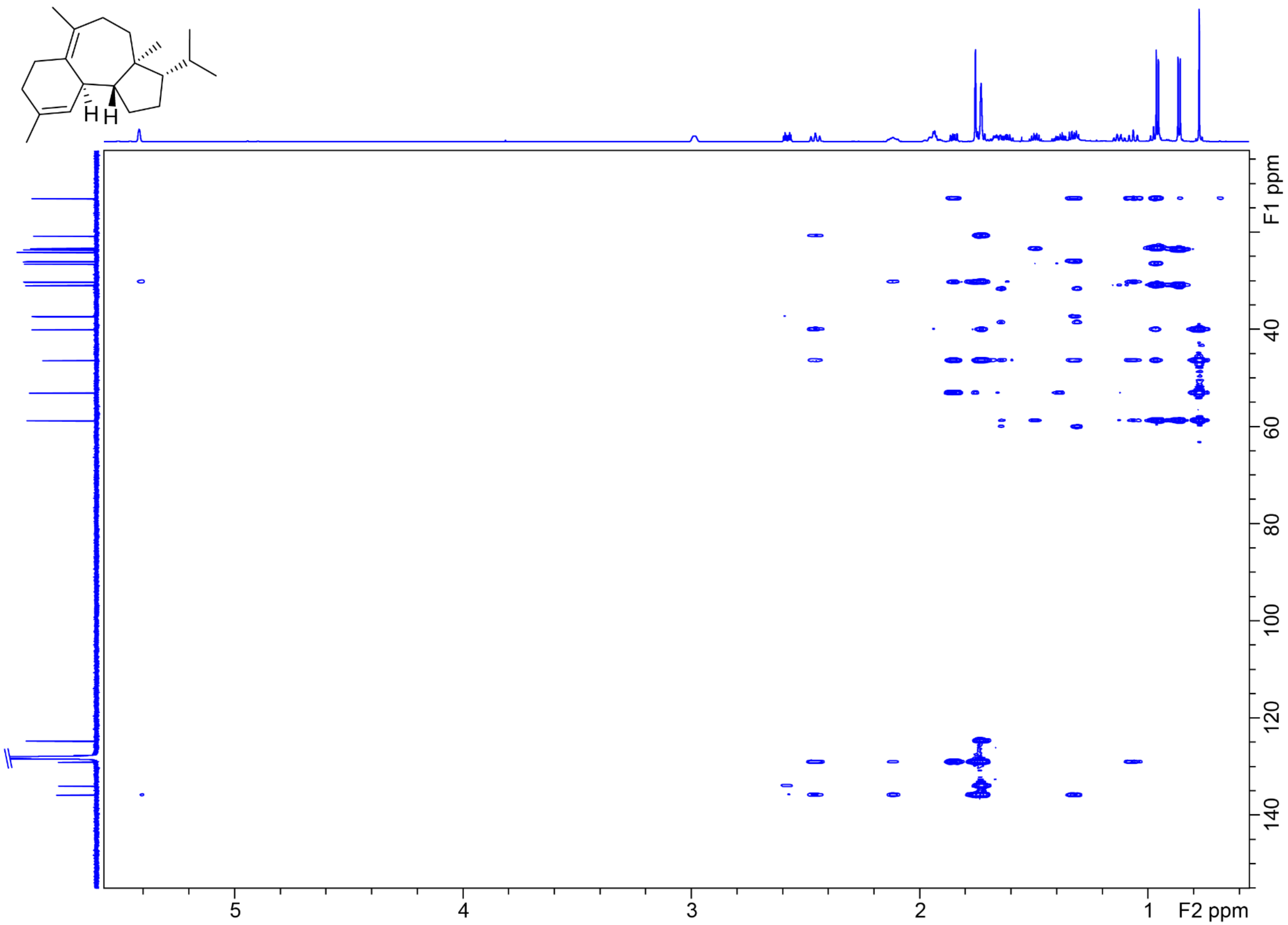

Figure S22. HMBC spectrum (C_6D_6) of **13**.

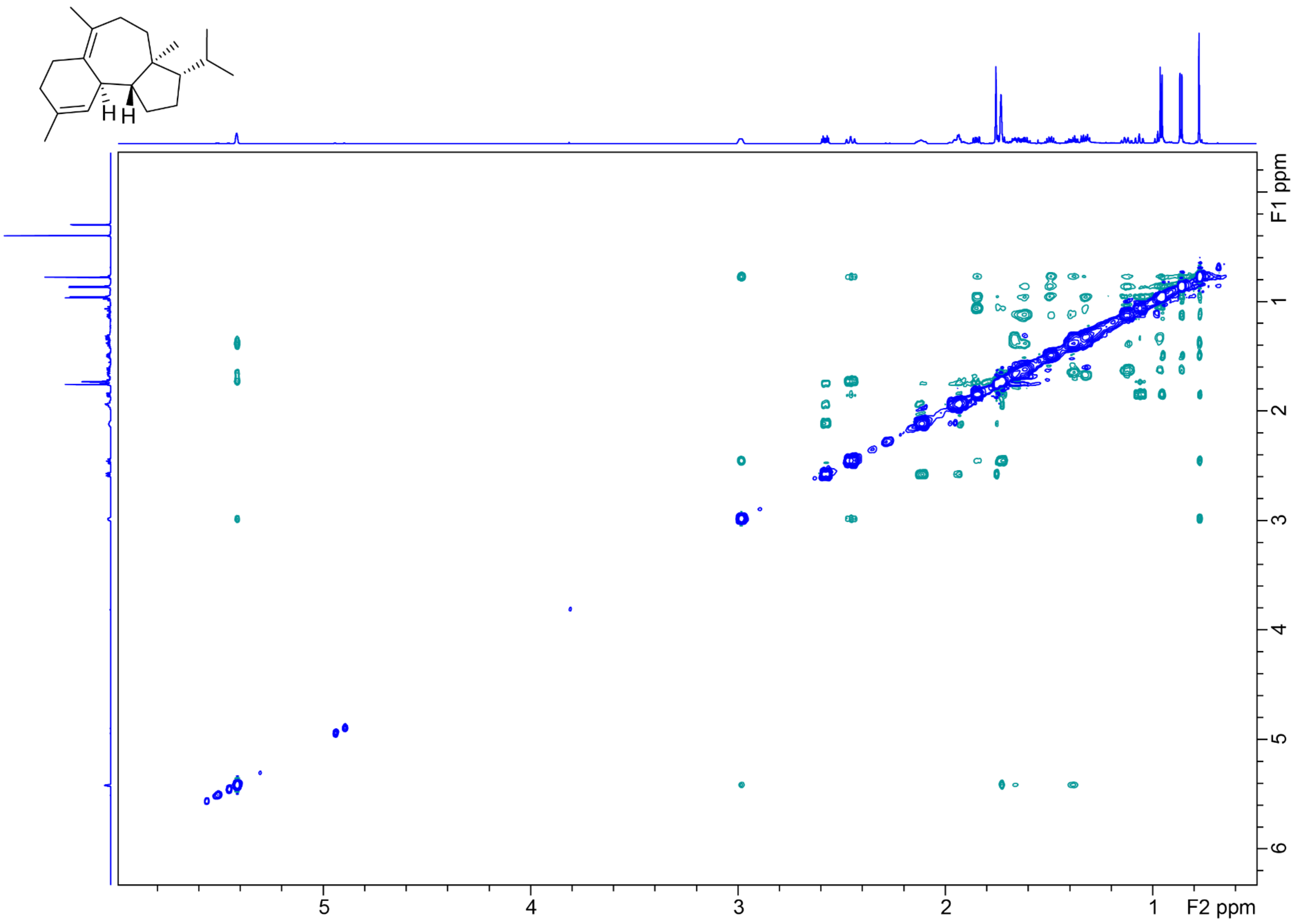

Figure S23. NOESY spectrum (C_6D_6) of **13**.

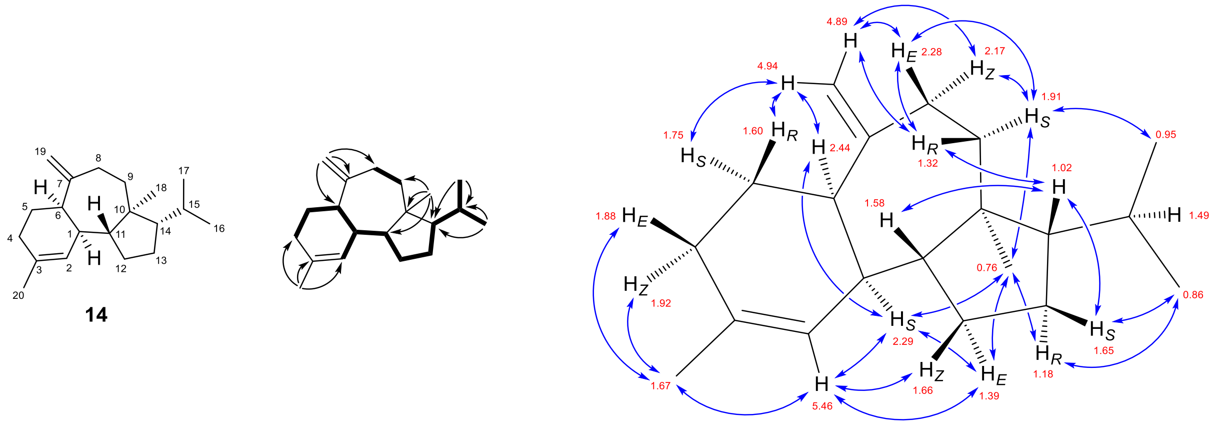

Figure S24. Structure elucidation of isosphaerodiene 2 (**14**). Bold: $^{1}H,^{1}H$-COSY, single headed arrows: key HMBC, and double headed arrows: NOESY correlations. Carbon numbering follows GGPP numbering to indicate the origin of each carbon. H_R, H_S, H_E and H_Z indicate the results of stereoselective labelling experiments (Figures S60 and S63).

Table S5. NMR data of isosphaerodiene 2 (**14**) in C_6D_6 recorded at 298 K.

C[a]	type	^{13}C[b]	^{1}H[b]
1	CH	37.13	2.29 (m)
2	CH	126.22	5.46 (m)
3	C_q	132.76	–
4	CH_2	30.78	1.92 (m)
			1.88 (m)
5	CH_2	23.96	1.75 (m, 2H)
6	CH	45.07	2.44 (br d, J = 12.0)
7	C_q	155.18	–
8	CH_2	31.33	2.28 (m)
9	CH_2	43.39	1.91 (m)
			1.32 (m)
10	C_q	46.21	–
11	CH	51.73	1.58 (m)
12	CH_2	27.04	1.66 (m)
			1.39 (m)
13	CH_2	27.27	1.65 (m)
			1.18 (m)
14	CH	59.19	1.02 (m)
15	CH	30.61	1.49 (m)
16	CH_3	22.99	0.86 (d, J = 6.7)
17	CH_3	23.67	0.95 (d, J = 6.7)
18	CH_3	13.79	0.76 (s)
19	CH_2	110.66	4.94 (br s)
			4.89 (br s)
20	CH_3	23.92	1.67 (s)

[a] Carbon numbering as shown in Figure S24 indicates the origin of each carbon from GGPP by same number. [b] Chemical shifts δ in ppm, multiplicity: s = singlet, d = doublet, m = multiplet, br = broad, coupling constants J are given in Hertz.

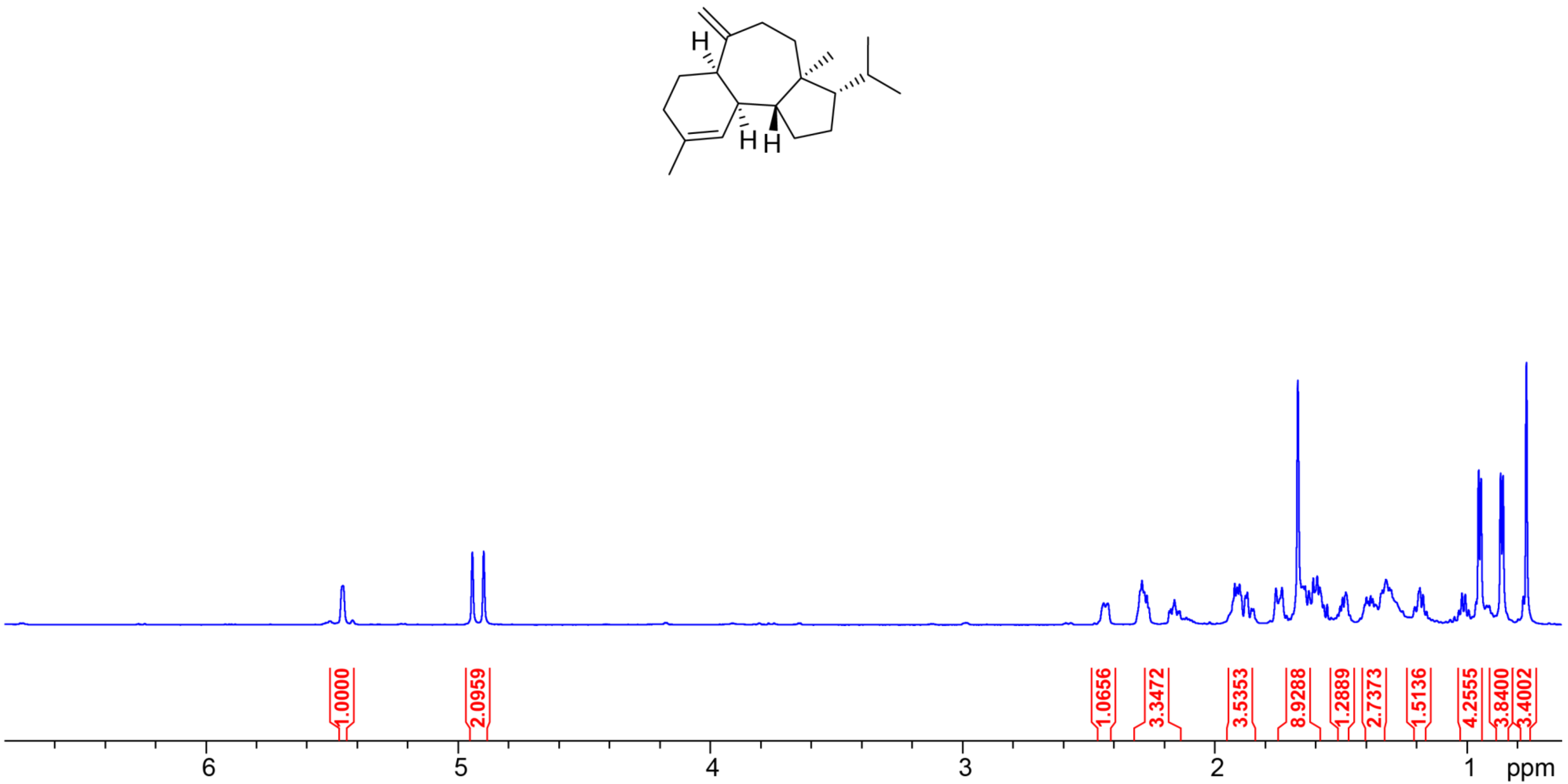

Figure S25. ^{1}H-NMR spectrum of **14** (700 MHz, C_6D_6).

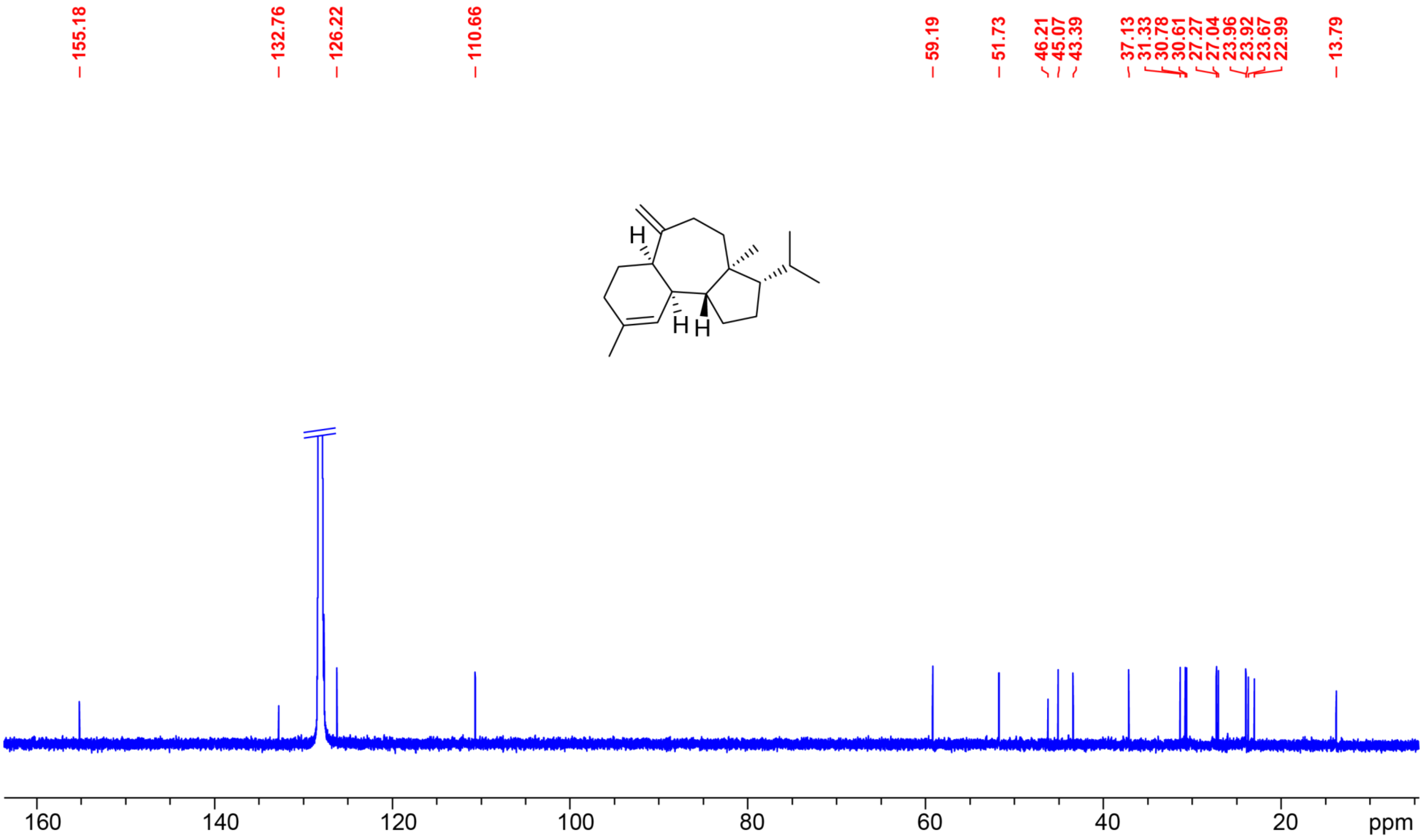

Figure S26. ^{13}C-NMR spectrum of **14** (176 MHz, C_6D_6).

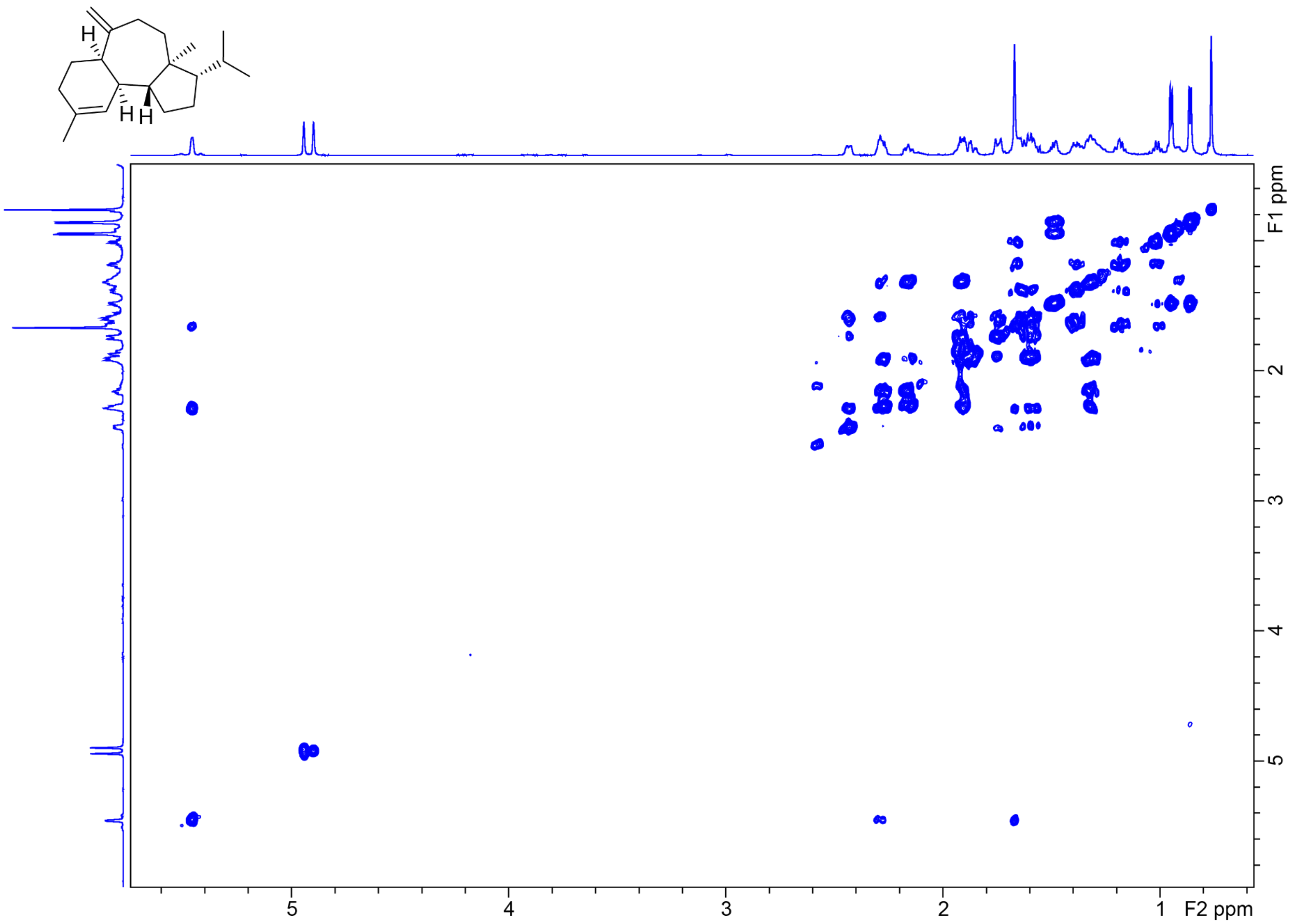

Figure S27. ^{1}H-^{1}H-COSY spectrum (C_6D_6) of **14**.

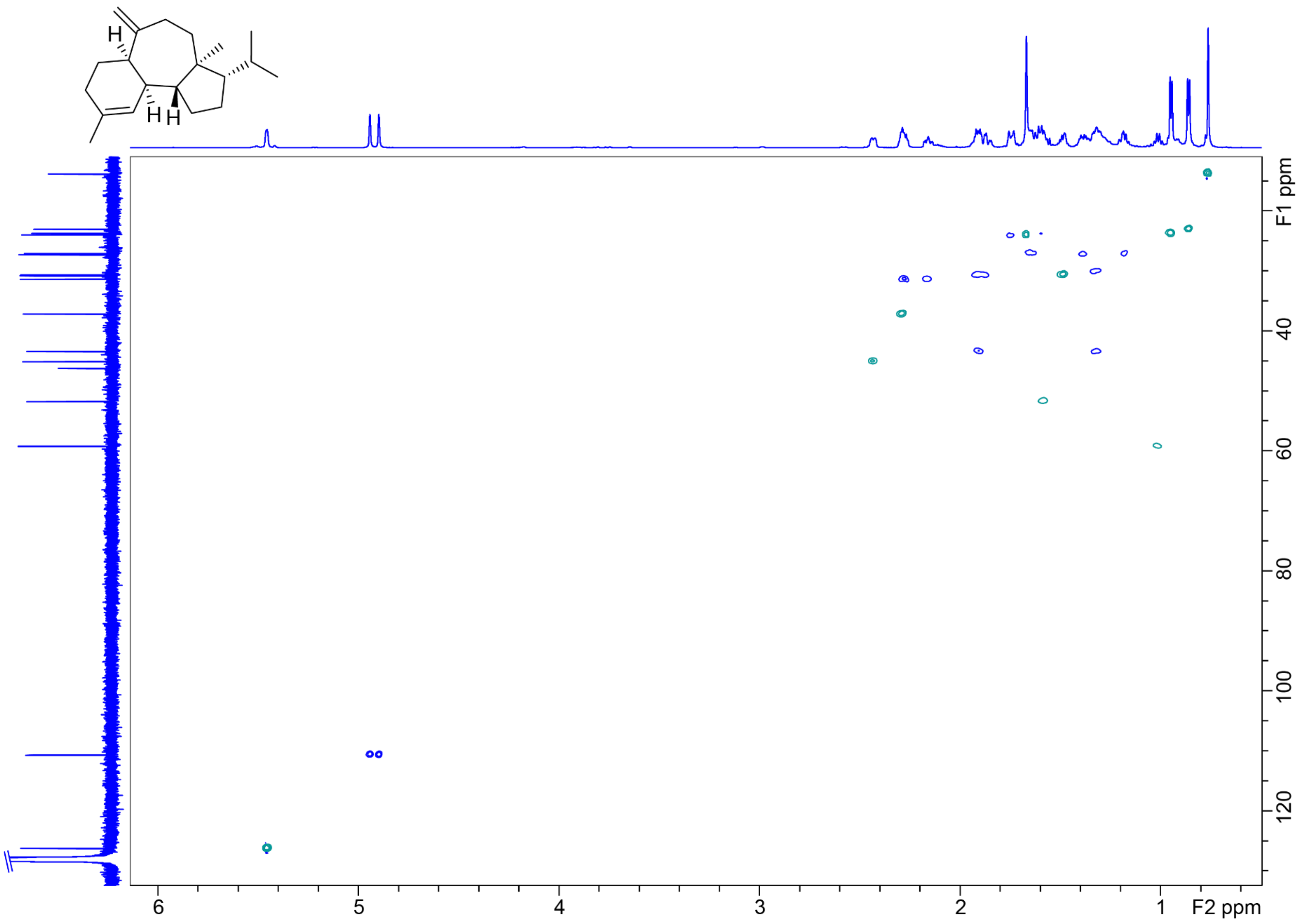

Figure S28. HSQC spectrum (C_6D_6) of **14**.

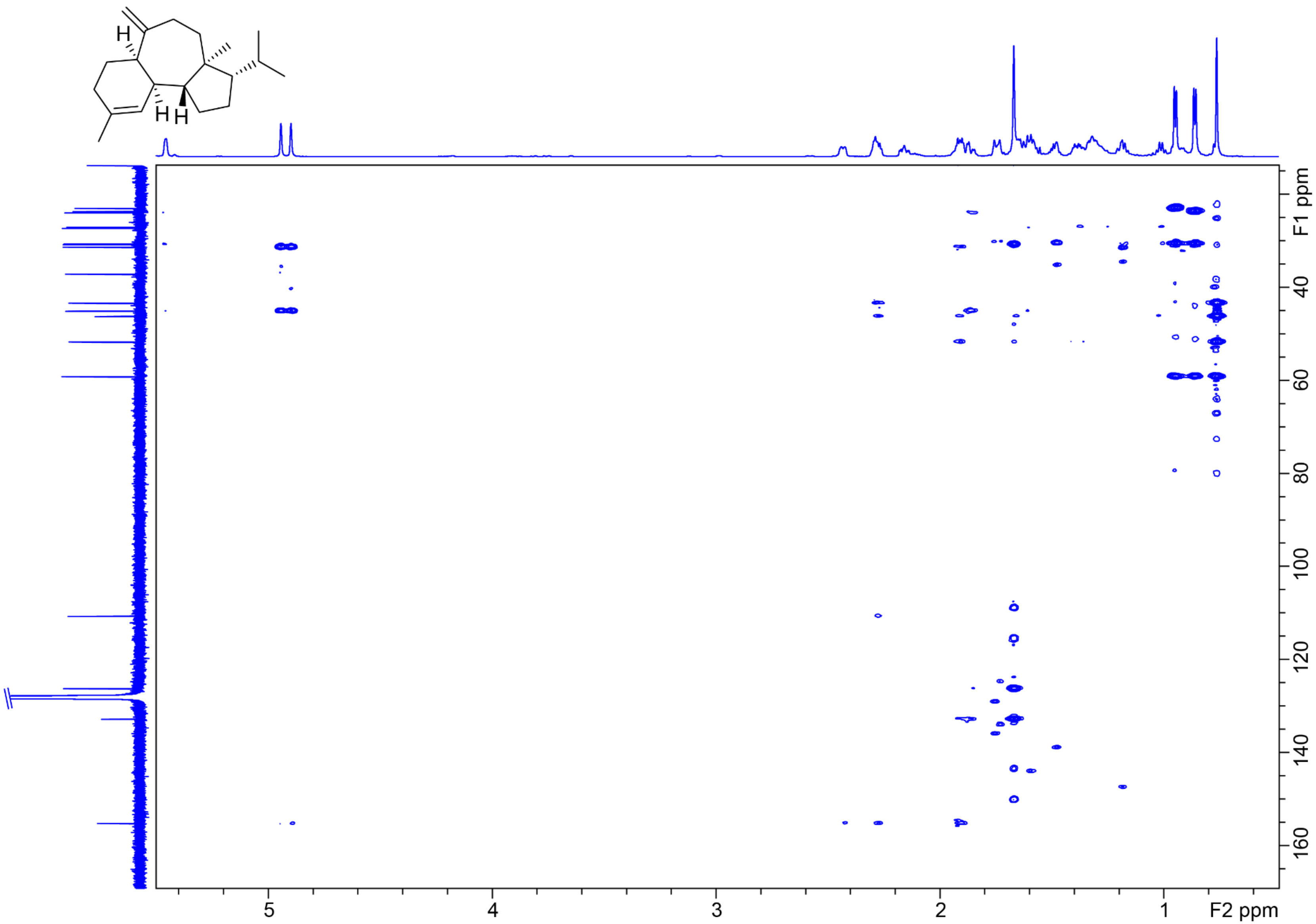

Figure S29. HMBC spectrum (C_6D_6) of **14**.

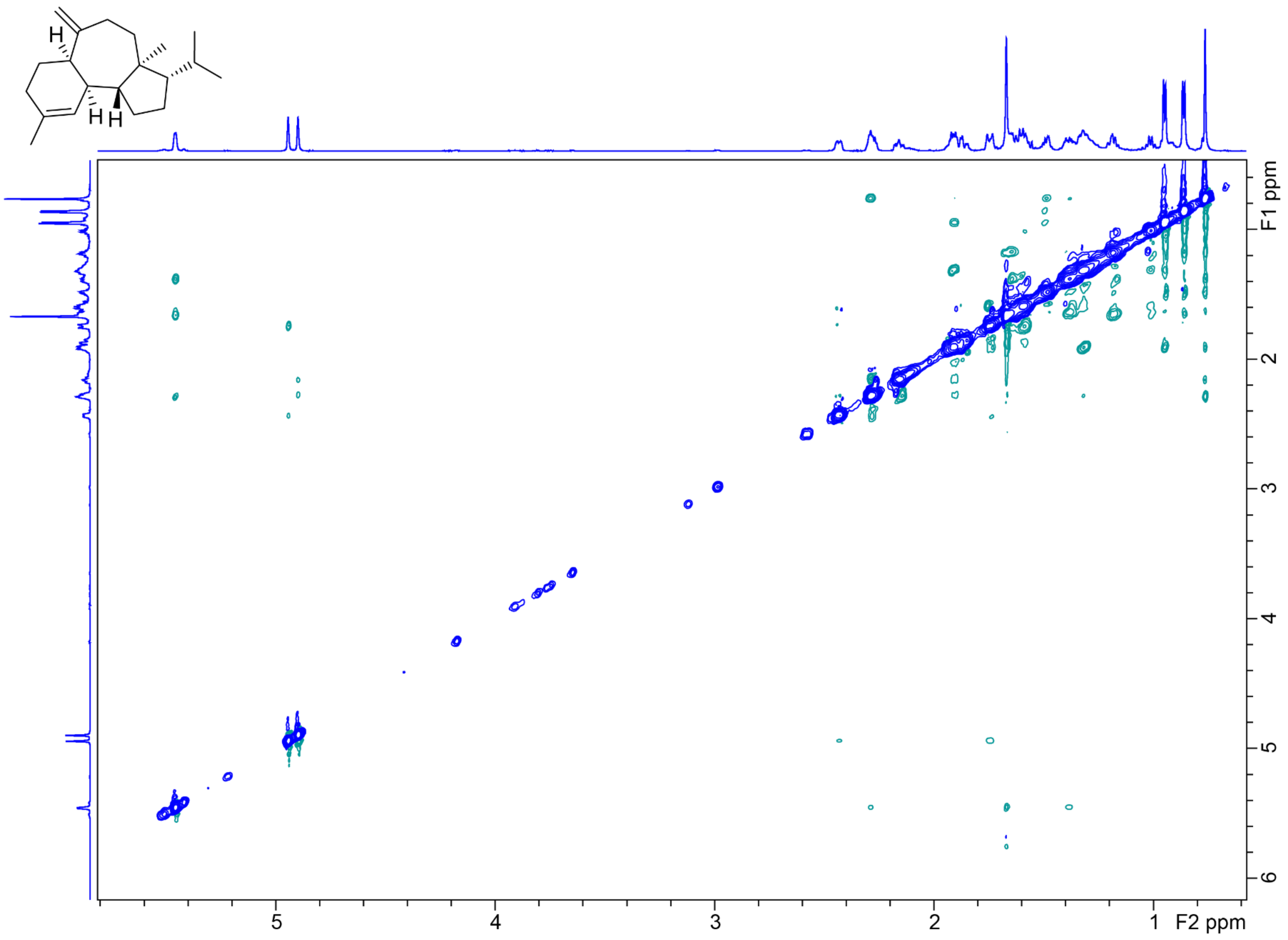

Figure S30. NOESY spectrum (C_6D_6) of **14**.

A)

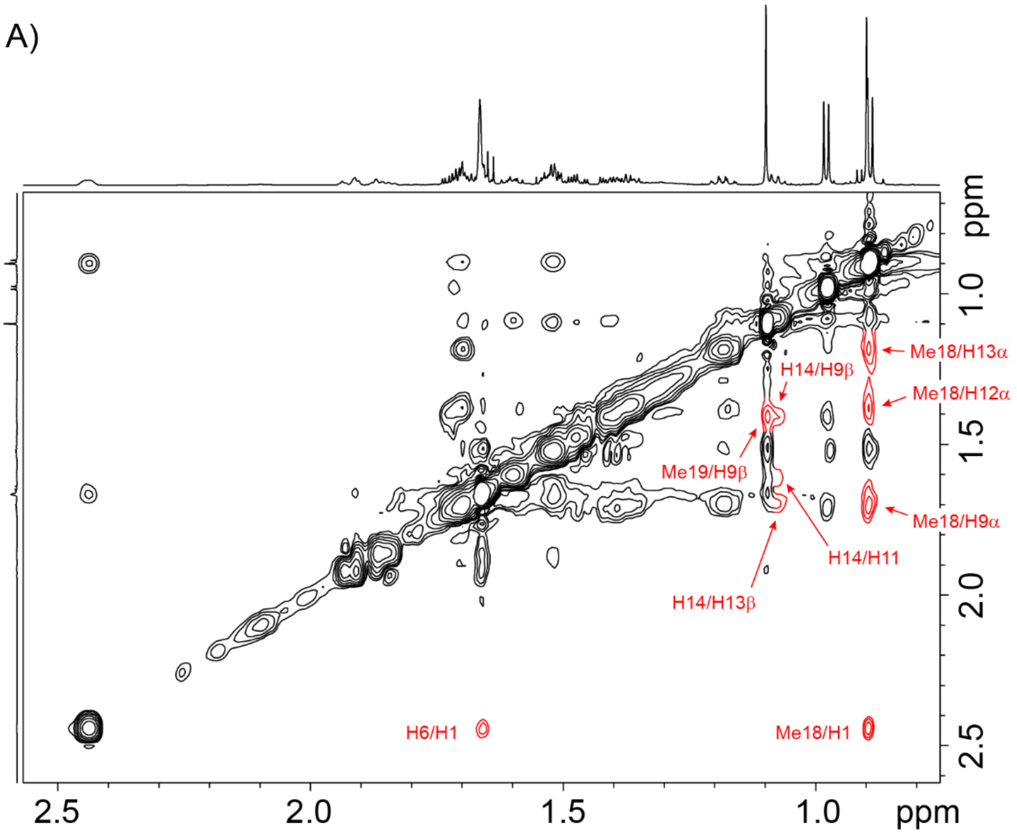

B)

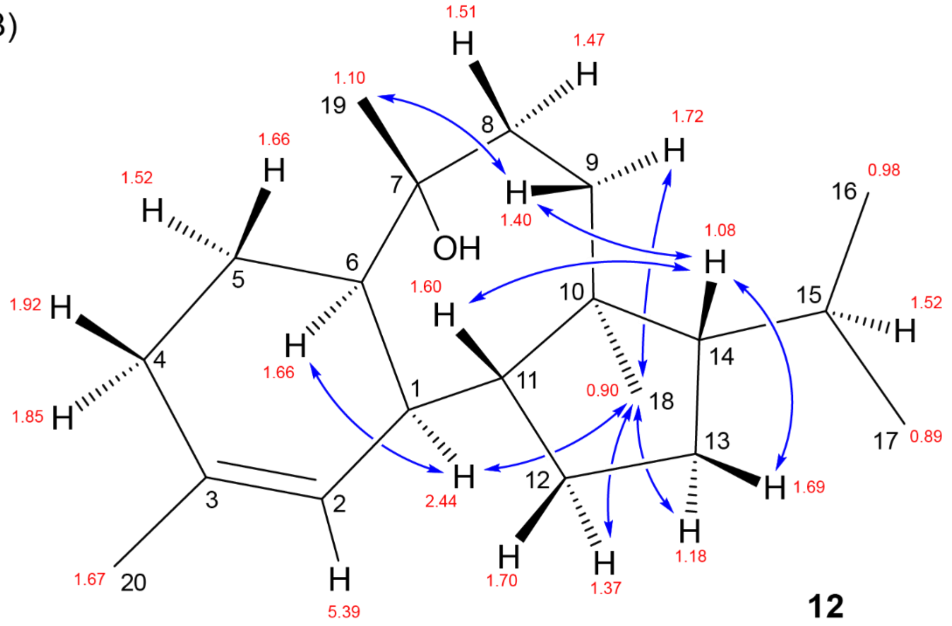

Figure S31. Structure elucidation of **12**. A) NOESY spectrum of **12**. The crosspeaks highlighted in red are the NOESY correlations indicated by the blue arrows at the structure. B) Key NOESY correlations of **12** (blue double headed arrows). Carbon numbers are shown in black, ^{1}H chemical shifts in ppm are shown in red.

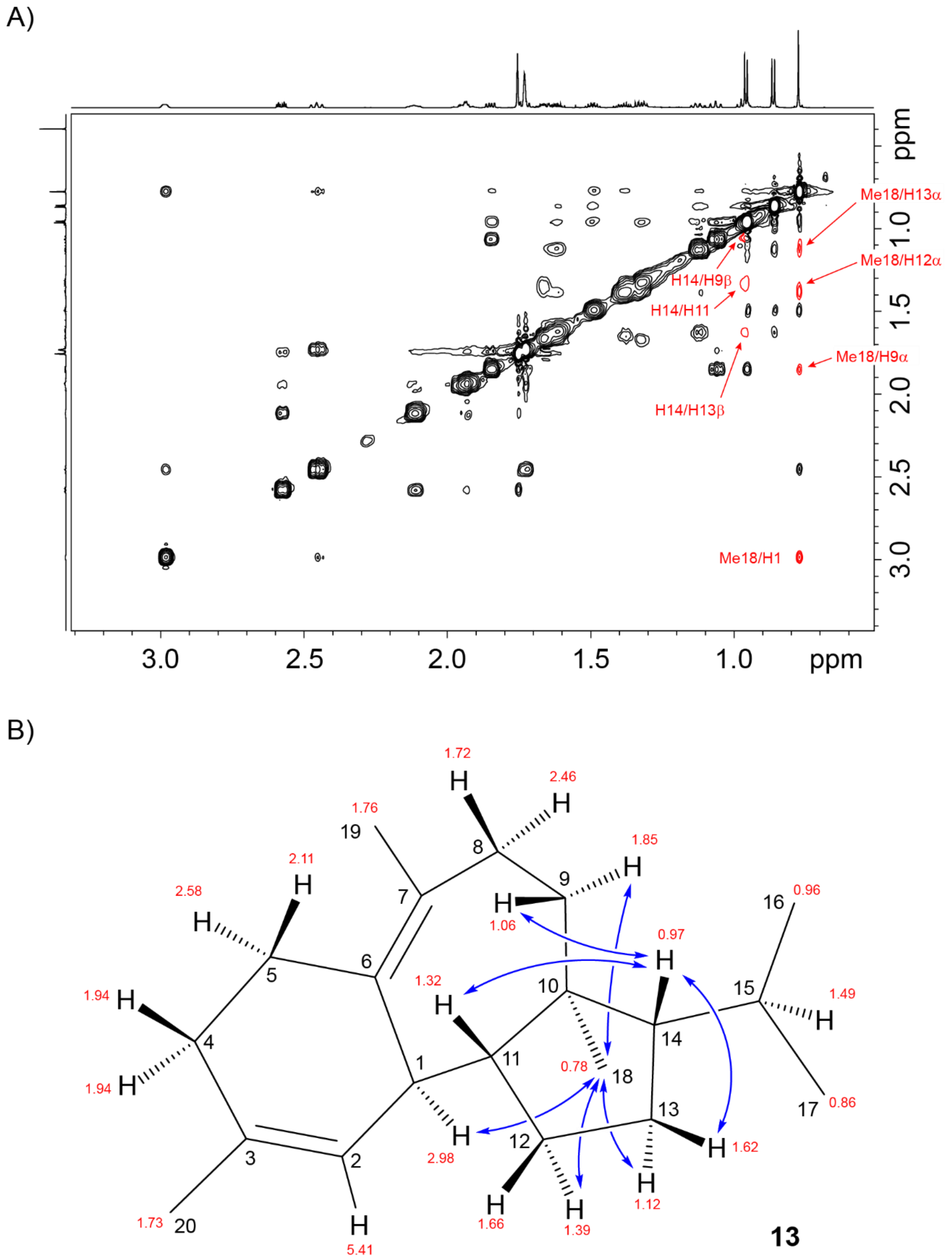

Figure S32. Structure elucidation of **13**. A) NOESY spectrum of **13**. The crosspeaks highlighted in red are the NOESY correlations indicated by the blue arrows at the structure. B) Key NOESY correlations of **13** (blue double headed arrows). Carbon numbers are shown in black, ^{1}H chemical shifts in ppm are shown in red.

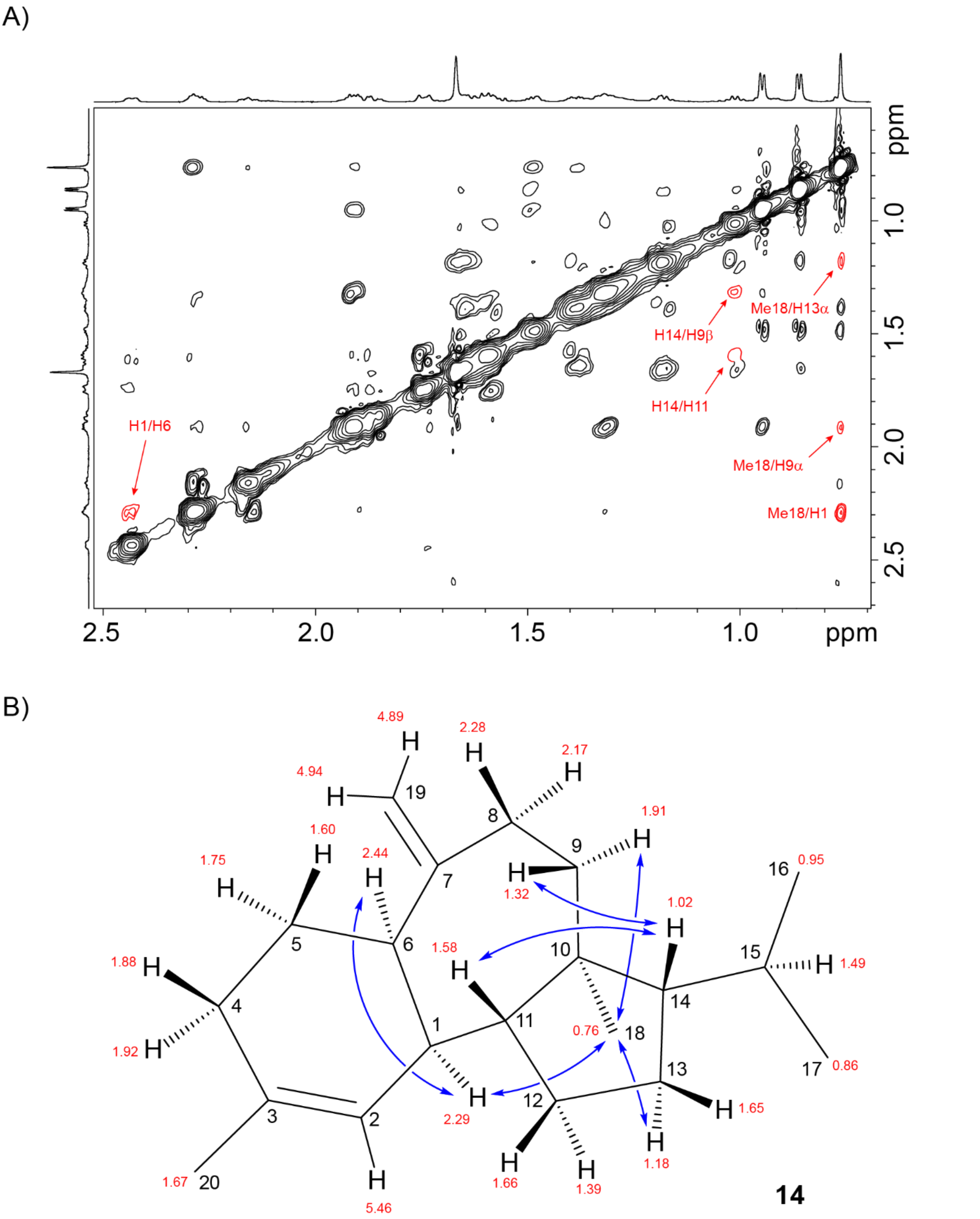

Figure S33. Structure elucidation of **14**. A) NOESY spectrum of **14**. The crosspeaks highlighted in red are the NOESY correlations indicated by the blue arrows at the structure. B) Key NOESY correlations of **14** (blue double headed arrows). Carbon numbers are shown in black, ^{1}H chemical shifts in ppm are shown in red.

Preparative scale incubation with recombinant CjCsS and isolation of 11

A preparative scale enzymatic conversion of GGPP (80 mg, 160 μmol) was conducted in incubation buffer (140 mL) with the addition of an enzyme preparation of CjCsS (20 mL, 3.6 mg mL^{-1}), followed by incubation for 16 h at 30 °C. The reaction mixture was extracted with n-pentane (3 x 100 mL), the combined extracts were dried with $MgSO_4$ and the solvent was evaporated to yield crude **11** (ca. 95% pure, 0.8 mg, 2.8 μmol, 1.8%).

Chitinosphaerol (11). TLC (pentane:Et_2O = 2:1): R_f = 0.44. GC (HP-5MS): *I* = 2281. MS (EI, 70 eV): *m*/*z* (%) = 41 (3), 55 (4), 67 (5), 81 (10), 84 (18), 95 (14), 109 (7), 121 (7), 135 (5), 149 (3), 153 (7), 163 (3), 173 (1), 191 (1), 208 (1), 229 (3), 247 (2), 272 (1), 290 (3). IR (diamond ATR): ṽ = 3455 (br), 2947 (s), 2922 (s), 2869 (m), 1665 (w), 1445 (w), 1384 (w), 1260 (w), 1076 (w), 1022 (w), 849 (w), 797 (w) cm^{-1}. HR-MS (Q-TOF, 70 eV): calc. $[C_{20}H_{34}O]^+$ *m*/*z* = 290.2604; found: *m*/*z* = 290.2599. Optical rotation: $[\alpha]_D^{25}$ = –43.3 (*c* 0.18, CH_2Cl_2). NMR data are given in Tables S6 and S7.

A)

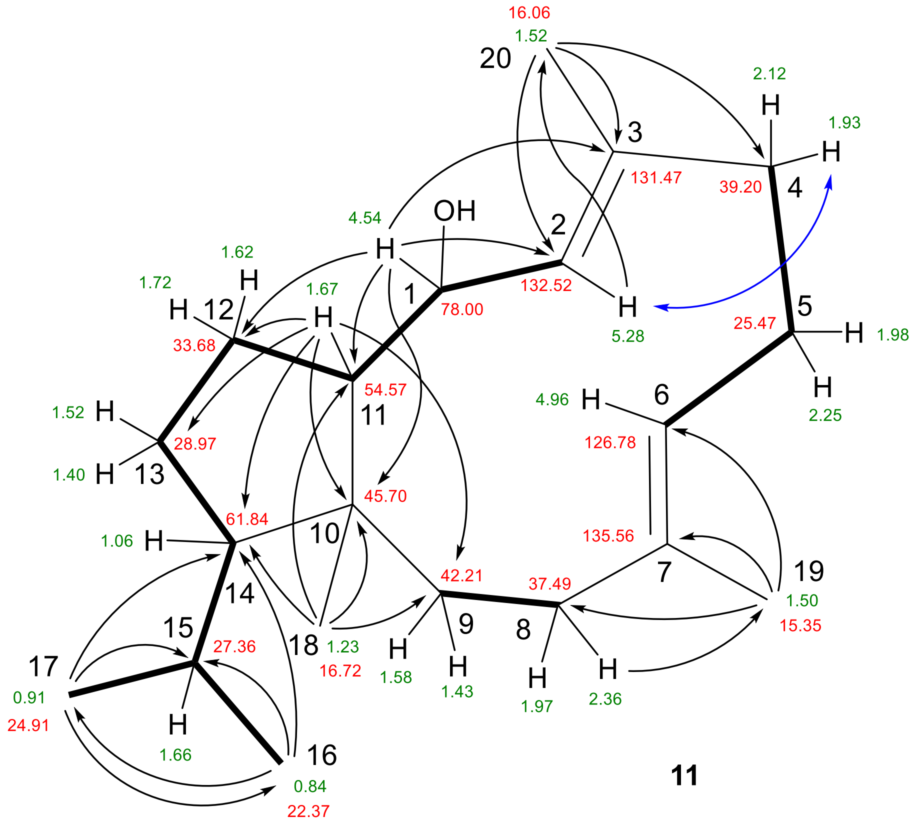

11

B)

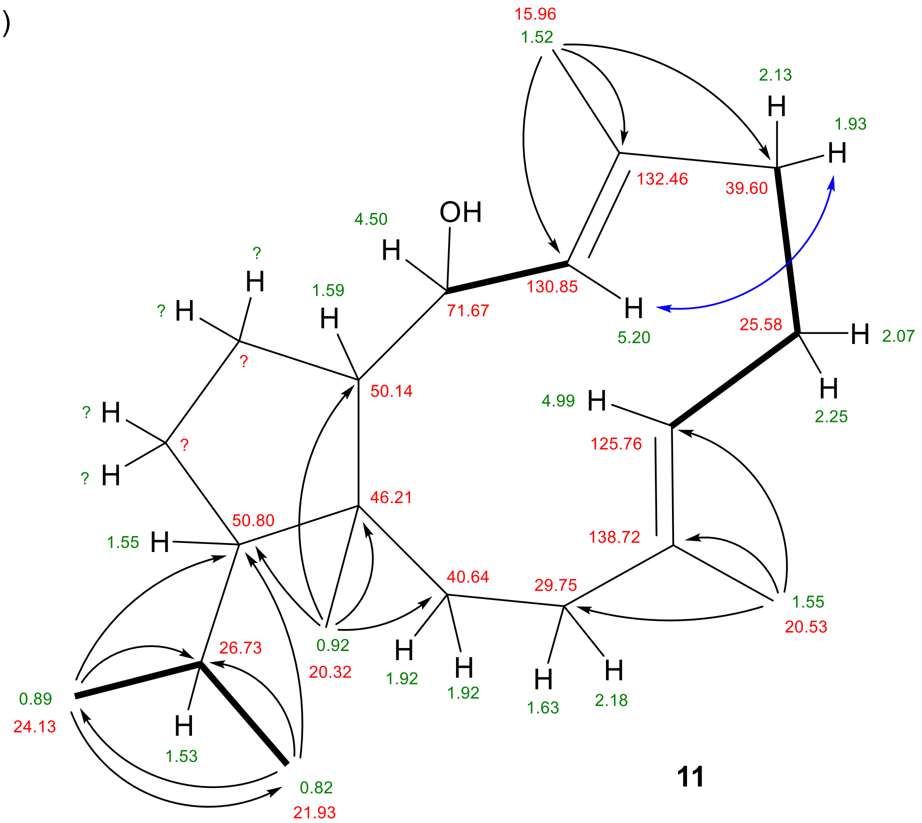

11

Figure S34. Structure elucidation of chitinosphaerol (**11**). A) Conformer 1 (Table S6) and B) conformer 2 (Table S7). Bold: ^{1}H,^{1}H-COSY, single headed arrows: key HMBC, and blue: key NOESY correlations, green: ^{1}H-NMR chemical shifts, red: ^{13}C-NMR chemical shifts in ppm. Carbon numbering (black) follows GGPP numbering to indicate the origin of each carbon.

Table S6. NMR data of chitinosphaerol (**11**, conformer 1) in C_6D_6 recorded at 238 K.

C[a]	type	^{13}C[b]	^{1}H[b]
1	CH	78.00	4.54 (d, *J* = 9.1)
2	CH	132.52	5.28 (d, *J* = 9.1)
3	C_q	131.46	–
4	CH_2	39.20	2.13 (m)<br>1.93 (m)
5	CH_2	25.47	2.25 (m)<br>1.98 (m)
6	CH	126.78	4.96 (d, *J* = 11.6)
7	C_q	135.56	–
8	CH_2	37.49	2.36 (dd, *J* = 13.4, 13.4)<br>1.97 (m)
9	CH_2	42.21	1.58 (m)<br>1.43 (m)
10	CH_q	45.70	–
11	CH	54.57	1.67 (m)
12	CH_2	33.68	1.72 (m)<br>1.62 (m)
13	CH_2	28.97	1.52 (m)<br>1.40 (m)
14	CH	61.84	1.06 (m)
15	CH	27.36	1.66 (m)
16	CH_3	22.37	0.84 (m)
17	CH_3	24.91	0.91 (m)
18	CH_3	16.72	1.23 (br s)
19	CH_3	15.35	1.50 (br s)
20	CH_3	16.06	1.52 (br s)

[a] Carbon numbering as shown in Figure S34 indicates the origin of each carbon from GGPP by same number. [b] Chemical shifts δ in ppm, multiplicity: s = singlet, d = doublet, m = multiplet, br = broad, coupling constants *J* are given in Hertz.

Table S7. NMR data of chitinosphaerol (**11**, conformer 2) in C_6D_6 recorded at 238 K.

C[a]	type	^{13}C[b]	^{1}H[b]
1	CH	71.67	4.50 (d, *J* = 8.5)
2	CH	130.85	5.20 (m)
3	C_q	132.46	–
4	CH_2	39.60	2.13 (m)<br>1.93 (m)
5	CH_2	25.58	2.25 (m)<br>2.07 (m)
6	CH	125.76	4.99 (br s)
7	C_q	138.72	–
8	CH_2	29.75	2.18 (m)<br>1.63 (m)
9	CH_2	40.64	1.92 (m, 2H)
10	CH_q	46.21	–
11	CH	50.14	1.59 (m)
12	CH_2	?	?
13	CH_2	?	?
14	CH	50.80	1.55 (m)
15	CH	26.73	1.53 (m)
16	CH_3	21.93	0.82 (d, *J* = 6.2)
17	CH_3	24.13	0.89 (d, *J* = 6.4)
18	CH_3	20.32	0.92 (s)
19	CH_3	20.53	1.55 (br s)
20	CH_3	15.96	1.52 (br s)

[a] Carbon numbering as shown in Figure S34 indicates the origin of each carbon from GGPP by same number. [b] Chemical shifts δ in ppm, multiplicity: s = singlet, d = doublet, m = multiplet, br = broad, coupling constants *J* are given in Hertz.

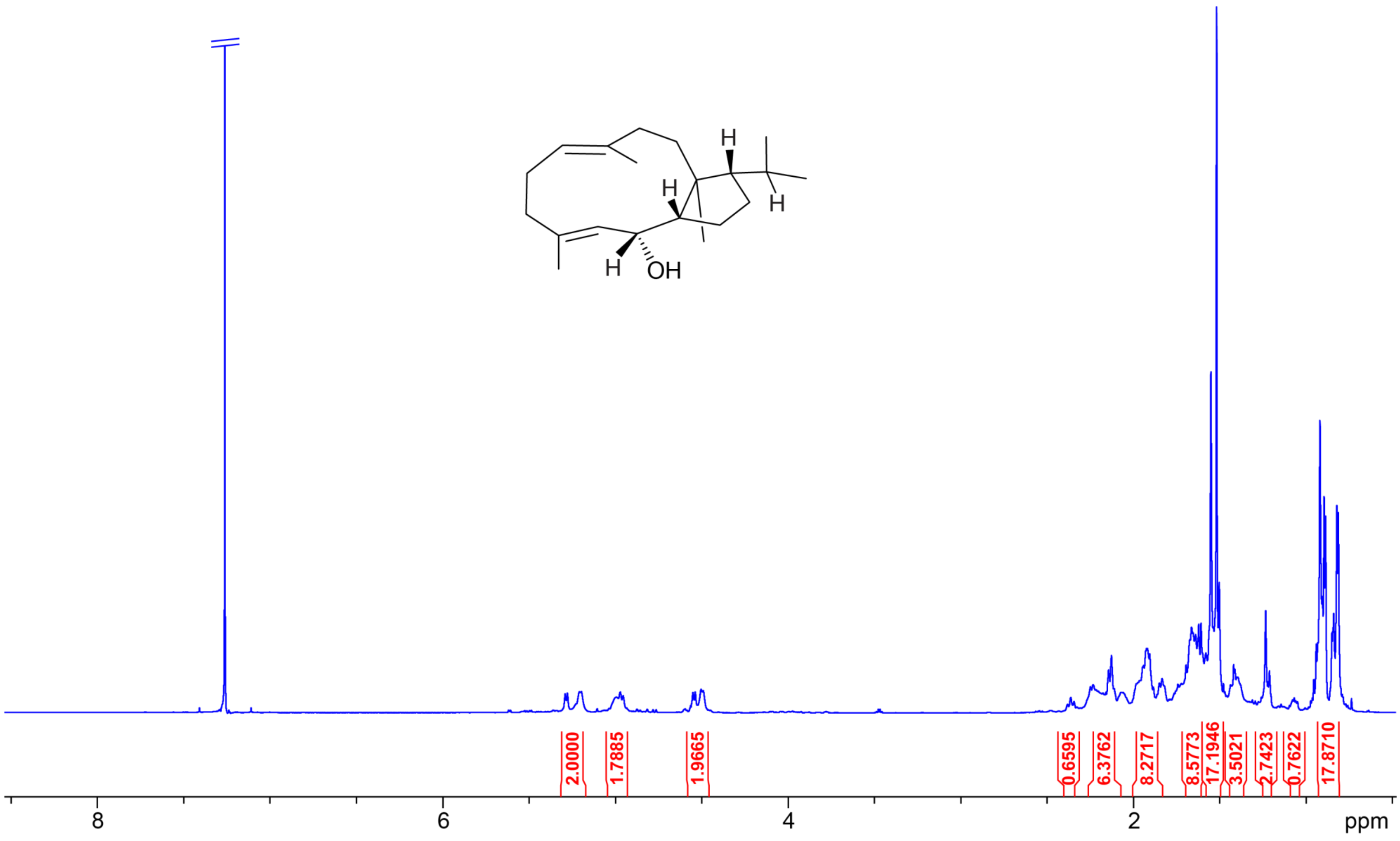

Figure S35. ^{1}H-NMR spectrum of **11** (700 MHz, C_6D_6).

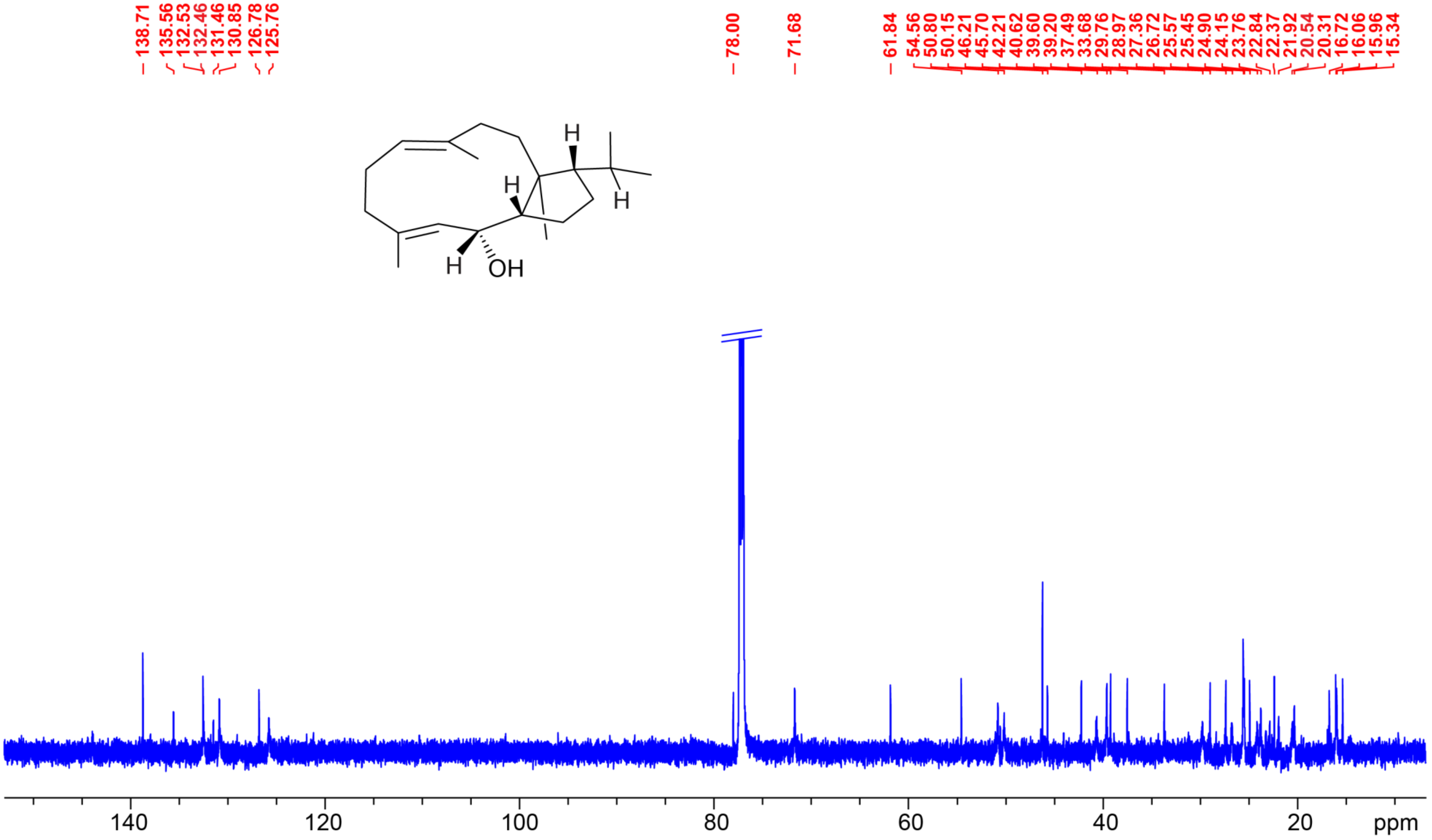

Figure S36. ^{13}C-NMR spectrum of **11** (176 MHz, C_6D_6).

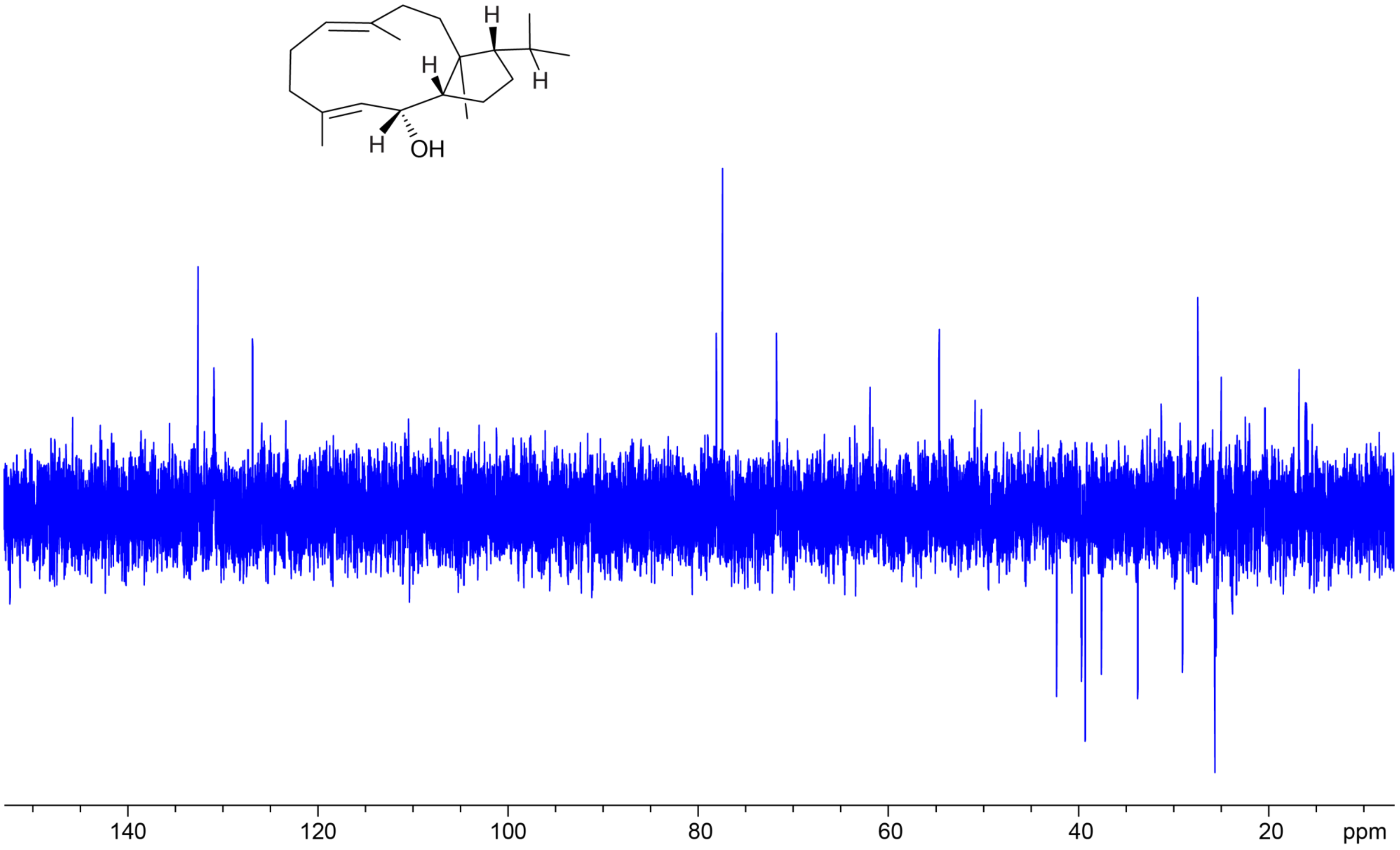

Figure S37. ^{13}C-DEPT135 spectrum of **11** (176 MHz, C_6D_6).

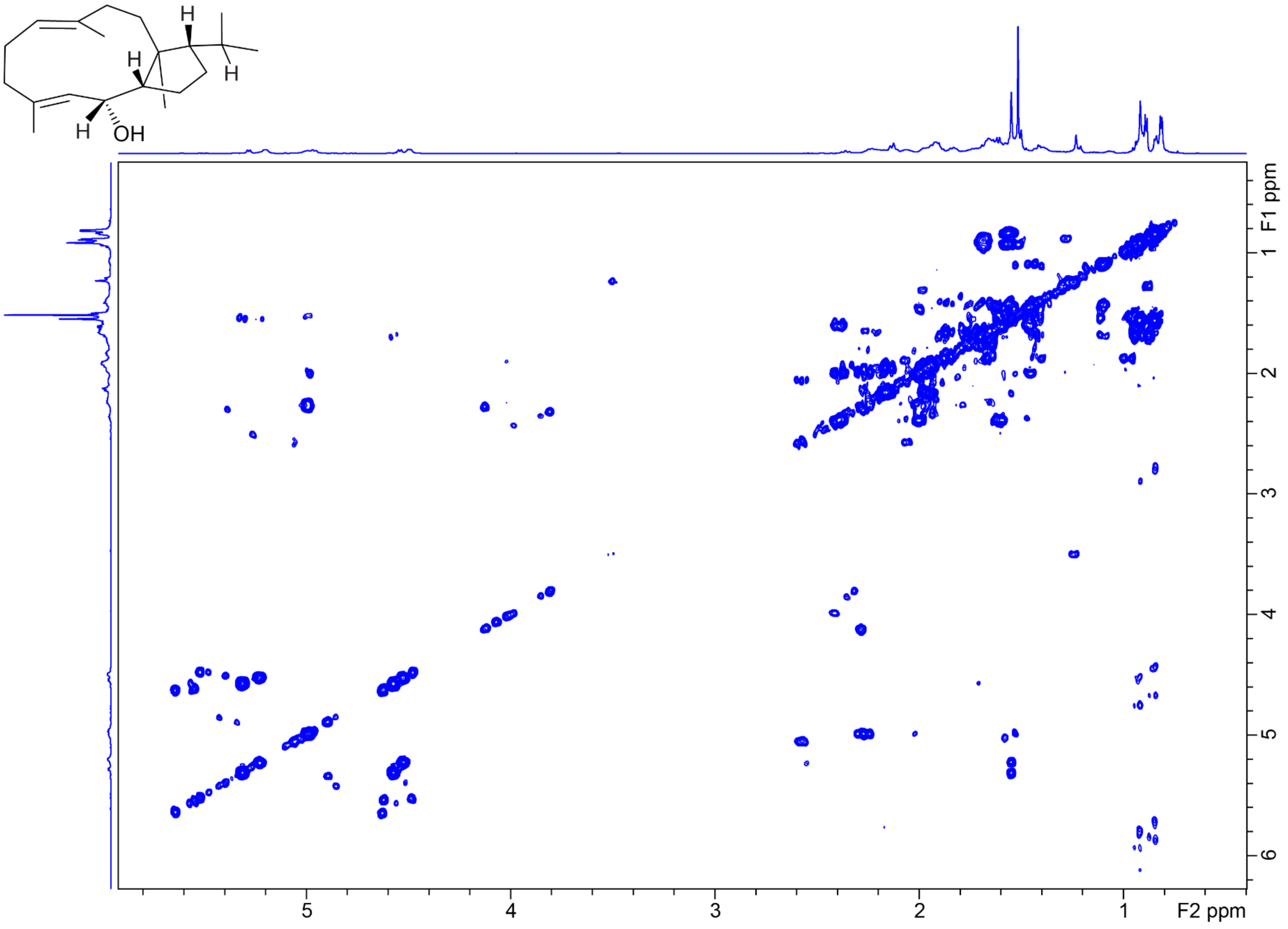

Figure S38. ^{1}H-^{1}H-COSY spectrum (C_6D_6) of **11**.

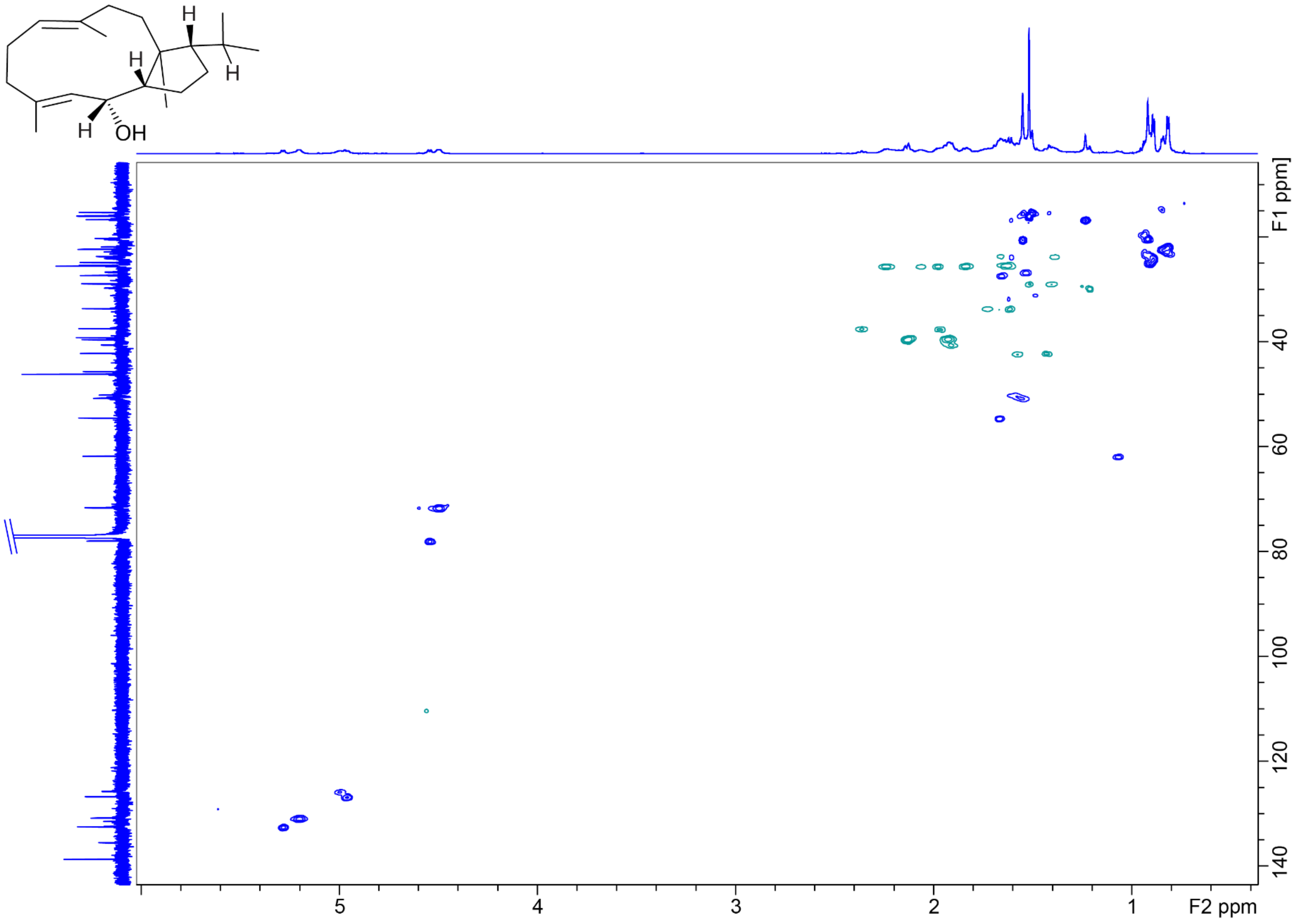

Figure S39. HSQC spectrum (C_6D_6) of **11**.

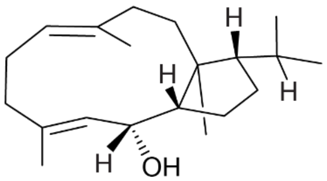

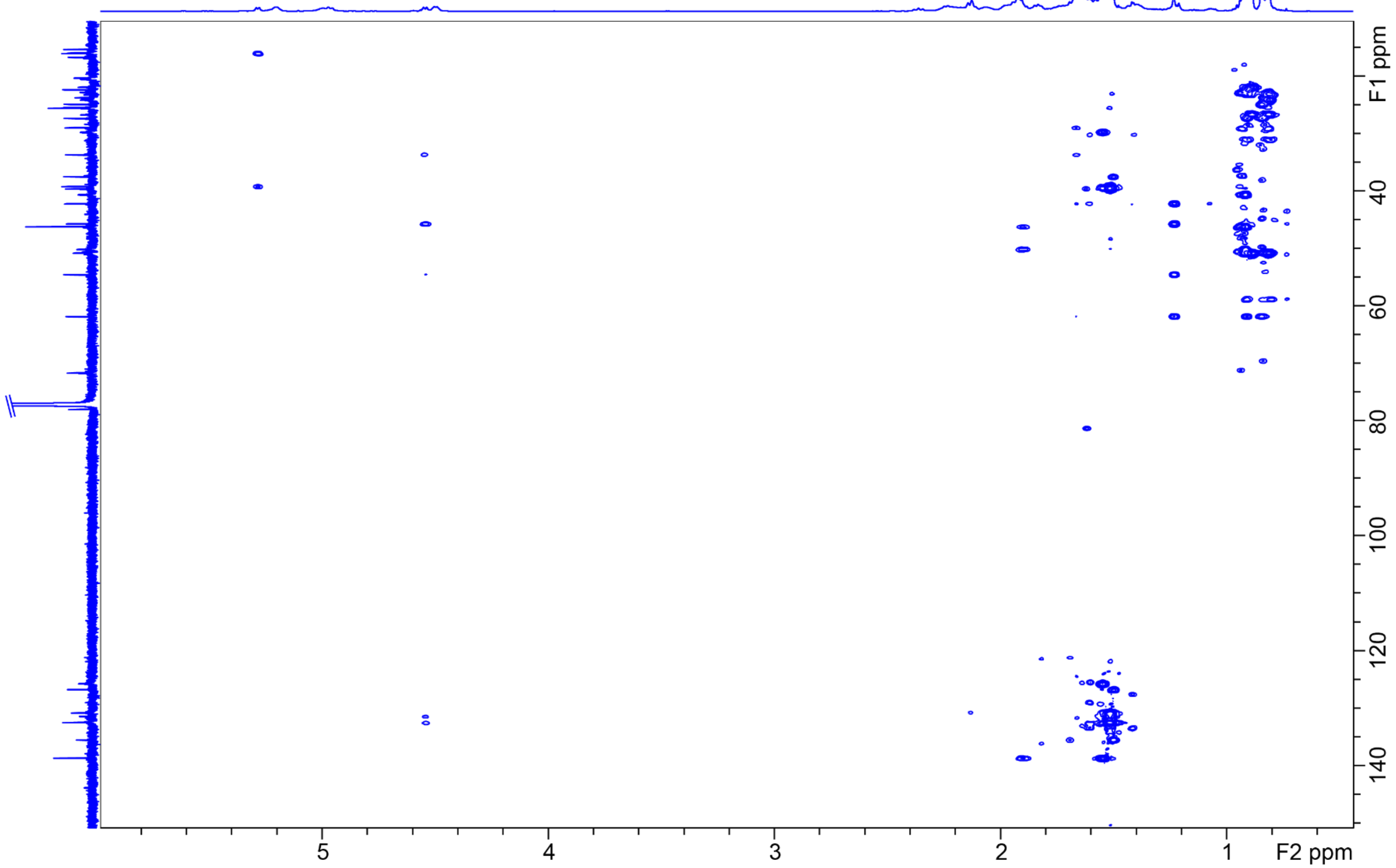

Figure S40. HMBC spectrum (C_6D_6) of **11**.

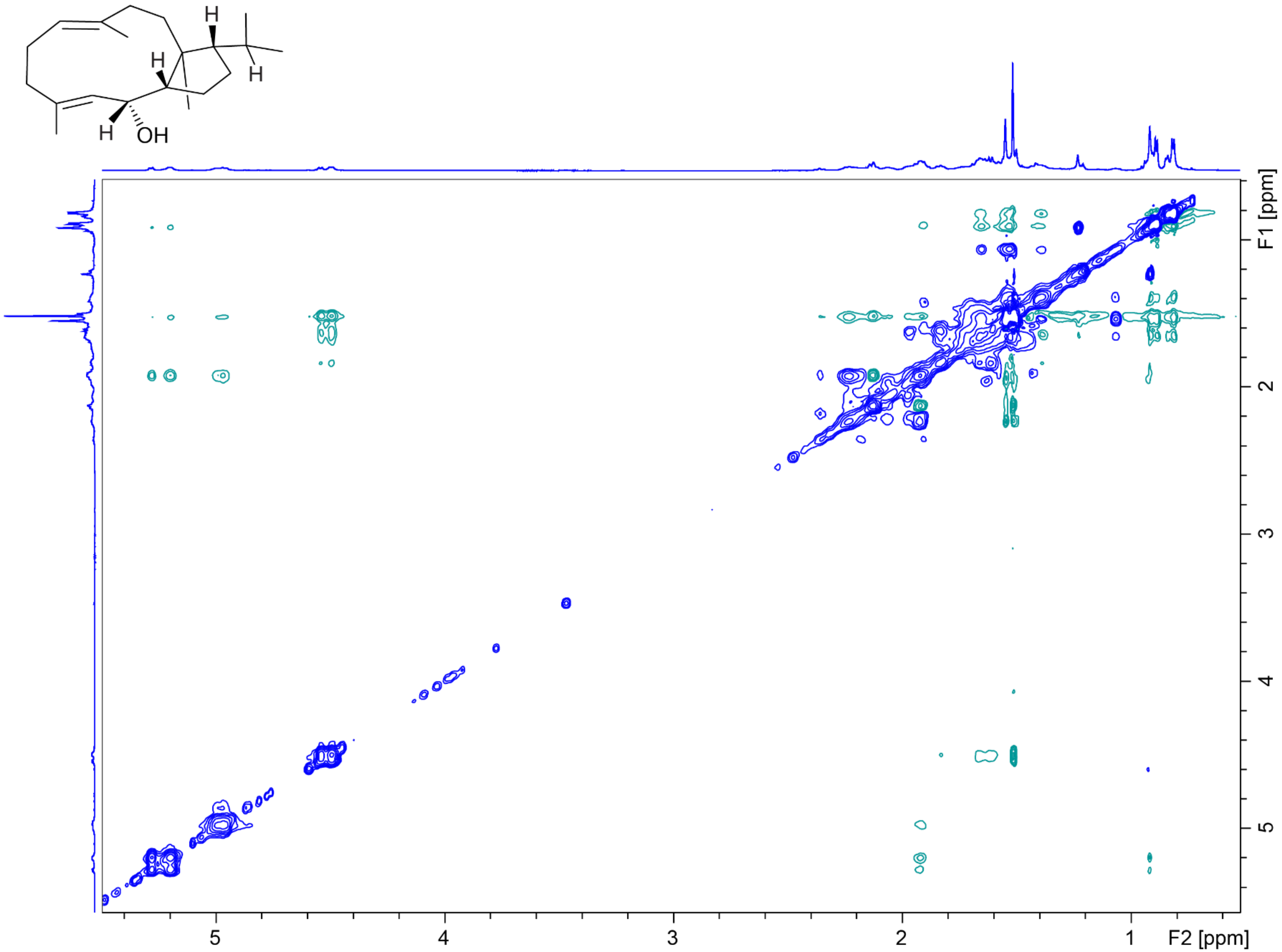

Figure S41. NOESY spectrum (C_6D_6) of **11**.

Conversion of 11 with N-bromosuccinimide

To a solution of **11** (5.5 mg, 19 μmol) in CH_2Cl_2 (1.0 mL) was added N-bromosuccinimide (4.32 mmol, 19 μmol, dissolved in 1.0 mL CH_2Cl_2) at –78 °C under argon atmosphere. After stirring at –78 °C for 20 min, the reaction mixture warmed to room temperature over 1 h and stirring was continued for another 40 h. The reaction was quenched by the addition of sat. $NaHCO_3$ solution and extracted with n-pentane (3x 50 mL). The combined organic layers were dried with $MgSO_4$ and concentrated under reduced pressure. The residues were subjected to column chromatography (n-pentane) and the fraction was collected, followed by HPLC separation to yield pure **17** (1.43 mg, 3.9 μmol, 21%) and **18** (0.36 mg, 1.0 μmol, 5.2%) as colourless oils.

Bromosphaerol A (17). TLC (pentane/Et_2O = 2:1): R_f = 0.38. GC (HP-5MS): *I* = 2630. MS (EI, 70 eV): *m*/*z* (%) = 41 (3), 55 (5), 67 (5), 81 (8), 95 (8), 107 (20), 121 (8), 135 (5), 153 (10), 163 (3), 179 (3), 187 (11), 201 (2), 215 (3), 227 (1), 271 (2), 289 (3), 309 (1), 337 (1), 353 (0.2), 368 (0.1). IR (diamond ATR): ṽ = 3596 (w), 2953 (s), 2929 (s), 2868 (m), 1999 (w), 1976 (w), 1729 (w), 1666 (w), 1644 (w), 1454 (w), 1382 (w), 1260 (w), 1207 (w), 1110 (w), 1073 (w), 908 (w), 892 (w), 733 (w), 678 (w) cm^{-1}. HR-MS (Q-TOF, 70 eV): calc. $[C_{20}H_{33}OBr]^+$ *m*/*z* = 368.1709; found: *m*/*z* = 368.1710. Optical rotation: $[\alpha]_D^{25}$ = –23.3 (*c* 0.06, CH_2Cl_2). NMR data are given in Table S8.

Bromosphaerol B (18). TLC (pentane/Et_2O = 2:1): R_f = 0.38. GC (HP-5MS): *I* = 2702. MS (EI, 70 eV): *m*/*z* (%) = 41 (2), 55 (3), 69 (3), 81 (5), 95 (8), 107 (25), 119 (9), 135 (4), 153 (17), 163 (4), 179 (2), 187 (5), 201 (1), 219 (4), 227 (1), 271 (2), 289 (3), 307 (1), 337 (2), 368 (0). IR (diamond ATR): ṽ = 2954 (s), 2923 (s), 2853 (m), 1721 (w), 1674 (w), 1461 (w), 1384 (w), 1365 (w), 1260 (m), 1180 (w), 1087 (m), 1067 (m), 1016 (m), 966 (w), 798 (s), 698 (w) cm^{-1}. HR-MS (Q-TOF, 70 eV): calc. $[C_{20}H_{33}OBr]^+$ *m*/*z* = 368.1709; found: *m*/*z* = 368.1705. Optical rotation: $[\alpha]_D^{25}$ = –76.0 (*c* 0.05, CH_2Cl_2). NMR data are given in Table S9.

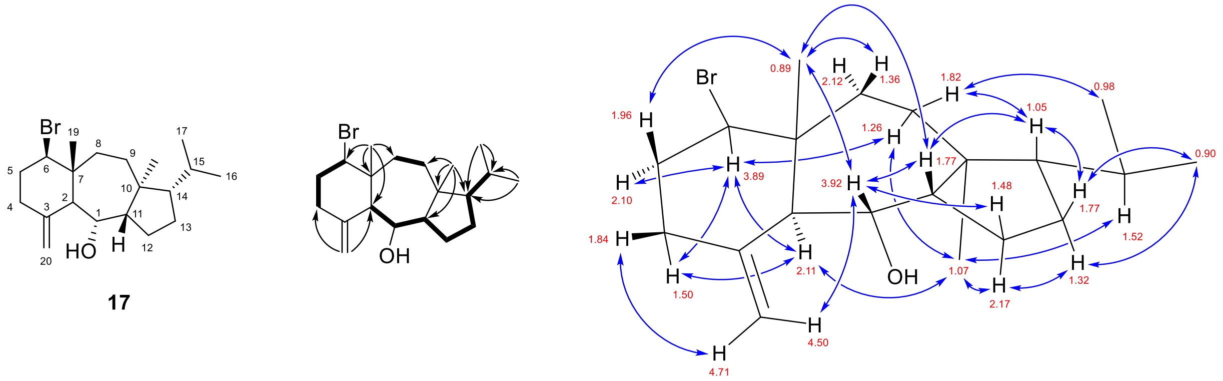

Figure S42. Structure elucidation of bromosphaerol A (**17**). Bold: ^{1}H,^{1}H-COSY, single headed arrows: key HMBC, and double headed arrows: NOESY correlations. Carbon numbering follows GGPP numbering to indicate the origin of each carbon.

Table S8. NMR data of bromosphaerol A (**17**) in C_6D_6 recorded at 298 K.

C[a]	type	^{13}C[b]	^{1}H[b]
1	CH	69.34	3.92 (m)
2	CH	53.63	2.11 (m)
3	C_q	146.90	–
4	CH_2	38.72	1.84 (ddd, *J* = 12.8, 4.7, 2.4)
			1.50 (m)
5	CH_2	36.98	2.10 (m)
			1.96 (dddd, *J* = 13.3, 13.3, 13.3, 4.8)
6	CH	67.08	3.89 (dd, *J* = 12.4, 4.7)
7	C_q	43.56	–
8	CH_2	35.55	2.12 (m)
			1.36 (m)
9	CH_2	32.81	1.82 (m)
			1.26 (m)
10	C_q	43.43	–
11	CH	48.34	1.77 (m)
12	CH_2	23.06	2.17 (m)
			1.48 (m)
13	CH_2	27.14	1.77 (m)
			1.32 (m)
14	CH	55.14	1.05 (m)
15	CH	30.69	1.52 (m)
16	CH_3	22.86	0.90 (d, *J* = 6.7)
17	CH_3	23.88	0.98 (d, *J* = 6.7)
18	CH_3	19.08	1.07 (s)
19	CH_3	18.08	0.89 (s)
20	CH_2	108.56	4.71 (s)
			4.50 (s)

[a] Carbon numbering as shown in Figure S42 indicates the origin of each carbon from GGPP by same number. [b] Chemical shifts δ in ppm, multiplicity: s = singlet, d = doublet, m = multiplet, coupling constants *J* are given in Hertz.

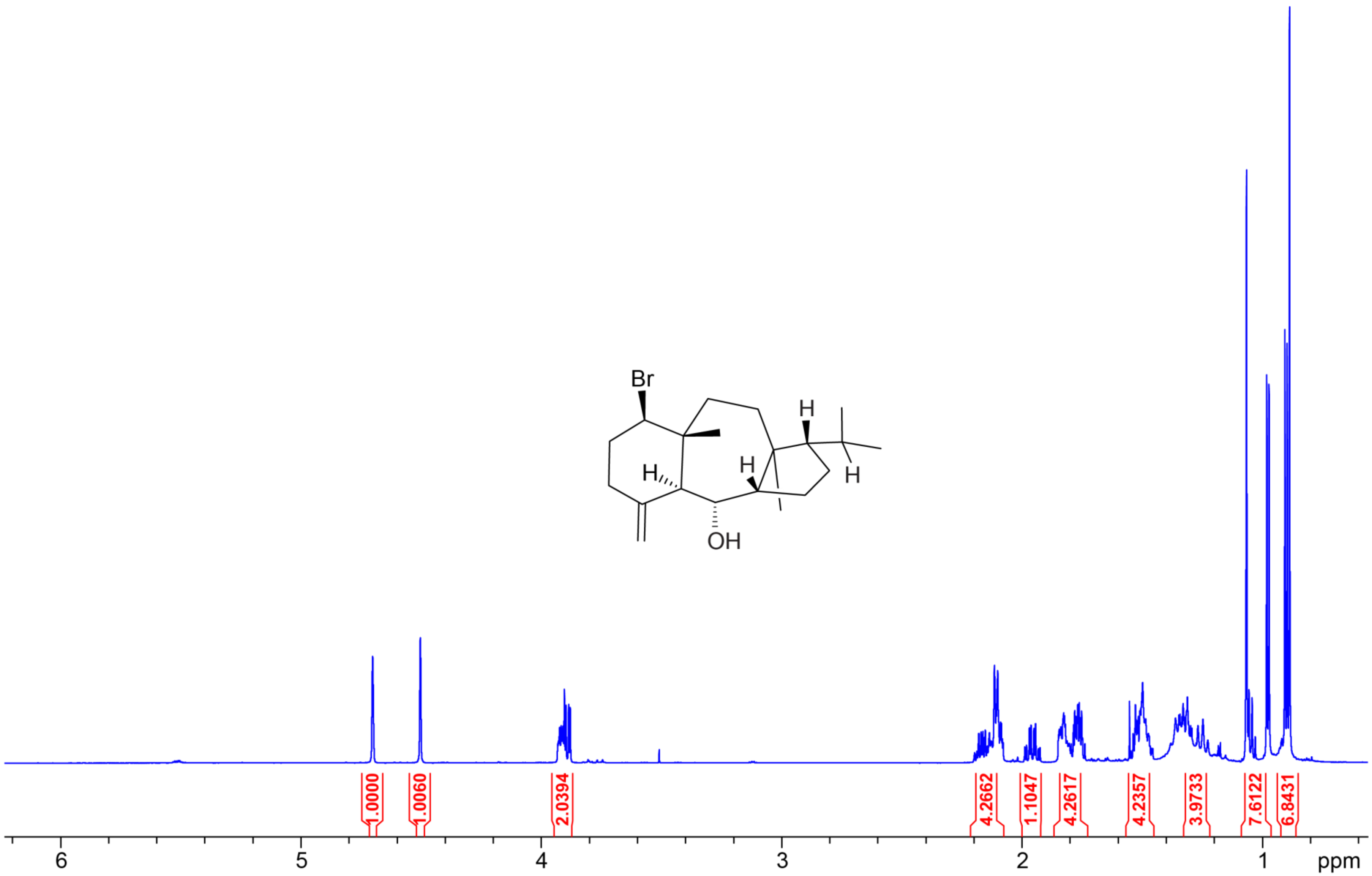

Figure S43. ^{1}H-NMR spectrum of **17** (700 MHz, C_6D_6).

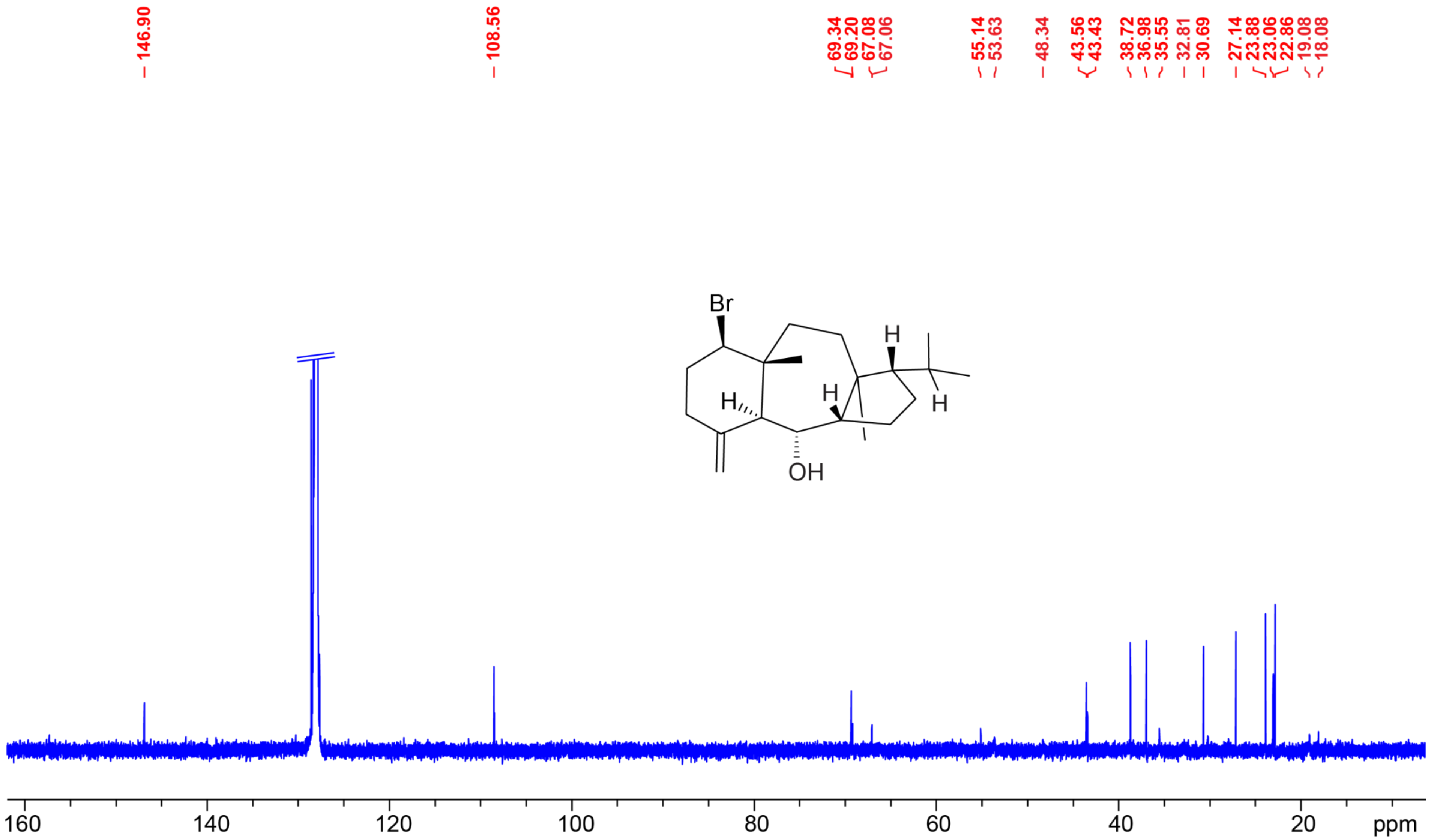

Figure S44. ^{13}C-NMR spectrum of **17** (176 MHz, C_6D_6).

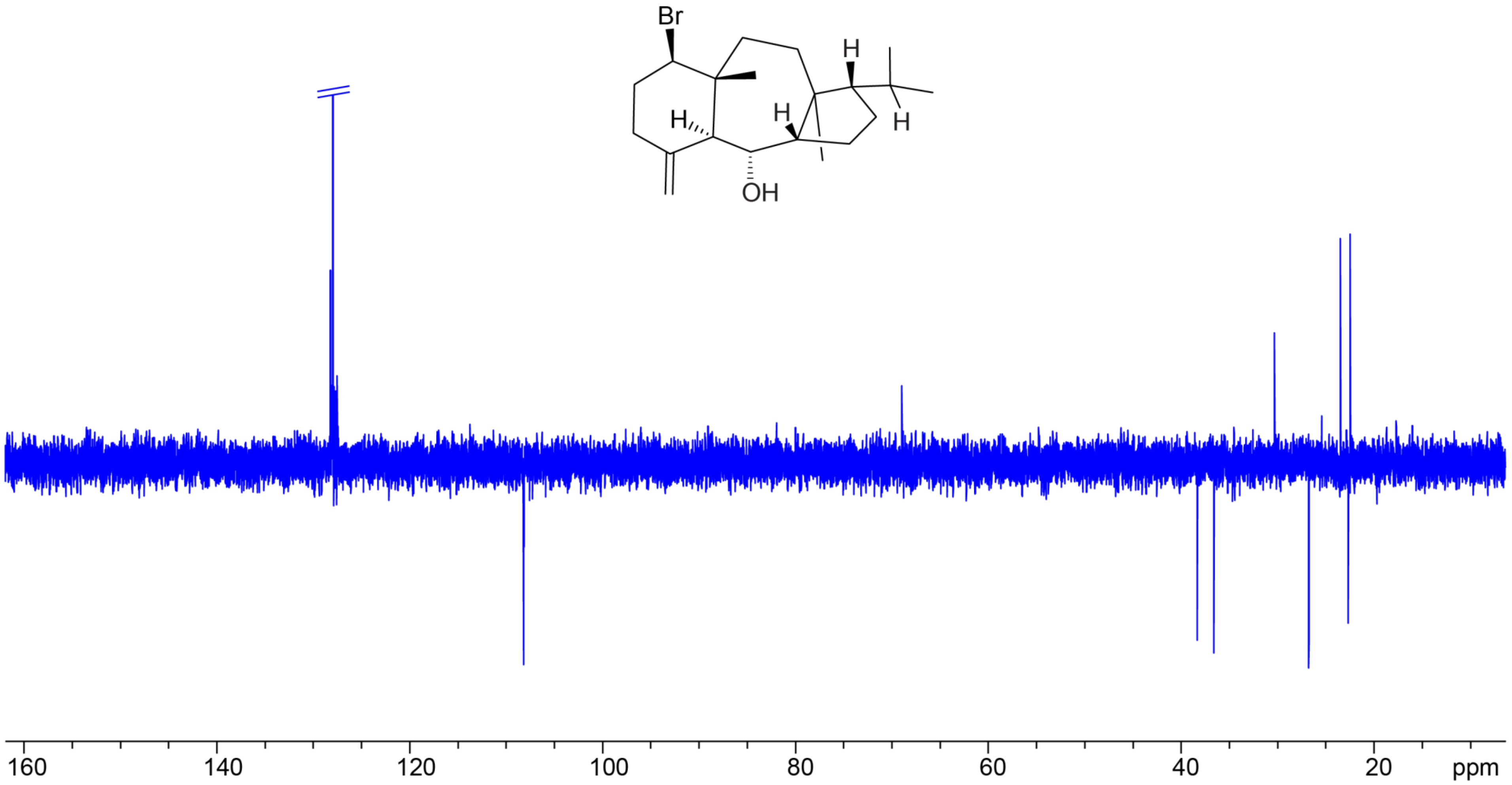

Figure S45. ^{13}C-DEPT135 spectrum of **17** (176 MHz, C_6D_6).

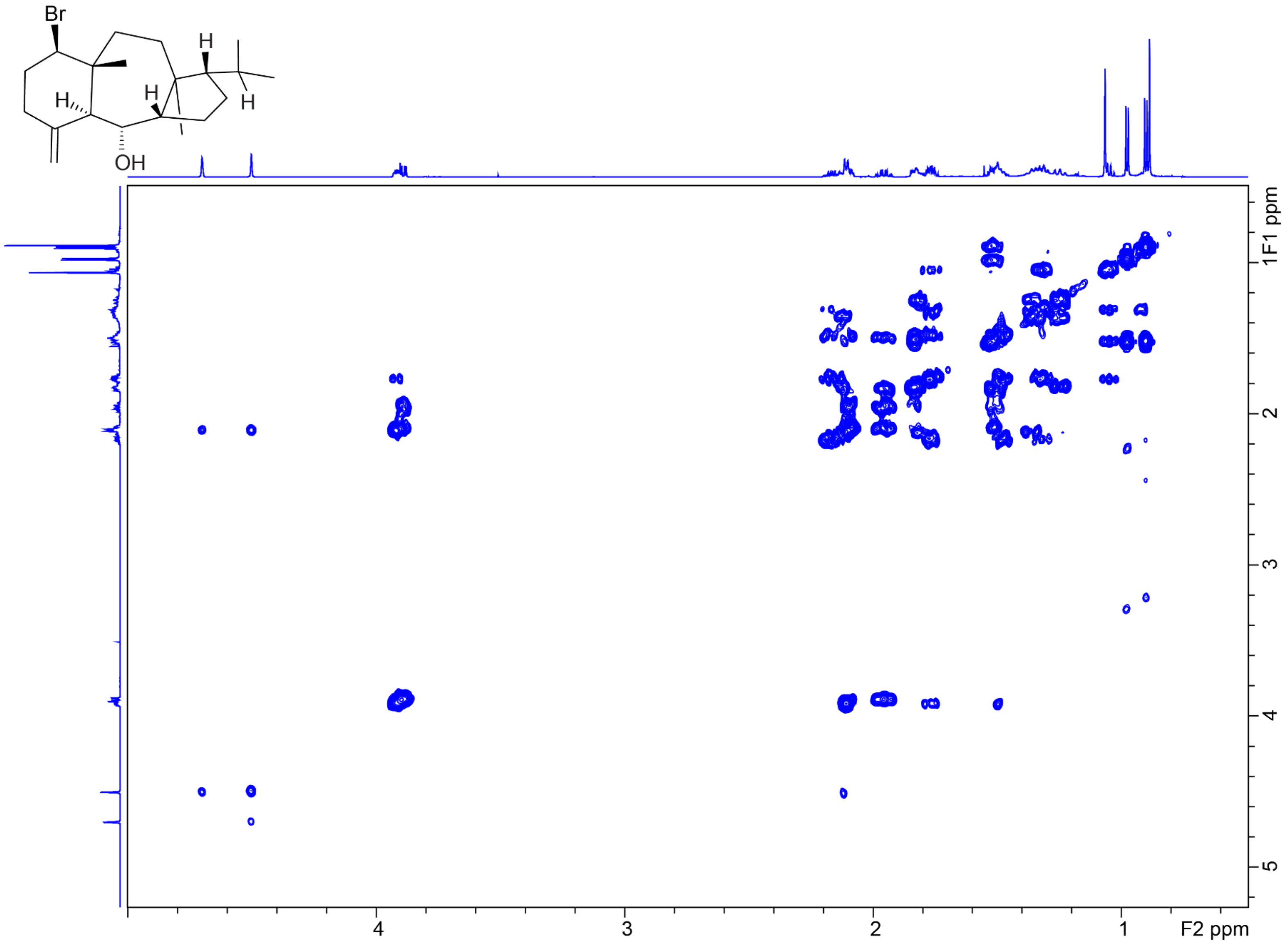

Figure S46. ^{1}H-^{1}H-COSY spectrum (C_6D_6) of **17**.

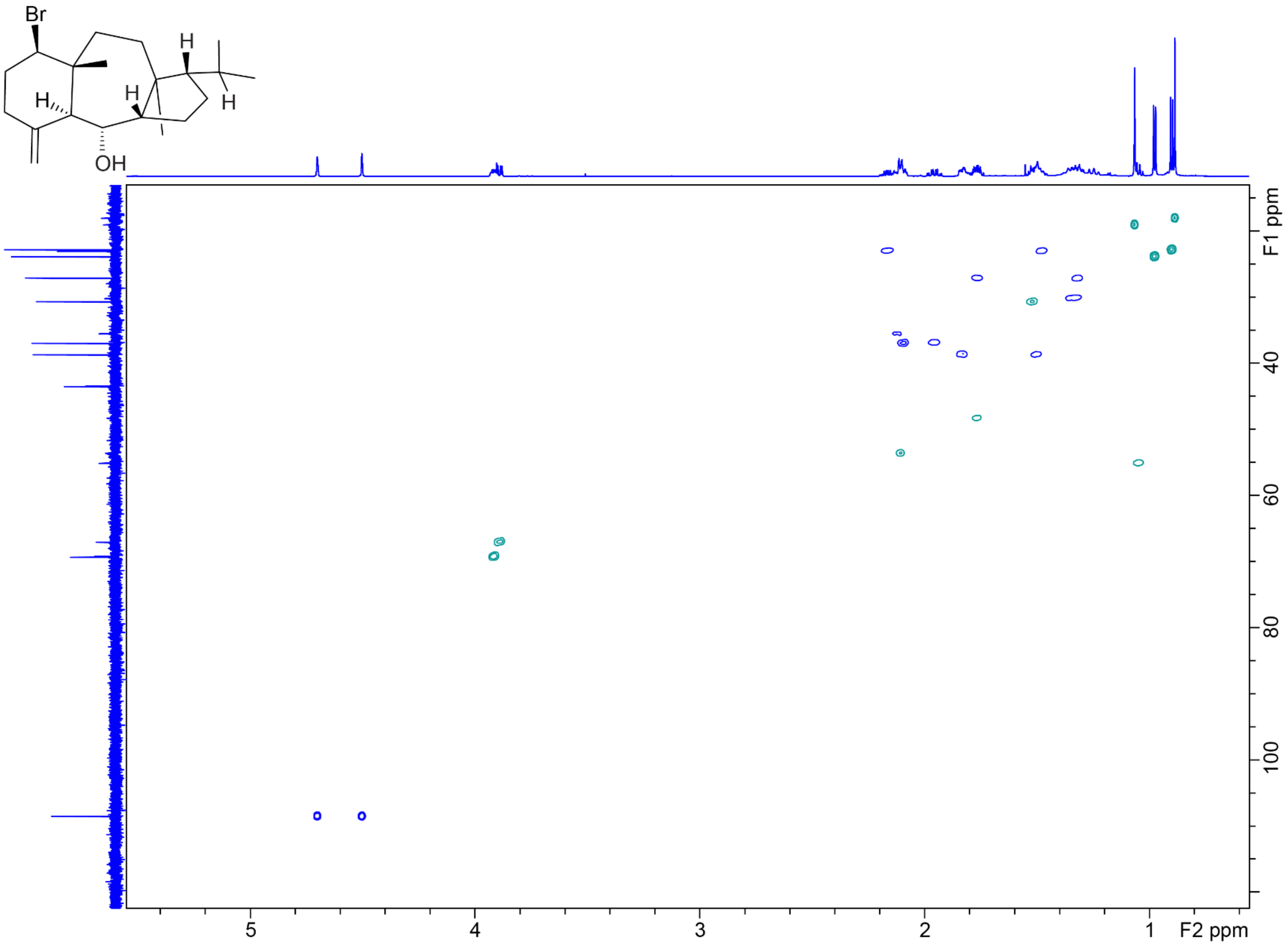

Figure S47. HSQC spectrum (C_6D_6) of **17**.

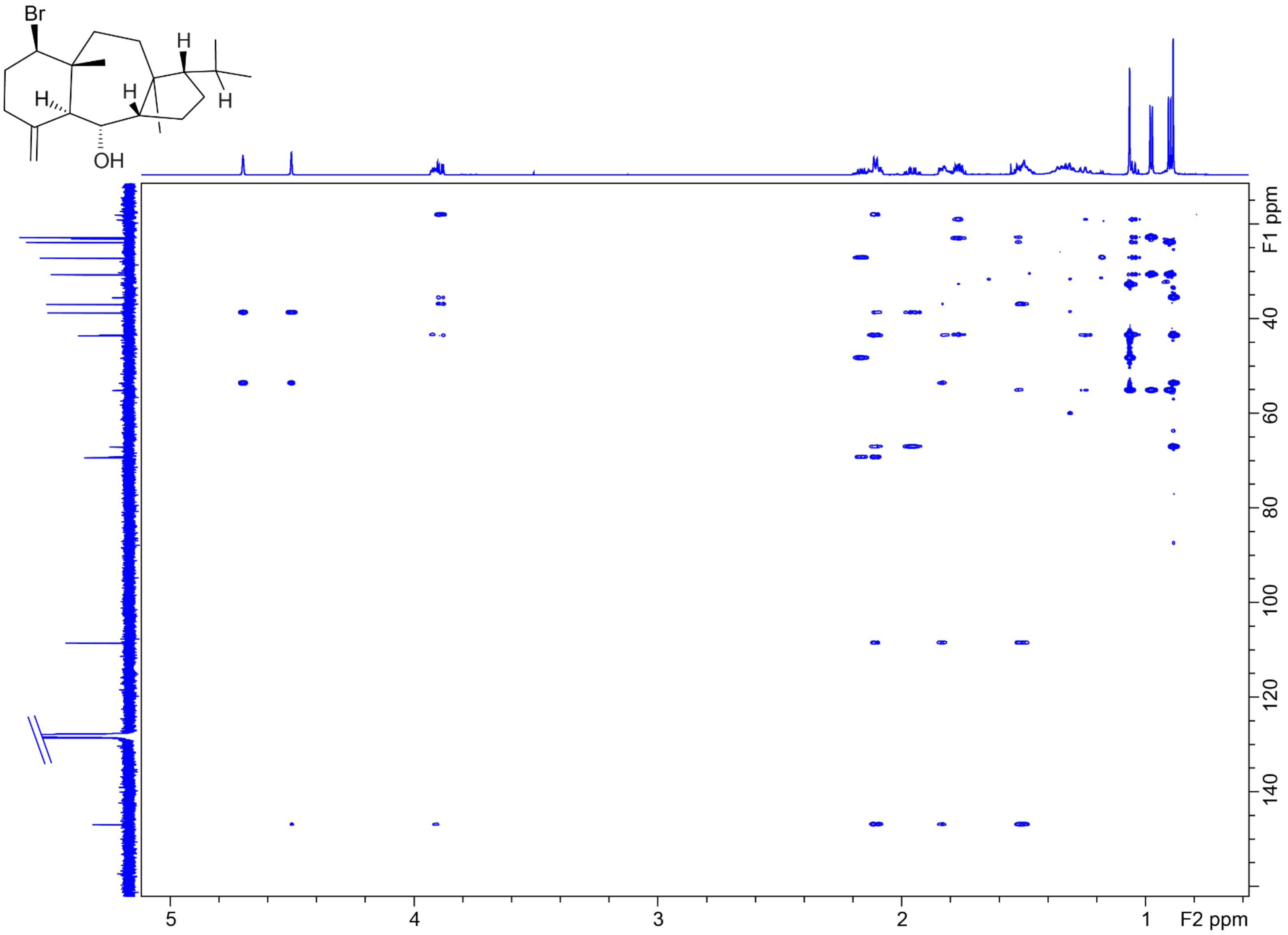

Figure S48. HMBC spectrum (C_6D_6) of **17**.

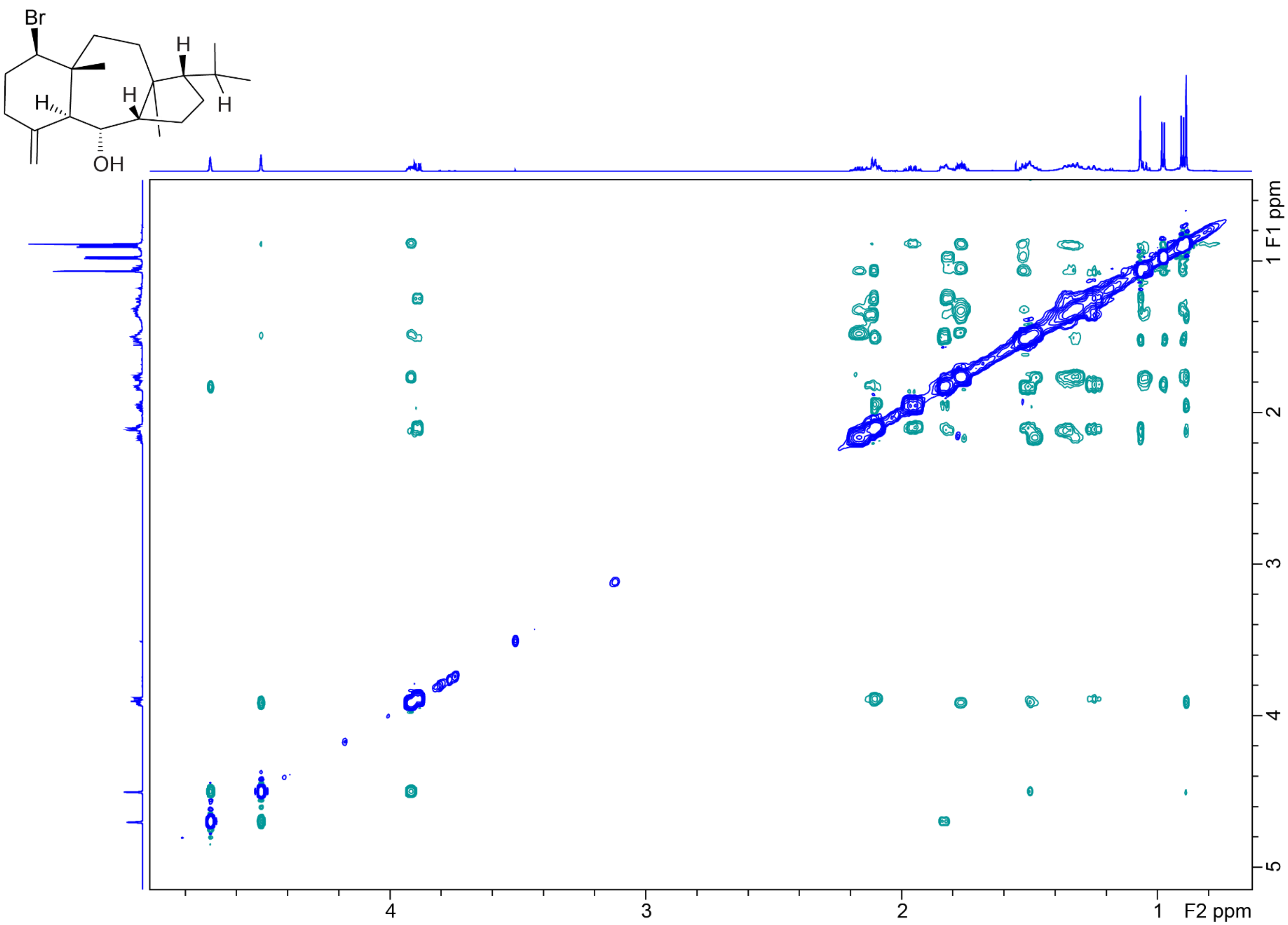

Figure S49. NOESY spectrum (C_6D_6) of **17**.

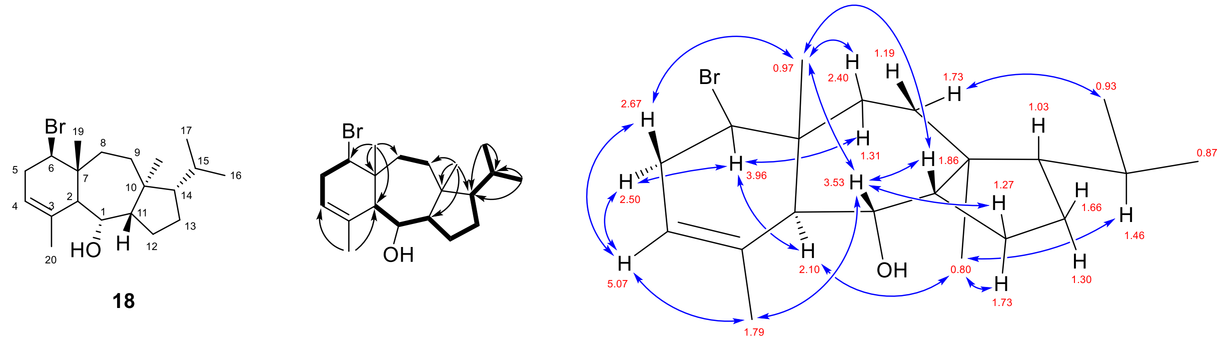

Figure S50. Structure elucidation of bromosphaerol B (**18**). Bold: ^{1}H,^{1}H-COSY, single headed arrows: key HMBC, and double headed arrows: NOESY correlations. Carbon numbering follows GGPP numbering to indicate the origin of each carbon.

Table S9. NMR data of bromosphaerol B (**18**) in C_6D_6 recorded at 298 K.

C[a]	type	^{13}C[b]	^{1}H[b]
1	CH	73.70	3.53 (m)
2	CH	57.08	2.10 (m)
3	C_q	136.74	–
4	CH	122.40	5.07 (br s)
5	CH_2	36.54	2.67 (m)<br>2.50 (m)
6	CH	68.20	3.96 (dd, *J* = 11.4, 5.8)
7	C_q	40.63	–
8	CH_2	38.73	2.40 (m)<br>1.31 (m)
9	CH_2	38.88	1.73 (m)<br>1.19 (m)
10	C_q	45.72	–
11	CH	53.44	1.86 (m)
12	CH_2	24.41	1.73 (m)<br>1.27 (m)
13	CH_2	26.33	1.66 (m)<br>1.30 (m)
14	CH	59.00	1.03 (m)
15	CH	29.37	1.46 (m)
16	CH_3	22.66	0.87 (d, *J* = 6.7)
17	CH_3	24.11	0.93 (d, *J* = 6.7)
18	CH_3	14.99	0.80 (s)
19	CH_3	11.99	0.97 (s)
20	CH_3	23.67	1.79 (s)

[a] Carbon numbering as shown in Figure S50 indicates the origin of each carbon from GGPP by same number. [b] Chemical shifts δ in ppm, multiplicity: s = singlet, d = doublet, m = multiplet, br = broad, coupling constants *J* are given in Hertz.

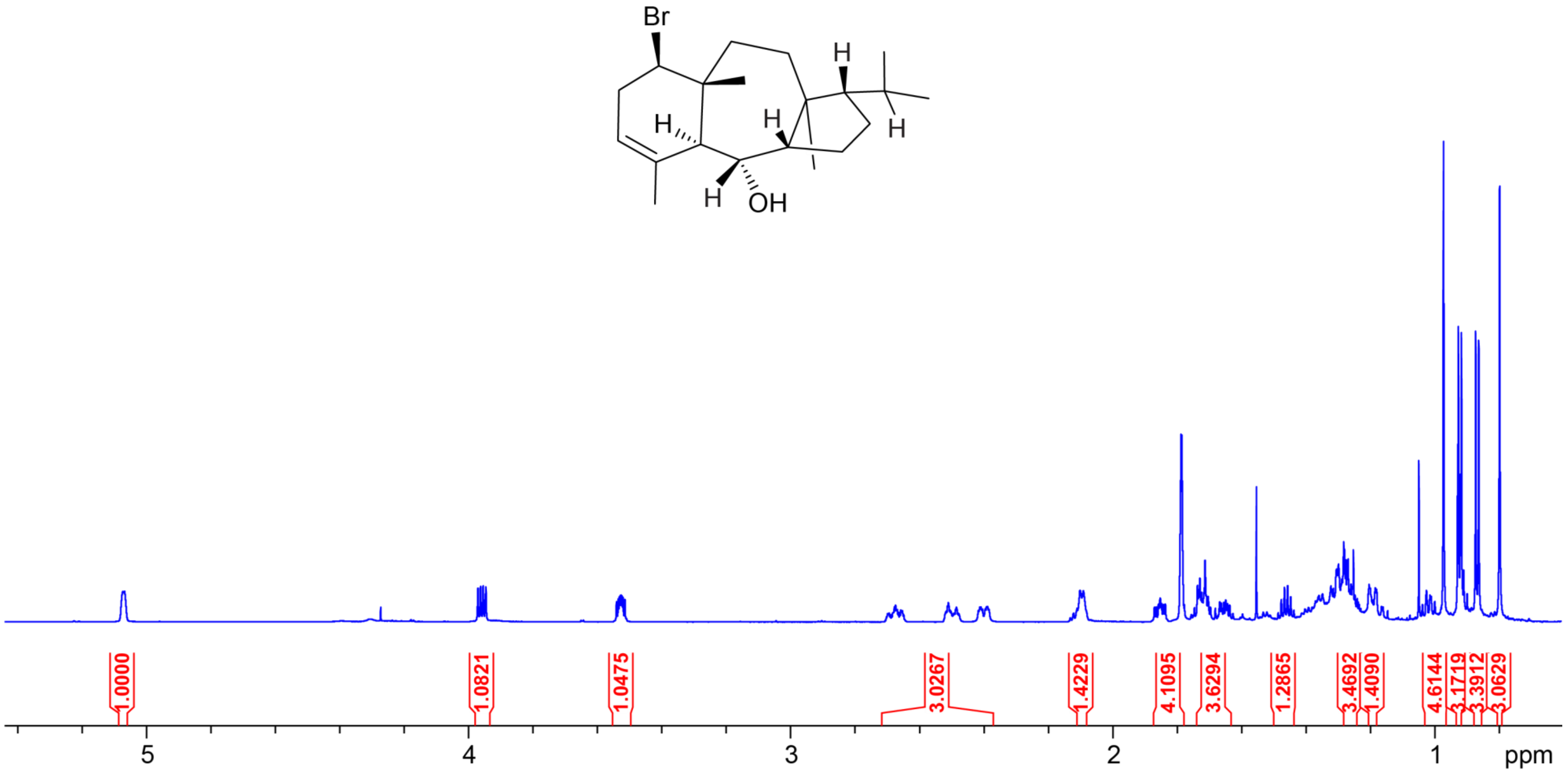

Figure S51. ^{1}H-NMR spectrum of **18** (700 MHz, C_6D_6).

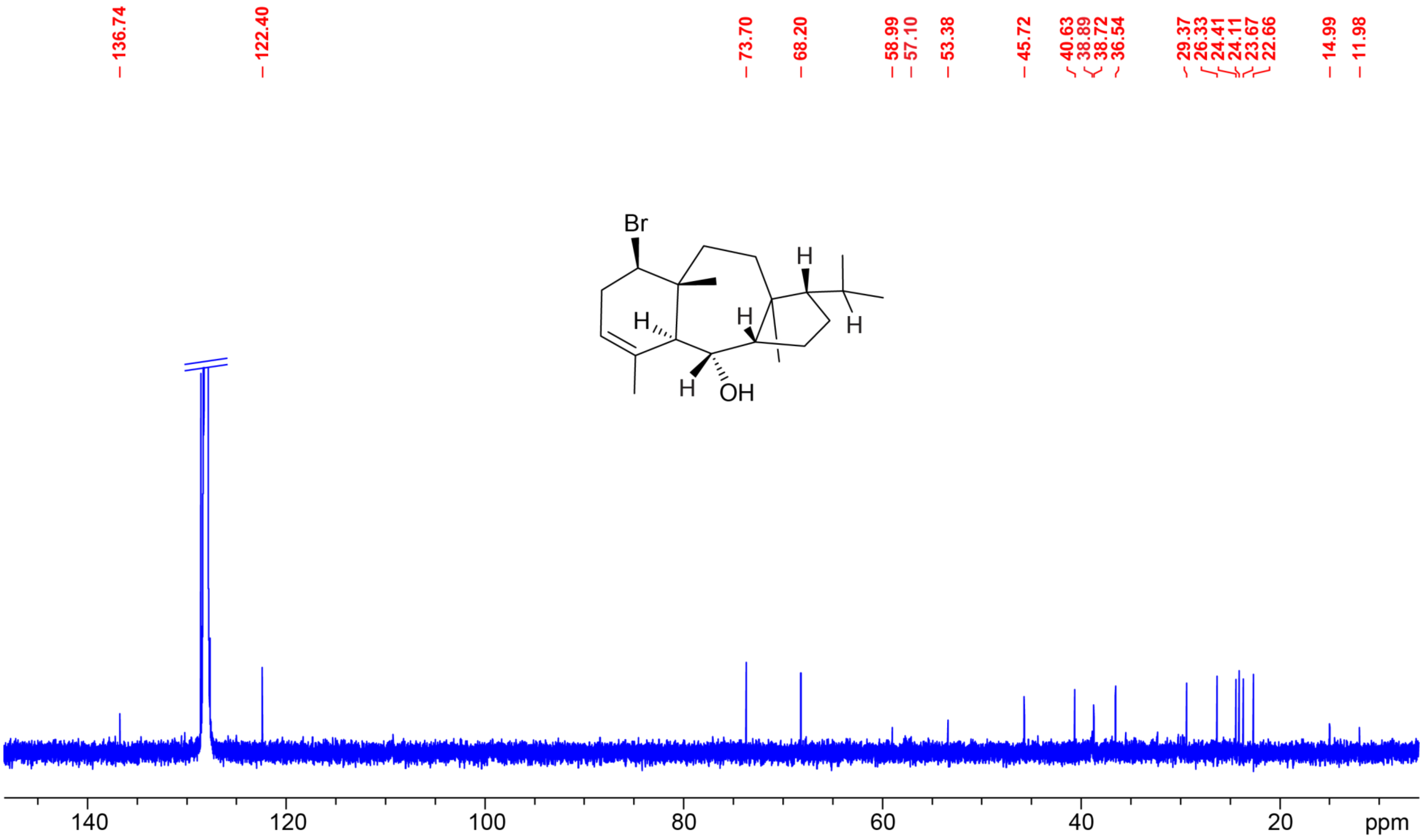

Figure S52. ^{13}C-NMR spectrum of **18** (176 MHz, C_6D_6).

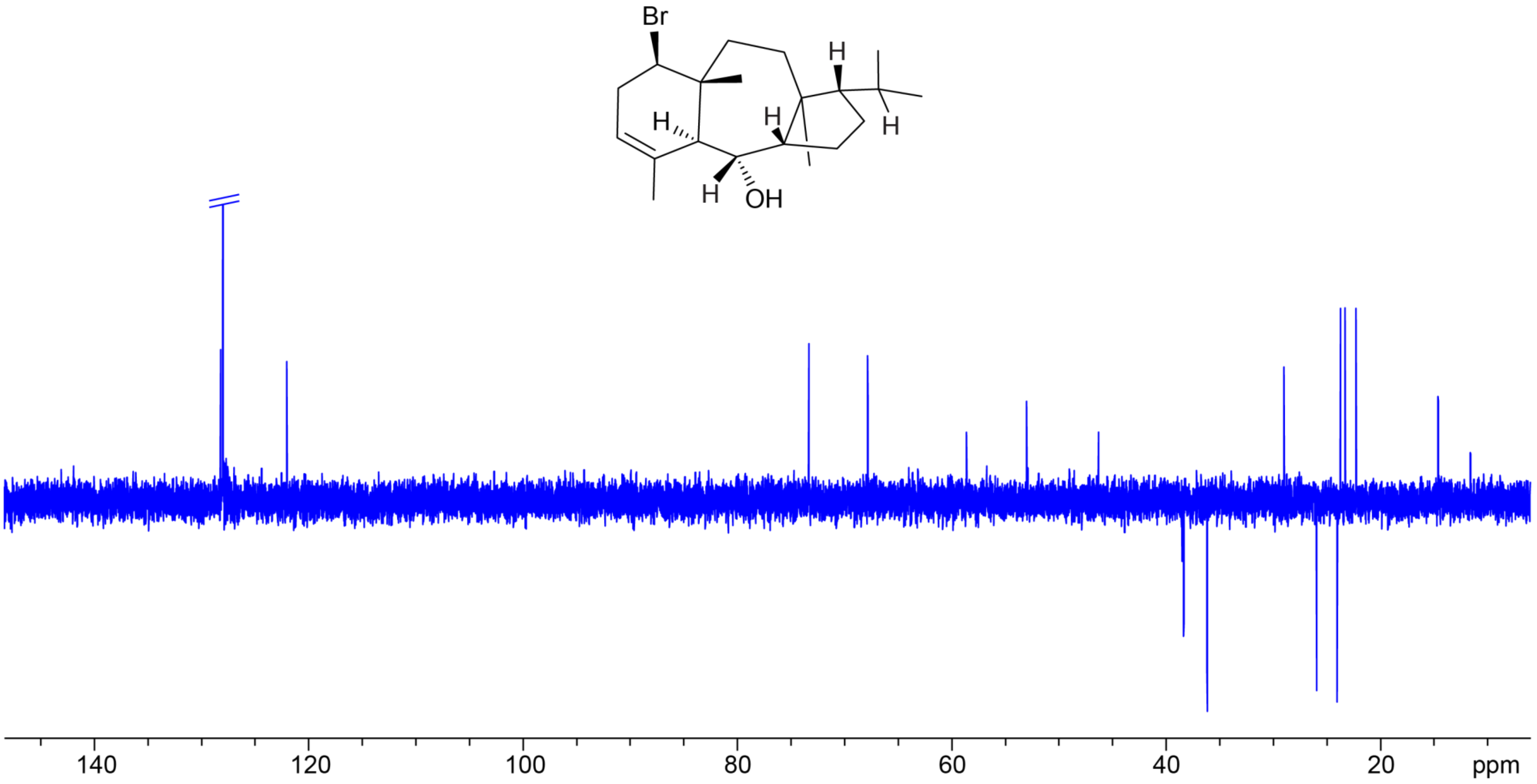

Figure S53. ^{13}C-DEPT135 spectrum of **18** (176 MHz, C_6D_6).

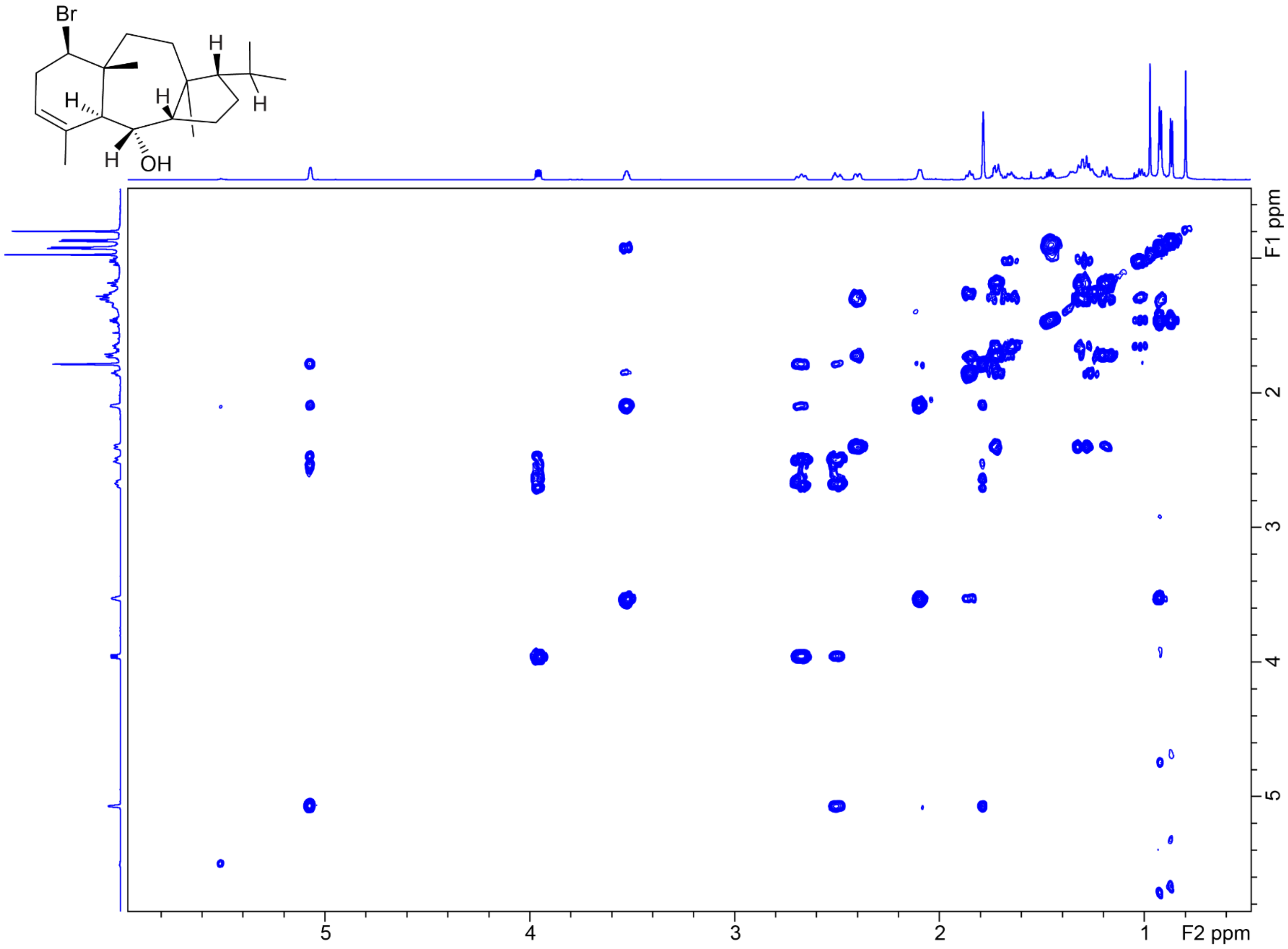

Figure S54. ^1H-^1H-COSY spectrum (C_6D_6) of **18**.

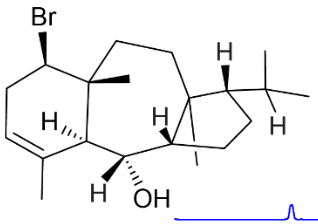

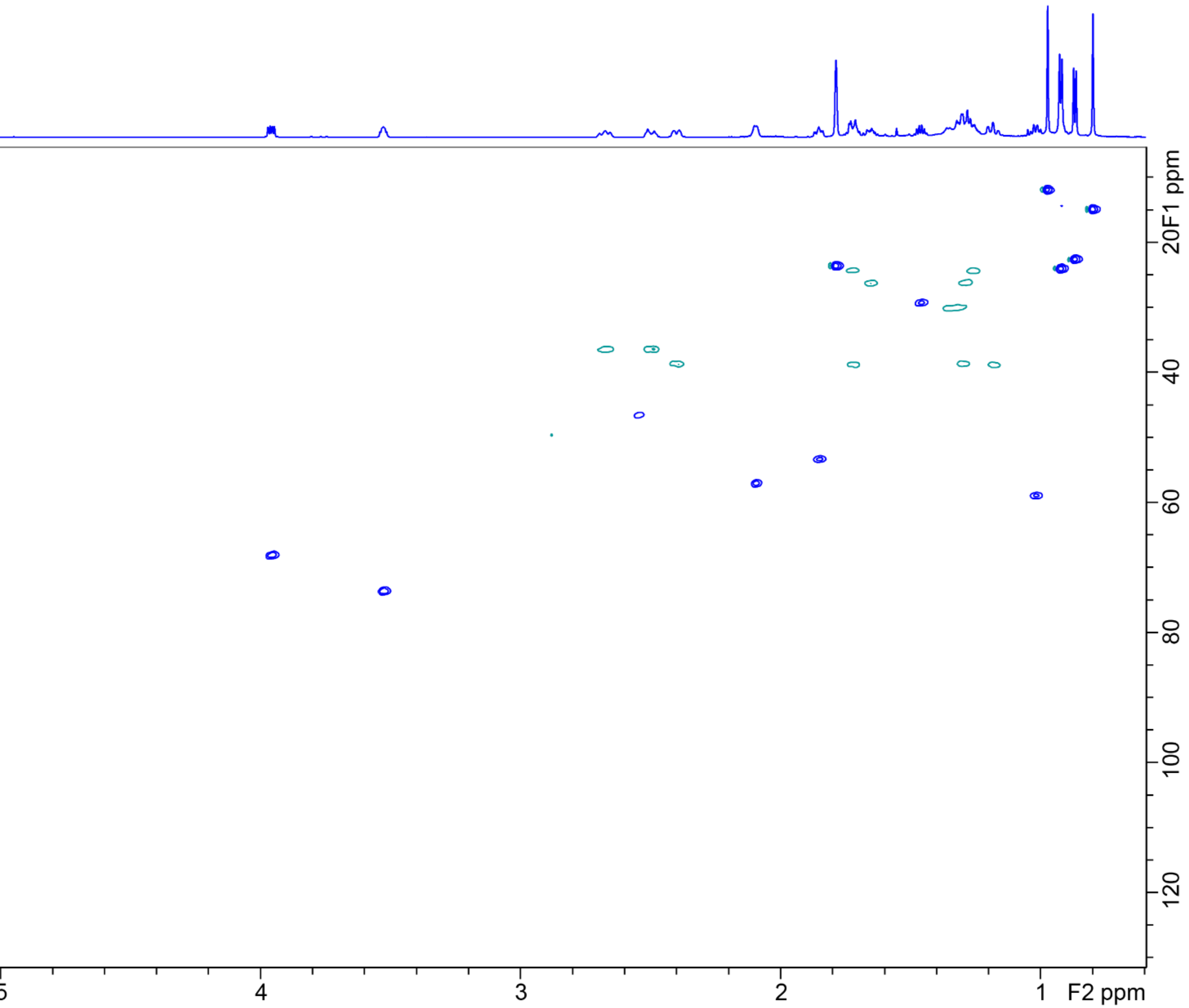

Figure S55. HSQC spectrum (C_6D_6) of **18**.

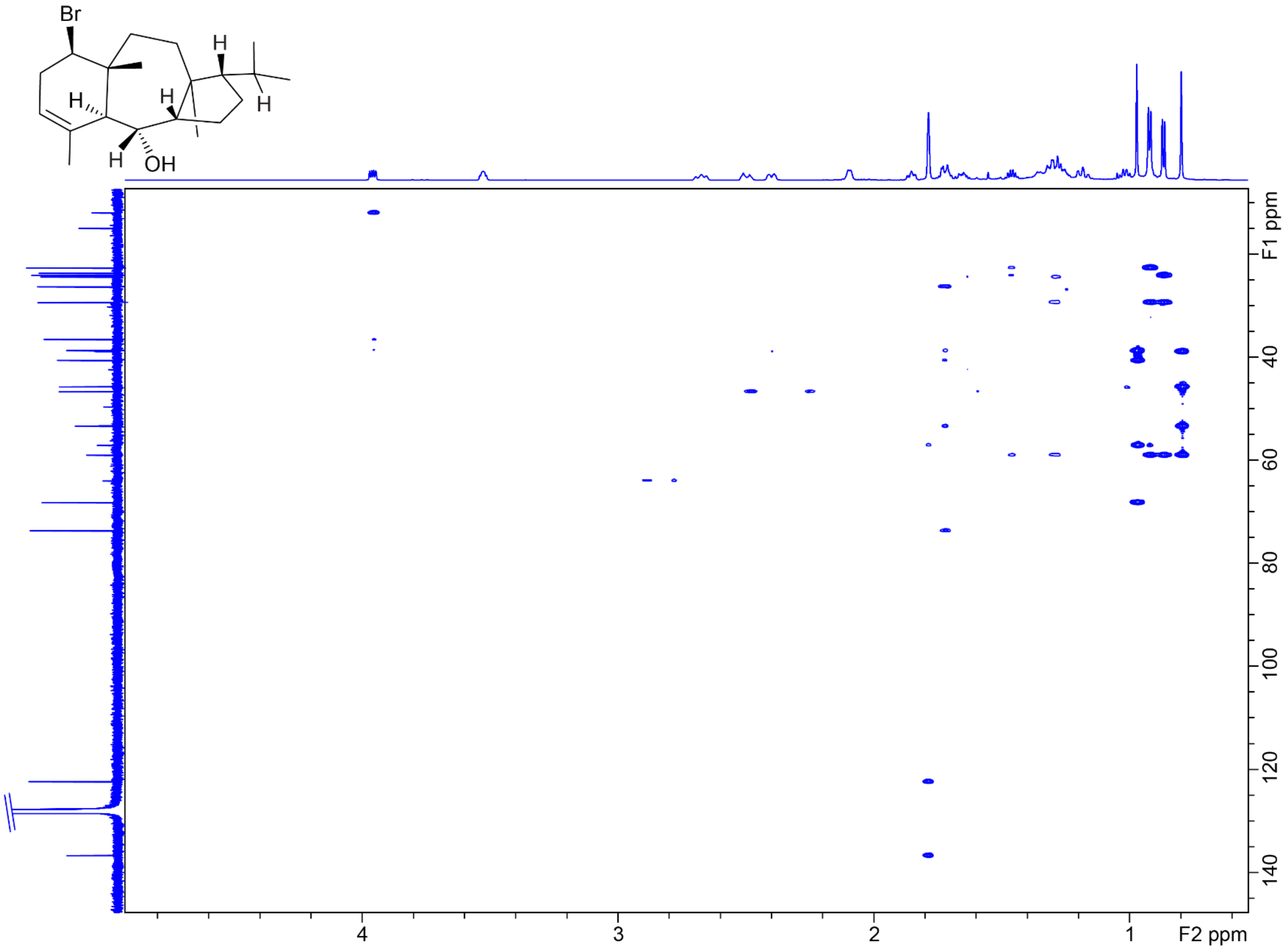

Figure S56. HMBC spectrum (C_6D_6) of **18**.

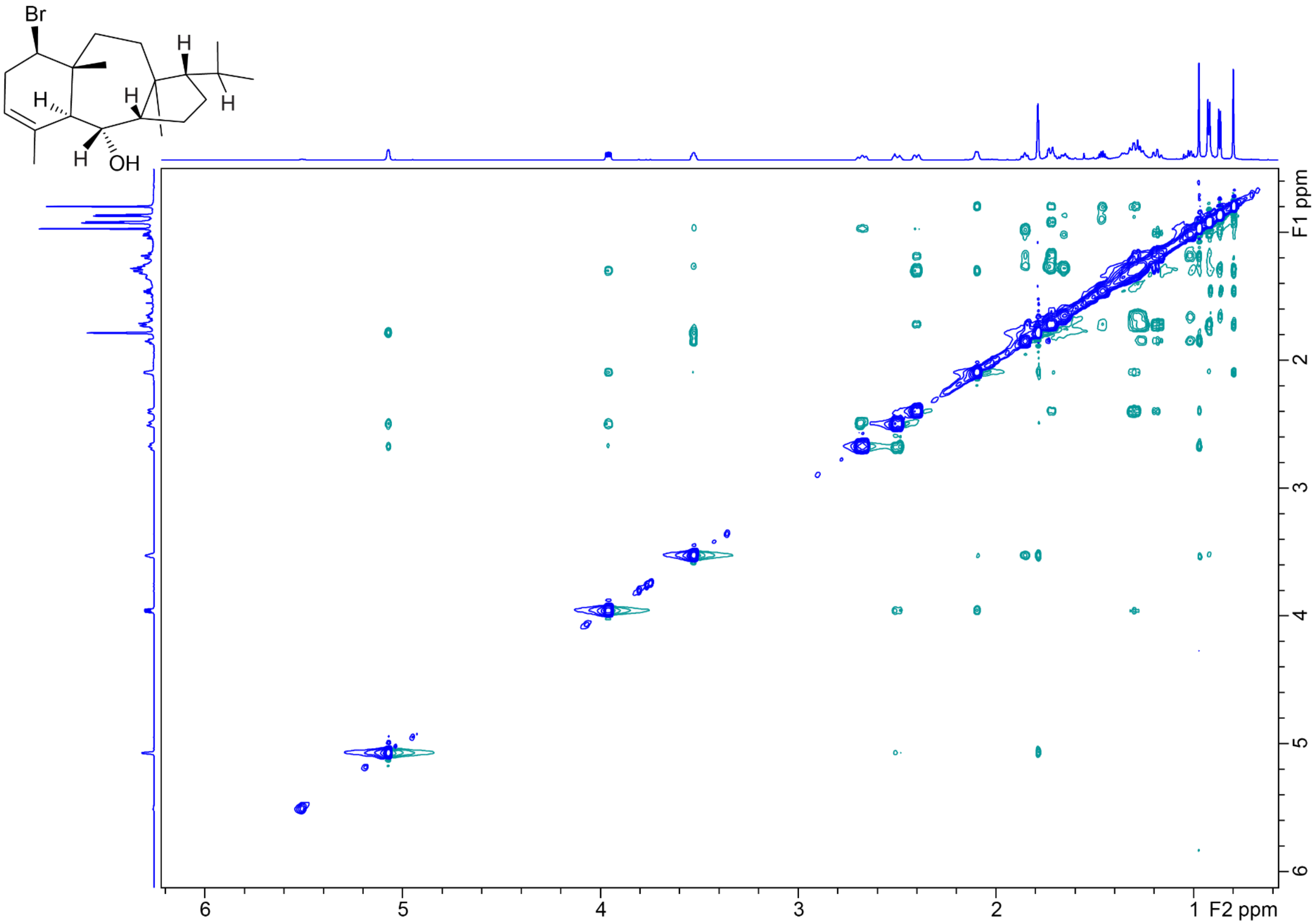

Figure S57. NOESY spectrum (C_6D_6) of **18**.

Conversion of *iso*-GGPP I with CjCsS and product isolation

A preparative scale enzymatic conversion of *iso*-GGPP I (80 mg, 160 μmol) was conducted in incubation buffer (130 mL) with the addition of an enzyme preparation of CjCsS (20 mL, 3.4 mg mL^{-1}), followed by incubation for 16 h at 30 °C. The reaction mixture was extracted with n-pentane (3 x 100 mL), the combined extracts were dried with $MgSO_4$ and the solvent was evaporated. The crude product was purified via column chromatography on silica gel to yield pure **19** (1.6 mg, 5.5 μmol, 3.4%), and **20** (0.7 mg, 2.6 μmol, 1.6%).

Isochitinosphaerol (19). TLC (pentane/Et_2O = 2:1): R_f = 0.45. GC (HP-5MS): *I* = 2276. MS (EI, 70 eV): *m*/*z* (%) = 41 (9), 55 (10), 69 (9), 81 (12), 97 (17), 109 (8), 123 (7), 135 (5), 153 (7), 163 (2), 177 (1), 187 (1), 207 (1), 220 (1), 229 (4), 247 (3), 257 (1), 275 (2), 290 (1). IR (diamond ATR): ṽ = 3417 (br), 3066 (w), 2930 (s), 2869 (m), 1641 (w), 1447 (w), 1383 (w), 1260 (w), 1077 (w), 1020 (w), 889 (w), 847 (w), 814 (w), 684 (w) cm^{-1}. HR-MS (Q-TOF, 70 eV): calc. $[C_{20}H_{34}O]^+$ *m*/*z* = 290.2604; found: *m*/*z* = 290.2611. Optical rotation: $[\alpha]_D^{25}$ = –93.6 (*c* 0.14, CH_2Cl_2). NMR data are given in Table S10.

Chitinoxenene (20). TLC (pentane): R_f = 0.62. GC (HP-5MS): *I* = 1959. MS (EI, 70 eV): *m*/*z* (%) = 41 (9), 55 (4), 69 (12), 79 (10), 93 (12), 105 (10), 119 (6), 133 (10), 149 (13), 161 (3), 173 (1), 187 (2), 203 (1), 216 (1), 229 (2), 257 (1), 272 (3). IR (diamond ATR): ṽ = 3363 (br), 2951 (m), 2924 (s), 2853 (m), 1657 (w), 1632 (w), 1463 (w), 1377 (w), 1259 (w), 1092 (w), 1016 (w), 873 (w), 796 (w) cm^{-1}. HR-MS (Q-TOF, 70 eV): calc. $[C_{20}H_{32}]^+$ *m*/*z* = 272.2499; found: *m*/*z* = 272.2496. Optical rotation: $[\alpha]_D^{25}$ = +88.6 (*c* 0.07, CH_2Cl_2). NMR data are given in Table S11.

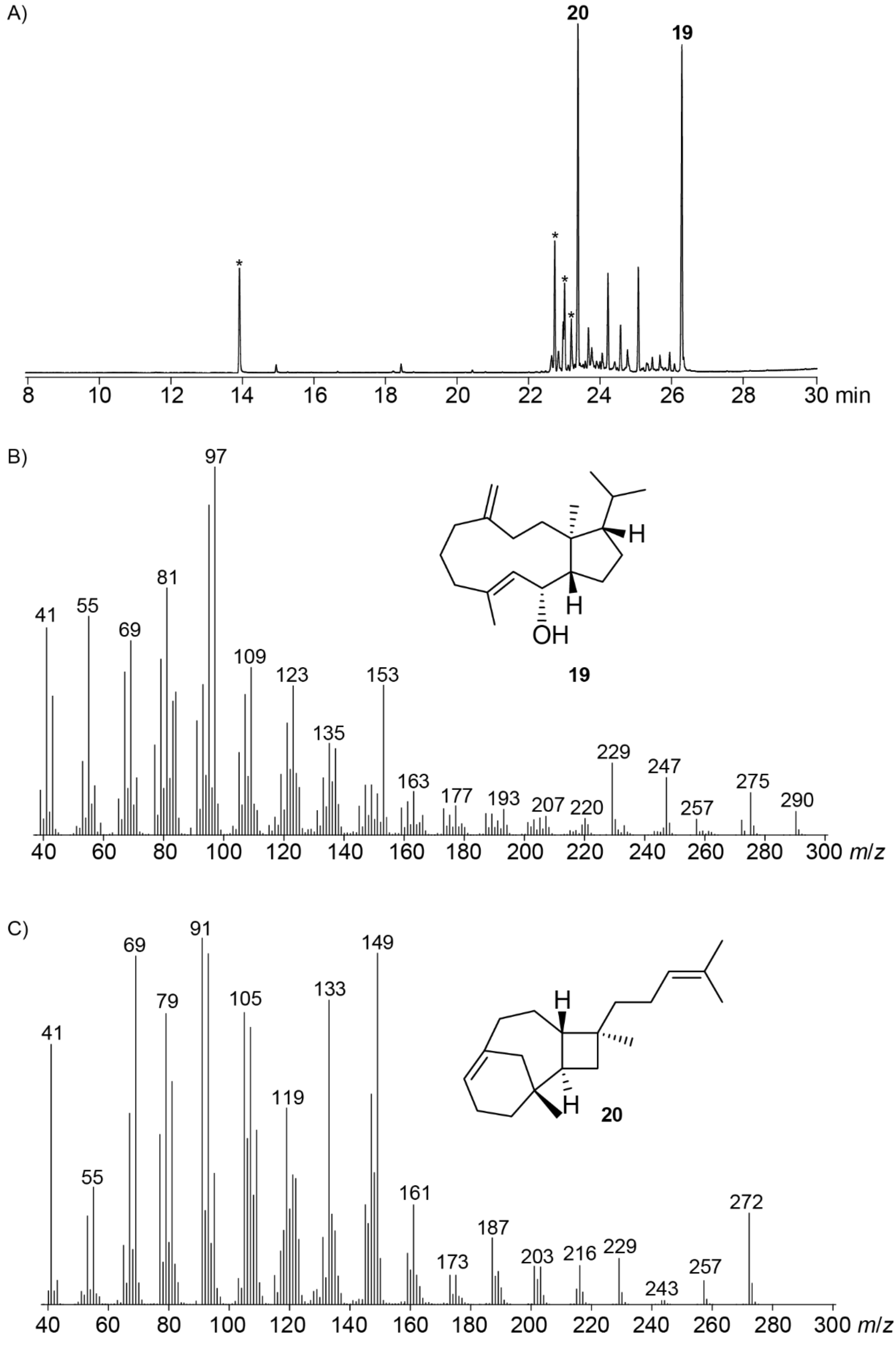

Figure S58. GC/MS analysis of the products obtained from *iso*-GGPP I with CjCsS. A) Total ion chromatogram of the enzyme products showing two major products (asterisks indicate spontaneous hydrolysis products and contaminants). EI mass spectra of B) isochitinosphaerol (**19**) and C) chitinoxenene (**20**).

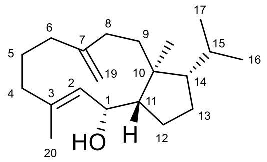

19

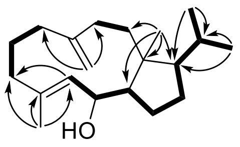

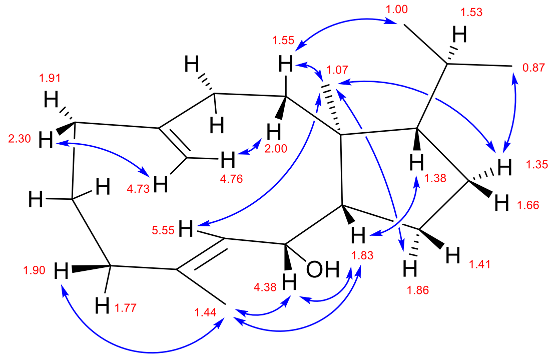

Figure S59. Structure elucidation of isochitinosphaerol (**19**). Bold: ^{1}H,^{1}H-COSY, single headed arrows: key HMBC, and double headed arrows: NOESY correlations. Carbon numbering follows GGPP numbering to indicate the origin of each carbon.

Table S10. NMR data of isochitinosphaerol (**19**) in C_6D_6 recorded at 298 K.

C[a]	type	^{13}C[b]	^{1}H[b]
1	CH	71.38	4.38 (m)
2	CH	131.14	5.55 (d, *J* = 7.0)
3	C_q	132.04	–
4	CH_2	42.39	1.90 (m)
			1.77 (m)
5	CH_2	26.76	1.78 (m)
			1.18 (m)
6	CH_2	34.06	2.30 (ddd, *J* = 14.2, 11.4, 2.9)
7			
8	CH_2	28.82	1.95 (m)
			1.75 (m)
9	CH_2	37.82	2.00 (m)
			1.55 (m)
10	C_q	46.43	–
11	CH	48.69	1.83 (m)
12	CH_2	24.18	1.86 (m)
			1.41 (m)
13	CH_2	25.45	1.66 (m)
			1.35 (m)
14	CH	51.21	1.38 (m)
15	CH	28.90	1.53 (m)
16	CH_3	23.34	1.00 (d, *J* = 6.7)
17	CH_3	22.60	0.87 (d, *J* = 6.7)
18	CH_3	19.70	1.07 (s)
19	CH_2	110.60	4.76 (dd, *J* = 2.4, 1.1)
			4.73 (br d, *J* = 2.4)
20	CH_3	16.45	1.44 (s)

[a] Carbon numbering as shown in Figure S59 indicates the origin of each carbon from GGPP by same number. [b] Chemical shifts δ in ppm, multiplicity: s = singlet, d = doublet, m = multiplet, br = broad, coupling constants *J* are given in Hertz.

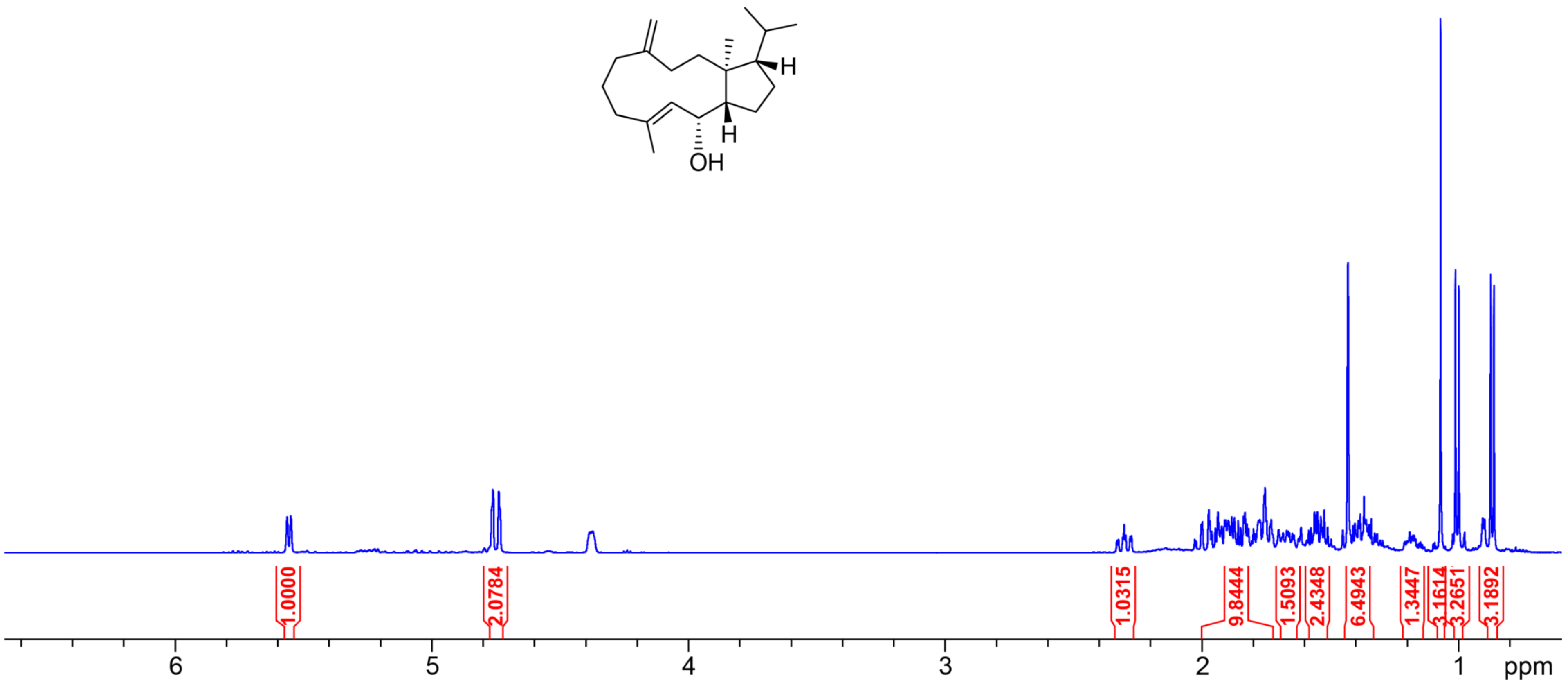

Figure S60. ^{1}H-NMR spectrum of **19** (500 MHz, C_6D_6).

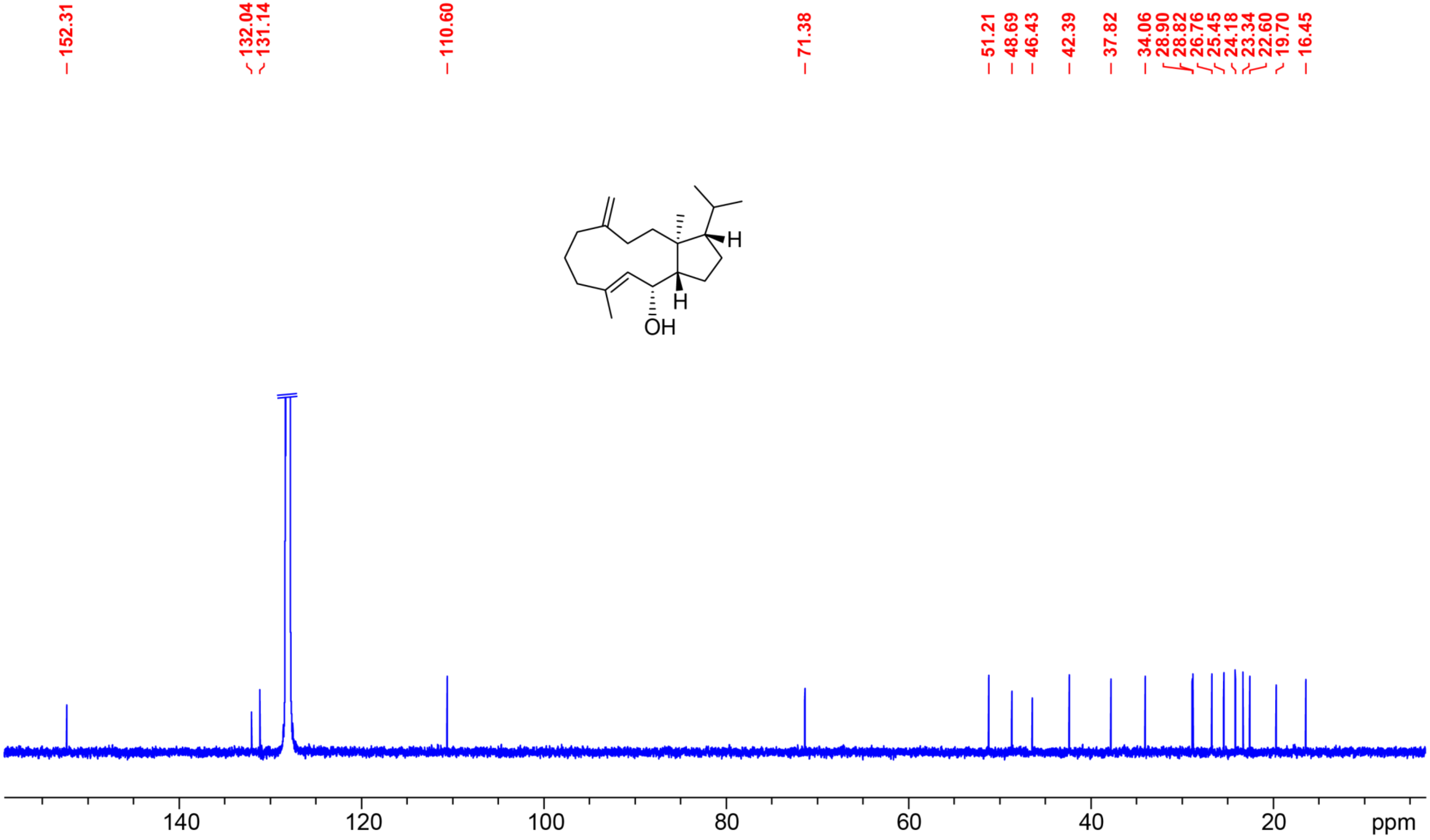

Figure S61. ^{13}C-NMR spectrum of **19** (126 MHz, C_6D_6).

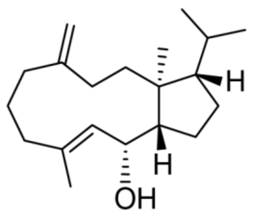

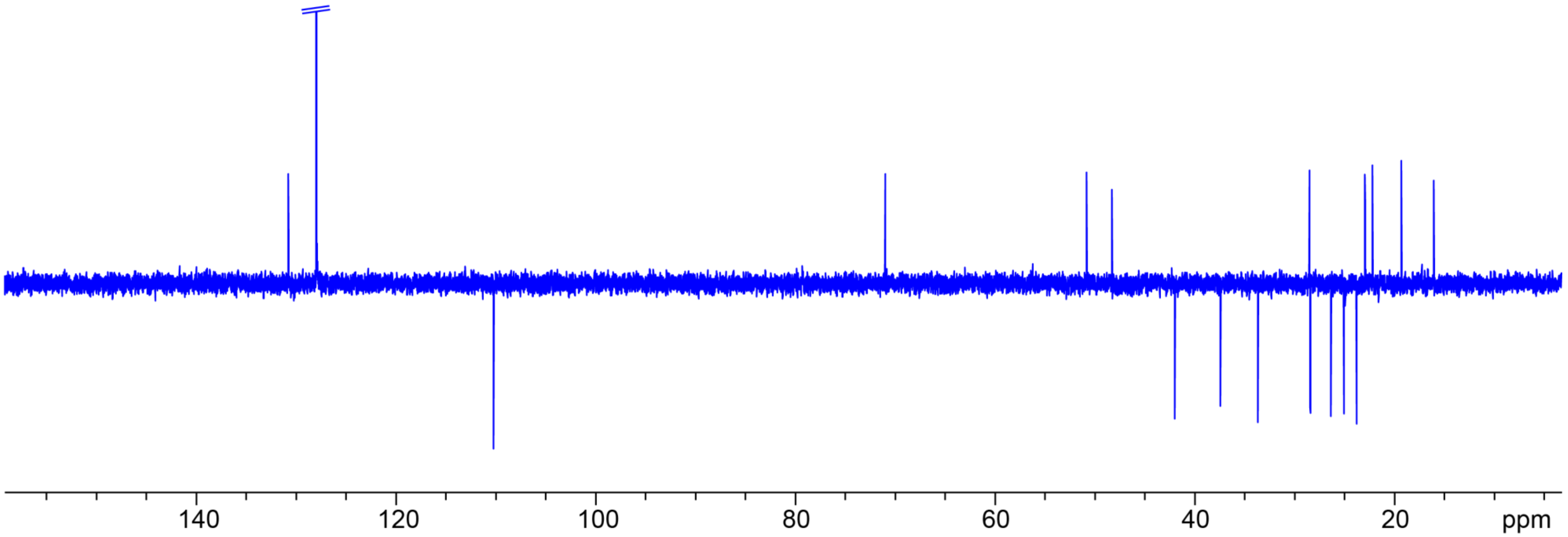

Figure S62. ^{13}C-DEPT135 spectrum of **19** (126 MHz, C_6D_6).

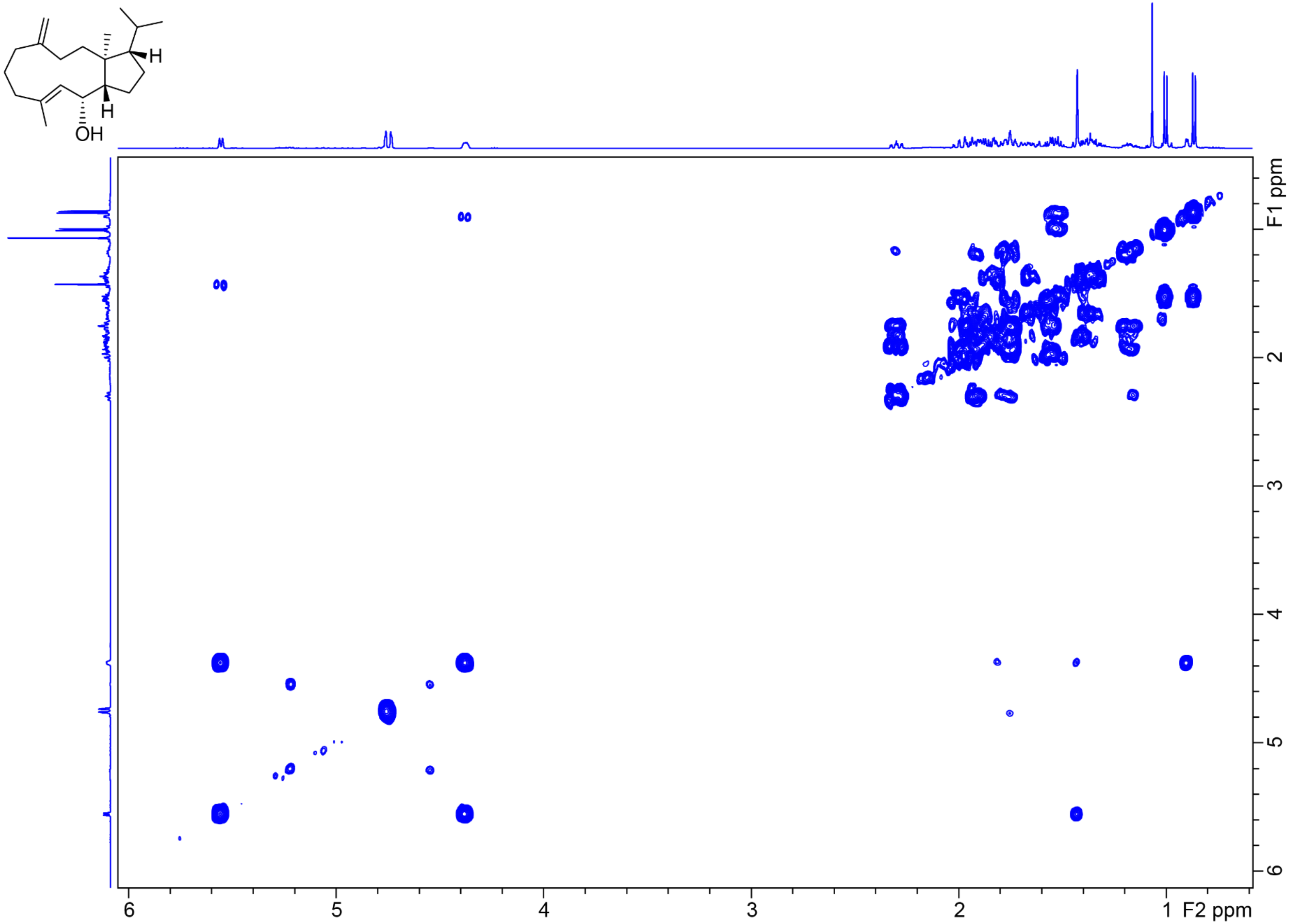

Figure S63. ^{1}H-^{1}H-COSY spectrum (C_6D_6) of **19**.

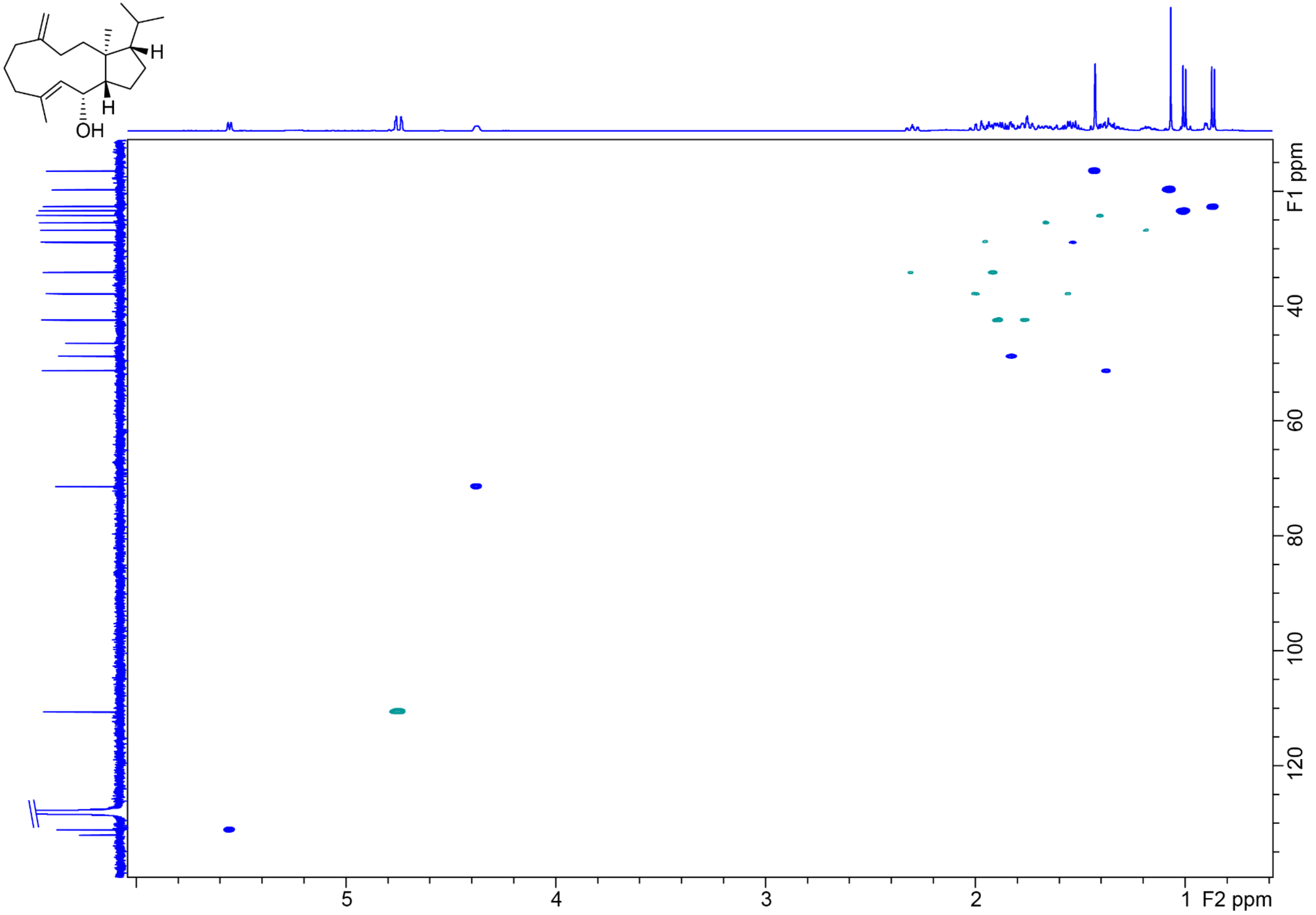

Figure S64. HSQC spectrum (C_6D_6) of **19**.

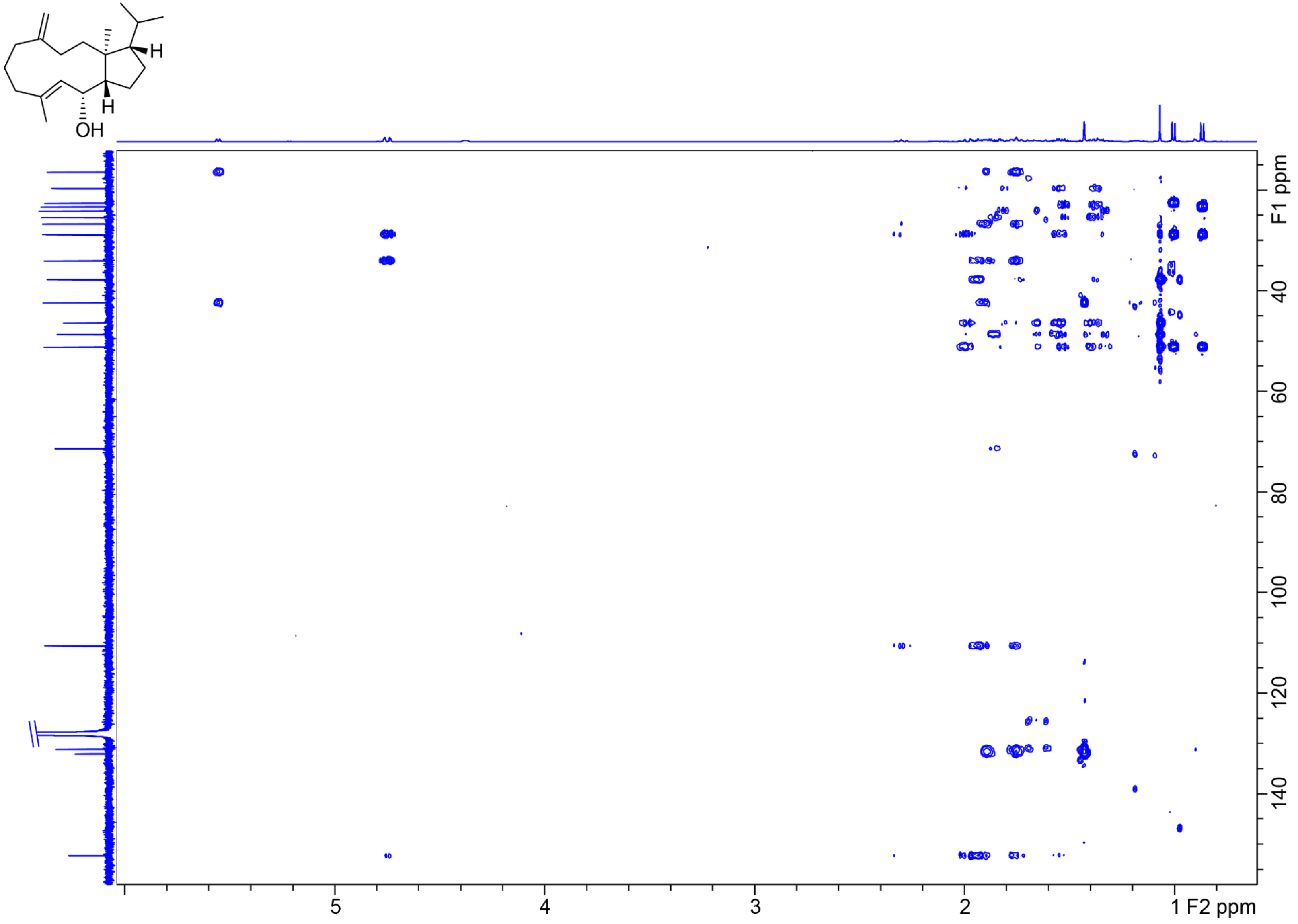

Figure S65. HMBC spectrum (C_6D_6) of **19**.

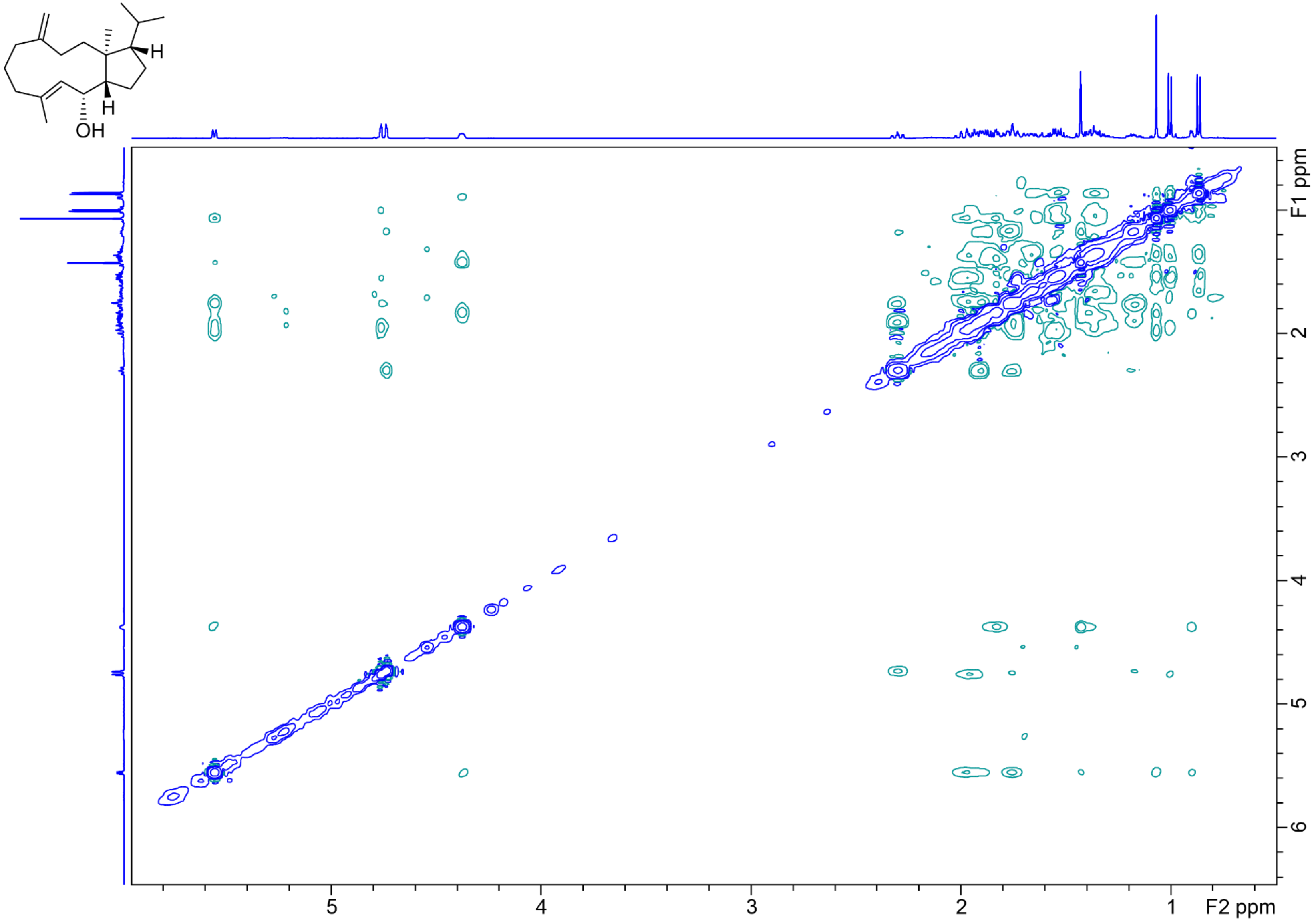

Figure S66. NOESY spectrum (C_6D_6) of **19**.

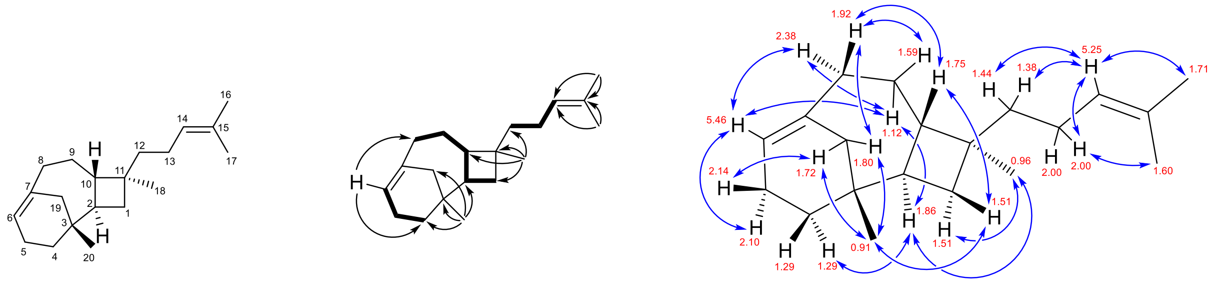

20

Figure S67. Structure elucidation of chitinoxenene (**20**). Bold: $^{1}H,^{1}H$-COSY, single headed arrows: key HMBC, and double headed arrows: NOESY correlations. Carbon numbering follows GGPP numbering to indicate the origin of each carbon.

Table S11. NMR data of chitinoxenene (**20**) in C_6D_6 recorded at 298 K.

C[a]	type	^{13}C[b]	^{1}H[b]
1	CH_2	35.49	1.51 (m, 2H)
2	CH	47.22	1.86 (m)
3	C_q	33.35	–
4	CH_2	36.88	1.29 (m, 2H)
5	CH_2	22.96	2.14 (m)
			2.10 (m)
6	CH	120.41	5.46 (m)
7	C_q	141.50	–
8	CH_2	35.41	2.38 (dd, *J* = 12.0, 7.1)
			1.92 (ddd, *J* = 12.0, 11.2, 5.9)
9	CH_2	32.14	1.59 (m)
			1.12 (dddd, *J* = 12.1, 12.1, 12.1, 7.0)
10	CH	47.13	1.75 (m)
11	C_q	36.39	–
12	CH_2	45.39	1.44 (ddd, *J* = 13.1, 9.1, 7.6)
			1.38 (ddd, *J* = 13.1, 9.1 ,7.9)
13	CH_2	23.71	2.00 (m, 2H)
14	CH	125.82	5.25 (thept, *J* = 7.0, 1.4)
15	C_q	130.70	–
16	CH_3	25.92	1.71 (d, *J* = 0.8)
17	CH_3	17.70	1.60 (s)
18	CH_3	19.23	0.96 (s)
19	CH_2	43.93	1.80 (ddd, *J* = 12.2, 1.4, 1.4)
			1.72 (m)
20	CH_3	25.71	0.91 (s)

[a] Carbon numbering as shown in Figure S67 indicates the origin of each carbon from GGPP by same number. [b] Chemical shifts δ in ppm, multiplicity: s = singlet, d = doublet, t = triplet, hept = heptet, m = multiplet, coupling constants *J* are given in Hertz.

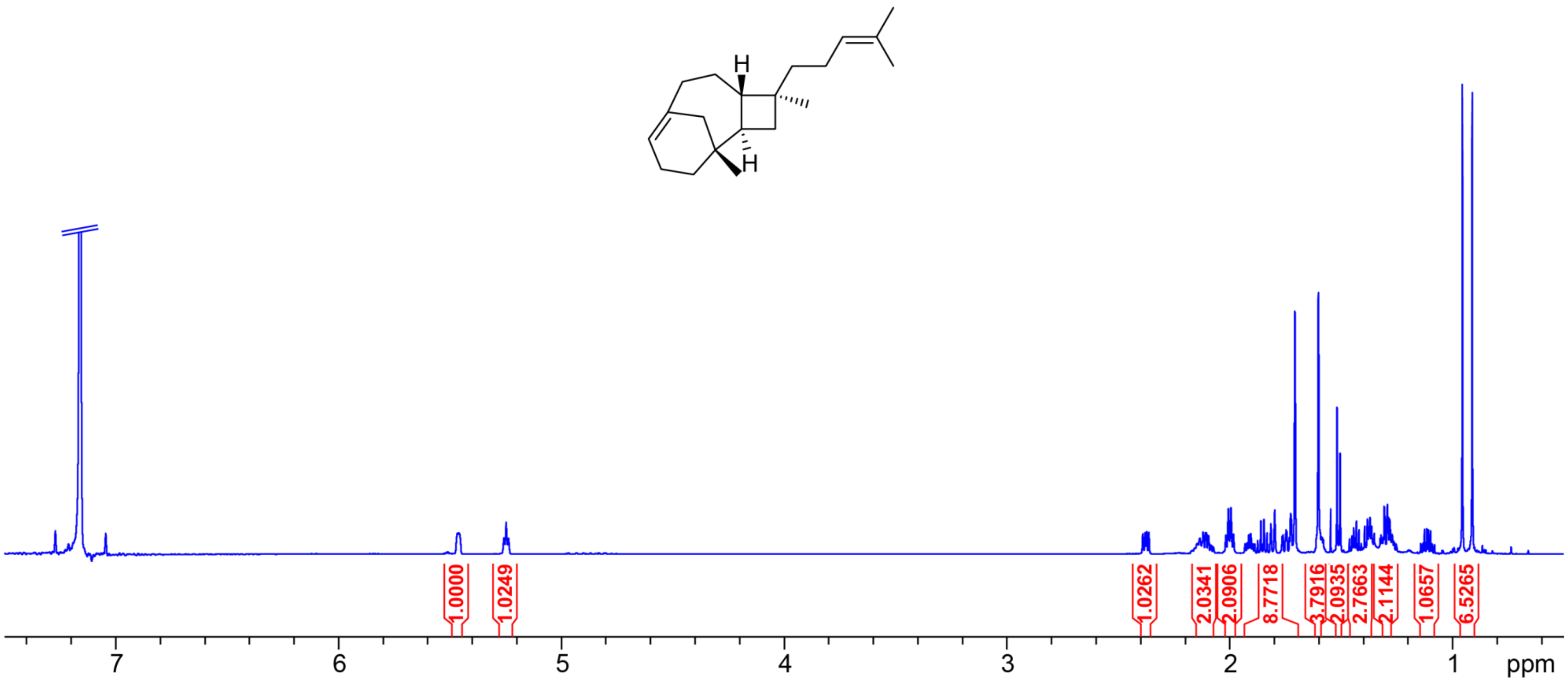

Figure S68. ^{1}H-NMR spectrum of **20** (700 MHz, C_6D_6).

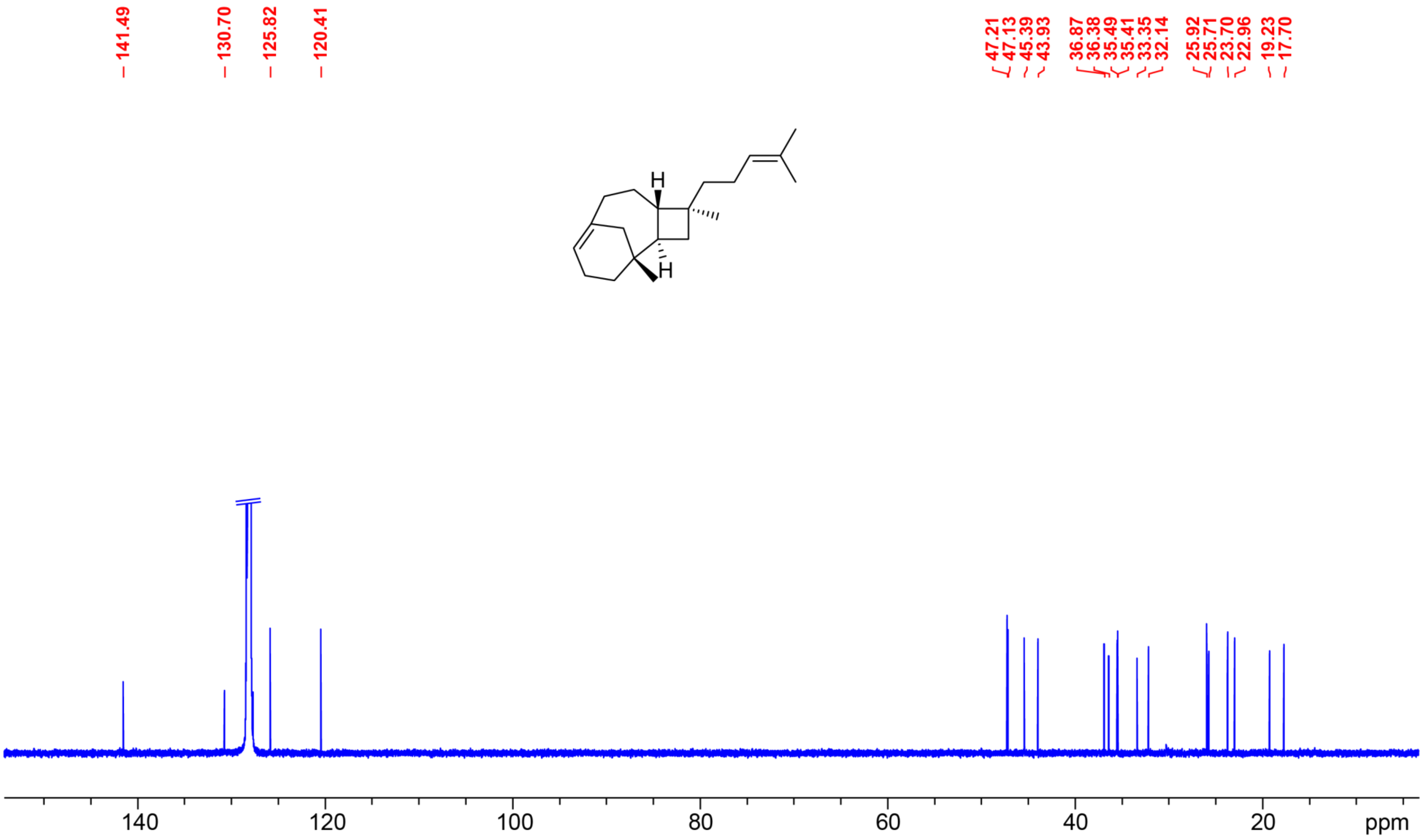

Figure S69. ^{13}C-NMR spectrum of **20** (176 MHz, C_6D_6).

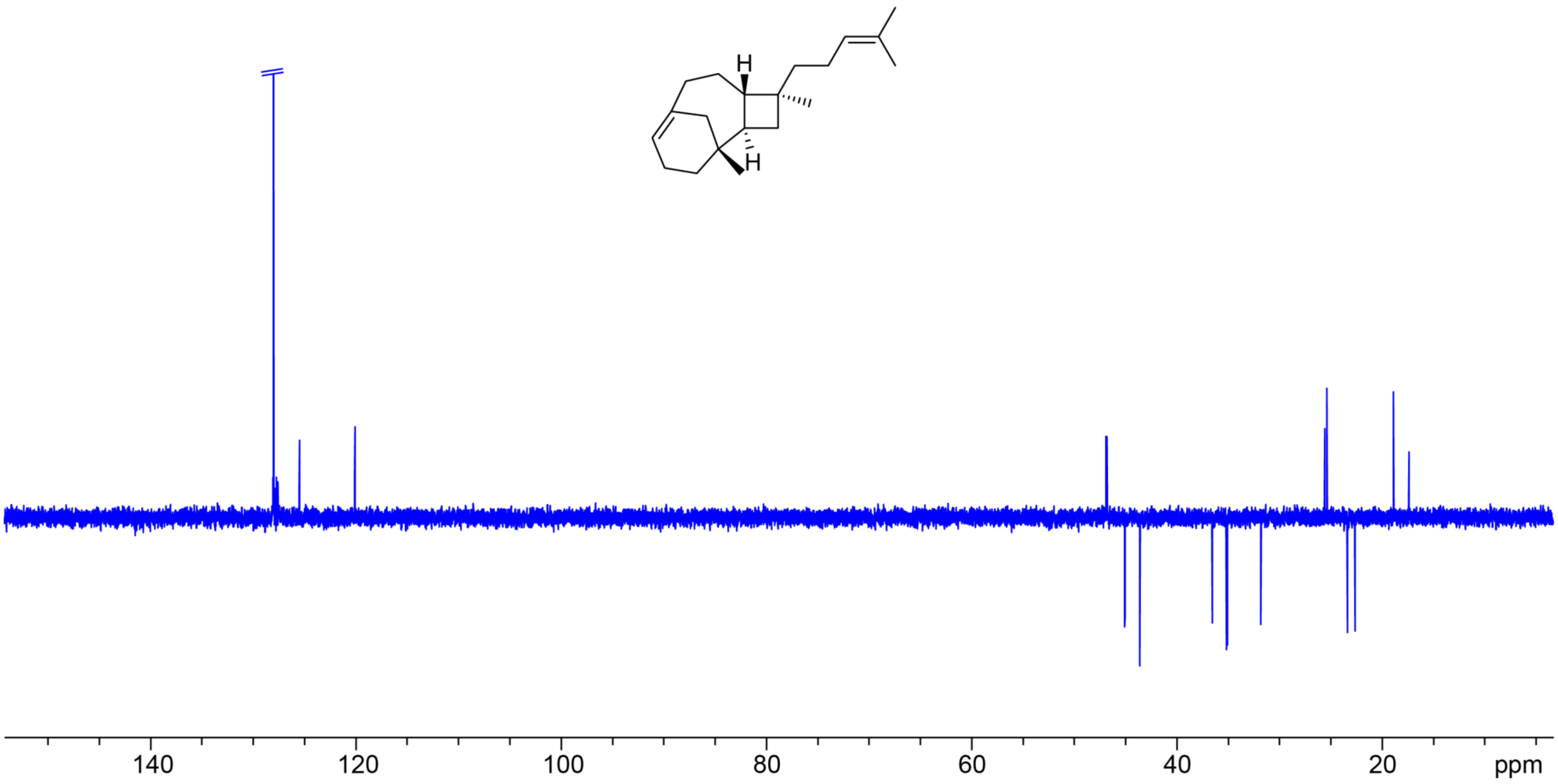

Figure S70. ^{13}C-DEPT135 spectrum of **20** (176 MHz, C_6D_6).

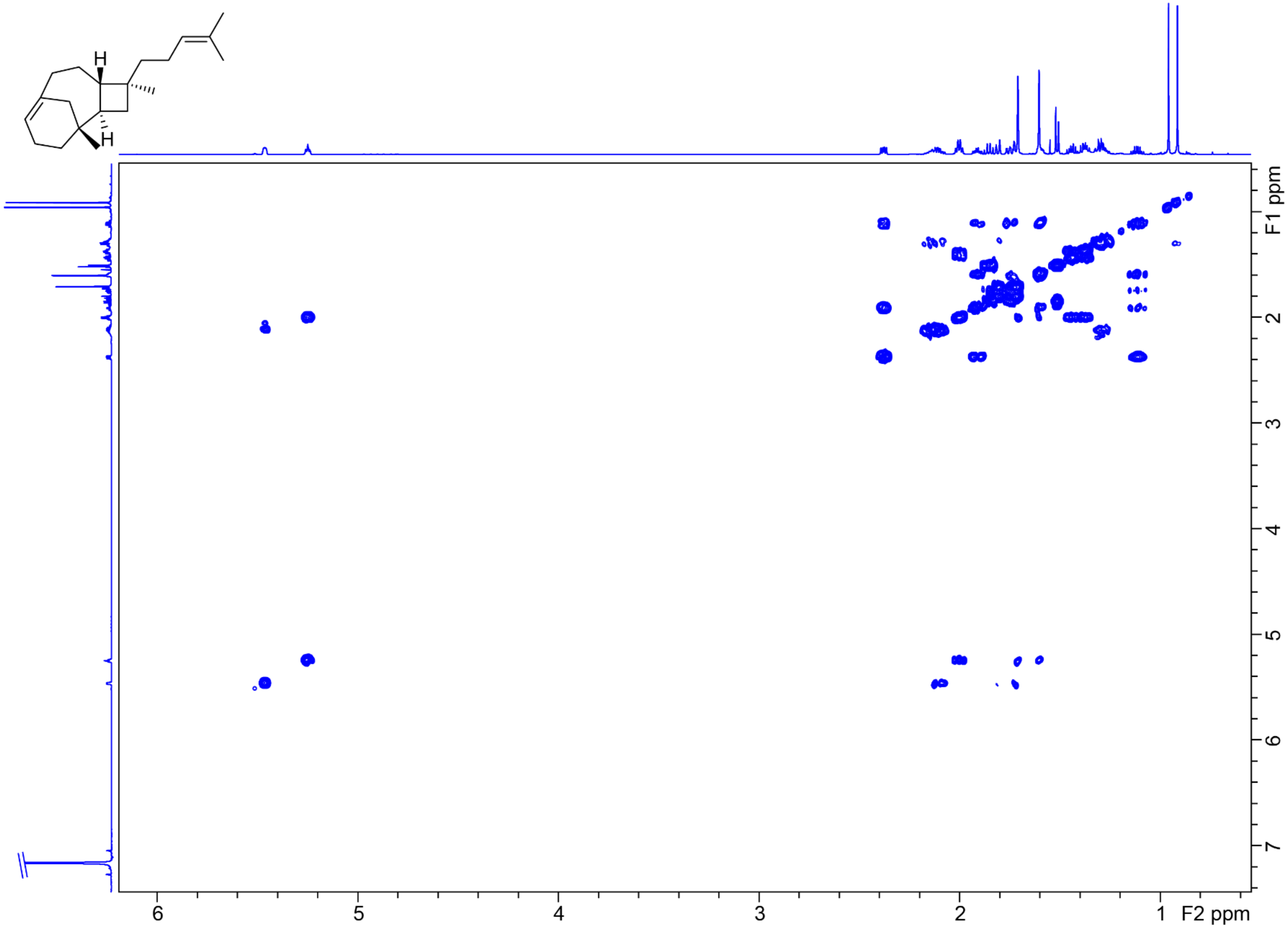

Figure S71. ^{1}H-^{1}H-COSY spectrum (C_6D_6) of **20**.

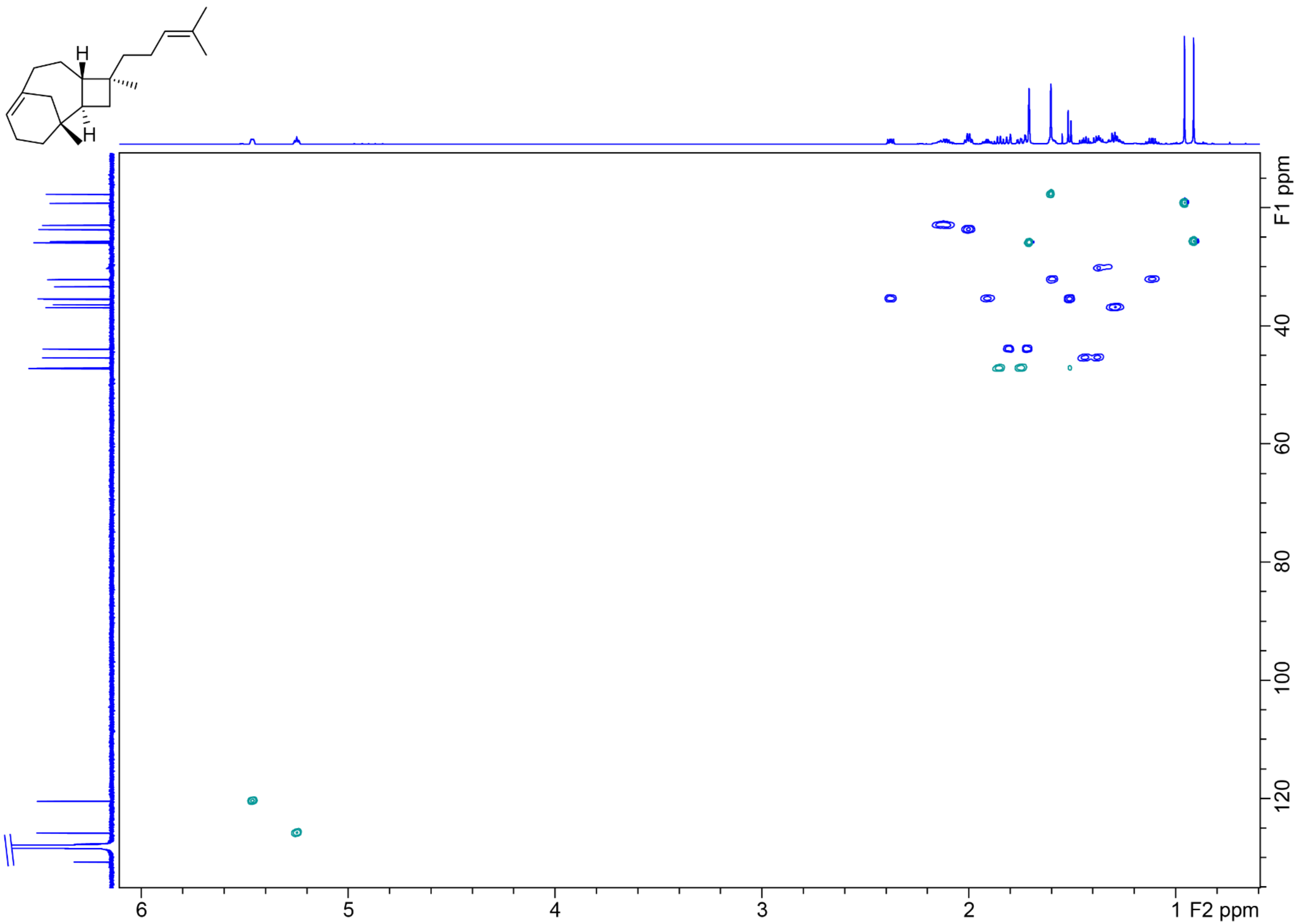

Figure S72. HSQC spectrum (C_6D_6) of **20**.

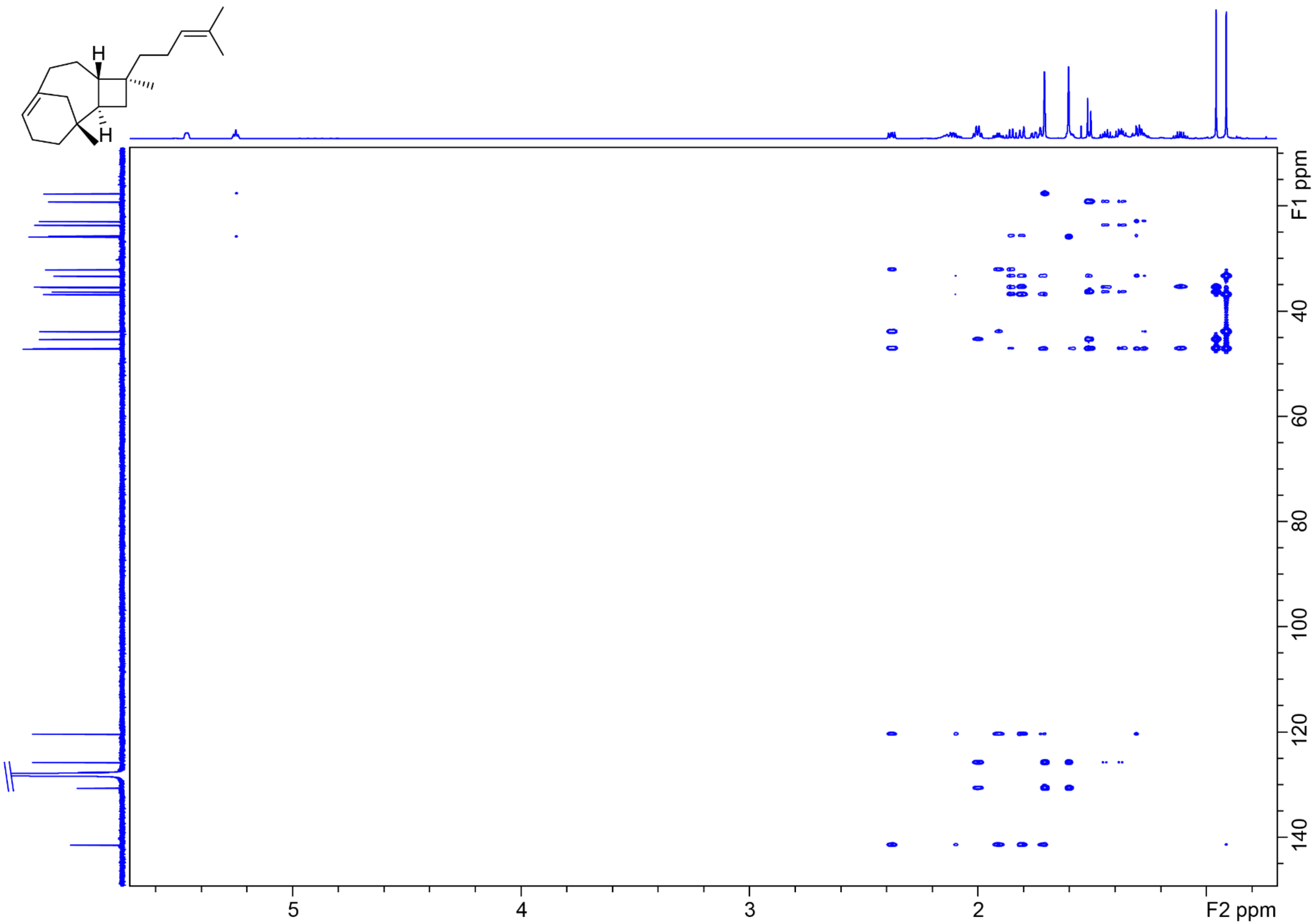

Figure S73. HMBC spectrum (C_6D_6) of **20**.

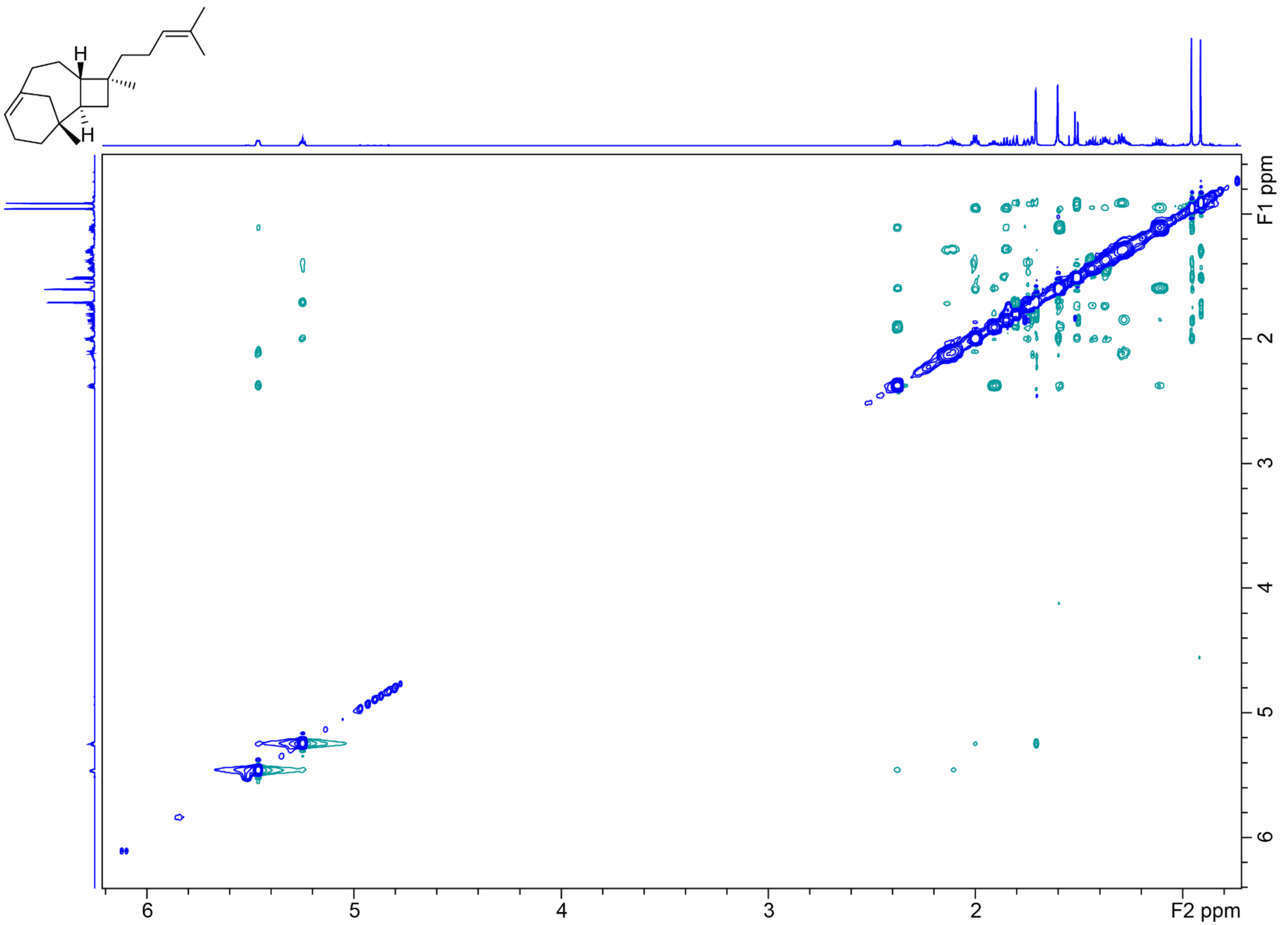

Figure S74. NOESY spectrum (C_6D_6) of **20**.

Incubation experiments with labelled substrates and with (*R*)- and (*S*)-GLPP

Incubation experiments with labelled substrates and with (*R*)- and (*S*)-GLPP were performed with labelled GGPP or its precursors, or with (*R*)- and (*S*)-GLPP (ca. 1 mg in 1 mL 25 mM NH_4HCO_3) in incubation buffer (5 mL) using preparations of purified enzymes (each as listed in Table S10. After incubation at 28 °C overnight, the products obtained with CjCsS were extracted with C_6D_6 (600 µL + 300 µL), then the extracts were dried with $MgSO_4$ and treated with silica gel, followed by analysis by NMR and/or GC/MS, while the products obtained with CjNdtS and DsJS were analysed by NMR and/or GC/MS directly after the extraction.

Table S12. Incubation experiments with labelled substrates and with (*R*)- and (*S*)-GLPP with CjCsS and CjNtPS.

entry	substrates	enzymes[a]	results shown in
1	DMAPP + (*E*)-(4-^{13}C,4-^{2}H)IPP[14]	GGPPS,[15] CjCsS	Figures S75 – S77
2	DMAPP + (*Z*)-(4-^{13}C,4-^{2}H)IPP[14]	GGPPS, CjCsS	Figures S75 – S77
3	(*R*)-(1-^{13}C,1-^{2}H)IPP[16]	GGPPS, IDI,[16] CjCsS	Figures S78 – S80 and S83
4	(*S*)-(1-^{13}C,1-^{2}H)IPP[16]	GGPPS, IDI, CjCsS	Figures S78 – S80 and S83
5	(3-^{13}C,2-^{2}H)DMAPP[17] + IPP	GGPPS, CjCsS	Figure S81
6	(3-^{13}C)GPP[18] + (1,1-$^{2}H_2$)IPP[19]	GGPPS, CjCsS	Figure S82
7	(*R*)-GLPP[19]	CjCsS	Figure S84
8	(*S*)-GLPP[19]	CjCsS	Figure S84
9	(10,10,10-$^{2}H_3$)GPP + (1-^{13}C)IPP[15]	GGPPS, CjNdtS, Δ	Figure S111
10	(1-^{13}C)GGPP[20] in D_2O	CjNdtS, Δ	Figure S112
11	(*S*)-(1-^{13}C,1-^{2}H)GGPP[19]	CjNdtS, Δ	Figure S113
12	**27** in D_2O	Δ	Figure S130
13	(*R*)-GLPP	CjNdtS	Figure S132
14	(*S*)-GLPP	CjNdtS	Figure S132
15	(3-^{13}C,2-^{2}H)DMAPP + IPP	GGPPS, CjNdtS	Figures S133 and S134
16	(*R*)-(1-^{13}C,1-^{2}H)IPP	IDI, GGPPS, CjNdtS	Figure S135
17	(*S*)-(1-^{13}C,1-^{2}H)IPP	IDI, GGPPS, CjNdtS	Figure S135
18	(3-^{13}C)GGPP[20] in D_2O	CjNdtS	Figure S136
19	DMAPP + (*E*)-(4-^{13}C,4-^{2}H)IPP	CjNdtS	Figure S137
20	DMAPP + (*Z*)-(4-^{13}C,4-^{2}H)IPP	CjNdtS	Figure S137
21	DMAPP + (*E*)-(4-^{13}C,4-^{2}H)IPP	GGPPS, DsJS	Figure S146
22	DMAPP + (*Z*)-(4-^{13}C,4-^{2}H)IPP	GGPPS, DsJS	Figure S146
23	(*R*)-(1-^{13}C,1-^{2}H)IPP	GGPPS, IDI, DsJS	Figures S147 and S150
24	(*S*)-(1-^{13}C,1-^{2}H)IPP	GGPPS, IDI, DsJS	Figures S147 and S150
25	(3-^{13}C,2-^{2}H)DMAPP + IPP	GGPPS, DsJS	Figure S149
26	(3-^{13}C)GGPP in D_2O	DsJS	Figure S151
27	(*R*)-GLPP	DsJS	Figure S152
28	(*S*)-GLPP	DsJS	Figure S152
29	(*R*)-(1-^{13}C,1-^{2}H)-*iso*-GGPP[21]	CjNdtS	Figures S170 and S171
30	(*S*)-(1-^{13}C,1-^{2}H)-*iso*-GGPP[21]	CjNdtS	Figures S170 and S171

[a] Further conversion of enzyme products through thermal reaction is indicated by "Δ" (for experimental details cf. "Thermal reactions with **21**", page 120).

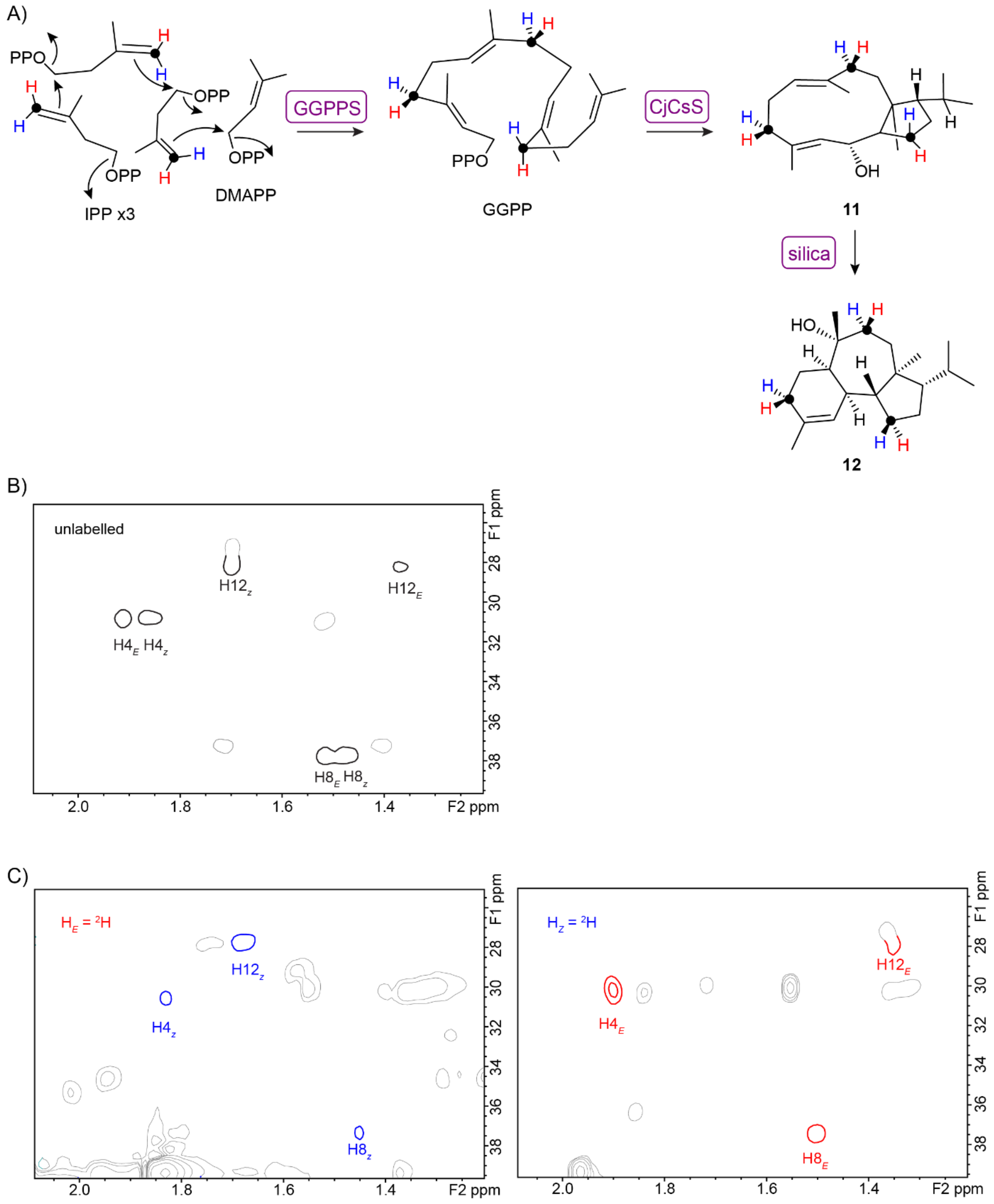

Figure S75. The absolute configuration of **12**. A) Cyclisation of labelled GGPP obtained from (*E*)-(4-^{13}C,4-^{2}H)IPP (red H = ^{2}H) or (*Z*)-(4-^{13}C,4-^{2}H)IPP (blue H = ^{2}H) plus DMAPP with GGPPS and CjCsS, followed by treatment with silica gel. B) Partial HSQC spectrum of unlabelled **12** showing the region for C4, C8 and C12. C) HSQC spectra of labelled **12** obtained from (*E*)-(4-^{13}C,4-^{2}H)IPP (left) and from (*Z*)-(4-^{13}C,4-^{2}H)IPP (right). Together with the NOESY based assignments for the hydrogens (Table S3) these data point to the shown absolute configuration of **12**. Black dots indicate ^{13}C-labelled carbons.

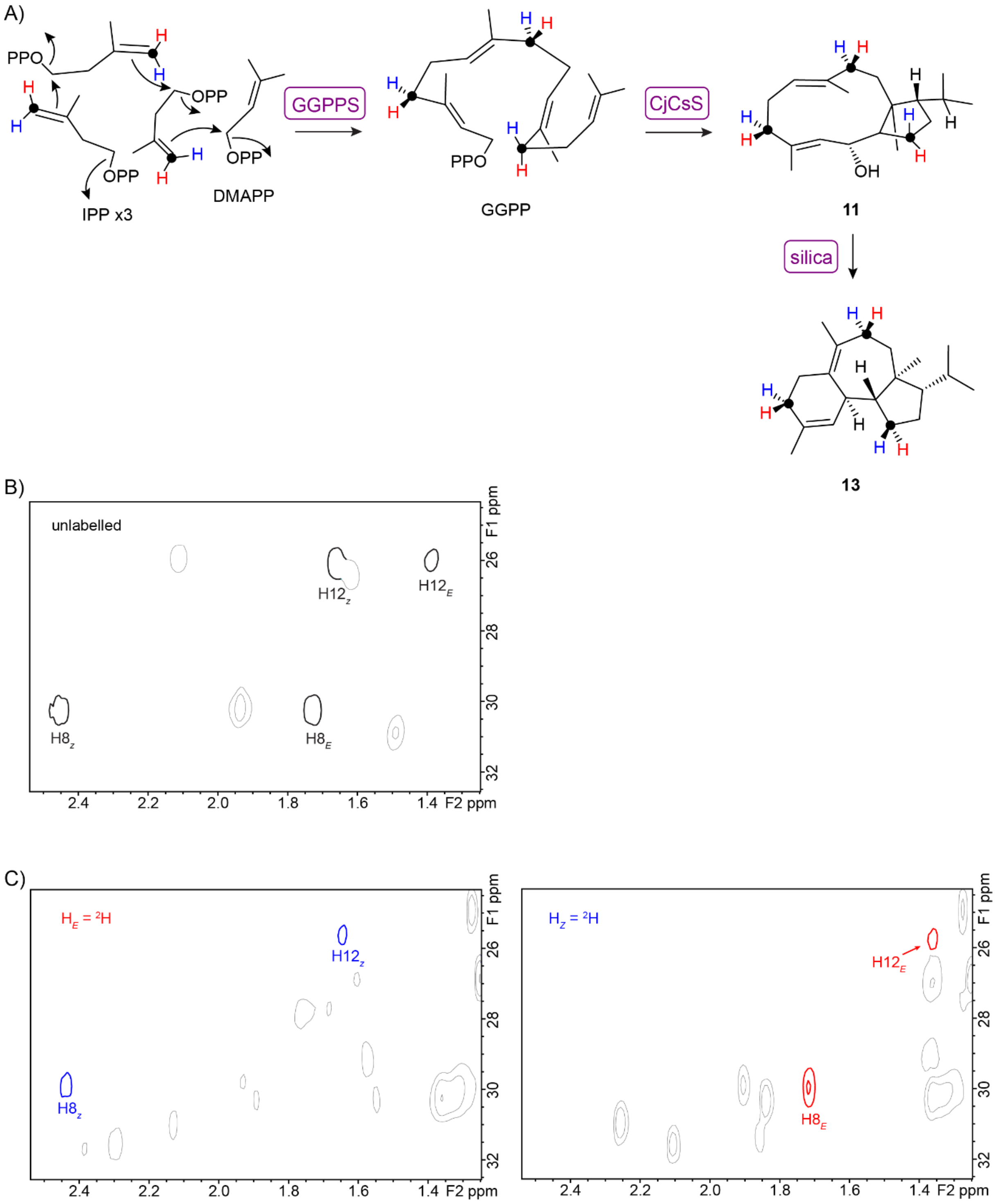

Figure S76. The absolute configuration of **13**. A) Cyclisation of labelled GGPP obtained from (*E*)-(4-^{13}C,4-^{2}H)IPP (red H = ^{2}H) or (*Z*)-(4-^{13}C,4-^{2}H)IPP (blue H = ^{2}H) plus DMAPP with GGPPS and CjCsS, followed by treatment with silica. B) Partial HSQC spectrum of unlabeled **13** showing the region for C4, C8 and C12. C) HSQC spectra of labelled **13** obtained from (*E*)-(4-^{13}C,4-^{2}H)IPP (left) and from (*Z*)-(4-^{13}C,4-^{2}H)IPP (right). Together with the NOESY based assignments for the hydrogens (Table S4) these data point to the shown absolute configuration of **13**. Black dots indicate ^{13}C-labelled carbons.

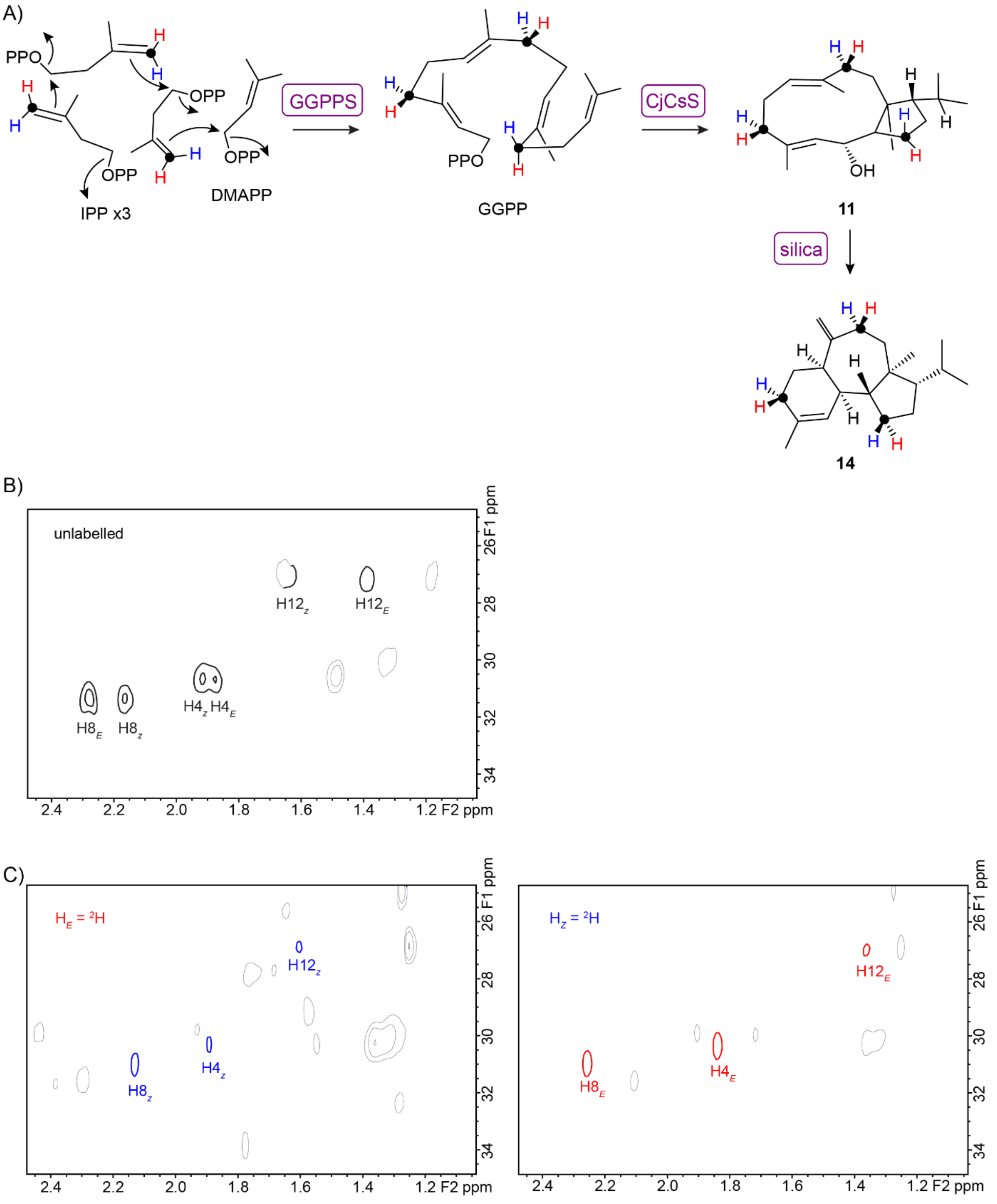

Figure S77. The absolute configuration of **14**. A) Cyclisation of labelled GGPP obtained from (E)-(4-^{13}C,4-^{2}H)IPP (red H = ^{2}H) or (Z)-(4-^{13}C,4-^{2}H)IPP (blue H = ^{2}H) plus DMAPP with GGPPS and CjCsS, followed by treatment with silica gel. B) Partial HSQC spectrum of unlabelled **14** showing the region for C4, C8 and C12. C) HSQC spectra of labelled **14** obtained from (E)-(4-^{13}C,4-^{2}H)IPP (left) and from (Z)-(4-^{13}C,4-^{2}H)IPP (right). Together with the NOESY based assignments for the hydrogens (Table S5) these data point to the shown absolute configuration of **14**. Black dots indicate ^{13}C-labelled carbons.

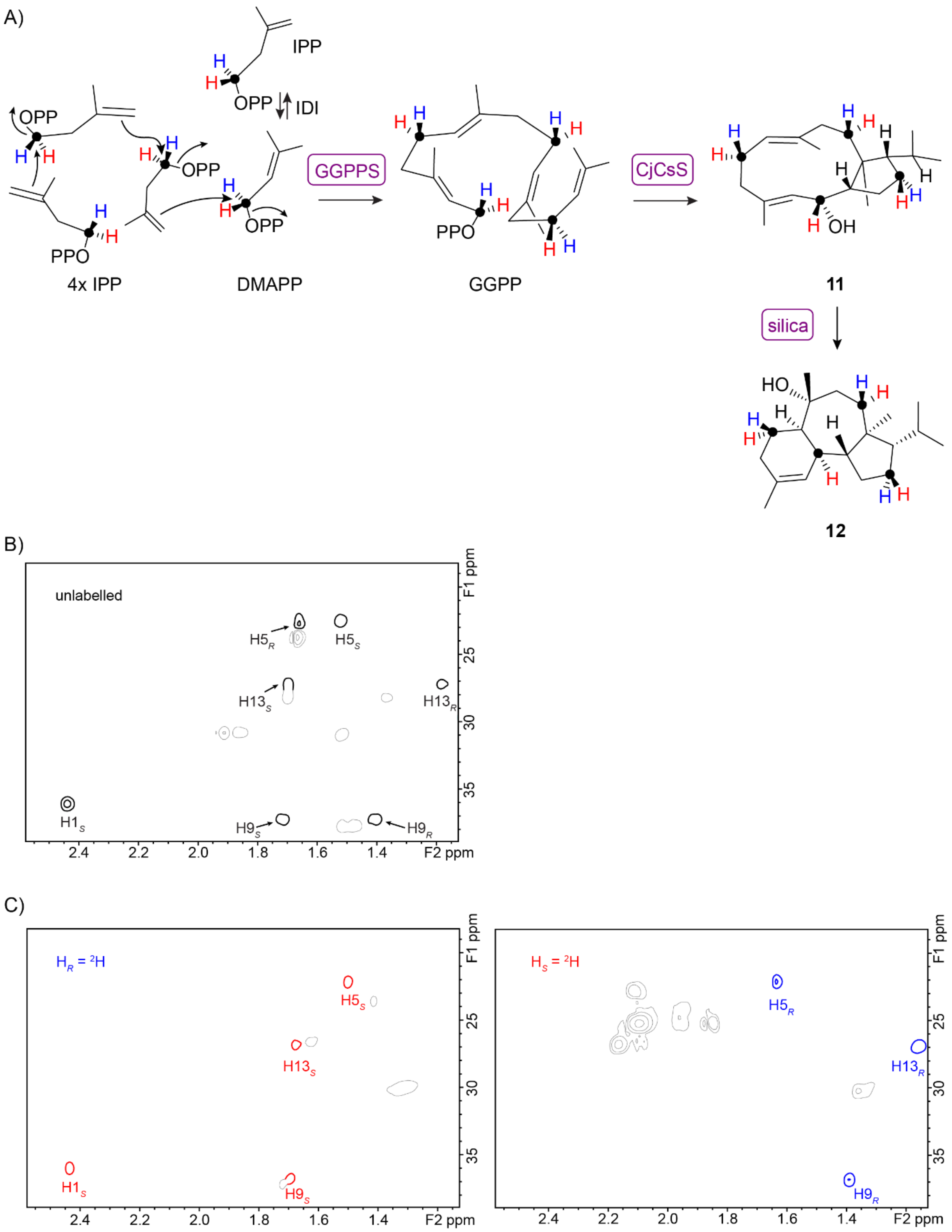

Figure S78. The absolute configuration of **12**. A) Cyclisation of labelled GGPP obtained from (*R*)-(1-^{13}C,1-^{2}H)IPP (red H = ^{2}H) or (*S*)-(1-^{13}C,1-^{2}H)IPP (blue H = ^{2}H) with GGPPS, IDI and CjCsS, followed by treatment with silica gel. B) Partial HSQC spectrum of unlabelled **12** showing the region for C1, C5, C9 and C13. C) HSQC spectra of labelled **12** obtained from (*R*)-(1-^{13}C,1-^{2}H)IPP (left) and from (*S*)-(1-^{13}C,1-^{2}H)IPP (right). Together with the NOESY based assignments for the hydrogens (Table S3) these data point to the shown absolute configuration of **12**. Black dots indicate ^{13}C-labelled carbons.

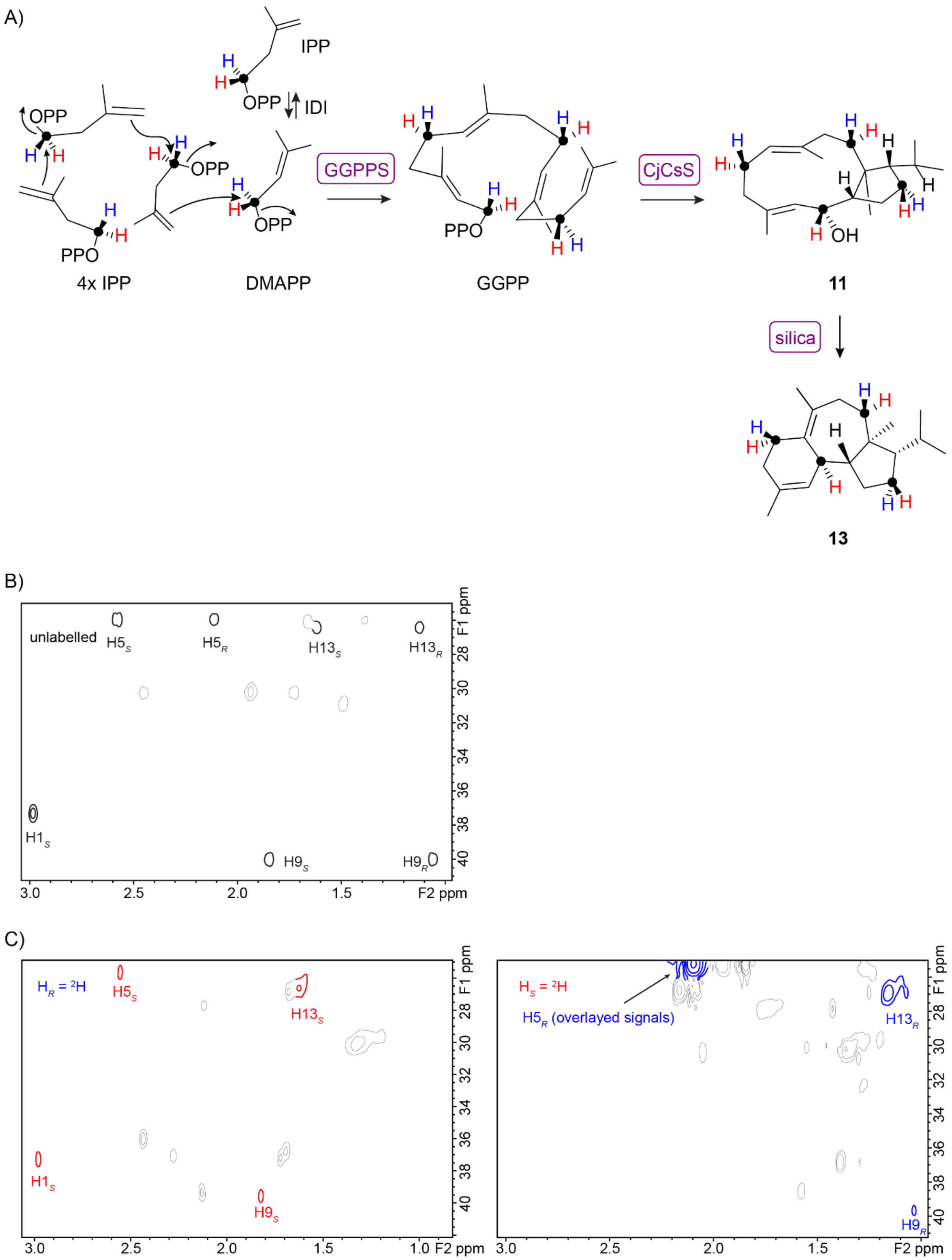

Figure S79. The absolute configuration of **13**. A) Cyclisation of labelled GGPP obtained from (*R*)-(1-^{13}C,1-^{2}H)IPP (red H = ^{2}H) or (*S*)-(1-^{13}C,1-^{2}H)IPP (blue H = ^{2}H) with GGPPS, IDI and CjCsS, followed by treatment with silica gel. B) Partial HSQC spectrum of unlabelled **13** showing the region for C1, C5, C9 and C13. C) HSQC spectra of labelled **13** obtained from (*R*)-(1-^{13}C,1-^{2}H)IPP (left) and from (*S*)-(1-^{13}C,1-^{2}H)IPP (right). Together with the NOESY based assignments for the hydrogens (Table S4) these data point to the shown absolute configuration of **13**. Black dots indicate ^{13}C-labelled carbons.

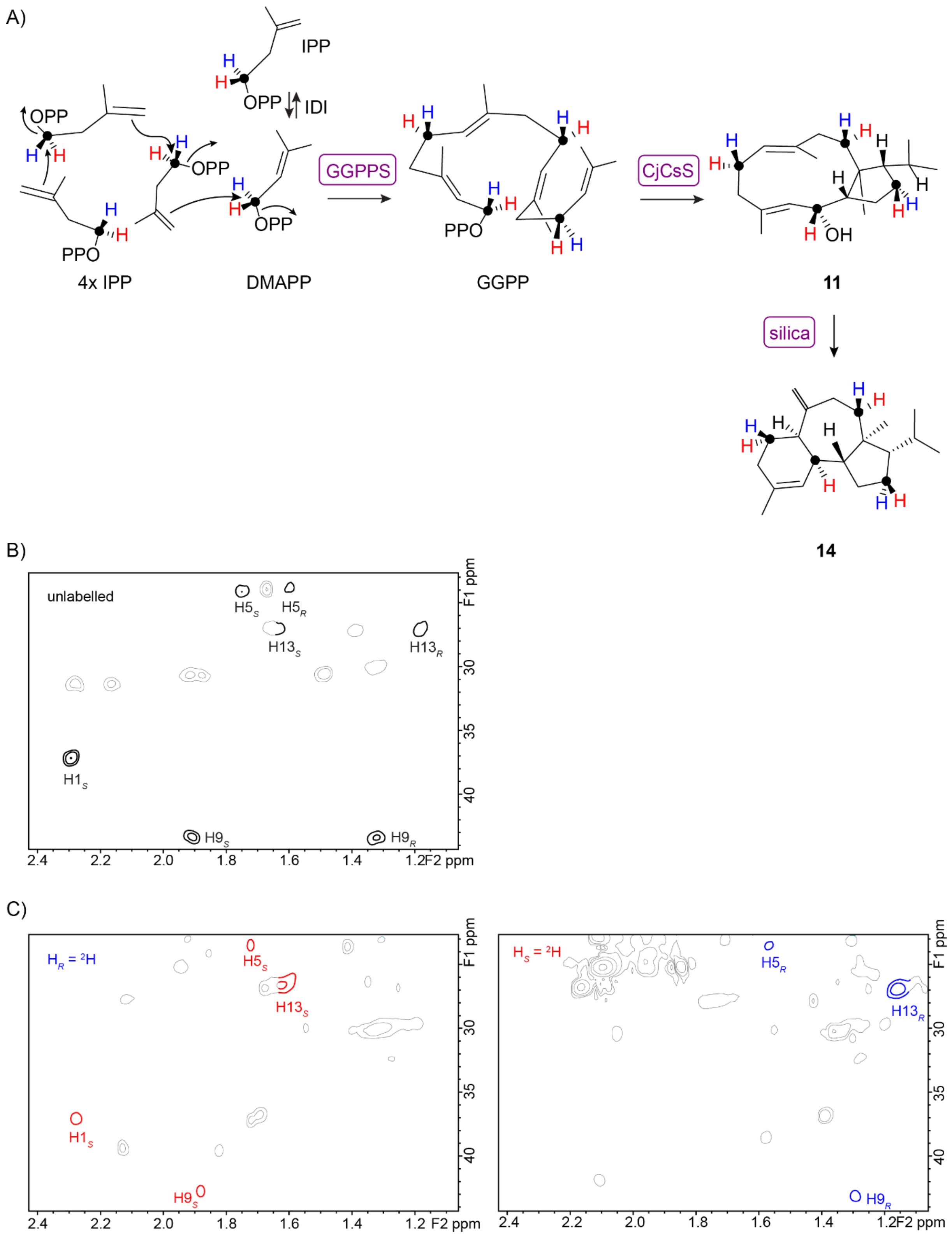

Figure S80. The absolute configuration of **14**. A) Cyclisation of labelled GGPP obtained from (*R*)-(1-^{13}C,1-^{2}H)IPP (red H = ^{2}H) or (*S*)-(1-^{13}C,1-^{2}H)IPP (blue H = ^{2}H) with GGPPS, IDI and CjCsS to **11**, followed by treatment with silica gel. B) Partial HSQC spectra of unlabelled **14** showing the region for C1, C5, C9 and C13. C) HSQC spectra of labelled **14** obtained from (*R*)-(1-^{13}C,1-^{2}H)IPP (left) and from (*S*)-(1-^{13}C,1-^{2}H)IPP (right). Together with the NOESY based assignments for the hydrogens (Table S5) these data point to the shown absolute configuration of **14**. Black dots indicate ^{13}C-labelled carbons.

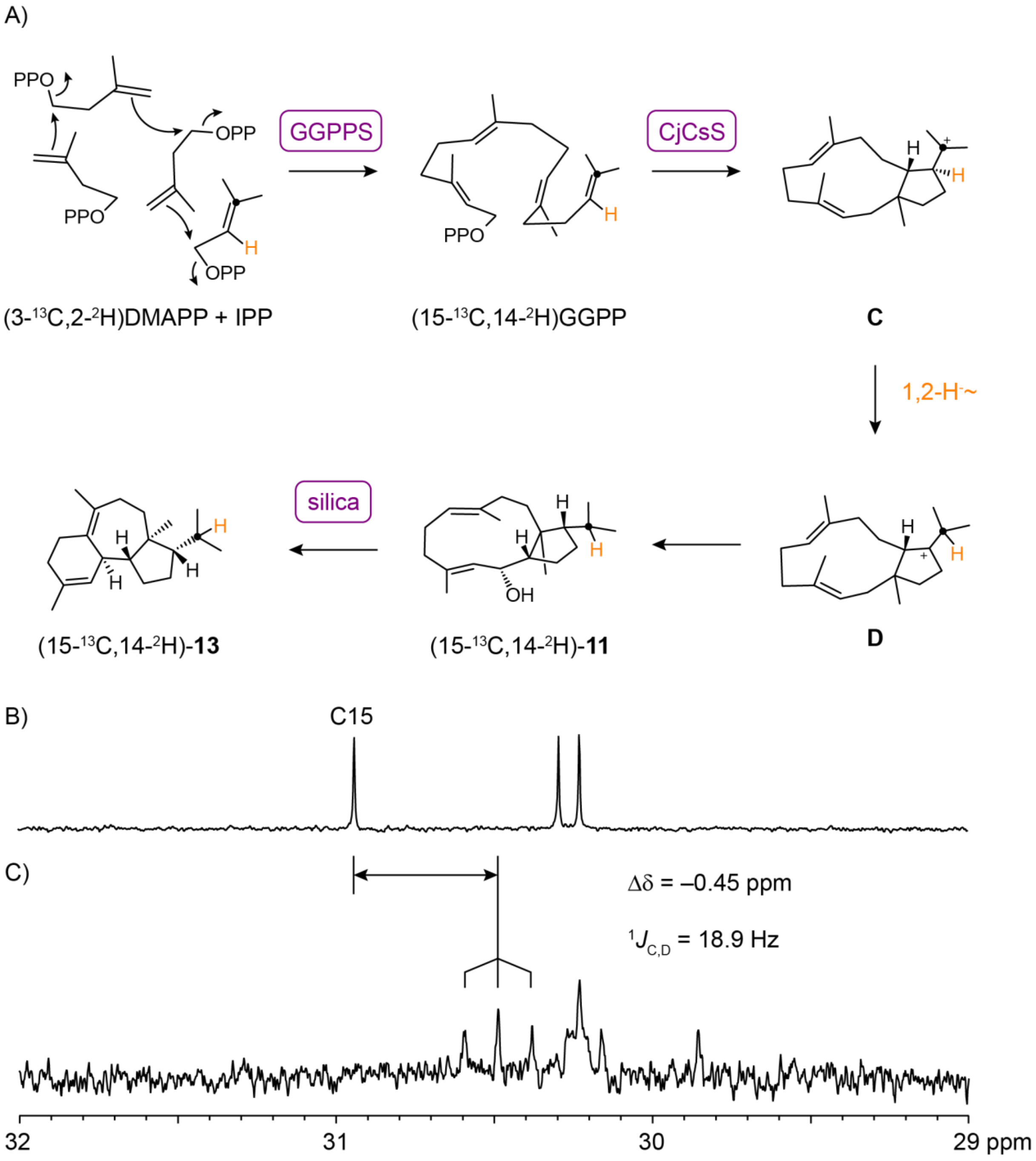

Figure S81. The 1,2-hydride shift from **C** to **D** in the biosynthesis of **11** (Scheme 1 of main text). A) Conversion of (3-^{13}C,2-^{2}H)DMAPP plus IPP with GGPPS and CjCsS to **11**, followed by the treatment with silica gel. B) Partial ^{13}C spectra showing the region for C15 of B) unlabelled **13** and C) labelled **13**. The upfield shifted triplet for C15 ($\Delta\delta$ = –0.45 ppm, $^{1}J_{C,D}$ = 18.9 Hz) indicates that deuterium migrates from C14 to C15, supporting the 1,2-hydride shift from **C** to **D**. Black dots represent ^{13}C-labelled carbons.

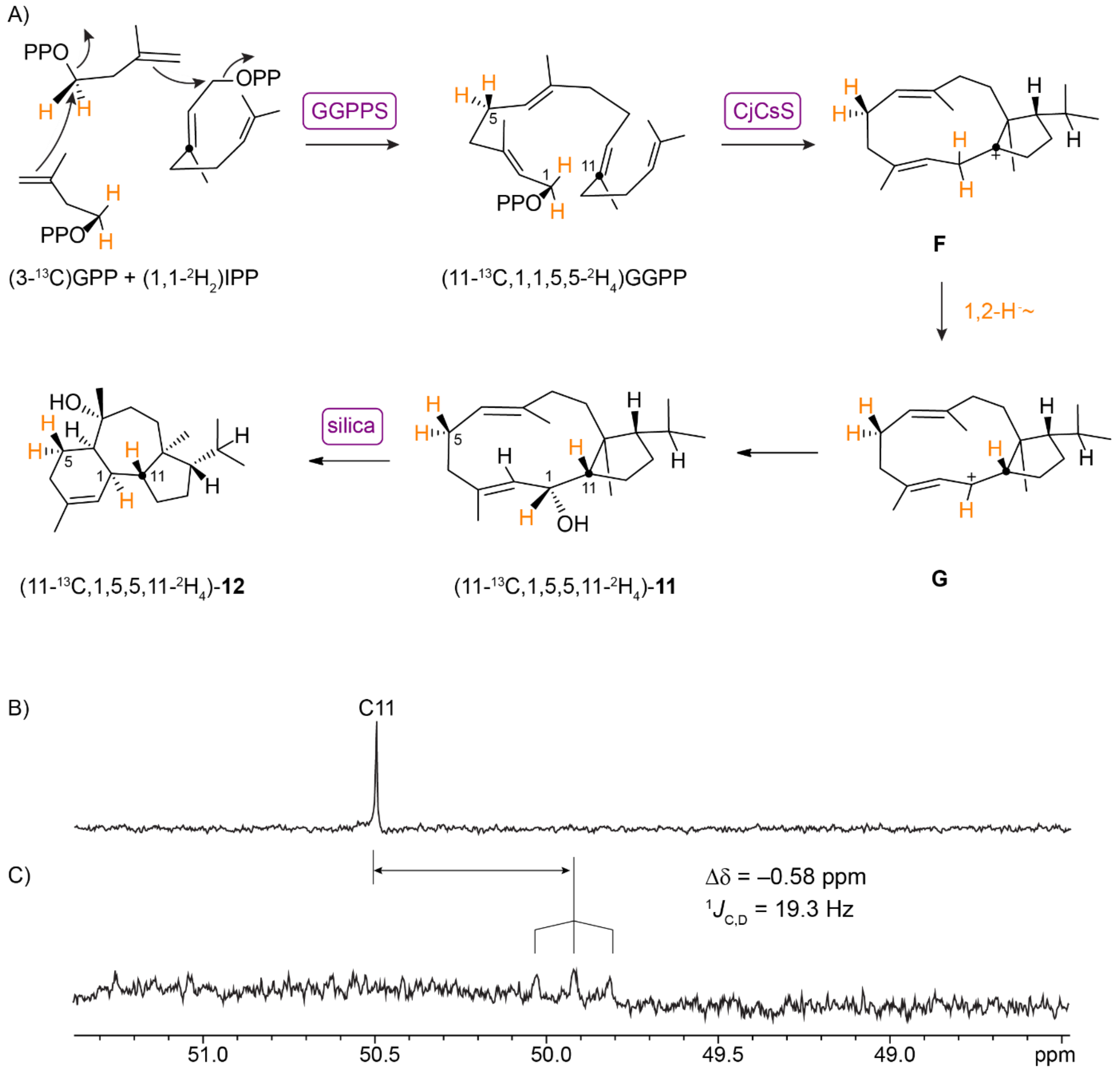

Figure S82. The 1,2-hydride shift from **F** to **G** in the biosynthesis of **11** (Scheme 1 of main text). A) Conversion of (3-^{13}C)GPP plus (1,1-$^{2}H_2$)IPP (orange H = ^{2}H) with GGPPS and CjCsS to **11**, followed by the treatment with silica gel. B) Partial ^{13}C spectra showing the region for C11 of B) unlabelled **12** and C) labelled **12**. The upfield shifted triplet for C11 ($\Delta\delta$ = –0.58 ppm, $^{1}J_{C,D}$ = 19.3 Hz) indicates the migration of a deuterium from C1 to C11, supporting the 1,2-hydride shift from **F** to **G**. Black dots represent ^{13}C-labelled carbons.

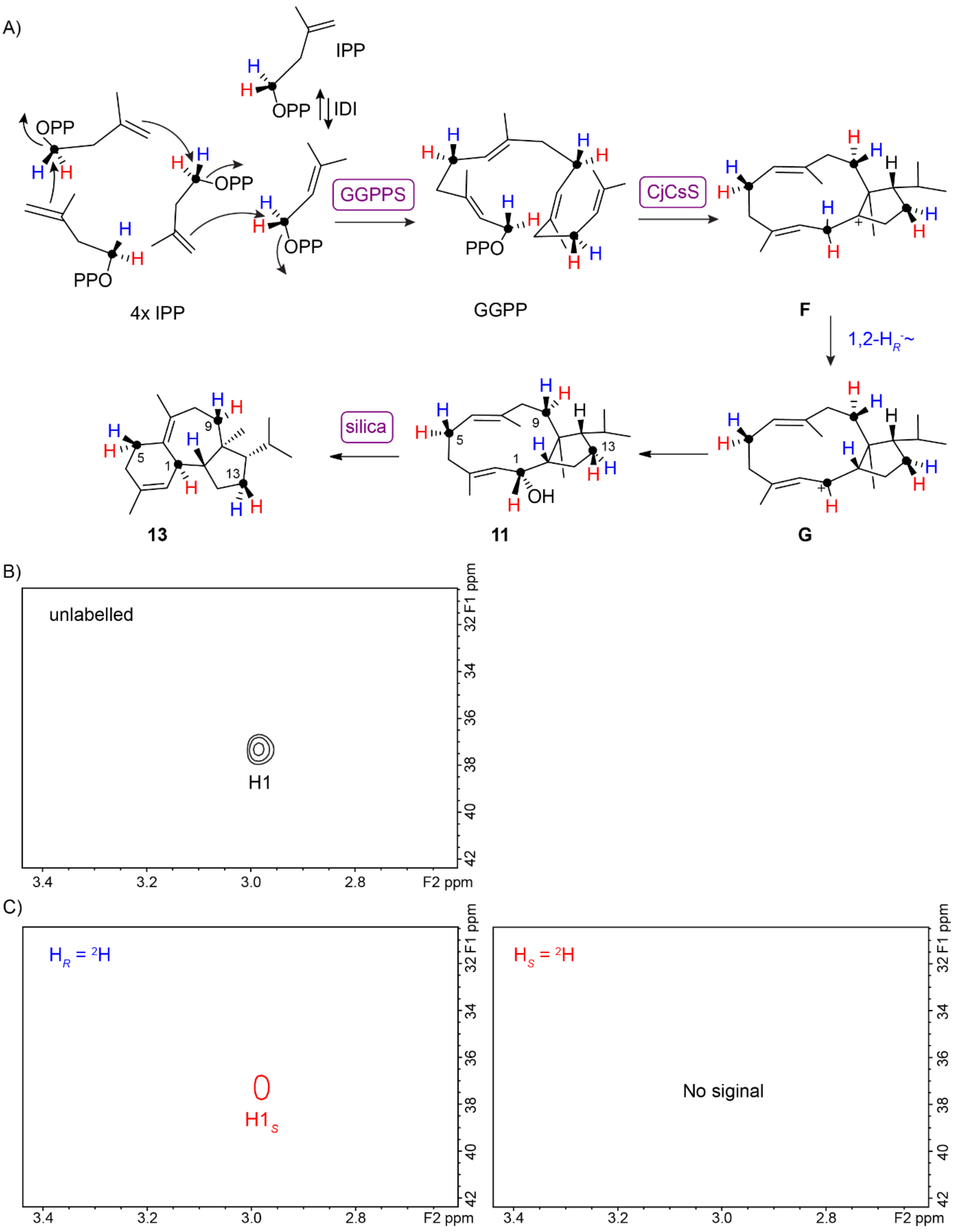

Figure S83. The stereoselectivity of the 1,2-hydride shift from **F** to **G** in the biosynthesis of **11** (Scheme 1 of main text). A) Cyclisation of labelled GGPP obtained from (*R*)-(1-^{13}C,1-^{2}H)IPP (blue H = ^{2}H) or (*S*)-(1-^{13}C,1-^{2}H)IPP (red H = ^{2}H) with GGPPS and CjCsS to **11**, followed by treatment with silica gel to yield **13**. B) Partial HSQC spectra showing the region for C1 of B) unlabelled **13** and C) labelled **13** obtained from (*R*)-(1-^{13}C,1-^{2}H)IPP (left) and from (*S*)-(1-^{13}C,1-^{2}H)IPP (right), indicating the migration of the 1-*pro-R* hydrogen from C1 to C11 and retainment of the 1-*pro-S* hydrogen at C1. Black dots represent ^{13}C-labelled carbons.

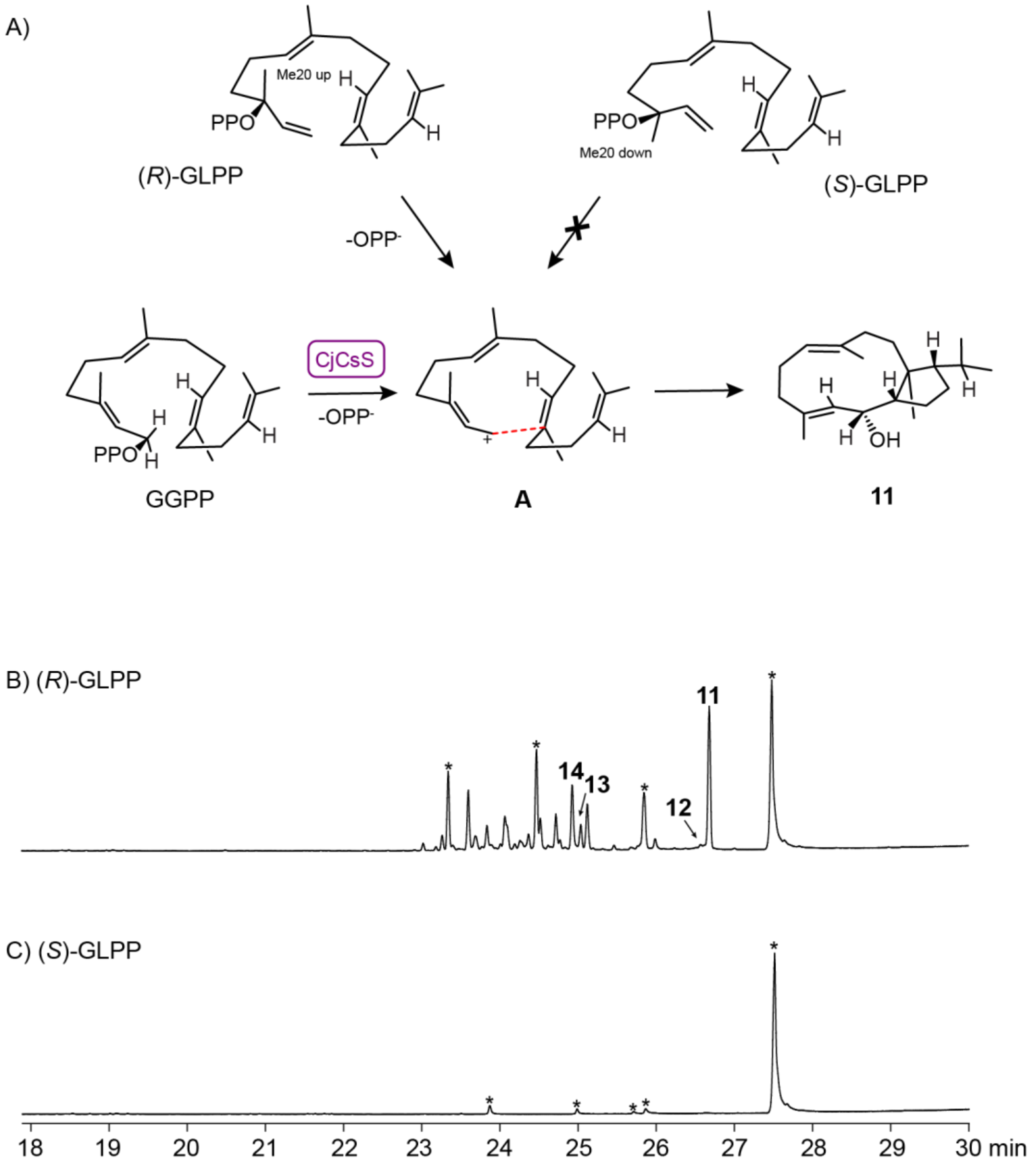

Figure S84. The conformational fold of GGPP in the biosynthesis of **11** (Scheme 1 of main text). A) Proposed substrate conformations of GGPP, (*R*)- and (*S*)-GLPP in the biosynthesis of **11** by CjCsS. GC/MS analysis of the crude extracts obtained from the incubation of B) (*R*)-GLPP and C) (*S*)-GLPP with CjCsS, showing efficient product formation from (*R*)-GLPP, but not from (*S*)-GLPP.

Computational Methods

All computed structures were geometry optimized without restrictions and were characterized as minima or as transition state structures by frequency analyses using the B97D3/6-31g(d,p) method with the density fitting approximation for s- and p-functions, including Grimme's empirical D3-dispersion correction[22] in Gaussian16.[23] Frequency computations also provided Gibbs corrections, which include Grimme's quasi-RRHO approach with a frequency cut-off value of 100.0 wave numbers using GoodVibes.[24,25] For single-point energies, Head-Gordon's range-separated hybrid meta-GGA functional ωB97M-V was applied with the Def2-TZVPPD basis set in Orca (6.1.0), due to its general suitability to describe reaction energies and barriers, also for the chemistry of carbocations.[26-29] Conformational analyses were performed with xTB-GFN2 in the CREST 2.12 program (github.com/crest-lab), developed by the Grimme group.[30-34]

Table S13. Results of DFT calculations (wB97M-V/Def2-TZVPPD//B97D3/6-31G(d,p)) for the cyclisation cascade from GGPP to **11**, the NBS-mediated cyclisation of **11** to **17** and **18**, and the protonation induced cyclisation of **11** to **12** – **14** (Scheme 1 of main text).

Structure[a]	Gibbs energy (298.15K) in Hartree	energy relative to A in kcal/mol	free energy of activation / kcal/mol	free reaction energy / kcal/mol
**Cyclisation cascade from GGPP to 11**				
**A**	–781.131261	0.0		
**A-C-TS**	–781.120828	6.5	6.5	
**C**	–781.164955	–21.1		–21.1
**C**	–781.158318	–17.0		
**C-D-TS**	–781.155585	–15.3	1.7	
**D**	–781.166865	–22.3		–5.4
**D**	–781.168330	–23.3		
**D-E-TS**	–781.162220	–19.4	3.8	
**E**	–781.169078	–23.7		–0.5
**E**	–781.165854	–21.7		
**E-F-TS**	–781.156210	–15.7	6.1	
**F**	–781.159781	–17.9		3.8
**F**	–781.159788	–17.9		
**F-G-TS**	–781.146627	–9.6	8.3	
**G**	–781.163866	–20.5		–2.6
**NBS-mediated cyclisation of 11 to 17 and 18**				
**H**	–3430.994502	0.0		
**H-I-TS**	–3430.992083	1.5	1.5	
**I**	–3431.037492	–27.0		–27.0
**Protonation-induced conversion of 11 to 12 – 14**				
**J**	–857.257587	0.0		
**J-K-TS**	–857.242040	9.8	9.8	
**K**	–857.258071	–0.3		–0.3
**L**	–781.189049	0.0		
**L-M-TS**	–781.187916	0.7	0.7	
**M**	–781.189600	–0.3		–0.3

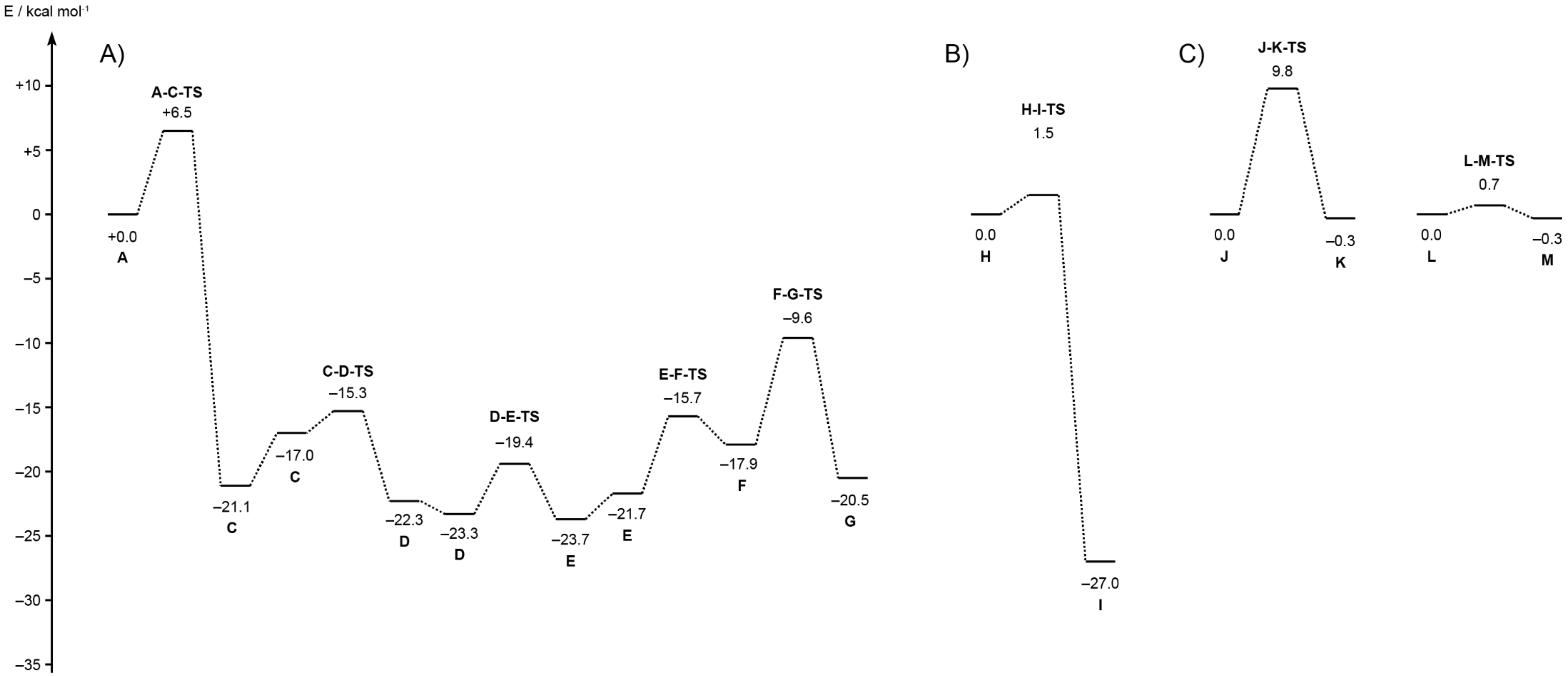

Figure S85. Computed energy profiles for A) the cyclisation cascade from GGPP to **11**, B) the NBS-mediated cyclisation of **11** to **17** and **18**, and C) the protonation induced cyclisation of **11** to **12** – **14** (Scheme 1 of main text, wB97M-V/Def2-TZVPPD//B97D3/6-31G(d,p), 298 K).

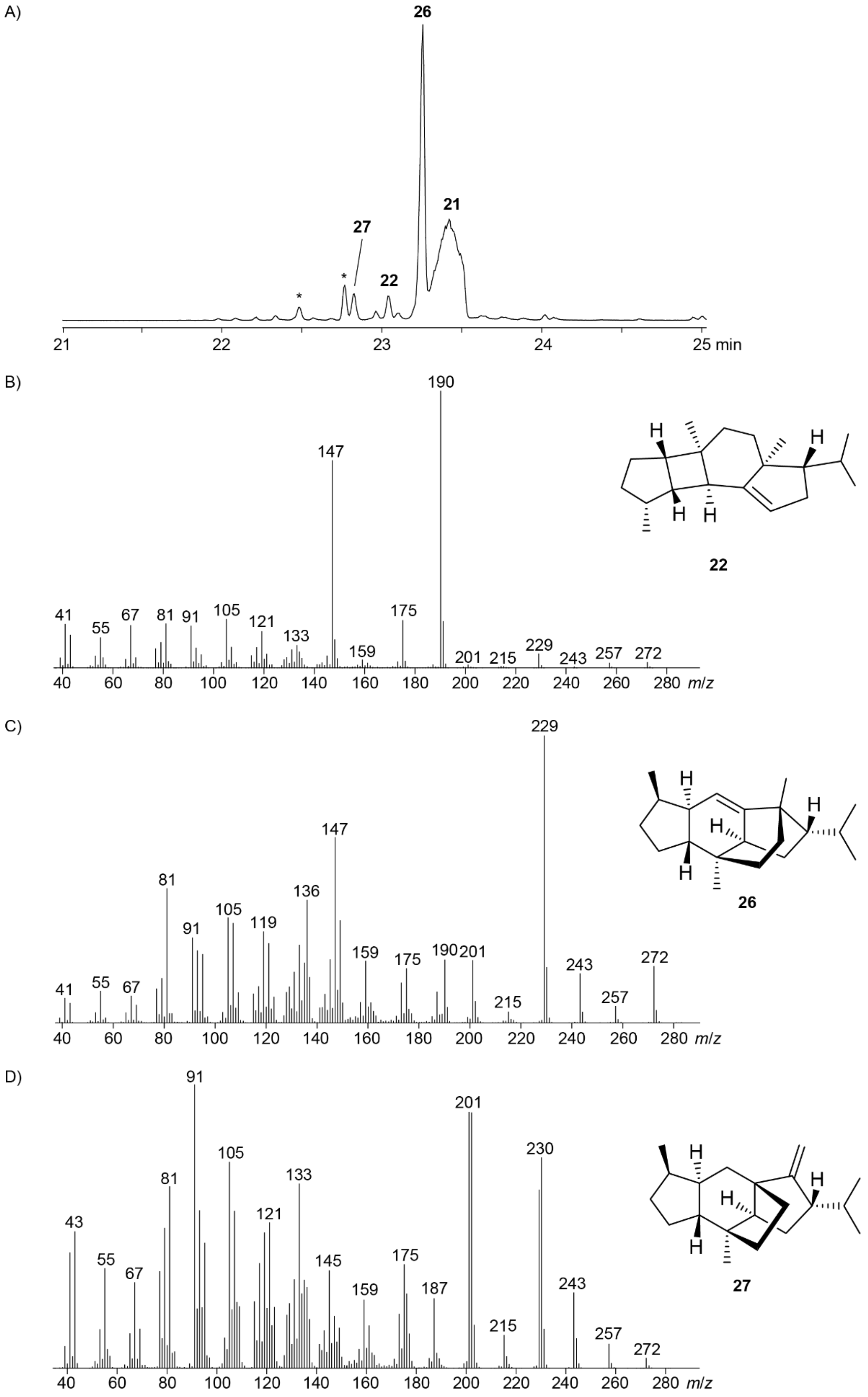

Figure S86. Enzymatic conversion of GGPP with CjNdtS (GPP, FPP and GFPP were not accepted). A) Total ion chromatogram of an extract from the incubation of GGPP with CjNdtS. The broad peak for **21** points to a thermal reaction during GC analysis. Asterisks indicate spontaneous lysis products of GGPP. EI mass spectra of B) **22**, C) **26** and D) **27**.

Conversion of GGPP with CjNdtS and product isolation

A preparative scale enzymatic conversion of GGPP (60 mg, 120 μmol) was conducted in incubation buffer (120 mL) with the addition of an enzyme preparation of CjNdtS (20 mL, 3.8 mg mL^{-1}), followed by incubation for 16 h at 30 °C. The reaction mixturs was extracted with n-pentane (3 x 100 mL), the combined extracts were dried with $MgSO_4$ and the solvent was evaporated. The crude product was purified via column chromatography on silica gel to yield pure **21** (2.2 mg, 8.1 μmol, 6.8%) as a colourless oil. The fraction containing **22** was further purified via column chromatography on $AgNO_3$ impregnated silica gel with n-pentane to yield pure **22** (0.4 mg, 1.5 μmol, 1.3%) as a colourless oil.

Neodolabella-1(14),2,7-triene (21). TLC (pentane): R_f = 0.78. GC (HP-5MS): *I* = 1969. MS (EI, 70 eV): *m*/*z* (%) = 43 (6), 55 (5), 67 (4), 81 (12), 91 (10), 105 (10), 119 (7), 131 (4), 136 (1), 141 (2), 147 (11), 159 (4), 175 (3), 190 (3), 201 (3), 229 (11), 243 (2), 257 (1), 272 (2). IR (diamond ATR): ṽ = 2955 (m), 2922 (s), 2868 (w), 2853 (w), 1731 (w), 1659 (w), 1632 (w), 1456 (w), 1376 (m), 1259 (m), 1091 (w), 1018 (w), 853 (w), 800 (m) cm^{-1}. HR-MS (Q-TOF, 70 eV): calc. $[C_{20}H_{32}]^+$ *m*/*z* = 272.2499; found: *m*/*z* = 272.2505. Optical rotation: $[\alpha]_D^{25}$ = –115.3 (*c* 0.17, CH_2Cl_2). NMR data are given in Table S14.

Japonene A (22). TLC (pentane): R_f = 0.90. GC (HP-5MS): *I* = 1952. MS (EI, 70 eV): *m*/*z* (%) = 41 (7), 55 (5), 67 (6), 81 (7), 91 (6), 105 (7), 119 (5), 133 (3), 147 (3), 175 (7), 190 (40), 229 (2), 257 (1), 272 (1). IR (diamond ATR): ṽ = 3026 (w), 2953 (s), 2924 (s), 2868 (m), 2846 (m), 1740 (w), 1661 (w), 1632 (w), 1454 (m), 1383 (w), 1371 (w), 1260 (w), 965 (w), 794 (w) cm^{-1}. HR-MS (Q-TOF, 70 eV): calc. $[C_{20}H_{32}]^+$ *m*/*z* = 272.2499; found: *m*/*z* = 272.2508. Optical rotation: $[\alpha]_D^{25}$ = +15.0 (*c* 0.04, acetone). NMR data are given in Table S15.

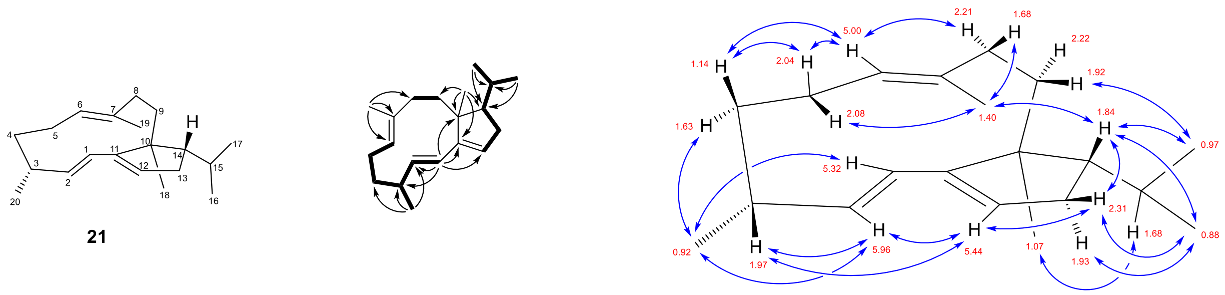

Figure S87. Structure elucidation of neodolabella-1(14),2,7-triene (**21**). Bold: ^{1}H,^{1}H-COSY, single headed arrows: key HMBC, and double headed arrows: NOESY correlations. Carbon numbering follows GGPP numbering to indicate the origin of each carbon.

Table S14. NMR data of neodolabella-1(14),2,7-triene (**21**) in $CDCl_3$ recorded at 238 K.

C[a]	type	^{13}C[b]	^{1}H[b]
1	CH	121.97	5.96 (d, *J* = 16.4)
2	CH	136.93	5.32 (dd, *J* = 9.5, 14.6)
3	CH	41.54	1.96 (m)
4	CH_2	36.42	1.63 (m)
			1.14 (m)
5	CH_2	27.72	2.08 (m)
			2.04 (m)
6	CH	127.17	5.00 (d, *J* = 10.9)
7	C_q	137.24	–
8	CH_2	31.11	2.21 (m)
			1.68 (m)
9	CH_2	39.65	2.22 (m)
			1.92 (m)
10	C_q	50.89	–
11	C_q	148.31	–
12	CH	129.60	5.44 (m)
13	CH_2	34.64	2.31 (m)
			1.93 (m)
14	CH	51.48	1.84 (m)
15	CH	28.89	1.68 (m)
16	CH_3	23.72	0.97 (d, *J* = 6.2)
17	CH_3	23.20	0.88 (d, *J* = 6.9)
18	CH_3	21.52	1.07 (s)
19	CH_3	21.89	1.40 (s)
20	CH_3	22.91	0.92 (d, *J* = 6.7)

[a] Carbon numbering as shown in Figure S87 indicates the origin of each carbon from GGPP by same number. [b] Chemical shifts δ in ppm, multiplicity: s = singlet, d = doublet, m = multiplet, coupling constants *J* are given in Hertz.

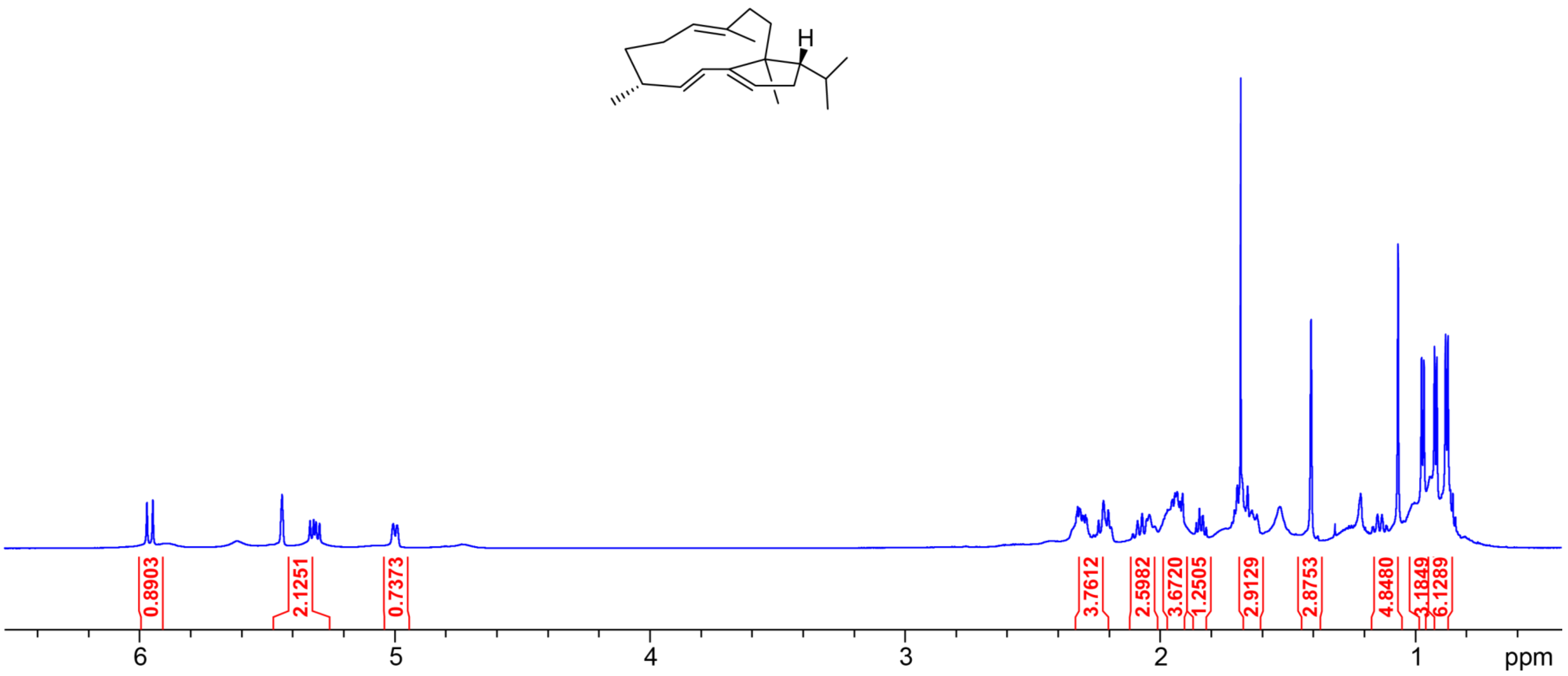

Figure S88. ^{1}H-NMR spectrum of **21** (700 MHz, $CDCl_3$).

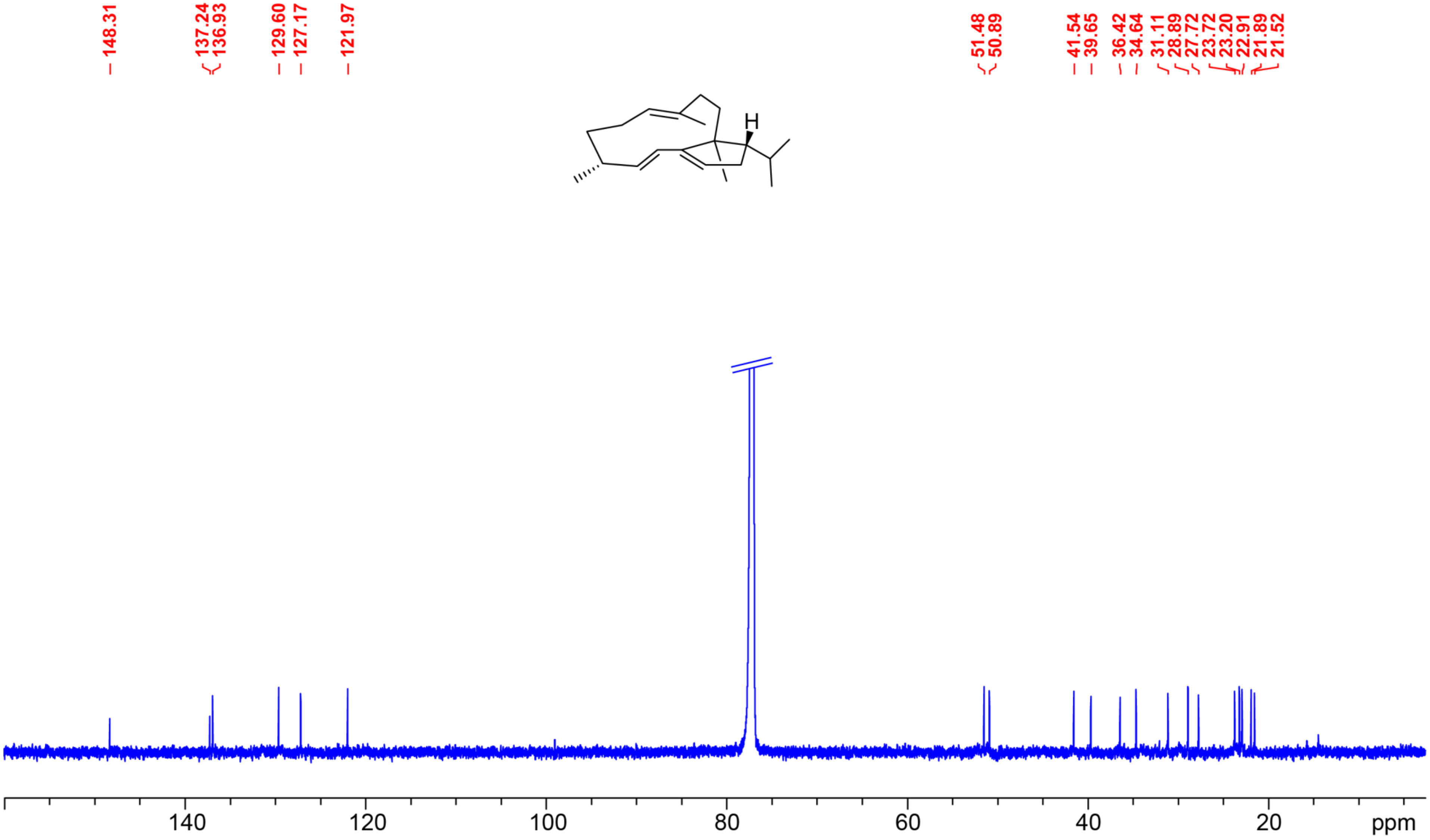

Figure S89. ^{13}C-NMR spectrum of **21** (176 MHz, $CDCl_3$).

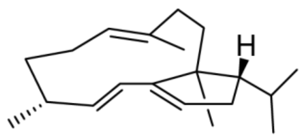

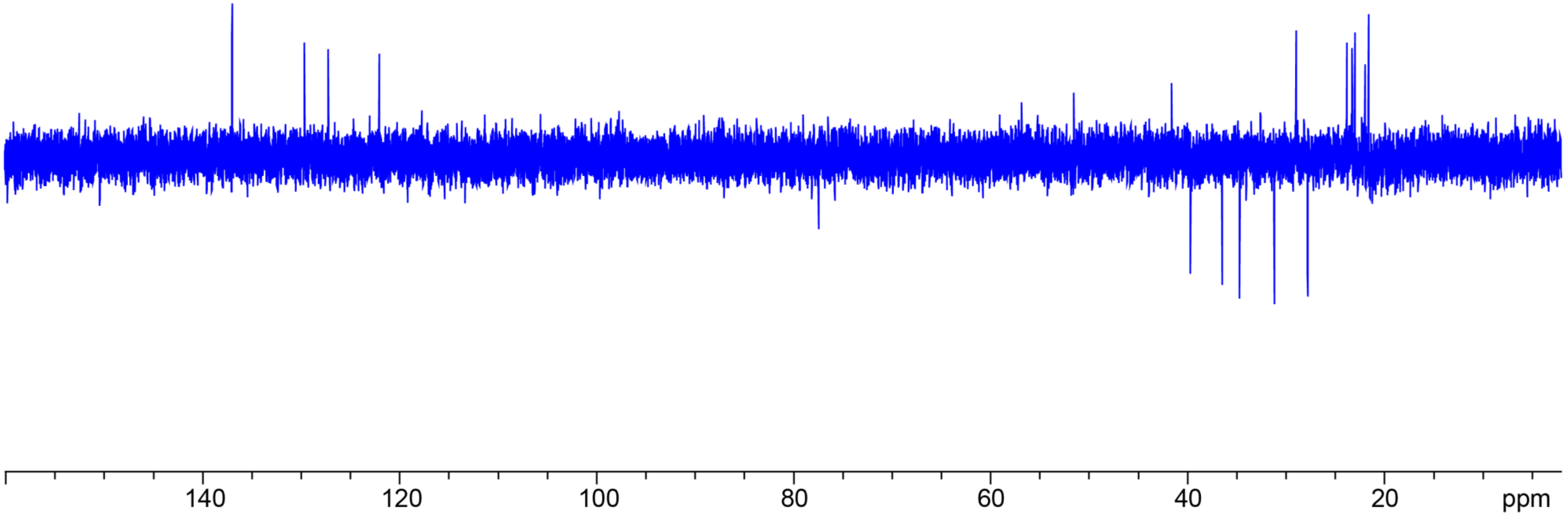

Figure S90. ^{13}C-DEPT135 spectrum of **21** (176 MHz, $CDCl_3$).

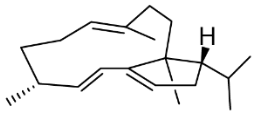

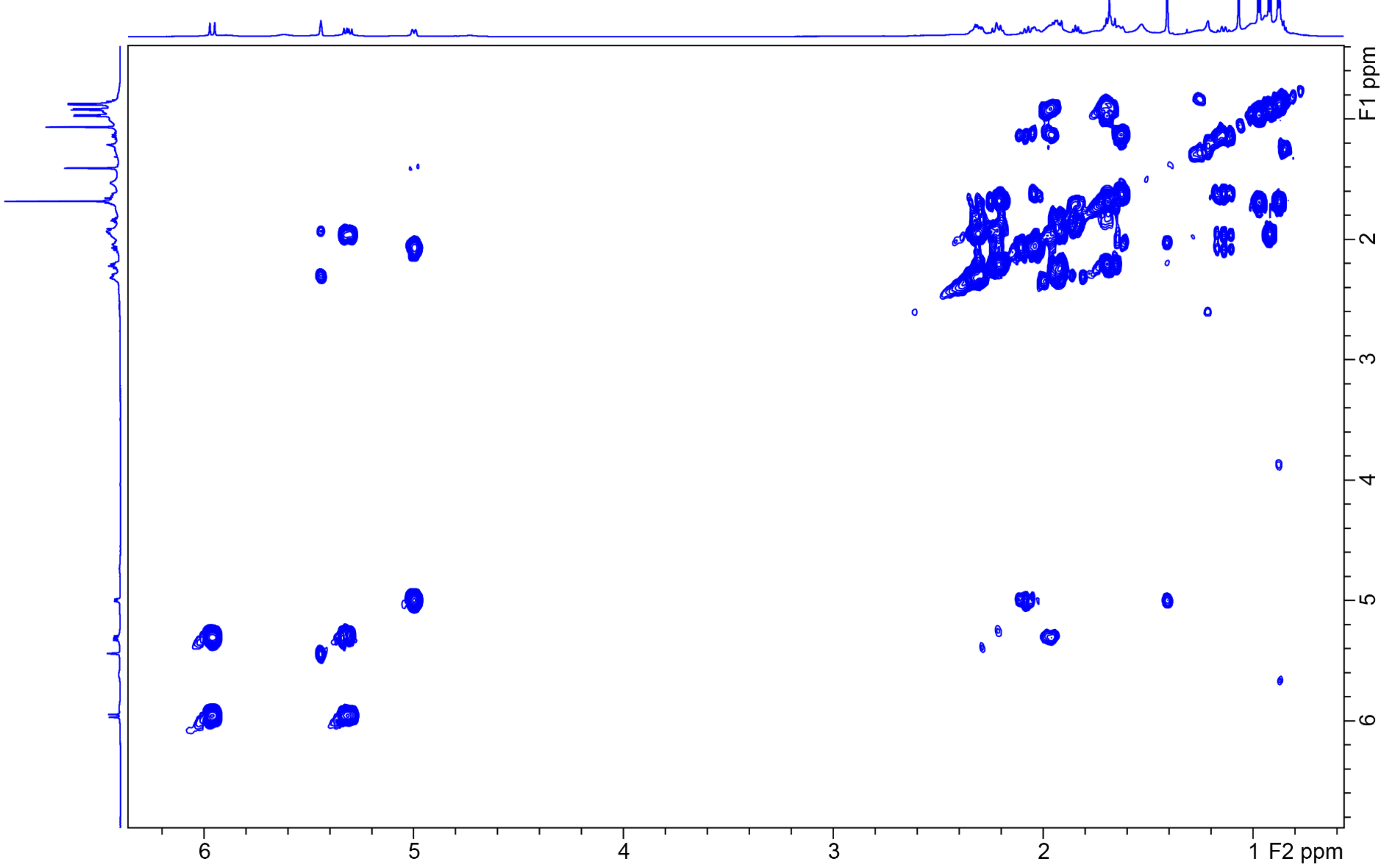

Figure S91. ^{1}H-^{1}H-COSY spectrum ($CDCl_3$) of **21**.

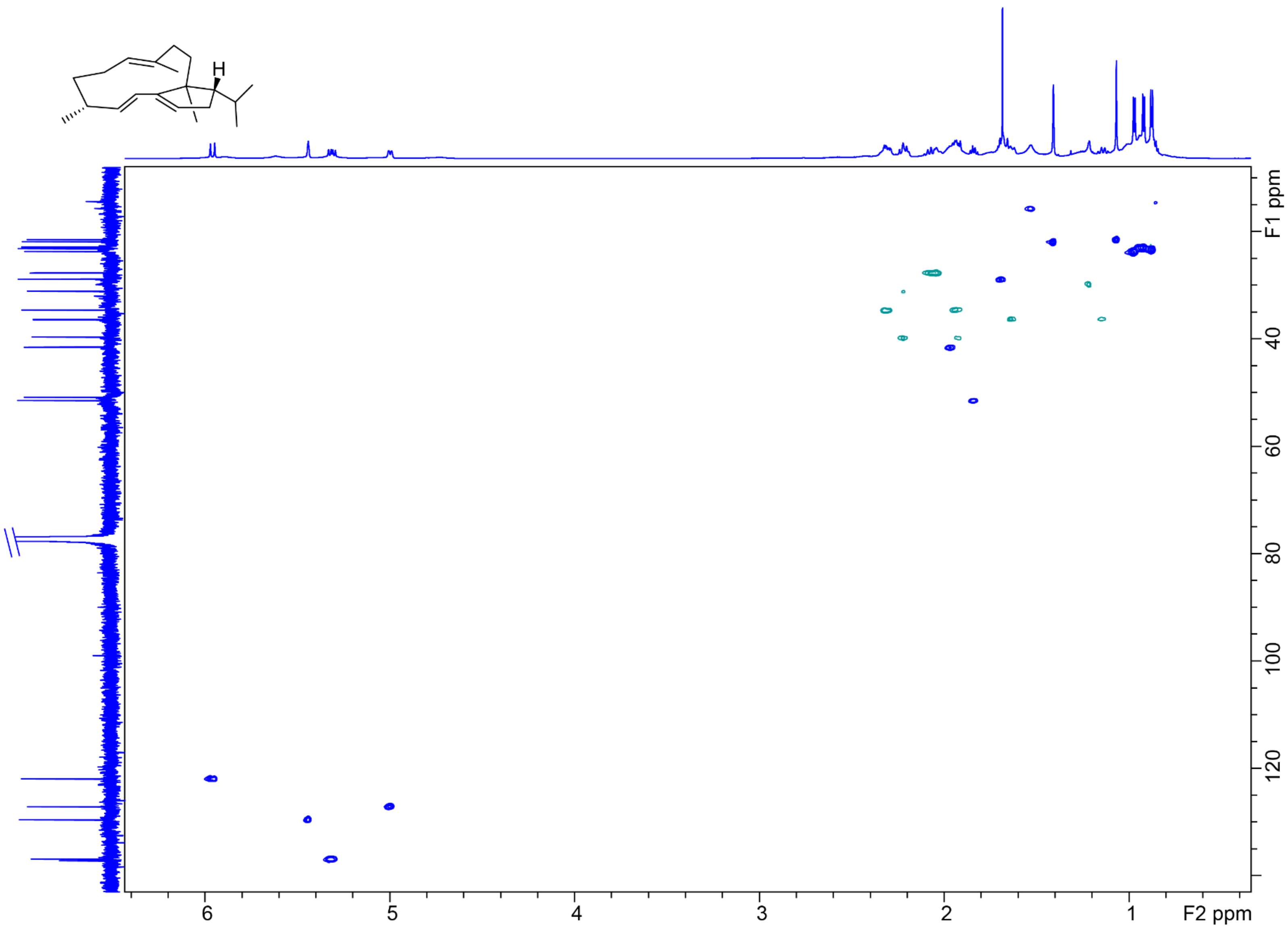

Figure S92. HSQC spectrum ($CDCl_3$) of **21**.

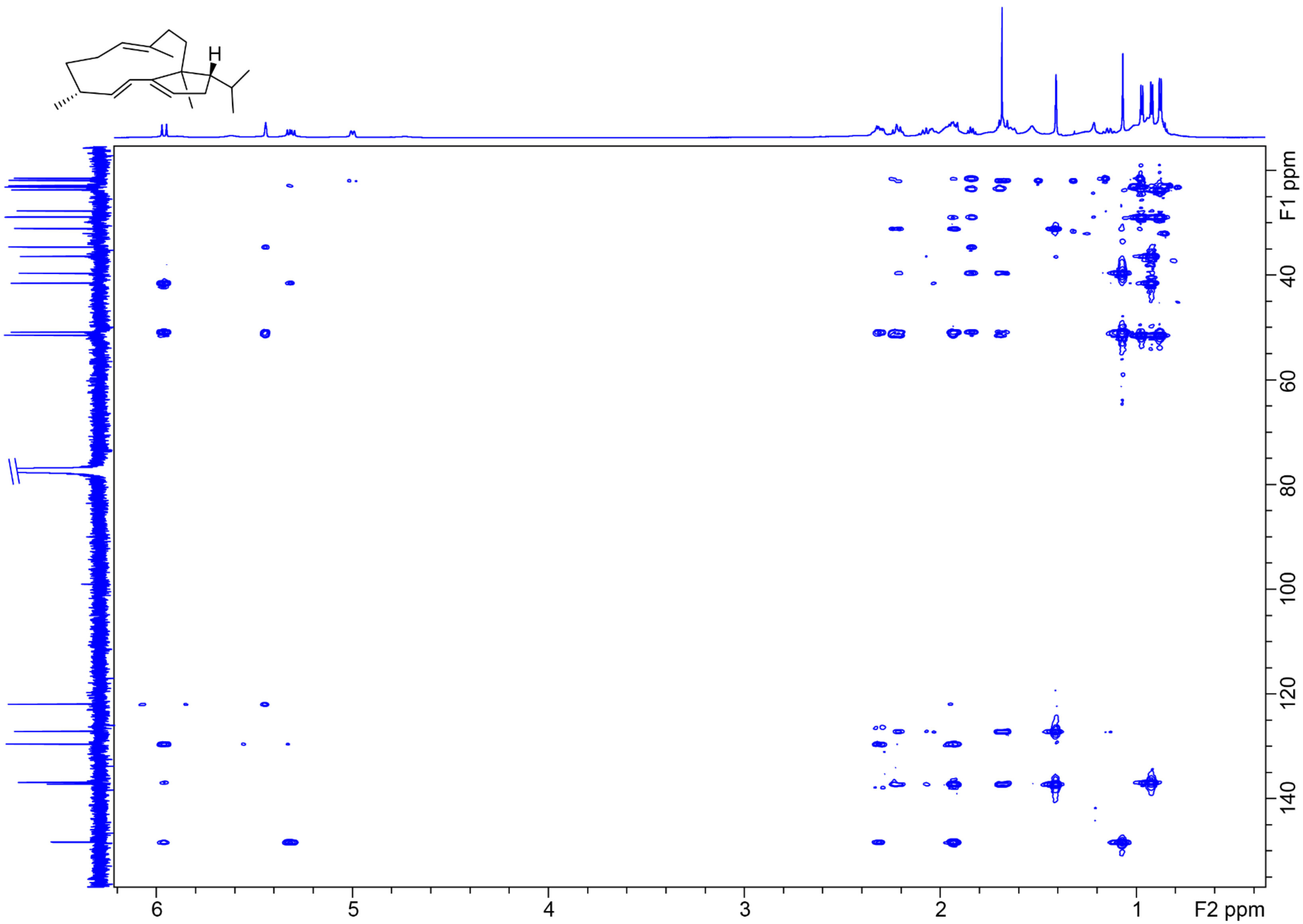

Figure S93. HMBC spectrum ($CDCl_3$) of **21**.

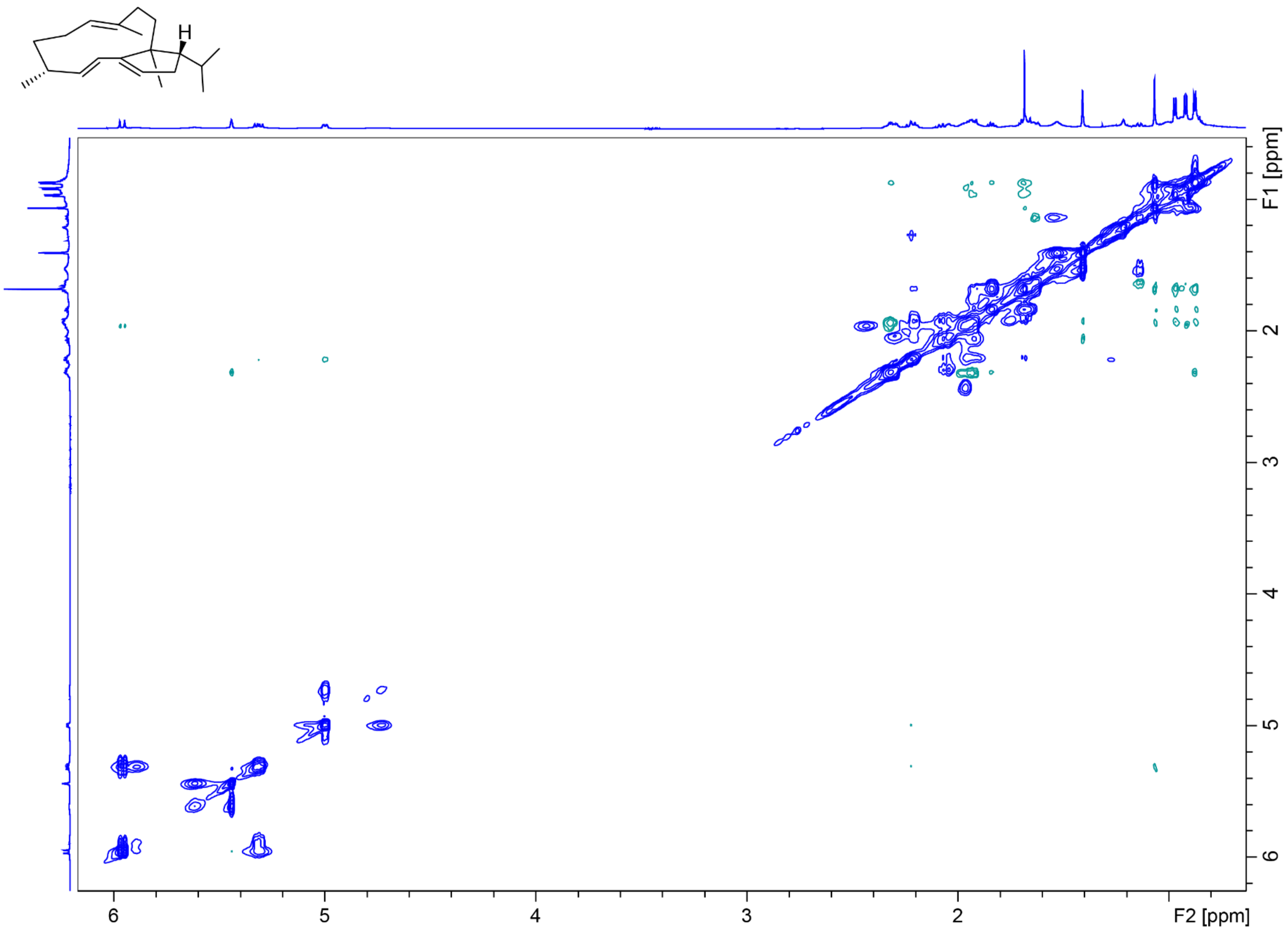

Figure S94. NOESY spectrum ($CDCl_3$) of **21**.

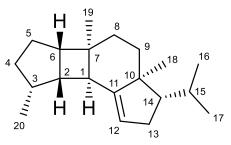

22

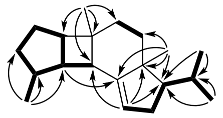

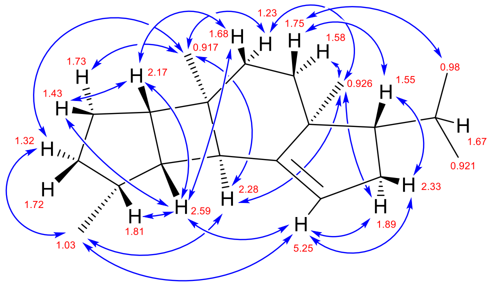

Figure S95. Structure elucidation of japonene A (**22**). Bold: ^{1}H,^{1}H-COSY, single headed arrows: key HMBC, and double headed arrows: NOESY correlations. Carbon numbering follows GGPP numbering to indicate the origin of each carbon.

Table S15. NMR data of japonene A (**22**) in C_6D_6 recorded at 298 K.

C[a]	type	^{13}C[b]	^{1}H[b]
1	CH	37.94	2.28 (br s)
2	CH	49.87	2.59 (ddd, *J* = 6.2, 6.2, 6.2)
3	CH	37.00	1.81 (dddq, *J* = 12.8, 6.5, 6.5, 6.5)
4	CH_2	35.04	1.72 (m)
			1.32 (m)
5	CH_2	27.91	1.73 (m)
			1.43 (m)
6	CH	47.95	2.17 (dd, *J* = 7.7, 7.7)
7	C_q	35.21	–
8	CH_2	35.40	1.68 (m)
			1.23 (m)
9	CH_2	36.61	1.75 (m)
			1.58 (m)
10	C_q	45.65	–
11	C_q	155.81	–
12	CH	120.23	5.25 (m)
13	CH_2	36.20	2.33 (dddd, *J* = 15.2, 7.6, 3.2, 2.1)
			1.89 (dddd, *J* = 15.1, 10.2, 4.8, 1.7)
14	CH	60.48	1.55 (m)
15	CH	29.92	1.67 (m)
16	CH_3	22.95	0.92 (d, *J* = 6.4)
17	CH_3	22.93	0.98 (d, *J* = 6.5)
18	CH_3	21.70	0.926 (s)
19	CH_3	19.20	0.917 (s)
20	CH_3	14.40	1.03 (d, *J* = 6.6)

[a] Carbon numbering as shown in Figure S95 indicates the origin of each carbon from GGPP by same number. [b] Chemical shifts δ in ppm, multiplicity: s = singlet, d = doublet, m = multiplet, br = broad, coupling constants *J* are given in Hertz.

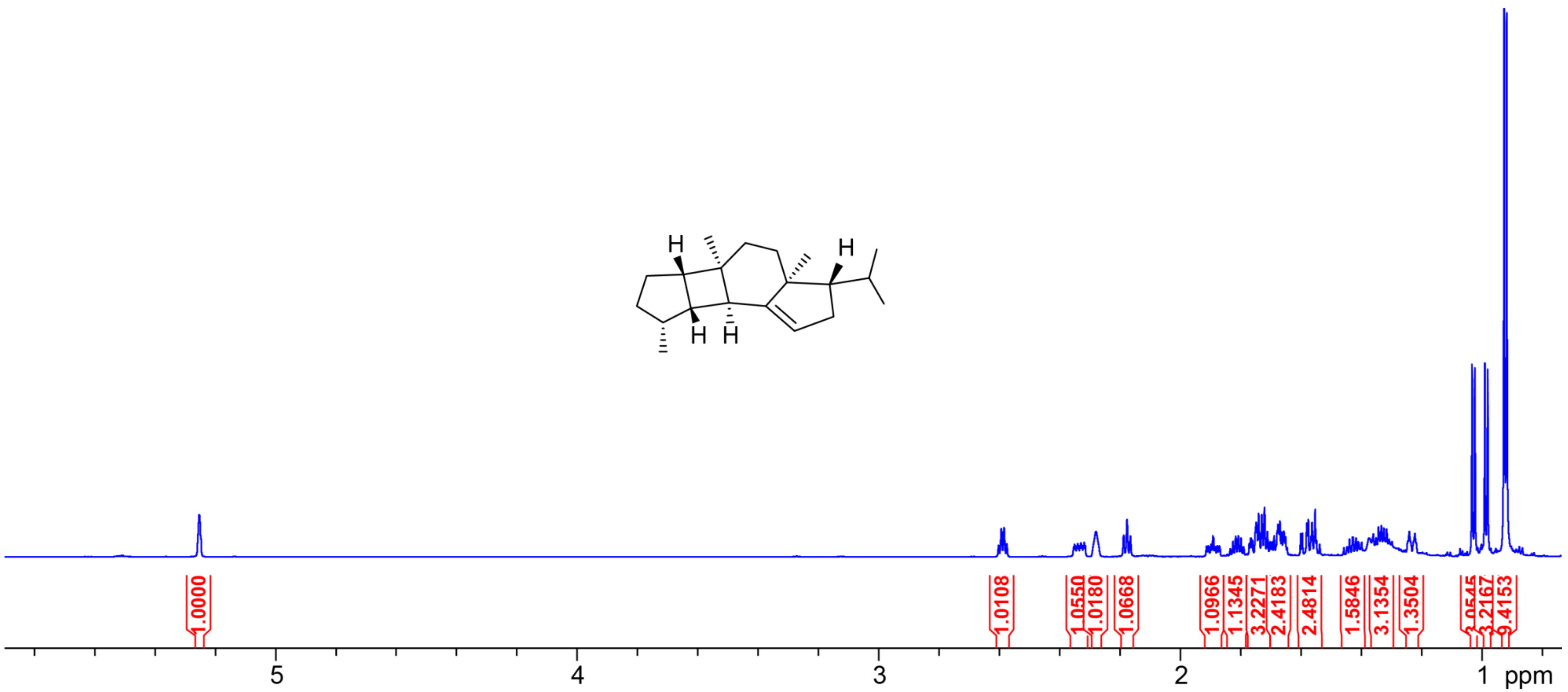

Figure S96. ^{1}H-NMR spectrum of **22** (700 MHz, C_6D_6).

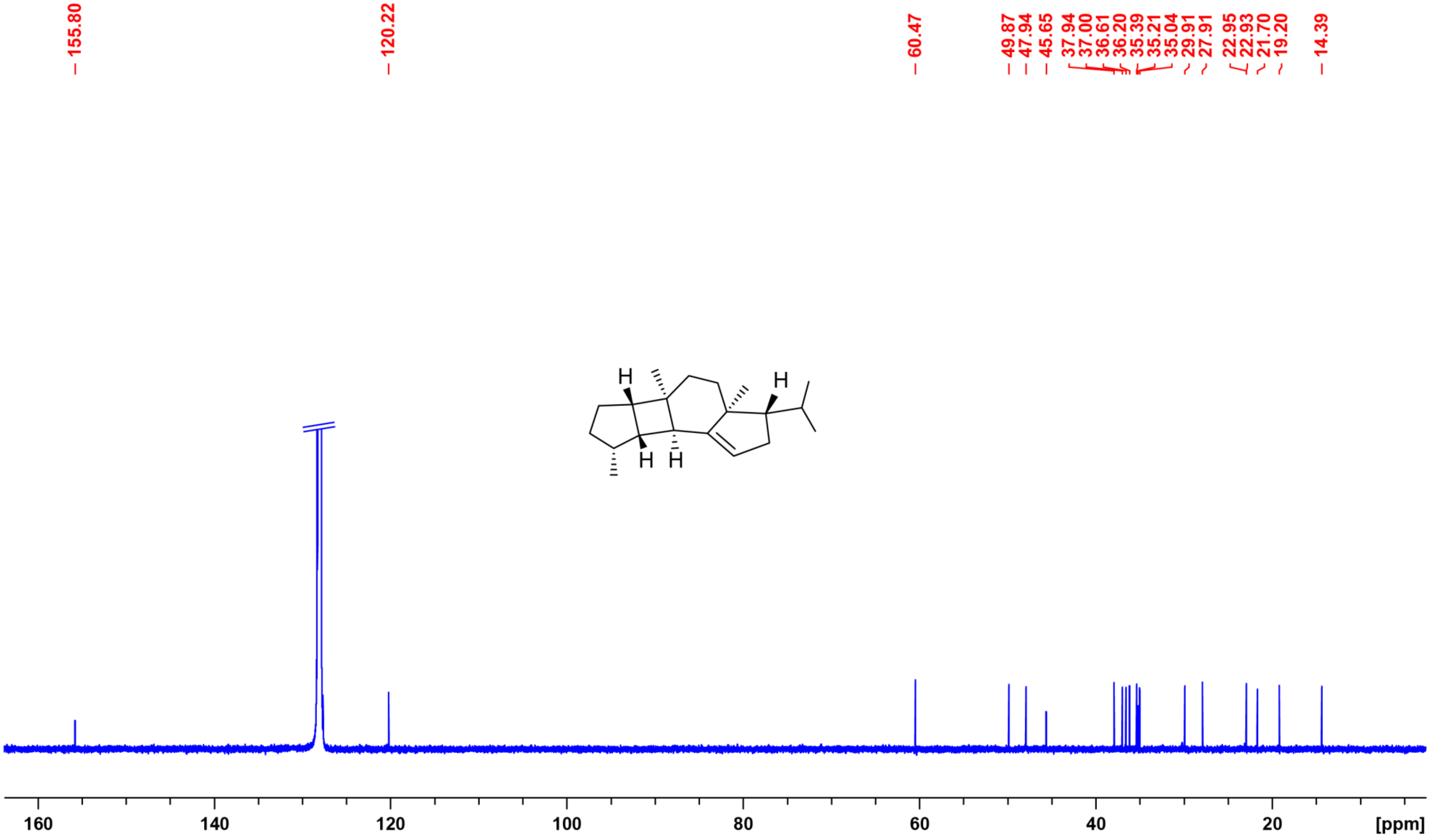

Figure S97. ^{13}C-NMR spectrum of **22** (176 MHz, C_6D_6).

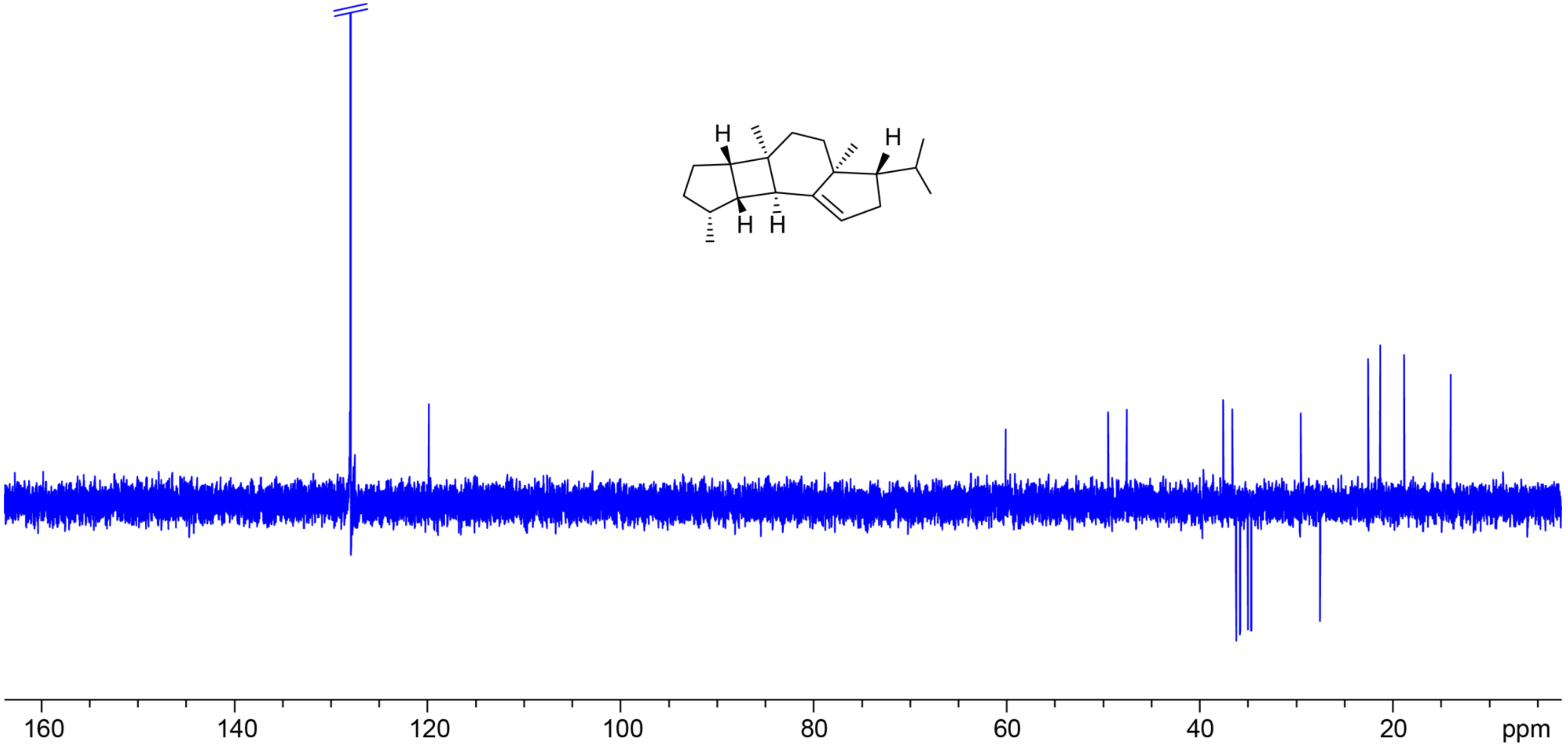

Figure S98. ^{13}C-DEPT135 spectrum of **22** (176 MHz, C_6D_6).

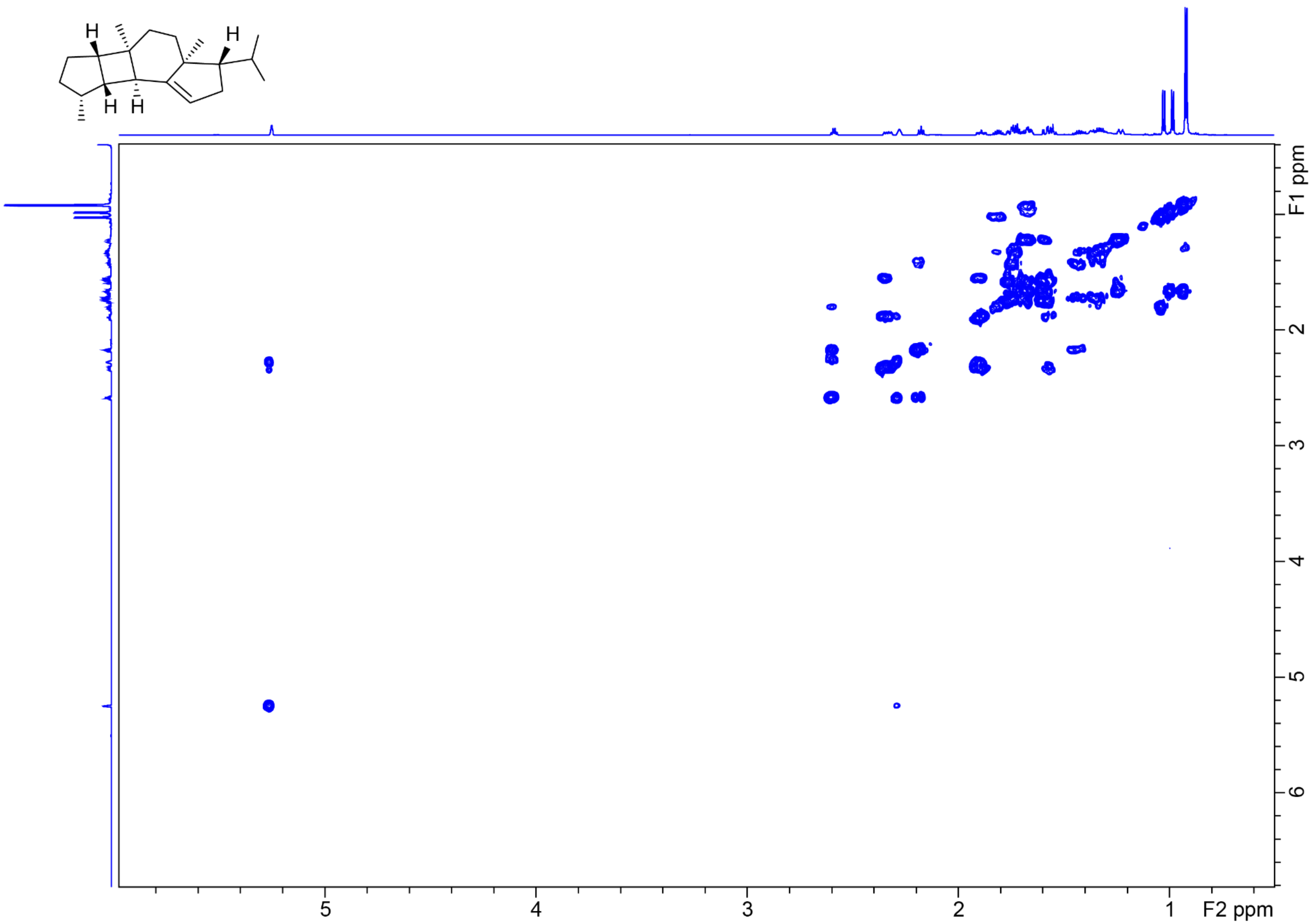

Figure S99. ^{1}H-^{1}H-COSY spectrum (C_6D_6) of **22**.

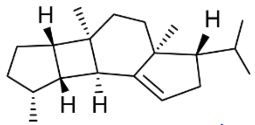

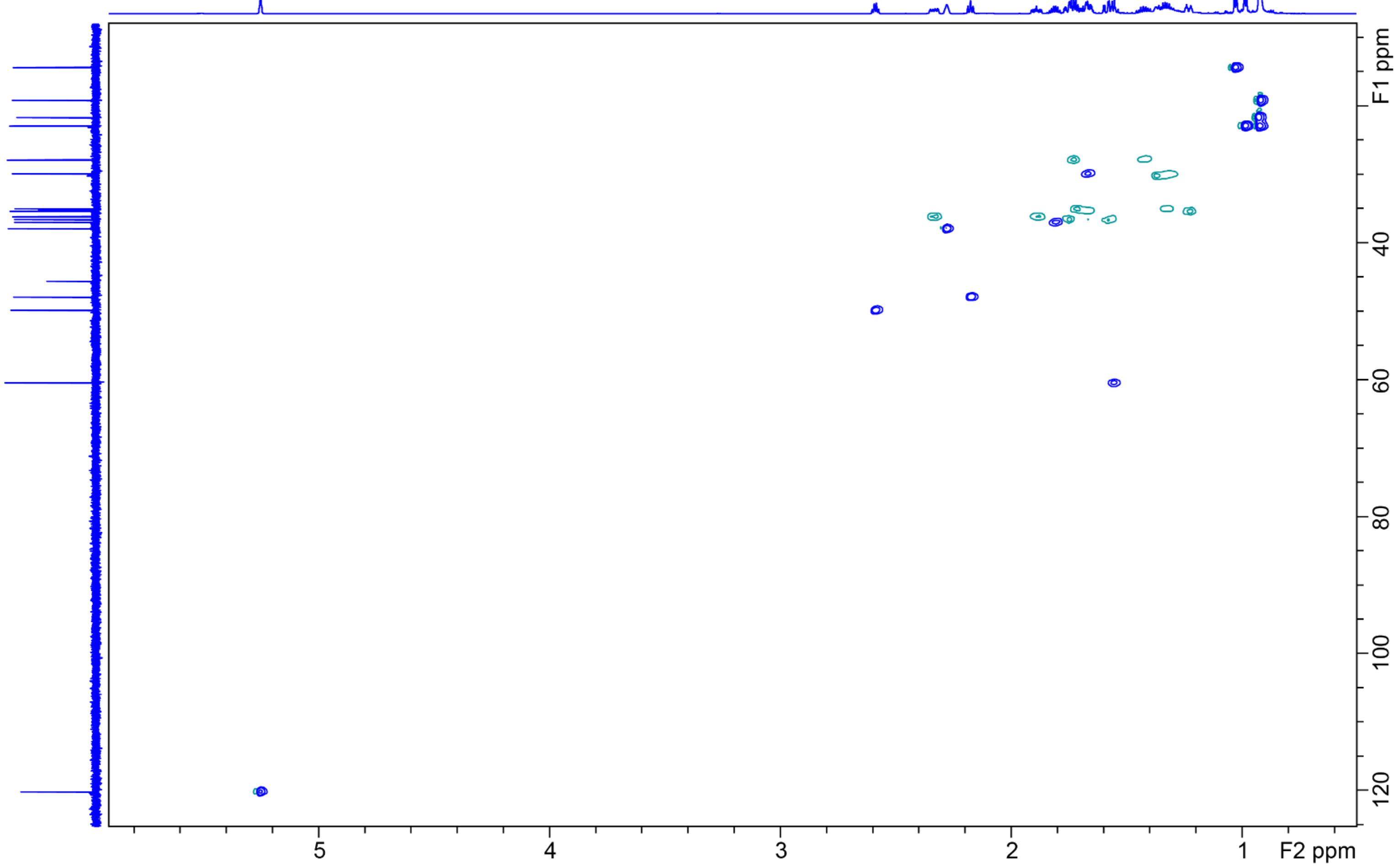

Figure S100. HSQC spectrum (C_6D_6) of **22**.

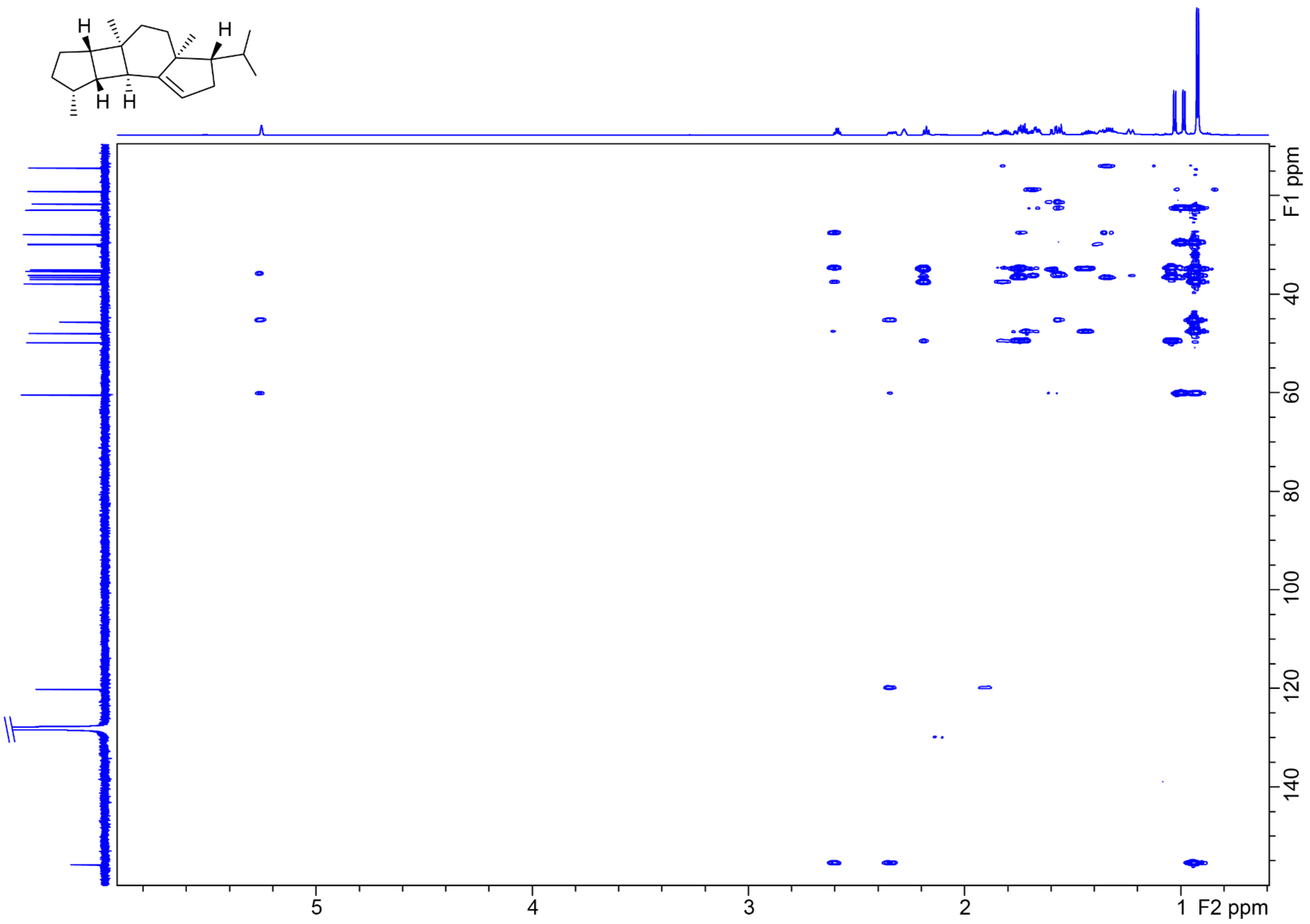

Figure S101. HMBC spectrum (C_6D_6) of **22**.

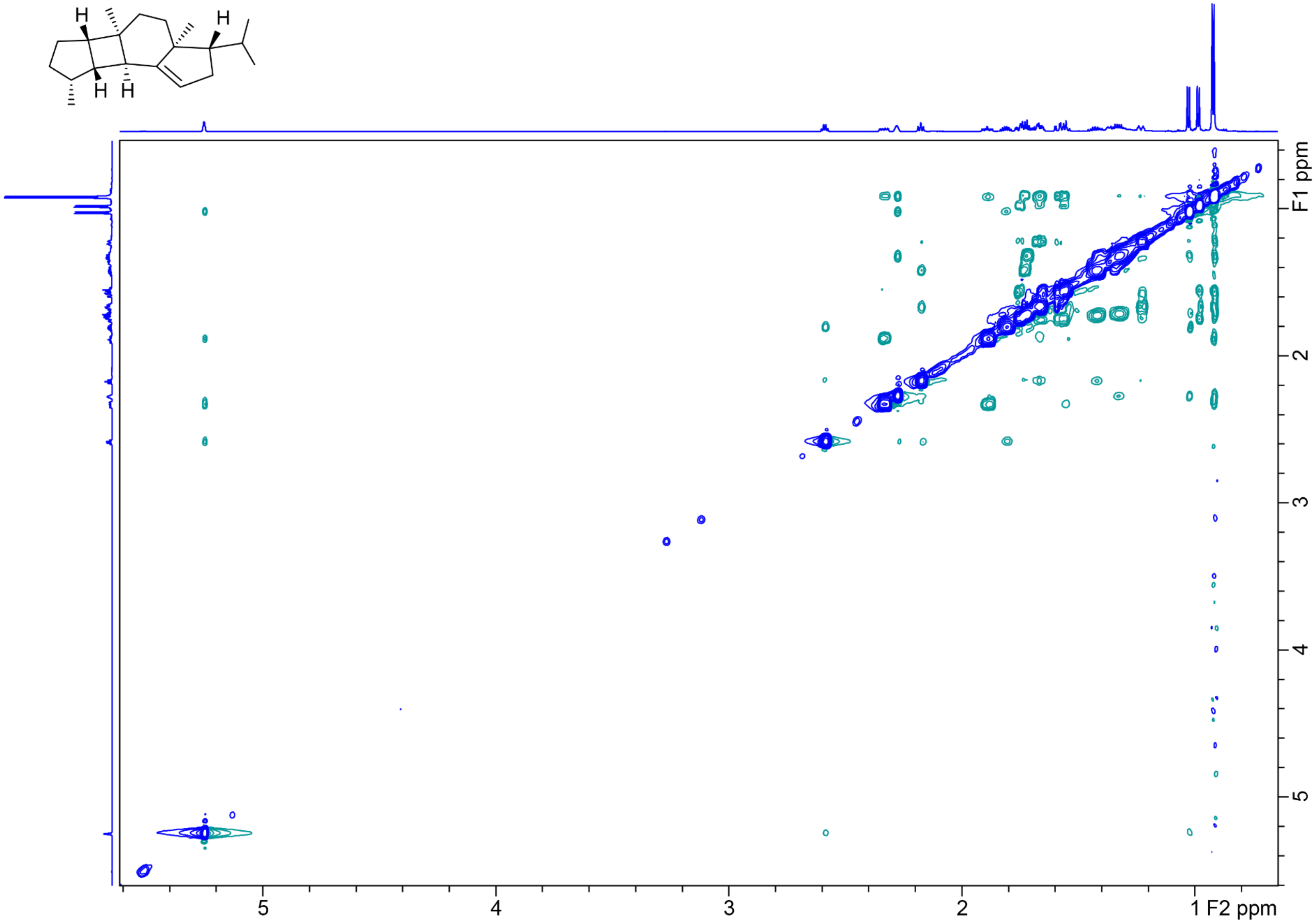

Figure S102. NOESY spectrum (C_6D_6) of **22**.

Thermal reactions with 21

Preparation of 27. Compound **21** (3.5 mg, 12.9 μmol) was dissolved in pentane (50 μL) and the solution was transferred into a sealed tube. Diphenyl ether (0.2 mL) was added. The solution was heated to 230 °C for 5 h, followed by cooling to room temperature. The crude product was purified via column chromatography on silica gel with pentane to obtain pure **27** (0.9 mg, 3.3 μmol, 26%) and reisolated starting material **21** (0.3 mg, 1.1 μmol, 9%).

Preparation of 26. Compound **21** (4.2 mg, 15.4 μmol) was dissolved in pentane (50 μL) and the solution was transferred into a sealed tube. Diphenyl ether (0.2 mL) was added. The solution was heated to 180 °C for 2 h, followed by cooling to room temperature. The crude product was purified via column chromatography on silica gel with pentane to obtain **26** (1.2 mg, 4.4 μmol, 29%).

Preparation of 28. Compound **21** (10.0 mg, 36.8 μmol) was dissolved in pentane (50 μL) and the solution was transferred into a sealed tube. Diphenyl ether (0.2 mL) and water (10 μL) were added. The solution was heated to 230 °C for 9 h, followed by cooling to room temperature. The crude product was purified via column chromatography on silica gel with pentane to obtain **28** (3.4 mg, 12.5 μmol, 34%) and reisolated starting material **21** (3.2 mg, 11.8 μmol, 32%).

Thermal conversion of labelled 21

For thermal conversions of labelled **21** (Table S10, entries 9 – 11), the crude labelled enzyme products obtained from (10,10,10-$^{2}H_3$)GPP and (1-^{13}C)IPP, from (1-^{13}C)GGPP, or from (*S*)-(1-^{13}C,1-^{2}H)GGPP, respectively, were extracted from incubation experiments with C_6D_6 (50 μL) for NMR measurements. After confirmation of the incorporation of labelling into **21**, the solutions were transferred into a sealed tube. Diphenyl ether (0.2 mL) was added. Only for the conversion of labelled **21** obtained from (1-^{13}C)GGPP one drop of D_2O (ca. 10 μL) was added. The mixtures were heated to 230 °C for 5 h and cooled to room temperature. To remove Ph_2O, the solutions were filtered through a short pad of silica gel using pentane for compound elution. The solvent was evaporated, the samples were dissolved in C_6D_6 (0.5 mL) and analysed by GC/MS and NMR, showing the formation of labelled products (**26** and **27**; Figures S111 – S113).

Thermocyclene B (27). TLC (pentane): R_f = 0.83. GC (HP-5MS): *I* = 1935. MS (EI, 70 eV): *m*/*z* (%) = 43 (6), 55 (3), 67 (3), 81 (6), 91 (10), 105 (7), 115 (2), 121 (5), 133 (6), 145 (4), 159 (2), 175 (4), 187 (3), 201 (10), 202 (9), 229 (7), 230 (8), 243 (3), 257 (1), 272 (0.4). IR (diamond ATR): ṽ = 2952 (s), 2926 (s), 2868 (m), 1727 (w), 1648 (w), 1411 (w), 1379 (w), 1259 (s), 1091 (s), 1015 (s), 875 (w), 796 (s), 701 (w), 692 (w) cm^{-1}. HR-MS (Q-TOF, 70 eV): calc. $[C_{20}H_{32}]^+$ *m*/*z* = 272.2499; found: *m*/*z* = 272.2490. Optical rotation: $[\alpha]_D^{25}$ = –27.5 (*c* 0.04, CH_2Cl_2). NMR data are given in Table S16.

Thermocyclene A (26). TLC (pentane): R_f = 0.79. GC (HP-5MS): *I* = 1977. MS (EI, 70 eV): *m*/*z* (%) = 41 (2), 55 (2), 67 (2), 81 (9), 91 (6), 105 (7), 119 (6), 136 (8), 147 (13), 159 (4), 175 (4), 190 (4), 201 (4), 215 (1), 229 (20), 243 (3), 257 (1), 272 (4). IR (diamond ATR): ṽ = 3367 (br), 2954 (s), 2923 (s), 2853 (s), 1735 (w), 1657 (w), 1633 (w), 1463 (w), 1377 (w), 1259 (w), 1090 (w), 1015 (w), 797 (w). HR-MS (Q-TOF, 70 eV): calc. $[C_{20}H_{32}]^+$ *m*/*z* = 272.2499; found: *m*/*z* = 272.2506. Optical rotation: $[\alpha]_D^{25}$ = –40.0 (*c* 0.02, CH_2Cl_2). NMR data are given in Table S17.

Thermocyclene C (28). TLC (pentane): R_f = 0.83. GC (HP-5MS): *I* = 1885. MS (EI, 70 eV): *m*/*z* (%) = 41 (3), 55 (3), 67 (2), 81 (6), 91 (5), 105 (5), 119 (7), 133 (3), 145 (3), 159 (2), 173 (1), 187 (1), 201 (2), 215 (0.4), 229 (1), 243 (47), 257 (4), 272 (4). IR (diamond ATR): ṽ = 2952(s), 2927 (s), 2867 (m), 1457 (w), 1379 (w), 1360 (w), 1314 (w), 1261 (w), 1204 (w), 1100 (w), 1051 (w), 1018 (w), 803 (w). HR-MS (Q-TOF, 70 eV): calc. $[C_{20}H_{32}]^+$ *m*/*z* = 272.2499; found: *m*/*z* = 272.2485. Optical rotation: $[\alpha]_D^{25}$ = +10.0 (*c* 0.19, CH_2Cl_2). NMR data are given in Table S18.

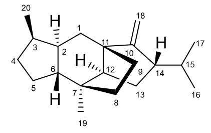

27

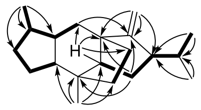

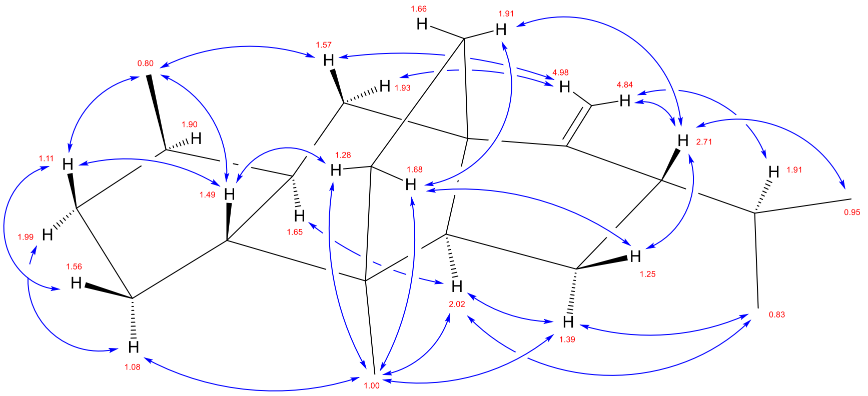

Figure S103. Structure elucidation of thermocyclene B (**27**). Bold: $^{1}H,^{1}H$-COSY, single headed arrows: key HMBC, and double headed arrows: NOESY correlations. Carbon numbering follows GGPP numbering to indicate the origin of each carbon.

Table S16. NMR data of thermocyclene B (**27**) in C_6D_6 recorded at 298 K.

C[a]	type	^{13}C[b]	^{1}H[b]
1	CH_2	36.98	1.93 (m)
			1.57 (m)
2	CH	43.15	1.65 (m)
3	CH	30.96	1.90 (m)
4	CH_2	33.54	1.99 (m)
			1.11 (m)
5	CH_2	27.65	1.56 (m)
			1.08 (m)
6	CH	51.55	1.49 (m)
7	C_q	43.46	–
8	CH_2	41.45	1.68 (m)
			1.28 (m)
9	CH_2	42.67	1.91 (m)
			1.66 (m)
10	C_q	165.63	–
11	C_q	56.50	–
12	CH	52.38	2.02 (dt, J = 10.8, 9.0)
13	CH_2	24.28	1.39 (m)
			1.25 (m)
14	CH	50.29	2.71 (m)
15	CH	31.77	1.91 (m)
16	CH_3	17.35	0.83 (d, J = 6.9)
17	CH_3	22.16	0.95 (d, J = 6.9)
18	CH_2	102.39	4.98 (t, J = 2.2)
			4.84 (t, J = 2.1)
19	CH_3	19.28	1.00 (s)
20	CH_3	18.57	0.80 (d, J = 6.9)

[a] Carbon numbering as shown in Figure S103 indicates the origin of each carbon from GGPP by same number. [b] Chemical shifts δ in ppm, multiplicity: s = singlet, d = doublet, t = triplet, m = multiplet, br = broad, coupling constants *J* are given in Hertz.

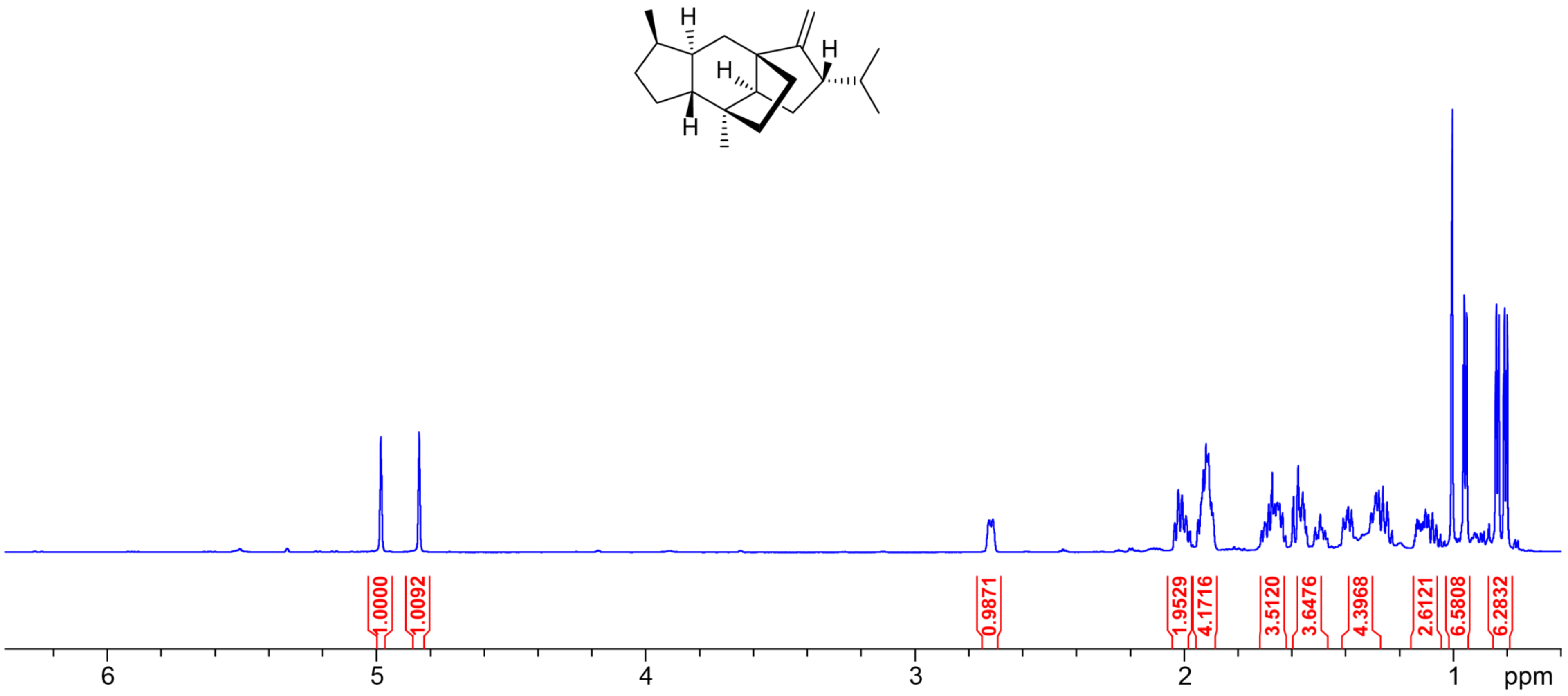

Figure S104. ^{1}H-NMR spectrum of **27** (700 MHz, C_6D_6).

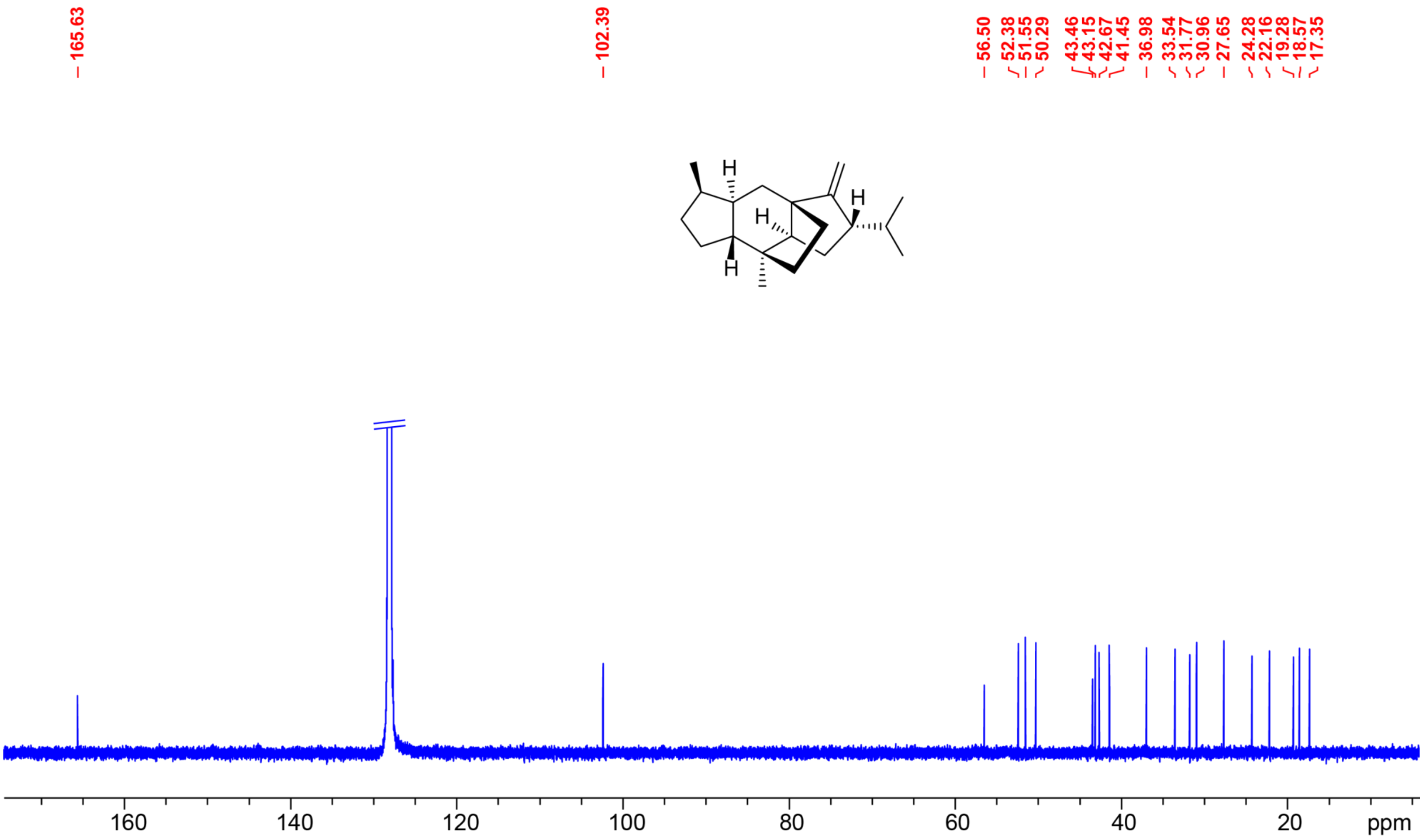

Figure S105. ^{13}C-NMR spectrum of **27** (176 MHz, C_6D_6).

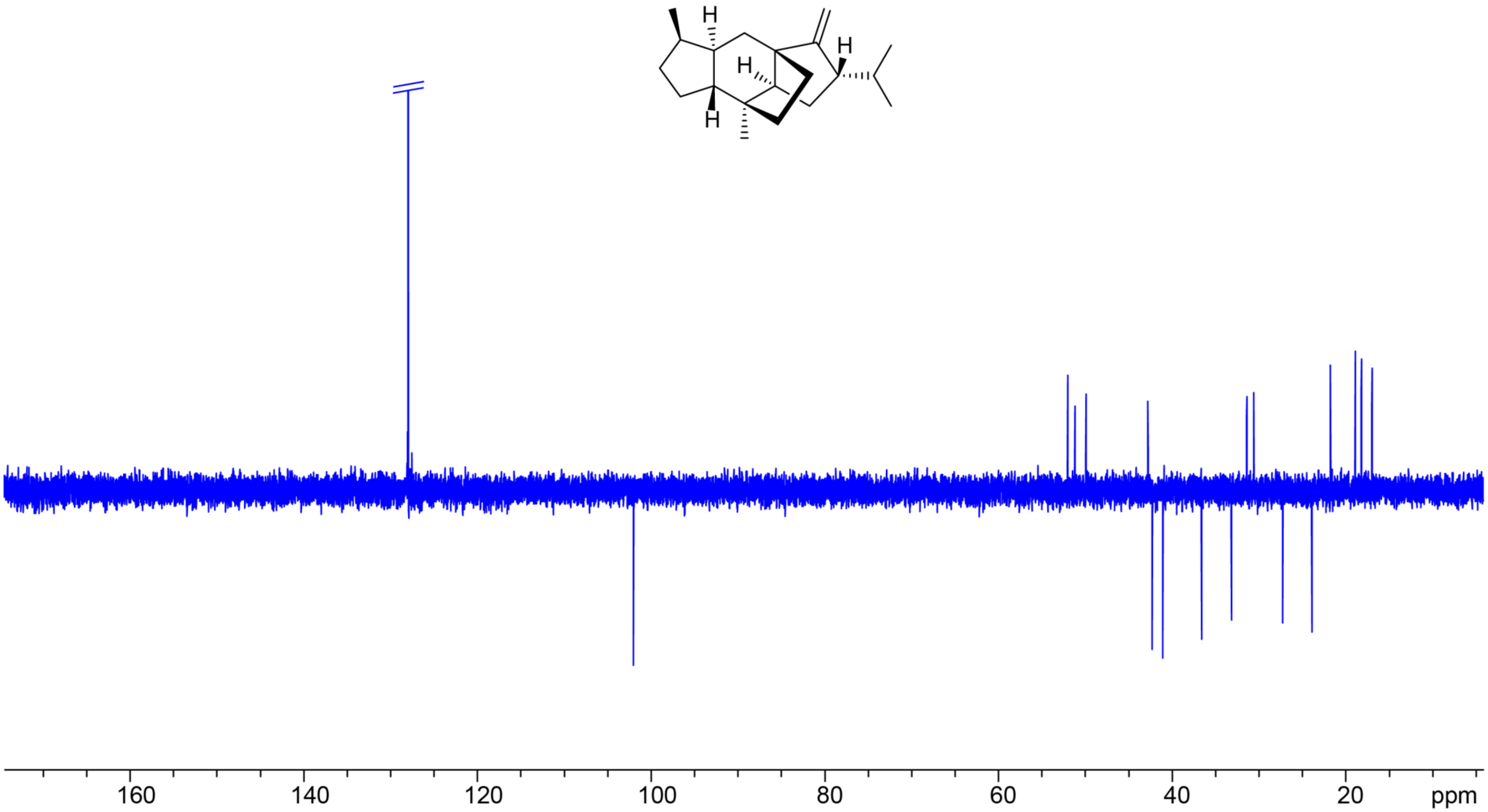

Figure S106. ^{13}C-DEPT135 spectrum of **27** (176 MHz, C_6D_6).

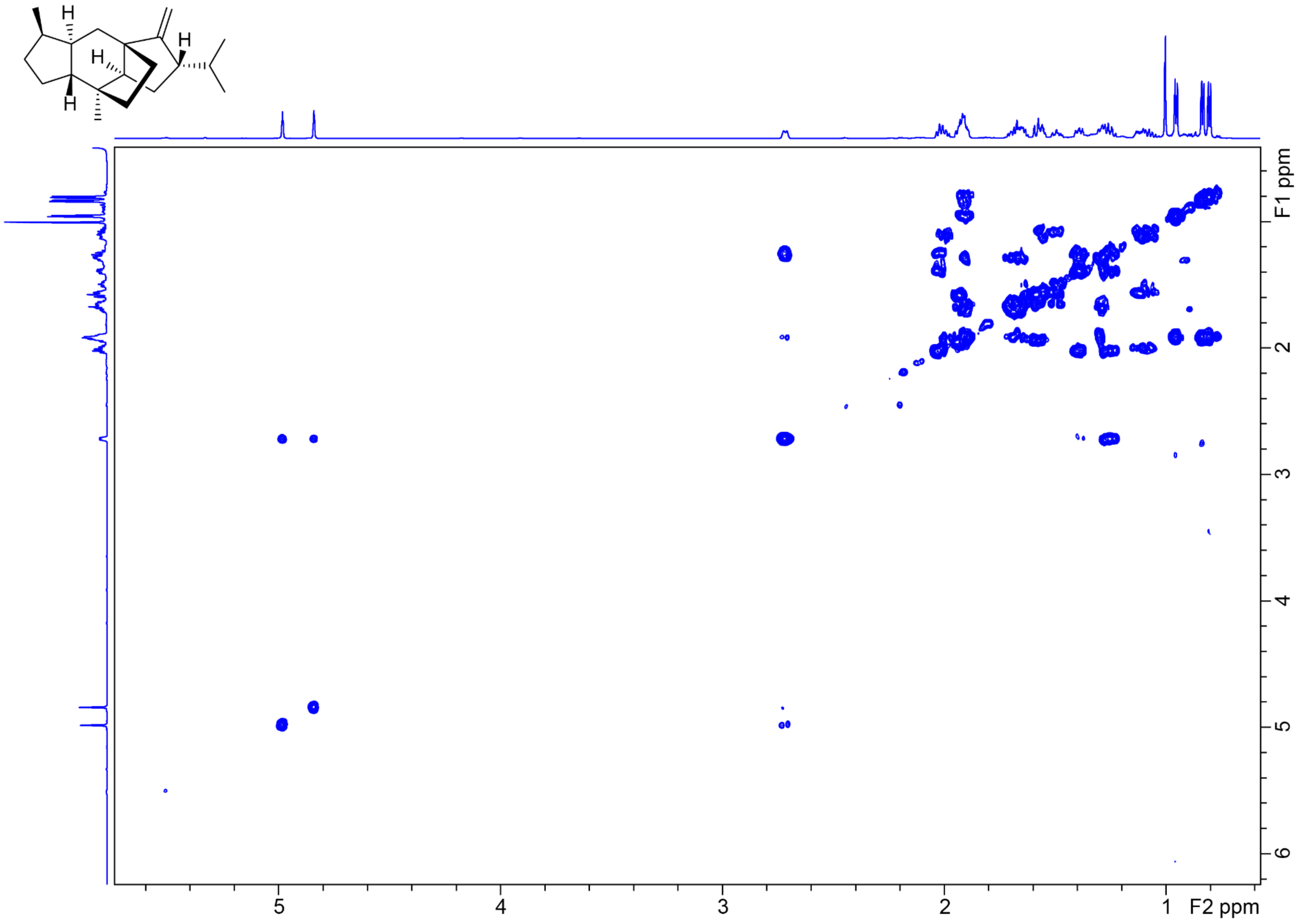

Figure S107. ^1H-^1H-COSY spectrum (C_6D_6) of **27**.

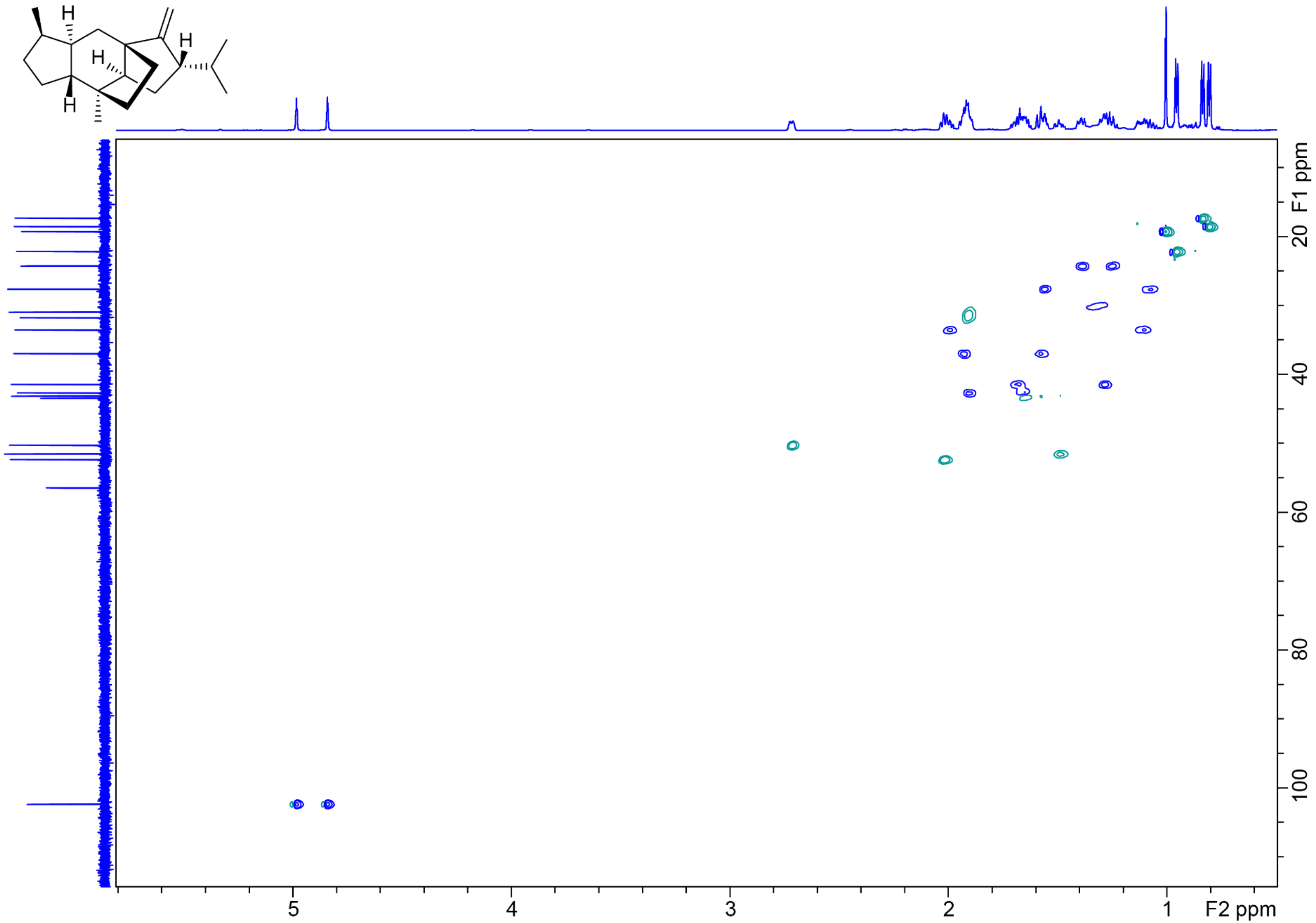

Figure S108. HSQC spectrum (C_6D_6) of **27**.

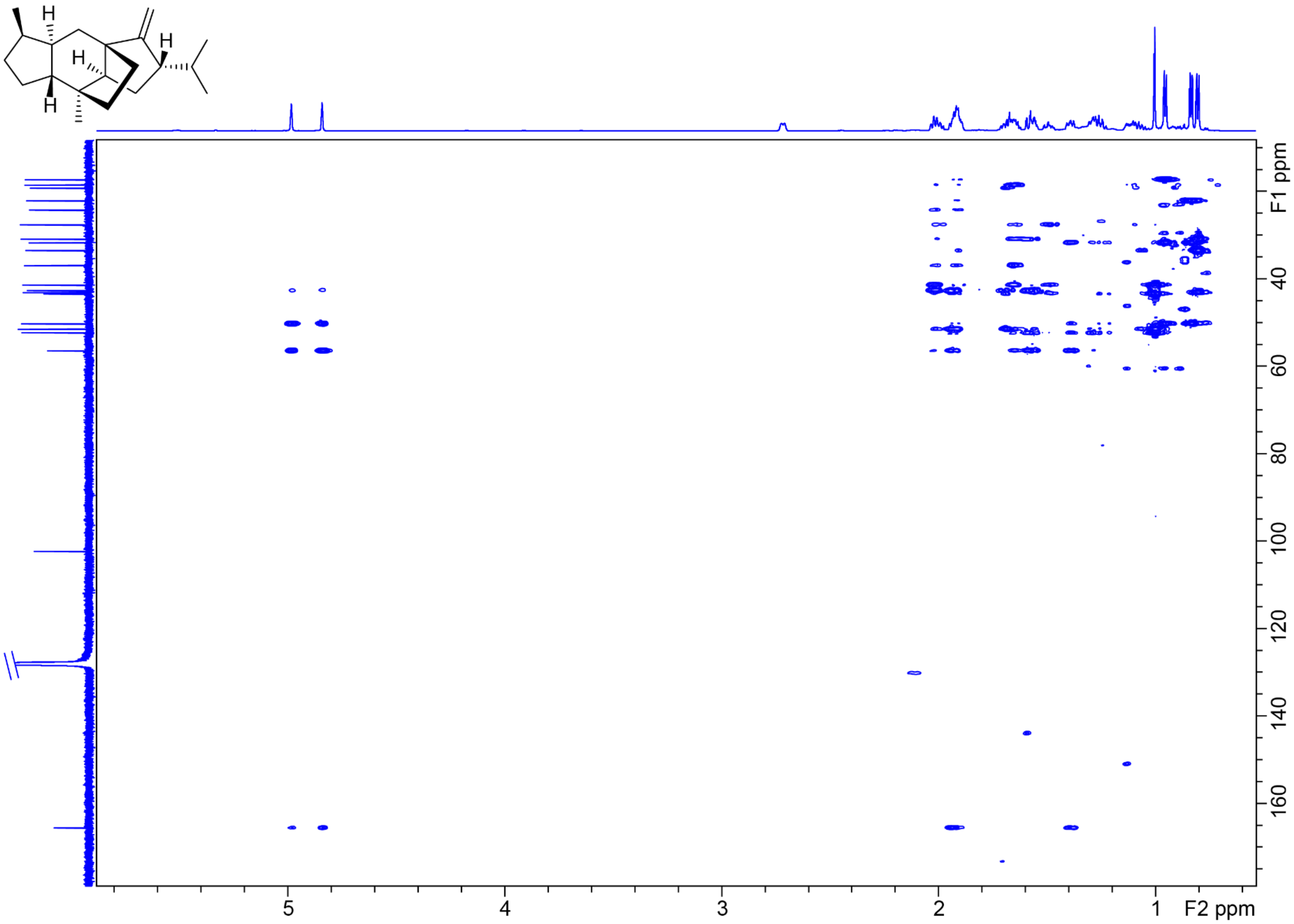

Figure S109. HMBC spectrum (C_6D_6) of **27**.

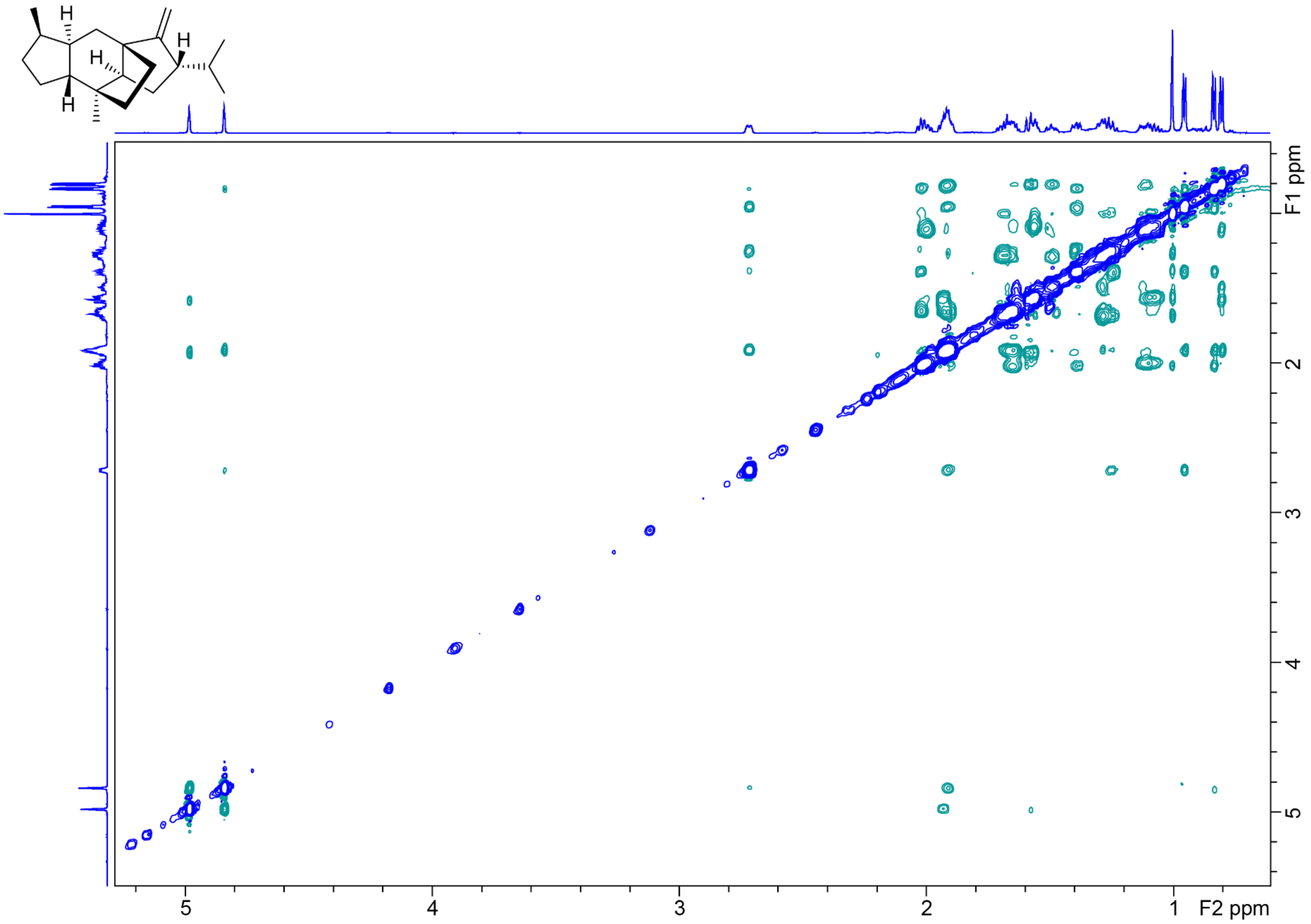

Figure S110. NOESY spectrum (C_6D_6) of **27**.

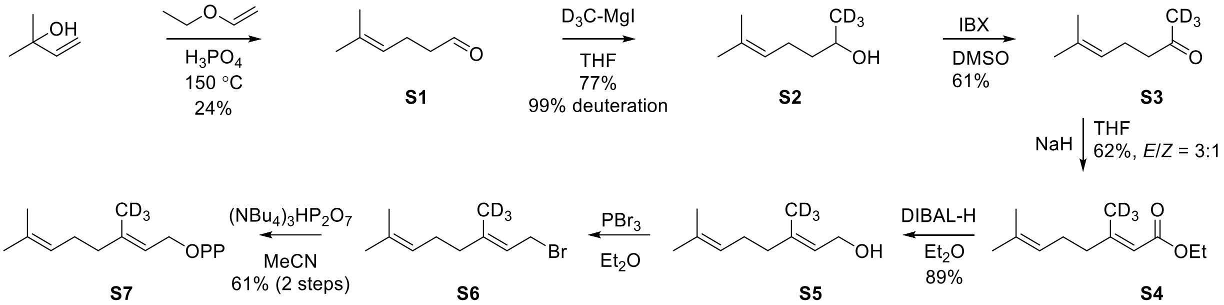

Scheme S1. Synthesis of (10,10,10-$^2H_3$)GPP.

Synthesis of (1,1,1-$^2H_3$)-6-methylhept-5-en-2-ol (S2)

Mg turnings (542 mg, 22.6 mol, 4.20 eq) were suspended in dry THF (20 mL) under Ar atmosphere, followed by the dropwise addition of ($^2H_3$)MeI (1.46 g, 10.3 mmol, 1.90 eq). The reaction mixture started refluxing and was stirred for 1 h. After the addition of aldehyde **S1** (600 mg, 5.36 mmol, 1.00 eq), synthesised following a reported procedure,[35] the reaction mixture was stirred overnight at room temperature. Sat. NH_4Cl (20 mL) was added to quench the reaction and the mixture was extracted with Et_2O (3x 50 mL). The combined organic extracts were dried over $MgSO_4$ and concentrated by evaporation of the solvents. The crude product was purified by column chromatography (petroleum ether: Et_2O = 2:1) to yield the alcohol **S2** (540 mg, 4.1 mmol, 77%) as a colourless oil.

TLC (petroleum ether: Et_2O = 2:1): R_f = 0.35. GC (HP-5): I = 1082. ^{1}H-NMR (C_6D_6, 500 MHz): δ = 5.17 (ddq, 1H, J = 8.7, 5.8, 1.4 Hz), 3.54 (dd, 1H, J = 9.5, 3.4 Hz), 2.05 (m, 2H), 1.65 (s, 3H), 1.55 (3H) ppm. ^{13}C-NMR (C_6D_6, 125 MHz): δ = 131.46 (C_q), 124.97 (CH), 67.72 (CH), 39.64 (CH_2), 25.85 (CH_3), 24.89 (CH_2), 17.69 (CH_3) ppm.

Synthesis of (1,1,1-$^2H_3$)-6-methylhept-5-en-2-one (S3)

To a solution of IBX (2.98 g, 10.69 mmol, 1.40 eq) in DMSO (25 mL) was slowly added **S2** (1.0 g, 7.63 mmol, 1.00 eq) and the reaction mixture was stirred for 2 h at room temperature. The reaction was monitored by TLC analysis. After completion of the reaction, Et_2O (30 mL) was added under ice cooling, followed by washing with sat. NH_4Cl (20 mL). After extraction with Et_2O (3x 50 mL), the combined organic layers were dried with $MgSO_4$ and concentrated under reduced pressure. Purification by column chromatography (petroleum ether: Et_2O = 8:1) yielded the ketone **S3** (605 mg, 4.69 mmol, 61%) as a pale yellow oil.

TLC (petroleum ether: Et_2O = 8:1): R_f = 0.45. ^{1}H-NMR (C_6D_6, 500 MHz): δ = 5.06 (ddq, 1H, J = 8.6, 5.8, 1.4 Hz), 2.21 (m, 2H), 1.99 (m, 2H), 1.61 (d, 3H, J = 1.4 Hz), 1.51 (br s, 3H) ppm. ^{13}C-NMR (C_6D_6, 125 MHz): δ = 205.93 (C_q), 131.13 (C_q), 123.76 (CH), 43.39 (CH_2), 25.76 (CH_3), 22.84 (CH_2), 17.62 (CH_3) ppm.

Synthesis of ethyl (*E*)-7-methyl-3-(methyl-d$_3$)octa-2,6-dienoate (S4)

To a cooled (0 °C) solution of triethyl phosphonoacetate (2.28 g, 10.2 mmol, 2.20 eq) in THF (10 mL) was added NaH (368 mg, 9.2 mmol, 2.00 eq, 60% in mineral oil) in small portions. After stirring the reaction mixture for 1 h at 0 °C, the reaction mixture was cooled to –78 °C and the methyl ketone **S3** (600 mg, 4.6 mmol, 1.00 eq) was added. The reaction mixture was allowed to warm to room temperature and stirred overnight. Water (15 mL) was added to quench the reaction. The aqueous phase was extracted with Et_2O (3x 50 mL), and the combined organic layers were dried with $MgSO_4$ and concentrated under reduced pressure. Purification by column chromatography (petroleum ether: Et_2O = 7:1) yielded pure (*E*)-**S4** (450 mg, 2.26 mmol, 49%) and (*Z*)-**S4** (120 mg, 0.6 mmol, 13%) as colourless oils.

(*E*)-**S4**: TLC (petroleum ether: Et_2O = 7:1): R_f = 0.63. GC (HP-5): I = 1395. ^{1}H-NMR (C_6D_6, 500 MHz): δ = 5.83 (m, 1H), 5.02 (ddq, 1H, J = 8.5, 5.6, 1.4 Hz), 4.04 (q, 2H, J = 7.2 Hz), 1.98 (m, 2H), 1.90 (m, 2H), 1.60 (br s, 3H), 1.45 (br s, 3H), 1.01 (t, 3H, J = 7.1 Hz) ppm. ^{13}C-NMR (C_6D_6, 125 MHz): δ = 166.50 (C_q), 159.33 (C_q), 132.14 (C_q), 123.64 (CH), 116.39 (CH), 59.39 (CH_2), 40.39 (CH_2), 26.30 (CH_2), 25.76 (CH_3), 17.65 (CH_3), 14.44 (CH_3) ppm.

(*Z*)-**S4**: TLC (petroleum ether: Et_2O = 7:1): R_f = 0.67. GC (HP-5): *I* = 1357. ^{1}H-NMR (C_6D_6, 500 MHz): δ = 5.75 (s, 1H), 5.28 (m, 1H), 4.03 (qd, 2H, *J* = 7.1, 1.0 Hz), 2.79 (t, 2H, *J* = 7.9 Hz), 2.22 (q, 2H, *J* = 7.2 Hz), 1.64 (s, 3H), 1.60 (s, 3H), 1.00 (t, 3H, *J* = 7.1 Hz) ppm. ^{13}C-NMR (C_6D_6, 125 MHz): δ = 166.05 (C_q), 159.85 (C_q), 132.04 (C_q), 124.04 (CH), 116.91 (CH), 59.37 (CH_2), 33.69 (CH_2), 27.32 (CH_2), 25.84 (CH_3), 17.70 (CH_3), 14.42 (CH_3) ppm.

Synthesis of (10,10,10-$^2H_3$)geraniol (S5)

To a cooled (0 °C) solution of the ester (*E*)-**S4** (160 mg, 0.8 mmol, 1.00 eq) in Et_2O (2 mL) was added DIBAl-H (1.1 mL, 1.93 mmol, 1.0 M in hexane, 2.40 eq) and the reaction mixture was stirred for 1 h at room temperature. The mixture was cooled to 0 °C and a saturated solution of Na-K-tartrate (10 mL) was added. The resulting slurry was stirred for 1 h to dissolve the precipitate and the aqueous phase was extracted with Et_2O (3x 20 mL). The organic layers were dried with $MgSO_4$ and concentrated under reduced pressure. The residue was purified by column chromatography (petroleum ether/Et_2O, 2:1) to yield the alcohol **S5** (111 mg, 0.71 mmol, 89%) as a colourless oil.

TLC (petroleum ether: Et_2O = 2:1): R_f = 0.50. GC (HP-5): *I* = 1256. ^{1}H-NMR (C_6D_6, 500 MHz): δ = 5.40 (tt, 1H, *J* = 6.8, 1.4 Hz), 5.17 (m, 1H), 3.97 (d, 2H, *J* = 6.7 Hz), 2.10 (m, 2H), 1.98 (m, 2H), 1.66 (s, 3H), 1.53 (s, 3H) ppm. ^{13}C-NMR (C_6D_6, 125 MHz): δ = 138.03 (C_q), 131.45 (C_q), 124.97 (CH), 124.60 (CH), 59.37 (CH_2), 39.84 (CH_2), 26.84 (CH_2), 25.84 (CH_3), 17.73 (CH_3) ppm.

Synthesis of (10,10,10-$^2H_3$)GPP (S7)

To a cooled (0 °C) solution of **S5** (111 mg, 0.71 mmol, 1.00 eq) in Et_2O (2 mL) PBr_3 (24 μL, 0.25 mmol, 0.40 eq) was added. The mixture was stirred for 1 h at 0 °C and then poured onto an ice/water mixture. The aqueous layer was extracted with Et_2O (3x 20 mL), the organic layers were dried with $MgSO_4$ and concentrated under reduced pressure. The bromide **S6** (ca. 400 mg) was directly used for the next reaction without purification.

To a solution of tris(tetra-n-butylammonium)hydrogen diphosphate (693 g, 0.77 mmol, 1.20 eq) in acetonitrile (1 mL) a solution of the allyl bromide **S3** was added and the mixture was stirred at room temperature overnight. Acetonitrile was removed under reduced pressure. The residue was dissolved in an aqueous NH_4HCO_3 solution (1 mL, 0.25 M) and loaded onto a DOWEX 50WX8 ion-exchange column (NH_4^+ form, pH 7.0). The column was flushed slowly with 1.5 column volumes of NH_4HCO_3 buffer (25 mM, 5% iPrOH) and the eluate was lyophilised to yield the diphosphate **S7** as a colourless hygroscopic powder (160 mg, 0.44 mmol, 61%).

^{1}H-NMR (D_2O, 500 MHz): δ = 5.47 (t, 1H, *J* = 7.2 Hz), 5.22 (m, 1H), 4.49 (t, 2H, *J* = 6.7 Hz), 2.17 (m, 2H), 2.12 (m, 2H), 1.71 (s, 3H), 1.65 (s, 3H) ppm. ^{13}C-NMR (D_2O, 125 MHz): δ = 142.84 (C_q), 133.79 (C_q), 124.21 (CH), 119.90 (d, 1H, $^3J_{C,P}$ = 8.2 Hz), 62.79 (d, CH_2, $^2J_{C,P}$ = 5.4 Hz), 38.80 (CH_2), 25.69 (CH_2), 24.91 (CH_3), 17.06 (CH_3) ppm. ^{31}P-NMR (D_2O, 203 MHz): δ = –7.99 (d, $^2J_{P,P}$ = 20.9 Hz), –10.29 (d, $^2J_{P,P}$ = 21.5 Hz) ppm.

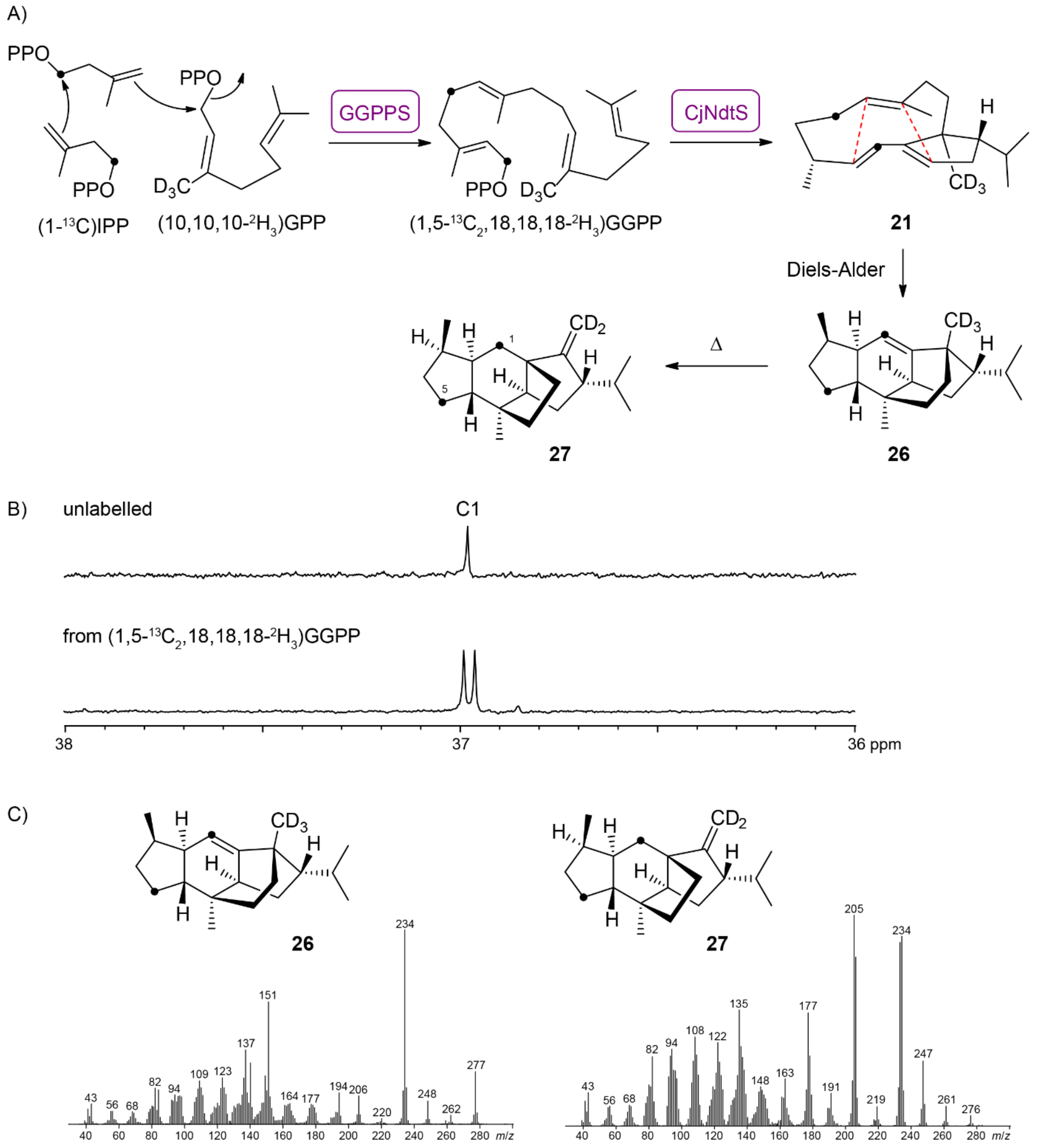

Figure S111. The mechanism of the rearrangement from **26** to **27**. A) Enzymatic conversion of (10,10,10-$^2H_3$)GPP and (1-^{13}C)IPP with GGPPS and CjNdtS to **21**, followed by Diels-Alder reaction to **26** and thermal rearrangement to **27**. B) ^{13}C-NMR spectra showing the region for C1 of unlabelled **27** (top) and labelled **27** obtained from (10,10,10-$^2H_3$)GPP and (1-^{13}C)IPP through (1,5-$^{13}C_2$,18,18,18-$^2H_3$)GGPP (bottom). The doublet as a result of a $^3J_{C,C}$ coupling (no triplet $^1J_{C,D}$ coupling) excludes the mechanistic hypothesis of a concerted reaction as shown in the Box 1 in Scheme 3 of main text. C) EI mass spectra of labelled **26** and **27** obtained from (10,10,10-$^2H_3$)GPP and (1-^{13}C)IPP. The molecular ion of **26** at *m/z* 277 shows incorporation of three deuterium atoms, while the molecular ion of **27** at *m/z* 276 reveals the loss of one deuterium in the step from **26** to **27**, further excluding an intramolecular hydrogen shift. Black dots represent ^{13}C-labelled carbons.

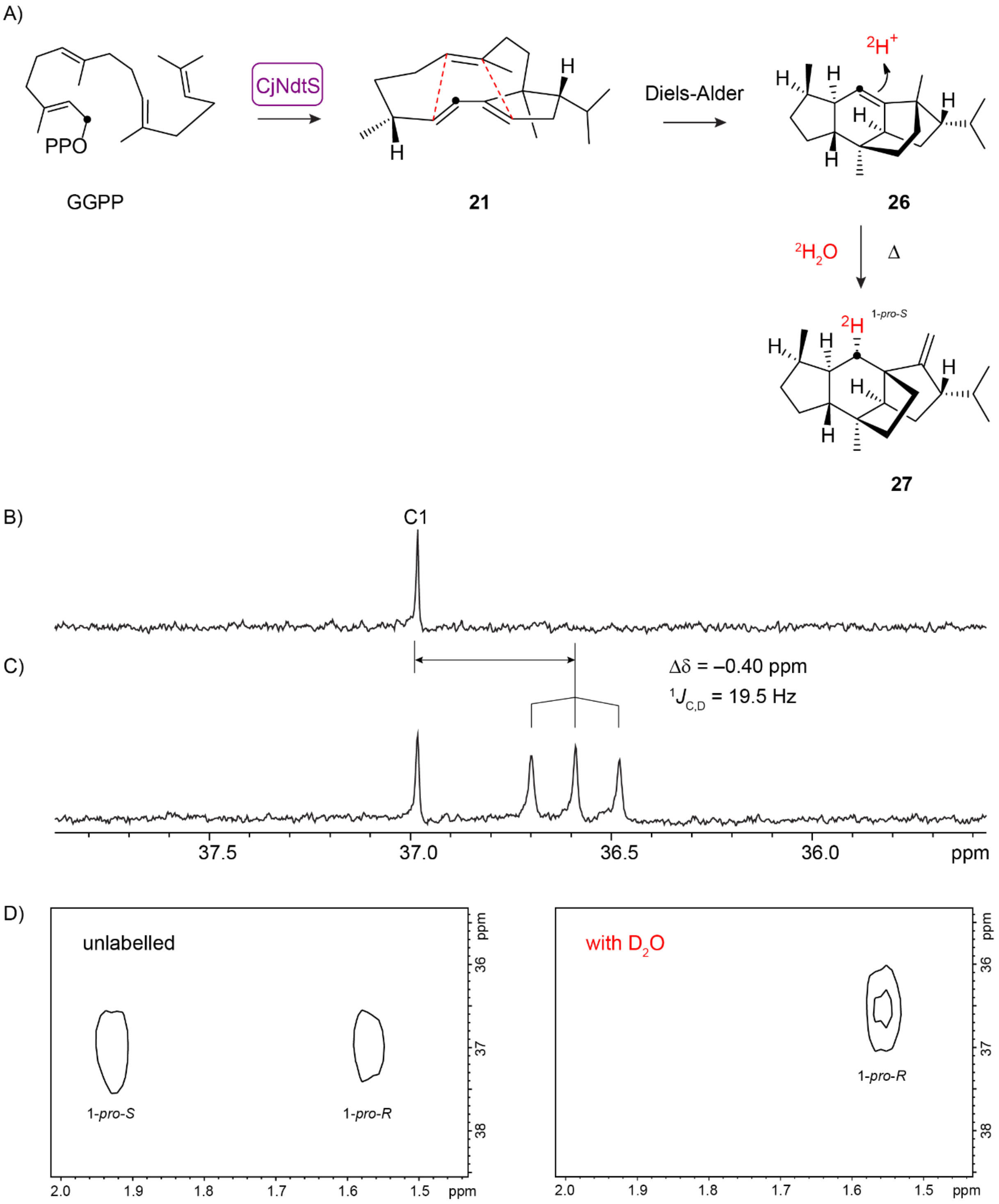

Figure S112. The mechanism of the rearrangement of **26** to **27** (Scheme 3 of main text). A) Enzymatic conversion of (1-^{13}C)GGPP into labelled **21** with CjNdtS, followed by thermal Diels-Alder reaction and skeletal rearrangement at 230 °C in Ph_2O saturated with D_2O. Partial ^{13}C spectra showing the region for C1 of B) unlabelled **27** and C) labelled **27**. The upfield shifted triplet for C1 ($\Delta\delta$ = –0.40 ppm, $^{1}J_{C,D}$ = 19.5 Hz) indicates a deuterium incorporation at C1, supporting the mechanistic proposal involving an external proton source. D) HSQC spectra showing the region of C1 for unlabelled **27** (left) and labelled **27** (right), revealing selective deuterium incorporation into the 1-*pro-S* position of **27** and thus protonation of **26** from the *Si* face (i. e. the sterically less hindered convex side). Black dots represent ^{13}C-labelled carbons.

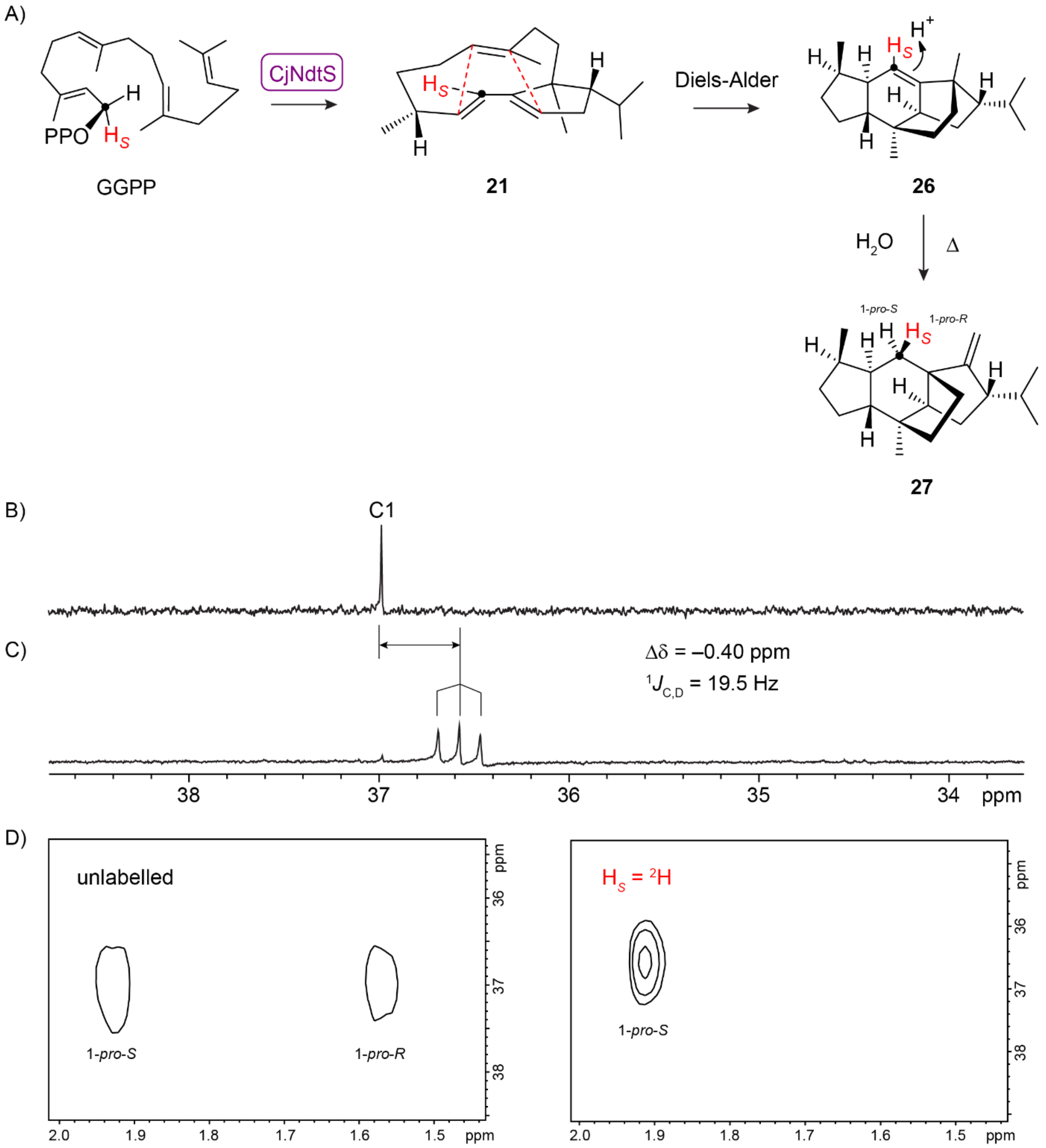

Figure S113. The mechanism of the rearrangement of **26** to **27** (Scheme 3 of main text). A) Enzymatic conversion of (*S*)-(1-^{13}C,1-^{2}H)GGPP (H_S = ^{2}H) into labelled **21** with CjNdtS, followed by thermal Diels-Alder reaction and skeletal rearrangement at 230 °C in Ph_2O saturated with H_2O. Partial ^{13}C spectra showing the region for C1 of B) unlabelled **27** and C) labelled **27**. The upfield shifted triplet for C1 ($\Delta\delta$ = –0.40 ppm, $^1J_{C,D}$ = 19.5 Hz) indicates a deuterium incorporation at C1. D) HSQC spectra showing the region of C1 for unlabelled **27** (left) and labelled **27** (right), revealing selective deuterium incorporation into the 1-*pro-R* position of **27** and thus protonation of **26** from the *Si* face (i. e. the sterically less hindered convex side). Black dots represent ^{13}C-labelled carbons.

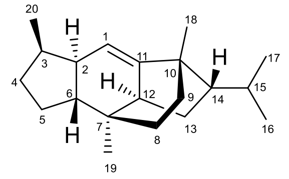

26

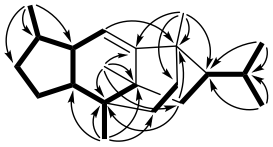

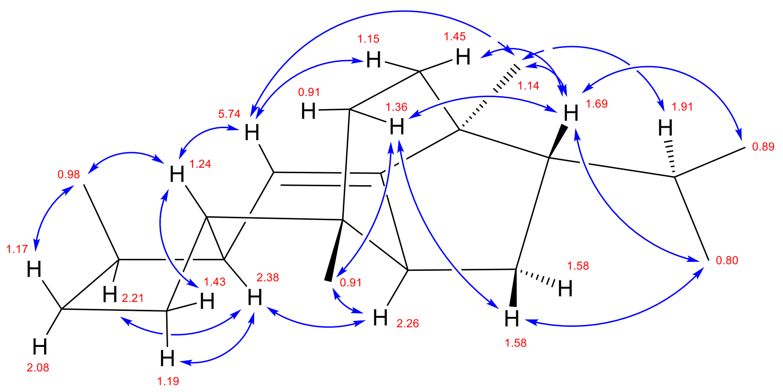

Figure S114. Structure elucidation of thermocyclene A (**26**). Bold: $^{1}H,^{1}H$-COSY, single headed arrows: key HMBC, and double headed arrows: NOESY correlations. Carbon numbering follows GGPP numbering to indicate the origin of each carbon.

Table S17. NMR data of thermocyclene A (**26**) in C_6D_6 recorded at 298 K.

C[a]	type	^{13}C[b]	^{1}H[b]
1	CH	115.90	5.75 (br dd, *J* = 6.0, 1.5)
2	CH	50.92	2.38 (ddd, *J* = 10.9, 8.4, 6.2)
3	CH	29.62	2.21 (m)
4	CH_2	34.86	2.08 (m)
			1.17 (m)
5	CH_2	26.55	1.43 (m)
			1.19 (m)
6	CH	55.96	1.24 (m)
7	C_q	42.32	–
8	CH_2	36.86	1.36 (ddd, *J* = 13.9, 12.3, 6.8)
			0.91 (m)
9	CH_2	52.51	1.45 (m)
			1.15 (m)
10	C_q	50.53	–
11	C_q	159.74	–
12	CH	51.01	2.26 (br d, *J* = 7.9)
13	CH_2	23.28	1.58 (m, 2H)
14	CH	51.36	1.69 (ddd, *J* = 8.5, 4.2, 3.0)
15	CH	29.07	1.91 (dhept, *J* = 2.9, 6.8)
16	CH_3	15.65	0.80 (d, *J* = 6.7)
17	CH_3	22.25	0.89 (d, *J* = 6.9)
18	CH_3	17.34	1.14 (s)
19	CH_3	23.14	0.90 (s)
20	CH_3	19.60	0.98 (d, *J* = 7.2)

[a] Carbon numbering as shown in Figure S114 indicates the origin of each carbon from GGPP by same number. [b] Chemical shifts δ in ppm, multiplicity: s = singlet, d = doublet, hept = heptet, m = multiplet, br = broad, coupling constants *J* are given in Hertz.

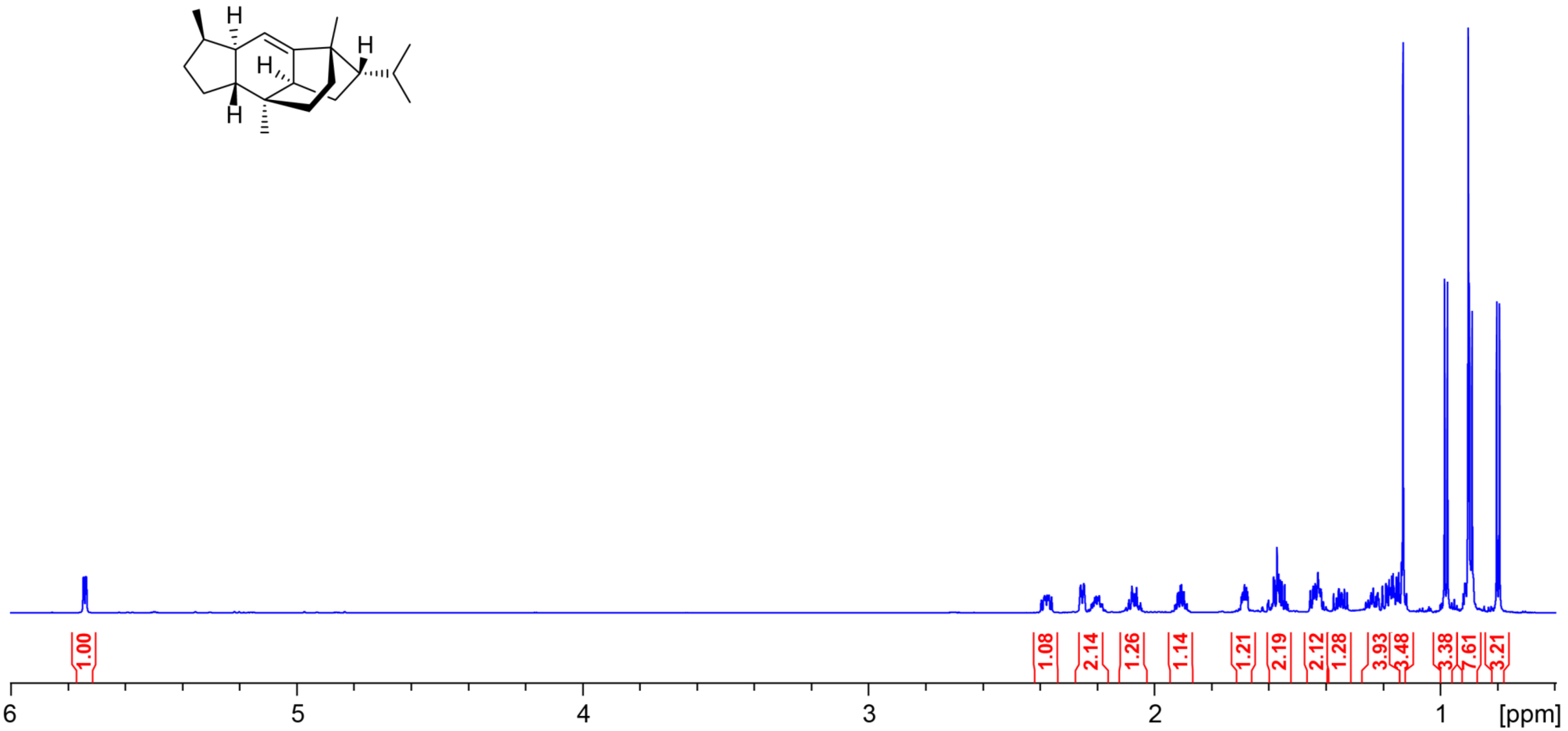

Figure S115. ^{1}H-NMR spectrum of **26** (700 MHz, C_6D_6).

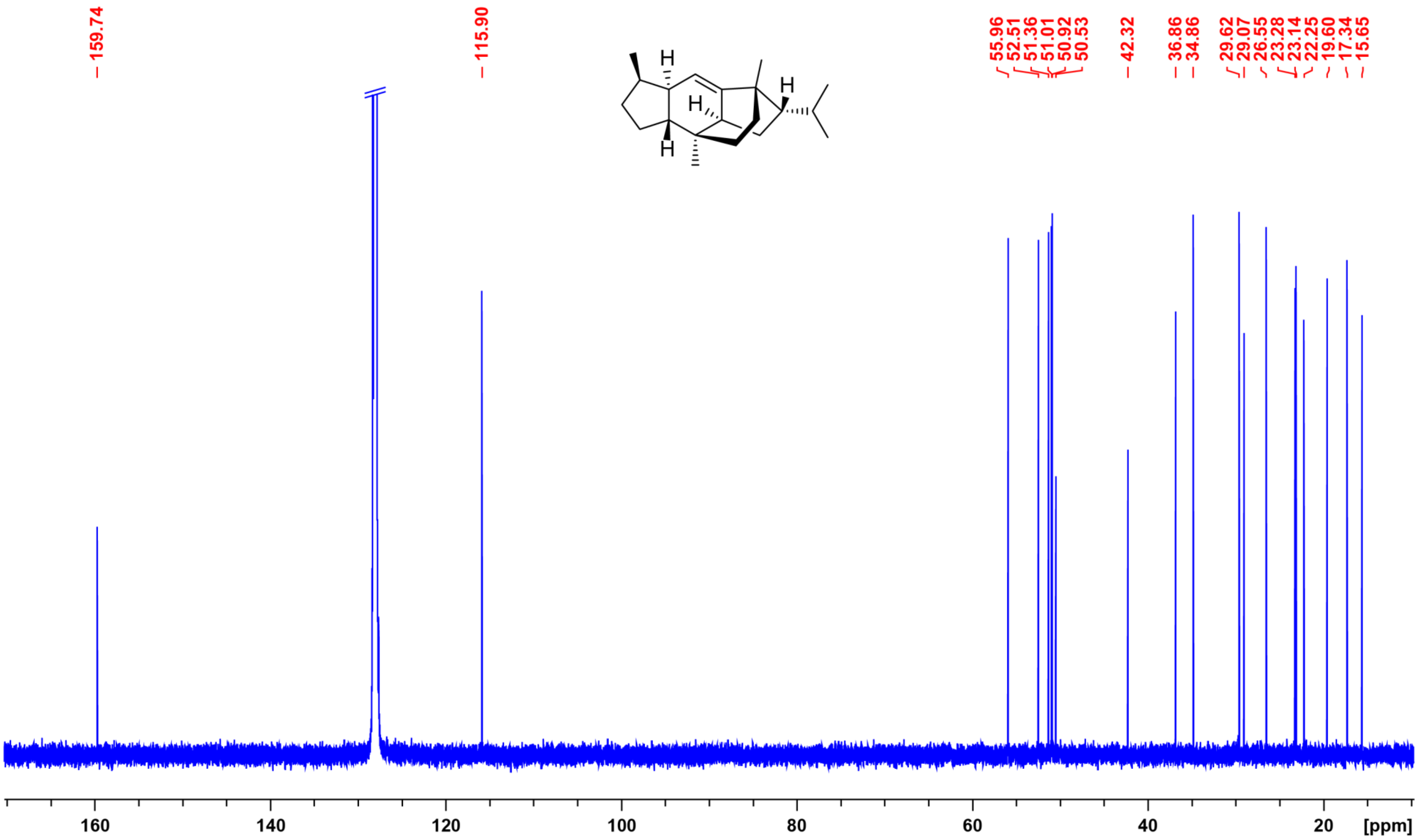

Figure S116. ^{13}C-NMR spectrum of **26** (176 MHz, C_6D_6).

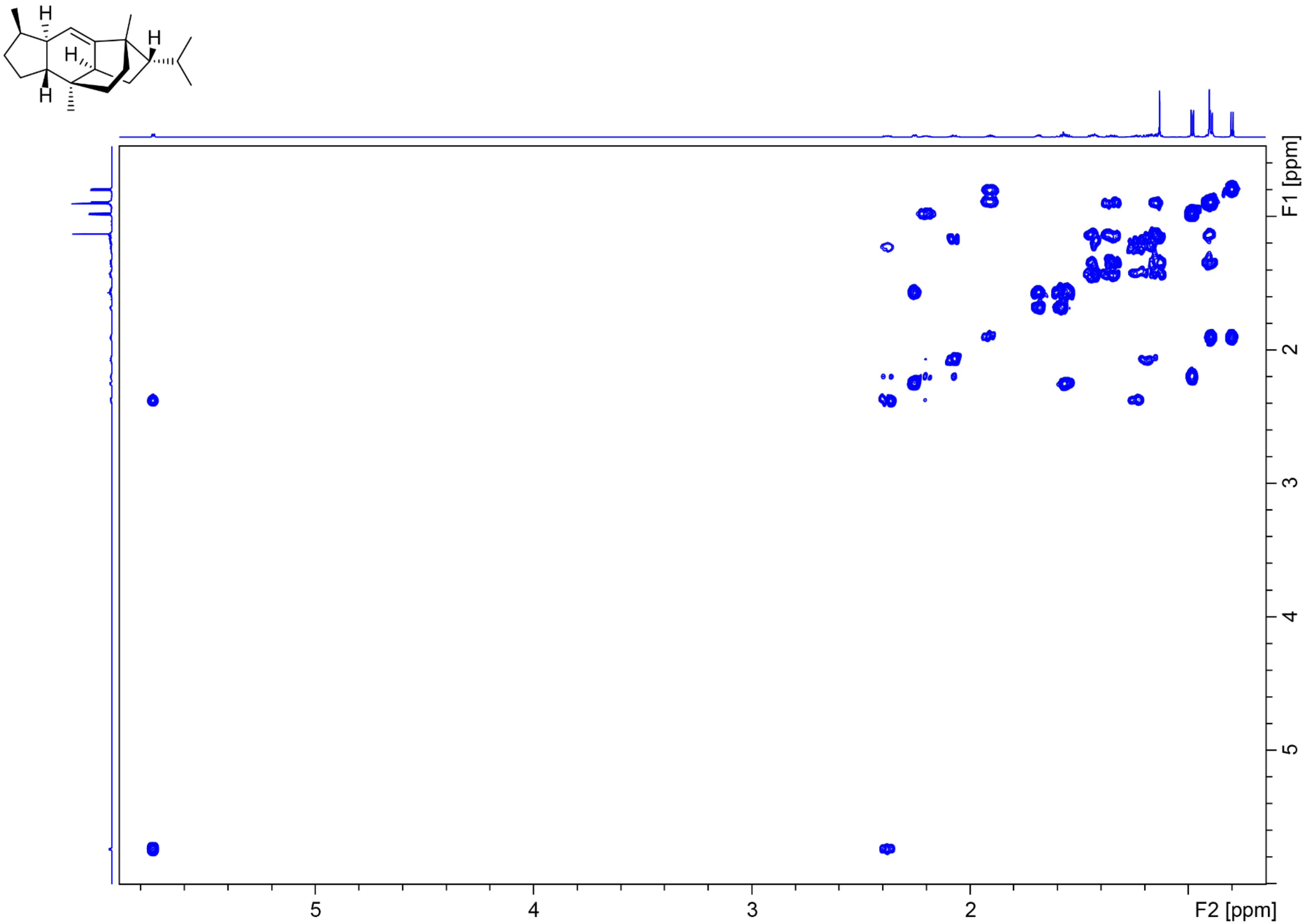

Figure S117. ^{1}H-^{1}H-COSY spectrum (C_6D_6) of **26**.

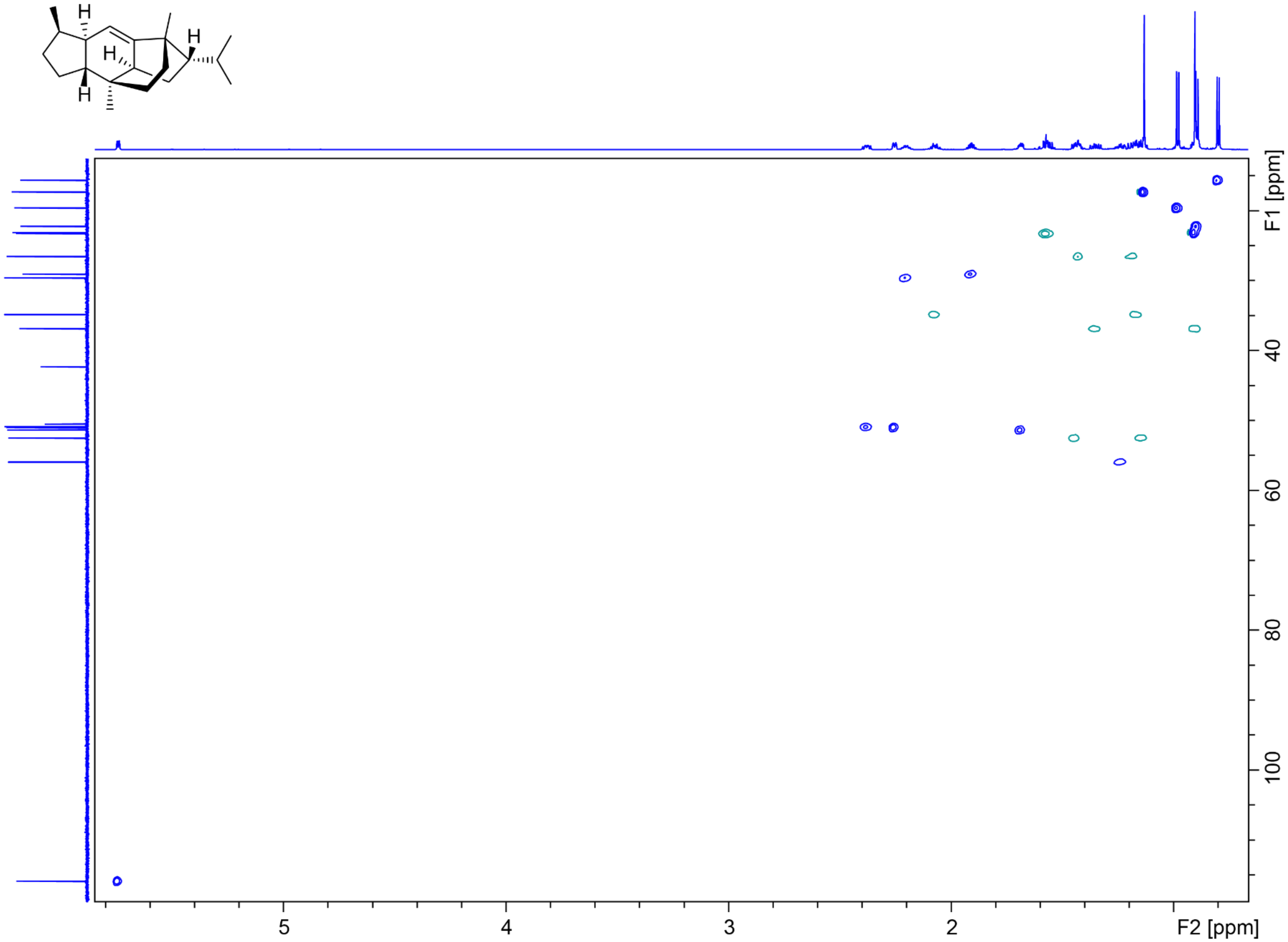

Figure S118. HSQC spectrum (C_6D_6) of **26**.

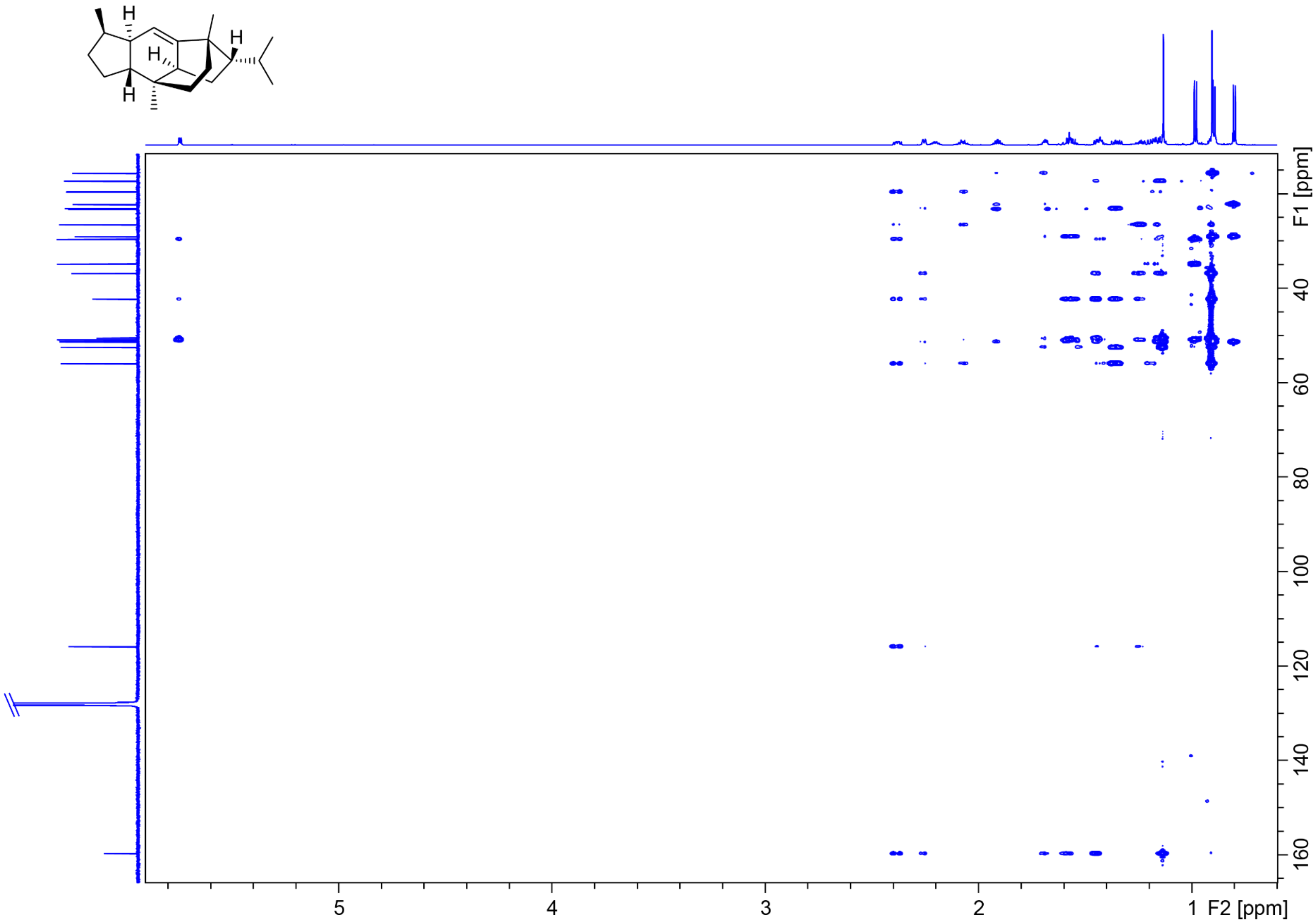

Figure S119. HMBC spectrum (C_6D_6) of **26**.

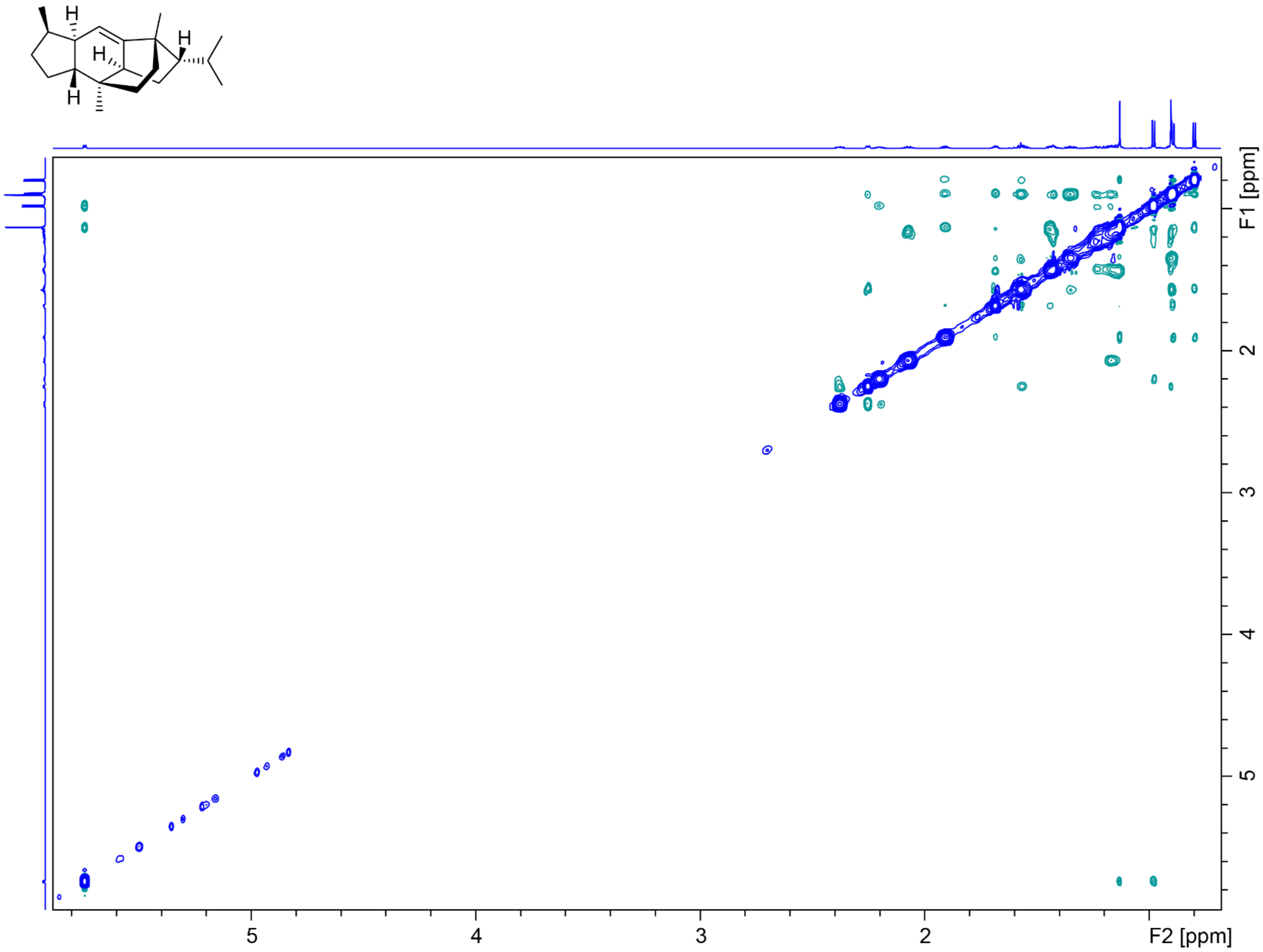

Figure S120. NOESY spectrum (C_6D_6) of **26**.

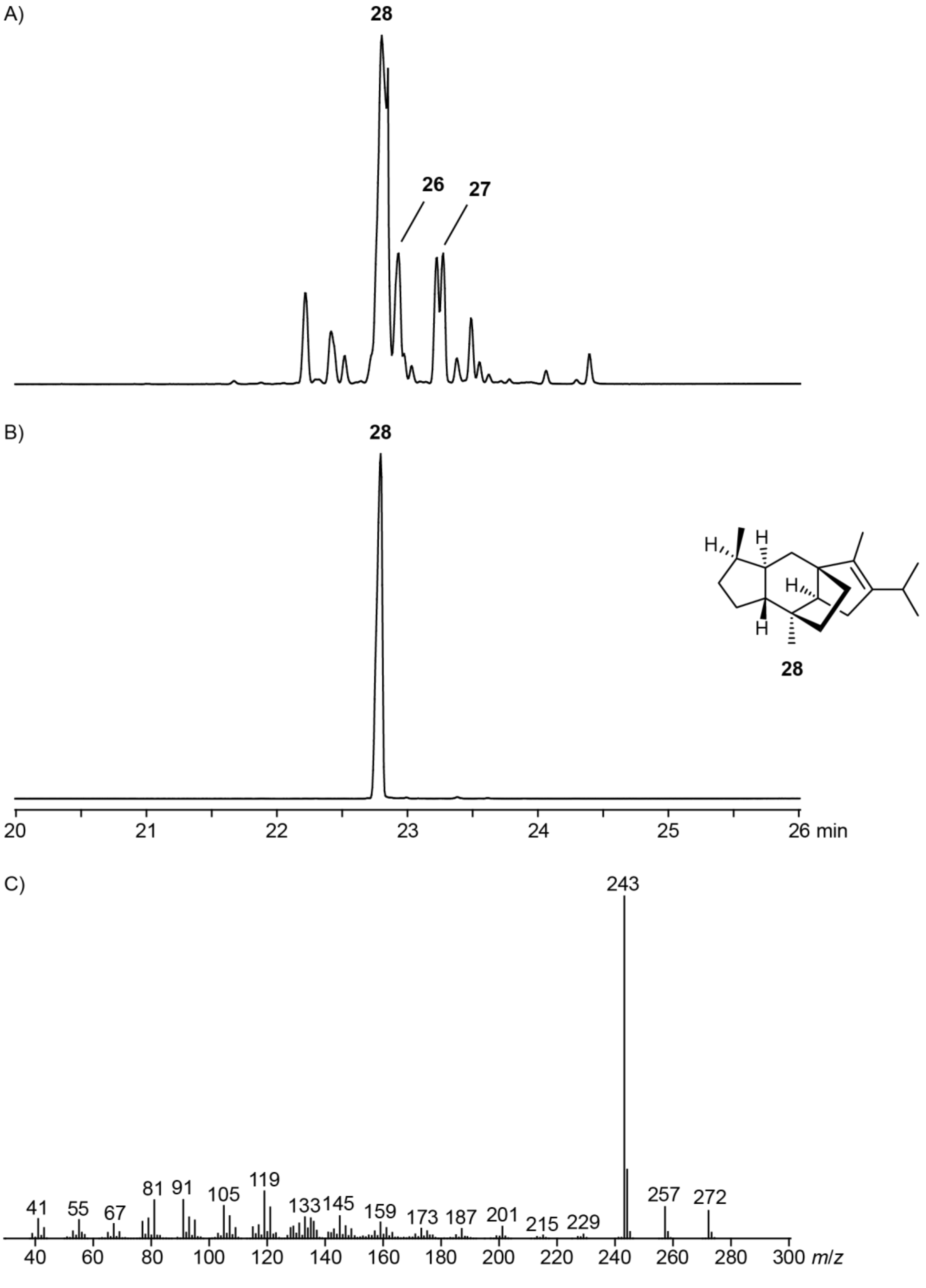

Figure S121. Thermal reaction products of **21** obtained after prolonged heating. Total ion chromatogrammes of A) the crude thermal reaction products, and B) purified **28**. The sharp peak in B) shows no further conversion by the thermal impact during GC/MS analysis. C) EI mass spectrum of **28**.

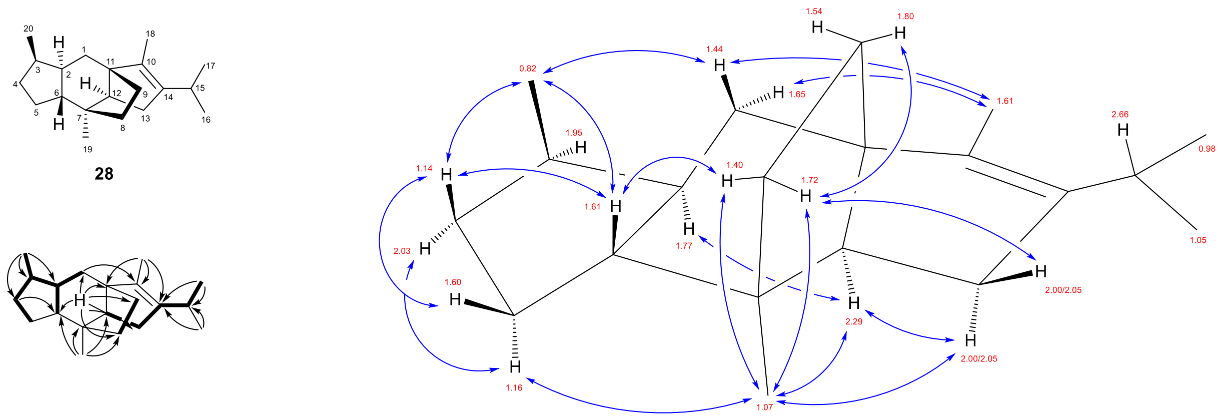

Figure S122. Structure elucidation of thermocyclene C (**28**). Bold: ^{1}H,^{1}H-COSY, single headed arrows: key HMBC, and double headed arrows: NOESY correlations. Carbon numbering follows GGPP numbering to indicate the origin of each carbon.

Table S18. NMR data of thermocyclene C (**28**) in C_6D_6 recorded at 298 K.

C[a]	type	^{13}C[b]	^{1}H[b]
1	CH_2	34.95	1.65 (m)
			1.44 (dd, *J* = 11.8, 11.0)
2	CH	43.28	1.77 (m)
3	CH	30.94	1.95 (m)
4	CH_2	33.52	2.03 (m)
			1.14 (m)
5	CH_2	27.68	1.60 (m)
			1.16 (m)
6	CH	50.73	1.61 (m)
7	C_q	41.89	–
8	CH_2	43.17	1.72 (m)
			1.40 (br dd, *J* = 9.8, 9.8)
9	CH_2	41.38	1.80 (m)
			1.54 (m)
10	C_q	137.70	–
11	C_q	59.45	–
12	CH	53.65	2.29 (t, *J* = 8.6)
13	CH_2	28.88	2.05 (m)
			2.00 (m)
14	C_q	138.63	–
15	CH	27.21	2.66 (hept, *J* = 6.9)
16	CH_3	21.35	0.98 (d, *J* = 6.8)
17	CH_3	21.66	1.05 (d, *J* = 6.9)
18	CH_3	10.50	1.61 (m)
19	CH_3	19.12	1.07 (s)
20	CH_3	18.76	0.82 (d, *J* = 7.1)

[a] Carbon numbering as shown in Figure S122 indicates the origin of each carbon from GGPP by same number. [b] Chemical shifts δ in ppm, multiplicity: s = singlet, d = doublet, t = triplet, hept = heptet, m = multiplet, br = broad, coupling constants *J* are given in Hertz.

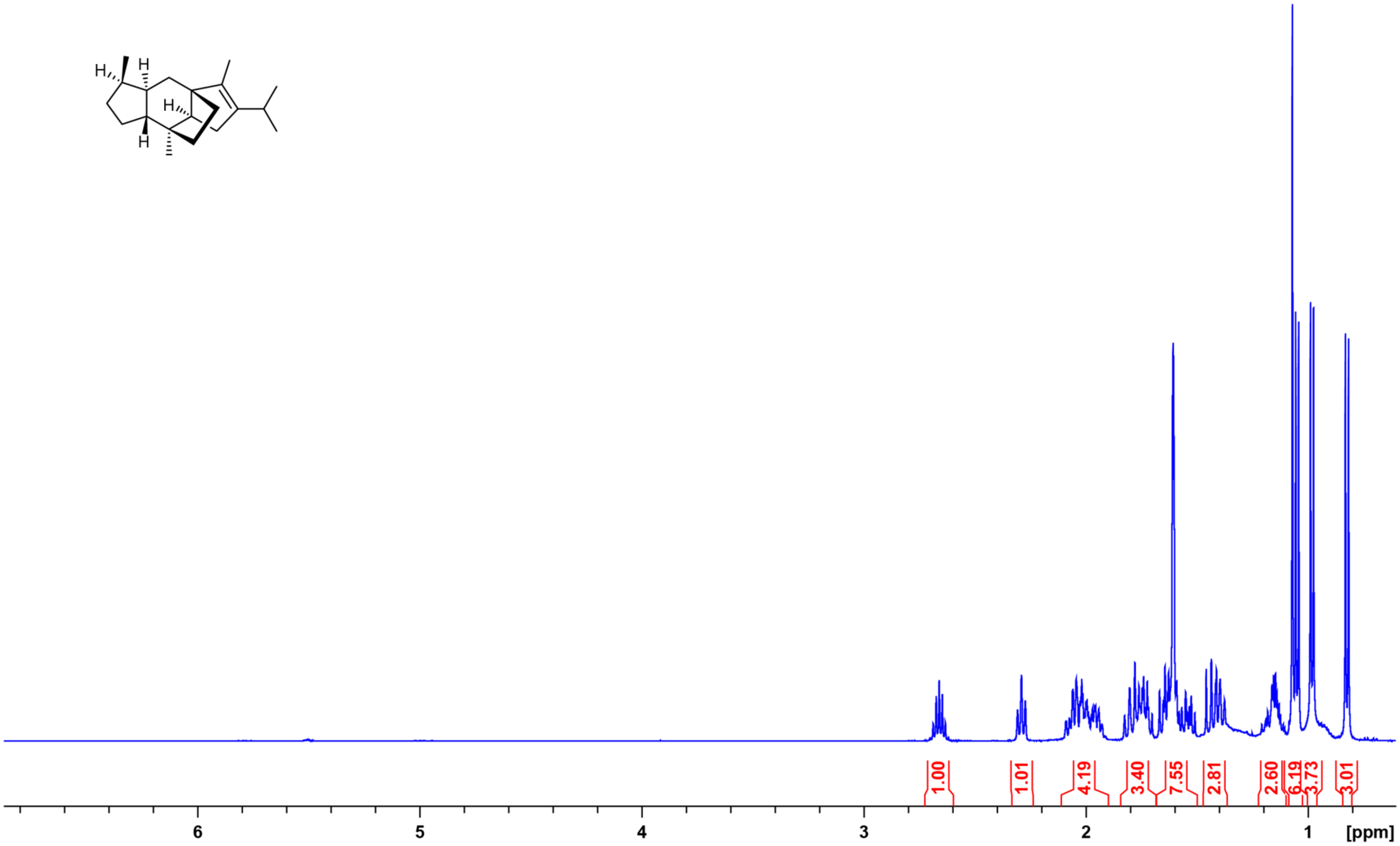

Figure S123. ^{1}H-NMR spectrum of **28** (700 MHz, C_6D_6).

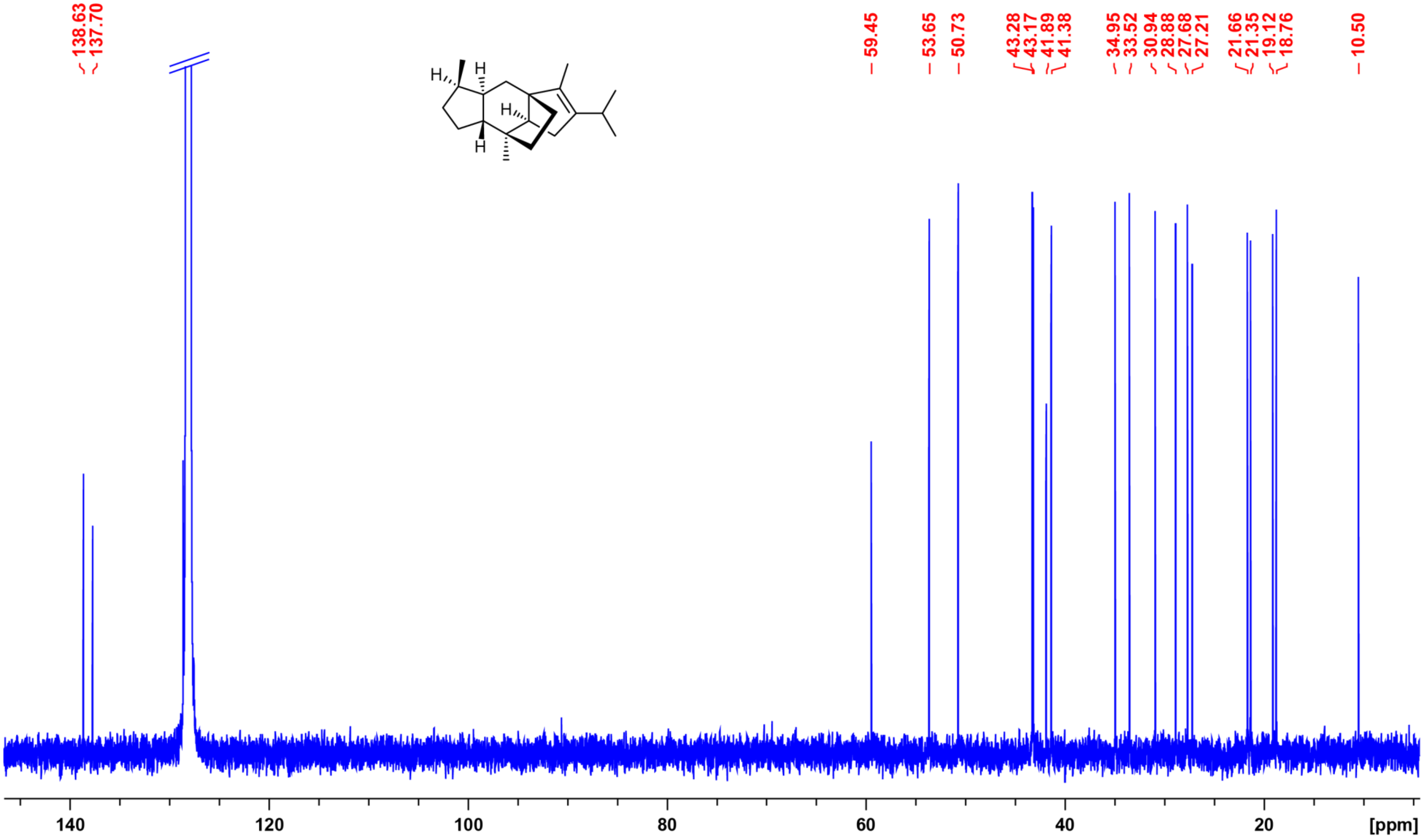

Figure S124. ^{13}C-NMR spectrum of **28** (176 MHz, C_6D_6).

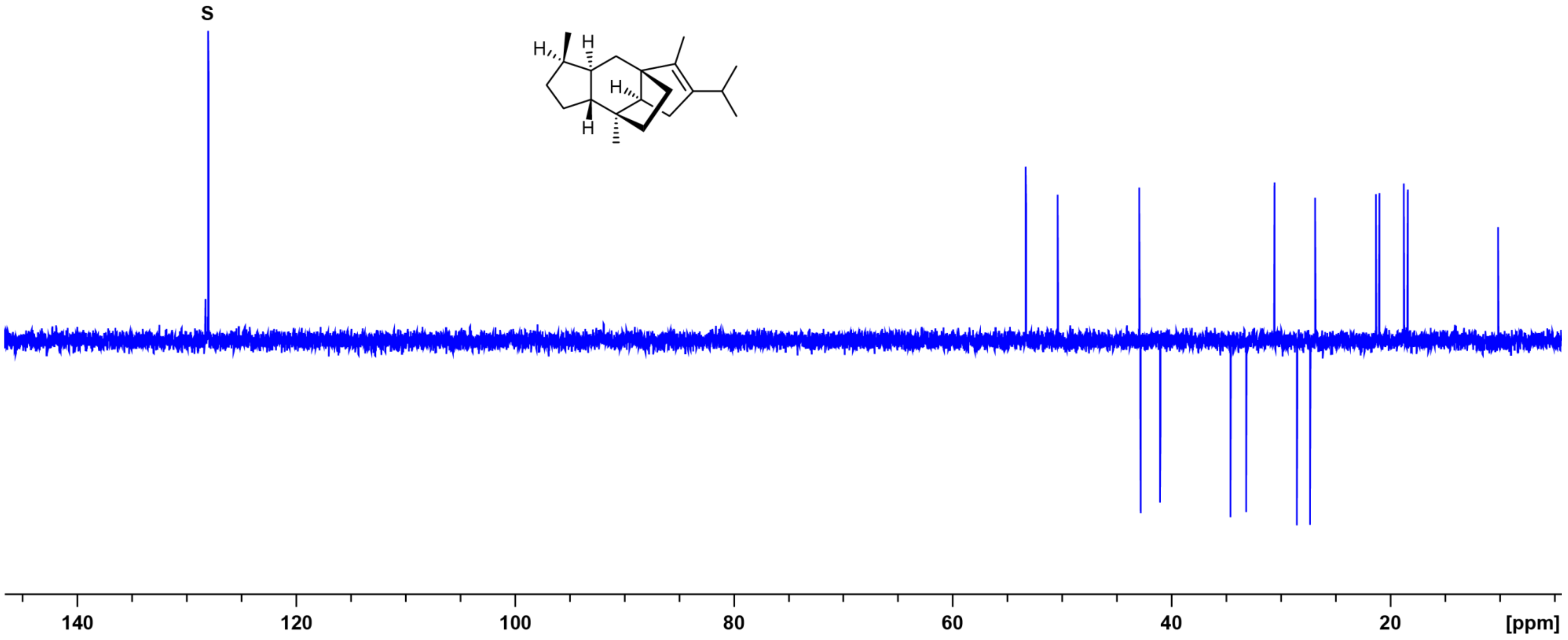

Figure S125. ^{13}C-DEPT135 spectrum of **28** (176 MHz, C_6D_6). The peak labelled "S" is the residual solvent signal (from C_6D_5H).

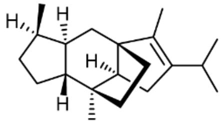

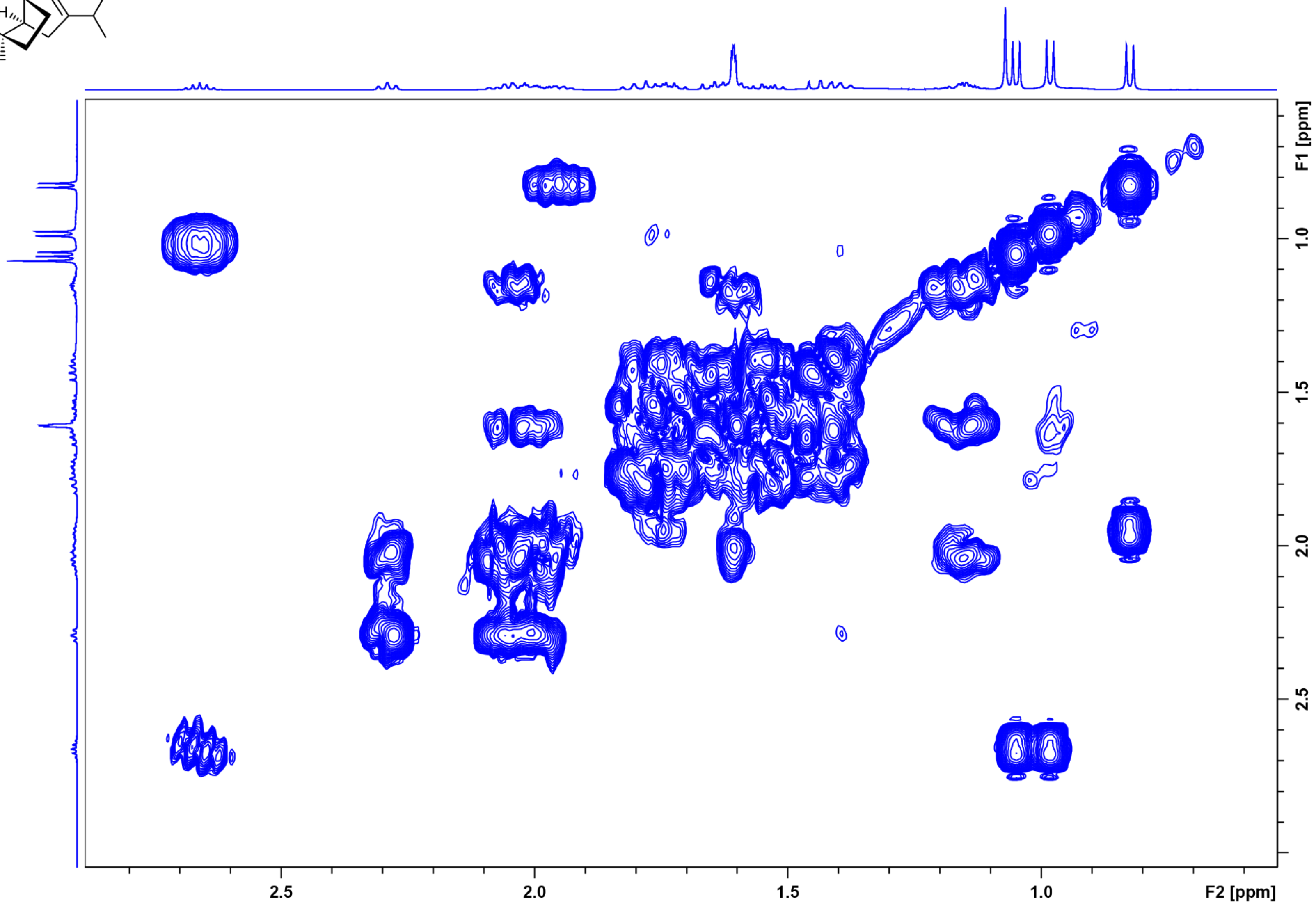

Figure S126. ^{1}H-^{1}H-COSY spectrum (C_6D_6) of **28**.

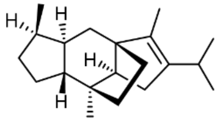

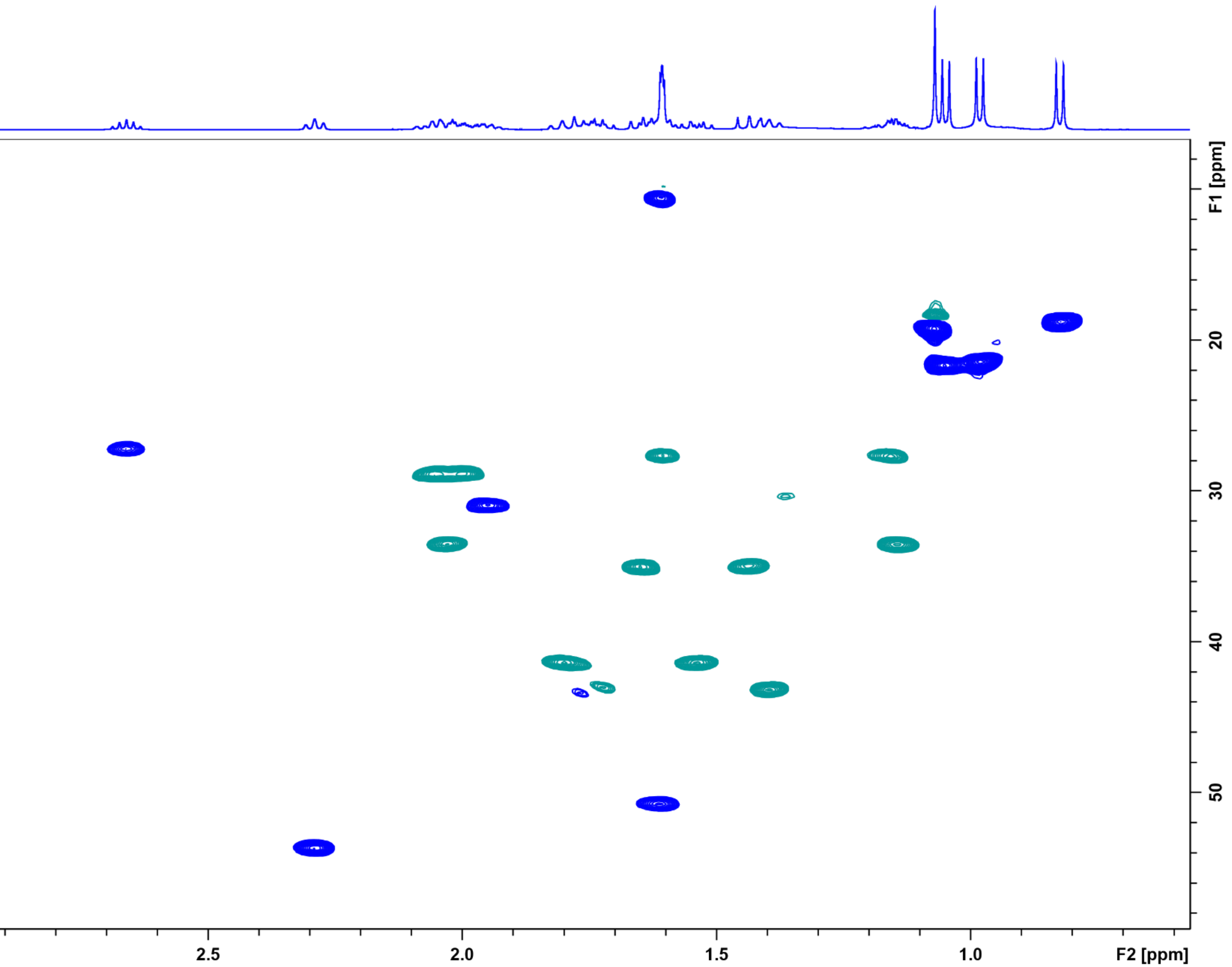

Figure S127. HSQC spectrum (C_6D_6) of **28**.

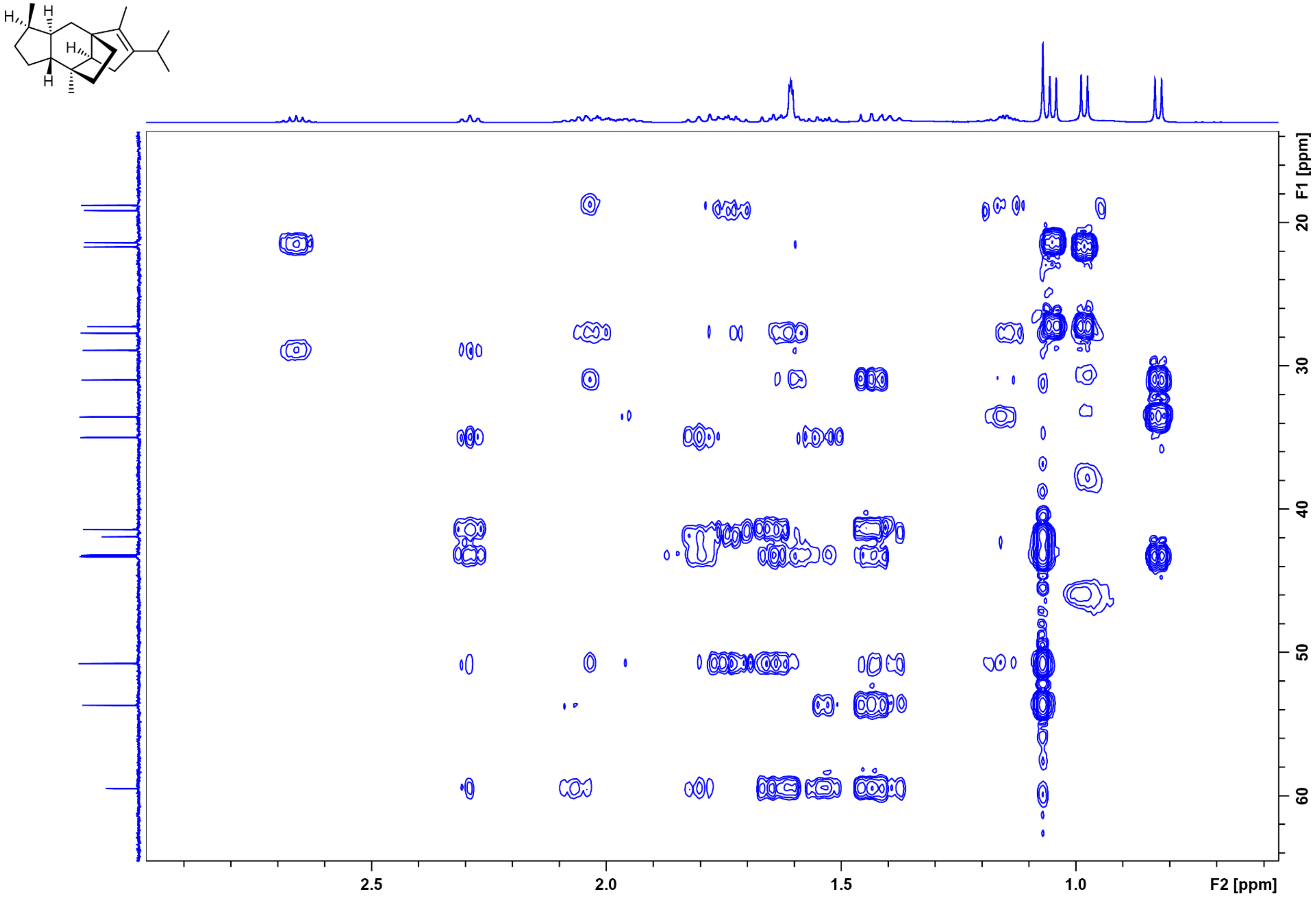

Figure S128. HMBC spectrum (C_6D_6) of **28**.

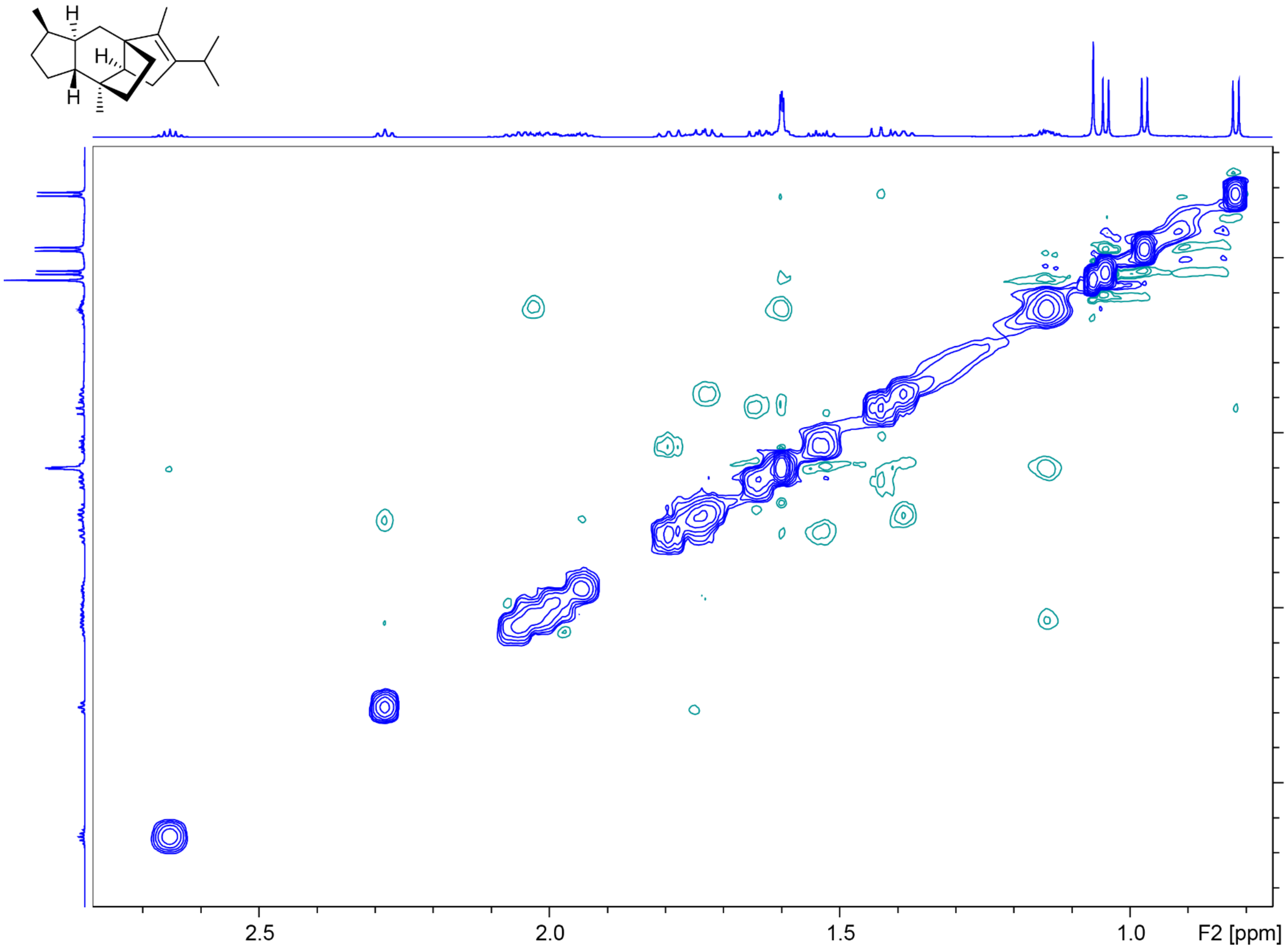

Figure S129. NOESY spectrum (C_6D_6) of **28**.

A)

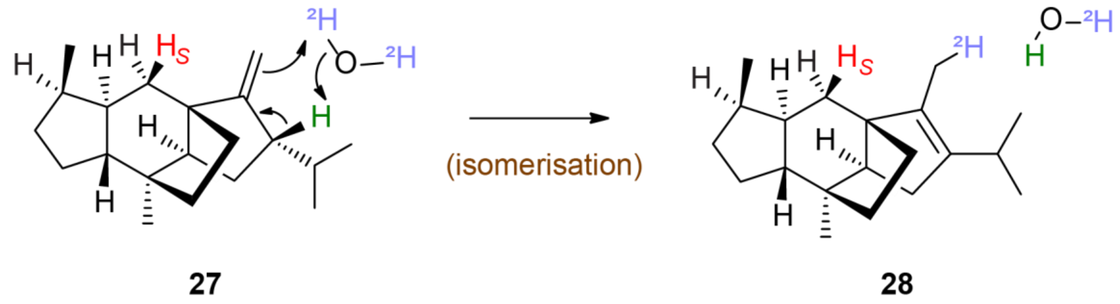

B)

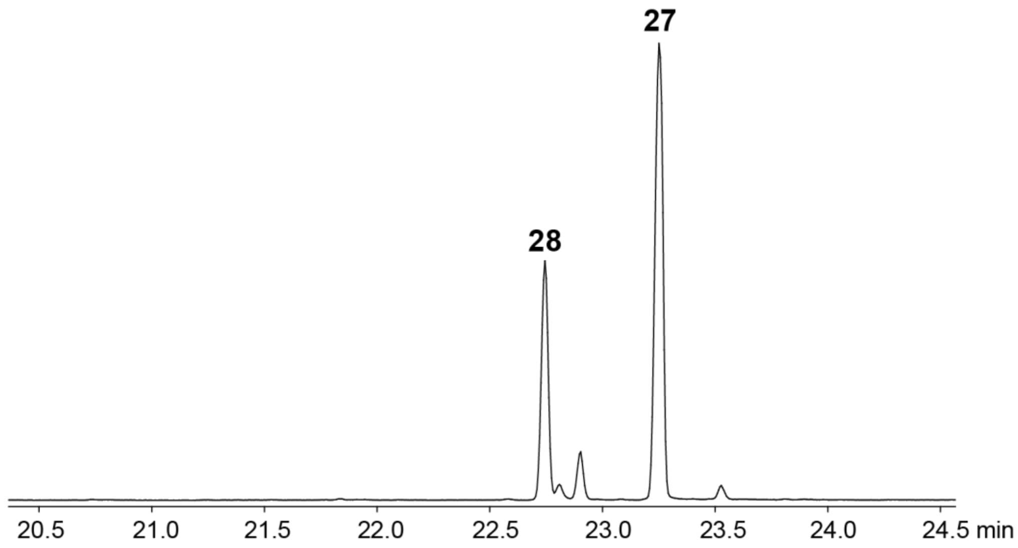

C)

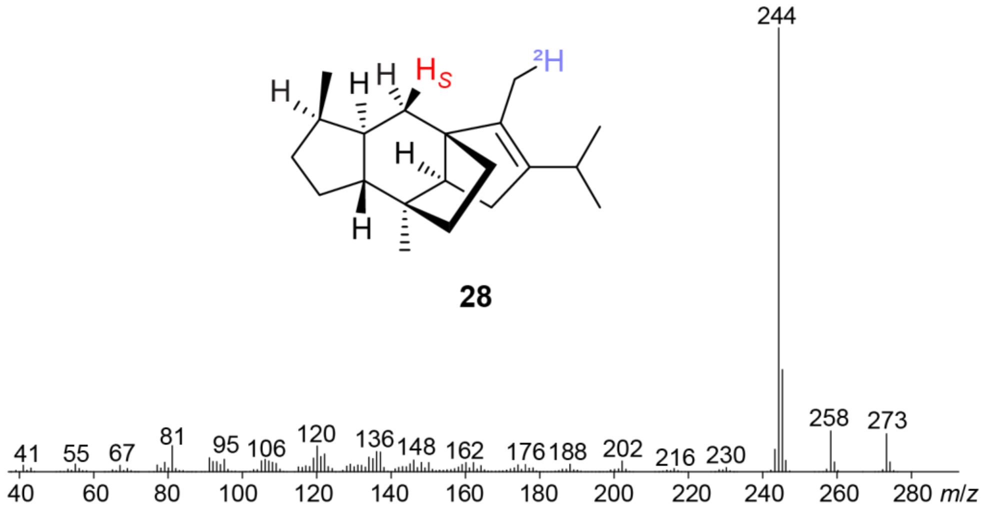

Figure S130. Isomerisation of **27** to **28**. A) The isomerisation of **27** to **28** proceeds with incorporation of a proton from D_2O. B) Total ion chromatogram of the crude thermal reaction products. C) EI mass spectrum of (^{2}H)-**28**.

Table S19. Results of DFT calculations (wB97M-V/Def2-TZVPPD//B97D3/6-31G(d,p)) for the Diels-Alder reaction from **21** to **26**, and the thermal rearrangement from **26** to **27** and **28** (Scheme 3 of main text).

Structure	Gibbs energy (298.15K) / Hartree	free energy relative to reactant / kcal/mol	free energy of activation / kcal/mol	free reaction energy / kcal/mol
**Diels-Alder reaction from 21 to 26[a]**				
**21**	–780.827844	0.0		
**DA-TS**	–780.783105	28.1	28.1	
**26**	–780.857560	–18.7		–18.7

Structure	Gibbs energy (298.15K) / Hartree	free energy relative to reactant / kcal/mol	free energy of activation / kcal/mol	free reaction energy / kcal/mol
**Thermal rearrangement in water from 26 to 27[b]**				
**26**	–857.291200	0.0		
**26-X-TS**	–857.187655	65.0	65.0	
**X**	–857.184350	67.1		67.1
**X**	–857.184328	67.1		
**X-27-TS**	–857.188489	64.5	–2.6	
**27**	–857.301492	–6.5		–73.5

Structure	Gibbs energy (298.15K) / Hartree	free energy relative to reactant / kcal/mol	free energy of activation / kcal/mol	free reaction energy / kcal/mol
**Thermal isomerisation in water from 27 to 28[b]**				
**27**	–867.301951	0.0		
**Y-TS**	–857.217227	53.2	53.2	
**28**	–857.309076	–4.5		–4.5

[a] Calculated for a reaction in the gas phase (wB97M-V/Def2-TZVPPD//B97D3/6-31G(d,p)) .
[b] Calculated for a polar medium (H_2O, wB97M-V/Def2-TZVPPD-CPCM(water)//B97D3/6-31G(d,p)).

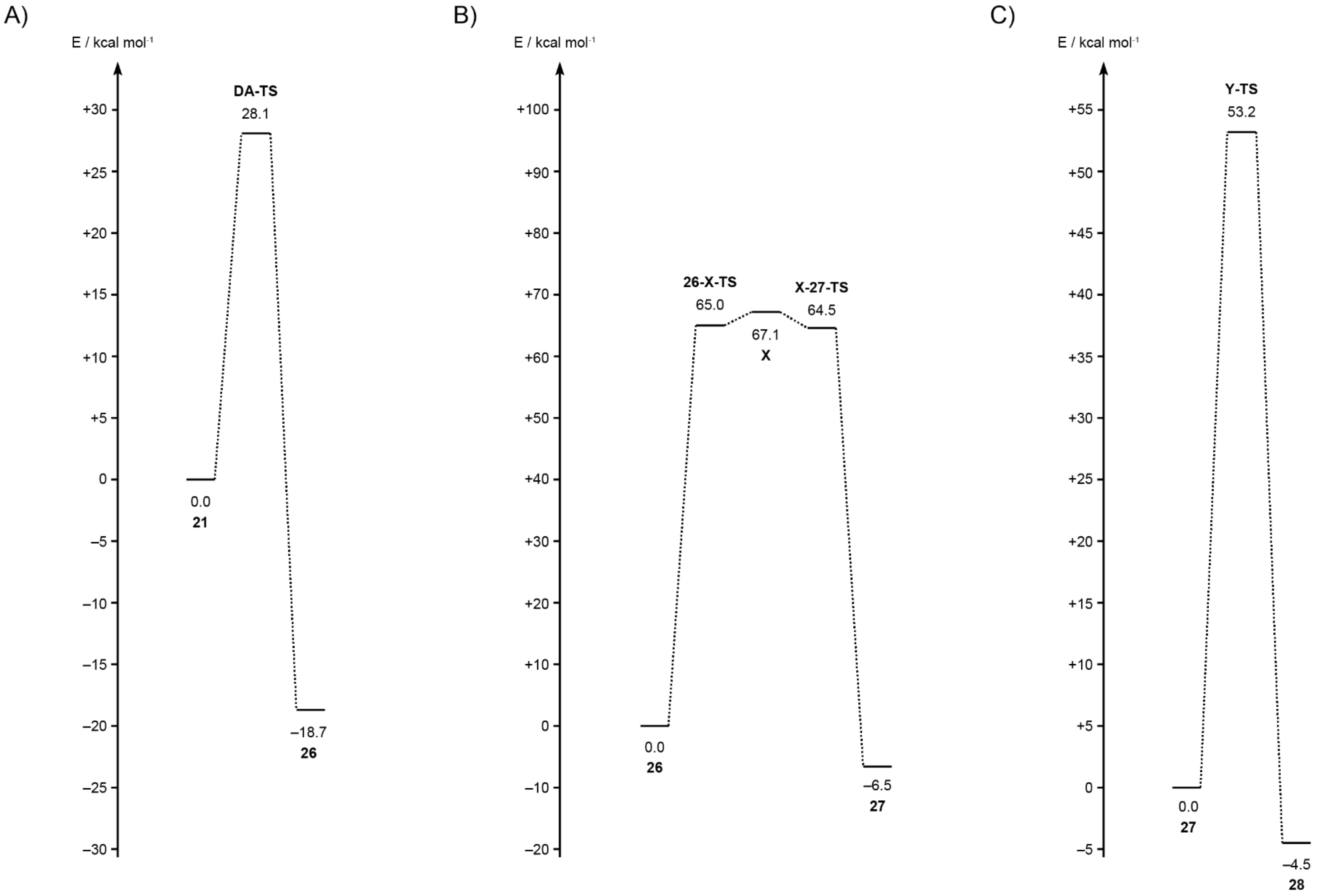

Figure S131. Computed energy profiles for A) the Diels-Alder reaction from **21** to **26** (gas phase reaction), and B) the thermal rearrangement from **26** to **27** (in H_2O), and C) the thermal isomerisation of **27** to **28** (in H_2O) (Scheme 3 of main text, wB97M-V/Def2-TZVPPD//B97D3/6-31G(d,p), 298 K).

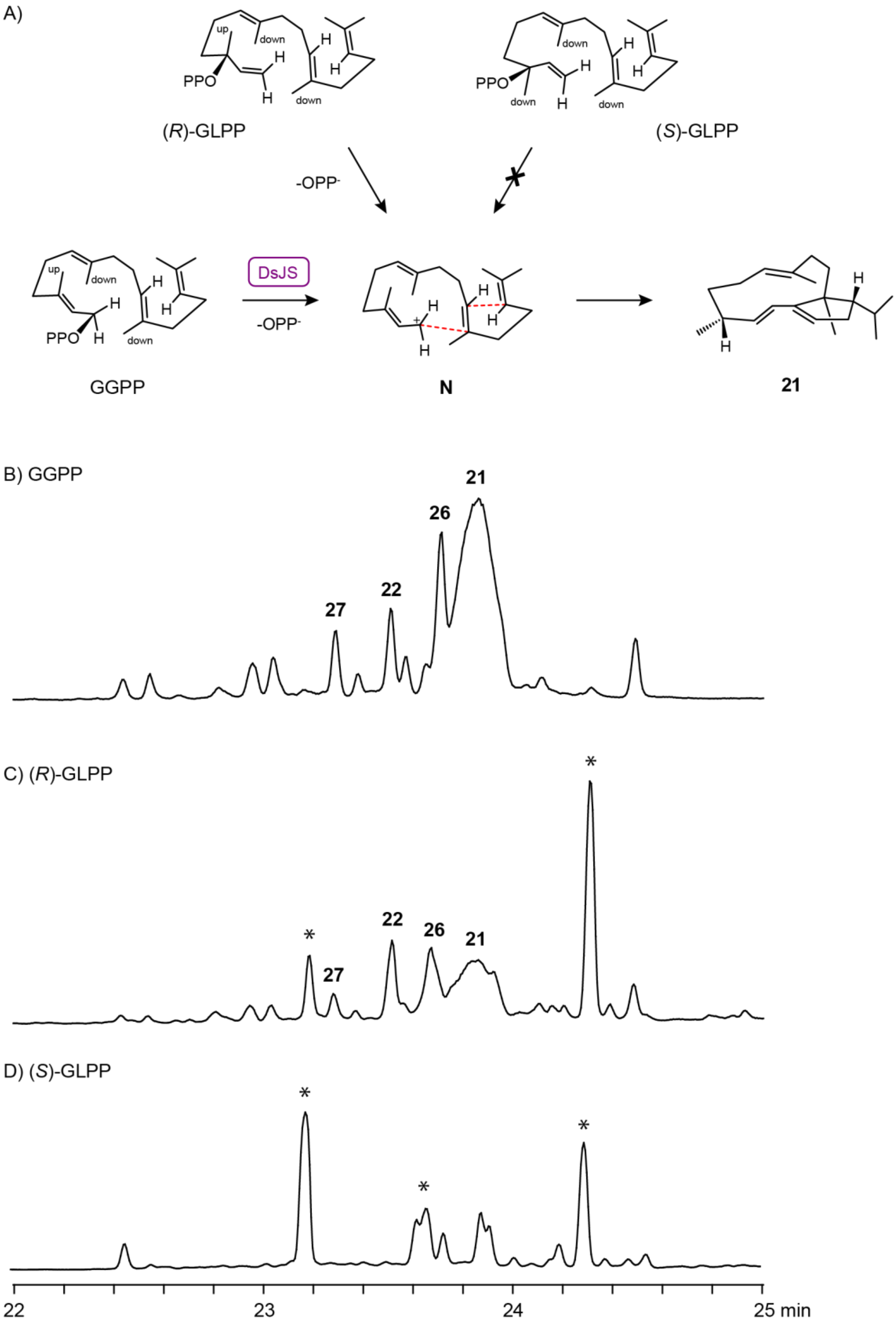

Figure S132. The starting conformation of GGPP in the biosynthesis of **21** and **22** by CjNdtS (Scheme 3 of main text). A) Proposed substrate conformations of GGPP, (*R*)- and (*S*)-GLPP in the biosynthesis of **21** and **22** by CjNdtS. GC/MS analysis of the crude extracts obtained from the incubation of B) (*R*)-GLPP and C) (*S*)-GLPP with CjNdtS, showing more efficient product formation from (*R*)-GLPP. Asterisks indicate spontaneous hydrolysis products. The peak broadening in B) and C) is a consequence of the thermal behaviour of **21**.

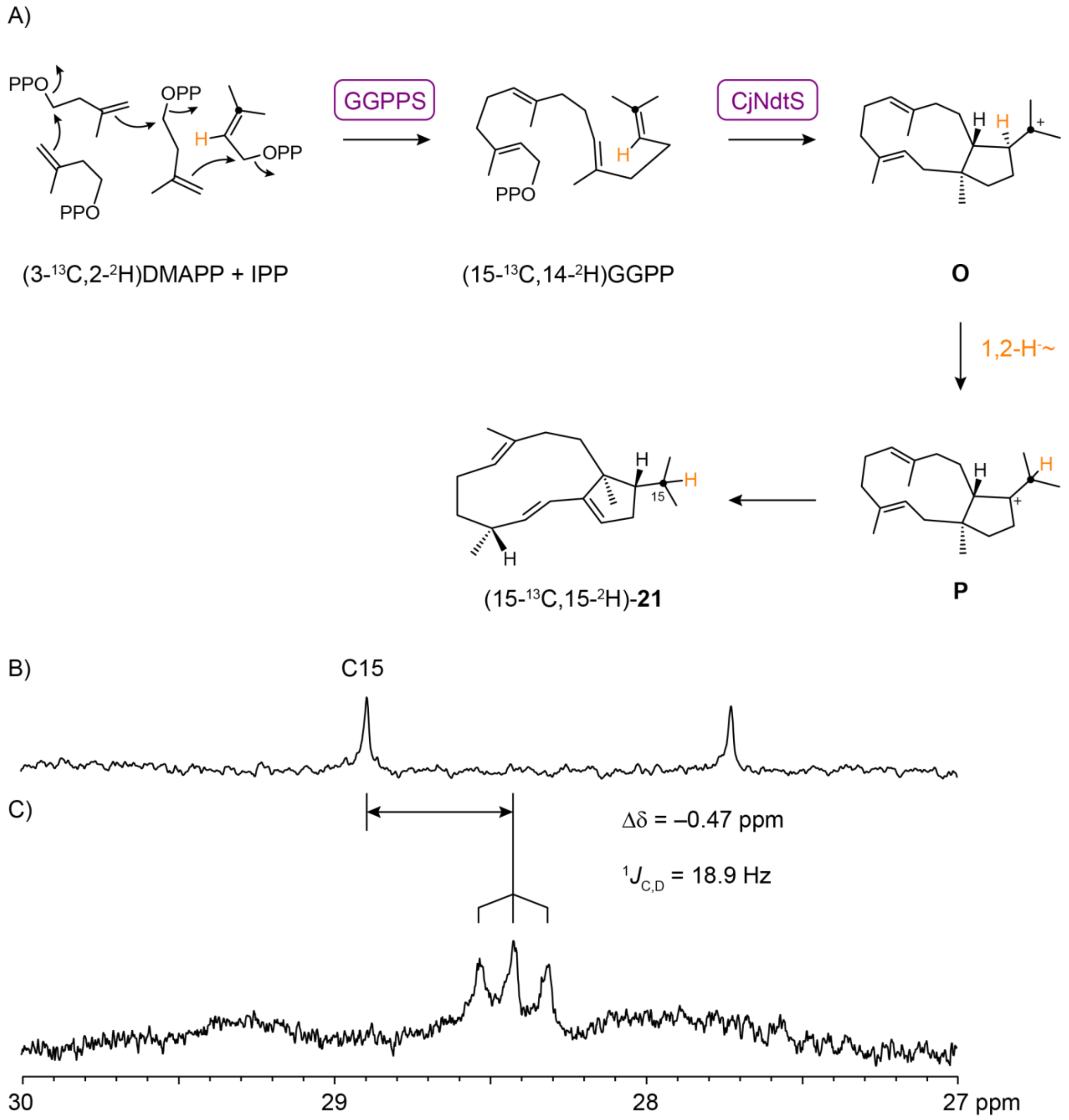

Figure S133. The 1,2-hydride shift from **O** to **P** in the biosynthesis of **21** by CjNdtS (Scheme 3 of main text). A) The enzymatic conversion of (3-^{13}C,2-^{2}H)DMAPP and IPP into labelled **21** with GGPPS and CjNdtS. Partial ^{13}C-NMR spectra showing the region for C15 of B) unlabelled **21** and C) labelled **21**. The upfield shifted triplet for C15 ($\Delta\delta$ = –0.47 ppm, $^{1}J_{C,D}$ = 18.9 Hz) indicates a deuterium migration from C14 to C15, supporting the 1,2-hydride shift from **O** to **P**. Black dots represent ^{13}C-labelled carbons and orange hydrogens indicate deuterium substitutions.

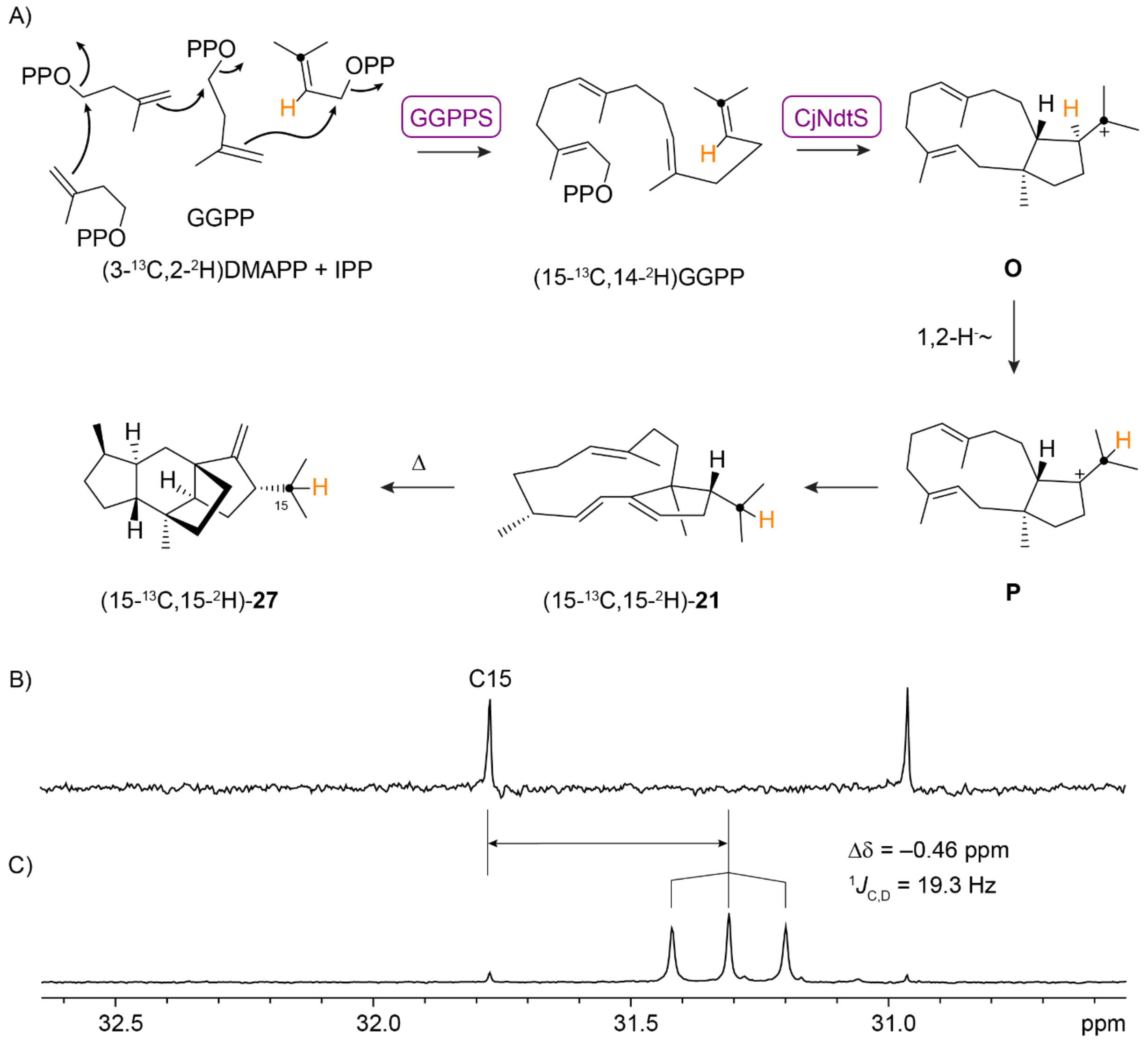

Figure S134. The 1,2-hydride shift from **O** to **P** in the biosynthesis of **21** by CjNdtS (Scheme 3 of main text). A) The enzymatic conversion of (3-^{13}C,2-^{2}H)DMAPP and IPP into labelled **21** with GGPPS and CjNdtS, followed by a thermal reaction to obtain labelled **27**. Partial ^{13}C-NMR spectra showing the region for C15 of B) unlabelled **27** and C) labelled **27**. The upfield shifted triplet for C15 ($\Delta\delta$ = –0.46 ppm, $^{1}J_{C,D}$ = 19.3 Hz) indicates a deuterium migration from C14 to C15, supporting the 1,2-hydride shift from **O** to **P**. Black dots represent ^{13}C-labelled carbons and orange hydrogens indicate deuterium substitutions.

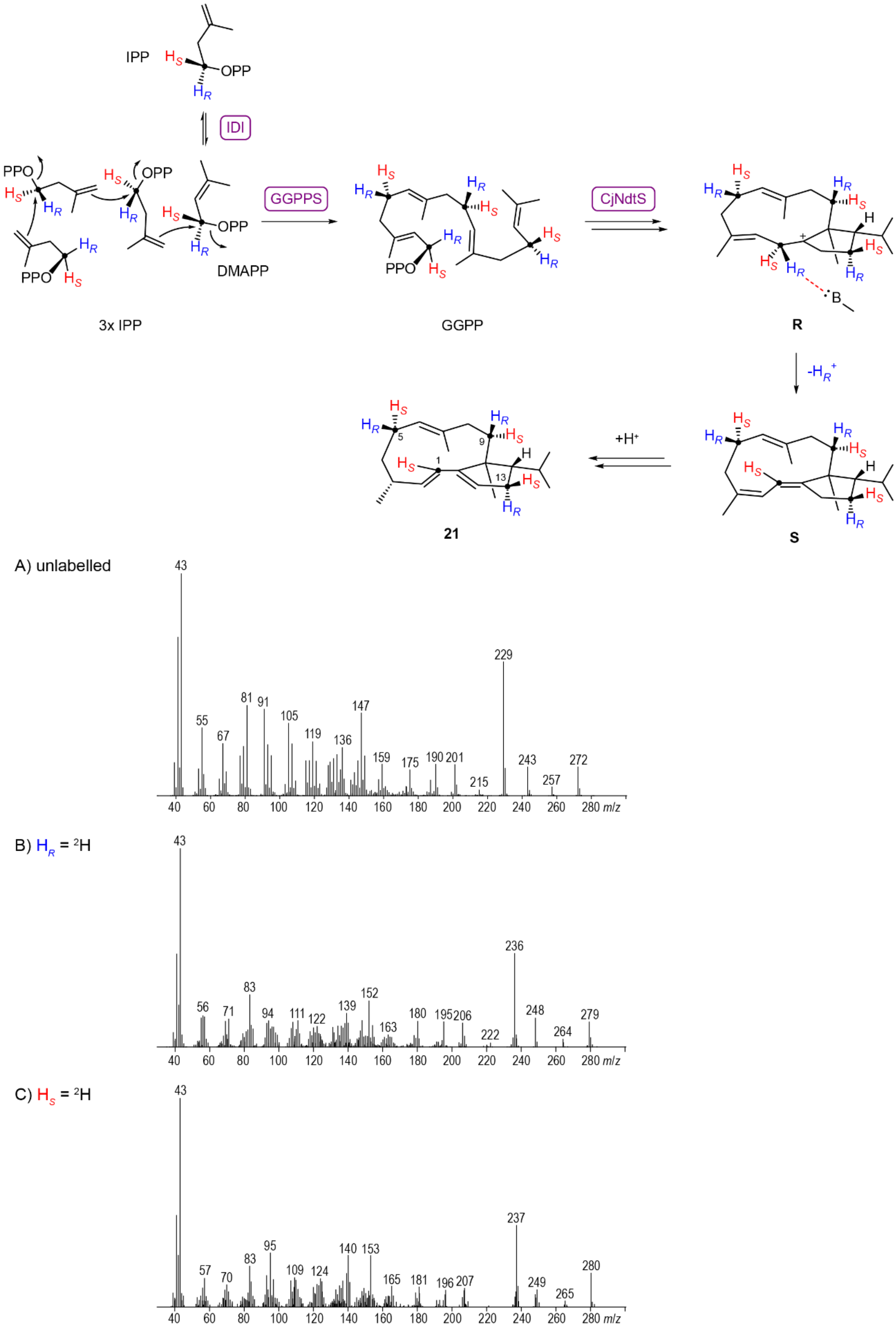

Figure S135. The deprotonation from **R** to **S** in the biosynthesis of **21** by CjNdtS. EI mass spectra of A) unlabelled **21**, B) labelled **21** obtained from (*R*)-(1-^{13}C,1-^{2}H)IPP and C) from (*S*)-(1-^{13}C,1-^{2}H)IPP with IDI, GGPPS and CjNdtS. The molecular ion at *m*/*z* 279 in B) indicates loss of the 1-*pro*-*R* proton in the deprotonation from **R** to **S**, while the molecular ion at *m*/*z* 280 in C) shows retainment of the 1-*pro*-*S* hydrogen.

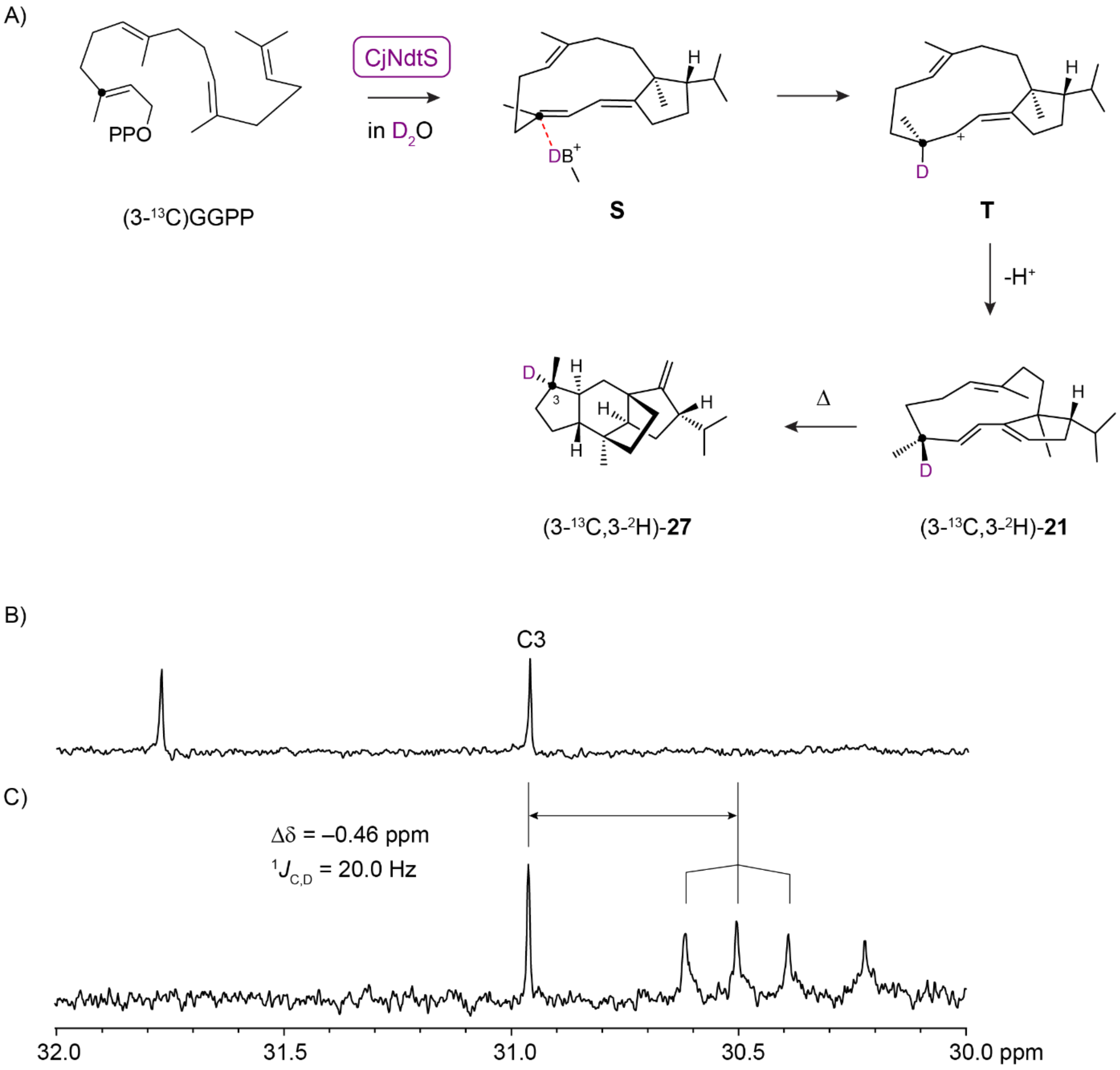

Figure S136. The reprotonation at C3 in the biosynthesis of **21** by CjNdtS. A) Enzymatic conversion of (3-^{13}C)GGPP in D_2O buffer into **21**, followed by thermal reaction to **27**. ^{13}C-NMR spectra showing the region for C3 of B) unlabelled **27**, and C) labelled **27** obtained from (3-^{13}C)GGPP with CjNdtS in D_2O. The upfield shifted triplet for C3 in C) confirms the reprotonation at this carbon in the biosynthesis of **21**. Black dots indicate ^{13}C-labelled carbons.

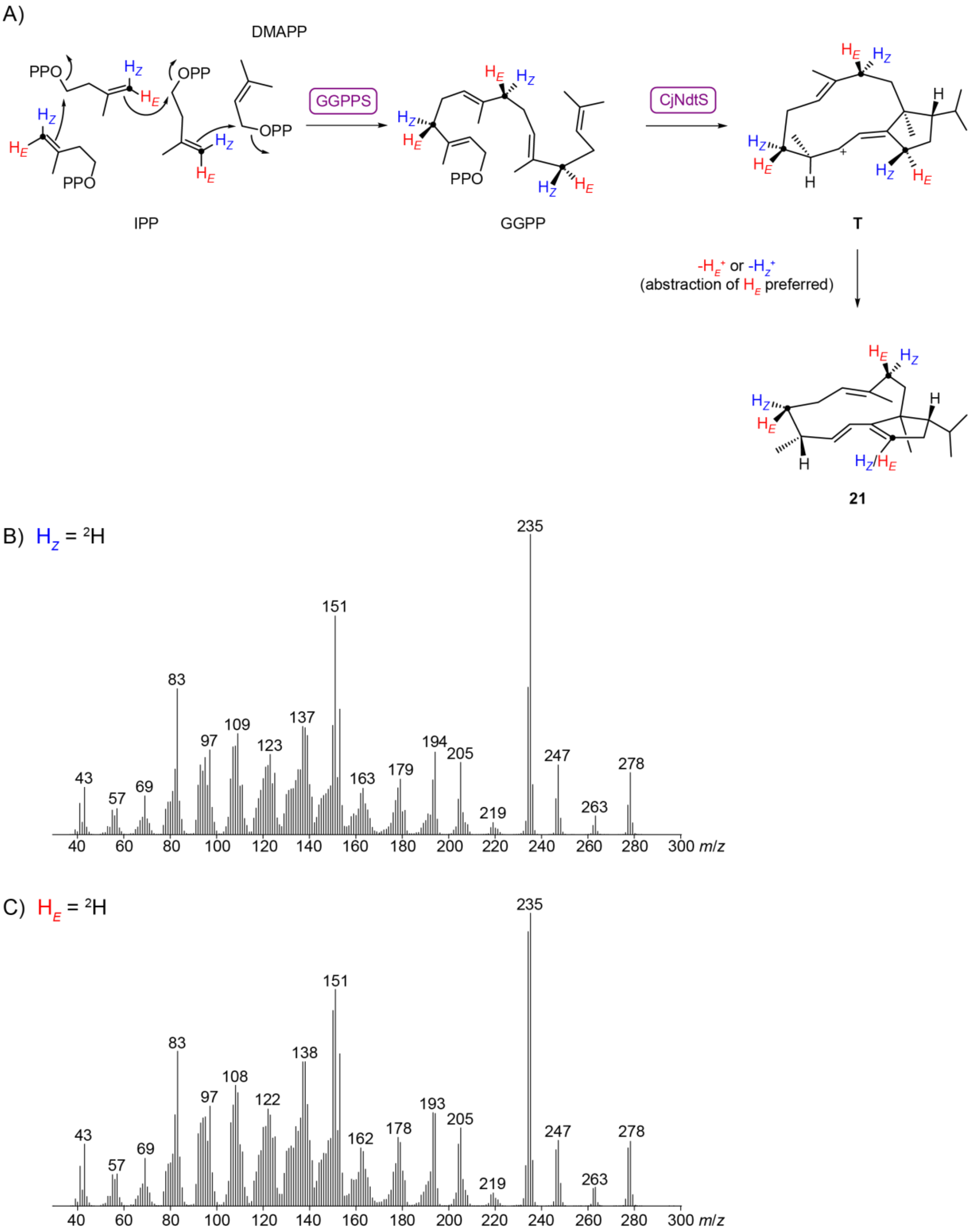

Figure S137. The stereoselectivity of the deprotonation from **T** to **21**. A) Biosynthesis of **21** from DMAPP and (*E*)- and (*Z*)-(4-^{13}C,4-^{2}H)IPP. B) EI mass spectrum of labelled **21** obtained from (*Z*)-(4-^{13}C,4-^{2}H)IPP. The large molecular ion peak at *m*/*z* 278 and the smaller peak at *m*/*z* 277 demonstrate the preferred retainment of H_Z and thus abstraction H_E (ca. 2:1 = 4:2 ratio). C) EI mass spectrum of labelled **21** obtained from (*E*)-(4-^{13}C,4-^{2}H)IPP. In this case, because of a kinetic isotope effect the proportion of H_E retained is larger (ca. 1:1 = 4:4 ratio). For the unlabelled compound the ratio may be in between (ca. 4:3 ratio for the abstraction of H_E versus H_Z).

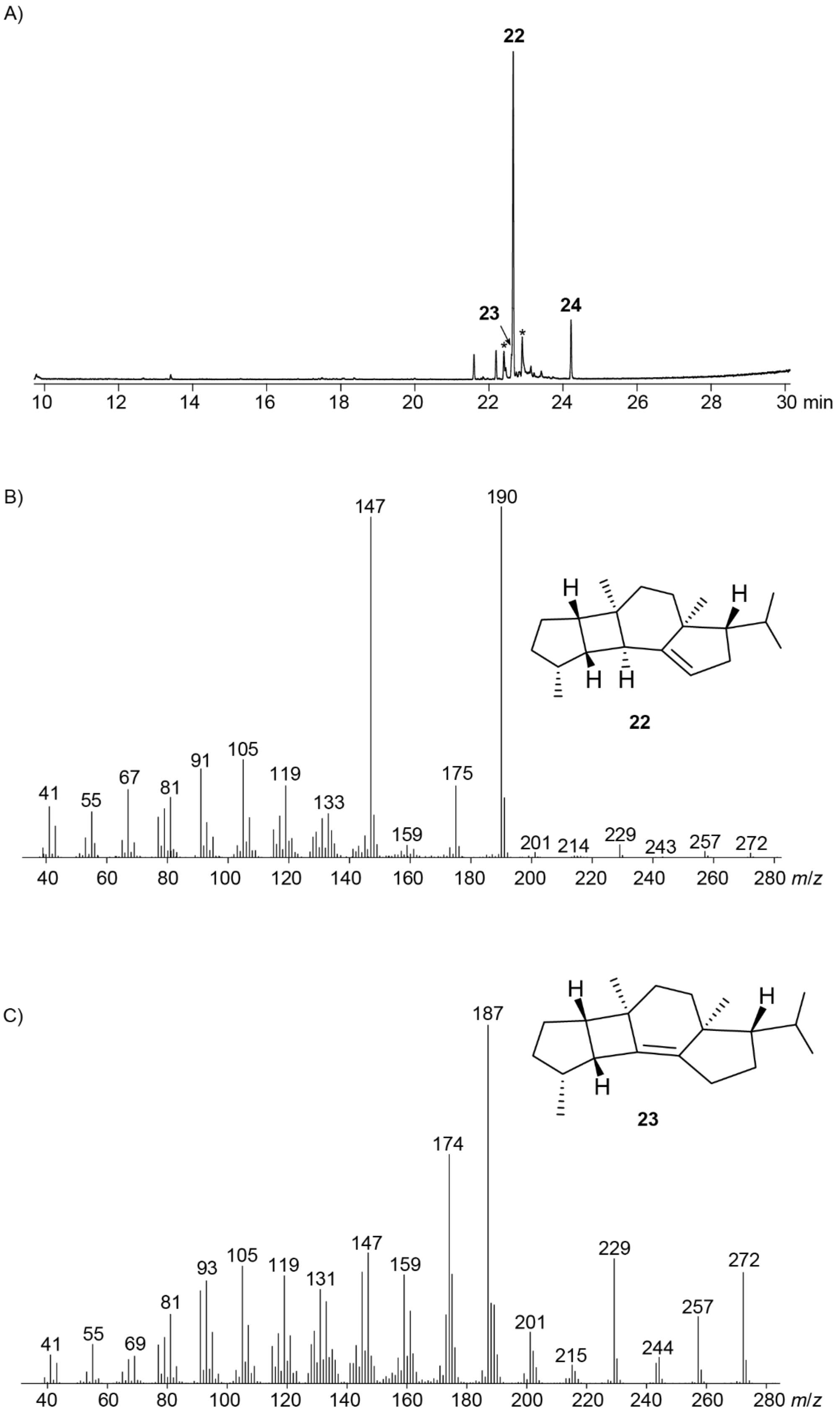

Figure S138. Enzymatic conversion of GGPP with DsJS (GPP, FPP and GFPP were not accepted). A) Total ion chromatogram of an extract from the incubation of GGPP with DsJS. B) EI mass spectrum of japonene A (**22**), and C) EI mass spectrum of japonene B (**23**). Asterisks indicate spontaneous lysis products of GGPP.

Preparative scale incubation with recombinant DsJS and compound isolation

A preparative scale enzymatic conversion of GGPP (100 mg, 200 μmol) was conducted in incubation buffer (60 mL) with the addition of an enzyme preparation of DsJS (15 mL, 1.5 mg mL^{-1}), followed by incubation for 16 h at 30 °C. The reaction mixture was extracted with n-pentane (3 x 100 mL), the combined extracts were dried with $MgSO_4$ and the solvent was evaporated. The crude product was purified via column chromatography on silica gel with a solvent gradient from pure pentane to a mixture of pentane:Et_2O (2:1) to obtain pure **24** (0.4 mg, 1.5 μmol, 0.8%) and an unpolar fraction containing a mixture of **22** and **23**. This mixture was separated using column chromatography on $AgNO_3$ impregnated silica gel with pentane to obtain **22** (1.2 mg, 4.4 μmol, 2.2%) and **23** (0.2 mg, 0.1 μmol, 0.1%).

Japonene A (22). Optical rotation: $[\alpha]_D^{25}$ = +8.3 (*c* 0.12, CH_2Cl_2). NMR data were identical to **22** isolated from CjNdtS.

Japonene B (23). TLC (pentane): R_f = 0.90. GC (HP-5MS): *I* = 1956. MS (EI, 70 eV): *m/z* (%) = 41 (2), 55 (2), 69 (2), 81 (4), 93 (6), 105 (7), 119 (6), 131 (5), 147 (7), 159 (6), 174 (13), 187 (20), 201 (3), 229 (7), 257 (3), 272 (6). IR (diamond ATR): $\tilde{v}$ = 3358 (w), 3187 (w), 2953 (s), 2924 (s), 2852 (s), 1731 (w), 1658 (m), 1632 (m), 1468 (w), 1373 (w), 1260 (w), 808 (w) cm^{-1}. HR-MS (Q-TOF, 70 eV): calc. $[C_{20}H_{32}]^+$ *m/z* = 272.2499; found: *m/z* = 272.2505. Optical rotation: $[\alpha]_D^{25}$ = –9.3 (*c* 0.02, CH_2Cl_2). NMR data are given in Table S20.

Anaerol B (24). Optical rotation: $[\alpha]_D^{25}$ = +10.0 (*c* 0.03, CH_2Cl_2). NMR data were identical to those reported before.[4]

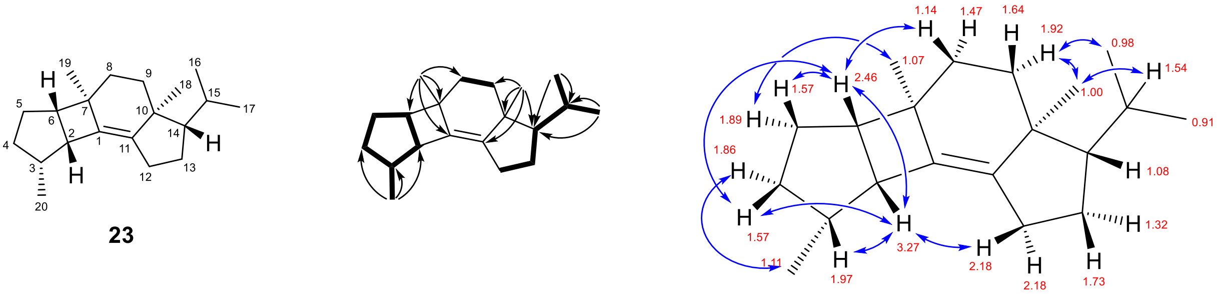

Figure S139. Structure elucidation of japonene B (**23**). Bold: ^{1}H,^{1}H-COSY, single headed arrows: key HMBC, and double headed arrows: NOESY correlations. Carbon numbering follows GGPP numbering to indicate the origin of each carbon.

Table S20. NMR data of japonene B (**23**) in C_6D_6 recorded at 298 K.

C[a]	type	^{13}C[b]	^{1}H[b]
1	C_q	139.30	–
2	CH	53.83	3.27 (dd, J = 6.9, 6.9)
3	CH	38.27	1.97 (m)
4	CH_2	36.69	1.86 (m)
			1.57 (m)
5	CH_2	29.29	1.89 (m)
			1.57 (m)
6	CH	50.50	2.46 (dd, J = 6.9, 6.9)
7	C_q	44.05	–
8	CH_2	39.33	1.47 (dd, J = 12.1, 4.2)
			1.14 (dd, J = 13.0, 6.2)
9	CH_2	35.36	1.92 (dd, J = 14.2, 4.7)
			1.64 (td, J = 14.0, 5.3)
10	C_q	43.14	–
11	C_q	144.57	–
12	CH_2	25.14	2.18 (m)
13	CH_2	27.72	1.73 (m)
			1.32 (m)
14	CH	56.37	1.08 (m)
15	CH	30.27	1.54 (m)
16	CH_3	22.81	0.91 (d, J = 6.7)
17	CH_3	23.68	0.99 (d, J = 6.9)
18	CH_3	25.05	1.00 (s)
19	CH_3	16.25	1.07 (s)
20	CH_3	17.55	1.11 (d, J = 6.4)

[a] Carbon numbering as shown in Figure S139 indicates the origin of each carbon from GGPP by same number. [b] Chemical shifts δ in ppm, multiplicity: s = singlet, d = doublet, t = triplet, m = multiplet, coupling constants J are given in Hertz.

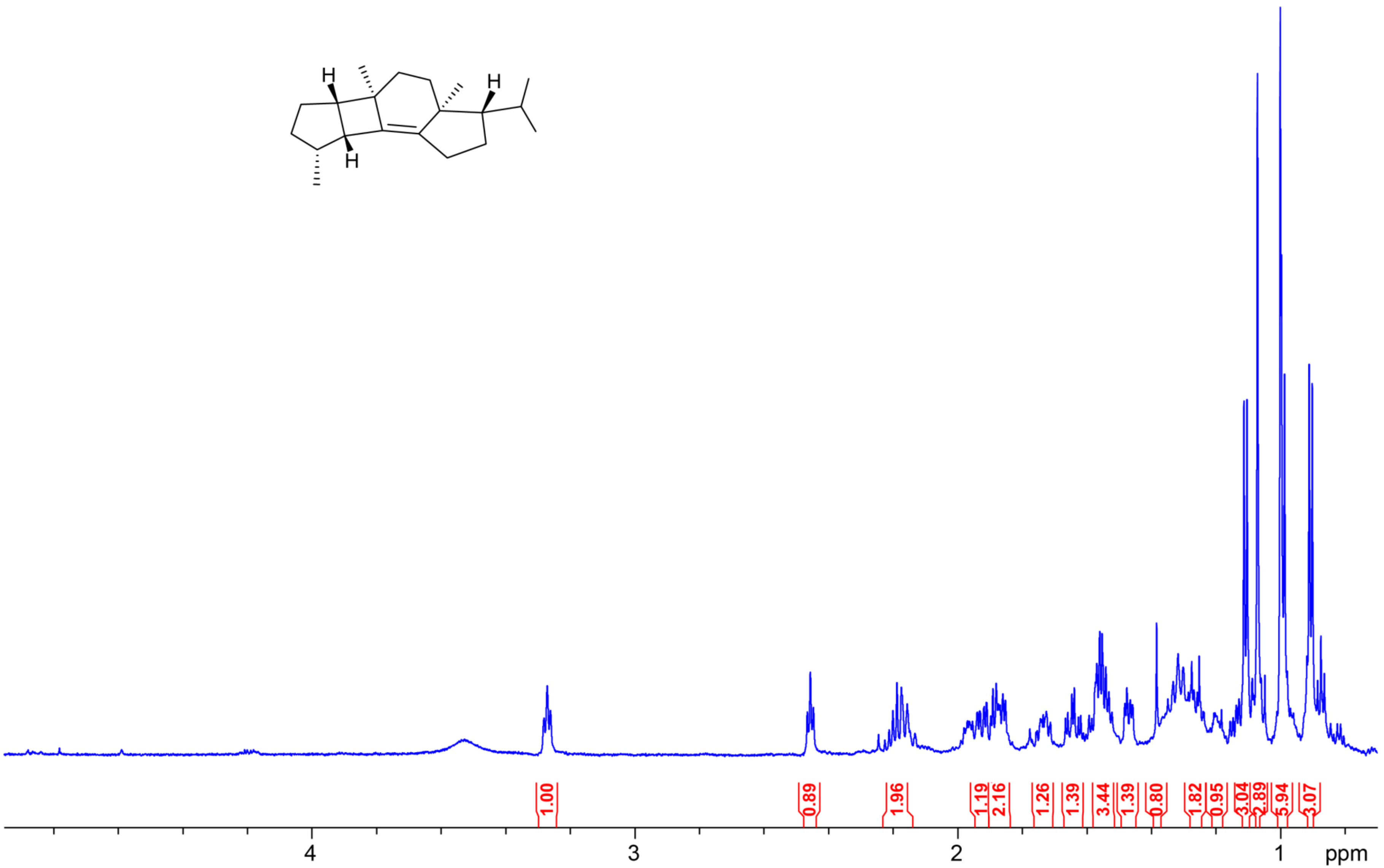

Figure S140. ^{1}H-NMR spectrum of **23** (700 MHz, C_6D_6).

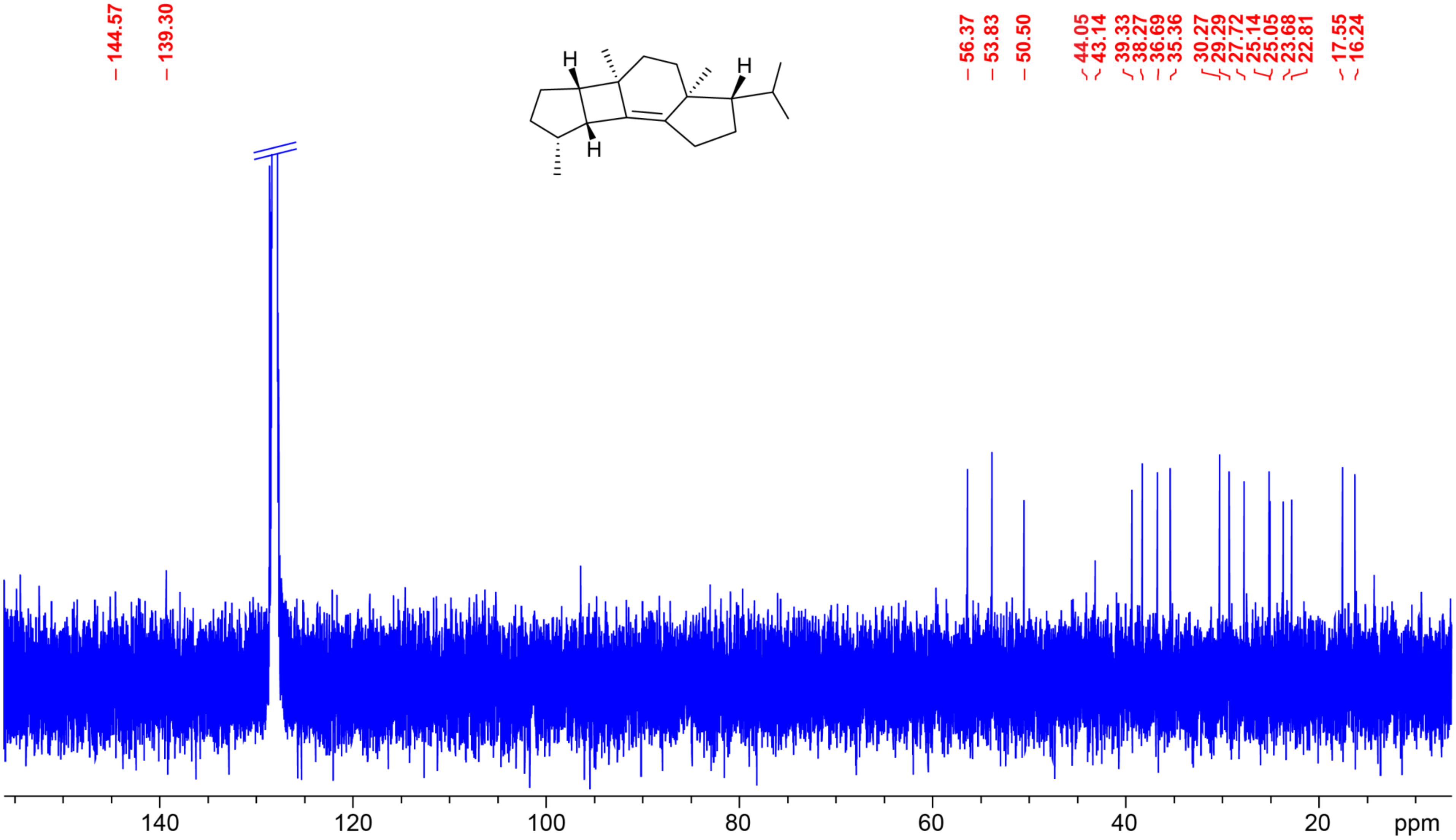

Figure S141. ^{13}C-NMR spectrum of **23** (176 MHz, C_6D_6).

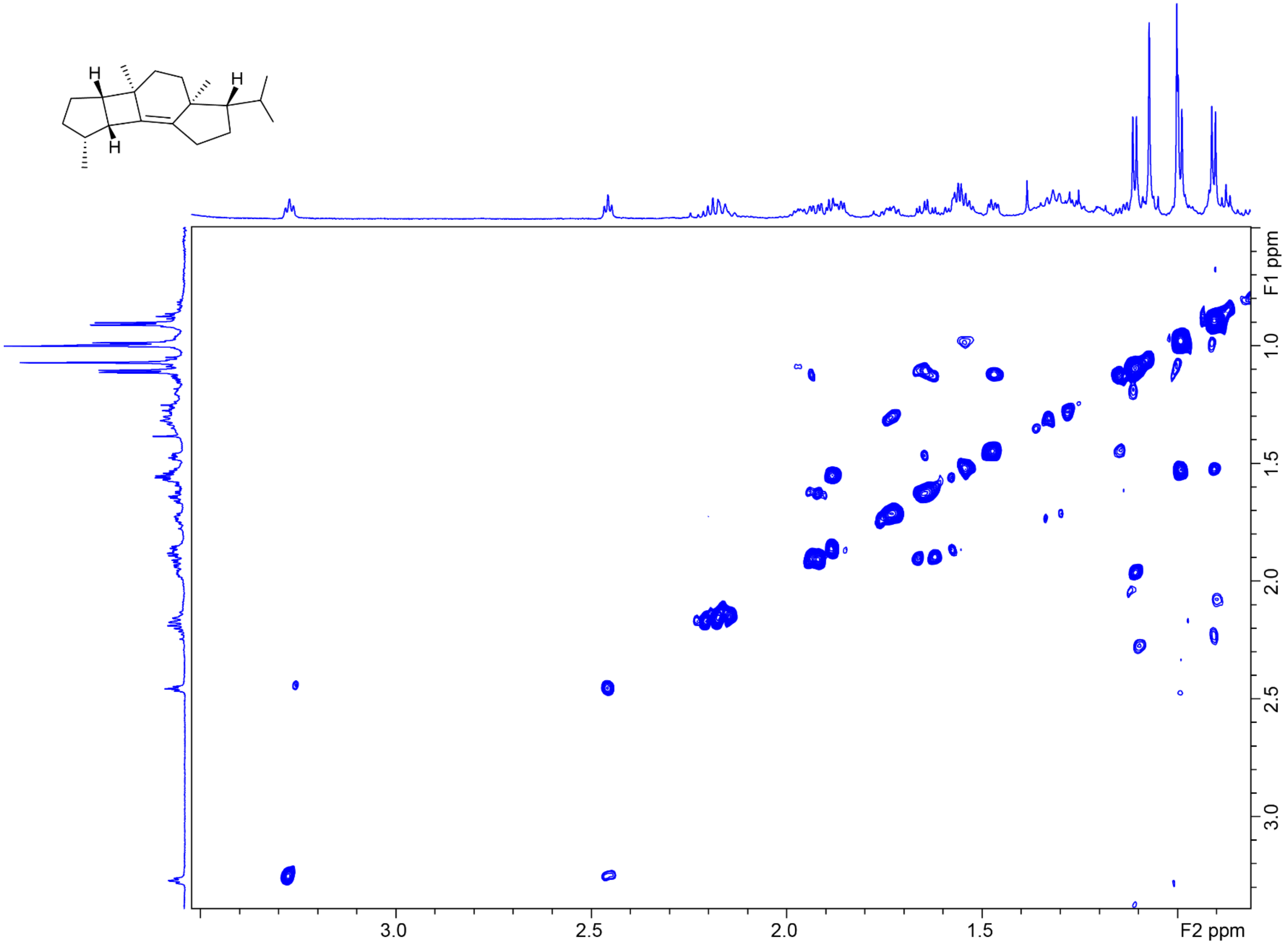

Figure S142. ^{1}H-^{1}H-COSY spectrum (C_6D_6) of **23**.

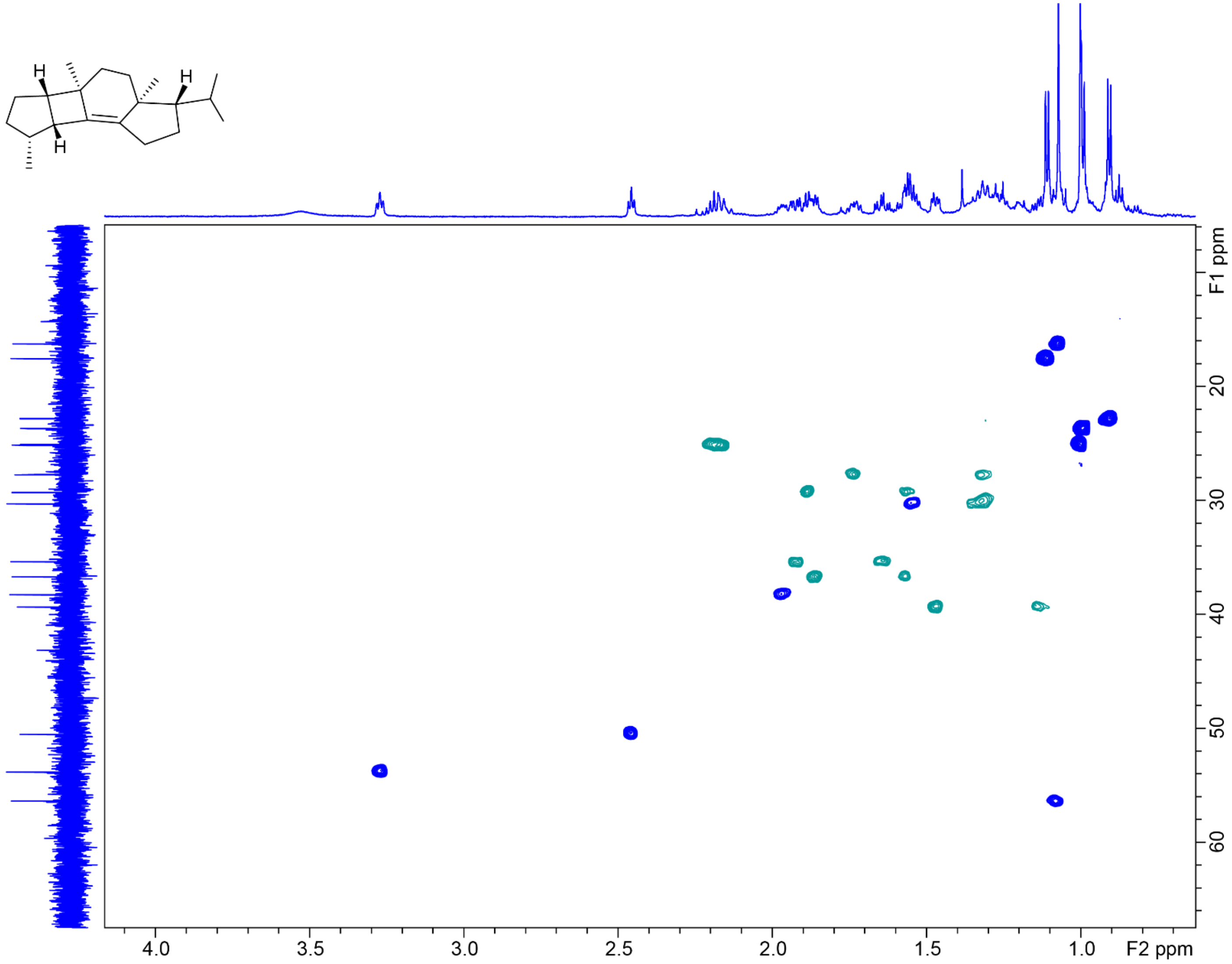

Figure S143. HSQC spectrum (C_6D_6) of **23**.

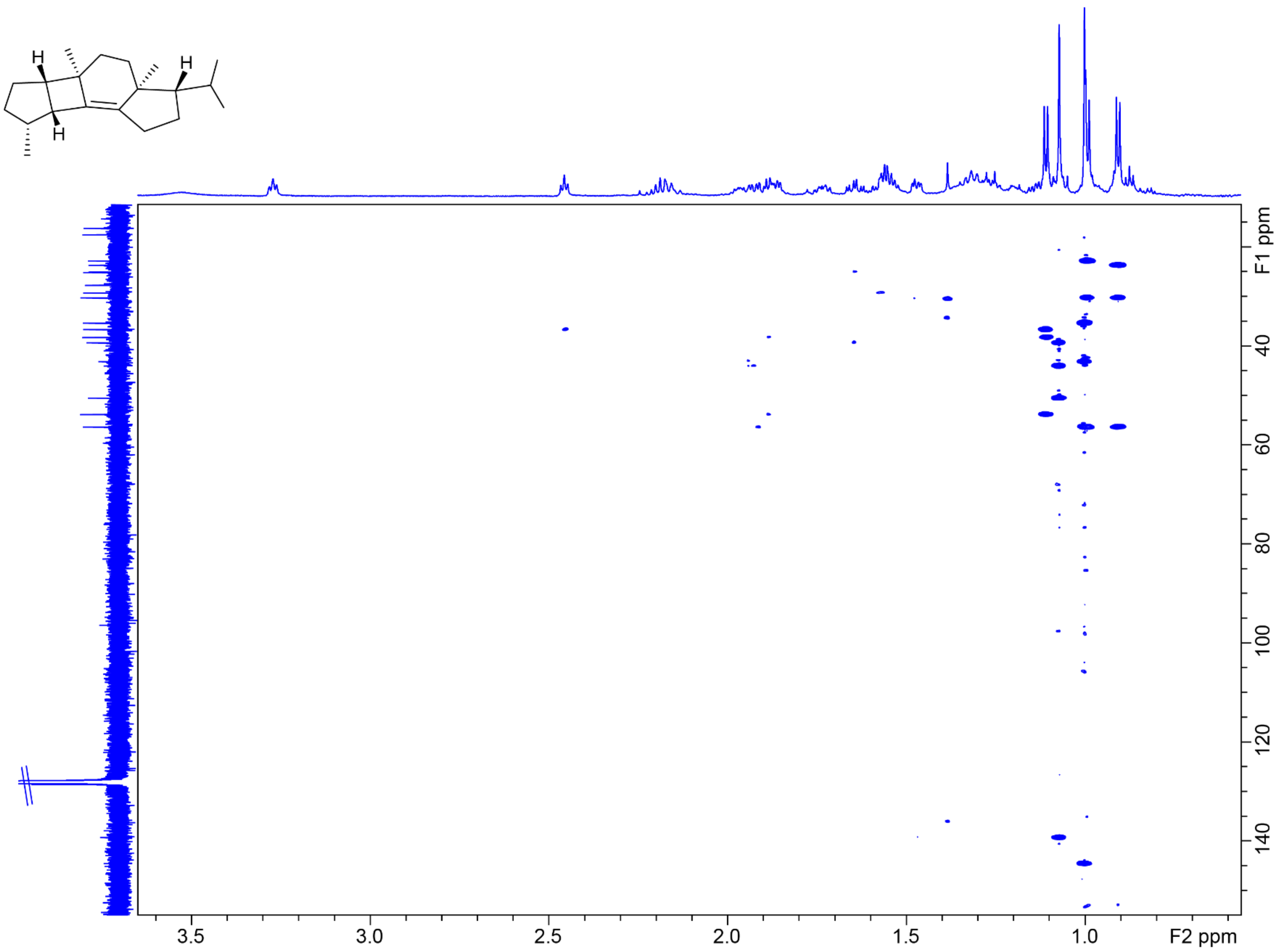

Figure S144. HMBC spectrum (C_6D_6) of **23**.

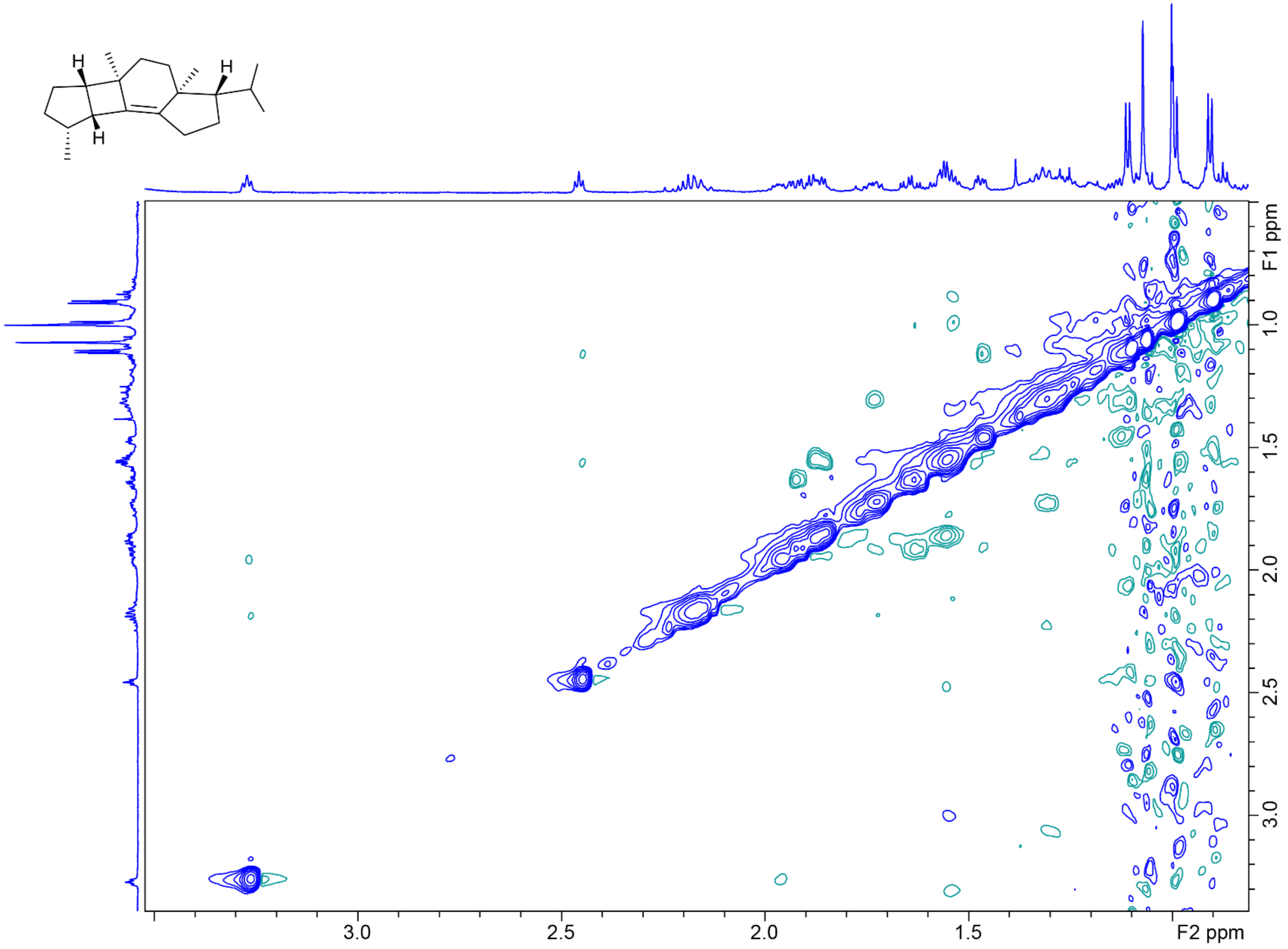

Figure S145. NOESY spectrum (C_6D_6) of **23**. The poor spectral quality is a result of the low isolated yield (0.2 mg) and the relative configuration of **23** is tentatively assigned based on biosynthetic considerations.

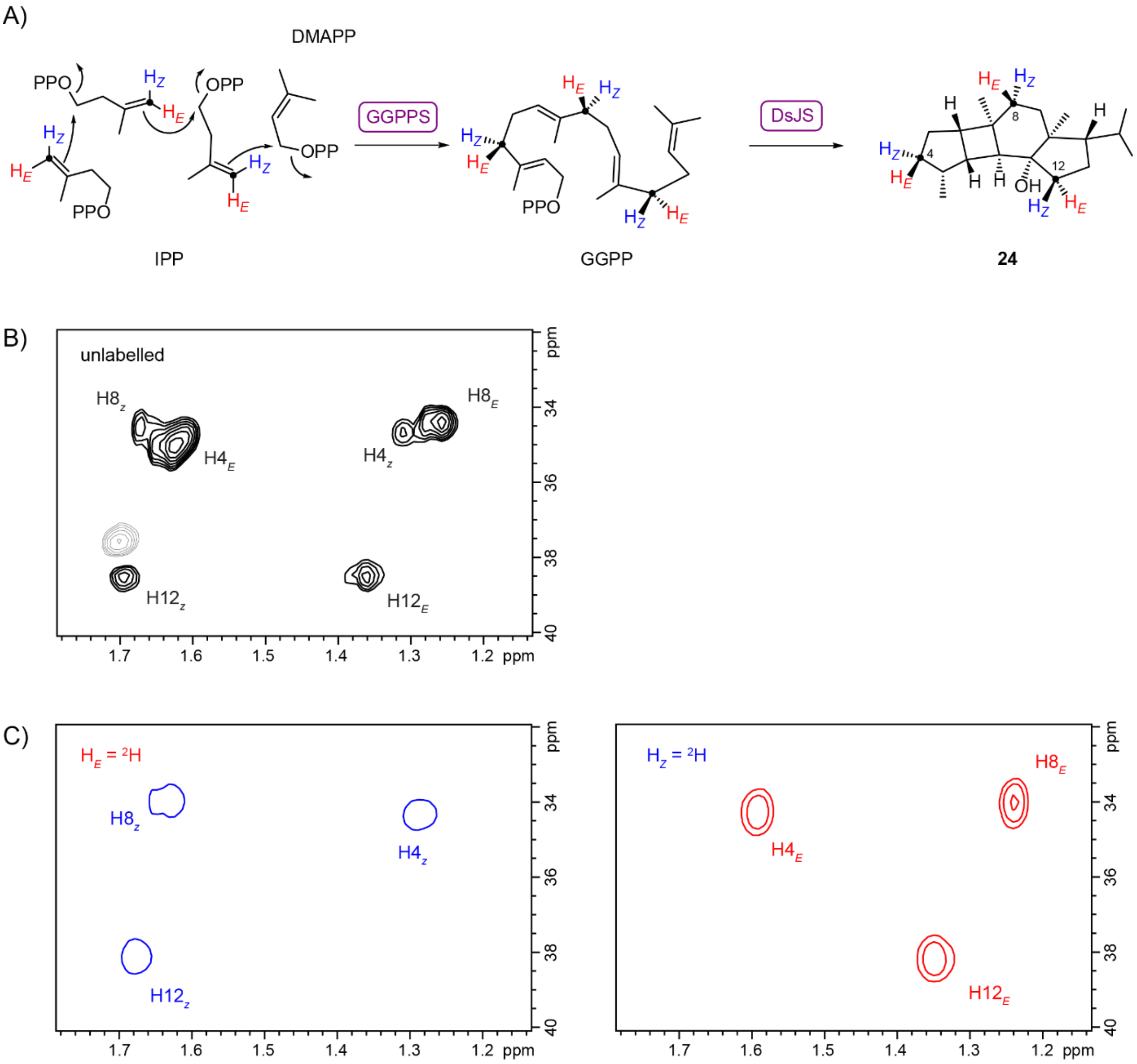

Figure S146. The absolute configuration of **24**. A) Cyclisation of labelled GGPP obtained from (*E*)-(4-^{13}C,4-^{2}H)IPP (red H = ^{2}H) or (*Z*)-(4-^{13}C,4-^{2}H)IPP (blue H = ^{2}H) plus DMAPP with GGPPS and DsJS. B) Partial HSQC spectrum of unlabelled **24** showing the region for C4, C8 and C12. C) HSQC spectrum of labelled **24** obtained from (*E*)-(4-^{13}C,4-^{2}H)IPP (left) and from (*Z*)-(4-^{13}C,4-^{2}H)IPP (right). These data point to the shown absolute configuration of **24**.

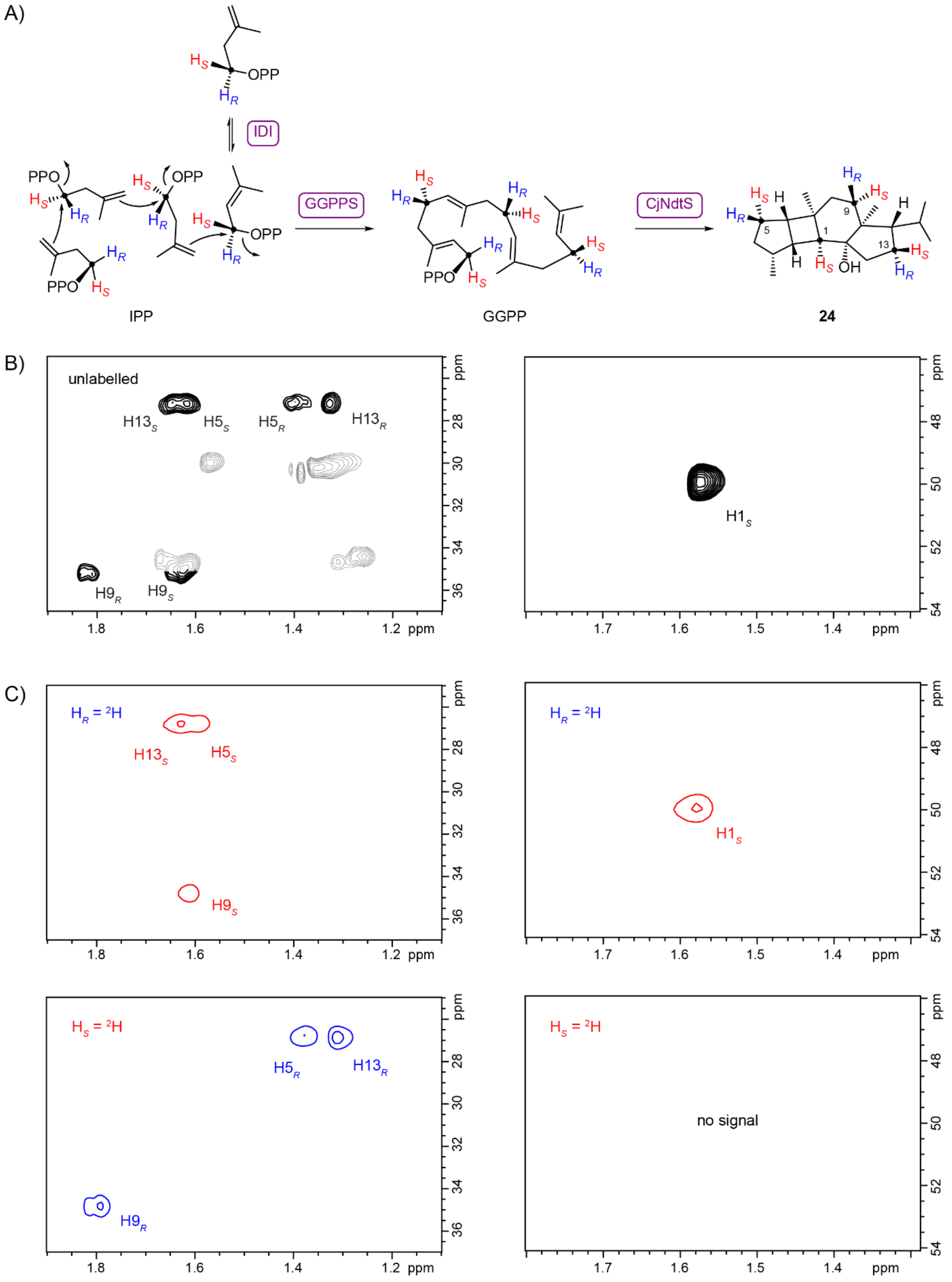

Figure S147. The absolute configuration of **24**. A) Cyclisation of labelled GGPP obtained from (*R*)-(1-^{13}C,1-^{2}H)IPP (red H = ^{2}H) or (*S*)-(1-^{13}C,1-^{2}H)IPP (blue H = ^{2}H) with GGPPS, IDI and DsJS. B) Partial HSQC spectra of unlabelled **24** showing the region for C1, C5, C9 and C13. C) Partial HSQC spectra of labelled **24** obtained from (*R*)-(1-^{13}C,1-^{2}H)IPP (top) and from (*S*)-(1-^{13}C,1-^{2}H)IPP (bottom). These data point to the shown absolute configuration of **24**.

Chemical correlation of 24 to 22 and 23

A solution of $SOCl_2$ (**24**, 1.6 mg, 13.8 µmol) in CH_2Cl_2 (0.1 mL) was added to a solution of anaerol B (**24**, 0.1 mg, 0.35 µmol) in CH_2Cl_2 (0.1 mL). The reaction mixture was stirred for 0.5 h. The solvent was removed under reduced pressure. The reaction product was analysed by GC-MS.

A solution of anaerol B (**24**, 0.1 mg, 0.35 µmol) in CH_3CN (0.1 mL) was added to a solution of Burgess' reagent (1.2 mg, 4.0 µmol) in CH_3CN (0.05 mL). The reaction mixture was warmed to 50 °C for 1.5 h. TLC analysis at this stage showed no conversion of the starting material. Therefore, according to a reported procedure,[36] the solvent was removed under reduced pressure. The residue was dissolved in THF (0.1 mL) at room temperature and treated with NaH (1 mg)**.** The reaction mixture was stirred for 0.5 h. The solvent was removed, the residue was suspended in toluene (0.2 mL) and warmed to 100 °C for 1.5 h. After solvent removal, the reaction product was analysed by GC-MS, showing conversion of **24** into japonene A (**22**), but not japonene B (**23**).

Japonene B (**23**, 0.1 mg, 0.37 µmol) was dissolved in $CHCl_3$ (0.1 mL), followed by the addition of *p*-toluenesulfonic acid (1.0 mg) at room temperature. After 24 h, pentane (1 mL) was added and the organic layer was neutralised by washing with NaOH (0.5 M, 2x 1 mL), followed by washing with brine (1 mL). The organic layer was dried with $MgSO_4$ and filtered through a pad of silica gel to remove remaining traces of *p*-TsOH. After concentration by removal of solvents under reduced pressure the sample was analysed by GC-MS, showing full consumption of **23** with conversion into japonene A (**22**).

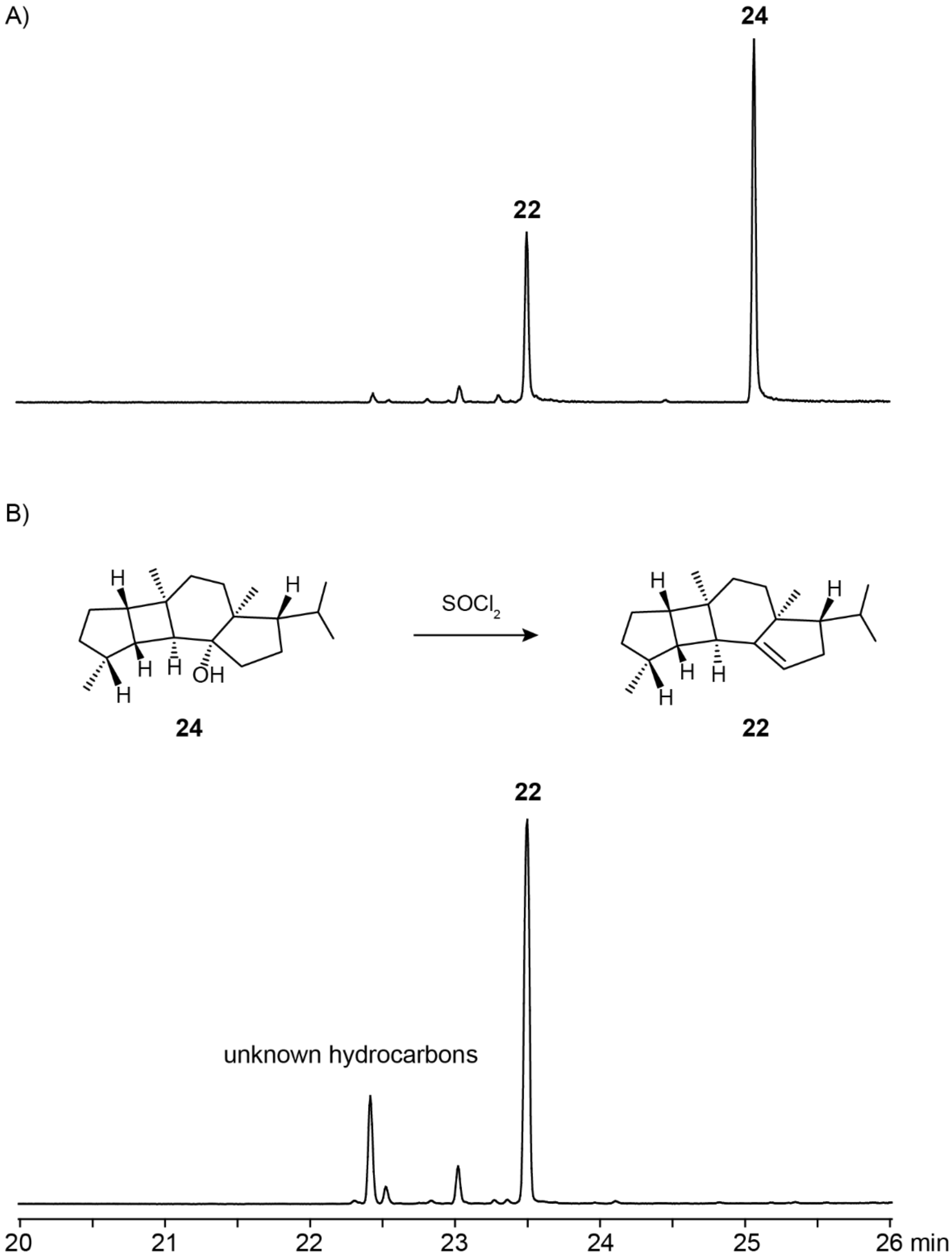

Figure S148. Chemical correlation of **24** to **22**. Total ion chromatograms of A) pure **24** (confirmed by NMR, **22** is formed in a thermal reaction in the injector) and B) the products obtained from **24** by treatment with $SOCl_2$. The major product is the expected *anti*-elimination product **22**, while no *syn*-elimination to **23** was observed.

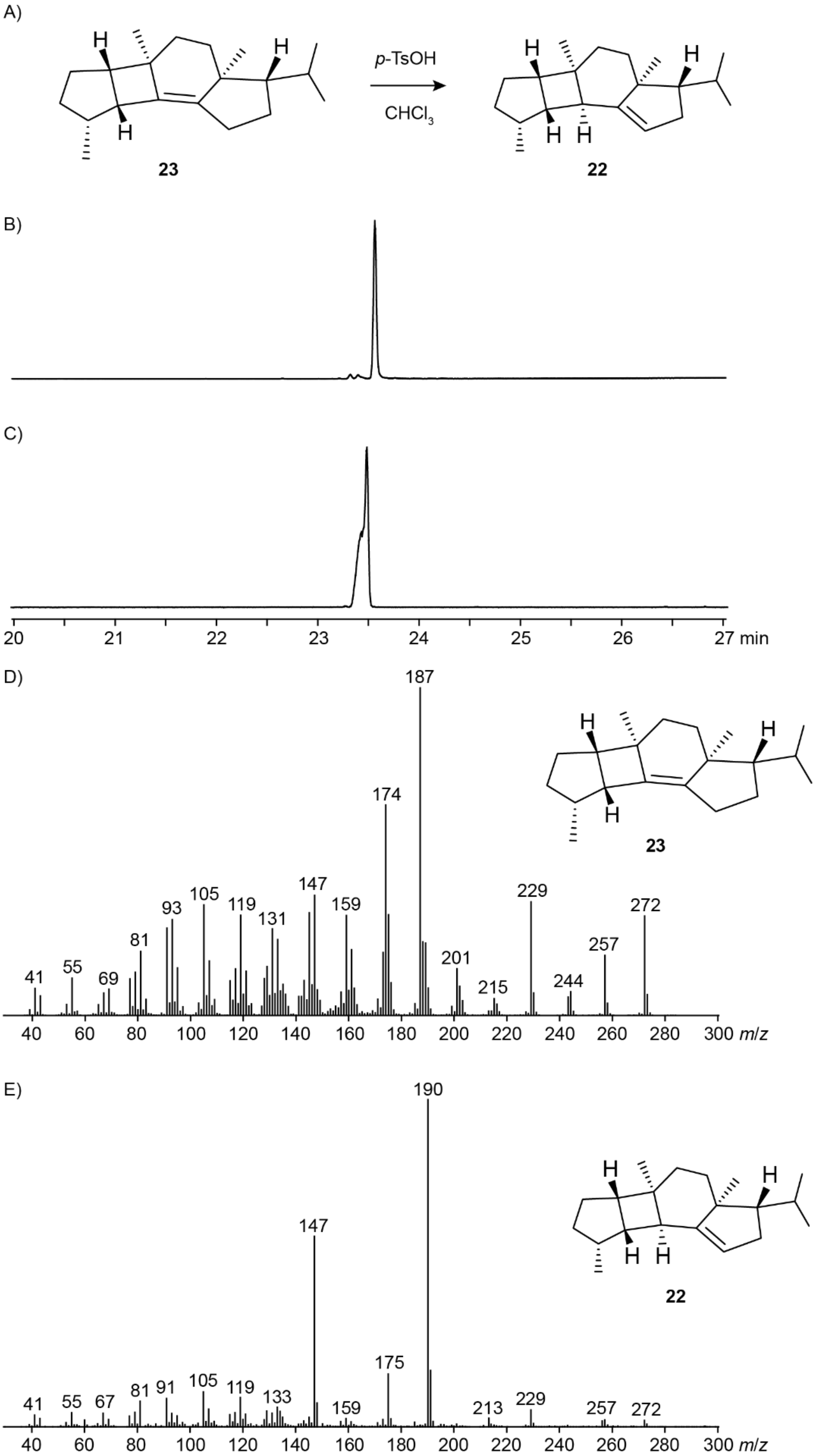

Figure S149. Chemical correlation of **23** to **22**. A) Acid catalysed conversion of **23** to **22**, B) total ion chromatogram of isolated **23** showing no presence of **22** in the sample, C) extracted ion chromatogram (*m*/*z* 190, representing the base peak ion of **22**) of the crude product of the acid catalysed isomerisation, D) EI mass spectrum of **23** (from the sample shown in B), E) EI mass spectrum of **22** (from the sample shown in C).

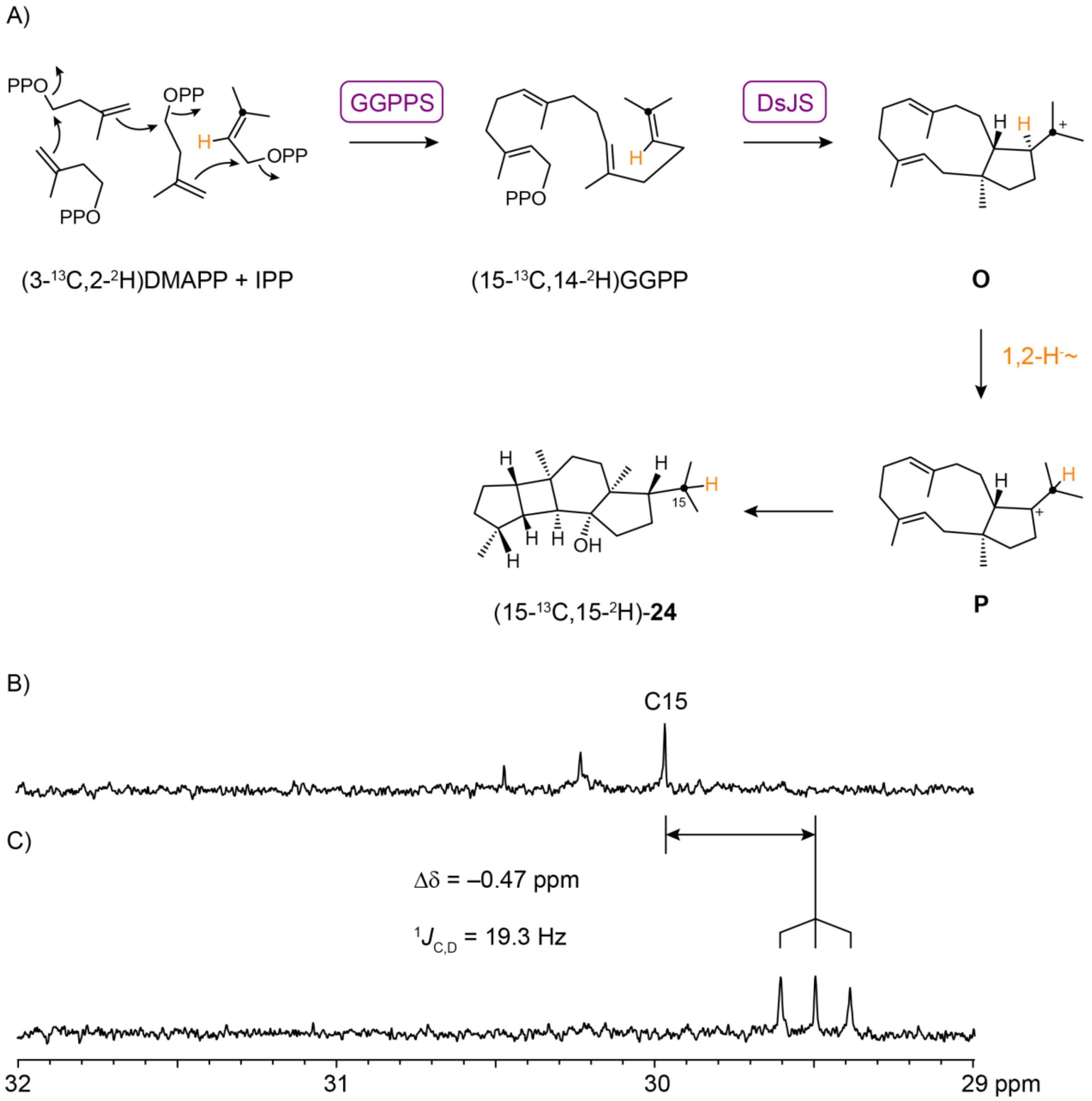

Figure S150. The 1,2-hydride shift from **O** to **P** in the biosynthesis of **24** by DsJS (Scheme 3 of main text). A) The enzymatic conversion of (3-^{13}C,2-^{2}H)DMAPP and IPP into labelled **24** with GGPPS and DsJS. Partial ^{13}C-NMR spectra showing the region for C15 of B) unlabelled **24** and C) labelled **24**. The upfield shifted triplet for C15 ($\Delta\delta$ = –0.47 ppm, $^{1}J_{C,D}$ = 18.9 Hz) indicates a deuterium migration from C14 to C15, supporting the 1,2-hydride shift from **O** to **P**. Black dots represent ^{13}C-labelled carbons and orange hydrogens indicate deuterium substitutions.

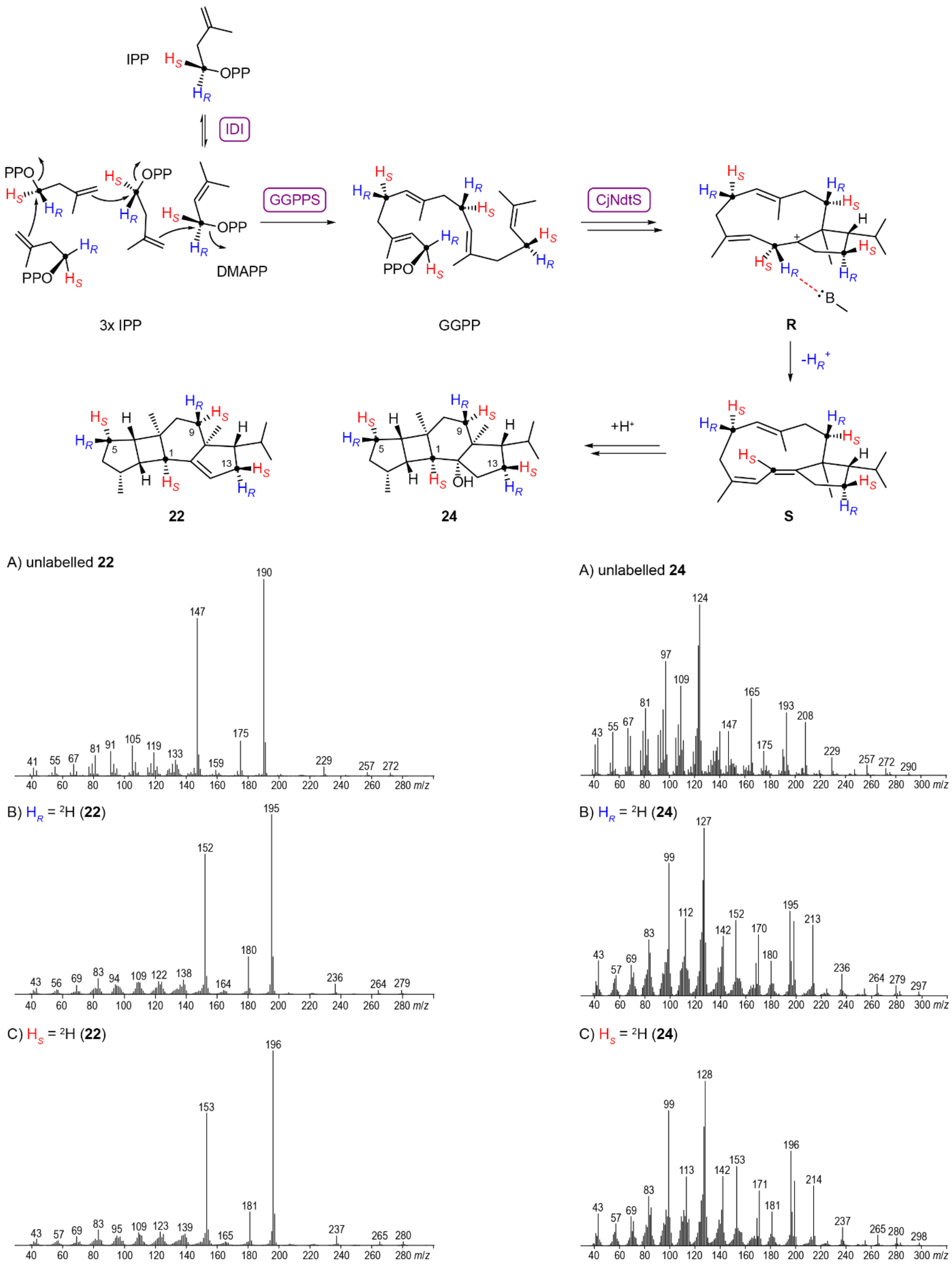

Figure S151. The deprotonation from **R** to **S** in the biosynthesis of **22** and **24** by DsJS. EI mass spectra of A) unlabelled **22** and **24**, B) labelled **22** and **24** obtained from (*R*)-(1-^{13}C,1-^{2}H)IPP and C) from (*S*)-(1-^{13}C,1-^{2}H)IPP with IDI, GGPPS and DsJS. The molecular ion at *m*/*z* 279 in B) indicates loss of the 1-*pro*-*R* proton in the deprotonation from **R** to **S**, while the molecular ion at *m*/*z* 280 in C) shows retainment of the 1-*pro*-*S* hydrogen.

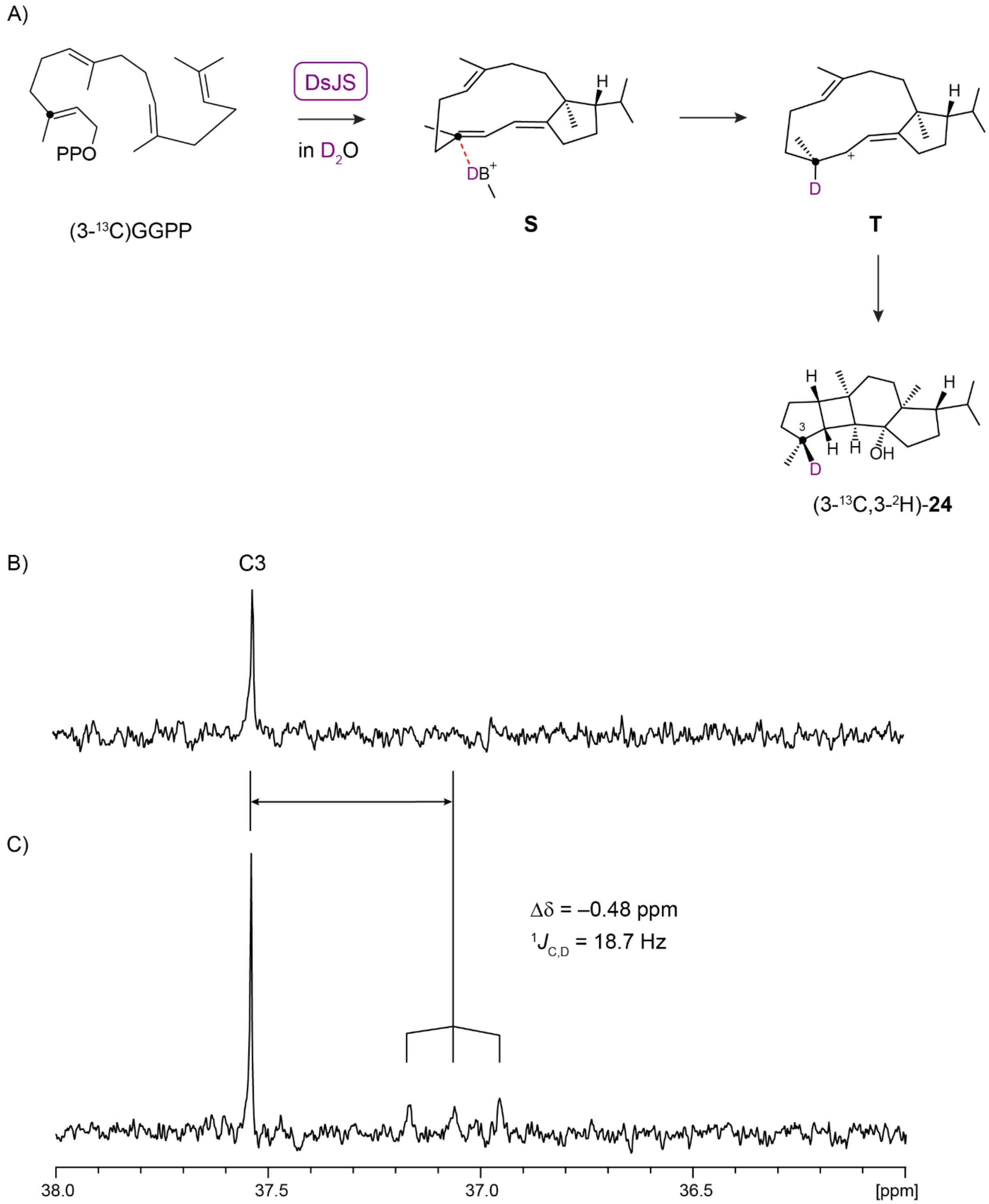

Figure S152. The reprotonation from **S** to **T** in the biosynthesis of **24** by DsJS. A) Enzymatic conversion of (3-^{13}C)GGPP by DsJS in D_2O buffer. ^{13}C-NMR spectra showing the region for C3 of B) unlabelled **24**, and C) labelled **24** obtained from (3-^{13}C)GGPP with DsJS in D_2O. The upfield shifted triplet for C3 in C) confirms the reprotonation at this carbon in the biosynthesis of **24**. Black dots indicate ^{13}C-labelled carbons.

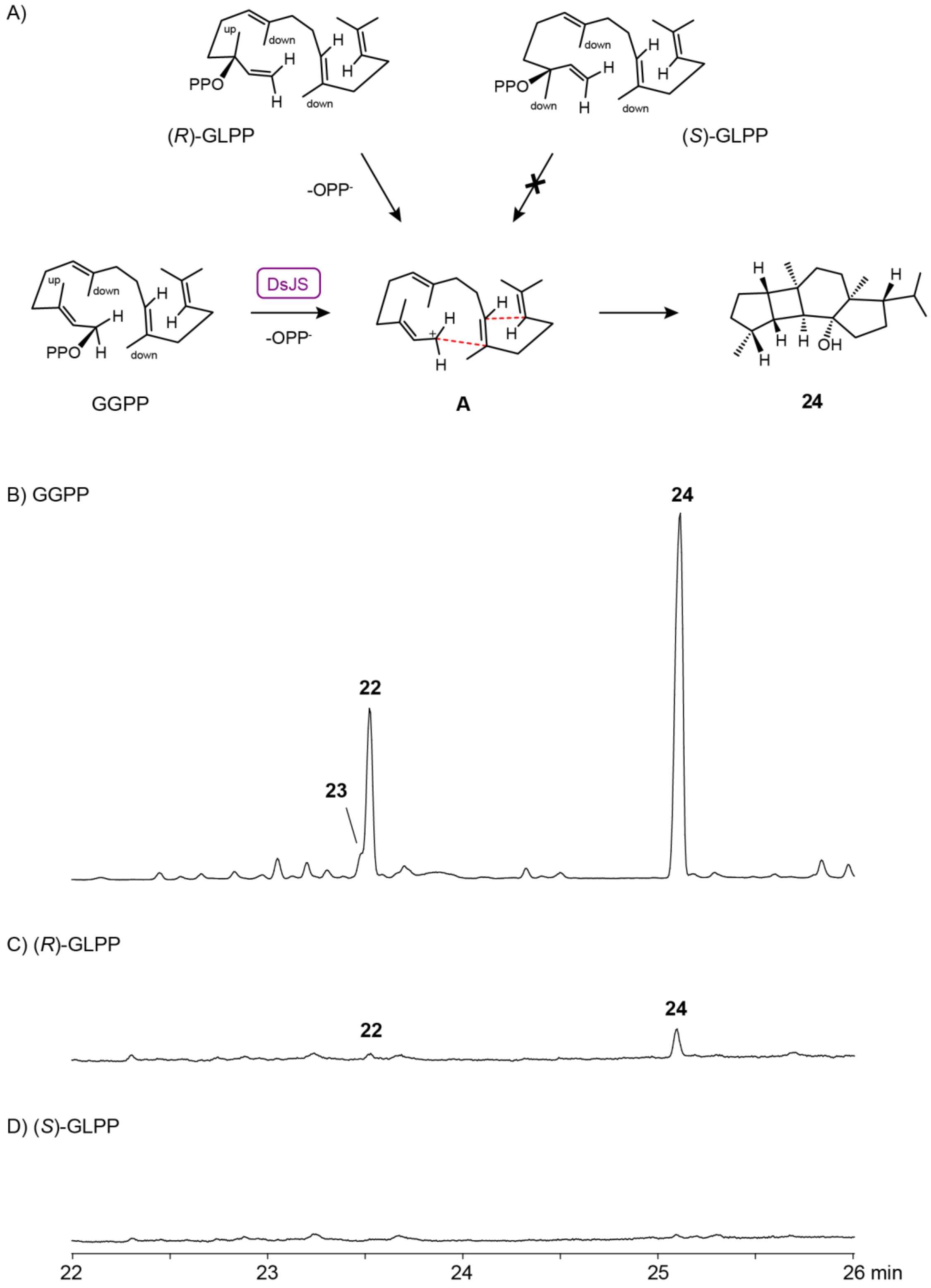

Figure S153. The conformational fold of GGPP in the biosynthesis of **24** by DsJS (Scheme 3 of main text). A) Proposed substrate conformations of GGPP, (*R*)- and (*S*)-GLPP in the biosynthesis of **24** by DsJS. GC/MS analysis of the crude extracts obtained from the incubation of B) GGPP, C) (*R*)-GLPP and D) (*S*)-GLPP with DsJS. Product formation from (*R*)-GLPP, but not from (*S*)-GLPP suggests a GGPP fold with Me20 pointing up.

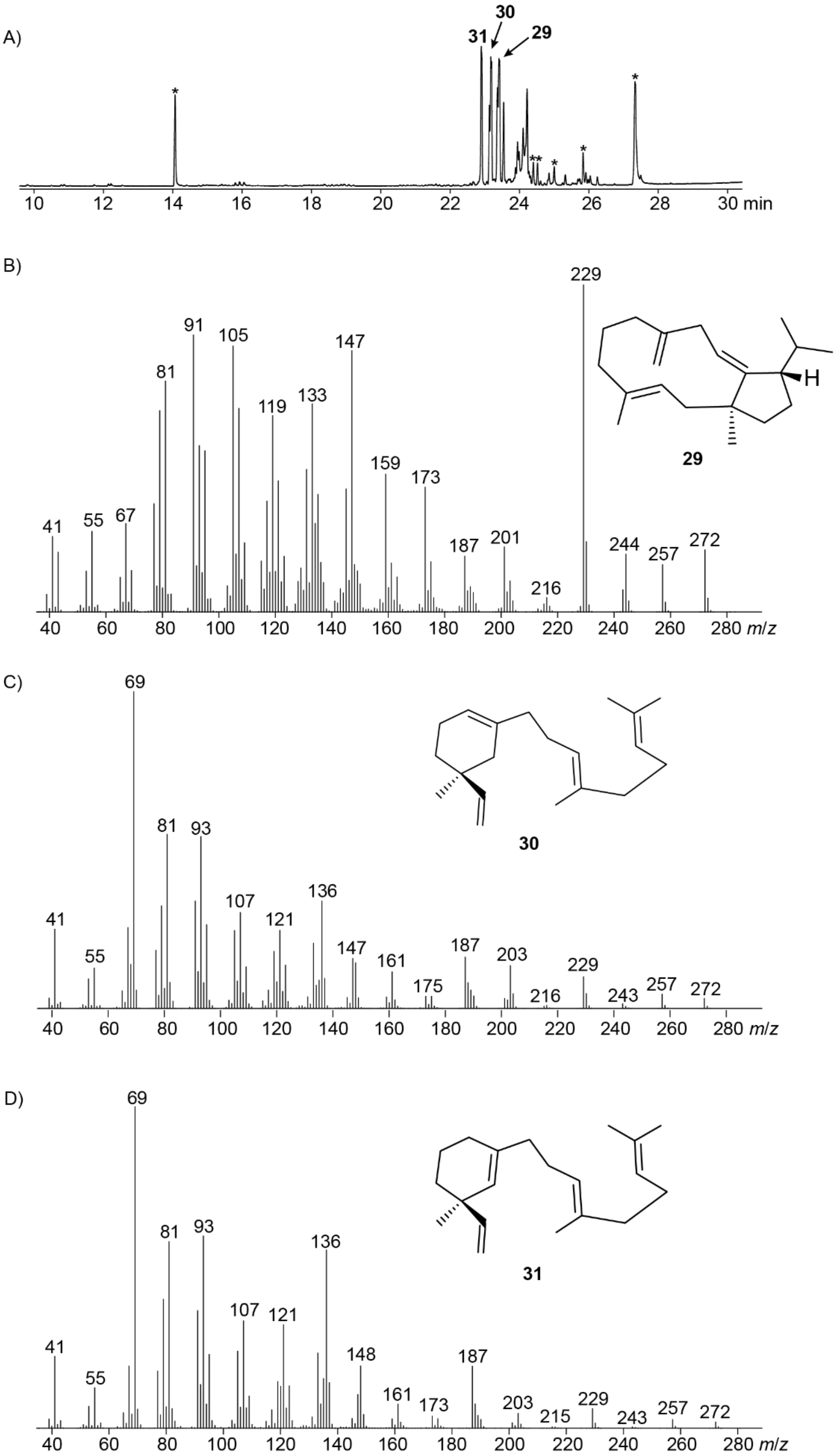

Figure S154. Enzymatic conversion of *iso*-GGPP I with CjNdtS. A) Total ion chromatogram of an extract from the incubation of *iso*-GGPP I with CjNdtS. EI mass spectrum of B) **29**, C) **30** and D) **31**. Asterisks indicate contaminants and spontaneous lysis and hydrolysis products of *iso*-GGPP I.

Conversion of *iso*-GGPP I with CjNdtS and product isolation

A solution of the trisammonium salt of *iso*-GGPP I (60 mg, 120 μmol) in substrate buffer (10 mL) was added to incubation buffer (100 mL) containing CjNtPS (40 mg, 0.31 mg L^{-1}), followed by incubation for 16 h at 30 °C. The reaction mixture was extracted with n-pentane (3x 100 mL), the combined organic extracts were dried with $MgSO_4$ and the solvent was evaporated, followed by compound isolation through column chromatography on silica gel to yield pure **29** (0.79 mg, 2.9 μmol, 2.4%) as a colourless oil. The fraction containing **30** and **31** was collected and evaporated to remove the solvent, followed by column chromatography on $AgNO_3$ impregnated silica gel. Pure **30** (0.40 mg, 1.5 μmol, 1.3%) and **31** (0.38 mg, 1.4 μmol, 1.2%) were obtained as colourless oil.

Dolabella-1,4(16),8-triene (29). TLC (pentane): R_f = 0.76. GC (HP-5MS): *I* = 1993. MS (EI, 70 eV): *m*/*z* (%) = 41 (6), 55 (8), 69 (7), 79 (10), 91 (11), 105 (9), 119 (7), 133 (5), 147 (5), 159 (4), 177 (3), 187 (4), 201 (2), 229 (13), 244 (0.4), 257 (2), 272 (3). IR (diamond ATR): ṽ = 3071 (w), 2953 (s), 2929 (s), 2868 (m), 1641 (w), 1468 (w), 1452 (w), 1397 (w), 1365 (m), 1092 (m), 1015 (s), 881 (w), 860 (w), 796 (s), 704 (w) cm^{-1}. HR-MS (Q-TOF, 70 eV): calc. $[C_{20}H_{32}]^+$ *m*/*z* = 272.2499; found: *m*/*z* = 272.2497. Optical rotation: $[\alpha]_D^{25}$ = –140 (*c* 0.08, CH_2Cl_2). NMR data are given in Table S21.

Taxasimplene (30). TLC (pentane/Et_2O = 8:1, on $AgNO_3$ impregnated TLC plates): R_f = 0.40. Optical rotation: $[\alpha]_D^{25}$ = +30.0 (*c* 0.04, CH_2Cl_2). NMR data were identical to previously reported data.[37]

Japosimplene (31). TLC (pentane/Et_2O = 8:1, on $AgNO_3$ impregnated TLC plates): R_f = 0.56. GC (HP-5MS): *I* = 1871. MS (EI, 70 eV): *m*/*z* (%) = 41 (4), 55 (3), 69 (26), 81 (15), 93 (15), 107 (7), 121 (7), 136 (11), 148 (4), 161 (1), 173 (1), 187 (3), 203 (1), 229 (1), 257 (0.4), 272 (0.3). IR (diamond ATR): ṽ = 2960 (w), 2924 (w), 2853 (w), 1738 (w), 1665 (w), 1450 (w), 1411 (w), 1376 (w), 1258 (s), 1085 (s), 1011 (s), 909 (w), 864 (w), 791 (s), 700 (w) cm^{-1}. HR-MS (Q-TOF, 70 eV): calc. $[C_{20}H_{32}]^+$ *m*/*z* = 272.2499; found: *m*/*z* = 272.2505. Optical rotation: $[\alpha]_D^{25}$ = –10.0 (*c* 0.03, CH_2Cl_2). NMR data are given in Table S22.

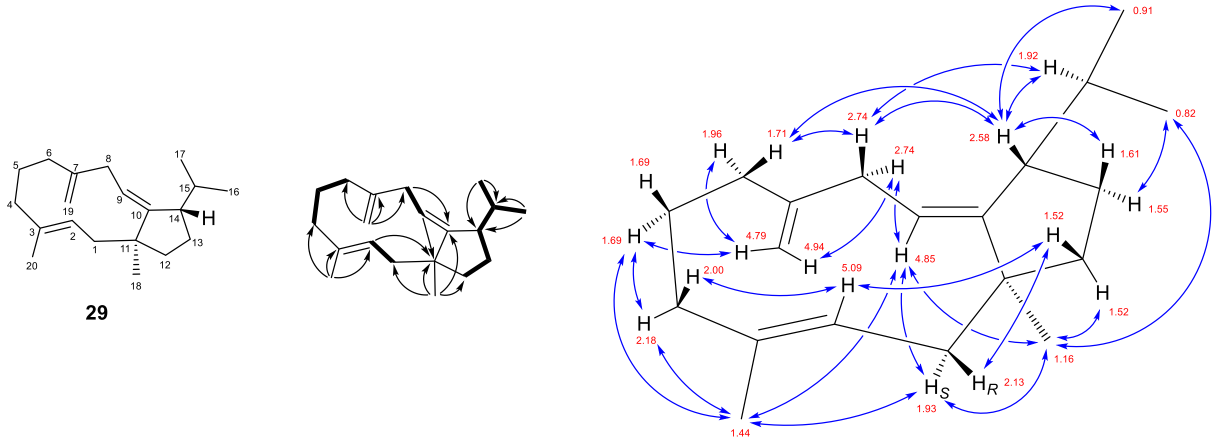

Figure S155. Structure elucidation of dolabella-1,4(16),8-triene (**29**). Bold: $^{1}H,^{1}H$-COSY, single headed arrows: key HMBC, and double headed arrows: NOESY correlations. Carbon numbering follows GGPP numbering to indicate the origin of each carbon. H_R and H_S indicate the results of stereoselective labelling experiments (Figure S171).

Table S21. NMR data of dolabella-1,4(16),8-triene (**29**) in C_6D_6 recorded at 298 K.

C[a]	type	^{13}C[b]	^{1}H[b]
1	CH_2	39.67	2.13 (dd, J = 8.6, 14.2)<br>1.93 (m)
2	CH	126.62	5.09 (t, J = 7.4)
3	C_q	133.17	–
4	CH_2	38.91	2.18 (m)<br>2.00 (m)
5	CH_2	23.93	1.69 (m, 2H)
6	CH_2	29.59	1.96 (m)<br>1.71 (m)
7	C_q	149.91	–
8	CH_2	40.97	2.74 (m, 2H)
9	CH	123.50	4.85 (ddd, J = 11.0, 4.5, 2.4)
10	C_q	149.91	–
11	C_q	47.29	–
12	CH_2	40.51	1.52 (m, 2H)
13	CH_2	24.00	1.61 (m)<br>1.55 (m)
14	CH	47.66	2.58 (m)
15	CH	32.09	1.92 (m)
16	CH_3	22.02	0.91 (d, J = 6.8)
17	CH_3	18.23	0.82 (d, J = 6.8)
18	CH_3	27.44	1.16 (s)
19	CH_2	108.61	4.94 (m)<br>4.79 (m)
20	CH_3	15.32	1.44 (br s)

[a] Carbon numbering as shown in Figure S155. [b] Chemical shifts δ in ppm, multiplicity: s = singlet, d = doublet, m = multiplet, t = triplet, br = broad, coupling constants J are given in Hertz.

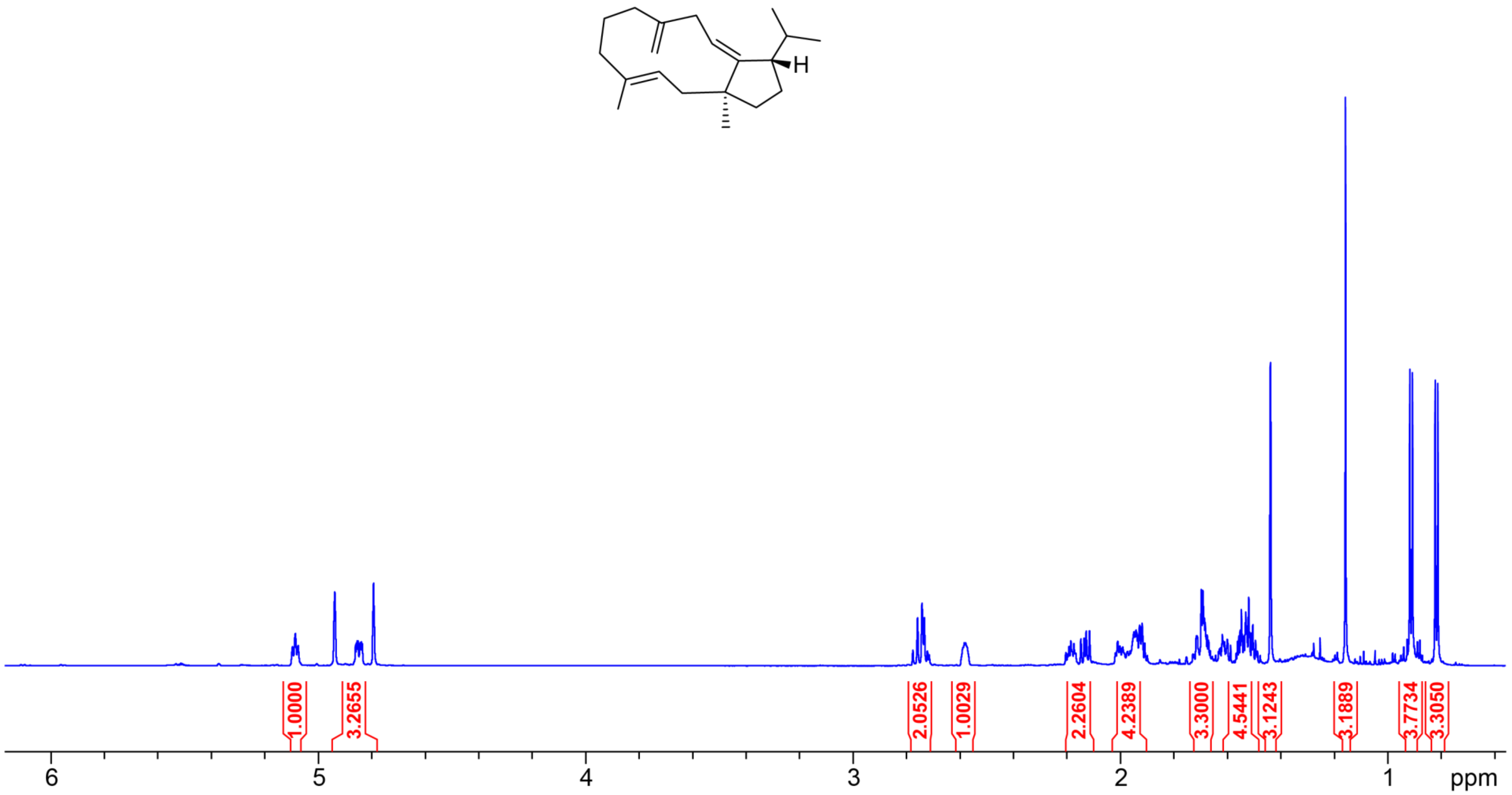

Figure S156. ^{1}H-NMR spectrum of **29** (700 MHz, C_6D_6).

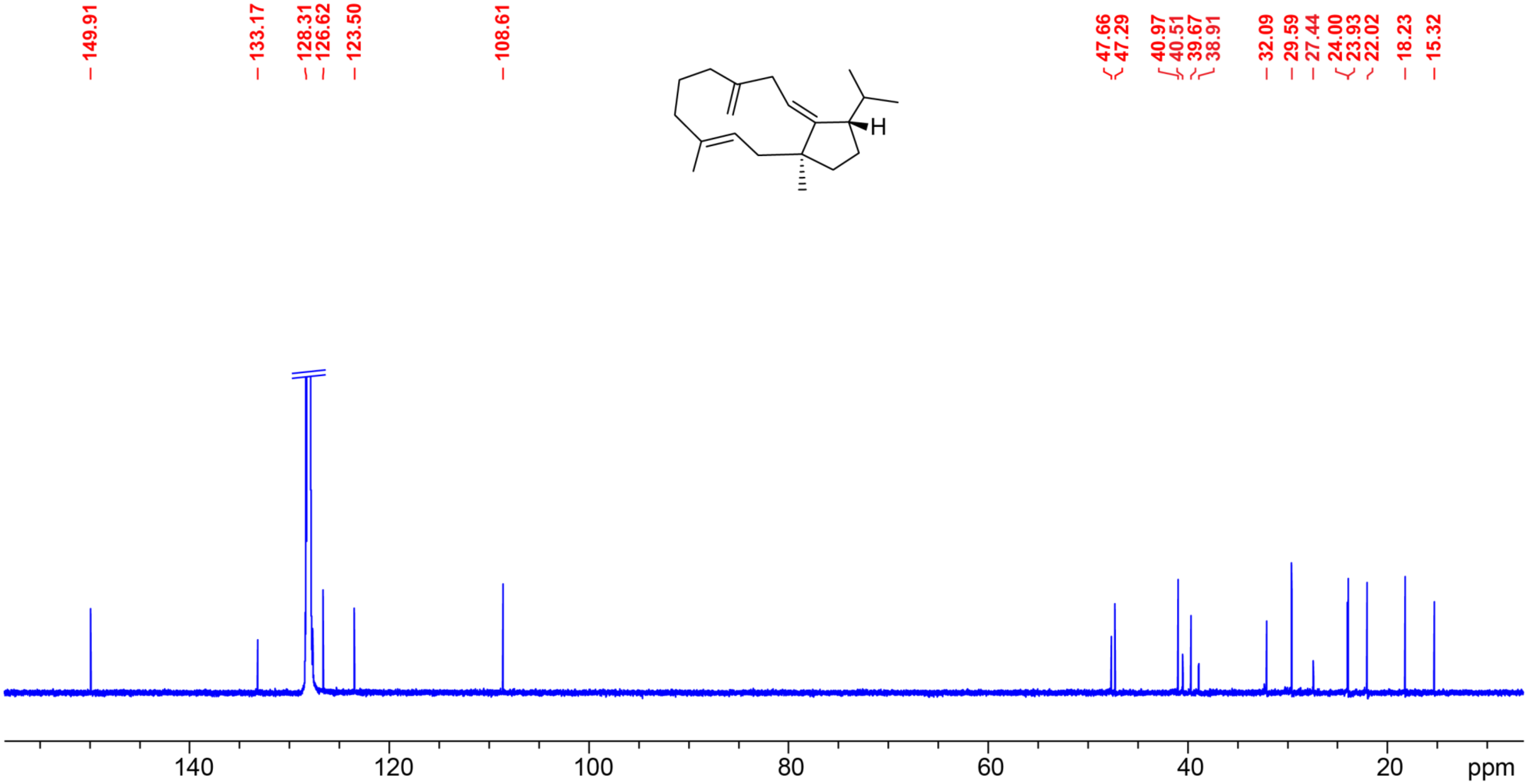

Figure S157. ^{13}C-NMR spectrum of **29** (176 MHz, C_6D_6).

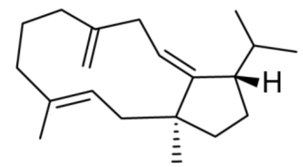

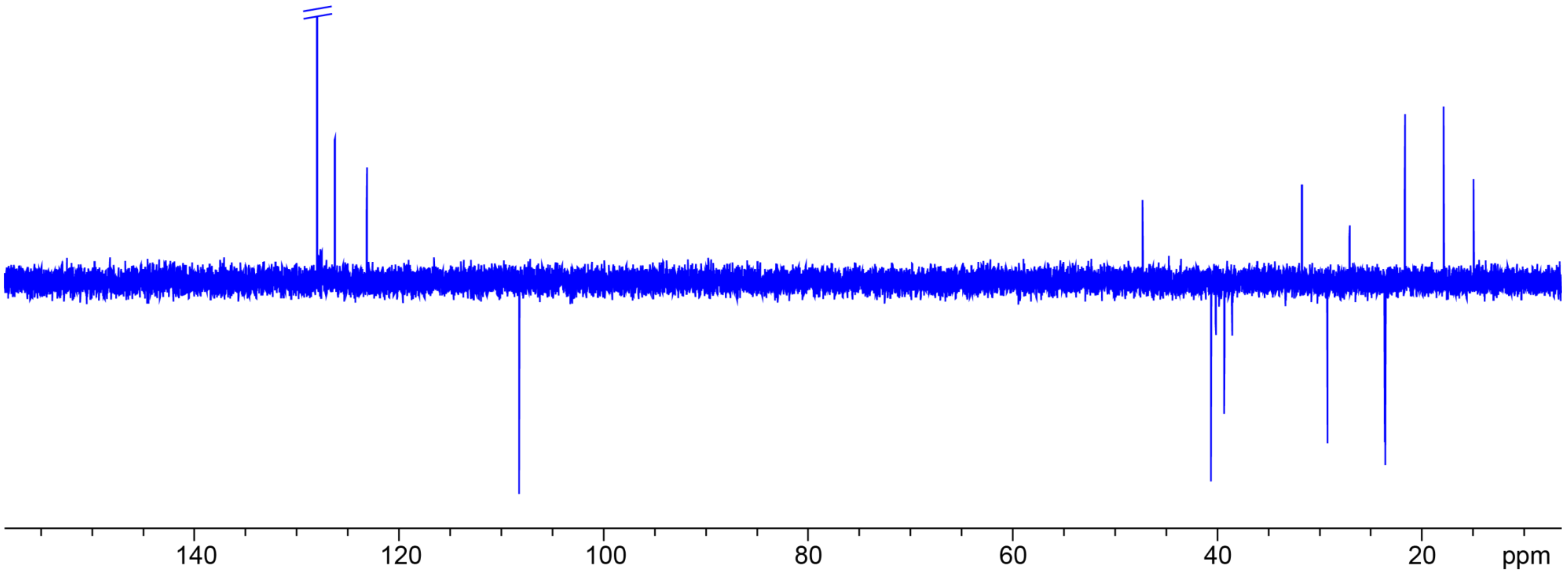

Figure S158. ^{13}C-DEPT135 spectrum of **29** (176 MHz, C_6D_6).

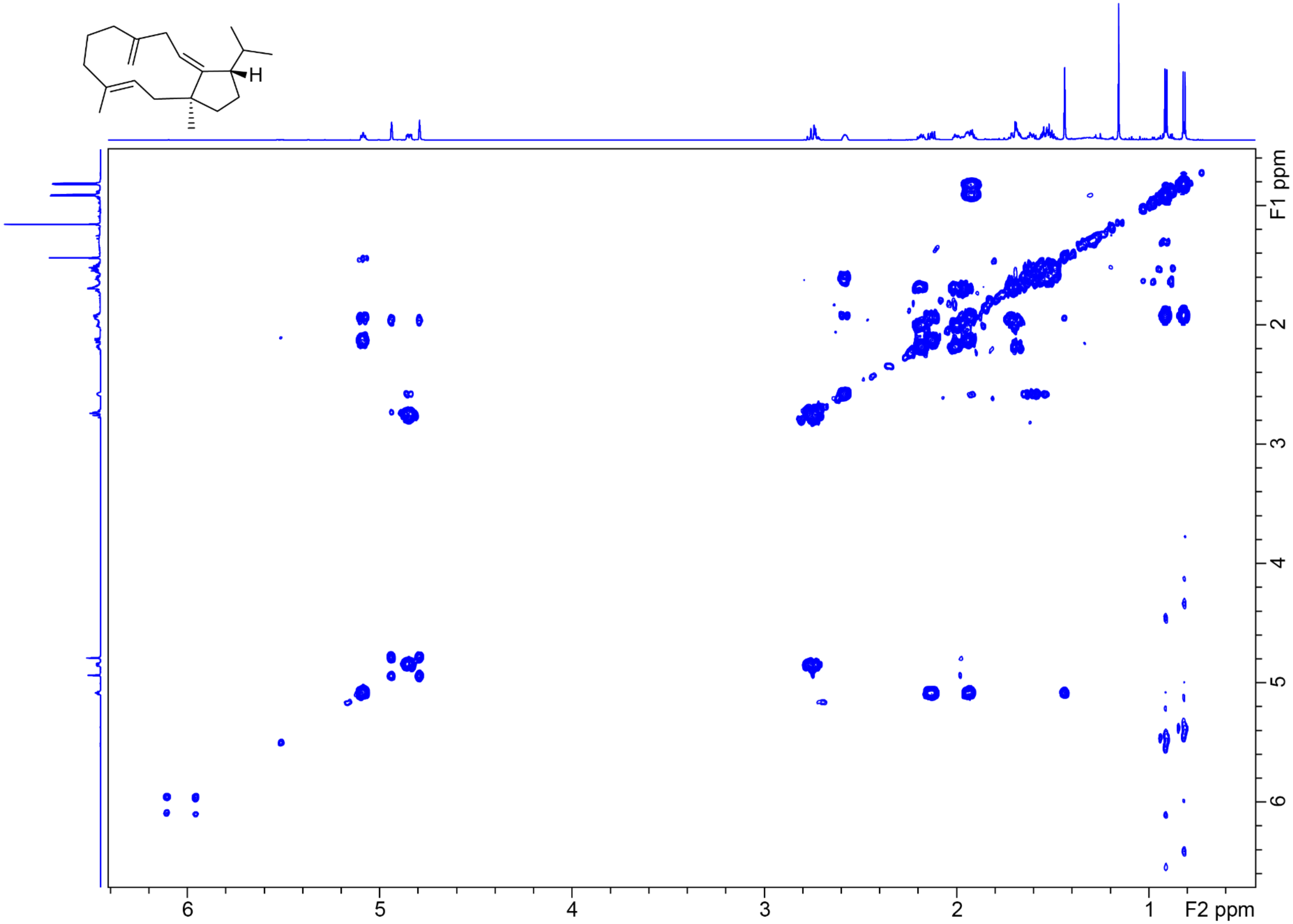

Figure S159. ^{1}H-^{1}H-COSY spectrum (C_6D_6) of **29**.

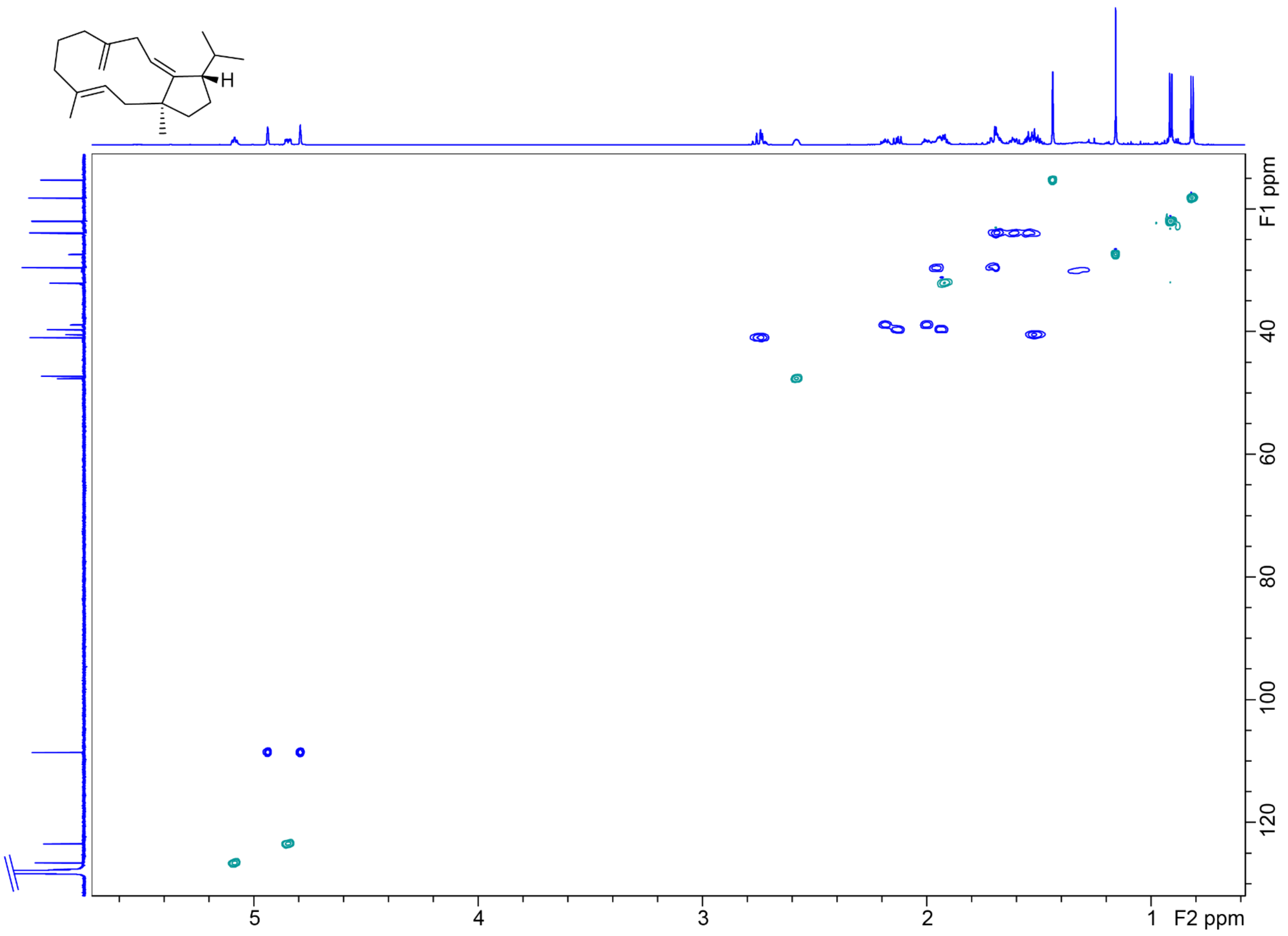

Figure S160. HSQC spectrum (C_6D_6) of **29**.

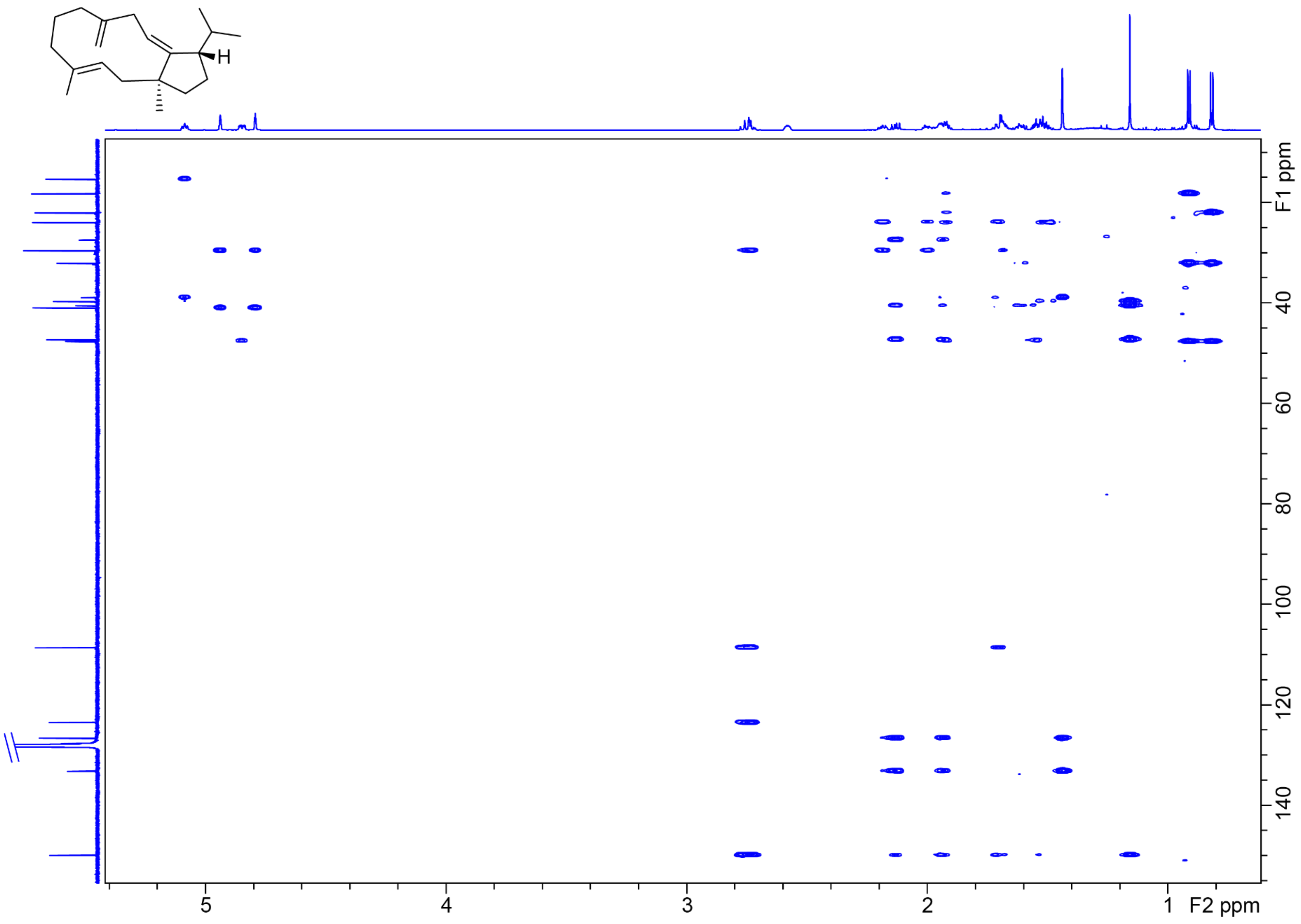

Figure S161. HMBC spectrum (C_6D_6) of **29**.

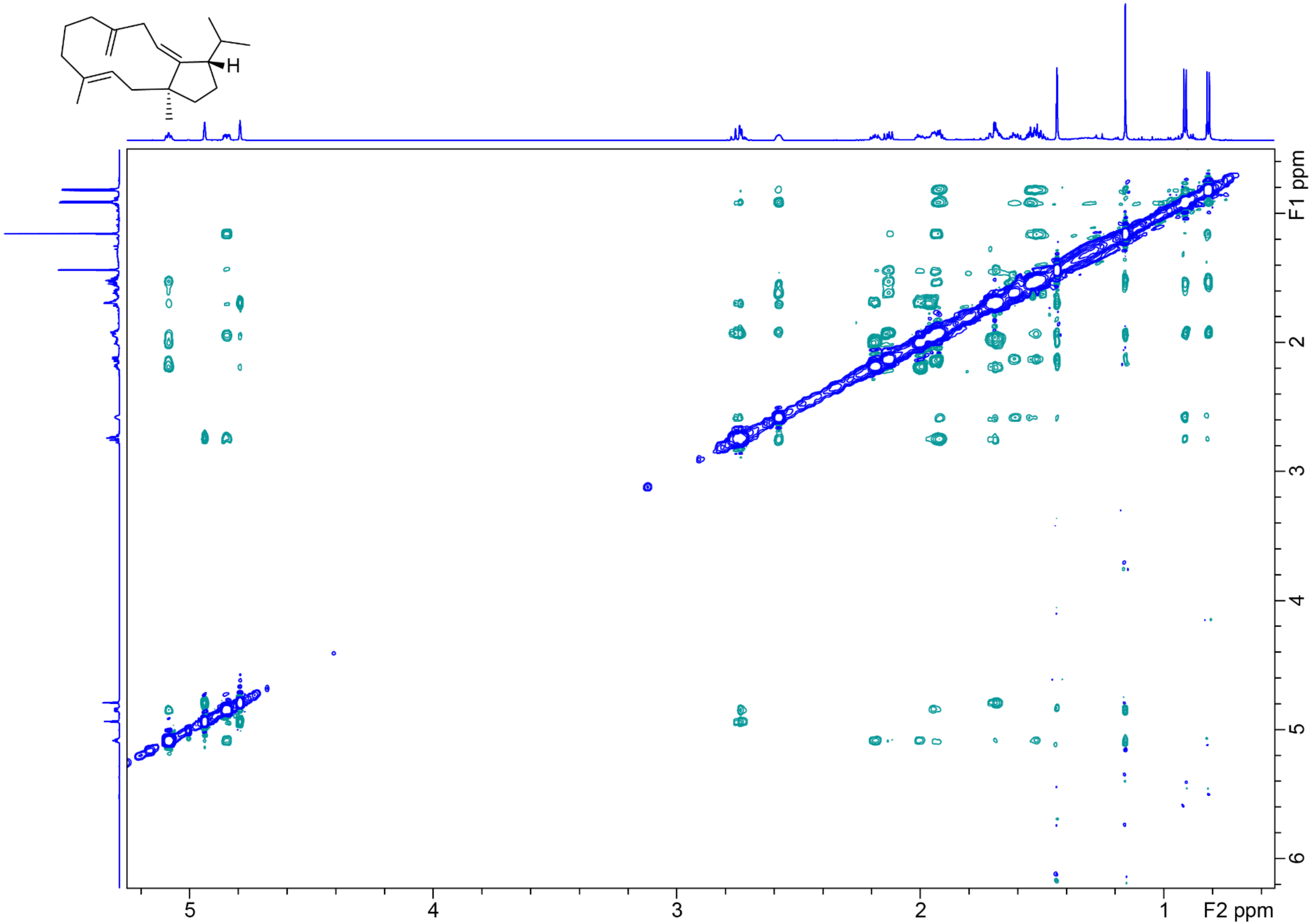

Figure S162. NOESY spectrum (C_6D_6) of **29**.

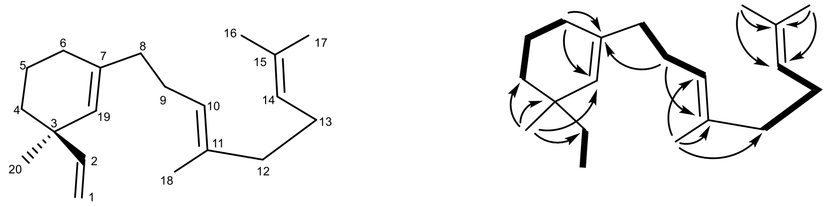

31

Figure S163. Structure elucidation of japosimplene (**31**). Bold: ^{1}H,^{1}H-COSY, and single headed arrows: key HMBC correlations. Carbon numbering follows GGPP numbering to indicate the origin of each carbon.

Table S22. NMR data of japosimplene (**31**) in C_6D_6 recorded at 298 K.

C[a]	type	^{13}C[b]	^{1}H[b]
1	CH_2	112.11	5.04 (m, 2H)
2	CH	148.13	5.80 (dd, *J* = 10.6, 16.6)
3	C_q	38.72	–
4	CH_2	36.08	1.54 (m)
			1.35 (m)
5	CH_2	19.80	1.60 (m)
			1.54 (m)
6	CH_2	28.71	1.82 (m, 2H)
7	C_q	137.60	–
8	CH_2	38.33	2.05 (m, 2H)
9	CH_2	26.89	2.18 (m, 2H)
10	CH	124.79	5.28 (m)
11	C_q	135.08	
12	CH_2	40.25	2.10 (m, 2H)
13	CH_2	27.26	2.20 (m, 2H)
14	CH	124.99	5.25 (m)
15	C_q	131.17	–
16	CH_3	17.77	1.57 (s)
17	CH_3	25.89	1.68 (s)
18	CH_3	16.20	1.59 (s)
19	CH	128.46	5.26 (m)
20	CH_3	28.73	1.10 (s)

[a] Carbon numbering as shown in Figure S163 indicates the origin of each carbon from GGPP by same number. [b] Chemical shifts δ in ppm, multiplicity: s = singlet, d = doublet, m = multiplet, coupling constants *J* are given in Hertz.

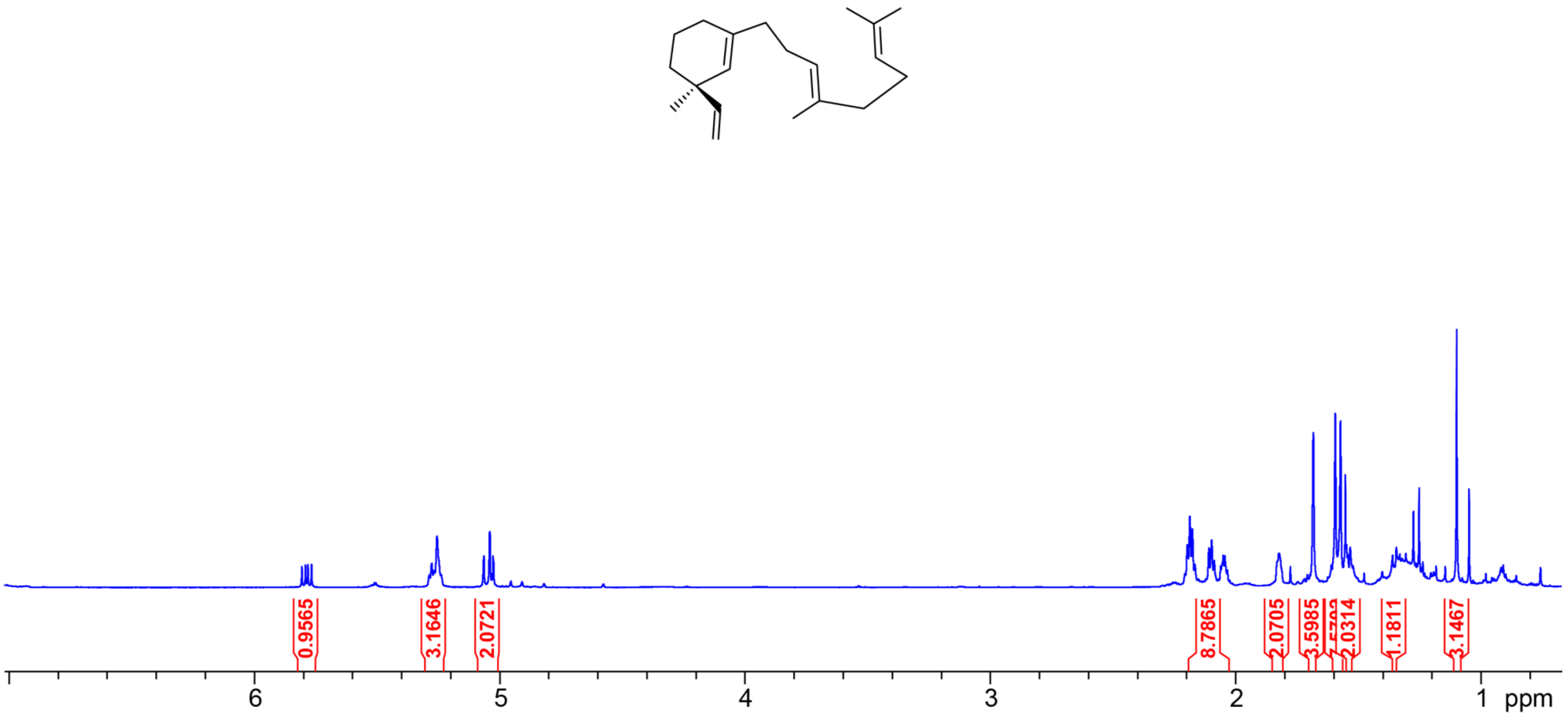

Figure S164. ^{1}H-NMR spectrum of **31** (700 MHz, C_6D_6).

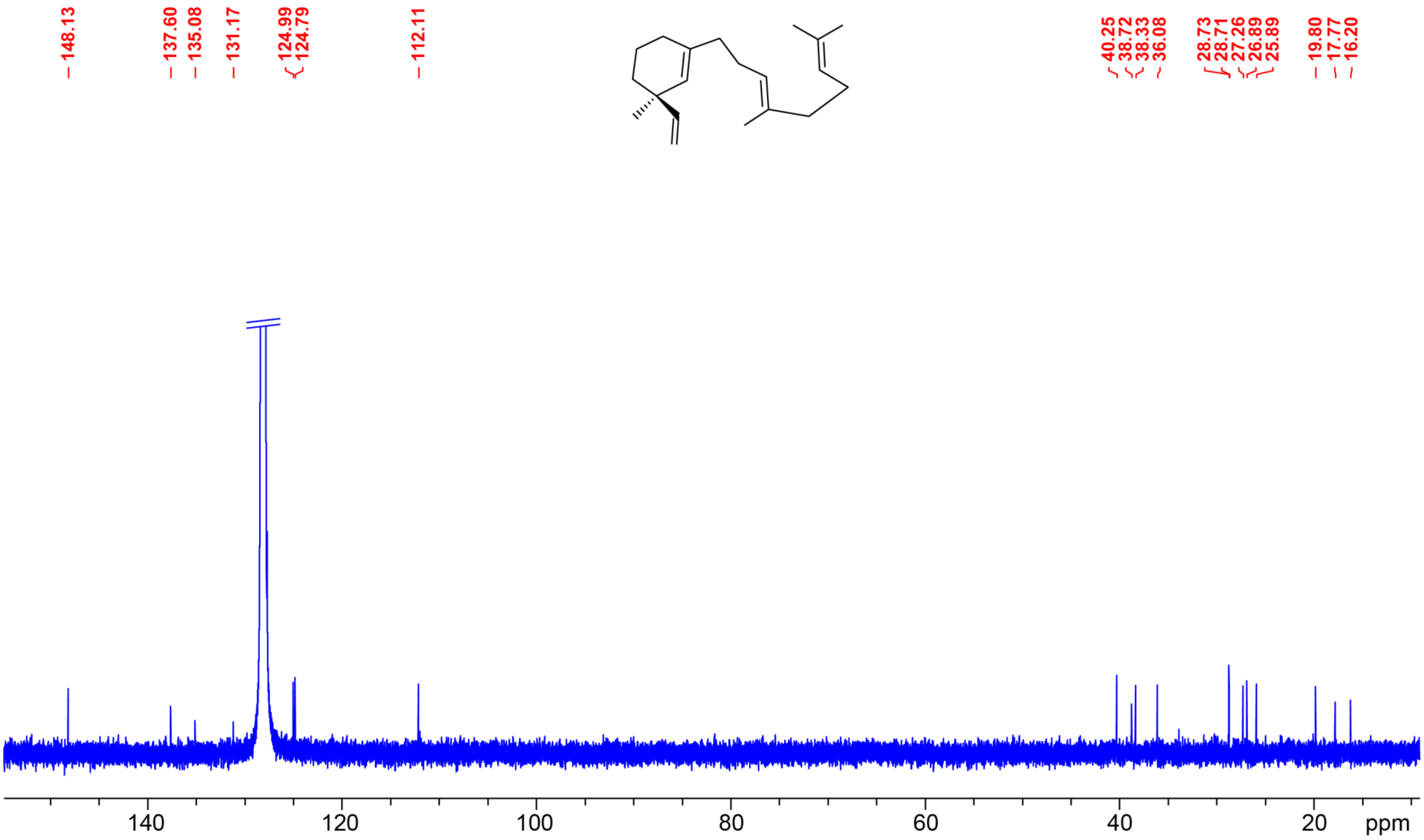

Figure S165. ^{13}C-NMR spectrum of **31** (176 MHz, C_6D_6).

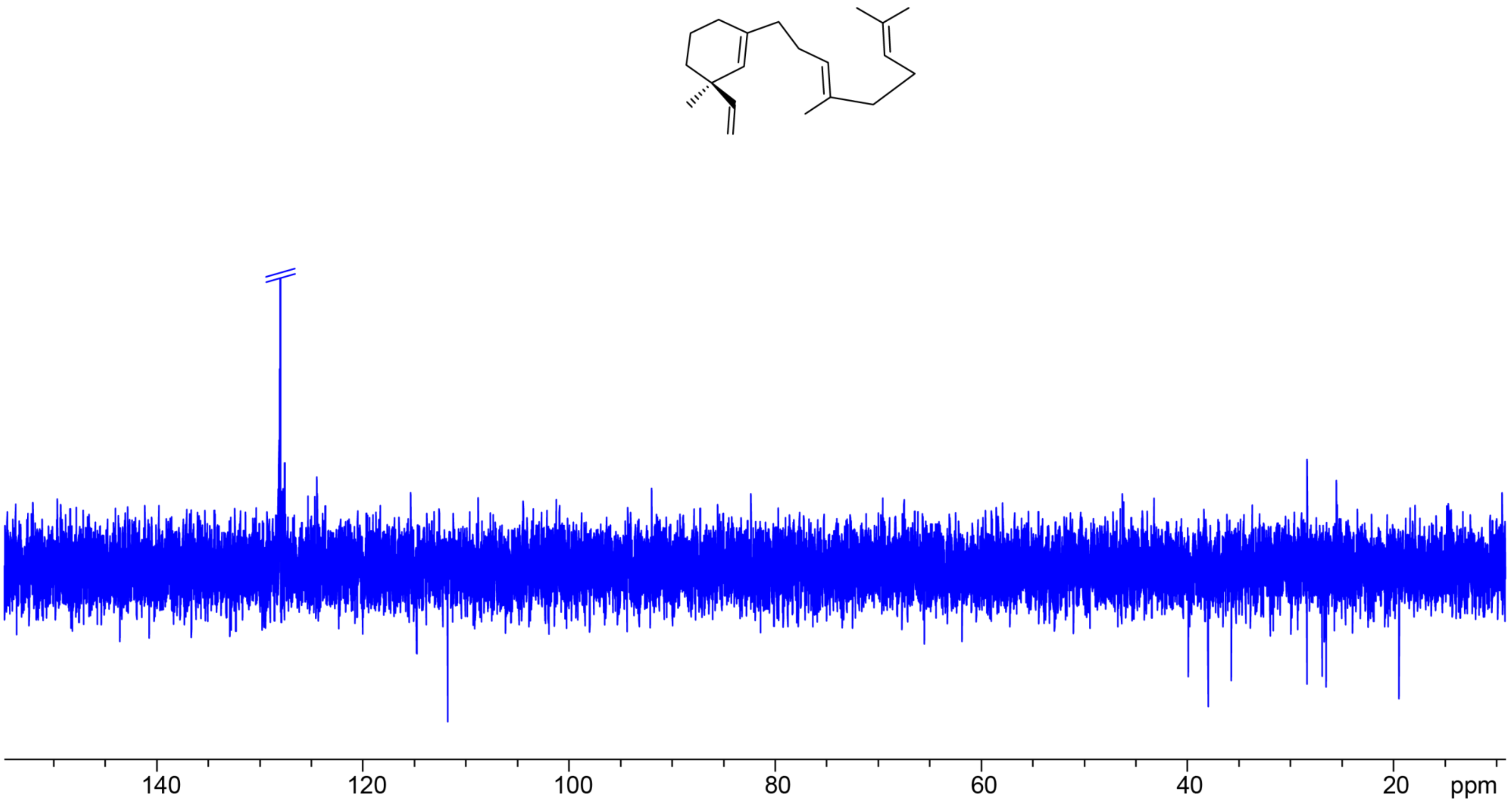

Figure S166. ^{13}C-DEPT135 spectrum of **31** (176 MHz, C_6D_6).

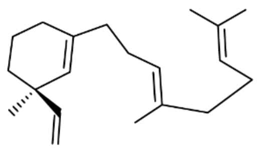

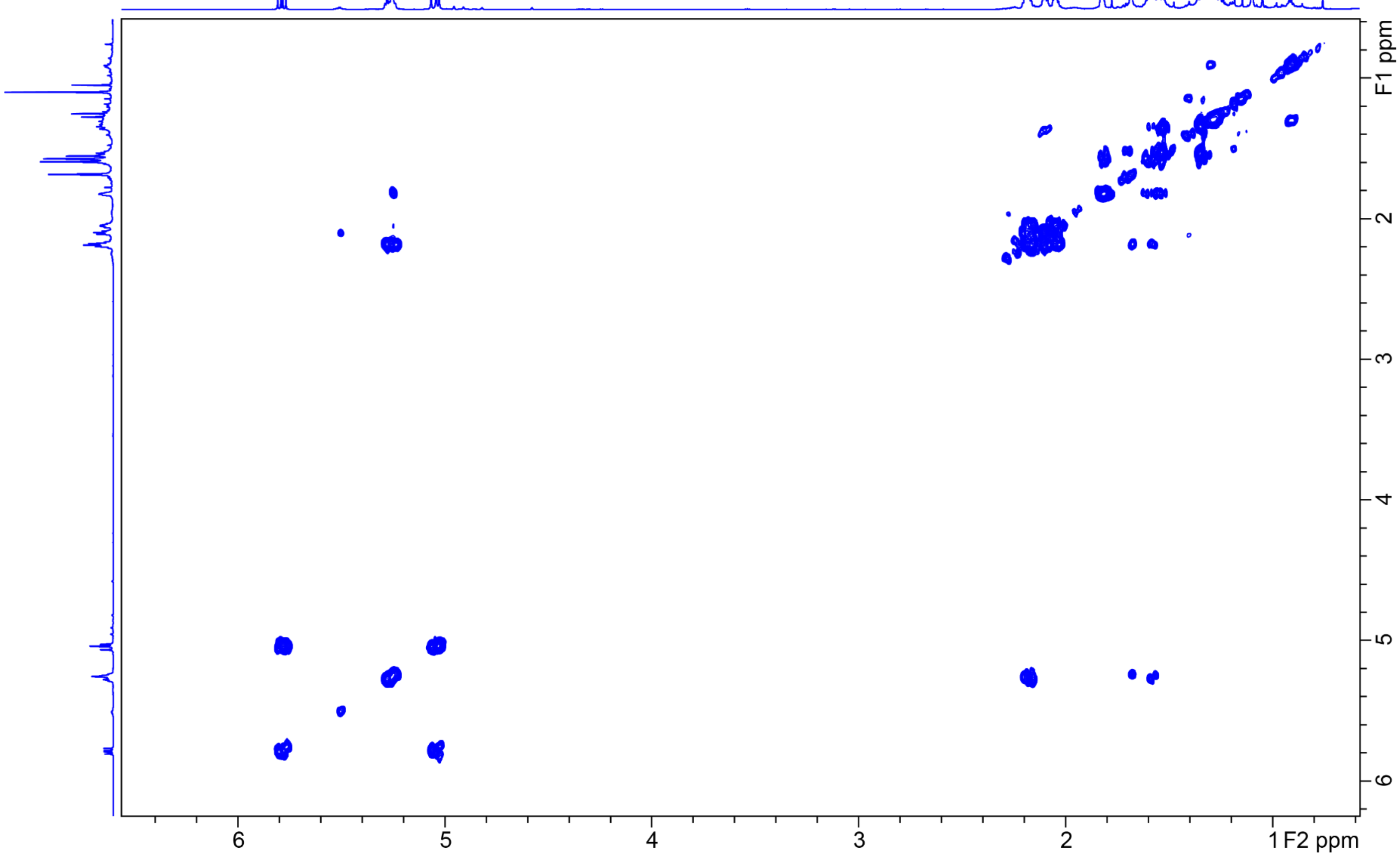

Figure S167. ^{1}H-^{1}H-COSY spectrum (C_6D_6) of **31**.

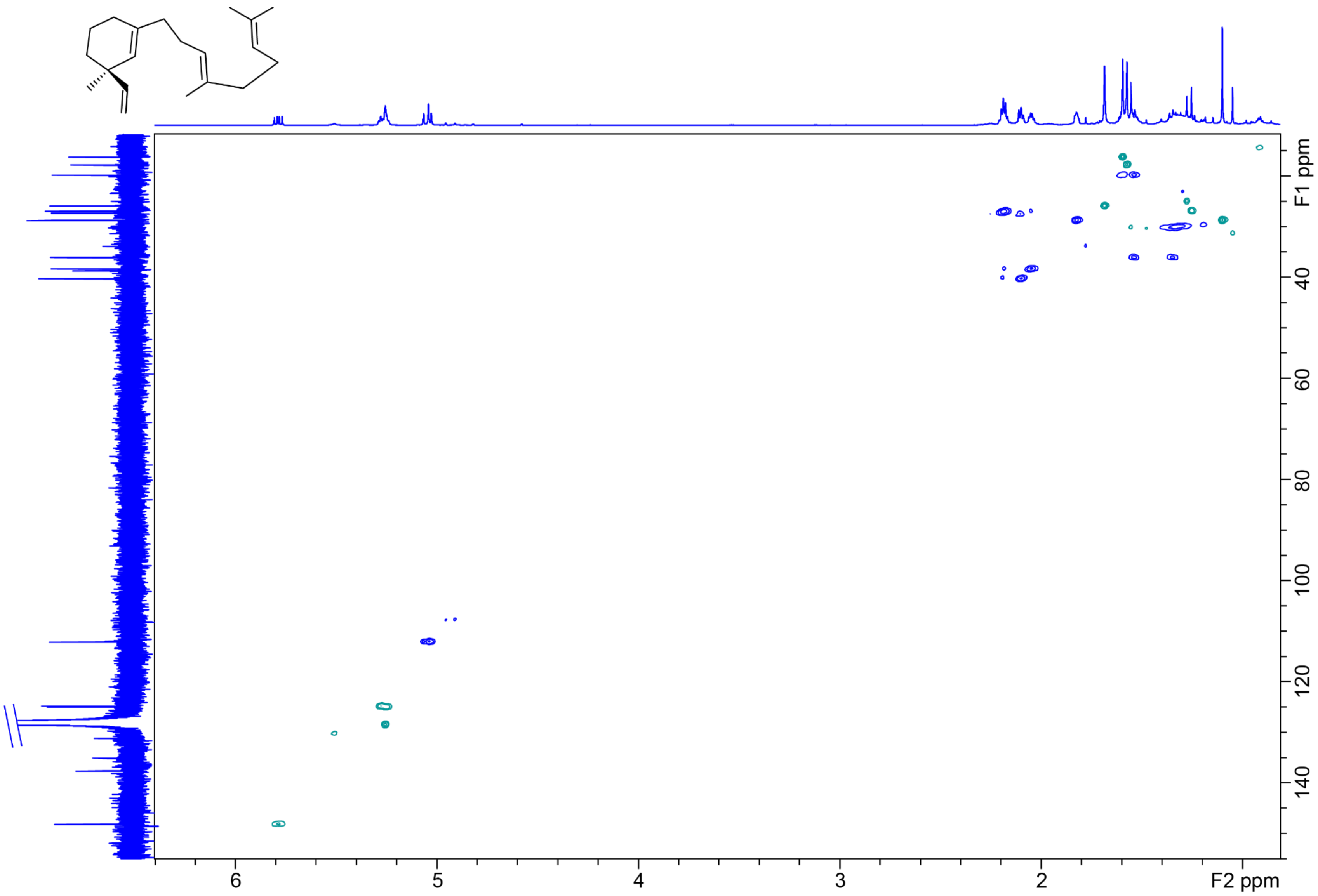

Figure S168. HSQC spectrum (C_6D_6) of **31**.

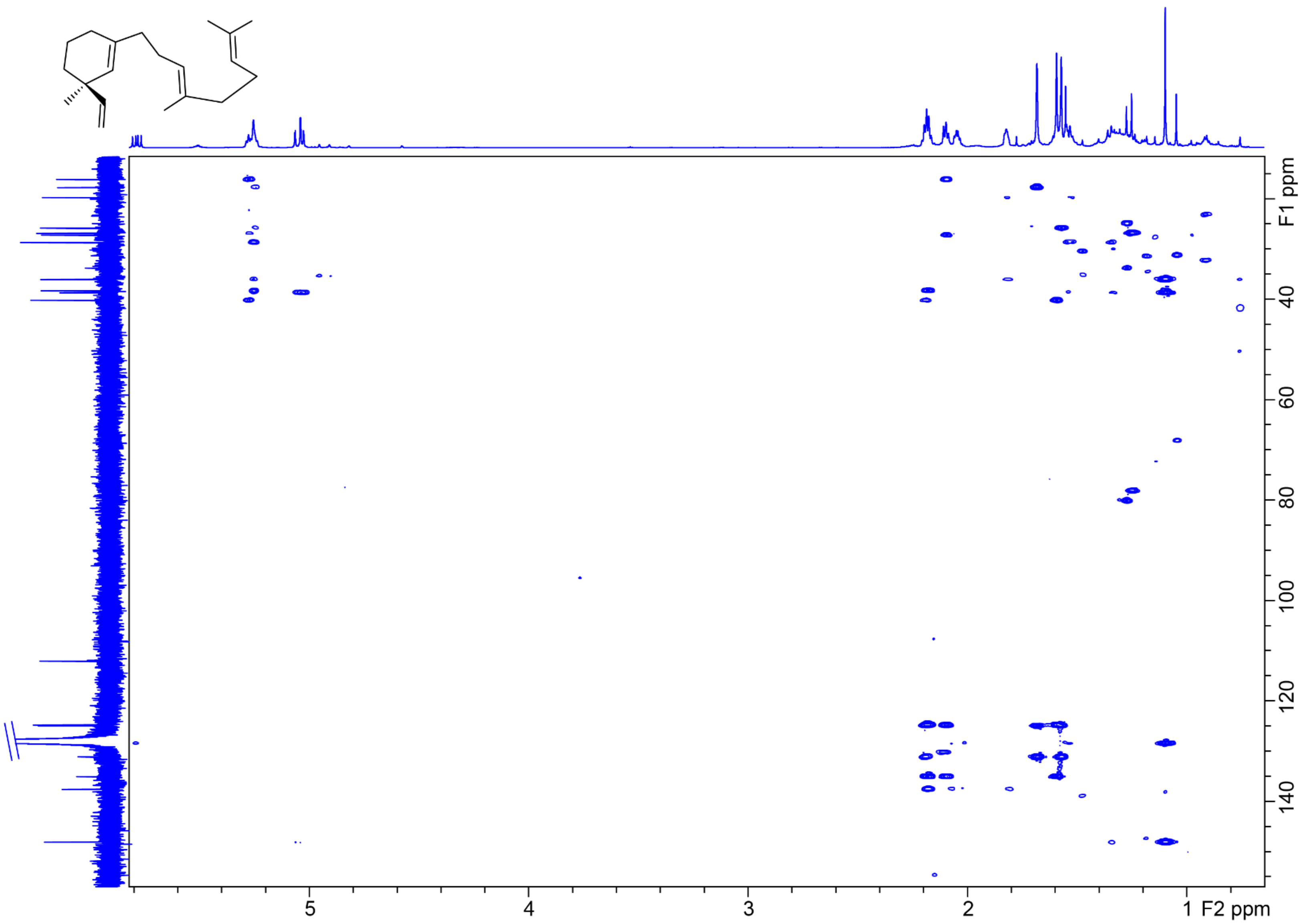

Figure S169. HMBC spectrum (C_6D_6) of **31**.

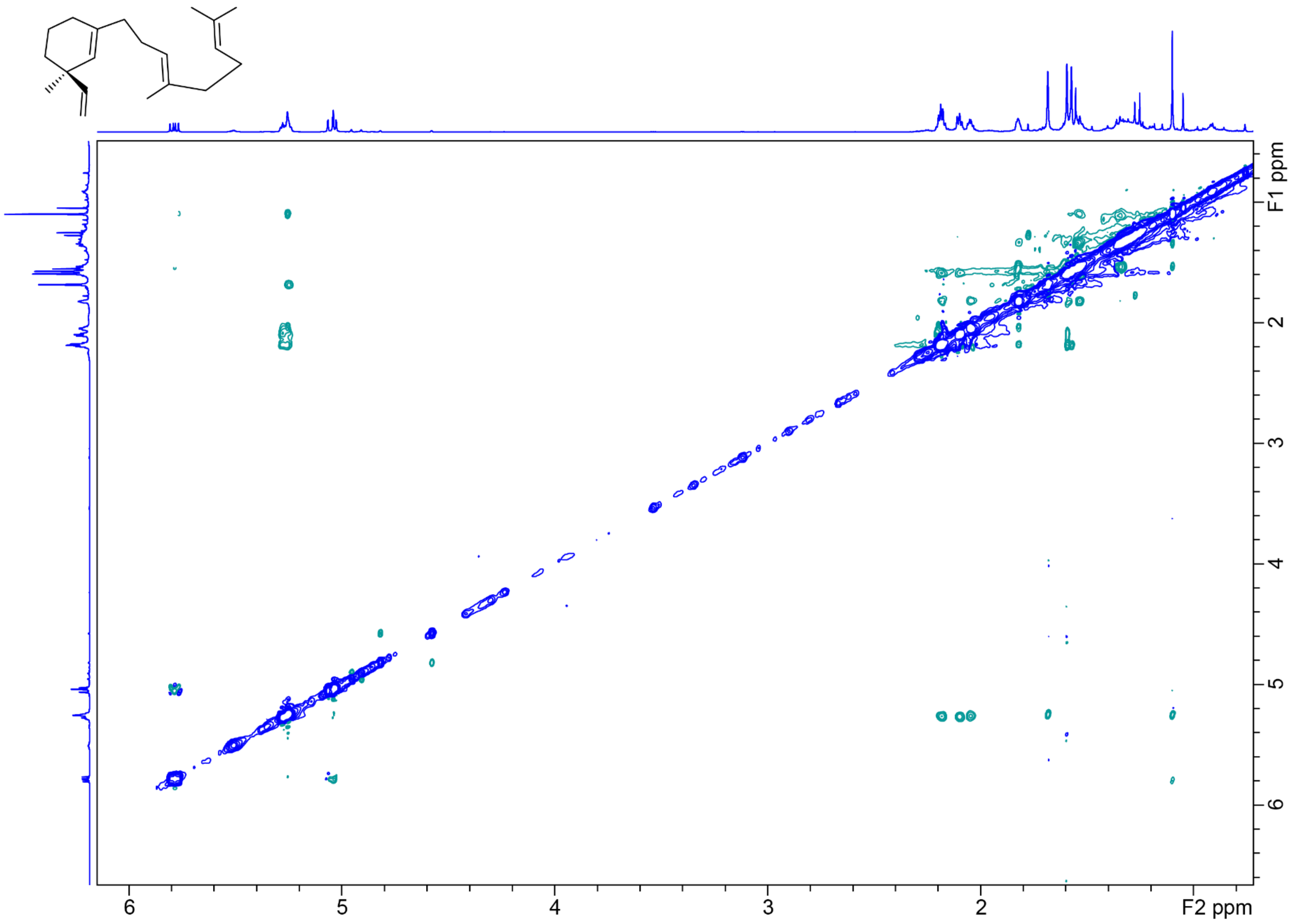

Figure S170. NOESY spectrum (C_6D_6) of **31**.

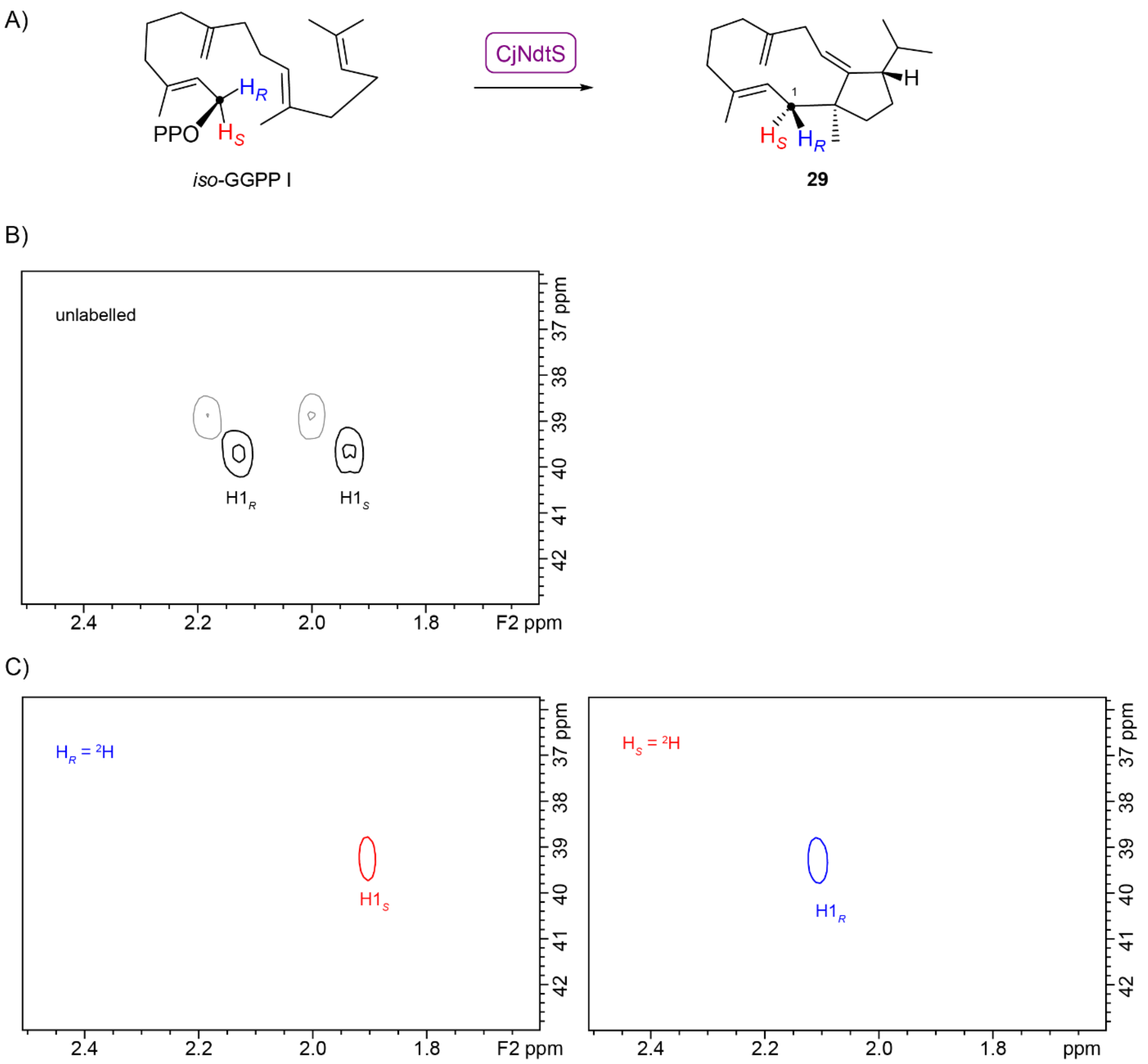

Figure S171. The absolute configuration of **29**. A) Cyclisation of (*R*)- (blue H = ^{2}H) and (*S*)-(1-^{13}C,1-^{2}H)-*iso*-GGPP (red H = ^{2}H) with CjNdtS. B) Partial HSQC spectra of unlabelled **29** showing the region for C1. C) Partial HSQC spectra of labelled **29** obtained from (*R*)-(1-^{13}C,1-^{2}H)-*iso*-GGPP (left) and from (*S*)-(1-^{13}C,1-^{2}H)-*iso*-GGPP (right). Together with the NOESY-based assignments (Figure S155), these data point to the shown absolute configuration of **29**. Black dots represent ^{13}C-labelled carbons.

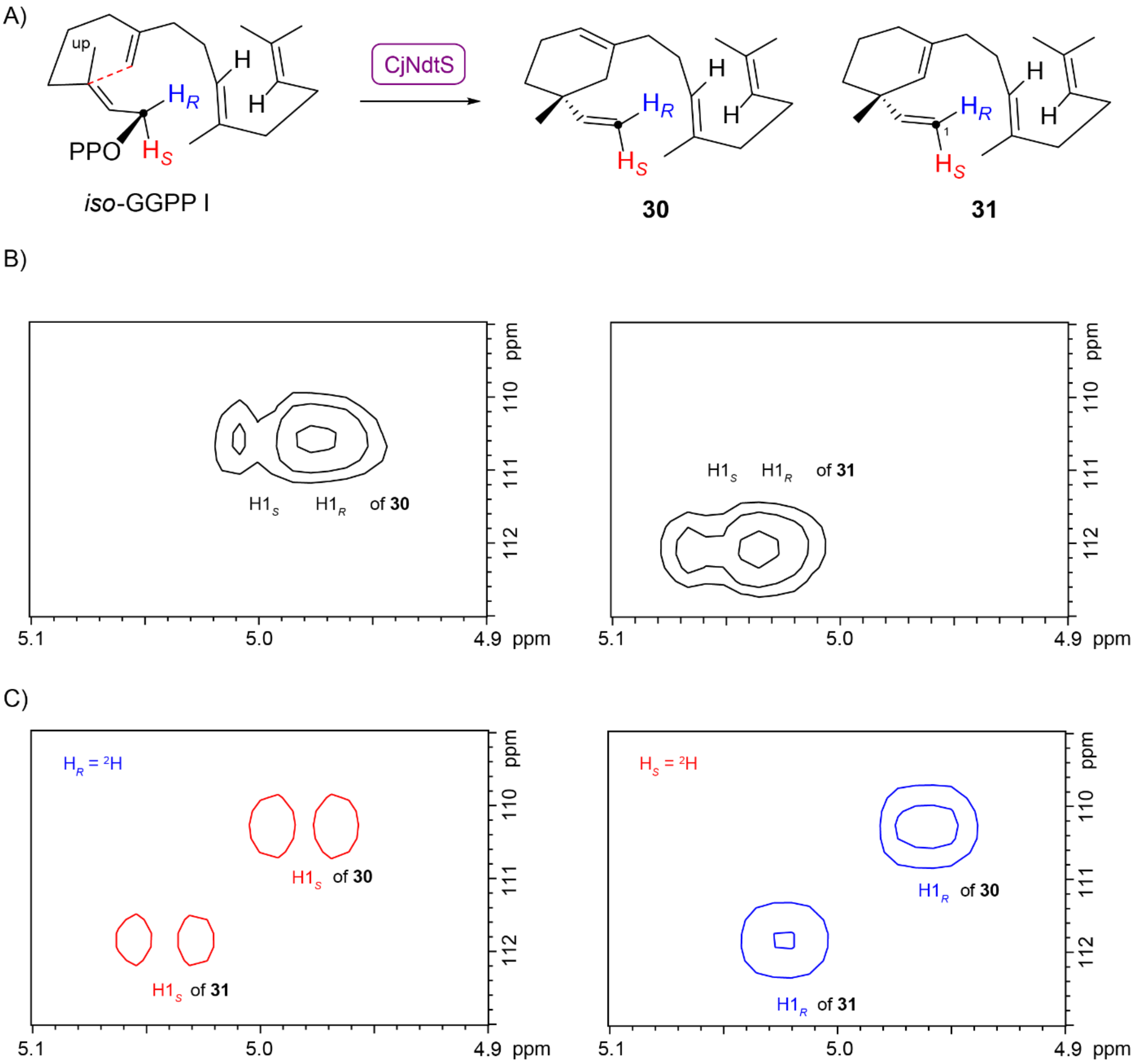

Figure S172. The stereochemical course for the formation of **30** and **31** from *iso*-GGPP I by CjNdtS. A) Conversion of *iso*-GGPP I into **30** and **31** by CjNdtS. Partial HSQC spectra showing the region for C1 of B) unlabelled **30** (left) and unlabelled **31** (right), and C) the crude enzyme products containing labelled **30** and **31** obtained from (*R*)-(1-^{13}C,1-^{2}H)GGPP (left) and from (*S*)-(1-^{13}C,1-^{2}H)GGPP (right) with CjNdtS. The observed stereochemical course with incorporation of $H1_R$ into the 1*Z* position and of $H1_S$ into the 1*E* position of the vinyl groups of **30** and **31** are in agreement with the model for the conformational fold of *iso*-GGPP I. Black dots represent ^{13}C-labelled carbons.

Table S23. Results of DFT calculations (wB97M-V/Def2-TZVPPD//B97D3/6-31G(d,p)) for the cyclisation cascade from GGPP to **21** (Scheme 3 of main text).

Structure[a]	Gibbs energy (298.15K) in Hartree	free energy relative to N / kcal/mol	free energy of activation / kcal/mol	free reaction energy / kcal/mol
**Cyclisation cascade from GGPP to 21**				
**N**	–781.127266	0.0		
**N-O-TS**	–781.123651	2.3	2.3	
**O**	–781.161420	–21.4		–21.4
**O**	–781.161423	–21.4		
**O-P-TS**	–781.150348	–14.5	6.9	
**P**	–781.169967	–26.8		–5.4
**P**	–781.169969	–26.8		
**P-Q-TS**	–781.164621	–23.4	3.4	
**Q**	–781.166329	–24.5		2.3
**Q**	–781.166337	–24.5		
**Q-R-TS**	–781.155484	–17.7	6.8	
**R**	–781.160182	–20.7		3.9
**R**	–781.147798[a]	–12.9		
**R-S-TS**	–781.142917[a]	–9.8	3.1	
**S**	–781.148505[a]	–13.3		–0.4
**S**	–781.148507[a]	–13.3		
**S-S-TS**	–781.117350[a]	6.2	19.6	
**S**	–781.162997[a]	–22.4		–9.1
**S**	–781.162244[a]	–22.0		
**S-T-TS**	–781.159458[a]	–20.2	1.7	
**T**	–781.171327[a]	–27.7		–5.7

[a] Steps were calculated with NH_3 as a surrogate base. For direct comparison, the Gibbs energies given here are the calculated energies with the Gibbs energy of NH_3 (56.537076 H) subtracted.

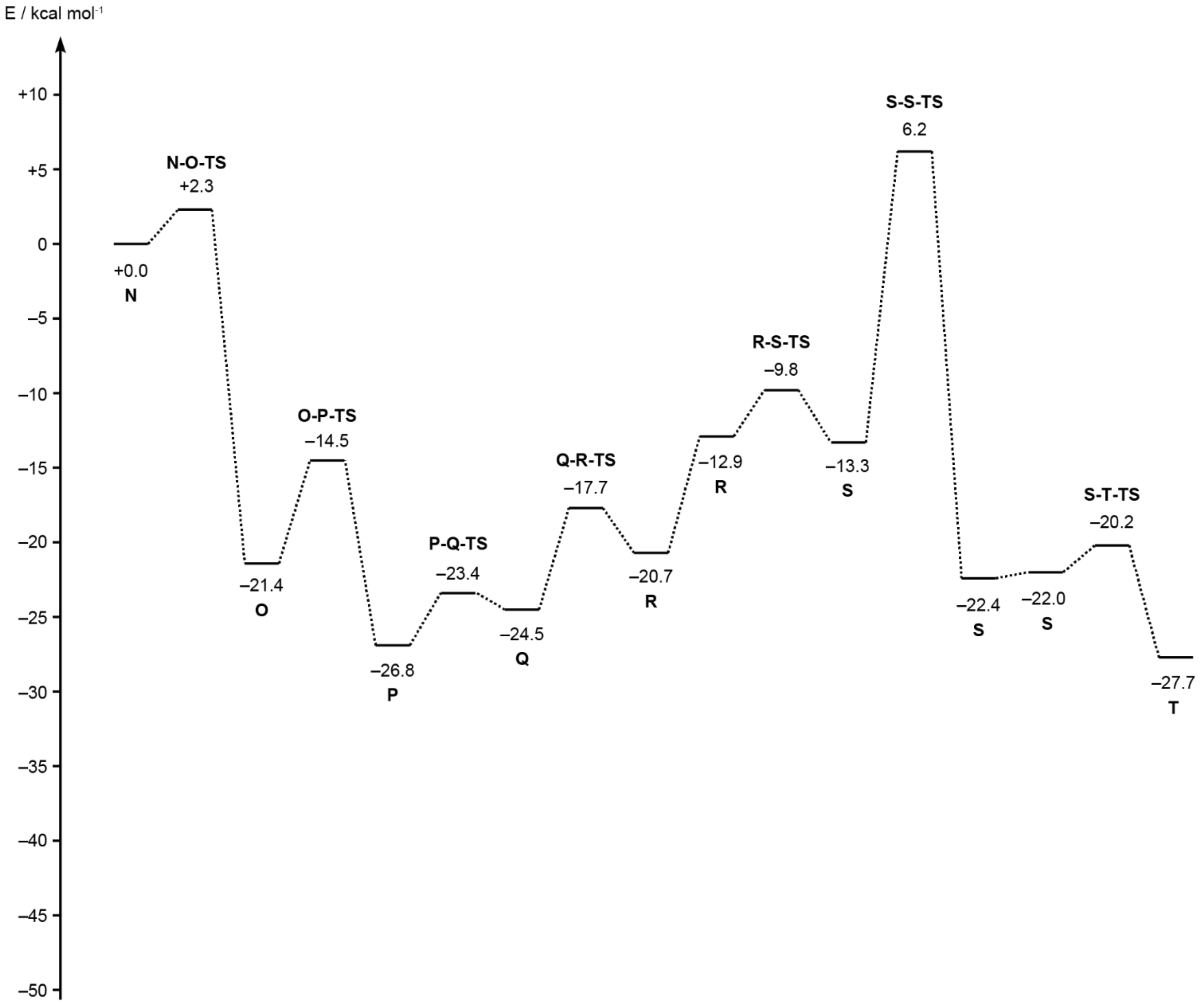

Figure S173. Computed energy profiles for the cyclisation cascade from GGPP to **21** (Scheme 3 of main text, mPW1PW91/6-311+G(d,p)//B97D3/6-31g(d,p), 298 K).

A)

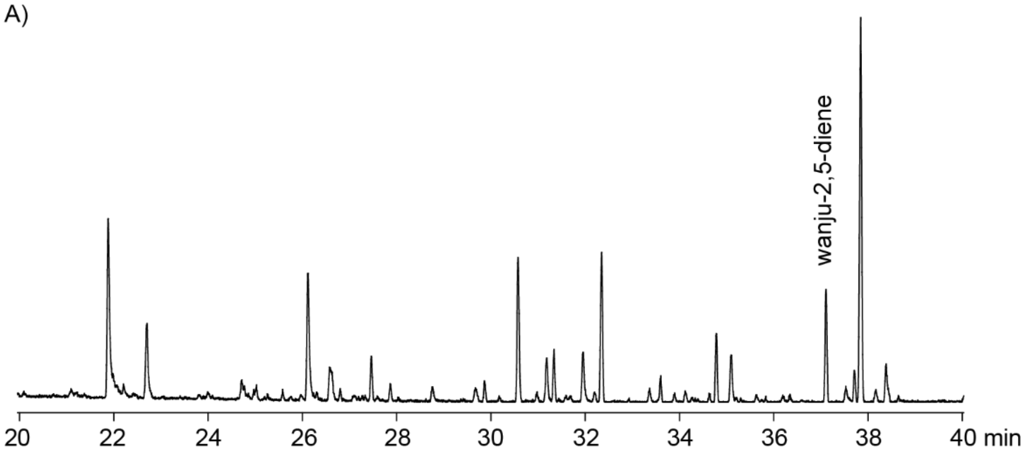

B) wanju-2,5-diene

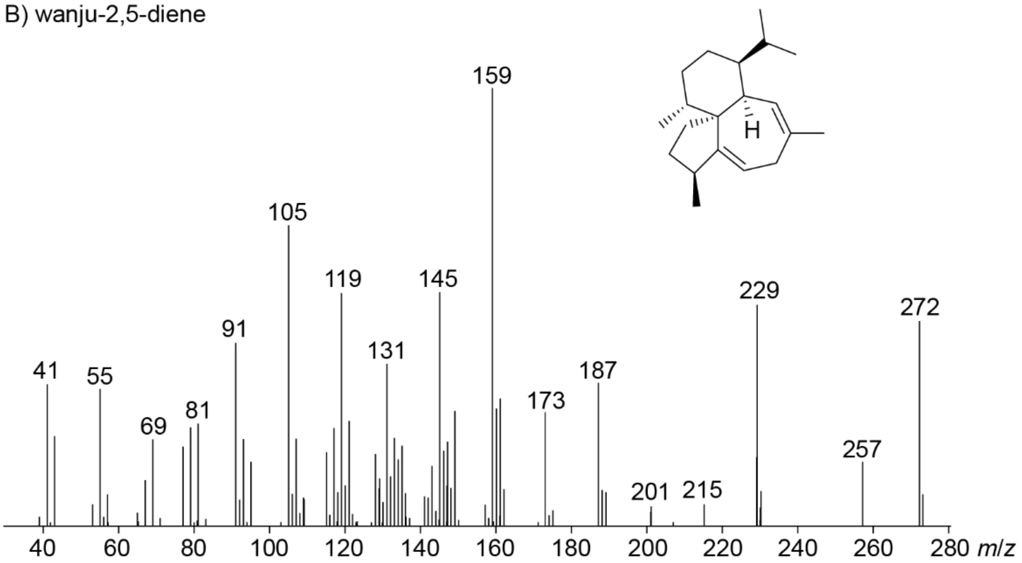

Figure S174. The volatiles released by *Chitinophaga japonensis*. The only compound identified in the headspace extracts that was also observed as an enzyme product (of CjWS) is wanju-2,5-diene (mass specral match factor: 874/1000, retention index *I* = 1939, lit.: *I* = 1945[1]).

Cartesian coordinates of computed structures (Scheme 1 of main text, Table S11)

Gibbs energies (G"..." in Hartree) and imaginary frequencies of TS (T-"..." in cm^{-1}), wB97M-V/Def2-TZVPPD//B97D3/6-31G(d,p)-sp-density-fitting, 1 bar, 298.15 K.

**A**	G781.131261		
C	1.257156	2.257502	0.213082
C	-0.820852	2.044729	-1.284401
C	-2.681665	-0.745409	1.169654
C	0.310819	1.319911	0.596581
C	-1.434889	-0.962978	1.635596
C	-0.587018	1.449771	1.808664
C	-0.789133	0.091727	2.534375
H	-3.198216	0.156623	1.520830
H	-1.574387	1.840607	1.520057
H	-0.162883	2.178761	2.514394
H	-1.412369	0.277481	3.421856
H	0.181847	-0.271074	2.902025
C	-0.585225	-2.170287	1.322081
H	-0.367399	-2.721725	2.250726
H	-1.058020	-2.875450	0.628905
H	0.392730	-1.872932	0.913112
C	-3.423806	-1.495824	0.095098
H	-2.881989	-2.391208	-0.235639
H	-4.414944	-1.828200	0.439597
C	-2.061776	1.389511	-1.114723
H	-2.850987	1.930267	-0.589460
C	-2.336854	0.088658	-1.506539
C	-3.650583	-0.538791	-1.130188
C	-1.330620	-0.795766	-2.178911
H	-0.787752	-1.367411	-1.408386
H	-1.820816	-1.529932	-2.830074
H	-0.586064	-0.246867	-2.765295
H	-4.065040	-1.115386	-1.970274
H	-4.378340	0.235407	-0.849828
C	2.329579	1.962876	-0.805045
H	2.170032	2.634912	-1.669312
H	3.277938	2.321814	-0.364231
C	2.883735	-0.437937	-0.163826
C	2.507839	0.512347	-1.282062
H	3.295155	0.522388	-2.054703
H	1.598526	0.166986	-1.799496
H	0.414820	0.318538	0.180557
C	2.790030	-1.784521	-0.187480
H	3.318379	0.024939	0.729214
C	2.263738	-2.572506	-1.365848
H	1.872878	-1.946682	-2.176966
H	3.064659	-3.197620	-1.791008
H	1.470663	-3.268295	-1.048932
C	3.264110	-2.617926	0.981438

H	4.086609	-3.282790	0.673790
H	3.619211	-2.001038	1.816442
H	2.460506	-3.274146	1.351919
C	1.295346	3.640067	0.804903
H	2.003304	3.648254	1.650961
H	1.668199	4.374894	0.079022
H	0.324727	3.971972	1.188903
H	-0.772673	3.115132	-1.094268
H	-0.087881	1.660860	-1.989155

A-C-TS G781.120828, T-140

C	-0.821449	1.952840	-0.342044
C	0.480931	2.355369	0.441936
C	2.364824	-1.396436	-1.041259
C	-0.944822	0.368158	-0.345856
C	1.231125	-1.938302	-0.553961
C	-0.502113	-0.369612	-1.609015
C	-0.053324	-1.828791	-1.380930
H	2.317850	-0.949570	-2.042789
H	0.352828	0.177562	-2.027253
H	-1.291679	-0.316352	-2.377078
H	0.079372	-2.304983	-2.365154
H	-0.857757	-2.396600	-0.880888
C	1.121451	-2.633922	0.783295
H	0.608779	-3.602761	0.672049
H	2.096458	-2.833550	1.240030
H	0.535670	-2.045765	1.509704
C	3.661570	-1.150912	-0.323740
H	3.710749	-1.686868	0.633582
H	4.514901	-1.499632	-0.925974
C	1.760550	1.754672	-0.059192
H	2.008832	1.967102	-1.104363
C	2.637976	0.986814	0.629302
C	3.836353	0.384318	-0.080483
C	2.506172	0.624786	2.089054
H	2.562110	-0.464271	2.230174
H	3.346396	1.051401	2.659222
H	1.578919	0.977342	2.554668
H	4.754224	0.541178	0.508746
H	3.984131	0.880718	-1.051200
C	-2.070571	2.467757	0.437383
H	-1.817391	3.247985	1.167341
H	-2.796344	2.907545	-0.262272
C	-2.508612	0.154368	0.029263
C	-2.695091	1.240028	1.115567
H	-3.750585	1.370464	1.388441
H	-2.140505	0.974925	2.026929
H	-0.361866	-0.012096	0.507047
C	-2.754750	-1.228272	0.390292
H	-3.076182	0.419320	-0.877362
C	-2.367281	-1.775313	1.710524

H	-1.673302	-1.141172	2.268084
H	-3.297115	-1.858212	2.307565
H	-1.979785	-2.798690	1.626262
C	-3.496775	-2.102534	-0.550806
H	-4.569897	-1.852388	-0.427920
H	-3.262697	-1.863578	-1.597025
H	-3.377458	-3.172833	-0.353841
C	-0.818081	2.555299	-1.759491
H	-1.692680	2.230994	-2.341050
H	-0.860797	3.650539	-1.687496
H	0.081076	2.298822	-2.331789
H	0.539926	3.456243	0.368543
H	0.325324	2.125302	1.506047

**C**	G781.164955		
C	-0.821449	1.952840	-0.342044
C	0.480931	2.355369	0.441936
C	2.364824	-1.396436	-1.041259
C	-0.944822	0.368158	-0.345856
C	1.231125	-1.938302	-0.553961
C	-0.502113	-0.369612	-1.609015
C	-0.053324	-1.828791	-1.380930
H	2.317850	-0.949570	-2.042789
H	0.352828	0.177562	-2.027253
H	-1.291679	-0.316352	-2.377078
H	0.079372	-2.304983	-2.365154
H	-0.857757	-2.396600	-0.880888
C	1.121451	-2.633922	0.783295
H	0.608779	-3.602761	0.672049
H	2.096458	-2.833550	1.240030
H	0.535670	-2.045765	1.509704
C	3.661570	-1.150912	-0.323740
H	3.710749	-1.686868	0.633582
H	4.514901	-1.499632	-0.925974
C	1.760550	1.754672	-0.059192
H	2.008832	1.967102	-1.104363
C	2.637976	0.986814	0.629302
C	3.836353	0.384318	-0.080483
C	2.506172	0.624786	2.089054
H	2.562110	-0.464271	2.230174
H	3.346396	1.051401	2.659222
H	1.578919	0.977342	2.554668
H	4.754224	0.541178	0.508746
H	3.984131	0.880718	-1.051200
C	-2.070571	2.467757	0.437383
H	-1.817391	3.247985	1.167341
H	-2.796344	2.907545	-0.262272
C	-2.508612	0.154368	0.029263
C	-2.695091	1.240028	1.115567
H	-3.750585	1.370464	1.388441
H	-2.140505	0.974925	2.026929

H	-0.361866	-0.012096	0.507047
C	-2.754750	-1.228272	0.390292
H	-3.076182	0.419320	-0.877362
C	-2.367281	-1.775313	1.710524
H	-1.673302	-1.141172	2.268084
H	-3.297115	-1.858212	2.307565
H	-1.979785	-2.798690	1.626262
C	-3.496775	-2.102534	-0.550806
H	-4.569897	-1.852388	-0.427920
H	-3.262697	-1.863578	-1.597025
H	-3.377458	-3.172833	-0.353841
C	-0.818081	2.555299	-1.759491
H	-1.692680	2.230994	-2.341050
H	-0.860797	3.650539	-1.687496
H	0.081076	2.298822	-2.331789
H	0.539926	3.456243	0.368543
H	0.325324	2.125302	1.506047

C G781.158318

C	1.137033	-1.772417	-0.382265
C	0.291050	-2.355804	0.802737
C	-2.366023	0.499918	-0.923503
C	0.799813	-0.224860	-0.526726
C	-1.669605	1.640615	-1.111868
C	0.398333	0.297485	-1.939671
C	-0.361675	1.638870	-1.905583
H	-1.989204	-0.407504	-1.401398
H	-0.230599	-0.457916	-2.422061
H	1.287347	0.394252	-2.583447
H	-0.543406	1.960425	-2.945806
H	0.290501	2.434952	-1.492522
C	-2.123308	2.998856	-0.625481
H	-2.369979	3.641423	-1.486258
H	-3.016315	2.949733	0.007012
H	-1.334151	3.529503	-0.068864
C	-3.565962	0.252824	-0.045843
H	-3.671864	1.046533	0.707620
H	-4.499521	0.258352	-0.632944
C	-1.190327	-2.196223	0.584670
H	-1.606119	-2.777920	-0.244368
C	-2.039598	-1.350821	1.211595
C	-3.438429	-1.138376	0.657220
C	-1.683887	-0.489343	2.400637
H	-2.358478	-0.710247	3.242049
H	-0.656186	-0.631674	2.755182
H	-1.824183	0.578493	2.168303
H	-4.198109	-1.203595	1.452114
H	-3.665847	-1.926669	-0.075563
C	2.662872	-1.815632	-0.016129
H	2.798907	-1.953443	1.067062
H	3.190923	-2.635252	-0.518899

C	2.078865	0.438867	-0.043098
C	3.245087	-0.443809	-0.432845
H	3.386610	-0.414328	-1.523570
H	4.197553	-0.204127	0.048795
H	-0.039816	0.006560	0.137260
C	2.155037	1.545673	0.847799
H	2.143911	1.669046	-0.502856
C	3.476420	2.054163	1.375750
H	3.445548	3.138659	1.529734
H	3.636857	1.587926	2.360669
H	4.329949	1.802707	0.740749
C	0.919436	2.178265	1.432511
H	1.004720	3.270963	1.449855
H	-0.005544	1.882715	0.934351
H	0.864647	1.842665	2.480476
C	0.897437	-2.585297	-1.667686
H	1.521837	-2.232858	-2.499939
H	1.153169	-3.637430	-1.482093
H	-0.148049	-2.556739	-1.996086
H	0.559202	-3.420715	0.908398
H	0.610293	-1.862216	1.733721

C-D-TS G781.155585, T-653

C	1.137033	-1.772417	-0.382265
C	0.291050	-2.355804	0.802737
C	-2.366023	0.499918	-0.923503
C	0.799813	-0.224860	-0.526726
C	-1.669605	1.640615	-1.111868
C	0.398333	0.297485	-1.939671
C	-0.361675	1.638870	-1.905583
H	-1.989204	-0.407504	-1.401398
H	-0.230599	-0.457916	-2.422061
H	1.287347	0.394252	-2.583447
H	-0.543406	1.960425	-2.945806
H	0.290501	2.434952	-1.492522
C	-2.123308	2.998856	-0.625481
H	-2.369979	3.641423	-1.486258
H	-3.016315	2.949733	0.007012
H	-1.334151	3.529503	-0.068864
C	-3.565962	0.252824	-0.045843
H	-3.671864	1.046533	0.707620
H	-4.499521	0.258352	-0.632944
C	-1.190327	-2.196223	0.584670
H	-1.606119	-2.777920	-0.244368
C	-2.039598	-1.350821	1.211595
C	-3.438429	-1.138376	0.657220
C	-1.683887	-0.489343	2.400637
H	-2.358478	-0.710247	3.242049
H	-0.656186	-0.631674	2.755182
H	-1.824183	0.578493	2.168303
H	-4.198109	-1.203595	1.452114

H	-3.665847	-1.926669	-0.075563
C	2.662872	-1.815632	-0.016129
H	2.798907	-1.953443	1.067062
H	3.190923	-2.635252	-0.518899
C	2.078865	0.438867	-0.043098
C	3.245087	-0.443809	-0.432845
H	3.386610	-0.414328	-1.523570
H	4.197553	-0.204127	0.048795
H	-0.039816	0.006560	0.137260
C	2.155037	1.545673	0.847799
H	2.143911	1.669046	-0.502856
C	3.476420	2.054163	1.375750
H	3.445548	3.138659	1.529734
H	3.636857	1.587926	2.360669
H	4.329949	1.802707	0.740749
C	0.919436	2.178265	1.432511
H	1.004720	3.270963	1.449855
H	-0.005544	1.882715	0.934351
H	0.864647	1.842665	2.480476
C	0.897437	-2.585297	-1.667686
H	1.521837	-2.232858	-2.499939
H	1.153169	-3.637430	-1.482093
H	-0.148049	-2.556739	-1.996086
H	0.559202	-3.420715	0.908398
H	0.610293	-1.862216	1.733721

**D**	G781.166865		
C	-1.041958	-1.887310	0.529388
C	-0.166459	-2.423373	-0.660113
C	2.255089	0.695239	0.989425
C	-0.812335	-0.321652	0.619946
C	1.415806	1.750230	1.135636
C	-0.553151	0.314032	2.046442
C	0.212155	1.652809	2.066080
H	2.068668	-0.185927	1.613251
H	0.034894	-0.443841	2.576153
H	-1.497907	0.414286	2.598618
H	0.538282	1.810525	3.110073
H	-0.467342	2.495946	1.869105
C	1.607370	3.087500	0.463679
H	1.806958	3.859062	1.224824
H	2.444842	3.102776	-0.241393
H	0.698970	3.413580	-0.066536
C	3.409231	0.543827	0.038331
H	3.375331	1.307287	-0.750873
H	4.364857	0.689138	0.569977
C	1.283985	-2.097200	-0.451485
H	1.726627	-2.530290	0.451071
C	2.083355	-1.266693	-1.165034
C	3.437291	-0.884132	-0.600612
C	1.709250	-0.616021	-2.473200

H	2.356886	-1.000860	-3.276460
H	0.670625	-0.791890	-2.774471
H	1.880852	0.471052	-2.441313
H	4.213425	-0.896657	-1.381106
H	3.736814	-1.607852	0.171352
C	-2.544371	-2.017187	0.157520
H	-2.749278	-2.924041	-0.424925
H	-3.160297	-2.067515	1.068029
C	-1.890497	0.280236	-0.154174
C	-2.879971	-0.729207	-0.609758
H	-3.912193	-0.365012	-0.487962
H	-2.762696	-0.832009	-1.708726
H	0.161564	-0.057636	0.106559
C	-1.963588	1.705297	-0.556965
H	-1.501565	2.318654	0.231892
C	-3.377623	2.227571	-0.867565
H	-3.325699	3.299737	-1.092193
H	-3.813916	1.727188	-1.741966
H	-4.054527	2.094600	-0.013732
C	-1.029889	1.855022	-1.811583
H	-1.056311	2.907216	-2.121095
H	0.002633	1.579143	-1.580899
H	-1.392207	1.239935	-2.644518
C	-0.740557	-2.674611	1.814309
H	-1.369214	-2.340033	2.649968
H	-0.952065	-3.739929	1.649227
H	0.307949	-2.596638	2.127755
H	-0.320140	-3.513240	-0.722498
H	-0.533618	-2.003008	-1.608789

**D**	G781.168330		
C	0.943776	-1.934556	-0.587902
C	0.058517	-2.551939	0.557622
C	-2.293360	0.803599	-0.898161
C	0.715444	-0.368625	-0.577588
C	-1.424541	1.839856	-0.988788
C	0.478116	0.362289	-1.960674
C	-0.203328	1.743376	-1.898801
H	-2.126898	-0.055925	-1.556505
H	-0.157000	-0.328853	-2.526013
H	1.426317	0.432945	-2.511071
H	-0.491885	1.998267	-2.934398
H	0.518843	2.521163	-1.612665
C	-1.605440	3.159287	-0.275644
H	-0.718239	3.449981	0.308653
H	-1.751871	3.959057	-1.019597
H	-2.473996	3.173976	0.390472
C	-3.443018	0.633602	0.054864
H	-3.402077	1.377514	0.861737
H	-4.405210	0.783997	-0.462259
C	-1.382873	-2.157523	0.412998

H	-1.912242	-2.616009	-0.428287
C	-2.072477	-1.231806	1.127411
C	-3.451046	-0.809208	0.661016
C	-1.528592	-0.490332	2.325498
H	-2.232438	-0.556566	3.168308
H	-0.559532	-0.868896	2.669495
H	-1.417598	0.583547	2.099890
H	-4.171983	-0.829298	1.493193
H	-3.812932	-1.512888	-0.102399
C	2.440398	-2.073786	-0.199531
H	2.660360	-3.038260	0.274826
H	3.077821	-1.991465	-1.093210
C	1.750097	0.172215	0.284285
C	2.702745	-0.877645	0.730625
H	3.739210	-0.507733	0.771827
H	2.456197	-1.110591	1.786723
H	-0.270325	-0.129647	-0.067299
C	1.867229	1.587226	0.714907
H	0.926532	2.101586	0.486912
C	3.002316	2.251727	-0.133133
H	3.018674	3.322540	0.104197
H	3.980087	1.825403	0.121544
H	2.841541	2.139672	-1.211837
C	2.163230	1.730236	2.226068
H	2.189342	2.796517	2.482463
H	1.382004	1.252106	2.830730
H	3.134138	1.295703	2.495378
C	0.648799	-2.635378	-1.921760
H	1.269173	-2.237861	-2.735684
H	0.870857	-3.707330	-1.831352
H	-0.403502	-2.544804	-2.220712
H	0.168214	-3.646632	0.508645
H	0.452452	-2.246910	1.538425

D-E-TS G781.162220, T-616

C	-0.844941	2.001929	-0.380084
C	0.384875	2.292679	0.557144
C	2.501927	-1.326451	-1.089373
C	-0.978924	0.479541	-0.655343
C	1.281525	-1.832566	-0.820312
C	-0.348292	-0.161159	-1.867247
C	0.172003	-1.623521	-1.848592
H	2.641510	-0.849237	-2.068411
H	0.480854	0.483756	-2.177903
H	-1.126333	-0.055095	-2.647052
H	0.568054	-1.796222	-2.859479
H	-0.646862	-2.344434	-1.724236
C	0.934344	-2.563595	0.460376
H	0.381646	-1.936823	1.182820
H	0.301163	-3.439094	0.253185
H	1.826099	-2.922642	0.984597

C	3.657233	-1.160729	-0.146752
H	3.548285	-1.780641	0.753485
H	4.600175	-1.460703	-0.628406
C	1.693564	1.686191	0.136207
H	2.040432	1.950342	-0.868512
C	2.502326	0.888497	0.869620
C	3.781445	0.344576	0.259127
C	2.235229	0.453781	2.292546
H	2.974357	0.910042	2.969337
H	1.240425	0.723009	2.667467
H	2.352144	-0.634297	2.399040
H	4.615542	0.444006	0.971623
H	4.047585	0.928667	-0.634264
C	-2.151213	2.303146	0.407823
H	-2.039306	3.172825	1.066630
H	-2.969877	2.521560	-0.295345
C	-1.878903	-0.094314	0.283015
C	-2.465522	1.000749	1.169687
H	-3.540034	0.821392	1.303217
H	-2.025479	0.964563	2.177431
H	-0.576544	-0.123799	0.485982
C	-2.437033	-1.502281	0.316220
H	-1.711521	-2.181400	-0.142458
C	-3.711203	-1.512429	-0.567164
H	-4.122495	-2.529185	-0.585474
H	-4.485783	-0.845141	-0.166943
H	-3.493044	-1.212230	-1.600435
C	-2.721900	-1.989154	1.747949
H	-3.081964	-3.024589	1.715674
H	-1.819377	-1.966942	2.373389
H	-3.496128	-1.385933	2.239661
C	-0.752347	2.823577	-1.678675
H	-1.567826	2.581851	-2.373356
H	-0.833615	3.888504	-1.424930
H	0.201458	2.682888	-2.200770
H	0.468256	3.392366	0.600960
H	0.123060	1.971836	1.576444

**E**	G781.169078		
C	-0.869193	1.590042	-0.448019
C	0.538498	1.977254	0.263646
C	2.646765	-1.783914	-0.808247
C	-1.024283	0.114391	-0.415987
C	1.475590	-1.916690	-0.142577
C	-0.395731	-0.783877	-1.391569
C	0.200691	-2.157346	-0.917085
H	2.632116	-1.958500	-1.890848
H	0.343946	-0.248089	-1.997487
H	-1.236562	-1.011787	-2.082553
H	0.396513	-2.734933	-1.829750
H	-0.544939	-2.707776	-0.325672

C	1.305314	-1.668317	1.333577
H	0.852589	-0.679591	1.521061
H	0.635650	-2.417294	1.783748
H	2.251433	-1.686380	1.881990
C	3.930221	-1.245919	-0.248052
H	4.048508	-1.492852	0.816165
H	4.791380	-1.680800	-0.774384
C	1.789161	1.424621	-0.338537
H	1.815862	1.348075	-1.428993
C	2.897042	1.019175	0.327178
C	4.006792	0.318948	-0.424869
C	3.113033	1.186804	1.812799
H	3.799181	2.030605	1.987657
H	2.197717	1.391899	2.380653
H	3.594657	0.302379	2.251737
H	4.993699	0.646299	-0.064054
H	3.953658	0.551253	-1.498519
C	-2.014368	2.103433	0.477805
H	-1.733851	3.035558	0.984211
H	-2.903468	2.320439	-0.130935
C	-1.953391	-0.338765	0.645882
C	-2.286891	0.942571	1.454837
H	-3.315585	0.942686	1.831582
H	-1.626429	1.004696	2.331419
H	-1.465746	-1.120834	1.251397
C	-3.215160	-1.096830	0.020821
H	-2.795769	-1.906132	-0.599468
C	-4.084994	-0.198216	-0.868546
H	-4.903110	-0.788886	-1.300174
H	-4.542715	0.617176	-0.293275
H	-3.520856	0.245255	-1.701253
C	-4.032405	-1.740550	1.148990
H	-4.864234	-2.313205	0.717038
H	-3.424079	-2.425937	1.753957
H	-4.468304	-0.986607	1.817951
C	-0.894389	2.211834	-1.861937
H	-1.828223	1.961840	-2.381291
H	-0.842308	3.303740	-1.763975
H	-0.054485	1.892920	-2.489072
H	0.501712	3.078566	0.188936
H	0.468240	1.734316	1.331549

**E**	G781.165854		
C	-0.641365	1.595817	-0.225921
C	0.849877	2.009978	0.028254
C	2.535250	-2.030234	-0.467080
C	-1.064733	0.158872	-0.265391
C	1.413018	-1.871752	0.278714
C	-0.487384	-0.892581	-1.104875
C	0.082972	-2.184083	-0.338545
H	2.405728	-2.459060	-1.468399

H	0.298572	-0.521060	-1.763465
H	-1.312434	-1.283139	-1.722118
H	0.166534	-2.956067	-1.112922
H	-0.659930	-2.514889	0.399565
C	1.352999	-1.265391	1.652258
H	0.822869	-1.932391	2.350036
H	2.334930	-1.035685	2.073598
H	0.781781	-0.318461	1.631414
C	3.906265	-1.511021	-0.159896
H	4.105954	-1.486181	0.920008
H	4.670264	-2.156913	-0.614558
C	1.951344	1.228368	-0.632001
H	1.784423	0.894446	-1.660001
C	3.135673	0.907584	-0.064403
C	4.071670	-0.061549	-0.753939
C	3.602235	1.403869	1.285878
H	4.325716	2.222475	1.144292
H	2.801262	1.789743	1.927268
H	4.132656	0.619036	1.843292
H	5.122066	0.238698	-0.620912
H	3.872911	-0.098458	-1.835223
C	-1.565409	2.227513	0.858662
H	-1.063655	2.158365	1.836596
H	-1.757337	3.290023	0.663597
C	-2.305228	-0.057623	0.532891
C	-2.832705	1.364906	0.861782
H	-3.535449	1.687786	0.081197
H	-3.368451	1.393575	1.816235
H	-1.871545	-0.462191	1.482751
C	-3.338990	-1.111533	0.037176
H	-2.787561	-2.044255	-0.166421
C	-4.046526	-0.676780	-1.260552
H	-4.682408	-1.491891	-1.628103
H	-4.698409	0.190102	-1.089212
H	-3.347267	-0.414836	-2.068019
C	-4.362844	-1.412768	1.144949
H	-5.062551	-2.191161	0.815231
H	-3.874287	-1.763480	2.064779
H	-4.955535	-0.521333	1.392146
C	-1.086371	2.082391	-1.665368
H	-2.122822	1.818777	-1.908268
H	-1.005636	3.177180	-1.660886
H	-0.427948	1.678180	-2.441538
H	0.891868	3.077340	-0.254626
H	0.997199	1.991814	1.117116

E-F-TS G781.156210, T-314

C	-0.388266	1.495262	-0.185177
C	0.934592	2.121096	-0.643157
C	2.477017	-1.968220	-0.483807
C	-1.028902	0.277702	-0.557434

C	1.257591	-1.853152	0.082350
C	-0.522419	-0.803190	-1.487806
C	0.017711	-2.080747	-0.763332
H	2.519174	-2.270576	-1.537515
H	0.261862	-0.410427	-2.144808
H	-1.363716	-1.113816	-2.126289
H	0.242592	-2.793014	-1.569264
H	-0.789130	-2.528435	-0.164132
C	1.048644	-1.465123	1.530703
H	0.223093	-2.039183	1.977746
H	1.938827	-1.645372	2.142687
H	0.806017	-0.396171	1.644558
C	3.784180	-1.544017	0.121148
H	3.757989	-1.570705	1.219550
H	4.594433	-2.217179	-0.194805
C	2.097436	1.177435	-0.843548
H	2.147297	0.682314	-1.815537
C	3.093693	0.919003	0.030071
C	4.155133	-0.095842	-0.350835
C	3.211078	1.510372	1.413426
H	4.135665	2.101550	1.498199
H	2.372347	2.156852	1.698532
H	3.285923	0.712111	2.167093
H	5.125378	0.176923	0.090427
H	4.286441	-0.110193	-1.442746
C	-1.050040	2.042840	1.065619
H	-0.354796	1.788969	1.886382
H	-1.139166	3.137907	1.057442
C	-2.089652	-0.070870	0.499981
C	-2.376406	1.274518	1.204252
H	-3.190035	1.815368	0.699051
H	-2.676580	1.143656	2.248638
H	-1.501969	-0.684076	1.208897
C	-3.306408	-0.951908	0.111030
H	-2.899156	-1.892013	-0.297682
C	-4.255152	-0.367718	-0.952213
H	-5.139237	-1.010623	-1.050384
H	-4.622924	0.632687	-0.680195
H	-3.799989	-0.319910	-1.951983
C	-4.091492	-1.309530	1.388743
H	-4.867450	-2.052714	1.166444
H	-3.435756	-1.728149	2.165285
H	-4.593124	-0.426274	1.808828
C	-1.614744	1.656712	-1.623018
H	-2.623179	1.248703	-1.698605
H	-1.717235	2.707329	-1.334417
H	-1.039225	1.550602	-2.541014
H	0.758899	2.677521	-1.577092
H	1.175179	2.888599	0.104737

F G781.159781

C	-0.035408	-1.377075	0.137119
C	0.974351	-2.222557	0.803796
C	2.267232	1.828465	0.647913
C	-1.071556	-0.508016	0.816660
C	1.004938	1.968129	0.178719
C	-0.571429	0.620335	1.776997
C	-0.158622	1.983849	1.159287
H	2.411244	1.798883	1.735031
H	0.248386	0.226769	2.395277
H	-1.396365	0.822496	2.476430
H	0.110062	2.615627	2.020783
H	-1.038341	2.457658	0.702596
C	0.720224	2.155354	-1.300702
H	-0.238871	2.664659	-1.459655
H	1.494482	2.758957	-1.788762
H	0.670473	1.202653	-1.854680
C	3.487373	1.558559	-0.173553
H	3.291608	1.668502	-1.248061
H	4.300049	2.255243	0.085409
C	2.113056	-1.202519	0.800690
H	2.144442	-0.581530	1.694500
C	3.036226	-0.967348	-0.172734
C	4.059301	0.112603	0.097751
C	3.079467	-1.660715	-1.503425
H	4.100692	-2.004486	-1.720864
H	2.404241	-2.520377	-1.575497
H	2.816641	-0.956408	-2.311015
H	4.952084	-0.029827	-0.525577
H	4.378272	0.072090	1.149100
C	-0.316000	-1.493080	-1.322049
H	0.321938	-0.758211	-1.850737
H	-0.055684	-2.478668	-1.730421
C	-1.941535	0.021427	-0.380030
C	-1.796329	-1.064330	-1.461758
H	-2.448408	-1.926811	-1.266235
H	-2.022470	-0.698398	-2.469931
H	-1.399789	0.896105	-0.764078
C	-3.380976	0.513953	-0.060436
H	-3.373818	0.902875	0.973688
C	-4.472428	-0.569612	-0.156813
H	-5.433587	-0.160998	0.181003
H	-4.609362	-0.896925	-1.197334
H	-4.263776	-1.460655	0.447551
C	-3.745287	1.690613	-0.988296
H	-4.761567	2.052049	-0.784144
H	-3.056664	2.538421	-0.863185
H	-3.711657	1.381662	-2.043786
C	-1.854444	-1.538443	1.716361
H	-2.761147	-1.047295	2.084986
H	-2.151857	-2.441586	1.170154

H	-1.247529	-1.834943	2.579246
H	0.726154	-2.489710	1.836343
H	1.211195	-3.125364	0.228877

F G781.159788

C	-0.035736	-1.377141	0.137380
C	0.974295	-2.222165	0.804230
C	2.267509	1.828780	0.647761
C	-1.071718	-0.507751	0.816649
C	1.005223	1.967978	0.178408
C	-0.571530	0.620752	1.776731
C	-0.158461	1.984119	1.158779
H	2.411542	1.799843	1.734891
H	0.248220	0.227285	2.395166
H	-1.396485	0.823183	2.476049
H	0.110237	2.615968	2.020213
H	-1.038040	2.457989	0.701879
C	0.720809	2.154144	-1.301233
H	-0.239391	2.661067	-1.461002
H	1.493915	2.759553	-1.788928
H	0.673968	1.201168	-1.854990
C	3.487617	1.558622	-0.173672
H	3.291858	1.668562	-1.248182
H	4.300359	2.255233	0.085260
C	2.113055	-1.202163	0.800858
H	2.144354	-0.580835	1.694428
C	3.036264	-0.967353	-0.172576
C	4.059414	0.112617	0.097677
C	3.079858	-1.661250	-1.502973
H	4.100825	-2.006724	-1.719041
H	2.403423	-2.519902	-1.575577
H	2.819208	-0.956753	-2.311076
H	4.952122	-0.029916	-0.525739
H	4.378530	0.072171	1.148985
C	-0.316476	-1.493692	-1.321676
H	0.321652	-0.759143	-1.850629
H	-0.056325	-2.479453	-1.729750
C	-1.941718	0.021414	-0.380167
C	-1.796700	-1.064671	-1.461572
H	-2.448947	-1.926976	-1.265855
H	-2.022712	-0.698951	-2.469846
H	-1.399878	0.895917	-0.764492
C	-3.381055	0.514140	-0.060509
H	-3.373769	0.903209	0.973559
C	-4.472622	-0.569335	-0.156697
H	-5.433655	-0.160704	0.181440
H	-4.609859	-0.896444	-1.197252
H	-4.263853	-1.460532	0.447392
C	-3.745396	1.690728	-0.988462
H	-4.761642	2.052232	-0.784256
H	-3.056732	2.538528	-0.863501

H	-3.711882	1.381672	-2.043925
C	-1.854770	-1.537948	1.716592
H	-2.761464	-1.046569	2.084933
H	-2.152123	-2.441193	1.170549
H	-1.247950	-1.834227	2.579615
H	0.726178	-2.489019	1.836883
H	1.211152	-3.125175	0.229637

F-G-TS G781.146627, T-867

C	0.439466	-1.260369	0.282295
C	-0.938618	-1.625690	0.198032
C	-2.512871	1.804578	-0.425843
C	1.202219	-0.199410	-0.508727
C	-1.630337	1.669531	0.588214
C	0.519816	1.172314	-0.804535
C	-0.142996	1.959313	0.357991
H	-2.123771	2.156939	-1.388665
H	-0.203392	1.061093	-1.622170
H	1.327681	1.788897	-1.224109
H	-0.020823	3.030780	0.135328
H	0.412672	1.795784	1.293991
C	-2.009641	1.264596	1.993014
H	-1.723850	2.055060	2.704517
H	-3.077853	1.066174	2.123773
H	-1.463635	0.363579	2.336677
C	-3.949679	1.361728	-0.458550
H	-4.387775	1.289716	0.545752
H	-4.563064	2.079666	-1.021687
C	-1.934820	-1.075458	-0.703743
H	-1.576498	-0.488543	-1.545785
C	-3.276342	-1.053362	-0.439674
C	-4.102234	-0.037565	-1.183569
C	-3.959685	-1.797931	0.672031
H	-4.771221	-2.411944	0.252856
H	-3.306369	-2.453944	1.257331
H	-4.441192	-1.090885	1.364841
H	-5.165932	-0.309946	-1.206727
H	-3.751505	0.068602	-2.220445
C	1.373683	-1.998485	1.229364
H	1.085034	-1.773480	2.269602
H	1.280145	-3.087370	1.108191
C	2.505402	-0.031312	0.362341
C	2.777427	-1.440787	0.919963
H	3.272172	-2.073382	0.171439
H	3.419630	-1.427704	1.808025
H	2.193978	0.591941	1.222340
C	3.718702	0.692300	-0.290680
H	3.321044	1.402919	-1.036334
C	4.714949	-0.231423	-1.017974
H	5.477673	0.374988	-1.523633
H	5.241873	-0.885841	-0.309347

H	4.244861	-0.867149	-1.777904
C	4.463354	1.523218	0.773085
H	5.320442	2.048466	0.331773
H	3.805452	2.274539	1.232276
H	4.850706	0.876675	1.574706
C	1.493122	-0.883325	-1.883150
H	2.110830	-0.211032	-2.490283
H	2.029664	-1.833089	-1.776039
H	0.561320	-1.076874	-2.428525
H	-1.229205	-2.467603	0.836414
H	-0.654589	-0.741781	1.042512

**G**	G781.163866		
C	0.341370	-0.916249	0.654368
C	-1.040611	-1.383952	0.469142
C	-2.450607	1.745860	-0.526580
C	1.157645	-0.142792	-0.421443
C	-1.585283	1.782593	0.518739
C	0.586087	1.252538	-0.816182
C	-0.109976	2.106595	0.275048
H	-2.055182	1.983070	-1.520244
H	-0.103562	1.137611	-1.665842
H	1.427363	1.833983	-1.220476
H	-0.033985	3.164088	-0.026196
H	0.441245	2.039142	1.225338
C	-1.994720	1.589588	1.957701
H	-1.847438	2.532262	2.508199
H	-3.041191	1.294190	2.090232
H	-1.361412	0.846625	2.466250
C	-3.866521	1.281489	-0.518839
H	-4.300309	1.219131	0.486073
H	-4.506822	1.945411	-1.116867
C	-1.888211	-1.111274	-0.586674
H	-1.494933	-0.583180	-1.451850
C	-3.293192	-1.161604	-0.449488
C	-4.050157	-0.162209	-1.232847
C	-4.028889	-1.981369	0.555053
H	-4.729396	-2.638073	0.012607
H	-3.396294	-2.611638	1.186534
H	-4.661248	-1.339229	1.188447
H	-5.124083	-0.379968	-1.270508
H	-3.654683	-0.062531	-2.253041
C	1.303100	-2.033272	1.195694
H	1.057134	-2.296732	2.232561
H	1.198736	-2.943500	0.591097
C	2.515253	-0.018654	0.364127
C	2.713763	-1.403845	1.037167
H	3.335062	-2.059335	0.416465
H	3.221648	-1.305503	2.005183
H	2.305237	0.699270	1.180075
C	3.749498	0.550253	-0.396300

H	3.367511	1.246618	-1.163988
C	4.626483	-0.489517	-1.121178
H	5.378404	0.026133	-1.733341
H	5.173386	-1.120129	-0.405982
H	4.057122	-1.149942	-1.785656
C	4.625022	1.376965	0.566544
H	5.496629	1.796124	0.046206
H	4.062208	2.211073	1.009442
H	5.002180	0.749655	1.388121
C	1.333750	-0.974216	-1.712112
H	1.956104	-0.426728	-2.430010
H	1.816593	-1.941235	-1.526817
H	0.375194	-1.177107	-2.208010
H	-1.458222	-1.894465	1.344042
H	0.234170	-0.236675	1.530319

**H**	G3430.994502		
C	1.914668	1.448868	-0.629533
C	-0.306886	2.332739	0.438256
C	-1.537191	-1.391756	0.711487
C	1.984002	0.108378	0.187374
C	-0.255349	-0.914120	1.227661
C	0.664812	-0.717277	-0.053934
H	1.444439	1.178437	-1.591865
H	-0.412608	2.479836	1.520203
H	0.904057	-1.717603	-0.425545
H	0.074233	-0.219598	-0.829669
C	-2.817645	-0.663473	0.908258
H	-3.270958	-1.333336	1.677695
C	-1.423765	2.033278	-0.262615
C	-2.758413	1.924406	0.464230
C	-2.899655	0.765387	1.470595
H	-3.885502	0.849621	1.950086
H	-2.157711	0.855284	2.278765
C	3.381048	1.828954	-0.920065
H	3.466658	2.343556	-1.886296
H	3.738695	2.534667	-0.160552
C	3.170050	-0.635406	-0.549332
C	4.177352	0.501844	-0.878503
H	4.955818	0.540686	-0.102101
H	4.688825	0.298288	-1.827384
H	2.756817	-1.005303	-1.507955
C	3.845773	-1.832789	0.161659
H	4.303085	-1.453866	1.093059
C	4.985140	-2.393940	-0.715233
H	5.467594	-3.246996	-0.219708
H	4.595693	-2.746494	-1.682449
H	5.760115	-1.645181	-0.916613
C	2.896310	-2.985143	0.540936
H	3.461654	-3.804581	1.004288
H	2.124435	-2.683901	1.263843

H	2.397764	-3.400773	-0.349088
C	2.326137	0.340619	1.670701
H	2.422127	-0.599123	2.228411
H	3.286679	0.862594	1.767967
H	1.569278	0.946179	2.188281
H	-0.331714	0.015931	1.790836
H	0.224943	-1.698340	1.834319
C	-1.563872	-2.701033	0.014288
H	-2.528991	-3.213903	0.120253
H	-1.471341	-2.503810	-1.070153
H	-0.725429	-3.342383	0.307610
Br	-4.039018	-0.908444	-0.633651
H	-2.938322	2.847394	1.036394
H	-3.576768	1.851423	-0.264932
C	-1.481616	1.890212	-1.765107
H	-2.146009	2.658684	-2.191722
H	-0.506513	1.985589	-2.254708
H	-1.925434	0.921514	-2.044808
C	1.087800	2.648982	-0.082374
H	0.981756	3.339480	-0.936967
O	1.786732	3.436973	0.887204
H	1.992233	2.873417	1.648164

H-I-TS G3430.992083, T-39

C	2.040086	1.406850	-0.652046
C	-0.252239	2.184762	0.369253
C	-1.566812	-1.301666	0.298171
C	2.080619	0.056892	0.152475
C	-0.239189	-1.086466	0.907954
C	0.811190	-0.789892	-0.226941
H	1.654984	1.139397	-1.652441
H	-0.327929	2.272940	1.459473
H	1.126819	-1.754118	-0.636278
H	0.289482	-0.269328	-1.036853
C	-2.804746	-0.698554	0.853307
H	-3.023265	-1.422965	1.672767
C	-1.402626	1.931309	-0.304912
C	-2.700559	1.858969	0.487423
C	-2.788158	0.702861	1.499893
H	-3.712119	0.796100	2.086280
H	-1.953607	0.745495	2.214784
C	3.510924	1.844248	-0.825679
H	3.657603	2.340817	-1.794004
H	3.781088	2.581590	-0.060133
C	3.360234	-0.622946	-0.471798
C	4.352223	0.551267	-0.685716
H	5.032746	0.624289	0.176011
H	4.981450	0.373813	-1.566447
H	3.057227	-0.990768	-1.471968
C	4.003632	-1.810311	0.285333
H	4.353946	-1.432768	1.262850

C	5.241661	-2.322871	-0.479194
H	5.698853	-3.169377	0.050500
H	4.962477	-2.670954	-1.485439
H	6.009356	-1.548141	-0.591557
C	3.054241	-2.992823	0.553490
H	3.591144	-3.802253	1.065886
H	2.203132	-2.719755	1.193693
H	2.661317	-3.410874	-0.386645
C	2.270773	0.282278	1.665586
H	2.322932	-0.662369	2.221466
H	3.212886	0.811121	1.861847
H	1.460711	0.873639	2.114055
H	-0.251842	-0.323908	1.686529
H	0.057989	-2.047850	1.372350
C	-1.667722	-2.271860	-0.811951
H	-2.584038	-2.876244	-0.740161
H	-1.806719	-1.696733	-1.748629
H	-0.776344	-2.895870	-0.919320
Br	-4.371810	-0.871887	-0.334420
H	-2.821181	2.787505	1.066912
H	-3.561845	1.804881	-0.191726
C	-1.518268	1.868522	-1.808615
H	-2.070042	2.745689	-2.183924
H	-0.548623	1.841714	-2.318704
H	-2.106372	0.990482	-2.119240
C	1.125246	2.562766	-0.150145
H	0.989731	3.233028	-1.016341
O	1.740210	3.409452	0.828932
H	2.066680	2.852197	1.552304

I G3431.037492

C	1.381425	1.007017	-0.704525
C	-0.977980	0.547031	0.371172
C	-1.090382	-0.915092	-0.248561
C	2.105766	-0.086069	0.159041
C	0.042944	-1.801726	0.348721
C	1.492556	-1.493846	-0.081667
H	1.217128	0.542907	-1.688700
H	-0.559256	0.347113	1.378156
H	2.109114	-2.241041	0.430508
H	1.598430	-1.721903	-1.152772
C	-2.444324	-1.484647	0.241663
H	-2.556004	-2.544024	-0.020615
C	-2.323945	1.096588	0.748469
C	-2.978715	0.350938	1.868915
C	-2.773507	-1.182741	1.705721
H	-3.687770	-1.703479	2.014550
H	-1.958236	-1.559682	2.339727
C	2.413138	2.138258	-0.933933
H	2.257341	2.612885	-1.912423
H	2.294205	2.936041	-0.190997

C	3.557686	-0.033548	-0.470426
C	3.797171	1.464134	-0.807650
H	4.382272	1.939252	-0.005991
H	4.387811	1.570778	-1.725447
H	3.494314	-0.588564	-1.427033
C	4.755322	-0.628629	0.319766
H	4.867444	-0.041185	1.249604
C	6.055624	-0.451397	-0.493626
H	6.910571	-0.864936	0.057824
H	5.990467	-0.986940	-1.453242
H	6.279282	0.600078	-0.709131
C	4.617500	-2.112386	0.708948
H	5.559020	-2.474955	1.143017
H	3.834753	-2.293128	1.457015
H	4.401171	-2.736775	-0.170869
C	2.165497	0.276699	1.659253
H	2.742383	-0.465209	2.224220
H	2.657162	1.247183	1.821727
H	1.176312	0.318804	2.139908
H	-0.023545	-1.773918	1.447163
H	-0.158891	-2.845952	0.059434
C	-1.034657	-0.927532	-1.794568
H	-1.463592	-1.868368	-2.165830
H	-1.599406	-0.107454	-2.249334
H	-0.009896	-0.873089	-2.163407
Br	-3.885863	-0.527687	-0.800860
H	-2.481633	0.707739	2.791854
H	-4.036399	0.622188	1.961498
C	-2.794353	2.437925	0.362533
H	-2.139672	3.143664	0.910496
H	-2.604127	2.654900	-0.695691
H	-3.840823	2.615951	0.624932
C	0.003096	1.566056	-0.264315
H	-0.455315	2.018449	-1.158899
O	0.102861	2.657041	0.671530
H	0.778516	2.423202	1.327017

**J**	G857.257587		
C	0.539688	-0.923170	0.953426
C	-1.732890	-1.322144	-0.128424
C	-1.178726	2.255967	0.208031
C	1.412213	0.084301	0.102915
C	0.296154	2.527074	-0.026248
C	1.291091	1.544845	0.647895
H	0.800199	-0.696337	2.004100
H	-1.287637	-1.860457	-0.969597
H	2.275094	2.024904	0.587771
H	1.070607	1.490570	1.727109
C	-1.963826	1.835869	-0.802982
H	-1.489085	1.730481	-1.785958
C	-3.240868	-1.178802	-0.234033

C	-3.648211	-0.041529	-1.219097
C	-3.411278	1.424912	-0.775584
H	-3.973117	2.064077	-1.480028
H	-3.867247	1.596216	0.209338
C	1.140094	-2.304795	0.634948
H	0.908428	-3.031596	1.425916
H	0.706257	-2.707125	-0.291266
C	2.849702	-0.497707	0.384751
C	2.655918	-2.046508	0.466967
H	3.033924	-2.527852	-0.447793
H	3.234012	-2.469068	1.300598
H	3.142471	-0.133984	1.389785
C	4.004643	-0.133445	-0.586540
H	3.754293	-0.570045	-1.570944
C	5.319356	-0.787032	-0.111263
H	6.142155	-0.555436	-0.803155
H	5.603632	-0.403874	0.881572
H	5.242700	-1.879009	-0.037514
C	4.251814	1.371551	-0.796063
H	5.112131	1.520372	-1.465236
H	3.394010	1.886601	-1.244555
H	4.488983	1.872359	0.155368
C	1.071739	-0.021771	-1.395547
H	1.644691	0.699276	-1.992723
H	1.299871	-1.020639	-1.791764
H	0.008266	0.165577	-1.571264
H	0.499137	2.569011	-1.106296
H	0.535328	3.531351	0.368800
C	-1.658981	2.486634	1.622034
H	-1.509464	3.540424	1.909840
H	-2.719202	2.249889	1.765974
H	-1.081407	1.887275	2.343070
H	-3.136299	-0.228354	-2.176514
H	-4.729299	-0.164271	-1.412751
C	-3.935185	-1.020917	1.130278
H	-3.701061	-1.883727	1.765107
H	-3.621508	-0.109464	1.654420
H	-5.026267	-0.964309	0.995282
C	-0.964355	-0.829619	0.853471
H	-1.454366	-0.292440	1.668053
O	-3.658504	-2.436775	-0.828962
H	-4.620748	-2.378471	-0.946647

J-K-TS G857.242040, T-84

C	0.727652	-1.436391	0.676866
C	-1.640301	-0.952757	-0.048613
C	-1.377332	2.245868	0.171488
C	1.358259	-0.148495	0.024529
C	0.137168	2.307774	0.039903
C	0.876892	1.146653	0.791925
H	0.928122	-1.320134	1.763536

H	-1.266405	-0.063018	-0.528329
H	1.753644	1.587058	1.283351
H	0.232124	0.813664	1.617140
C	-2.149611	1.896588	-0.879001
H	-1.644975	1.766433	-1.845016
C	-3.151032	-1.093260	-0.109749
C	-3.734975	-0.087618	-1.149372
C	-3.587790	1.439279	-0.861457
H	-4.159935	1.967338	-1.643596
H	-4.077250	1.679508	0.092921
C	1.627909	-2.600127	0.205957
H	1.583138	-3.452278	0.899549
H	1.278293	-2.970536	-0.769087
C	2.881349	-0.445552	0.319237
C	3.043926	-1.980341	0.097297
H	3.480127	-2.176596	-0.894259
H	3.737489	-2.412788	0.831646
H	3.030268	-0.242578	1.398368
C	3.958367	0.362193	-0.446545
H	3.864358	0.109222	-1.518418
C	5.369763	-0.069149	0.001996
H	6.140846	0.498952	-0.538429
H	5.509658	0.122313	1.077635
H	5.556065	-1.136060	-0.174556
C	3.834090	1.890396	-0.312972
H	4.641238	2.385925	-0.872364
H	2.881863	2.275687	-0.695459
H	3.925196	2.204828	0.738160
C	1.152160	-0.096759	-1.507006
H	1.704427	0.740481	-1.951783
H	1.520566	-1.013002	-1.987147
H	0.104914	0.011898	-1.800056
H	0.420131	2.327002	-1.021098
H	0.496513	3.256530	0.474159
C	-1.914217	2.468764	1.568480
H	-1.448756	3.359307	2.019151
H	-3.000397	2.612310	1.588468
H	-1.678618	1.622157	2.232709
H	-3.271107	-0.321776	-2.120906
H	-4.811855	-0.307721	-1.252124
C	-3.777410	-0.892989	1.285842
H	-3.408447	-1.668795	1.968397
H	-3.518021	0.085178	1.708098
H	-4.876995	-0.958959	1.233858
C	-0.749851	-1.734297	0.572916
H	-1.078158	-2.647781	1.083993
O	-3.432633	-2.438136	-0.566280
H	-4.397116	-2.547345	-0.518590

K G857.258071

C	-0.480855	-0.994832	-0.376105

C	2.025396	-1.096931	-0.582592
C	1.185641	2.144452	-0.195813
C	-1.509453	0.094975	0.108402
C	-0.280531	2.477117	-0.007116
C	-1.291393	1.464014	-0.605707
H	-0.440300	-0.918878	-1.478431
H	1.923208	-1.288342	-1.656654
H	-2.253202	1.987172	-0.649539
H	-1.025000	1.262324	-1.656585
C	1.978570	1.878736	0.861463
H	1.519248	1.927834	1.856514
C	3.446875	-1.054423	-0.044156
C	3.658345	-0.057057	1.127858
C	3.425626	1.454754	0.850268
H	3.955107	2.011660	1.641672
H	3.918381	1.733932	-0.092015
C	-1.153913	-2.341521	-0.024727
H	-0.764305	-3.151577	-0.656002
H	-0.914621	-2.615787	1.014133
C	-2.865794	-0.556318	-0.371167
C	-2.669256	-2.095594	-0.197675
H	-3.224375	-2.454447	0.682402
H	-3.071892	-2.642358	-1.061709
H	-2.945689	-0.340740	-1.455222
C	-4.199615	-0.097998	0.281357
H	-4.158762	-0.386563	1.347950
C	-5.382878	-0.856051	-0.358014
H	-6.332997	-0.559163	0.109182
H	-5.454697	-0.622151	-1.432021
H	-5.288677	-1.944530	-0.259699
C	-4.499638	1.410995	0.218847
H	-5.493473	1.612157	0.645245
H	-3.776567	2.016255	0.778373
H	-4.511802	1.771713	-0.821174
C	-1.489908	0.237815	1.645118
H	-2.213489	0.989417	1.986614
H	-1.742167	-0.707399	2.145111
H	-0.504722	0.549086	2.010628
H	-0.498656	2.634509	1.059324
H	-0.480246	3.443719	-0.505022
C	1.656800	2.092954	-1.630354
H	1.194347	2.894267	-2.226736
H	2.744916	2.186134	-1.724651
H	1.373629	1.136386	-2.097138
H	3.035342	-0.380926	1.975264
H	4.705400	-0.183781	1.454757
C	4.422417	-0.766583	-1.204093
H	4.338396	-1.558761	-1.959804
H	4.207542	0.194516	-1.689963
H	5.462036	-0.737525	-0.842871
C	0.920495	-0.925435	0.155496

H	1.023958	-0.775009	1.232253
O	3.708085	-2.393657	0.463120
H	4.647857	-2.412302	0.713104

L G781.189049

C	-0.255061	-0.852137	-0.282325
C	2.210562	-1.319439	-0.419265
C	1.453104	1.804745	-0.349039
C	-1.335777	0.194714	0.160784
C	0.076485	2.398638	-0.151134
C	-1.122890	1.525999	-0.595578
H	-0.202022	-0.838083	-1.384301
H	2.016305	-1.782252	-1.390620
H	-2.011437	2.154479	-0.478681
H	-1.050376	1.310532	-1.673734
C	2.252736	1.567721	0.742528
H	1.807894	1.753494	1.725673
C	3.522494	-1.233270	0.017415
C	3.889479	-0.369435	1.176313
C	3.683827	1.152311	0.758587
H	4.212768	1.747785	1.516877
H	4.185823	1.326775	-0.200808
C	-0.853992	-2.240683	0.133348
H	-0.433879	-3.042049	-0.487052
H	-0.594438	-2.471432	1.176230
C	-2.644137	-0.541888	-0.324475
C	-2.381580	-2.049129	-0.036699
H	-2.900392	-2.359539	0.880943
H	-2.763472	-2.680184	-0.848678
H	-2.689230	-0.404547	-1.422386
C	-4.016449	-0.092608	0.247165
H	-4.006446	-0.295674	1.333482
C	-5.140127	-0.948520	-0.375746
H	-6.116130	-0.653727	0.031778
H	-5.179288	-0.804160	-1.466325
H	-5.011930	-2.020776	-0.182683
C	-4.356109	1.395138	0.045863
H	-5.388652	1.588714	0.365918
H	-3.712689	2.066302	0.628964
H	-4.283844	1.682169	-1.013925
C	-1.351032	0.396172	1.689670
H	-2.103894	1.140577	1.974747
H	-1.593154	-0.530508	2.225592
H	-0.389175	0.756880	2.076733
H	-0.052786	2.696335	0.898171
H	0.037041	3.327790	-0.744199
C	1.928261	1.660272	-1.770882
H	2.809628	1.019481	-1.874329
H	1.132638	1.261996	-2.413872
H	2.183853	2.654429	-2.170483
H	3.272660	-0.565646	2.064321

H	4.939670	-0.514069	1.455694
C	4.634424	-1.840177	-0.779282
H	4.285302	-2.354849	-1.681106
H	5.366701	-1.067791	-1.066995
H	5.190778	-2.556968	-0.153862
C	1.112603	-0.768759	0.272160
H	1.201240	-0.583472	1.342463

L-M-TS G781.187916, T-266

C	-0.140469	-0.652406	-0.250425
C	2.292160	-1.205232	-0.397967
C	1.256392	1.896851	-0.371812
C	-1.334716	0.250906	0.209144
C	-0.063068	2.529381	-0.109270
C	-1.267983	1.614487	-0.504462
H	-0.064368	-0.539202	-1.347440
H	1.983466	-1.762083	-1.285600
H	-2.161447	2.210320	-0.292316
H	-1.251322	1.449076	-1.592431
C	1.950486	1.246989	0.677989
H	1.519698	1.450994	1.662795
C	3.597515	-1.209836	-0.010556
C	3.998901	-0.260279	1.089332
C	3.474484	1.151521	0.713797
H	3.825916	1.890307	1.448845
H	3.906573	1.434454	-0.254767
C	-0.606184	-2.104777	0.040352
H	-0.123800	-2.824940	-0.632896
H	-0.316099	-2.392744	1.062189
C	-2.558724	-0.589897	-0.329576
C	-2.144836	-2.072787	-0.108018
H	-2.623614	-2.469228	0.798556
H	-2.479824	-2.702411	-0.941279
H	-2.602939	-0.408596	-1.421380
C	-3.975187	-0.303363	0.235135
H	-3.960201	-0.545142	1.313377
C	-5.000398	-1.241759	-0.436592
H	-6.006300	-1.059109	-0.036096
H	-5.038306	-1.062795	-1.522118
H	-4.769200	-2.302076	-0.278057
C	-4.455748	1.151000	0.080186
H	-5.508225	1.233463	0.382585
H	-3.893044	1.858554	0.703324
H	-4.390539	1.484059	-0.966751
C	-1.396735	0.395087	1.743525
H	-2.239254	1.027968	2.046498
H	-1.527495	-0.576500	2.236834
H	-0.490708	0.850379	2.165586
H	-0.151792	2.812882	0.946699
H	-0.138436	3.441067	-0.721897
C	1.784890	1.878705	-1.763975

H	2.334586	0.950084	-1.978525
H	0.995475	2.039050	-2.506906
H	2.512948	2.701299	-1.874804
H	3.567191	-0.566025	2.057316
H	5.088402	-0.233119	1.216961
C	4.652316	-2.013428	-0.708378
H	4.246510	-2.617907	-1.528018
H	5.440617	-1.355173	-1.108035
H	5.151969	-2.684890	0.007685
C	1.255587	-0.470012	0.338692
H	1.251455	-0.678927	1.417337

**M**	G781.189600		
C	-0.121693	-0.640141	-0.235674
C	2.309713	-1.196559	-0.394783
C	1.220990	1.898948	-0.382701
C	-1.327123	0.252315	0.219144
C	-0.073907	2.556861	-0.120848
C	-1.279824	1.613903	-0.496630
H	-0.042274	-0.514821	-1.332239
H	1.982144	-1.789182	-1.251502
H	-2.170531	2.210476	-0.274174
H	-1.268159	1.449947	-1.584225
C	1.871008	1.156873	0.666355
H	1.438050	1.406671	1.641010
C	3.617013	-1.185738	-0.026200
C	4.011100	-0.226174	1.070913
C	3.413012	1.161617	0.737921
H	3.706853	1.901594	1.495504
H	3.842366	1.495366	-0.215725
C	-0.579636	-2.093851	0.037789
H	-0.093247	-2.808487	-0.638680
H	-0.288703	-2.388270	1.057899
C	-2.543860	-0.596952	-0.329007
C	-2.117611	-2.076736	-0.111963
H	-2.595145	-2.479915	0.792426
H	-2.449208	-2.705249	-0.947421
H	-2.584262	-0.412122	-1.420361
C	-3.964836	-0.323039	0.229725
H	-3.953257	-0.566964	1.307449
C	-4.979335	-1.268180	-0.449020
H	-5.988634	-1.093941	-0.053387
H	-5.012933	-1.087807	-1.534448
H	-4.740437	-2.326732	-0.290754
C	-4.455901	1.127786	0.074856
H	-5.510429	1.202556	0.372034
H	-3.902021	1.838511	0.702688
H	-4.388083	1.463047	-0.971298
C	-1.402464	0.395432	1.752773
H	-2.257961	1.012740	2.051411
H	-1.519005	-0.579339	2.243154

H	-0.506871	0.864655	2.181593
H	-0.162050	2.847048	0.933077
H	-0.168988	3.450867	-0.754396
C	1.767136	1.880855	-1.761059
H	2.283798	0.931897	-1.976809
H	1.004085	2.099582	-2.516663
H	2.540238	2.666763	-1.838276
H	3.627871	-0.569211	2.047426
H	5.102229	-0.149908	1.164320
C	4.672885	-2.002539	-0.708161
H	4.265947	-2.623380	-1.515133
H	5.460146	-1.352380	-1.122734
H	5.173543	-2.660797	0.019345
C	1.282564	-0.433126	0.361449
H	1.259540	-0.742548	1.419593

Cartesian coordinates of computed structures (Scheme 3 of main text, Table S19)

Gibbs energies (G"..." in Hartree) and imaginary frequencies of TS (T-"..." in cm^{-1}), wB97M-V/Def2-TZVPPD//B97D3/6-31G(d,p)-sp-density-fitting, 1 bar, 298.15 K.
The conversions from **24** to **25** and from **25** to **26** were calculated in the gas phase and in polar medium (water, wB97M-V/Def2-TZVPPD-CPCM(water)//B97D3/6-31G(d,p)).

**19**	G780.827844		
C	-1.330596	2.055123	0.103432
C	0.903452	-0.194373	-1.532132
C	0.394675	-0.625150	-0.357545
C	-0.878597	-1.332964	-0.171397
C	-1.983363	-0.990984	-0.863656
C	-2.376299	1.457396	0.705903
H	0.453909	-0.407309	-2.504250
H	-0.948864	-2.053822	0.649684
H	-1.870207	-0.204260	-1.613593
H	-2.227633	1.106839	1.734017
C	-3.737824	1.101375	0.154885
H	-4.506340	1.699556	0.677195
H	-3.828498	1.353059	-0.912502
C	-4.083236	-0.395596	0.379040
H	-3.786736	-0.665711	1.407517
H	-5.175099	-0.537317	0.322487
C	-3.407282	-1.413521	-0.593301
H	-3.952753	-1.349838	-1.553105
C	-3.558899	-2.844931	-0.054000
H	-4.618977	-3.100964	0.086785
H	-3.113410	-3.576502	-0.741649
H	-3.063723	-2.954681	0.921911
C	-1.364537	2.611862	-1.299985
H	-1.090971	3.680147	-1.299585
H	-0.625462	2.099674	-1.935660
H	-2.347047	2.525040	-1.779193
C	0.001753	2.227060	0.841171
H	0.763919	2.577558	0.129052
H	-0.107455	3.053543	1.566163
C	0.531607	1.001518	1.619846
H	1.251735	1.355521	2.377439
H	-0.299189	0.555984	2.188714
C	1.247785	-0.146112	0.828241
C	2.140961	0.646118	-1.362144
H	2.936081	0.386452	-2.078985
H	1.918235	1.712033	-1.550081
C	2.560695	0.399198	0.118713
H	2.833261	1.355797	0.596037
C	1.496404	-1.264732	1.867393
H	1.955625	-2.158472	1.430701
H	2.145566	-0.911282	2.681352
H	0.546450	-1.572063	2.326872
C	3.824037	-0.504620	0.216297

H	3.939106	-0.797265	1.273421
C	5.086229	0.295894	-0.159574
H	5.036897	0.657287	-1.197868
H	5.215663	1.171897	0.492604
H	5.987965	-0.327928	-0.073396
C	3.755268	-1.796530	-0.622130
H	2.832596	-2.363361	-0.452502
H	3.803396	-1.577333	-1.698207
H	4.608557	-2.448468	-0.385706

DA-TS G780.783105, T-383

C	-0.933020	1.701450	0.296171
C	0.513314	0.561329	-1.011525
C	0.365863	-0.639824	-0.301489
C	-0.817963	-1.392680	-0.284677
C	-1.963799	-0.819704	-0.856130
C	-2.125733	0.935250	0.373826
H	-0.022478	0.778531	-1.936542
H	-0.924807	-2.192557	0.453019
H	-1.799196	-0.174212	-1.722570
H	-2.239487	0.350909	1.293481
C	-3.456997	1.237606	-0.282799
H	-4.016426	2.024771	0.254831
H	-3.308615	1.613199	-1.307988
C	-4.282210	-0.068987	-0.296800
H	-4.629077	-0.266243	0.731145
H	-5.186670	0.032510	-0.915060
C	-3.403330	-1.292814	-0.756864
H	-3.729687	-1.578922	-1.773640
C	-3.609695	-2.506772	0.160606
H	-4.672848	-2.782762	0.207000
H	-3.044950	-3.380815	-0.192819
H	-3.279848	-2.283357	1.186468
C	-0.955059	3.008983	-0.475947
H	-1.467534	3.799242	0.101159
H	0.057050	3.377749	-0.692865
H	-1.488154	2.913757	-1.431698
C	0.004364	1.671796	1.517185
H	0.852501	2.350893	1.340031
H	-0.529346	2.110062	2.380657
C	0.541206	0.290211	1.961922
H	1.198836	0.445498	2.834476
H	-0.298636	-0.329901	2.318465
C	1.325426	-0.557655	0.891777
C	1.882997	1.165110	-0.780297
H	2.469657	1.156540	-1.713551
H	1.837506	2.224390	-0.484844
C	2.533375	0.274458	0.325968
H	2.936787	0.910067	1.134078
C	1.703271	-1.892851	1.550860
H	2.162618	-2.593498	0.843685

H	2.405858	-1.736633	2.382935
H	0.811312	-2.382068	1.967849
C	3.726947	-0.564086	-0.209433
H	4.040750	-1.238248	0.606081
C	4.931192	0.344548	-0.520943
H	4.697385	1.060651	-1.323121
H	5.237268	0.922796	0.363162
H	5.795067	-0.248530	-0.854972
C	3.388236	-1.438597	-1.431889
H	2.514744	-2.079579	-1.261448
H	3.167291	-0.823501	-2.315993
H	4.242811	-2.081364	-1.688545

**24**	G780.857560		
C	-0.711260	1.465837	0.241441
C	0.348659	0.785447	-0.699499
C	0.355687	-0.654029	-0.227504
C	-0.761569	-1.397329	-0.353644
C	-1.959522	-0.606063	-0.812615
C	-2.050120	0.635385	0.143394
H	0.016400	0.896572	-1.744630
H	-0.871959	-2.365779	0.140563
H	-1.782163	-0.204789	-1.828852
H	-2.252982	0.222045	1.149534
C	-3.360570	1.279174	-0.338226
H	-3.772241	2.029149	0.352082
H	-3.204419	1.778539	-1.307880
C	-4.303256	0.057440	-0.510031
H	-4.866419	-0.111912	0.421313
H	-5.048390	0.223471	-1.301319
C	-3.395479	-1.193461	-0.803972
H	-3.631901	-1.601333	-1.800089
C	-3.617435	-2.314694	0.222435
H	-4.676984	-2.608573	0.249176
H	-3.029475	-3.211708	-0.018476
H	-3.339026	-1.991824	1.237126
C	-0.928486	2.941623	-0.138919
H	-1.643126	3.430570	0.539091
H	0.013738	3.504886	-0.086373
H	-1.315131	3.039973	-1.162508
C	-0.083706	1.387594	1.672719
H	0.667904	2.188752	1.762335
H	-0.853744	1.622706	2.425360
C	0.583322	0.033784	2.041902
H	1.255860	0.191383	2.902873
H	-0.191744	-0.677897	2.367578
C	1.385467	-0.692858	0.884543
C	1.805115	1.291100	-0.516090
H	2.294018	1.383523	-1.495351
H	1.842228	2.292746	-0.068980
C	2.537764	0.237317	0.376855

H	2.999691	0.738065	1.246740
C	1.815458	-2.072585	1.394535
H	2.285932	-2.676106	0.609132
H	2.528077	-1.980612	2.227754
H	0.945314	-2.632499	1.765873
C	3.687742	-0.493784	-0.369519
H	4.076371	-1.267632	0.315136
C	4.853725	0.470568	-0.655128
H	4.547498	1.282035	-1.332220
H	5.228283	0.932240	0.270256
H	5.691304	-0.056357	-1.135268
C	3.244676	-1.199607	-1.665322
H	2.395601	-1.876542	-1.506080
H	2.938264	-0.473997	-2.432701
H	4.075956	-1.784237	-2.085324

24 G857.280179 (gas phase)
24 G857.291200 (in water, CPCM)

C	-0.841766	-1.666843	-0.027802
C	0.244640	-0.841556	-0.817369
C	0.296977	0.463711	-0.053231
C	-0.805062	1.246297	-0.006777
C	-2.022939	0.611395	-0.624551
C	-2.156021	-0.797746	0.051066
H	-0.092208	-0.723766	-1.859629
H	-0.901855	2.060892	0.715845
H	-1.847330	0.427222	-1.700575
H	-2.353903	-0.598509	1.121418
C	-3.482569	-1.284544	-0.554654
H	-3.920695	-2.149298	-0.036610
H	-3.335694	-1.573665	-1.607530
C	-4.385790	-0.024600	-0.464157
H	-4.944497	-0.035165	0.484951
H	-5.134190	0.000956	-1.268908
C	-3.438782	1.231177	-0.495976
H	-3.654060	1.840222	-1.388450
C	-3.630887	2.127699	0.736250
H	-4.679999	2.446051	0.821010
H	-3.012450	3.034927	0.680900
H	-3.371920	1.596945	1.665194
C	-1.100383	-3.026397	-0.701798
H	-1.835808	-3.618420	-0.138149
H	-0.177312	-3.619115	-0.762786
H	-1.481631	-2.901926	-1.724454
C	-0.220083	-1.900000	1.388648
H	0.517548	-2.715173	1.310563
H	-0.998173	-2.272042	2.074496
C	0.468604	-0.665200	2.030338
H	1.119740	-1.007211	2.852806
H	-0.292918	-0.006393	2.477066
C	1.320251	0.236682	1.043509

C	1.680654	-1.422075	-0.750259
H	2.180166	-1.278536	-1.717688
H	1.681708	-2.504654	-0.570129
C	2.431828	-0.648723	0.381754
H	2.790351	-1.366516	1.141303
C	1.810669	1.468844	1.810588
H	2.336326	2.175331	1.157348
H	2.495229	1.181398	2.621673
H	0.962933	1.997398	2.271193
C	3.696220	0.099751	-0.123578
H	4.095383	0.671204	0.732922
C	4.784041	-0.914196	-0.527142
H	4.459500	-1.530591	-1.379044
H	5.030253	-1.594200	0.301804
H	5.706987	-0.399259	-0.830510
C	3.443857	1.095487	-1.270272
H	2.711844	1.874313	-1.025136
H	3.087129	0.583731	-2.176039
H	4.382322	1.601494	-1.539556
H	0.554332	2.822462	-0.983560
O	1.030982	3.673463	-0.979578
H	1.114280	3.857217	-0.032638

24-X-TS G857.164870, T-1524 (gas phase)
24-X-TS G857.187655, T-1524 (in water, CPCM)

C	-0.859255	-1.648347	0.100075
C	0.268343	-0.923069	-0.771557
C	0.328261	0.433012	-0.172198
C	-0.812224	1.355508	-0.304361
C	-2.044167	0.548288	-0.734159
C	-2.153478	-0.744200	0.114392
H	-0.074667	-0.919777	-1.817736
H	-0.964654	1.896519	0.641604
H	-1.899361	0.233520	-1.783563
H	-2.310245	-0.414623	1.160157
C	-3.502248	-1.306825	-0.366722
H	-3.916059	-2.090746	0.283096
H	-3.392125	-1.741403	-1.372840
C	-4.405396	-0.046684	-0.422986
H	-4.937607	0.074825	0.533399
H	-5.175242	-0.131070	-1.202855
C	-3.458493	1.186325	-0.652527
H	-3.694736	1.669265	-1.614033
C	-3.616591	2.244354	0.450121
H	-4.659564	2.588859	0.501646
H	-2.981534	3.122411	0.269074
H	-3.357874	1.838286	1.440089
C	-1.126286	-3.056377	-0.456827
H	-1.875486	-3.585468	0.148533
H	-0.209236	-3.660789	-0.453918
H	-1.495902	-3.015667	-1.490066

C	-0.241068	-1.725567	1.518427
H	0.518976	-2.520905	1.549591
H	-1.017175	-2.019207	2.243660
C	0.373094	-0.391901	1.990854
H	1.027367	-0.561094	2.854829
H	-0.407610	0.312550	2.305095
C	1.332539	0.418282	0.902795
C	1.693266	-1.508974	-0.609254
H	2.201780	-1.504053	-1.581581
H	1.678457	-2.555430	-0.280330
C	2.432892	-0.584082	0.413101
H	2.769453	-1.178848	1.280646
C	1.755191	1.694286	1.577915
H	1.707474	2.599126	0.524799
H	2.652481	1.558644	2.197032
H	0.957018	2.128572	2.199221
C	3.699355	0.090862	-0.181161
H	4.067472	0.796393	0.581869
C	4.801474	-0.959858	-0.411167
H	4.492285	-1.709484	-1.155984
H	5.053724	-1.495399	0.516497
H	5.718729	-0.486379	-0.789820
C	3.430482	0.899464	-1.461876
H	2.643496	1.653225	-1.311722
H	3.137387	0.248311	-2.299886
H	4.349529	1.418297	-1.772899
H	-0.490119	2.169753	-0.999329
O	1.304776	3.209643	-0.466891
H	2.071215	3.757650	-0.706457

X G857.166542 (gas phase)
X G857.184350 (in water, CPCM)

C	-0.905950	-1.659587	0.103071
C	0.241627	-0.958141	-0.748307
C	0.344268	0.408665	-0.170642
C	-0.791996	1.358782	-0.283628
C	-2.034637	0.572232	-0.723863
C	-2.181038	-0.727551	0.104770
H	-0.080836	-0.954187	-1.802500
H	-0.943476	1.864688	0.685482
H	-1.882352	0.265870	-1.775046
H	-2.352428	-0.409762	1.152177
C	-3.530726	-1.259143	-0.409226
H	-3.970516	-2.043580	0.222565
H	-3.407936	-1.683781	-1.418211
C	-4.411593	0.016526	-0.467630
H	-4.965572	0.132759	0.477017
H	-5.163188	-0.042639	-1.267490
C	-3.438107	1.235453	-0.655120
H	-3.647945	1.742549	-1.610492
C	-3.596587	2.273512	0.466422

H	-4.633897	2.636219	0.507804
H	-2.943173	3.143835	0.314833
H	-3.361948	1.842616	1.451841
C	-1.198550	-3.067527	-0.440220
H	-1.970811	-3.574917	0.154710
H	-0.295717	-3.693005	-0.414704
H	-1.547053	-3.029806	-1.481257
C	-0.304284	-1.728582	1.524040
H	0.438097	-2.538995	1.580495
H	-1.090234	-1.983997	2.253727
C	0.330833	-0.399326	1.980309
H	0.978851	-0.575913	2.845292
H	-0.427170	0.334336	2.277566
C	1.364209	0.401876	0.885877
C	1.655330	-1.574810	-0.575445
H	2.161911	-1.604517	-1.548682
H	1.618350	-2.613349	-0.223456
C	2.424112	-0.647729	0.422448
H	2.758889	-1.228310	1.300232
C	1.808435	1.589128	1.632683
H	1.851035	2.893419	0.333136
H	2.554481	1.375536	2.405669
H	1.005360	2.233955	2.012800
C	3.689481	0.006094	-0.194837
H	4.078524	0.703497	0.564683
C	4.771048	-1.054306	-0.466928
H	4.436090	-1.789519	-1.214844
H	5.035879	-1.606095	0.447380
H	5.686719	-0.587775	-0.858227
C	3.388600	0.828640	-1.458292
H	2.615025	1.586990	-1.277553
H	3.057640	0.190557	-2.291406
H	4.298656	1.346457	-1.796070
H	-0.515361	2.183310	-0.971062
O	1.559529	3.498962	-0.424455
H	2.401026	3.806636	-0.793670

**X**	G857.166520 (gas phase)		
**X**	G857.184328 (in water, CPCM)		
C	-0.906002	-1.659697	0.103169
C	0.241696	-0.958354	-0.748241
C	0.344307	0.408454	-0.170695
C	-0.791889	1.358639	-0.283617
C	-2.034523	0.572163	-0.723901
C	-2.180984	-0.727551	0.104855
H	-0.080637	-0.954473	-1.802472
H	-0.943324	1.864248	0.685675
H	-1.882057	0.265604	-1.775004
H	-2.352347	-0.409725	1.152250
C	-3.530787	-1.259003	-0.409095
H	-3.970541	-2.043574	0.222547

H	-3.408067	-1.683385	-1.418206
C	-4.411495	0.016716	-0.467038
H	-4.964518	0.133146	0.478149
H	-5.163884	-0.042404	-1.266151
C	-3.437964	1.235459	-0.655447
H	-3.647876	1.741900	-1.611160
C	-3.596271	2.274289	0.465396
H	-4.633561	2.637088	0.506668
H	-2.942801	3.144460	0.313133
H	-3.361526	1.844038	1.451058
C	-1.198715	-3.067597	-0.440082
H	-1.970910	-3.574935	0.154965
H	-0.295927	-3.693126	-0.414678
H	-1.547374	-3.029912	-1.481067
C	-0.304301	-1.728743	1.524128
H	0.437897	-2.539325	1.580616
H	-1.090247	-1.983825	2.253920
C	0.331130	-0.399491	1.980224
H	0.979183	-0.576298	2.845166
H	-0.426894	0.334018	2.277846
C	1.364200	0.401667	0.886014
C	1.655351	-1.575118	-0.575222
H	2.161951	-1.605269	-1.548414
H	1.618209	-2.613519	-0.222835
C	2.424181	-0.647783	0.422431
H	2.759284	-1.228268	1.300144
C	1.808381	1.589019	1.632665
H	1.850919	2.893196	0.334569
H	2.554126	1.375532	2.405964
H	1.005251	2.233983	2.012421
C	3.689234	0.006301	-0.195179
H	4.078298	0.703769	0.564280
C	4.770954	-1.053836	-0.467637
H	4.435909	-1.789112	-1.215452
H	5.036193	-1.605577	0.446563
H	5.686410	-0.587114	-0.859227
C	3.387758	0.828878	-1.458470
H	2.613832	1.586781	-1.277398
H	3.056925	0.190743	-2.291599
H	4.297492	1.347195	-1.796361
H	-0.515208	2.183385	-0.970748
O	1.559831	3.498709	-0.423400
H	2.401592	3.804607	-0.793510

X-25-TS G857.170573, T-209 (gas phase)
X-25-TS G857.188489, T-209 (in water, CPCM)

C	-0.920784	-1.666010	0.103559
C	0.242218	-0.953844	-0.709580
C	0.337493	0.403982	-0.106334
C	-0.793241	1.360122	-0.229215
C	-2.028806	0.584104	-0.705113

C	-2.195075	-0.731618	0.094054
H	-0.060033	-0.919882	-1.769541
H	-0.962018	1.858264	0.740886
H	-1.857325	0.297558	-1.758941
H	-2.389961	-0.434846	1.143504
C	-3.533052	-1.251124	-0.461169
H	-3.988112	-2.047051	0.144840
H	-3.387081	-1.656306	-1.475109
C	-4.411169	0.026862	-0.514878
H	-4.989190	0.123818	0.417438
H	-5.141968	-0.014414	-1.334857
C	-3.432229	1.248253	-0.651751
H	-3.620139	1.778299	-1.599159
C	-3.613695	2.259718	0.490358
H	-4.651700	2.621599	0.519421
H	-2.957793	3.133359	0.372189
H	-3.398730	1.805867	1.469972
C	-1.205807	-3.069236	-0.453987
H	-1.989141	-3.579977	0.123223
H	-0.303980	-3.695771	-0.416925
H	-1.535795	-3.024020	-1.500703
C	-0.340800	-1.739132	1.534262
H	0.395246	-2.553821	1.603993
H	-1.138233	-1.985135	2.255038
C	0.298644	-0.414254	1.984670
H	0.979904	-0.585643	2.823439
H	-0.438716	0.334955	2.290937
C	1.405452	0.434960	0.892315
C	1.655188	-1.575175	-0.534154
H	2.153293	-1.633308	-1.510289
H	1.613563	-2.604449	-0.156525
C	2.443253	-0.633231	0.434941
H	2.787676	-1.198620	1.318888
C	1.840155	1.609496	1.628261
H	1.862266	2.898000	0.293083
H	2.611750	1.417411	2.380575
H	1.042192	2.258924	2.008463
C	3.704010	0.001753	-0.212524
H	4.105477	0.711614	0.528733
C	4.776669	-1.069209	-0.478447
H	4.429366	-1.815648	-1.209441
H	5.049503	-1.606264	0.442264
H	5.689898	-0.614356	-0.888717
C	3.389945	0.801844	-1.487440
H	2.632733	1.576606	-1.307522
H	3.032313	0.151291	-2.299801
H	4.301060	1.297238	-1.854552
H	-0.492535	2.187015	-0.903637
O	1.563354	3.491238	-0.470325
H	2.399176	3.818218	-0.835530

25 G857.290907 (gas phase)
25 G857.301492 (in water, CPCM)

C	-1.003685	-1.740254	0.096586
C	0.301124	-0.948886	-0.177224
C	0.263177	0.137834	0.933909
C	-0.748330	1.270709	0.585136
C	-1.790566	0.716012	-0.398952
C	-2.213089	-0.743495	-0.121584
H	0.289423	-0.500492	-1.182264
H	-1.222569	1.640059	1.506927
H	-1.302427	0.703282	-1.390509
H	-2.805097	-0.745034	0.813821
C	-3.219884	-0.969707	-1.263210
H	-3.853958	-1.856829	-1.126701
H	-2.683852	-1.095883	-2.218072
C	-4.041989	0.350174	-1.274065
H	-4.961457	0.227755	-0.680546
H	-4.358919	0.622581	-2.290940
C	-3.134999	1.457177	-0.621843
H	-2.983516	2.288626	-1.328970
C	-3.762547	2.040190	0.653542
H	-4.760576	2.449443	0.438689
H	-3.152802	2.852326	1.073648
H	-3.883525	1.272326	1.432798
C	-1.135309	-3.040244	-0.705083
H	-2.056577	-3.581612	-0.445150
H	-0.287100	-3.708858	-0.498735
H	-1.154677	-2.850236	-1.787194
C	-0.884202	-1.990419	1.624898
H	-0.247184	-2.866220	1.821394
H	-1.862240	-2.206905	2.078458
C	-0.229984	-0.700223	2.186262
H	0.614352	-0.940960	2.846199
H	-0.930821	-0.099215	2.782050
C	1.711615	0.547838	1.124119
C	1.664700	-1.644379	0.020650
H	2.023399	-2.125996	-0.898831
H	1.602059	-2.436583	0.781020
C	2.629705	-0.520993	0.515198
H	3.287907	-0.915769	1.308471
C	2.142779	1.646011	1.772297
H	1.893510	2.980299	0.005458
H	3.204951	1.845358	1.927403
H	1.441588	2.358143	2.211702
C	3.578219	0.049296	-0.582579
H	4.078460	0.923920	-0.128897
C	4.671362	-0.966819	-0.954688
H	4.239039	-1.870411	-1.409667
H	5.246091	-1.280137	-0.071165
H	5.373784	-0.538275	-1.684209
C	2.837307	0.539576	-1.838620

H	2.063179	1.279441	-1.598145
H	2.357475	-0.293713	-2.371818
H	3.545223	1.003835	-2.541254
H	-0.236389	2.126840	0.123695
O	1.659819	3.610650	-0.702326
H	2.392277	3.517295	-1.328567

25 G857.291689 (gas phase)
25 G857.301951 (in water, CPCM)

C	-0.958020	-0.900203	1.302324
C	0.222035	-0.799951	0.301209
C	0.262704	0.723741	-0.004199
C	-0.889900	1.145611	-0.963140
C	-2.012760	0.100428	-0.884610
C	-2.265550	-0.441952	0.539242
H	0.027540	-1.391528	-0.606939
H	-1.255075	2.147098	-0.689048
H	-1.672881	-0.767406	-1.478747
H	-2.712845	0.379420	1.131314
C	-3.404598	-1.439597	0.264201
H	-3.935835	-1.766061	1.168918
H	-3.003492	-2.343658	-0.222325
C	-4.327409	-0.660954	-0.715765
H	-5.140210	-0.168118	-0.159914
H	-4.806771	-1.332486	-1.442350
C	-3.432966	0.425730	-1.417689
H	-3.457729	0.287707	-2.511115
C	-3.917219	1.852732	-1.118061
H	-4.966450	1.975368	-1.424027
H	-3.323833	2.607235	-1.653759
H	-3.859882	2.081586	-0.043147
C	-1.099041	-2.268904	1.978529
H	-1.922165	-2.274565	2.707875
H	-0.176221	-2.529626	2.516088
H	-1.295665	-3.065532	1.247587
C	-0.589777	0.226178	2.307597
H	0.126773	-0.146378	3.054750
H	-1.471055	0.575079	2.865254
C	0.058827	1.339186	1.441377
H	1.022324	1.651237	1.865227
H	-0.568634	2.239741	1.376254
C	1.675160	0.970764	-0.498463
C	1.651392	-1.123037	0.785722
H	1.910356	-2.178748	0.630446
H	1.761039	-0.931890	1.862487
C	2.591117	-0.161359	-0.010352
H	3.341408	0.264173	0.676790
C	2.074188	2.013220	-1.251076
H	3.107242	2.125432	-1.584544
H	1.368971	2.776373	-1.584659
C	3.379715	-0.834128	-1.171585

H	3.921989	-0.021560	-1.686702
C	4.434363	-1.817618	-0.637230
H	3.967043	-2.666872	-0.116959
H	5.116105	-1.325995	0.071538
H	5.036280	-2.230898	-1.459604
C	2.465791	-1.506875	-2.208920
H	1.726732	-0.797934	-2.605950
H	1.923253	-2.358753	-1.772739
H	3.055139	-1.893375	-3.052530
H	-0.521630	1.216715	-1.998278
O	3.214398	2.832040	1.721978
H	2.603812	3.496161	2.074193
H	2.850874	2.653663	0.834126

Y-TS G857.206228, T-1855 (gas phase)
Y-TS G857.217227, T-1855 (in water, CPCM)

C	1.029463	1.437840	0.594608
C	-0.096312	0.829683	-0.276558
C	-0.125019	-0.643272	0.223883
C	1.032227	-1.473641	-0.403894
C	2.179737	-0.510650	-0.758588
C	2.363114	0.659533	0.238819
H	0.152027	0.878912	-1.349447
H	1.355928	-2.250528	0.304217
H	1.903538	-0.037690	-1.718422
H	2.749485	0.232634	1.184218
C	3.546655	1.398063	-0.410560
H	4.031866	2.126031	0.254153
H	3.203258	1.948885	-1.301353
C	4.502758	0.243794	-0.822626
H	5.262053	0.084305	-0.041353
H	5.048710	0.478905	-1.747450
C	3.619634	-1.048813	-0.971352
H	3.714230	-1.456370	-1.991252
C	4.042744	-2.151358	0.011524
H	5.105325	-2.399968	-0.124768
H	3.464182	-3.074577	-0.133676
H	3.911718	-1.833590	1.056958
C	1.163107	2.961074	0.494466
H	1.949308	3.341645	1.162533
H	0.219908	3.448914	0.779375
H	1.408728	3.282131	-0.527392
C	0.588265	0.963576	2.004398
H	-0.223777	1.600255	2.386549
H	1.411018	1.026066	2.731762
C	0.084089	-0.487247	1.791461
H	-0.846139	-0.673916	2.340826
H	0.816070	-1.228408	2.140968
C	-1.527950	-1.103431	-0.103055
C	-1.556942	1.325579	-0.126003
H	-1.827234	2.027072	-0.928905

H	-1.719836	1.866564	0.820033
C	-2.401040	0.033075	-0.158668
H	-2.840191	-0.304746	1.029348
C	-2.018865	-2.418328	0.133080
H	-2.878538	-2.748651	-0.460739
H	-1.279049	-3.206266	0.294759
C	-3.720209	0.014516	-0.948132
H	-4.212292	-0.952405	-0.744418
C	-4.674043	1.122760	-0.468302
H	-4.247700	2.119808	-0.651312
H	-4.868884	1.036620	0.609873
H	-5.635469	1.072051	-0.999132
C	-3.468362	0.099571	-2.465502
H	-2.826954	-0.727454	-2.800670
H	-2.968094	1.042701	-2.731081
H	-4.412717	0.056687	-3.028218
H	0.691199	-1.997303	-1.311094
O	-3.266842	-1.126563	2.104276
H	-4.213866	-1.268287	1.940861
H	-2.641376	-2.011475	1.246057

26 G857.298892 (gas phase)
26 G857.309076 (in water, CPCM)

C	0.995124	1.477577	0.428911
C	-0.070099	0.773133	-0.441463
C	-0.104187	-0.653989	0.182149
C	1.069463	-1.501550	-0.388460
C	2.236225	-0.547240	-0.724440
C	2.355222	0.697581	0.193756
H	0.241276	0.726616	-1.496980
H	1.364933	-2.271055	0.340798
H	2.020712	-0.143043	-1.730097
H	2.703771	0.350172	1.184922
C	3.558973	1.404603	-0.452639
H	4.002935	2.186476	0.179000
H	3.252818	1.883907	-1.396794
C	4.548469	0.237744	-0.732802
H	5.278404	0.154443	0.087341
H	5.127439	0.408728	-1.651678
C	3.690962	-1.078400	-0.813450
H	3.846031	-1.571383	-1.787389
C	4.074148	-2.084106	0.283110
H	5.145639	-2.325534	0.225976
H	3.515802	-3.026192	0.188584
H	3.882148	-1.679459	1.288269
C	1.104153	2.990382	0.214555
H	1.853502	3.439941	0.882226
H	0.140027	3.477018	0.422003
H	1.386180	3.236879	-0.818700
C	0.502574	1.100167	1.849705
H	-0.378130	1.701245	2.124082

H	1.269201	1.290234	2.615793
C	0.125311	-0.397062	1.728447
H	-0.763741	-0.640309	2.324807
H	0.938540	-1.036221	2.101341
C	-1.521991	-1.125658	-0.125075
C	-1.544079	1.238170	-0.440763
H	-1.779937	1.893691	-1.293825
H	-1.815017	1.812565	0.463110
C	-2.317769	-0.081105	-0.473621
H	-2.821488	-0.418425	1.727457
C	-1.911209	-2.568634	0.035761
H	-2.957401	-2.763660	-0.232603
H	-1.279699	-3.219208	-0.588878
C	-3.777180	-0.123309	-0.887284
H	-4.196513	-1.099812	-0.588911
C	-4.616981	0.971496	-0.196799
H	-4.259500	1.975220	-0.468869
H	-4.569923	0.873666	0.895603
H	-5.669114	0.900407	-0.507807
C	-3.893950	-0.022321	-2.425299
H	-3.334959	-0.829894	-2.917429
H	-3.491007	0.935122	-2.786901
H	-4.945483	-0.081761	-2.742127
H	0.764400	-2.034757	-1.303839
O	-3.254228	-0.511087	2.597241
H	-3.700706	-1.367126	2.519833
H	-1.756383	-2.894669	1.077958

Cartesian coordinates of computed structures (Scheme 3 of main text, Table S23)

Gibbs energies (G"..." in Hartree) and imaginary frequencies of TS (T-"..." in cm^{-1}), wB97M-V/Def2-TZVPPD//B97D3/6-31G(d,p)-sp-density-fitting, 1 bar, 298.15 K. From the structures labeled with asterisks the energy of NH_3 (G56.537076, page 264) is subtracted for direct comparability.

**N**	G781.127266		
C	0.901270	-2.932514	-0.197305
C	2.154404	-2.383676	-0.907654
C	1.929237	-0.944922	-1.311667
C	-0.417872	2.522105	0.518325
C	1.384026	-1.480688	1.827294
C	0.432192	-2.157075	1.000666
H	2.330951	-3.011220	-1.796450
H	3.043205	-2.504703	-0.275120
H	1.071563	-3.969667	0.161035
H	0.065494	-3.014607	-0.909237
C	-1.006961	-2.121573	1.243276
H	-1.319218	-1.875163	2.261783
H	-1.510346	-3.029987	0.886978
H	-1.426842	-1.305885	0.571719
C	1.070694	-0.782912	2.959903
H	2.428884	-1.523073	1.524365
C	2.705320	0.132165	-1.046036
H	1.025454	-0.774614	-1.909177
C	2.296404	1.505227	-1.542519
H	3.117757	1.918613	-2.152619
H	1.415295	1.421472	-2.195578
C	3.995173	0.078265	-0.260397
H	4.352976	-0.938867	-0.065609
H	3.889345	0.594165	0.707837
H	4.793109	0.609086	-0.800311
C	0.844433	2.046538	0.456470
C	1.978743	2.522952	-0.407946
H	1.762327	3.495422	-0.868841
H	2.880383	2.668359	0.209650
C	-1.438610	1.859265	1.427602
H	-1.790997	2.584166	2.180595
H	-0.956480	1.037136	1.982135
C	-2.680237	1.308620	0.681936
H	-3.251300	2.156525	0.262402
H	-3.354337	0.830391	1.407273
C	-2.322320	0.371413	-0.440330
C	-3.073849	-0.652847	-0.939829
H	-1.370326	0.583272	-0.940888
C	-2.581911	-1.461058	-2.116250
H	-3.273934	-1.360843	-2.967214
H	-2.557881	-2.536315	-1.874642
H	-1.584454	-1.149475	-2.451624

C	-4.424971	-1.048398	-0.404099
H	-5.191153	-0.911412	-1.183613
H	-4.736115	-0.475926	0.475458
H	-4.442722	-2.120352	-0.149487
H	0.055168	-0.694564	3.342473
H	1.850869	-0.283201	3.533160
H	1.071511	1.172990	1.077019
C	-0.933081	3.688665	-0.294681
H	-0.134816	4.257056	-0.782726
H	-1.623745	3.350980	-1.083698
H	-1.502127	4.382932	0.342398

N-O-TS G781.123651, T-37

C	1.514749	-2.784405	0.466641
C	2.630475	-2.220309	-0.442546
C	2.116515	-0.985515	-1.148367
C	-0.960075	2.448487	0.395355
C	1.653829	-0.731020	1.940137
C	0.865363	-1.763805	1.360206
H	2.903430	-3.005026	-1.165792
H	3.537001	-2.014972	0.140473
H	1.905472	-3.587218	1.124990
H	0.733510	-3.263576	-0.142143
C	-0.589092	-1.879067	1.558476
H	-0.975478	-1.395860	2.460429
H	-0.915088	-2.927327	1.519233
H	-1.092634	-1.392829	0.680058
C	1.145316	0.331274	2.643084
H	2.726600	-0.755096	1.753414
C	2.637337	0.264694	-1.123416
H	1.197705	-1.136743	-1.727627
C	1.921020	1.408486	-1.814042
H	2.635065	1.944760	-2.459774
H	1.123929	1.019707	-2.464923
C	3.913061	0.640647	-0.407607
H	4.476023	-0.217190	-0.024457
H	3.707850	1.318249	0.437402
H	4.579122	1.194894	-1.085183
C	0.179641	1.835970	-0.022289
C	1.305552	2.437133	-0.822058
H	0.959399	3.310807	-1.391720
H	2.100303	2.813814	-0.154931
C	-2.057448	1.652801	1.089205
H	-2.595609	2.304187	1.794615
H	-1.626782	0.832113	1.678960
C	-3.084634	1.038012	0.097907
H	-3.509584	1.845375	-0.523714
H	-3.921782	0.623093	0.674303
C	-2.472790	0.008376	-0.816549
C	-2.801394	-1.305426	-0.940174
H	-1.686073	0.385667	-1.477174

C	-2.096445	-2.176751	-1.956351
H	-2.813545	-2.564462	-2.696962
H	-1.649187	-3.063616	-1.477705
H	-1.310491	-1.634955	-2.498185
C	-3.889986	-1.998592	-0.156410
H	-4.699496	-2.316159	-0.832230
H	-4.332564	-1.378195	0.629685
H	-3.505677	-2.920443	0.308966
H	0.087920	0.442328	2.871856
H	1.806079	1.114246	3.012603
H	0.268889	0.764758	0.164108
C	-1.266622	3.907263	0.175292
H	-0.551393	4.416443	-0.479049
H	-2.274374	4.034246	-0.249289
H	-1.274781	4.439461	1.140821

**O**	G781.161420		
C	-3.051711	0.488154	-1.422507
C	-3.184708	-0.960575	-0.884969
C	-1.840047	-1.596802	-0.629156
C	0.825627	1.460160	1.105611
C	-1.711968	1.154650	0.593746
C	-2.207448	1.474363	-0.623834
H	-3.751526	-1.535946	-1.635904
H	-3.794300	-0.958100	0.028413
H	-4.063073	0.905847	-1.568458
H	-2.618363	0.447340	-2.439749
C	-1.982560	2.796786	-1.320940
H	-1.428097	3.517361	-0.709713
H	-2.950794	3.253788	-1.579199
H	-1.446391	2.667666	-2.275605
C	-0.676968	1.859373	1.423570
H	-2.021432	0.200863	1.012154
C	-1.406204	-2.168036	0.518102
H	-1.115178	-1.476561	-1.444499
C	0.074327	-2.409102	0.754543
H	0.233906	-3.314253	1.360178
H	0.586371	-2.569618	-0.209840
C	-2.286940	-2.459633	1.710249
H	-3.347744	-2.262776	1.527872
H	-1.980674	-1.875146	2.594257
H	-2.190053	-3.518459	1.996161
C	0.939560	0.003284	0.549436
C	0.721752	-1.191780	1.478835
H	1.665562	-1.484613	1.964837
H	0.058905	-0.878325	2.299778
C	1.371617	2.273628	-0.085146
H	1.603308	3.312469	0.186774
H	0.612724	2.301940	-0.878619
C	2.614692	1.513634	-0.579546
H	3.535010	1.869209	-0.098521

H	2.768796	1.618221	-1.661110
C	2.417421	0.022297	-0.136984
C	2.402426	-0.971936	-1.193447
H	3.133585	-0.265629	0.644692
C	3.145692	-2.244906	-1.021314
H	4.197460	-2.025361	-1.296531
H	2.798458	-3.054332	-1.672323
H	3.177981	-2.565367	0.027685
C	1.716129	-0.725198	-2.486476
H	2.493661	-0.620437	-3.266776
H	1.096532	0.176014	-2.494290
H	1.125070	-1.601205	-2.789462
H	-0.726164	2.954547	1.324949
H	-0.866489	1.631189	2.482975
H	0.201749	-0.057874	-0.264618
C	1.676038	1.677601	2.371088
H	1.354631	1.017159	3.187314
H	2.747271	1.501695	2.200667
H	1.568932	2.712923	2.721345

**O**	G781.161423		
C	-3.051843	0.487778	-1.422362
C	-3.184603	-0.960991	-0.884912
C	-1.839831	-1.597045	-0.629192
C	0.825469	1.460355	1.105483
C	-1.712088	1.154379	0.593859
C	-2.207734	1.474092	-0.623639
H	-3.751396	-1.536397	-1.635836
H	-3.794135	-0.958632	0.028516
H	-4.063267	0.905331	-1.568301
H	-2.618474	0.447123	-2.439604
C	-1.983235	2.796651	-1.320617
H	-1.428673	3.517202	-0.709444
H	-2.951624	3.253567	-1.578456
H	-1.447363	2.667766	-2.275471
C	-0.677134	1.859215	1.423674
H	-2.021353	0.200512	1.012226
C	-1.405857	-2.168231	0.518037
H	-1.115032	-1.476704	-1.444580
C	0.074730	-2.409067	0.754385
H	0.234511	-3.314182	1.360024
H	0.586697	-2.569517	-0.210048
C	-2.286497	-2.459888	1.710244
H	-3.347441	-2.264071	1.527561
H	-1.980911	-1.874545	2.593932
H	-2.188716	-3.518435	1.996860
C	0.939648	0.003469	0.549176
C	0.722051	-1.191645	1.478596
H	1.665947	-1.484340	1.964529
H	0.059231	-0.878270	2.299592
C	1.371155	2.273970	-0.085321

H	1.602787	3.312805	0.186643
H	0.612072	2.302250	-0.878614
C	2.614176	1.514124	-0.580151
H	3.534676	1.869998	-0.099686
H	2.767682	1.618573	-1.661807
C	2.417405	0.022890	-0.137202
C	2.402570	-0.971769	-1.193289
H	3.133637	-0.264581	0.644569
C	3.145860	-2.244624	-1.020399
H	4.197615	-2.025139	-1.295735
H	2.798709	-3.054428	-1.670984
H	3.178120	-2.564501	0.028774
C	1.716478	-0.725621	-2.486524
H	2.494233	-0.620425	-3.266549
H	1.096450	0.175301	-2.494627
H	1.125969	-1.601942	-2.789631
H	-0.726572	2.954398	1.325242
H	-0.866505	1.630813	2.483062
H	0.201727	-0.057717	-0.264787
C	1.676023	1.677875	2.370845
H	1.354916	1.017269	3.187057
H	2.747277	1.502269	2.200228
H	1.568745	2.713144	2.721204

O-P-TS G781.150348, T-747

C	3.273366	-0.502263	-1.479142
C	3.351537	1.010601	-1.142443
C	1.985429	1.627299	-0.965068
C	-0.558589	-1.344962	1.125956
C	1.931255	-0.953363	0.594744
C	2.475660	-1.413463	-0.553040
H	3.895914	1.498577	-1.967889
H	3.962108	1.150762	-0.240294
H	4.302114	-0.891477	-1.572887
H	2.838988	-0.616144	-2.490524
C	2.359757	-2.837882	-1.049404
H	1.852628	-3.501125	-0.339538
H	3.362963	-3.253253	-1.233473
H	1.829228	-2.892376	-2.014340
C	0.939598	-1.600815	1.523672
H	2.159271	0.072228	0.864101
C	1.522540	2.284759	0.120504
H	1.269775	1.401329	-1.766942
C	0.030050	2.484665	0.307996
H	-0.178223	3.444593	0.806903
H	-0.463086	2.512119	-0.674472
C	2.378217	2.717229	1.289772
H	3.448927	2.565958	1.121587
H	2.108522	2.188347	2.219541
H	2.222613	3.788660	1.489886
C	-0.810518	0.025161	0.387096

C	-0.573499	1.328489	1.173243
H	-1.494872	1.647985	1.684598
H	0.129770	1.102668	1.985892
C	-1.001852	-2.342859	0.031386
H	-1.198583	-3.345028	0.432465
H	-0.205091	-2.430725	-0.716657
C	-2.249831	-1.720994	-0.619992
H	-3.174580	-2.150419	-0.209642
H	-2.294613	-1.867915	-1.709103
C	-2.153491	-0.222456	-0.296247
C	-3.162024	0.677613	-0.764893
H	-3.102704	0.069747	0.514539
C	-3.155377	2.175040	-0.622039
H	-4.168608	2.543564	-0.422717
H	-2.855678	2.581936	-1.601793
H	-2.467612	2.563217	0.124765
C	-4.319755	0.126124	-1.564031
H	-4.568435	-0.913163	-1.332568
H	-4.016157	0.156164	-2.623885
H	-5.213126	0.750023	-1.457279
H	1.065060	-2.692239	1.599057
H	1.104037	-1.206278	2.538483
H	-0.086194	0.019016	-0.458143
C	-1.440467	-1.475041	2.384541
H	-1.208034	-0.691357	3.117932
H	-2.520321	-1.417631	2.167482
H	-1.268032	-2.445053	2.869254

**P**	G781.169967		
C	-3.002921	0.444219	-1.616931
C	-3.206795	-0.994546	-1.075255
C	-1.904052	-1.663364	-0.714783
C	0.654038	1.472895	1.137418
C	-1.798732	1.142007	0.482059
C	-2.211814	1.437783	-0.779312
H	-3.728066	-1.562708	-1.863033
H	-3.885643	-0.966539	-0.212720
H	-3.994199	0.877691	-1.835843
H	-2.501543	0.389240	-2.601534
C	-1.944053	2.749056	-1.479227
H	-1.462027	3.493146	-0.836394
H	-2.893704	3.179708	-1.832928
H	-1.325240	2.609249	-2.380638
C	-0.845059	1.883549	1.378125
H	-2.149420	0.203837	0.901693
C	-1.551224	-2.165703	0.490103
H	-1.141013	-1.643105	-1.504375
C	-0.095566	-2.455400	0.799429
H	-0.001163	-3.318026	1.476218
H	0.449013	-2.711767	-0.120612
C	-2.497778	-2.331617	1.657537

H	-3.539167	-2.102003	1.412122
H	-2.208894	-1.703857	2.516567
H	-2.463966	-3.372318	2.014557
C	0.796492	-0.006645	0.579282
C	0.566154	-1.229597	1.496353
H	1.522487	-1.524936	1.953055
H	-0.074398	-0.900310	2.326163
C	1.268005	2.280975	-0.035624
H	1.612360	3.274918	0.274501
H	0.523589	2.414803	-0.829557
C	2.404231	1.392577	-0.564258
H	3.334775	1.549939	0.019591
H	2.708404	1.536541	-1.612663
C	1.986767	0.003196	-0.253003
C	2.725728	-1.179586	-0.760428
H	2.473623	-2.055765	-0.147390
C	2.199341	-1.456244	-2.211153
H	2.697176	-2.361087	-2.580716
H	2.445922	-0.625044	-2.883188
H	1.116044	-1.621369	-2.234161
C	4.259129	-0.987737	-0.775211
H	4.644501	-0.759811	0.226834
H	4.563550	-0.186542	-1.460469
H	4.730144	-1.917339	-1.116816
H	-0.906262	2.976638	1.267175
H	-1.106263	1.663532	2.423818
H	-0.052866	-0.017591	-0.197387
C	1.465717	1.665722	2.432440
H	1.108358	1.001228	3.229900
H	2.539056	1.471777	2.292808
H	1.359189	2.698634	2.790050

**P**	G781.169969		
C	-3.002926	0.444099	-1.616930
C	-3.206690	-0.994738	-1.075404
C	-1.903925	-1.663456	-0.714860
C	0.653906	1.472949	1.137434
C	-1.798817	1.141805	0.482142
C	-2.211922	1.437654	-0.779200
H	-3.727792	-1.562891	-1.863304
H	-3.885663	-0.966931	-0.212966
H	-3.994248	0.877483	-1.835825
H	-2.501527	0.389255	-2.601525
C	-1.944524	2.749111	-1.478914
H	-1.462010	3.493010	-0.836233
H	-2.894415	3.179918	-1.831797
H	-1.326371	2.609505	-2.380806
C	-0.845234	1.883423	1.378223
H	-2.149350	0.203539	0.901693
C	-1.551075	-2.165800	0.490021
H	-1.140831	-1.643100	-1.504395

C	-0.095395	-2.455442	0.799335
H	-0.000979	-3.318159	1.476012
H	0.449177	-2.711706	-0.120737
C	-2.497588	-2.332002	1.657436
H	-3.538763	-2.100780	1.412617
H	-2.207659	-1.705751	2.517195
H	-2.464984	-3.373227	2.013072
C	0.796494	-0.006684	0.579299
C	0.566240	-1.229667	1.496345
H	1.522575	-1.524940	1.953081
H	-0.074370	-0.900407	2.326123
C	1.267628	2.280973	-0.035741
H	1.611793	3.275045	0.274196
H	0.523105	2.414510	-0.829623
C	2.403956	1.392728	-0.564354
H	3.334416	1.550169	0.019634
H	2.708241	1.536788	-1.612707
C	1.986707	0.003302	-0.253062
C	2.725827	-1.179366	-0.760557
H	2.473602	-2.055592	-0.147642
C	2.200037	-1.455866	-2.211429
H	1.116713	-1.620808	-2.234955
H	2.697861	-2.360791	-2.580802
H	2.447061	-0.624675	-2.883316
C	4.259262	-0.987411	-0.774700
H	4.644204	-0.759531	0.227516
H	4.563880	-0.186157	-1.459792
H	4.730446	-1.916975	-1.116176
H	-0.906530	2.976509	1.267318
H	-1.106363	1.663346	2.423924
H	-0.052893	-0.017598	-0.197314
C	1.465599	1.665864	2.432389
H	1.108288	1.001343	3.229855
H	2.538956	1.471973	2.292759
H	1.359065	2.698756	2.790066

P-Q-TS G781.164621, T-700

C	-3.014360	0.289772	-1.607235
C	-3.114263	-1.169873	-1.091697
C	-1.772440	-1.738036	-0.702082
C	0.584868	1.531682	1.083545
C	-1.829255	1.015609	0.487166
C	-2.325655	1.329585	-0.730266
H	-3.568672	-1.768839	-1.897724
H	-3.810943	-1.205783	-0.243749
H	-4.031735	0.640267	-1.851201
H	-2.480602	0.287926	-2.576806
C	-2.266187	2.706718	-1.354952
H	-1.811085	3.459627	-0.701662
H	-3.285268	3.048925	-1.593364
H	-1.718736	2.700920	-2.311480

C	-0.922120	1.801708	1.396470
H	-2.027952	0.014941	0.851517
C	-1.416945	-2.219708	0.509345
H	-0.986765	-1.638292	-1.464607
C	0.046909	-2.392295	0.850491
H	0.198536	-3.247457	1.525119
H	0.628310	-2.595896	-0.057350
C	-2.375258	-2.472503	1.651296
H	-3.426122	-2.360885	1.368532
H	-2.187532	-1.800306	2.505760
H	-2.240283	-3.496928	2.030700
C	0.971884	0.074932	0.758798
C	0.623652	-1.132415	1.597837
H	1.557604	-1.423227	2.106503
H	-0.080106	-0.822778	2.380060
C	1.031268	2.287517	-0.199445
H	1.307509	3.326484	0.014454
H	0.196433	2.298260	-0.910173
C	2.181358	1.464633	-0.795040
H	3.154008	1.747471	-0.361224
H	2.283593	1.543128	-1.884028
C	1.901453	0.051794	-0.317914
C	2.740748	-1.124507	-0.779538
H	2.501325	-1.996718	-0.157412
C	2.470285	-1.474449	-2.257806
H	1.421025	-1.752849	-2.428192
H	3.096871	-2.323729	-2.555753
H	2.713113	-0.631579	-2.918306
C	4.234942	-0.792233	-0.544210
H	4.431360	-0.498197	0.495650
H	4.579939	0.010587	-1.207726
H	4.832145	-1.686326	-0.761539
H	-1.064728	2.891051	1.331924
H	-1.128175	1.525521	2.442070
H	0.603052	-0.133142	-0.538389
C	1.466628	1.924523	2.301823
H	1.222959	1.321217	3.185810
H	2.539908	1.815157	2.093696
H	1.275932	2.976724	2.549371

Q G781.166329

C	3.379990	-0.452966	-1.321653
C	3.412888	1.062637	-0.992107
C	2.020290	1.619646	-0.877011
C	-0.573146	-1.372607	1.003851
C	1.887720	-0.898255	0.655016
C	2.546674	-1.365523	-0.428835
H	3.972102	1.561790	-1.800729
H	3.979036	1.228770	-0.065710
H	4.416135	-0.830137	-1.357414
H	2.998828	-0.579480	-2.352099

C	2.537880	-2.809649	-0.878024
H	1.969149	-3.468526	-0.212940
H	3.569545	-3.191656	-0.924286
H	2.130062	-2.913547	-1.896193
C	0.871079	-1.592090	1.533279
H	2.057853	0.137373	0.932028
C	1.494982	2.309127	0.164037
H	1.333216	1.307326	-1.674471
C	0.001606	2.432310	0.276029
H	-0.294318	3.375050	0.754253
H	-0.486897	2.382901	-0.704584
C	2.293748	2.838493	1.333387
H	3.374028	2.793503	1.165655
H	2.079580	2.288246	2.265665
H	2.030603	3.889210	1.526513
C	-0.955918	0.006671	0.562041
C	-0.608447	1.272610	1.217157
H	-1.569949	1.683606	1.574218
H	0.037452	1.119250	2.088718
C	-0.921305	-2.251436	-0.230146
H	-1.119484	-3.291065	0.058779
H	-0.060858	-2.244345	-0.912480
C	-2.116923	-1.551496	-0.890628
H	-3.065314	-1.843147	-0.419052
H	-2.205801	-1.771677	-1.959721
C	-1.832264	-0.049937	-0.631976
C	-3.019020	0.953938	-0.749715
H	-2.618371	1.964667	-0.572251
C	-3.602072	0.922489	-2.171829
H	-2.836909	1.143118	-2.929120
H	-4.401520	1.667699	-2.270646
H	-4.037055	-0.059573	-2.402196
C	-4.106328	0.682214	0.306533
H	-3.715106	0.703780	1.333902
H	-4.587066	-0.292608	0.148579
H	-4.891087	1.445653	0.235574
H	1.022140	-2.679181	1.609779
H	0.951237	-1.197418	2.557727
H	-1.071149	0.261050	-1.397790
C	-1.613513	-1.650431	2.163605
H	-1.427110	-0.997545	3.024159
H	-2.655516	-1.533484	1.846617
H	-1.464986	-2.694068	2.471444

**Q**	G781.166337		
C	3.380431	-0.452753	-1.321472
C	3.412846	1.062896	-0.992061
C	2.020101	1.619546	-0.877012
C	-0.572769	-1.372538	1.003622
C	1.888335	-0.898129	0.655270
C	2.546945	-1.365325	-0.428812

H	3.971905	1.562169	-1.800720
H	3.978963	1.229301	-0.065695
H	4.416689	-0.829652	-1.356846
H	2.999630	-0.579481	-2.352017
C	2.537788	-2.809348	-0.878326
H	1.968938	-3.468264	-0.213381
H	3.569397	-3.191532	-0.924591
H	2.130026	-2.912998	-1.896535
C	0.871397	-1.591885	1.533236
H	2.058869	0.137353	0.932580
C	1.494672	2.309053	0.163939
H	1.333096	1.307004	-1.674445
C	0.001289	2.432161	0.275875
H	-0.294711	3.374971	0.753918
H	-0.487247	2.382524	-0.704713
C	2.293317	2.838615	1.333296
H	3.373599	2.794256	1.165390
H	2.079621	2.288023	2.265482
H	2.029632	3.889139	1.526715
C	-0.956106	0.006620	0.562173
C	-0.608757	1.272603	1.217278
H	-1.570189	1.683685	1.574363
H	0.037269	1.119333	2.088756
C	-0.921020	-2.251383	-0.230352
H	-1.118937	-3.291054	0.058572
H	-0.060677	-2.244078	-0.912816
C	-2.116891	-1.551615	-0.890543
H	-3.065154	-1.843517	-0.418863
H	-2.205916	-1.771729	-1.959642
C	-1.832523	-0.050076	-0.631797
C	-3.019287	0.953719	-0.749534
H	-2.618733	1.964433	-0.571733
C	-3.602084	0.922602	-2.171759
H	-2.836820	1.143605	-2.928836
H	-4.401652	1.667689	-2.270492
H	-4.036830	-0.059481	-2.402495
C	-4.106733	0.681660	0.306494
H	-3.715591	0.702974	1.333899
H	-4.587415	-0.293141	0.148244
H	-4.891505	1.445097	0.235632
H	1.022410	-2.678990	1.609781
H	0.951273	-1.197263	2.557722
H	-1.071330	0.261023	-1.397506
C	-1.613054	-1.650632	2.163523
H	-1.426836	-0.997775	3.024137
H	-2.655120	-1.534152	1.846597
H	-1.464027	-2.694228	2.471310

Q-R-TS G781.155484, T-357

C	3.497729	-0.488358	-1.234088
C	3.363102	1.055151	-1.128462

C	1.923858	1.506601	-1.046008
C	-0.273327	-1.179250	0.911480
C	2.100926	-0.772498	0.819306
C	2.713924	-1.337809	-0.242886
H	3.852638	1.482792	-2.018774
H	3.934405	1.413115	-0.261617
H	4.567443	-0.755626	-1.194020
H	3.166758	-0.805212	-2.240401
C	2.667394	-2.810189	-0.578478
H	2.149854	-3.414384	0.175647
H	3.692669	-3.200977	-0.668911
H	2.190341	-2.992229	-1.555250
C	1.016392	-1.360217	1.699641
H	2.252167	0.286343	0.990909
C	1.367230	2.265784	-0.078457
H	1.256161	1.069089	-1.799586
C	-0.139836	2.377731	0.041997
H	-0.424754	3.396950	0.343374
H	-0.627965	2.187509	-0.922461
C	2.142805	2.967076	1.015341
H	3.227839	2.894535	0.894408
H	1.893868	2.577734	2.017497
H	1.883724	4.036938	1.030946
C	-1.009433	0.014576	0.660128
C	-0.729330	1.421597	1.144191
H	-1.685740	1.843687	1.486419
H	-0.058774	1.405869	2.013663
C	-0.696329	-2.244001	-0.088896
H	-0.742873	-3.248079	0.352856
H	0.099368	-2.260083	-0.848506
C	-2.013689	-1.731528	-0.696867
H	-2.878283	-2.093149	-0.121510
H	-2.152794	-2.063750	-1.730514
C	-1.880817	-0.197399	-0.584413
C	-3.160368	0.655197	-0.777103
H	-2.848589	1.712665	-0.740330
C	-3.737101	0.395041	-2.182419
H	-2.975208	0.522800	-2.964340
H	-4.558092	1.091306	-2.394859
H	-4.140648	-0.623993	-2.265396
C	-4.249134	0.463760	0.295007
H	-3.944515	0.833688	1.284579
H	-4.558173	-0.587635	0.390380
H	-5.146085	1.031303	0.016228
H	1.143754	-2.428636	1.921704
H	0.982012	-0.820133	2.654825
H	-1.180498	0.115153	-1.387002
C	-1.685958	-0.925691	2.116882
H	-1.304059	-0.379493	2.977492
H	-2.719351	-0.674775	1.875167
H	-1.654823	-2.006780	2.292001

**R**	G781.160182		
C	3.484588	-0.492521	-1.202653
C	3.361908	1.051834	-1.107507
C	1.926692	1.504575	-1.013602
C	-0.125203	-1.232862	0.800355
C	2.017860	-0.814684	0.817402
C	2.667747	-1.352974	-0.257593
H	3.851363	1.460615	-2.006575
H	3.944463	1.411647	-0.249281
H	4.547720	-0.774757	-1.109734
H	3.203030	-0.819527	-2.221082
C	2.621990	-2.810374	-0.625753
H	2.174706	-3.450483	0.141842
H	3.641580	-3.172527	-0.825152
H	2.066368	-2.966044	-1.566530
C	1.000087	-1.512503	1.719693
H	2.108851	0.247319	1.005266
C	1.382121	2.244603	-0.024760
H	1.246622	1.063907	-1.753640
C	-0.121282	2.316259	0.144339
H	-0.413567	3.333901	0.446809
H	-0.632648	2.120278	-0.806357
C	2.175125	2.959173	1.047788
H	3.259554	2.874700	0.926314
H	1.920201	2.602181	2.059488
H	1.925796	4.031427	1.034791
C	-1.093982	-0.082751	0.870659
C	-0.649451	1.358965	1.258682
H	-1.539815	1.826131	1.701864
H	0.078733	1.300414	2.082512
C	-0.578440	-2.214256	-0.242459
H	-0.546539	-3.248429	0.131691
H	0.142255	-2.177574	-1.075014
C	-1.952701	-1.711504	-0.735346
H	-2.756447	-2.181444	-0.153689
H	-2.126034	-1.958547	-1.788748
C	-1.881335	-0.195201	-0.481587
C	-3.207123	0.606634	-0.615176
H	-3.126437	1.494307	0.036340
C	-3.366805	1.113307	-2.062747
H	-2.536383	1.768883	-2.360773
H	-4.300058	1.679430	-2.179681
H	-3.401976	0.270574	-2.769311
C	-4.475427	-0.167181	-0.202938
H	-4.423999	-0.594823	0.805995
H	-4.680565	-0.989886	-0.902527
H	-5.343732	0.503676	-0.230772
H	1.182481	-2.583105	1.865052
H	0.927067	-0.998940	2.684540
H	-1.184981	0.210003	-1.237949
C	-1.962736	-0.599780	2.100516

H	-1.404673	-0.482579	3.035932
H	-2.858153	0.029872	2.142071
H	-2.286597	-1.643165	2.012425

**R**	G837.684874		
C	-3.746842	1.161374	-0.879882
C	-3.962524	-0.374269	-0.676442
C	-2.693735	-1.166877	-0.895403
C	0.352094	0.120906	0.305899
C	-1.922596	1.034209	0.774261
C	-2.438520	1.681545	-0.285782
H	-4.734086	-0.697667	-1.394202
H	-4.376653	-0.559634	0.323285
H	-4.625830	1.706114	-0.497755
H	-3.717014	1.377288	-1.960813
C	-1.770537	2.816678	-1.022403
H	-0.819125	3.113311	-0.564444
H	-2.430891	3.698383	-1.018294
H	-1.591497	2.576309	-2.081949
C	-0.481763	0.882279	1.222345
H	-2.560499	0.321743	1.295480
C	-2.034407	-1.955306	-0.010794
H	-2.233607	-1.031170	-1.881921
C	-0.706348	-2.602416	-0.369349
H	-0.844191	-3.698874	-0.350399
H	-0.418173	-2.359202	-1.399739
C	-2.565775	-2.293605	1.367254
H	-3.632046	-2.072267	1.477019
H	-2.034448	-1.750944	2.166415
H	-2.428593	-3.364800	1.577778
C	1.300819	-0.959517	0.667371
C	0.440232	-2.342241	0.635870
H	1.219027	-3.107093	0.506288
H	0.042762	-2.468500	1.650823
C	0.450579	0.434167	-1.145431
H	0.728967	1.501081	-1.212459
H	-0.535321	0.382522	-1.624715
C	1.544999	-0.479090	-1.733028
H	2.165814	0.060317	-2.457713
H	1.098104	-1.321144	-2.274469
C	2.356793	-0.973666	-0.507259
C	3.637789	-0.136328	-0.236864
H	4.061616	-0.493283	0.716110
C	4.700463	-0.409460	-1.318442
H	4.939742	-1.479578	-1.383460
H	5.628955	0.130627	-1.090426
H	4.366335	-0.078090	-2.312060
C	3.386920	1.376608	-0.099770
H	2.617244	1.615755	0.649924
H	3.070839	1.819143	-1.056082
H	4.309450	1.889233	0.202704

H	0.051946	1.914349	1.267614
H	-0.424313	0.488841	2.245111
H	2.686925	-2.012094	-0.664483
C	1.897075	-0.889546	2.089102
H	2.567209	-1.743353	2.251059
H	2.471811	0.029427	2.246193
H	1.113630	-0.936462	2.855109
N	0.746732	3.594900	1.617633
H	-0.048065	4.221464	1.750328
H	1.257554	3.576029	2.501091
H	1.361009	4.035234	0.932116

R-S-TS G837.679993, T-1023

C	-3.757797	1.142709	-0.882422
C	-3.952955	-0.400192	-0.697547
C	-2.671880	-1.174806	-0.924781
C	0.355326	0.141467	0.308826
C	-1.938485	1.028930	0.777757
C	-2.441701	1.660626	-0.303214
H	-4.725145	-0.724167	-1.414209
H	-4.361753	-0.599682	0.301848
H	-4.634246	1.674072	-0.475347
H	-3.749785	1.375704	-1.959851
C	-1.765899	2.773263	-1.066036
H	-0.800913	3.066602	-0.632851
H	-2.413108	3.665230	-1.066160
H	-1.601193	2.517072	-2.123547
C	-0.530907	0.760025	1.213941
H	-2.620717	0.376135	1.326638
C	-1.989348	-1.942813	-0.042845
H	-2.225445	-1.036573	-1.917299
C	-0.649608	-2.569704	-0.407982
H	-0.779803	-3.666897	-0.406564
H	-0.370173	-2.308729	-1.436644
C	-2.502645	-2.285909	1.340095
H	-3.567179	-2.064472	1.466872
H	-1.956756	-1.748808	2.133150
H	-2.363690	-3.358316	1.544407
C	1.341554	-0.936119	0.654405
C	0.512063	-2.317556	0.593541
H	1.283142	-3.085139	0.432948
H	0.120803	-2.483775	1.606381
C	0.441669	0.458460	-1.151129
H	0.669567	1.531255	-1.257605
H	-0.541392	0.332263	-1.624883
C	1.573235	-0.414831	-1.735447
H	2.178342	0.148509	-2.455764
H	1.158333	-1.269007	-2.283596
C	2.398723	-0.895847	-0.513383
C	3.656746	-0.024668	-0.238577
H	4.098338	-0.383251	0.705769

C	4.720910	-0.241713	-1.330712
H	4.989270	-1.303088	-1.420317
H	5.635893	0.318880	-1.096427
H	4.371636	0.100472	-2.315408
C	3.362602	1.477884	-0.071669
H	2.607100	1.665557	0.706833
H	3.001964	1.924086	-1.009994
H	4.274586	2.017810	0.215556
H	0.072010	1.955648	1.431519
H	-0.486367	0.331027	2.224015
H	2.765141	-1.920006	-0.685280
C	1.942617	-0.871844	2.072271
H	2.629807	-1.713832	2.227183
H	2.502746	0.054743	2.243355
H	1.162186	-0.944455	2.840554
N	0.478113	3.237148	1.828492
H	-0.346426	3.839546	1.853348
H	0.888762	3.208707	2.763334
H	1.162352	3.642665	1.188040

**S**	G837.685581		
C	-3.774698	1.197706	-0.811980
C	-3.972729	-0.352751	-0.681600
C	-2.696539	-1.126626	-0.943087
C	0.354145	0.152458	0.332221
C	-1.940784	1.038368	0.833886
C	-2.438068	1.679661	-0.251818
H	-4.755744	-0.646779	-1.399714
H	-4.372522	-0.582471	0.314900
H	-4.635059	1.715472	-0.354963
H	-3.800012	1.475245	-1.878284
C	-1.748814	2.776067	-1.025089
H	-0.765504	3.045532	-0.615084
H	-2.373972	3.684277	-1.010750
H	-1.605501	2.521986	-2.085861
C	-0.576377	0.637483	1.225553
H	-2.661827	0.439436	1.399791
C	-2.010171	-1.915947	-0.084895
H	-2.257594	-0.963395	-1.935029
C	-0.669906	-2.534555	-0.469052
H	-0.809559	-3.629726	-0.510806
H	-0.391830	-2.235953	-1.487904
C	-2.519063	-2.296039	1.289755
H	-3.580634	-2.068880	1.431218
H	-1.961090	-1.789224	2.093704
H	-2.389025	-3.375675	1.459834
C	1.332765	-0.967431	0.639688
C	0.503576	-2.330292	0.537892
H	1.252427	-3.113873	0.347800
H	0.108753	-2.531966	1.544090
C	0.432374	0.494053	-1.131867

H	0.653304	1.565522	-1.257212
H	-0.549593	0.345243	-1.603911
C	1.565670	-0.369508	-1.732338
H	2.175190	0.207499	-2.438748
H	1.149965	-1.208634	-2.302972
C	2.388626	-0.890847	-0.525336
C	3.657000	-0.039258	-0.233451
H	4.104458	-0.430545	0.694994
C	4.710949	-0.223460	-1.341272
H	4.965433	-1.283686	-1.473758
H	5.634933	0.316587	-1.093184
H	4.357900	0.159388	-2.309455
C	3.380004	1.460339	-0.011540
H	2.657851	1.611972	0.806160
H	2.980513	1.934764	-0.919938
H	4.304818	1.988513	0.256907
H	0.262508	2.135216	1.506785
H	-0.515006	0.224412	2.240083
H	2.752281	-1.909927	-0.731512
C	1.946268	-0.947017	2.052822
H	2.628509	-1.797288	2.186775
H	2.515543	-0.030133	2.251052
H	1.168377	-1.035280	2.822889
N	0.591470	3.166860	1.779833
H	-0.209903	3.797613	1.685569
H	0.931007	3.181983	2.745831
H	1.344934	3.460339	1.150912

**S**	G837.685583		
C	-3.774781	1.197308	-0.812654
C	-3.972517	-0.353287	-0.682575
C	-2.696128	-1.127037	-0.943312
C	0.354019	0.153032	0.332333
C	-1.941244	1.038398	0.833696
C	-2.438344	1.679444	-0.252248
H	-4.755016	-0.647364	-1.401221
H	-4.372906	-0.583117	0.313661
H	-4.635295	1.714656	-0.355450
H	-3.800190	1.475137	-1.878861
C	-1.748931	2.775970	-1.025167
H	-0.765704	3.045233	-0.614826
H	-2.374028	3.684221	-1.010604
H	-1.605502	2.522217	-2.085998
C	-0.576816	0.637760	1.225474
H	-2.662248	0.439400	1.399579
C	-2.010034	-1.916036	-0.084596
H	-2.256774	-0.964103	-1.935120
C	-0.669532	-2.534648	-0.467959
H	-0.809038	-3.629856	-0.509195
H	-0.391127	-2.236537	-1.486863
C	-2.519486	-2.295817	1.289936

H	-3.581072	-2.068464	1.430985
H	-1.961735	-1.788985	2.094024
H	-2.389684	-3.375449	1.460214
C	1.332712	-0.966766	0.640091
C	0.503541	-2.329614	0.539318
H	1.252399	-3.113441	0.350250
H	0.108289	-2.530365	1.545540
C	0.432326	0.494453	-1.131760
H	0.653515	1.565849	-1.257312
H	-0.549709	0.345889	-1.603765
C	1.565329	-0.369497	-1.732205
H	2.174932	0.207219	-2.438789
H	1.149339	-1.208583	-2.302686
C	2.388314	-0.890845	-0.525232
C	3.657123	-0.039681	-0.233962
H	4.104595	-0.430676	0.694601
C	4.710803	-0.224949	-1.341871
H	4.964940	-1.285343	-1.473700
H	5.634996	0.314980	-1.094314
H	4.357677	0.157358	-2.310240
C	3.380755	1.460142	-0.012831
H	2.658664	1.612534	0.804785
H	2.981375	1.934230	-0.921452
H	4.305770	1.988130	0.255265
H	0.263037	2.135095	1.507050
H	-0.515440	0.224765	2.240048
H	2.751527	-1.910122	-0.731231
C	1.946558	-0.945535	2.053089
H	2.628829	-1.795714	2.187380
H	2.515886	-0.028538	2.250662
H	1.168837	-1.033361	2.823388
N	0.591743	3.166696	1.780562
H	-0.210400	3.796872	1.689014
H	0.933681	3.180990	2.745727
H	1.343387	3.461596	1.150128

S-S-TS G837.654426, T-130

C	-3.890668	0.757292	-0.868428
C	-3.870224	-0.817848	-0.773843
C	-2.539090	-1.519061	-1.040551
C	0.477662	0.222399	0.307420
C	-1.823650	0.863942	0.381125
C	-2.905405	1.562354	0.003647
H	-4.617982	-1.170886	-1.502279
H	-4.245124	-1.091720	0.220652
H	-4.921945	1.070444	-0.638308
H	-3.726468	1.059215	-1.920015
C	-3.222586	3.014791	0.255643
H	-2.491284	3.486210	0.929768
H	-4.210624	3.127242	0.727170
H	-3.268314	3.591125	-0.683938

C	-0.500408	0.842439	1.064929
H	-1.896476	-0.139671	-0.004453
C	-1.798015	-2.224289	-0.143979
H	-2.103144	-1.328791	-2.028885
C	-0.355727	-2.643147	-0.421292
H	-0.323279	-3.747187	-0.423125
H	-0.054555	-2.339268	-1.431733
C	-2.312259	-2.538027	1.247113
H	-3.396760	-2.691822	1.265221
H	-2.088269	-1.720578	1.953223
H	-1.842422	-3.447542	1.645613
C	1.477710	-0.816854	0.736396
C	0.703562	-2.235349	0.664082
H	1.516582	-2.976142	0.623750
H	0.228710	-2.363001	1.646239
C	0.551865	0.369441	-1.201682
H	0.588533	1.439260	-1.472432
H	-0.362956	-0.003432	-1.682002
C	1.823678	-0.387422	-1.658478
H	2.427813	0.220274	-2.343374
H	1.551071	-1.291673	-2.216103
C	2.597507	-0.761857	-0.363981
C	3.783309	0.197619	-0.061478
H	4.198754	-0.096225	0.916322
C	4.912561	0.020444	-1.093201
H	5.246187	-1.024990	-1.139701
H	5.780725	0.639782	-0.829660
H	4.594976	0.314856	-2.103851
C	3.384642	1.683212	0.044377
H	2.635294	1.839324	0.835321
H	2.980140	2.052473	-0.912725
H	4.259126	2.300840	0.288482
H	0.114323	2.391734	0.754050
H	-0.478521	0.711142	2.154382
H	3.039057	-1.765585	-0.469378
C	1.984985	-0.700219	2.184994
H	2.673736	-1.522256	2.421695
H	2.514238	0.242858	2.365203
H	1.150882	-0.756395	2.896976
N	0.345068	3.469450	0.482934
H	-0.367831	3.799697	-0.172837
H	0.330636	4.054476	1.322325
H	1.273008	3.510892	0.051003

**S**	G837.700073		
C	-3.940948	1.007577	-1.036271
C	-4.005764	-0.570730	-1.017927
C	-2.649306	-1.236148	-1.113198
C	0.518558	0.261932	-0.009072
C	-1.638097	1.389677	-0.339387
C	-2.951046	1.443721	0.019135

H	-4.634583	-0.879695	-1.867363
H	-4.539359	-0.882545	-0.109792
H	-4.951788	1.407472	-0.868476
H	-3.608630	1.333330	-2.033951
C	-3.455155	1.579365	1.429607
H	-2.660902	1.769902	2.161617
H	-4.001153	0.677289	1.743844
H	-4.177029	2.409052	1.489821
C	-0.547847	0.939595	0.522135
H	-1.446129	1.294909	-1.411978
C	-1.940071	-1.807267	-0.112464
H	-2.185204	-1.209620	-2.106619
C	-0.573304	-2.439095	-0.363426
H	-0.717121	-3.533935	-0.315670
H	-0.242052	-2.237609	-1.391618
C	-2.453879	-1.938661	1.304107
H	-3.529213	-1.752852	1.388312
H	-1.946861	-1.236007	1.984389
H	-2.259033	-2.947600	1.698420
C	1.354335	-0.770915	0.724529
C	0.550357	-2.154280	0.678684
H	1.305390	-2.945731	0.567890
H	0.112912	-2.302739	1.676022
C	0.894235	0.253975	-1.479593
H	1.126189	1.273953	-1.835167
H	0.048102	-0.082691	-2.098266
C	2.121630	-0.683845	-1.601818
H	2.880768	-0.273448	-2.278689
H	1.813541	-1.646531	-2.030810
C	2.651703	-0.893357	-0.158927
C	3.832560	0.045848	0.217260
H	4.062523	-0.136102	1.280098
C	5.099344	-0.321299	-0.578368
H	5.358576	-1.380436	-0.446702
H	5.956169	0.279215	-0.243494
H	4.973220	-0.139973	-1.655419
C	3.534815	1.550326	0.072980
H	2.690431	1.846060	0.713145
H	3.303346	1.811579	-0.972353
H	4.406098	2.147066	0.374723
H	0.465232	2.449290	0.328658
H	-0.747612	0.832326	1.591642
H	3.047617	-1.915837	-0.053178
C	1.625208	-0.459751	2.207693
H	2.241979	-1.248832	2.659023
H	2.146649	0.495162	2.350761
H	0.687196	-0.418575	2.777945
N	0.670443	3.500997	0.096157
H	-0.130332	3.838621	-0.447519
H	0.757529	4.044600	0.959139
H	1.536454	3.573954	-0.445558

**S**	G837.699320		
C	-3.907590	0.985060	-0.932500
C	-3.951813	-0.597455	-0.954747
C	-2.596055	-1.247125	-1.134348
C	0.554980	0.189080	0.010462
C	-1.588159	1.322695	-0.246316
C	-2.908330	1.394239	0.120387
H	-4.617337	-0.883127	-1.784337
H	-4.442475	-0.939128	-0.033548
H	-4.919510	1.369062	-0.739533
H	-3.590170	1.337402	-1.925962
C	-3.389070	1.499607	1.539655
H	-2.585614	1.706017	2.256559
H	-3.889130	0.568183	1.847461
H	-4.146210	2.294017	1.627437
C	-0.504760	0.805759	0.589426
H	-1.418713	1.212925	-1.322469
C	-1.844132	-1.870464	-0.196733
H	-2.171770	-1.163071	-2.142326
C	-0.487022	-2.477213	-0.546363
H	-0.631523	-3.572696	-0.579991
H	-0.199077	-2.195481	-1.568995
C	-2.297018	-2.092632	1.228002
H	-3.356100	-1.864442	1.385007
H	-1.718108	-1.475973	1.933079
H	-2.133786	-3.139106	1.527953
C	1.414926	-0.870491	0.672953
C	0.690594	-2.281471	0.459025
H	1.468883	-3.003808	0.172812
H	0.332976	-2.611565	1.444948
C	0.935702	0.281028	-1.460077
H	1.121227	1.326778	-1.761634
H	0.118606	-0.071688	-2.108600
C	2.214598	-0.583306	-1.613473
H	2.955562	-0.104792	-2.265436
H	1.961584	-1.543597	-2.083350
C	2.738077	-0.823432	-0.173726
C	3.796252	0.218357	0.291087
H	4.016437	0.000642	1.349097
C	5.117617	0.040373	-0.480529
H	5.491841	-0.988866	-0.396115
H	5.891897	0.713542	-0.087463
H	4.999388	0.266227	-1.550085
C	3.342365	1.691404	0.218585
H	2.421845	1.850464	0.804523
H	3.162299	2.001512	-0.822430
H	4.117124	2.354228	0.626963
H	-0.825407	3.029988	-0.132282
H	-0.696224	0.652269	1.653439
H	3.236145	-1.804501	-0.110371
C	1.628791	-0.683536	2.185259

H	2.298642	-1.461125	2.577601
H	2.065801	0.292419	2.430918
H	0.679221	-0.770953	2.731025
N	0.040117	3.669223	-0.051384
H	0.183596	4.217136	-0.904486
H	-0.049102	4.295788	0.753780
H	0.826656	3.017900	0.100310

S-T-TS G837.696534, T-26

C	-3.871048	0.465133	-1.032456
C	-3.748103	-1.109023	-0.998099
C	-2.322036	-1.601735	-1.113676
C	0.653115	0.277060	-0.016267
C	-1.609698	1.065369	-0.325620
C	-2.941379	1.033498	0.019382
H	-4.350130	-1.498265	-1.833930
H	-4.226270	-1.472207	-0.078163
H	-4.921759	0.748467	-0.871305
H	-3.577740	0.814026	-2.035205
C	-3.446063	1.086898	1.442143
H	-2.696324	1.463036	2.150510
H	-3.745786	0.086323	1.787617
H	-4.347528	1.715073	1.517802
C	-0.467518	0.823222	0.530978
H	-1.411509	1.000295	-1.398924
C	-1.528579	-2.074368	-0.123447
H	-1.880491	-1.520760	-2.114282
C	-0.096223	-2.523873	-0.399887
H	-0.105756	-3.628724	-0.367753
H	0.189850	-2.268988	-1.429514
C	-1.995039	-2.270255	1.300846
H	-3.083088	-2.212858	1.409225
H	-1.552795	-1.516683	1.971103
H	-1.674614	-3.251120	1.683080
C	1.610058	-0.640668	0.718641
C	1.007795	-2.120565	0.627399
H	1.858729	-2.794984	0.455116
H	0.629066	-2.374310	1.627680
C	1.040140	0.320047	-1.485165
H	1.148825	1.366285	-1.811043
H	0.261510	-0.122909	-2.125205
C	2.381701	-0.450821	-1.589796
H	3.082819	0.047308	-2.270378
H	2.208438	-1.454839	-2.001263
C	2.924117	-0.559022	-0.141243
C	3.912024	0.578240	0.243196
H	4.126424	0.466057	1.319064
C	5.254576	0.400946	-0.491432
H	5.693351	-0.586989	-0.294297
H	5.977527	1.162065	-0.166892
H	5.138913	0.504884	-1.580187

C	3.372893	2.004065	0.023947
H	2.403776	2.166543	0.517389
H	3.245338	2.220208	-1.047198
H	4.081209	2.744546	0.420105
H	-2.251396	2.871398	-0.112307
H	-0.634523	0.715325	1.605360
H	3.482044	-1.501372	-0.016212
C	1.810570	-0.313430	2.208526
H	2.547588	-0.995023	2.655089
H	2.161077	0.714067	2.362739
H	0.874181	-0.438555	2.770227
N	-2.051749	3.950665	0.072980
H	-2.179667	4.495452	-0.784848
H	-2.677361	4.308102	0.800709
H	-1.079498	4.030159	0.387395

**T**	G837.708403		
C	-3.860694	0.171184	-0.797918
C	-3.590906	-1.356684	-0.785469
C	-2.149139	-1.657650	-1.088254
C	0.772260	0.309927	-0.040994
C	-1.562510	0.893297	-0.222571
C	-2.958042	0.952936	0.204146
H	-4.239223	-1.815236	-1.549996
H	-3.902131	-1.776458	0.179769
H	-4.912343	0.372873	-0.549838
H	-3.689286	0.563827	-1.813295
C	-3.241294	0.641420	1.677526
H	-2.682450	1.312852	2.342445
H	-2.979509	-0.391610	1.939473
H	-4.309195	0.778060	1.888409
C	-0.439349	0.617333	0.561415
H	-1.398524	1.093480	-1.285565
C	-1.227351	-2.238163	-0.276990
H	-1.810221	-1.349540	-2.085415
C	0.208908	-2.422312	-0.752361
H	0.333722	-3.502521	-0.945623
H	0.352722	-1.938181	-1.728322
C	-1.530773	-2.775886	1.098087
H	-2.601815	-2.842608	1.311325
H	-1.071944	-2.146237	1.878172
H	-1.099896	-3.780523	1.223227
C	1.811122	-0.571082	0.594562
C	1.364999	-2.076873	0.242697
H	2.262381	-2.573593	-0.147465
H	1.133490	-2.562224	1.199787
C	1.181255	0.645263	-1.452617
H	1.202580	1.741488	-1.558373
H	0.445037	0.288239	-2.189029
C	2.584399	0.017735	-1.640526
H	3.238554	0.662540	-2.237935

H	2.497533	-0.934658	-2.182350
C	3.122043	-0.228945	-0.207154
C	3.964204	0.942310	0.368252
H	4.181973	0.688755	1.419166
C	5.324106	1.033473	-0.351270
H	5.871868	0.082411	-0.300735
H	5.950913	1.808884	0.108945
H	5.205985	1.298073	-1.412023
C	3.261727	2.311484	0.355876
H	2.273131	2.290113	0.839238
H	3.126158	2.683580	-0.670203
H	3.868425	3.055236	0.888798
H	-3.263809	2.041515	0.018997
H	-0.578948	0.375708	1.615769
H	3.775398	-1.115967	-0.192370
C	1.938074	-0.451974	2.123044
H	2.754686	-1.092926	2.480855
H	2.148055	0.575799	2.440416
H	1.021232	-0.783232	2.628815
N	-4.234612	3.734992	-0.116609
H	-4.796062	3.813645	-0.965340
H	-4.880780	3.840268	0.666427
H	-3.623663	4.552457	-0.096776

NH_3 G56.537076

N	-0.000000	-0.000000	0.123483
H	0.000000	0.935817	-0.288128
H	-0.810441	-0.467909	-0.288128
H	0.810441	-0.467909	-0.288128

References

[1] L. Lauterbach, B. Goldfuss, J. S. Dickschat, *Angew. Chem. Int. Ed.* **2020**, *59*, 11943-11947; *Angew. Chem.* **2020**, *132*, 12041-12045.

[2] A. Hou, J. S. Dickschat, *Angew. Chem. Int. Ed.* **2020**, *59*, 19961-19965; *Angew. Chem.* **2020**, *132*, 20135-20140.

[3] P. Rabe, J. S. Dickschat, *Angew. Chem. Int. Ed.* **2013**, *52*, 1810-1812; *Angew. Chem.* **2013**, *125*, 1855-1857.

[4] Z. Yin, Z. Hu, K. Yang, G. Lu, G. B. Tabekoueng, P. Wu, Q. Luo, J. Xu, B. Goldfuss, J. S. Dickschat, G. Bian, *Angew. Chem. Int. Ed.* **2025**, *64*, e202507752; *Angew. Chem.* **2025**, *137*, e202507752.

[5] X. Wei, W. Ning, C. A. McCadden, T. A. Alsup, Z. Li, D. P. Lomowska-Keehner, J. Nafie, T. Qu, M. O. Opoku, G. R. Gillia, B. Xu, D. G. Icenhour, J. D. Rudolf, *Nat. Commun.* **2025**, *16*, 3721.

[6] J. Rinkel, J. S. Dickschat, *Beilstein J. Org. Chem.* **2019**, *15*, 789-794.

[7] G. B. Tabekoueng, H. Li, B. Goldfuss, G. Schnakenburg, J. S. Dickschat, *Angew. Chem. Int. Ed.* **2024**, *63*, e202413860; *Angew. Chem.* **2024**, *136*, e202413860.

[8] J. S. Dickschat, K. A. K. Pahirulzaman, P. Rabe, T. A. Klapschinski, *ChemBioChem* **2014**, *15*, 810-814.

[9] R. D. Gietz, R. H. Schiestl, *Nat. Protoc.* **2007**, *2*, 31-34.

[10] M. M. Bradford, *Anal. Biochem.* **1976**, *72*, 248-254.

[11] G. R. Fulmer, A. J. M. Miller, N. H. Sherden, H. E. Gottlieb, A. Nudelman, B. M. Stoltz, J. E. Bercaw, K. I. Goldberg, *Organometallics* **2010**, *29*, 2176-2179.

[12] X. Lin, R. Hopson, D. E. Cane, *J. Am. Chem. Soc.* **2006**, *128*, 6022-6023.

[13] H. Xu, J. Rinkel, J. S. Dickschat, *Org. Chem. Front.* **2021**, *8*, 1177-1184.

[14] L. Lauterbach, J. Rinkel, J. S. Dickschat, *Angew. Chem. Int. Ed.* **2018**, *57*, 8280-8283.

[15] P. Rabe, J. Rinkel, E. Dolja, T. Schmitz, B. Nubbemeyer, T. H. Luu, J. S. Dickschat, *Angew. Chem. Int. Ed.* **2017**, *56*, 2776-2779.

[16] J. Rinkel, J. S. Dickschat, *Org. Lett.* **2019**, *21*, 2426-2429.

[17] Z. Quan, J. S. Dickschat, *Org. Lett.* **2020**, *22*, 7552-7555.

[18] T. Lou, A. Li, H. Xu, J. Pan, B. Xing, R. Wu, J. S. Dickschat, D. Yang, M. Ma, *J. Am. Chem. Soc.* **2023**, *145*, 8474-8485.

[19] J. Rinkel, P. Rabe, X. Chen, T. G. Köllner, F. Chen, J. S. Dickschat, *Chem. Eur. J.* **2017**, *23*, 10501-10505.

[20] Z. Quan, J. S. Dickschat, *Org. Biomol. Chem.* **2020**, *18*, 6072-6076.

[21] H. Li, J. S. Dickschat, *Angew. Chem. Int. Ed.* **2022**, *61*, e202211054; *Angew. Chem.* **2022**, *134*, e202211054.

[22] S. Grimme, S. Ehrlich, L. Goerigk, *J. Comp. Chem.* **2011**, *32*, 1456-1465.

[23] Gaussian 16, Revision C.01, M. J. Frisch, G. W. Trucks, H. B. Schlegel, G. E. Scuseria, M. A. Robb, J. R. Cheeseman, G. Scalmani, V. Barone, G. A. Petersson, H. Nakatsuji, X. Li, M. Caricato, A. V. Marenich, J. Bloino, B. G. Janesko, R. Gomperts, B. Mennucci, H. P. Hratchian, J. V. Ortiz, A. F. Izmaylov, J. L. Sonnenberg, D. Williams-Young, F. Ding, F. Lipparini, F. Egidi, J. Goings, B. Peng, A. Petrone, T. Henderson, D. Ranasinghe, V. G. Zakrzewski, J. Gao, N. Rega, G. Zheng, W. Liang, M. Hada, M. Ehara, K. Toyota, R. Fukuda, J. Hasegawa, M. Ishida, T. Nakajima, Y. Honda, O. Kitao, H. Nakai, T. Vreven, K. Throssell, J. A. Montgomery, Jr., J. E. Peralta, F. Ogliaro, M. J. Bearpark, J. J. Heyd, E. N. Brothers, K. N. Kudin, V. N. Staroverov, T. A. Keith, R. Kobayashi, J. Normand, K. Raghavachari, A. P. Rendell, J. C. Burant, S. S. Iyengar, J. Tomasi, M. Cossi, J. M. Millam, M. Klene, C. Adamo, R. Cammi, J. W. Ochterski, R. L. Martin, K. Morokuma, O. Farkas, J. B. Foresman, and D. J. Fox, Gaussian, Inc., Wallingford CT, 2019.

[24] S. Grimme, *Chem. Eur. J.* **2012**, *18*, 9955-9964.

[25] GoodVibes v3.0.1, G. Luchini, J. V. Alegre-Requena, Y. Guan, I. Funes-Ardoiz, R. S. Paton, 2019.

[26] N. Mardirossian, M. Head-Gordon, *J. Chem. Phys.* **2016**, *144*, 214110.
[27] F. Weigend, R. Ahlrichs, *Phys. Chem. Phys.* **2005**, *7*, 3297.
[28] a) F. Neese, *Comput. Mol. Sci.* **2025**, *15*, e70019; b) F. Neese, F. Wennmohs, A. Hansen, U. Becker, *Chem. Phys.* **2009**, *356*, 98-109.
[29] a) M. Bursch, J.-M. Mewes, A. Hansen, S. Grimme, *Angew. Chem. Int. Ed.* **2022**, *61*, e202205735; b) S. Zev, P. K. Gupta, E. Pahima, D. T. Major, *J. Chem. Theo. Comput.* **2022**, *18*, 167-178.
[30] P. Pracht, F. Bohle, S. Grimme, *Phys. Chem. Chem. Phys.* **2020**, *22*, 7169-7192.
[31] S. Grimme, *J. Chem. Theory Comput.* **2019**, *155*, 2847-2862.
[32] P. Pracht, S. Grimme, *Chem. Sci.* **2021**, *12*, 6551-6568.
[33] P. Pracht, C.A. Bauer, S. Grimme, *J. Comput. Chem.* **2017**, *38*, 2618-2631.
[34] S. Spicher, C. Plett, P. Pracht, A. Hansen, S. Grimme, *J. Chem. Theory Comput.* **2022**, *18*, 3174-3189.
[35] R. Wang, Y. Chen, M. Shu, W. Zhao, M. Tao, C. Du, X. Fu, A. Li, Z. Lin, *Chem. Eur. J.* **2020**, *26*, 1941-1946.
[36] Z. Q. Yuan, H. Ishikawa, D. L. Boger, *Org. Lett.* **2005**, *7*, 741-744.
[37] H. Li, B. Goldfuss, J. S. Dickschat, *Angew. Chem. Int. Ed.* **2025**, *64*, e202422788.
